# Diastereodivergent nucleophile–nucleophile alkene chlorofluorination

**DOI:** 10.1038/s41557-024-01561-6

**Published:** 2024-07-01

**Authors:** Sayad Doobary, Andrew J. D. Lacey, Stephen G. Sweeting, Sarah B. Coppock, Henry P. Caldora, Darren L. Poole, Alastair J. J. Lennox

**Affiliations:** 1 University of Bristol, Bristol, UK; 2 Discovery High-Throughput Chemistry, Medicinal Chemistry, GSK Medicines Research Centre, Stevenage, UK

**Keywords:** Synthetic chemistry methodology, Reaction mechanisms, Synthetic chemistry methodology

## Abstract

The selective hetero-dihalogenation of alkenes provides useful building blocks for a broad range of chemical applications. Unlike homo-dihalogenation, selective hetero-dihalogenation reactions, especially fluorohalogenation, are underdeveloped. Current approaches combine an electrophilic halogen source with a nucleophilic halogen source, which necessarily leads to *anti*-addition, and regioselectivity has only been achieved using highly activated alkenes. Here we describe an alternative, nucleophile–nucleophile approach that adds chloride and fluoride ions over unactivated alkenes in a highly regio-, chemo- and diastereoselective manner. A curious switch in the reaction mechanism was discovered, which triggers a complete reversal of the diastereoselectivity to promote either *anti-* or *syn*-addition. The conditions are demonstrated on an array of pharmaceutically relevant compounds, and detailed mechanistic studies reveal the selectivity and the switch between the *syn*- and *anti*-diastereomers are based on different active iodanes and which of the two halides adds first.

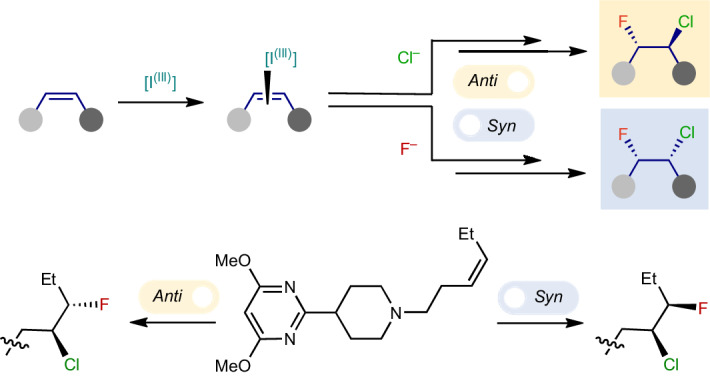

## Abstract (web summary)

Unlike homo-dihalogenation, selective hetero-dihalogenation reactions are underdeveloped. Now an oxidative alkene hetero-dihalogenation reaction adds chloride and fluoride ions over unactivated alkenes with high regio-, chemo- and diastereoselectivity. A switch in the mechanism triggers a reversal of the diastereoselectivity to promote either *anti-* or *syn*-addition.

## Main

**Fig. 1:**
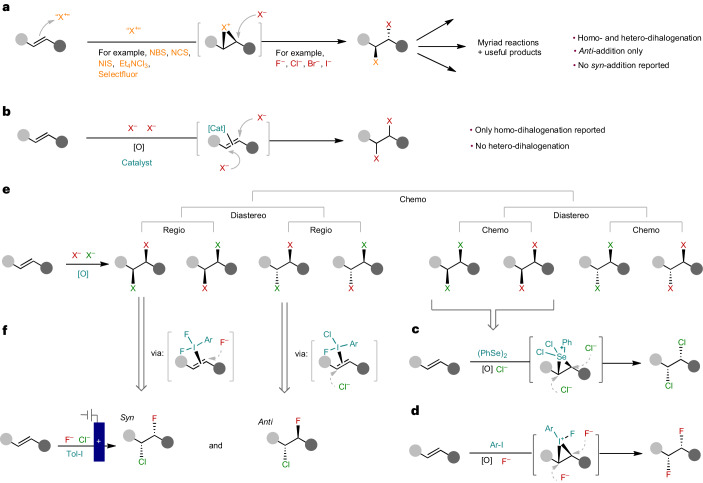
Approaches to alkene dihalogenation and the challenges in selective hetero-dihalogenation. **a**, The classical E–Nu approach for homo- and hetero-dihalogenation. **b**, The alternative Nu–Nu approach, reported for homo-dihalogenation. **c**, Nu–Nu *syn*-dichlorination from Denmark^10^. **d**, Nu–Nu *syn*-difluorination from Yoneda, Jacobsen, Gilmour and Lennox^11–14^. **e**, The selectivity challenge with hetero-dihalogenation using the Nu–Nu approach. **f**, Regio, chemo- and diastereo (*syn* and *anti*)-selective chlorofluorination of unactivated alkenes. Previously reported methods for this transformation use the E–Nu approach to this reaction, give only *anti*-addition and have limited scope.

Alkene dihalogenation is foundational to the field of organic synthesis—transforming a ubiquitous functional group into higher-value building blocks—from which decades of innovation and development have been built. This classic reaction continues to enjoy widespread use in contemporary organic synthesis, especially in the construction of complex and asymmetric scaffolds^1–3^. While alkene homo-dihalogenation is extensively exploited in this context, hetero-dihalogenation provides more complex, desymmetrized products that are equipped with two handles for further regioselective functionalization. The redox neutral ‘electrophile–nucleophile’ (‘E–Nu’) approach, in which an alkene attacks an electrophilic halogen, followed by attack by a nucleophilic halide, represents the only strategy towards the synthesis of these groups^4–7^ (Fig. 1a). While this approach is effective, the intermediate formation of a halonium ion stereospecifically renders an *anti*-arrangement of halides. Indeed, no *syn* hetero-dihalogenation has been reported.

An alternative approach involves only adding nucleophilic halide sources for the dihalogenation, which both attack the alkenyl carbon atoms (Fig. 1b). This oxidative ‘nucleophile–nucleophile’ (‘Nu–Nu’) strategy has the benefit of halogen sources existing in nature as halides (X^–^) and, hence, exhibit innate nucleophilic reactivity. Correspondingly, their cost and associated energy and waste produced in manufacturing nucleophilic halogen reagents is lower^8^. Nevertheless, the most intriguing opportunity with this strategy is to depart from a mechanism that necessarily proceeds through a halonium intermediate. This ‘Nu–Nu’ approach has been successfully achieved for homo-dihalogenation through, for example, a radical mechanism^9^ or in combination with alkene-activation catalysts to instigate stereospecific *syn-*dichloride or difluoride addition^10–15^ (Fig. 1c,d).

Adopting a ‘Nu–Nu’ strategy for the hetero-dihalogenation of alkenes is intrinsically more problematic and, as such, is notably absent from literature. The use of two different nucleophiles of similar reactivity introduces chemo-, regio- and diastereoselectivity issues, all of which need to be controlled (Fig. 1e). Nevertheless, we were drawn to attempt this challenge with fluoride and chloride (Fig. 1f) for several reasons: (1) Through its small size, high charge density and strong bonds to carbon, fluorine imparts desirable physicochemical properties to molecules through unique stereoelectronic effects and conformational control^16^. These effects enable the elaboration of bioisosteric moieties^17^ and have increased the demand for fluorine-containing building blocks and bioactive molecules^18,19^. (2) Chlorine is increasingly prevalent in bioactive molecules, due to the so-called ‘magic chloro effect’^20^, which bears resemblance to the well-established ‘magic methyl effect’^21^. (3) There is a relative scarcity of alkene chlorofluorination methods compared with other hetero-dihalogenation reactions^6^. In this Article, we document the development of the ‘Nu–Nu’ approach for alkene hetero-dihalogenation, in which excellent chemo-, regio- and diastereoselectivity is achieved for the chlorofluorination of unactivated alkenes. A simple diastereoselectivity switch was discovered to direct either *anti-* or *syn*-addition of the two halides in a regio- and chemoselective manner.

## Results and discussion

**Fig. 2:**
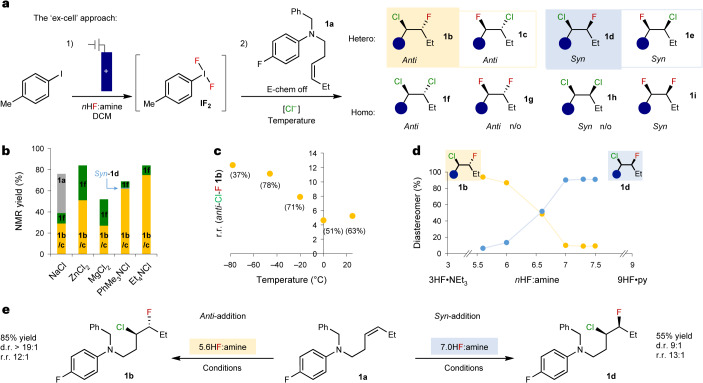
Reaction optimization. For full details, see Supplementary Tables 1–5. **a**, Challenges with Nu−Nu chlorofluorination to control chemo-, regio- and diastereoselectivity. Reaction of model compound **1a** to products **1b**–**i** (n/o, not observed) using the ‘ex-cell’ electrochemical approach. **b**, Chemoselectivity with different chloride sources. **c**, Temperature dependence on regioselectivity for *anti*-addition. **d**, Diastereoselectivity switch with changing *n*HF:amine ratio. **e**, A summary of the diastereoselectivity switch. **IF**_**2**_ generation: *p*-iodotoluene in 5.6HF:amine and DCM (13 mA, 2.2 F, divided cell, Pt||Pt). *Anti* conditions: alkene (0.6 mmol), **IF**_**2**_ (1 equiv.) solution in 5.6HF:amine, NEt_4_Cl (1 equiv., 0.2 equiv. h^−1^), DCM, −46 °C, 16 h; *syn* conditions: alkene (0.6 mmol), **IF**_**2**_ (1 equiv.) solution in 5.6HF:amine adjusted to 7HF:amine, NEt_4_Cl (1 equiv., 0.2 equiv. h^−1^), DCM, −46 °C, 16 h.

Our strategy drew inspiration from our electrochemical hypervalent iodine-mediated *syn*-difluorination of alkenes^14^, where two fluorides sequentially invert a proposed iodonium intermediate (Fig. 1d). Electrochemical oxidation of iodotoluene provides a controllable and sustainable method for the generation of the difluoro(tolyl)-λ^3^-iodane (**IF**_**2**_) mediator. Switching the electrochemical oxidation off and then adding in the substrate (‘ex-cell approach’) was found to better facilitate tolerance to oxidatively sensitive substrates that contain electron-rich functionality^14,22–24^. This is because there is no residual oxidant in solution to decompose the substrate. Wishing to exploit the same electron-rich chemical space, we adopted the ‘ex-cell’ electrochemical method for generating **IF**_**2**_ and deliberately chose oxidatively sensitive **1a** as the model substrate (Fig. 2a). This substrate deliberately contains an unactivated acyclic internal alkene, which is an underexplored alkene-type in fluoro- or chloro-functionalization reactions^25–30^. Adapting our difluorination conditions by adding an excess of various R_4_N^+^ chloride salts to a solution of **1a** and **IF**_**2**_ in 5.6HF:amine (1:1 (v/v) mixture of 3HF•NEt_3_ and 9HF•py) in dichloromethane (DCM)–hexafluoroisopropanol at room temperature led predominately to alkene dichlorination. Without hexafluoroisopropanol, the use of 1 equiv. of chloride provided more selective conditions but, surprisingly, not for the expected *syn*-addition product, **1d** or **1e**, rather to the *anti*-addition product, **1b**. Nevertheless, we observed six out of the eight possible products (**1g** and **1h** were not observed) (Fig. 2a), confirming the substantial challenge of controlling chemo-, regio-, and diastereoselectivity in the reaction.

A range of different chloride salts were tested (Fig. 2b); chloride with inorganic cations led to more dichlorination, and more soluble organic cations led to greater selectivity for chlorofluorination, with NEt_4_Cl giving the highest yield (Supplementary Table 1). The regioselectivity of the *anti*-addition product could be improved by lowering the temperature, with −46 °C (CO_2_(s) in MeCN) providing the best balance of selectivity and yield (Fig. 2c). By adding chloride slowly, the competing dichlorination could be attenuated, leading to an optimized 85% yield of the *anti*-addition product **1b** with a regioisomeric ratio (r.r.) of 12:1 (Fig. 2e).

During these efforts, product **1d** from *syn*-chlorofluorination was only observed in trace quantities (<5%). However, when we started to increase the *n*HF:amine ratio beyond 5.6 (by adding 9HF•py to the 5.6HF•amine mixture), the diastereoselectivity started to shift. A range of *n*HF:amine ratios were tested (Fig. 2d), which revealed the mechanism could be flipped with this highly sensitive trigger; increasing the ratio from just *n* = 5.6 to just 7 was sufficient to completely switch the diastereoselectivity, yielding the *syn-*addition product **1d** in good yield and excellent r.r. (Fig. 2e). Although the selectivity enhancement was maintained at ratios above 7HF:amine, the yield dropped, and therefore, optimized conditions for the *syn*-chlorofluorination of internal, unactivated alkenes remained with 7HF:amine (Supplementary Table 4).

To explore the generality of the reaction, a wide selection of alkene substrates was probed under the conditions (Table 1). Terminal alkenes transformed efficiently under the *anti*-addition 5.6HF:amine conditions, giving good to excellent yields and selectivity for the 1-chloro-2-fluoro products (*n***j**). Oxidizable functionalities, such as secondary and tertiary amines, alcohols, anilines and styrenes and more complex molecules, were all well tolerated. Remarkably, the expected 1-chloro-2-fluoro (*n***j**) regioselectivity was not observed for the cinchonine **11a**, as the 1-fluoro-2-chloro regioisomer **11k** preferentially formed, which is probably due to the internal position being sterically more inaccessible than all other substrates.

**Table 1:**
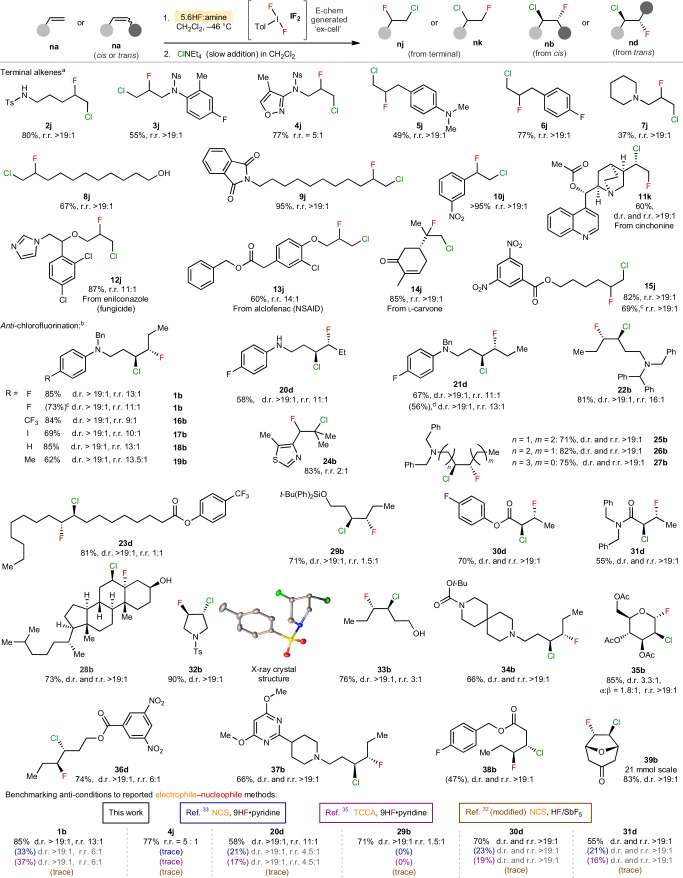
Substrate scope for terminal alkenes and *anti*-addition into unactivated internal alkenes

Geometry of alkene starting material is depicted into the drawn conformation of the product. The isolated yields are given, and the NMR yields are in parentheses. The reactions were conducted at a 0.6 mmol scale, unless otherwise stated. r.r. (*n***b**:*n***c** or *n***d**:*n***e**); d.r., diastereomeric ratio of isolated compound.

^a^Cl^–^ addition rate of 0.16 equiv. min^−1^.

^b^Cl^–^ addition rate of 0.16 equiv. h^−1^.

^c^Use of PIFA in place of electrochemically generated **IF**_**2**_; the data given are the mean average of two runs.

The *anti*-chlorofluorination conditions were then successfully applied to a broad range of internal alkenes, including *cis* and *trans* acyclic and cyclic alkenes, as well as substituted and electron-poor alkenes (Table 1). Although oxidants (Selectfluor and *meta*-chloroperoxybenzoic acid) previously used for **IF**_**2**_ formation were found to be inferior (Supplementary Table 8), we found that commercially available (bis(trifluoroacetoxy)iodo)benzene (PIFA) led to only a small drop in yield (73% versus 85% for **1b**), which represents a practical alternative to electrochemically generated **IF**_**2**_. Oxidizable and acid-sensitive (**29b**, **34b** and **35b**) functional groups were well tolerated, and the yields were good to excellent in all cases. High regioselectivity was observed with fluoride placed on the site best able to stabilize a positive charge, hence, further away from electron-withdrawing groups. Exquisite regioselectivity was observed even four bonds away from a tertiary amine (**27b**). When there are competing factors for positive charge stabilization (**24b**) or the alkene is more remote (**23d**), then the regioselectivity decreases or disappears. Biologically relevant compounds were also transformed, including glucal derivative **35b** and cholesterol **28b**. Finally, a multigram scale-up of **39b** was successfully demonstrated.

Previously reported chlorofluorination conditions are ‘E–Nu’ methods that combine an *N*-chloro electrophilic chlorine reagent (*N*-chlorosuccinimide (NCS)^31–34^, trichloroisocyanuric acid (TCCA)^35^, *N*-chlorosaccharin^36^) with a source of HF^37^, and all lead to exclusive *anti*-addition. With few exceptions^32^, these conditions are demonstrated on limited compound classes, for example, styrenes, and without complex functionality, especially that which is easily oxidized. Hence, we were intrigued to test the complementarity to our ‘Nu–Nu’ system on substrates containing more varied functionality and alkene-types (Table 1). In all cases, isolated yields from our ‘Nu–Nu’ conditions proved superior to the nuclear magnetic resonance (NMR) yields from reported procedures, including both *cis* and *trans* internal alkenes, electron-poor alkenes and terminal alkenes. The regioselectivity either matched or was superior to the reported conditions.

The scope of the alkene *syn*-chlorofluorination reaction was then probed (Table 2). Various hetero-cyclic and aliphatic homo-allylic amines afforded the desired products in moderate to very good yields, with excellent tolerance for oxidatively sensitive functional groups. *Cis* alkenes underwent the *syn*-addition with generally higher efficiency than *trans* alkenes (**40d** versus **40b**). When the yields are moderate, oxidative decomposition probably competes. The *anti*-addition pathway was strongly attenuated under these conditions, which ensured the diastereoselectivity was excellent throughout. The regioselectivity was also excellent, with an overwhelming preference for the chloride to be placed nearest to nitrogen. Finally, ester **38a** also underwent the *syn-*chlorofluorination.

**Table 2:**
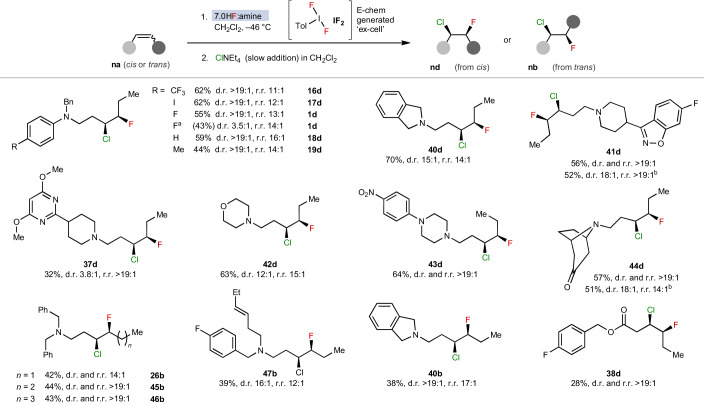
Substrate scope of *syn*-chlorofluorination

Isolated yields; r.r. (*n***d**:*n***e** or *n***b**:*n***c**); d.r., diastereomeric ratio of isolated compound. The geometry of alkene starting material is depicted in the conformation of the product.

^a^Use of PIFA in place of electrochemically generated **IF**_**2**_; the data given are the mean average of two runs.

^b^Addition of HBF_4_•Et_2_O instead of additional 9HF•py.

**Fig. 3:**
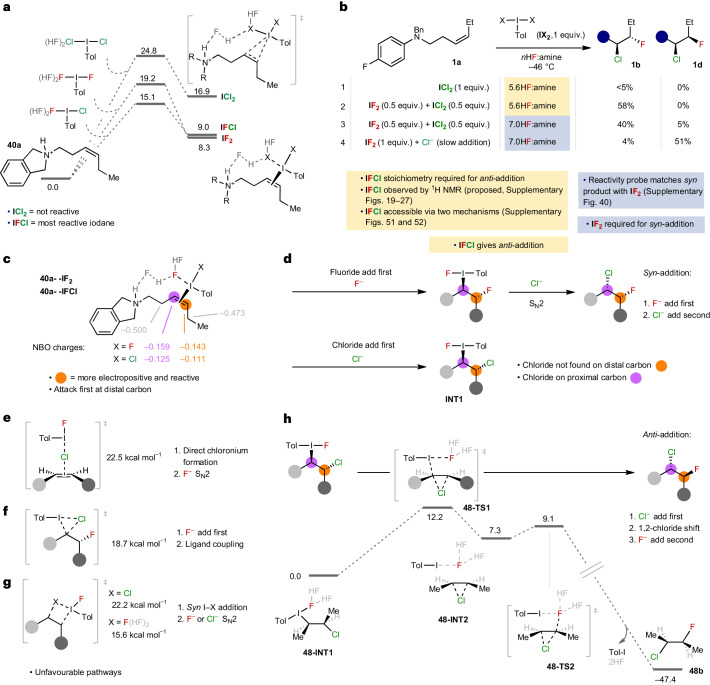
Mechanistic studies for *syn-* and *anti-*addition, addressing the active iodane and transition state. **a**, DFT calculations modelled at −46 °C of iodine(III)–π complex formation, showing **IFCl** is the most reactive. Level of theory: M06-2X/6-31 + G(d)/LANL2DZ(I) + SMD(CH_2_Cl_2_)//M06-2X/def2-TZVP + SMD(CH_2_Cl_2_). **b**, Reactivity studies using preformed samples of **IF**_**2**_ and **ICl**_**2**_ to establish the active iodane under each set of conditions. *Anti*-addition to **1b** is not observed with **ICl**_**2**_ alone but is with 50:50 **IF**_**2**_:**ICl**_**2**_, providing evidence for **IFCl** to be the active iodane for *anti*-addition. *Syn*-addition to **1d** does not predominate in the presence of **ICl**_**2**_ and only forms with **IF**_**2**_, providing evidence for **IF**_**2**_ to be the active iodane for *syn*-addition. **c**, Natural Bond Orbital (NBO) calculations (DFT) of iodine(III)–π complex to establish regioselectivity of nucleophile attack. **d**, Consideration of which halide attacks first. For *syn*-addition, fluoride attacks first and for *anti*-addition, chloride attacks first. **e**–**g**, *Anti*-addition mechanisms discounted due to unfavourable transition state energies. The energies refer to the following starting materials: **40a** in **e**, *cis*-but-2-ene in **f**, **40a** in **g**. **h**, DFT calculations for the proposed mechanism for *anti*-addition, which shows a favourable transition state energy for a 1,2-chloride shift.

To rationalize the synthetic results and, in particular, the origin for the regioselectivity and the intriguing switch in diastereoselectivity, we conducted a series of mechanistic experiments. Using **40a** as a model substrate, alkene activation with iodane was calculated to occur most favourably by forming an iodine(III)–π complex, as opposed to the commonly invoked iodonium intermediate (Supplementary Fig. 42)^38^. To identify the specific iodane species responsible for each mechanism, we calculated energetic barriers for iodine(III)–π complex formation (Fig. 3a). **IFCl** was found to have the lowest energy barrier for alkene activation, whereas the transition state with **ICl**_**2**_ is completely inaccessible at −46 °C. The enhanced reactivity of **IFCl** over **IF**_**2**_ and **ICl**_**2**_ was also supported by charge and orbital coefficient calculations (Supplementary Fig. 48 and Supplementary Table 18). These findings were consistent with experimental reactivity studies using preformed iodanes (Fig. 3b). When a sample of **ICl**_**2**_ was applied to **1a** under the *anti* conditions (Fig. 3b1), only trace product **1b** was formed, confirming that **ICl**_**2**_ cannot be an active iodane and a more reactive species is required. However, when a 50:50 mixture of **ICl**_**2**_ and **IF**_**2**_ was used in the reaction the reactivity switched back on and product **1b** formed readily (Fig. 3b2). These stoichiometries support **IFCl** to be responsible for *anti*-addition, which is notable considering fluoro-chloro-aryl iodanes have extremely limited presence in literature, with only one report proposing it as a potential intermediate^39^, in contrast to aryl dichloroiodanes, which are established reagents for alkene dichlorination^15,40–42^. Speciation studies (^1^H NMR; Supplementary Figs. 26 and 27) of **IF**_**2**_ with added NEt_4_Cl (0–1 equiv.) and **IF**_**2**_ mixed with **ICl**_**2**_, conducted at −46 °C, revealed the appearance of a new species that we propose is consistent with the formation of **IFCl**. Density functional theory (DFT) calculations modelled at −46 °C also demonstrated **IFCl** was readily accessible from either **IF**_**2**_ or **ICl**_**2**_ via two possible mechanisms (Supplementary Figs. 51 and 52 and Supplementary Scheme 5).

Under *syn*-conditions, the active iodane cannot be **IFCl**, considering *anti*-addition predominated with a 50:50 mixture of **ICl**_**2**_ and **IF**_**2**_ (Fig. 3b3). *Syn*-addition occurred only when **IF**_**2**_ was used with slow addition of chloride (Fig. 3b4), indicating **IF**_**2**_ to be the active species. As it is established *syn*-difluorination occurs through **IF**_**2**_^12,13,38^, we reasoned the levels of difluorination (in the absence of chloride) should mirror those of *syn*-chlorofluorination (in the presence of chloride) when the *n*HF:amine ratio is altered. Indeed, a direct match of products **1i** and **1d** is observed (Supplementary Fig. 40), with 7HF:amine giving the highest yields of both products, suggesting **IF**_**2**_ to be the active iodane for *syn*-chlorofluorination. An explanation for the current limitation of *syn*-chlorofluorination to homo-allylic amines was revealed by DFT calculations of iodine(III)–π complex formation with **IF**_**2**_ (Supplementary Fig. 49); while a barrier of 19.2 kcal mol^−1^ was calculated for homo-allylic amine, which is approaching the limit of accessibility at −46 °C, a barrier of 23.3 kcal mol^−1^ was calculated for the corresponding bis-homo allyl amine, which is inaccessible.

To understand the regioselectivity, we undertook natural population analysis calculations (Fig. 3c). A clear difference in charge distribution between the alkenyl carbons is indicated, with the carbon distal to nitrogen more electropositive and, therefore, more reactive towards nucleophilic attack. Transition state calculations predict fluoride and chloride attack onto activated alkene **48a–IF**_**2**_ to be rapid and facile (Supplementary Fig. 47). Hence, we propose *syn*-addition occurs when fluoride attacks first, followed by a subsequent chloride attack (Fig. 3d).

Formation of the *anti*-addition product is less obvious. Although chloride attack onto the more electropositive distal carbon occurs very readily and with a low barrier to form **INT1** (Fig. 3c and Supplementary Fig. 47), this was initially discounted because it is not consistent with the observed major regioisomer, which places chloride on the proximal carbon. Several inferred mechanisms in literature were considered, including direct chloronium formation, that is, alkene attack of a ‘Cl^+^’ equivalent (Fig. 3e)^41,43^, *syn-*ligand-coupling with fluoride attacking first (Fig. 3f)^44^ and *syn* I–X addition followed by fluoride or chloride attack (Fig. 3g)^45–48^. In each case, we considered different chlorinated or fluorinated iodanes and coordinated HF environments (Supplementary Figs. 44–47). Of these pathways, only the *syn* I–F addition pathway (Fig. 3g) was found to be energetically feasible. However, this pathway was discounted, because the competing chloride attack on the iodine(III)–π complex to form the chlorinated-iodanated intermediate (**INT1**) is far more favourable (Supplementary Fig. 47). A kinetically accessible transition state from **INT1** was located for a 1,2-chloride shift with Brønsted acid (HF) activation of the fluoride nucleofuge (Fig. 3h and Supplementary Fig. 43). Incipient chloronium formation through displacement of the iodane (from **INT1** to **INT2**) is followed by very rapid and exergonic attack by fluoride (**TS2**). Although this pathway for chloronium formation has been offered as a potential mechanism for alkene dihalogenation^4^, to the best of our knowledge, no examples with theoretical or experimental evidence have been reported. Hence, our proposed pathway for *anti*-addition is consistent with the observed regio-, chemo-, and diastereoselectivity, the barrier height is consistent with the observed reaction rates, and it is the only pathway that can explain the formation of each isomer of compound **35b** (Supplementary Figs. 36–39).

**Fig. 4:**
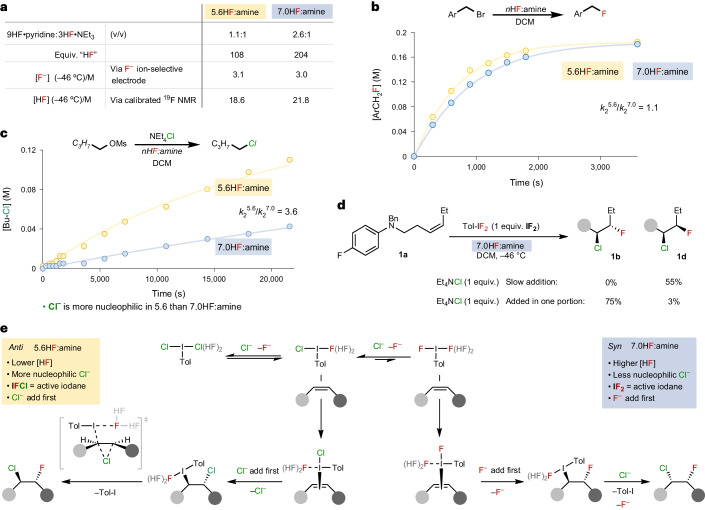
Mechanistic studies that focus on characterizing the differences between 5.6HF:amine and 7.0HF:amine and the diastereodivergence trigger. **a**, Analysis of the physical characteristics of each medium, which do not show a substantial difference between them. **b**, Assessment of the difference in nucleophilicity of fluoride in 5.6HF:amine and 7.0HF:amine by measuring the kinetics of the fluorination of *p*-nitrobenzyl bromide in each medium. The lines through plotted data are modelled second order fits. **c**, Assessment of the difference in nucleophilicity of chloride in 5.6HF:amine and 7.0HF:amine by measuring the kinetics of a chlorination reaction in each medium, which shows a lower nucleophilicity in 7.0HF:amine. The lines through plotted data are modelled second order fits. **d**, A diastereoselectivity switch can be achieved by controlling the concentration of chloride. **e**, A summary of the diastereodivergent Nu–Nu alkene chlorofluorination mechanisms. The bifurcation of mechanisms is dependent on the concentration and the relative nucleophilic activity of chloride and fluoride ions, which in turn dictates the structure and reactivity of the iodane, which halide adds first to the alkene, and the mechanism of iodane displacement.

Since the identity of the halide that attacks the iodine(III)–π complex first is diastereo-determining, we were inspired to understand how the reaction conditions differed to facilitate this. Hence, several fundamental physical characteristics were measured of the 5.6 and 7.0HF:amine solutions, including the concentrations of fluoride (F^−^) and HF (Fig. 4a). Despite distinct reaction outcomes under each set of conditions, only the equivalents of HF substantially differed. However, when the number of equivalents of HF in 5.6HF:amine were matched to that of 7.0HF:amine (that is, to 204), no *syn*-chlorofluorination was observed (Supplementary Table 13). Therefore, it cannot solely be the identity of the iodane and manipulation of the equilibrium between **ICl**_**2**_, **IFCl** and **IF**_**2**_ that dictates the diastereoselectivity.

The relative nucleophilicities of chloride and fluoride were next compared under both sets of conditions by measuring bimolecular nucleophilic substitution displacement rates in appropriately chosen transformations. The rate of reaction between *p*-nitrobenzyl bromide and fluoride proceeded at similar rates in both HF:amine solutions (Fig. 4b), indicating that fluoride has a similar nucleophilicity under each conditions. However, when chloride competes with fluoride in the substitution of *n*-butyl mesylate under both sets of conditions, the rate of chlorination was found to be 3.6 times faster in 5.6HF:amine compared with 7.0HF:amine, and no fluorinated product was observed (Fig. 4c). Nucleophilicity calculations of chloride and fluoride ion clusters also mirror these experimental observations (Supplementary Figs. 53–55). Combined, these data suggest that the dampened nucleophilicity of chloride in 7.0HF:amine promotes *syn*-chlorofluorination by allowing fluoride to add first, but in 5.6HF:amine, chloride has higher nucleophilicity and promotes *anti*-chlorofluorination by adding first.

Increasing the chloride concentration in 7.0HF:amine, via a single portion addition at the reaction outset, reversed the product outcome back to *anti*-addition product **1b** (Fig. 4d). This evidence adds further support to the diastereodivergence being controlled by which nucleophile attacks first; if chloride is in sufficiently high concentration or is sufficiently nucleophilic, then the more reactive **IFCl** is formed, and chloride can attack the alkene first, resulting in *anti*-chlorofluorination via a 1,2-chloride shift. Otherwise, fluoride adds first to an **IF**_**2**_-activated alkene and *syn*-chlorofluorination is achieved, following nucleophilic substitution by chloride (Fig. 4e).

In summary, we have developed a Nu–Nu strategy for the chlorofluorination of unactivated alkenes, which selectively gives either *anti-* or *syn*-addition. Good to excellent yields of products, including those that are electron-rich and readily oxidizable, are provided with very high regio-, chemo- and diastereoselectivity. A simple switch was discovered for transitioning between *anti*-and *syn*-chlorofluorination based on the HF:amine ratio used in the solution. Mechanistic studies revealed that different iodanes promote each pathway but that the identity of the halide adding to the alkene first is diastereo-determining, with fluoride leading to *syn*-addition and chloride leading to *anti*-addition. The *anti*-addition pathway follows an unusual 1,2-chloride shift followed by rapid fluoride addition from iodane. These results represent an important advance in the application of hypervalent iodine for the vital elaboration of fluorinated motifs in an ever-expanding chemical landscape, and show how capitalizing on a subtle and simple variation of reaction solvent composition can influence product selectivity.

## Online content

Any methods, additional references, Nature Portfolio reporting summaries, source data, extended data, supplementary information, acknowledgements, peer review information; details of author contributions and competing interests; and statements of data and code availability are available at 10.1038/s41557-024-01561-6.

## Supplementary information

Supplementary Information Supplementary discussion, Figs. 1–55, Tables 1–18 and Schemes 1–5.

Supplementary Data 1 Crystallographic data for compound **32b** (CCDC reference 2235848).

Supplementary Data 2 Eight files of *xyz* coordinates: **1,2_chloride_shift.docx** Cartesian coordinates of model alkene forming *anti*-chlorofluoride through 1,2-chloride shift via chloronium cation. **alkene_activation.docx** Cartesian coordinates of I(III)–alkene complexes and complexation transition states. **direct_chloronium_formation_transition_states.docx** Cartesian coordinates of direct Cl^+^ delivery to alkene transition states. **iodane_ligand_exchange.docx** Cartesian coordinates of iodanes **IF**_**2**_, **IFCl** and **ICl**_**2**_ and ligand exchange transition states between them with different sites and extents of HF coordination. **iodine(III)iranium_vs_iodine(III)-π_complex.docx** Cartesian coordinates of iodine(III)iranium and iodine(III)–π complex with model homoallylic amine showing latter is favoured thermodynamically. **isolated_fluoride_chloride_hf_clusters.docx** Cartesian coordinates of fluoride and chloride with 0–6 HF coordinated to anions. **ligand_coupling_transition_states.docx** Cartesian coordinates of ligand coupling of fluoride or chloride from C–I(III) intermediates. ***syn*-1,2-halo-λ3-iodanation.docx** Cartesian coordinates of alkene *syn*-difunctionalisation to form C–I(III) and C–X (X = F or Cl).

## Data Availability

All data, including experimental procedures, compound characterizations and mechanistic studies are available in the main text or the Supplementary Information. Crystallographic data for **32b** have been deposited at the Cambridge Crystallographic Data Centre, under deposition number CCDC 2235848. Copies of the data can be obtained free of charge via https://www.ccdc.cam.ac.uk/structures/.
