# Supplementary material for: Diastereodivergent nucleophile–nucleophile alkene chlorofluorination
Source: Nat Chem. 2024 Jul 1;16(10):1647–55. doi: 10.1038/s41557-024-01561-6 (PMC11446824; doi:10.1038/s41557-024-01561-6)

nature chemistry

Supplementary information https://doi.org/10.1038/s41557-024-01561-6

Diastereodivergent nucleophile–nucleophile alkene chlorofluorination

In the format provided by the authors and unedited

Table of Contents

General experimental **8**

Synthesis of standard: all products observed during optimization **11**

(Z)-N-benzyl-4-fluoro-N-(hex-3-en-1-yl)aniline, 1a-cis 11
(E)-N-benzyl-4-fluoro-N-(hex-3-en-1-yl)aniline, 1a-trans 13
N-benzyl-N-((3S,4S)-3-chloro-4-fluorohexyl)-4-fluoroaniline, 1b 15
N-benzyl-N-((3S,4R)-3-chloro-4-fluorohexyl)-4-fluoroaniline, 1d 17
N-benzyl-N-((3S,4S)-3,4-dichlorohexyl)-4-fluoroaniline, 1f 18
N-benzyl-N-((3S,4R)-3,4-dichlorohexyl)-4-fluoroaniline, 1h 19
N-benzyl-N-((3S,4S)-3,4-difluorohexyl)-4-fluoroaniline, 1g 20
N-benzyl-N-((3S,4R)-3,4-difluorohexyl)-4-fluoroaniline, 1i 22
Spectroscopic differences between chlorofluoride diastereomers by ^{19}F NMR 24

Optimisation **26**

Variation of chloride source **27**

Variation of temperature **28**

Variation of Chloride addition rate **29**

Variation of HF:amine ratio **30**

Variation of acid additive **31**

Synthesis of substrates **32**

Characterisation of substrates **34**

Terminal alkene substrates **34**

4-Methyl-N-(pent-4-en-1-yl)benzenesulfonamide, 2a 34
N-allyl-N-(4-methylisoxazol-3-yl)-4-nitrobenzenesulfonamide, 4a 35
(S)-quinolin-4-yl((1S,2S,4S,5R)-5-vinylquinuclidin-2-yl)methyl acetate, 11a 37
Benzyl 2-(4-(allyloxy)-3-chlorophenyl)acetate, 13a 38
Hex-5-en-1-yl-3,5-dinitrobenzoate, 15a 39

Internal alkene substrates **40**

(Z)-N-benzyl-N-(hex-3-en-1-yl)-4-(trifluoromethyl)aniline, 16a 40
(Z)-N-benzyl-N-(hex-3-en-1-yl)-4-iodoaniline, 17a 41
(Z)-N-benzyl-N-(hex-3-en-1-yl)aniline, 18a 42
(Z)-N-benzyl-N-(hex-3-en-1-yl)-4-methylaniline, 19a 43
(E)-4-fluoro-N-(hex-3-en-1-yl)aniline, 20a 44
4-(Trifluoromethyl)phenyl (E)-octadec-9-enoate, 23a 45
5-Methyl-4-(2-methylprop-1-en-1-yl)thiazole, 24a 46
(Z)-N,N-dibenzylhex-2-en-1-amine, 25a 47
(Z)-N,N-dibenzylhex-3-en-1-amine, 26a-cis 48
(Z)-N,N-dibenzylhex-4-en-1-amine, 27a 49
(Z)-tert-butyl(hex-3-en-1-yloxy)diphenylsilane, 29a 50
4-Fluorophenyl (E)-but-2-enoate, 30a 51
(E)-N,N-dibenzylbut-2-enamide, 31a 52
1-Tosyl-2,5-dihydro-1H-pyrrole, 32a 53
tert-Butyl-(Z)-9-(hex-3-en-1-yl)-3,9-diazaspiro[5.5]undecane-3-carboxylate, 34a 54
(E)-hex-3-en-1-yl 3,5-dinitrobenzoate, 36a 55
(Z)-2-(1-(Hex-3-en-1-yl)piperidin-4-yl)-4,6-dimethoxypyrimidine, 37a 56

4-fluorobenzyl (Z)-hex-3-enoate, 38a-cis 57
4-fluorobenzyl (E)-hex-3-enoate, 38a-trans 58
(Z)-2-(hex-3-en-1-yl)isoindoline, 40a-cis 59
(E)-2-(hex-3-en-1-yl)isoindoline, 40a-trans 60
(Z)-6-fluoro-3-(1-(hex-3-en-1-yl)piperidin-4-yl)benzo[d]isoxazole, 41a 61
(Z)-4-(hex-3-en-1-yl)morpholine, 42a 62
(Z)-1-(hex-3-en-1-yl)-4-(4-nitrophenyl)piperazine. 43a 63
(1R,5S)-8-((E)-hex-3-en-1-yl)-8-azabicyclo[3.2.1]octan-3-one, 44a 64
(E)-N,N-Dibenzylhex-3-en-1-amine, 26a-trans 65
(E)-N,N-dibenzyloct-3-en-1-amine, 45a 66
(E)-N,N-dibenzylnon-3-en-1-amine, 46a 67
(E)-N-(4-fluorobenzyl)-N-((E)-hex-3-en-1-yl)hex-3-en-1-amine, 47a 68

Synthesis of products **69**

Electrochemical synthesis of *p*-TolIF$_2$ **69**

Chlorofluorination procedure 1 (for terminal alkenes) **69**

***Anti*-chlorofluorination procedure 2 (Anti: internal alkenes)** **69**

***Syn*-chlorofluorination procedure 3 (Syn: internal alkenes)** **70**

***Anti*-chlorofluorination procedure 4 (Anti: PIFA instead of electrochemically generated tol-IF$_2$)** **70**

***Syn*-chlorofluorination procedure 5 (Syn: PIFA instead of electrochemically generated TolIF$_2$)** .. **71**

Characterisation of products **72**

Terminal chlorofluorination products **72**
N-(5-chloro-4-fluoropentyl)-4-methylbenzenesulfonamide, 2j 72
N-(3-chloro-2-fluoropropyl)-N-(4-methylisoxazol-3-yl)-4-nitrobenzenesulfonamide, 2j 73
N-(3-chloro-2-fluoropropyl)-N-(4-fluoro-2-methylphenyl)-4-nitrobenzenesulfonamide, 3j 74
4-(3-chloro-2-fluoropropyl)-N,N-dimethylaniline 5j 76
1-(2-chloro-3-fluoropropyl)-4-fluorobenzene, 6j 77
1-(3-chloro-2-fluoropropyl)piperidine, 7j 78
11-chloro-10-fluoroundecan-1-ol, 8j 79
2-(11-chloro-10-fluoroundecyl)isoindoline-1,3-dione, 9j 80
1-(2-chloro-1-fluoroethyl)-3-nitrobenzene, 10j 81
(S)-((1S,2S,4S,5R)-5-((R)-1-chloro-2-fluoroethyl)quinuclidin-2-yl)(quinolin-4-yl)methyl acetate, 11k 82
1-(2-(3-chloro-2-fluoropropoxy)-2-(2,4-dichlorophenyl)ethyl)-1H-imidazole, 12j 83
Benzyl 2-(3-chloro-4-(3-chloro-2-fluoropropoxy)phenyl)acetate, 13j 84
(4S)-4-(1-chloro-2-fluoropropan-2-yl)-2-methylcyclohex-2-en-1-one, 14j 85
6-chloro-5-fluorohexyl 3,5-dinitrobenzoate, 15j 86

Internal alkene *anti*-chlorofluorination products **87**
N-benzyl-N-((3S,4S)-3-chloro-4-fluorohexyl)-4-fluoroaniline, 1b 87
N-benzyl-N-((3S,4S)-3-chloro-4-fluorohexyl)-4-(trifluoromethyl)aniline, 16b 89
N-benzyl-N-((3S,4S)-3-chloro-4-fluorohexyl)-4-iodoaniline, 17b 90
N-benzyl-N-((3S,4S)-3-chloro-4-fluorohexyl)aniline, 18b 91
N-benzyl-N-((3S,4S)-3-chloro-4-fluorohexyl)-4-methylaniline, 19b 92
N-((3S,4R)-3-chloro-4-fluorohexyl)-4-fluoroaniline, 20d 93
N-benzyl-N-((3S,4R)-3-chloro-4-fluorohexyl)-4-fluoroaniline, 21d 94
(3R,4R)-N-benzhydryl-N-benzyl-3-chloro-4-fluorohexan-1-amine 22b 95
4-(trifluoromethyl)phenyl (9R,10S)-9-chloro-10-fluorooctadecanoate, 23d 96
(R)-4-(2-chloro-1-fluoro-2-methylpropyl)-5-methylthiazole, 24b 97
(2S,3S)-N,N-dibenzyl-2-chloro-3-fluorohexan-1-amine, 25b 98

N,N-dibenzyl-3-chloro-4-fluorohexan-1-amine, 26b ... 99
(4S,5S)-N,N-dibenzyl-4-chloro-5-fluorohexan-1-amine, 27b ... 100
(3S,5S,6S,8S,9S,10R,13R,14S)-5-chloro-6-fluoro-10,13-dimethyl-17-((R)-6-methylheptan-2-yl)hexadecahydro-1H-cyclopenta[a]phenanthren-3-ol, 28b ... 101
Tert-butyl(((3S,4S)-3-chloro-4-fluorohexyl)oxy)diphenylsilane, 29b ... 102
1-chloro-2-fluoropropyl 4-fluorobenzoate, 30d ... 103
(2S,3R)-N,N-dibenzyl-2-chloro-3-fluorobutanamide, 31d ... 104
(3S,4S)-3-chloro-4-fluoro-1-tosylpyrrolidine, 32b ... 105
(3S,4S)-3-Chloro-4-fluorohexan-1-ol, 33b ... 106
tert-butyl 9-((3R,4R)-3-chloro-4-fluorohexyl)-3,9-diazaspiro[5.5]undecane-3-carboxylate, 34b ... 107
3,4,6-tri-O-acetyl-2-deoxy-2-chloro-α-D-mannopyranosyl fluoride, α-anti-35b, and 3,4,6-tri-O-acetyl-2-deoxy-2-chloro-α-D-glucopyranosyl fluoride, α-syn-35b, and 3,4,6-tri-O-acetyl-2-deoxy-2-chloro-β-D-glucopyranosyl fluoride, β-anti-35b ... 108
(3R,4R)-3-chloro-4-fluorohexyl 3,5-dinitrobenzoate, 36d ... 111
2-(1-((3R,4R)-3-chloro-4-fluorohexyl)piperidin-4-yl)-4,6-dimethoxypyrimidine, 37b ... 112
4-fluorobenzyl (3R,4R)-3-chloro-4-fluorohexanoate, 38b ... 113
4-fluorobenzyl (3R,4R)-3-chloro-4-fluorohexanoate, 38d ... 114
6-Chloro-7-fluoro-8-oxabicyclo[3.2.1]octan-3-one, 39b ... 115

Internal alkene *syn*-chlorofluorination products ... 117

N-benzyl-N-((3S,4R)-3-chloro-4-fluorohexyl)-4-(trifluoromethyl)aniline, 16d ... 117
N-benzyl-N-((3S,4R)-3-chloro-4-fluorohexyl)-4-iodoaniline, 17d ... 118
N-benzyl-N-((3S,4R)-3-chloro-4-fluorohexyl)-4-fluoroaniline, 1d ... 119
N-benzyl-N-((3S,4R)-3-chloro-4-fluorohexyl)aniline, 18d ... 120
N-benzyl-N-((3S,4R)-3-chloro-4-fluorohexyl)-4-methylaniline, 19d ... 121
2-((3S,4R)-3-Chloro-4-fluorohexyl)isoindoline, 40d ... 122
3-(1-((3S,4R)-3-chloro-4-fluorohexyl)piperidin-4-yl)-6-fluorobenzo[d]isoxazole, 41d ... 123
2-(1-((3R,4S)-3-chloro-4-fluorohexyl)piperidin-4-yl)-4,6-dimethoxypyrimidine, 37d ... 124
4-((3S,4R)-3-Chloro-4-fluorohexyl)morpholine, 42d ... 125
1-((3S,4R)-3-Chloro-4-fluorohexyl)-4-(4-nitrophenyl)piperazine, 43d ... 126
(1R,5S)-8-((3R,4R)-3-chloro-4-fluorohexyl)-8-azabicyclo[3.2.1]octan-3-one, 44d ... 127
N,N-dibenzyl-3-chloro-4-fluorohexan-1-amine, 26b ... 128
(3R,4R)-N,N-dibenzyl-3-chloro-4-fluorooctan-1-amine, 45b ... 129
(3R,4R)-N,N-dibenzyl-3-chloro-4-fluorononan-1-amine, 46b ... 130
(E)-N-((3S,4S)-3-chloro-4-fluorohexyl)-N-(4-fluorobenzyl)hex-3-en-1-amine, 47b ... 131
2-((3S,4S)-3-Chloro-4-fluorohexyl)isoindoline, 40b ... 132
4-fluorobenzyl (3R,4S)-3-chloro-4-fluorohexanoate, 38d ... 133

***X-ray crystallography* ... 134**

***Benchmarking experiments* ... 136**

Comparison against current state of the art methods ... 136

Variation of oxidant used in the formation of *p*-TolIF$_2$ ... 139

Anti-chlorofluorination using electrophilic fluorine source and nucleophilic chloride source (F$^+$/Cl$^-$) ... 141

***Studies into identity of active iodane under anti-chlorofluorination regime* ... 142**

Synthesis of *p*TolICl$_2$ ... 142

Use of pre-formed *p*-TolICl$_2$ in the reaction ... 143

Use of pre-formed *p*-TolIF$_2$ and *p*-TolICl$_2$ in the reaction ... 144

Use of pre-formed p-Tol-ICl2 in presence of Tol-I 145

Reaction monitoring of anti-chlorofluorination *146*

Reaction monitoring of *p*-Tol-IF_2 in presence of alkene and chloride 146

^{1}H NMR spectrum of 4-iodotoluene at −46 °C 148
^{1}H NMR spectrum of p-Tol-IF_2 at −46 °C 149
^{19}F NMR spectrum of p-Tol-IF_2 at −46 °C 149
^{1}H NMR spectrum of p-Tol-IF_2 + alkene cis-1a at −46 °C 151
^{19}F NMR spectrum of p-Tol-IF_2 + alkene cis-1a at −46 °C 151
^{1}H NMR spectrum of alkene cis-1a at −46 °C 152
^{19}F NMR spectrum of alkene cis-1a at −46 °C 152
^{1}H NMR spectrum of p-Tol-IF_2 + alkene cis-1a + 0.25 eq. chloride at −46 °C 153
^{19}F NMR spectrum of p-Tol-IF_2 + alkene cis-1a + 0.25 eq. chloride at −46 °C 153
^{1}H NMR spectrum of p-Tol-IF_2 + alkene cis-1a + 0.5 eq. chloride at −46 °C 154
^{19}F NMR spectrum of p-Tol-IF_2 + alkene cis-1a + 0.5 eq. chloride at −46 °C 154
^{1}H NMR spectrum of p-Tol-IF_2 + alkene cis-1a + 0.75 eq. chloride at −46 °C 155
^{19}F NMR spectrum of p-Tol-IF_2 + alkene cis-1a + 0.75 eq. chloride at −46 °C 155
^{1}H NMR spectrum of p-Tol-IF_2 + alkene cis-1a + 1.0 eq. chloride at −46 °C 156
^{19}F NMR spectrum of p-Tol-IF_2 + alkene cis-1a + 1.0 eq. chloride at −46 °C 156
Stacked ^{1}H NMR spectra of p-Tol-IF_2 + cis-1a + Et_4NCl at −46 °C 157
Stacked ^{19}F NMR spectra of p-Tol-IF_2 + cis-1a + Et_4NCl at −46 °C 158

Reaction monitoring of *p*-Tol-IF_2 in presence of chloride 159

^{1}H NMR spectrum of p-Tol-IF_2 at −46 °C 160
^{1}H NMR spectrum of p-Tol-IF_2 + 0.25 eq. Et_4NCl at −46 °C 161
^{1}H NMR spectrum of p-Tol-IF_2 + 0.5 eq. Et_4NCl at −46 °C 162
^{1}H NMR spectrum of p-Tol-IF_2 + 0.75 eq. Et_4NCl at −46 °C 163
^{1}H NMR spectrum of p-Tol-IF_2 + 1.0 eq. Et_4NCl at −46 °C 164

^{1}H NMR of *p*-Tol-IF_2 and *p*-Tol-ICl_2 in 1:1 ratio 165

^{1}H NMR spectrum of p-Tol-IF_2 + p-TolICl_2 (1:1) at −46 °C 165

^{1}H NMR of *p*-Tol-ICl_2 166

^{1}H NMR spectrum of p-Tol-ICl_2 at −46 °C 166

Stacked ^{1}H NMR spectra of *p*-Tol-IF_2, *p*-Tol-ICl_2, *p*-Tol-IF_2 + 1.0 eq. Et_4NCl and *p*-Tol-IF_2 + 1.0 eq. *p*-Tol-ICl_2 167

Stacked ^{1}H NMR spectra of *p*-Tol-IF_2 + Et_4NCl (0-1.0 eq.) 168

Mimicking HF equivalents syn-chlorofluorination regime with 5.6HF:amine *169*

Variation of HF:amine ratio in Olah conditions *170*

Studies into chloride nucleophilicity in 5.6HF:amine and 7HF:amine *172*

Mass Percentage of HF in Stock Reagents 172
Fluoride Concentration Calibration Curve and Measurements 172
General procedure for NMR kinetics 176
Substrate synthesis 178
COPASI modelling of reaction of n-butyl mesylate in 5.6HF:amine 179
COPASI modelling of reaction with n-butyl mesylate in 7HF:amine 180
COPASI modelling of reaction of 4-nitrobenzyl bromide in 5.6HF•amine 181
COPASI modelling of reaction with 4-nitrobenzyl bromide in 7HF•amine 182

Mechanism that explains formation of three diastereomers of 35b *183*

Proposed mechanism delivering three observed diastereomers ... 183

Inconsistent mechanism: direct chloronium formation ... 185

Inconsistent mechanism: *syn*-1,2-fluoro-λ^3-iodanation ... 186

Inconsistent mechanism: *syn*-1,2-chloro-λ^3-iodanation ... 187

Variation of HF:amine in difluorination at −46 °C ... 188

Computational studies ... 190

Computational methods ... 190

Activation barrier for first order reaction at −46 °C ... 191

Iodine(III)iranium cation vs iodine(III)-π complex ... 192

Chloronium-forming transition states via anchimeric assistance ... 193

QTAIM analysis of F---I-Tol halogen bonding ... 193

Ligand coupling transition states ... 195

Terminal alkene model ... 195

Internal alkene model ... 196

Direct chloronium-forming transition states by formal "Cl$^+$" transfer from chloride-bearing iodanes ... 197

***Syn* 1,2-halo-λ^3-iodanation VS chloride nucleophilic addition ... 198**

Charge and orbital features of iodanes: IF2, IFCl and ICl2 ... 199

IF2: Computed structure and LUMO coefficients ... 199

IFCl: Computed structure and LUMO coefficients ... 199

ICl2: Computed structure and LUMO coefficients ... 199

π-complexation of alkene and three possible iodanes ... 201

π-complexation of *trans*-alkene ... 202

Ligand exchange ... 203

Ligand metathesis mechanism between iodanes ... 204

Calculations of fluoride and chloride nucleophilicities ... 205

References ... 207

NMR spectra of optimisation substrates and products ... 209

(Z)-N-benzyl-4-fluoro-N-(hex-3-en-1-yl)aniline, 1a-cis ... 209

(E)-N-benzyl-4-fluoro-N-(hex-3-en-1-yl)aniline, 1a-trans ... 211

N-benzyl-N-((3S,4S)-3-chloro-4-fluorohexyl)-4-fluoroaniline, 1b ... 213

N-benzyl-N-((3S,4R)-3-chloro-4-fluorohexyl)-4-fluoroaniline, 1d ... 215

N-benzyl-N-((3S,4S)-3,4-dichlorohexyl)-4-fluoroaniline, 1f ... 217

N-benzyl-N-((3S,4R)-3,4-dichlorohexyl)-4-fluoroaniline, 1h ... 219

N-benzyl-N-((3R,4S)-3,4-difluorohexyl)-4-fluoroaniline, 1g ... 221

N-benzyl-N-((3S,4S)-3,4-difluorohexyl)-4-fluoroaniline, 1i ... 223

NMR spectra of substrates ... 225

N-allyl-N-(4-fluoro-2-methylphenyl)-4-nitrobenzenesulfonamide, 3a ... 225

N-allyl-N-(4-methylisoxazol-3-yl)-4-nitrobenzenesulfonamide, 4a ... 226

4-allyl-N,N-dimethylaniline, 5a ... 227

2-(undec-10-en-1-yl)isoindoline-1,3-dione, 9a 228
(S)-quinolin-4-yl((1S,2S,4S,5R)-5-vinylquinuclidin-2-yl)methyl acetate, 11a 229
Hex-5-en-1-yl-3,5-dinitrobenzoate, 15a 230
(Z)-N-benzyl-N-(hex-3-en-1-yl)-4-(trifluoromethyl)aniline, 16a 231
(Z)-N-benzyl-N-(hex-3-en-1-yl)-4-iodoaniline, 17a 233
(Z)-N-benzyl-N-(hex-3-en-1-yl)aniline, 18a 234
(Z)-N-benzyl-N-(hex-3-en-1-yl)-4-methylaniline, 19a 235
(Z)-4-fluoro-N-(hex-3-en-1-yl)aniline, 20a 236
(Z)-N-benzhydryl-N-benzylhex-3-en-1-amine, 22a 238
4-(Trifluoromethyl)phenyl (E)-octadec-9-enoate, 23a 239
5-methyl-4-(2-methylprop-1-en-1-yl)thiazole, 24a 240
(Z)-N,N-dibenzylhex-3-en-1-amine, 26a-cis 241
(Z)-N,N-dibenzylhex-4-en-1-amine, 27a 242
(Z)-tert-butyl(hex-3-en-1-yloxy)diphenylsilane, 29a 243
(E)-N,N-dibenzylbut-2-enamide, 31a 245
1-tosyl-2,5-dihydro-1H-pyrrole, 32a 246
tert-Butyl-(Z)-9-(hex-3-en-1-yl)-3,9-diazaspiro[5.5]undecane-3-carboxylate, 34a 247
(E)-hex-3-en-1-yl 3,5-dinitrobenzoate, 36a 248
(Z)-2-(1-(Hex-3-en-1-yl)piperidin-4-yl)-4,6-dimethoxypyrimidine, 37a 249
(Z)-2-(hex-3-en-1-yl)isoindoline, 40a-cis 250
(Z)-6-fluoro-3-(1-(hex-3-en-1-yl)piperidin-4-yl)benzo[d]isoxazole, 41a 251
(Z)-4-(hex-3-en-1-yl)morpholine, 42a 252
(Z)-1-(hex-3-en-1-yl)-4-(4-nitrophenyl)piperazine, 43a 253
(1R,5S)-8-((E)-hex-3-en-1-yl)-8-azabicyclo[3.2.1]octan-3-one, 44a 254
(E)-N,N-dibenzyloct-3-en-1-amine, 45a 255
(E)-N,N-dibenzylnon-3-en-1-amine, 46a 256
(E)-N-(4-fluorobenzyl)-N-((E)-hex-3-en-1-yl)hex-3-en-1-amine, 47a 257
(E)-2-(hex-3-en-1-yl)isoindoline, 40a-trans 259

***NMR spectra of products* 260**

NMR spectra of terminal chlorofluorination products........ 260
N-(5-chloro-4-fluoropentyl)-4-methylbenzenesulfonamide, 2j 260
N-(3-chloro-2-fluoropropyl)-N-(4-fluoro-2-methylphenyl)-4-nitrobenzenesulfonamide, 3j 262
N-(3-chloro-2-fluoropropyl)-N-(4-methylisoxazol-3-yl)-4-nitrobenzenesulfonamide, 4j 264
4-(3-chloro-2-fluoropropyl)-N,N-dimethylaniline, 5j 266
1-(2-chloro-3-fluoropropyl)-4-fluorobenzene, 6j 268
11-chloro-10-fluoroundecan-1-ol, 8j 270
2-(11-chloro-10-fluoroundecyl)isoindoline-1,3-dione, 9j 271
1-(2-chloro-1-fluoroethyl)-3-nitrobenzene, 10j 273
(S)-((1S,2S,4S,5R)-5-((R)-1-chloro-2-fluoroethyl)quinuclidin-2-yl)(quinolin-4-yl)methyl acetate, 11k 275
1-(2-(3-chloro-2-fluoropropoxy)-2-(2,4-dichlorophenyl)ethyl)-1H-imidazole, 12j 277
Benzyl 2-(3-chloro-4-(3-chloro-2-fluoropropoxy)phenyl)acetate, 13j 279
(4S)-4-(1-chloro-2-fluoropropan-2-yl)-2-methylcyclohex-2-en-1-one, 14j 281
6-chloro-5-fluorohexyl 3,5-dinitrobenzoate, 15j 283

NMR spectra of internal alkene *anti*-chlorofluorination products 285
N-benzyl-N-((3S,4S)-3-chloro-4-fluorohexyl)-4-fluoroaniline, 1b 285
N-benzyl-N-((3S,4S)-3-chloro-4-fluorohexyl)-4-(trifluoromethyl)aniline, 16b 287
N-benzyl-N-((3S,4S)-3-chloro-4-fluorohexyl)-4-iodoaniline, 17b 289
N-benzyl-N-((3S,4S)-3-chloro-4-fluorohexyl)aniline, 18b 291
N-benzyl-N-((3S,4S)-3-chloro-4-fluorohexyl)-4-methylaniline, 19b 293
N-((3S,4R)-3-chloro-4-fluorohexyl)-4-fluoroaniline, 20d 295
N-benzyl-N-((3S,4R)-3-chloro-4-fluorohexyl)-4-fluoroaniline, 21d 297

(3R,4R)-N-benzhydryl-N-benzyl-3-chloro-4-fluorohexan-1-amine, 22b *299*
4-(trifluoromethyl)phenyl (9R,10S)-9-chloro-10-fluorooctadecanoate, 23d *301*
(R)-4-(2-chloro-1-fluoro-2-methylpropyl)-5-methylthiazole, 24b *303*
(2S,3S)-N,N-dibenzyl-2-chloro-3-fluorohexan-1-amine, 25b *305*
N,N-dibenzyl-3-chloro-4-fluorohexan-1-amine, 26b *307*
(4S,5S)-N,N-dibenzyl-4-chloro-5-fluorohexan-1-amine, 27b *309*
(3S,5S,6S,8S,9S,10R,13R,14S)-5-chloro-6-fluoro-10,13-dimethyl-17-((R)-6-methylheptan-2-yl)hexadecahydro-1H-cyclopenta[a]phenanthren-3-ol, 28b *311*
Tert-butyl(((3S,4S)-3-chloro-4-fluorohexyl)oxy)diphenylsilane, 29b *313*
1-chloro-2-fluoropropyl 4-fluorobenzoate, 30d *315*
(2S,3R)-N,N-dibenzyl-2-chloro-3-fluorobutanamide, 31d *317*
(3S,4S)-3-chloro-4-fluoro-1-tosylpyrrolidine, 32b *319*
(3S,4S)-3-chloro-4-fluorohexan-1-ol, 33b *321*
tert-butyl 9-((3R,4R)-3-chloro-4-fluorohexyl)-3,9-diazaspiro[5.5]undecane-3-carboxylate, 34b *323*
3,4,6-tri-O-acetyl-2-deoxy-2-chloro-α-D-mannopyranosyl fluoride, α-anti-35b *325*
3,4,6-tri-O-acetyl-2-deoxy-2-chloro-α-D-glucopyranosyl fluoride, α-syn-35b *327*
3,4,6-tri-O-acetyl-2-deoxy-2-chloro-β-D-glucopyranosyl fluoride, β-anti-35b *329*
(3R,4R)-3-chloro-4-fluorohexyl 3,5-dinitrobenzoate, 36d *331*
2-(1-((3R,4R)-3-chloro-4-fluorohexyl)piperidin-4-yl)-4,6-dimethoxypyrimidine, 37b *333*
6-chloro-7-fluoro-8-oxabicyclo[3.2.1]octan-3-one, 39b *335*

NMR spectra of internal *syn*-chlorofluorination products 337
N-benzyl-N-((3S,4R)-3-chloro-4-fluorohexyl)-4-(trifluoromethyl)aniline, 16d *337*
N-benzyl-N-((3S,4R)-3-chloro-4-fluorohexyl)-4-iodoaniline, 17d *339*
N-benzyl-N-((3S,4R)-3-chloro-4-fluorohexyl)-4-fluoroaniline, 1d *341*
N-benzyl-N-((3S,4R)-3-chloro-4-fluorohexyl)aniline, 18d *343*
N-benzyl-N-((3S,4R)-3-chloro-4-fluorohexyl)-4-methylaniline, 19d *345*
2-((3S,4R)-3-Chloro-4-fluorohexyl)isoindoline, 39d *347*
3-(1-((3S,4R)-3-chloro-4-fluorohexyl)piperidin-4-yl)-6-fluorobenzo[d]isoxazole, 41d *349*
2-(1-((3R,4S)-3-chloro-4-fluorohexyl)piperidin-4-yl)-4,6-dimethoxypyrimidine, 37d *351*
4-((3S,4R)-3-Chloro-4-fluorohexyl)morpholine, 42d *353*
1-((3S,4R)-3-Chloro-4-fluorohexyl)-4-(4-nitrophenyl)piperazine, 43d *355*
(1R,5S)-8-((3R,4R)-3-chloro-4-fluorohexyl)-8-azabicyclo[3.2.1]octan-3-one, 44d *357*
(3R,4R)-N,N-dibenzyl-3-chloro-4-fluorooctan-1-amine, 45b *359*
(3R,4R)-N,N-dibenzyl-3-chloro-4-fluorononan-1-amine, 46b *361*
(E)-N-((3S,4S)-3-chloro-4-fluorohexyl)-N-(4-fluorobenzyl)hex-3-en-1-amine, 47b *363*
2-((3S,4S)-3-Chloro-4-fluorohexyl)isoindoline, 40b *365*

General experimental

Techniques

Manipulations involving air and moisture sensitive materials were conducted employing standard Schlenk-line and glovebox techniques, using vacuum lines attached to a double manifold with greaseless J. Youngs valves equipped with an oil pump (0.1 mmHg) under an atmosphere of dry nitrogen. All glassware was dried overnight before use, in a 180 °C oven and then allowed to cool under vacuum at 0.1 mmHg. The removal of solvents *in vacuo* was achieved using a Büchi rotary evaporator (bath temperatures up to 40 °C) at a pressure of 15 mmHg (diaphragm pump), or at 0.1 mmHg (oil pump) on a vacuum line at room temperature. The addition of liquids, except for HF solutions, were added using a Gilson PIPETMAN p20.

Solvents

THF (tetrahydrofuran), CH_2Cl_2, CH_3CN and Et_2O was dried using an Anhydrous Engineering alumina column drying system situated in the University of Bristol's chemistry department. All solvents were collected using Strauss flasks using a gas-tight J. Youngs valve. Anhydrous 1,1,1,3,3,3-hexafluoroisopropanol (HFIP) was collected by distillation after stirring in $MgSO_4$ overnight. Deuterated solvents for NMR analysis were purchased from *Sigma Aldrich*.

Chromatography

TLC analysis was performed on Merck Silica gel $60F_{254}$ glass backed plates. Visualisation was achieved by UV fluorescence (254 nm). Flash column chromatography was conducted using Fluorochem 60 silica: 230-400 mesh (40-63 µm). HPLC purification was performed on a BUCHI C-850 FlashPrep fitted with a PrepPure C18 100 Å 10 µM, 250 x 20mm column using MeCN + 0.1% Formic Acid or H_2O, 0.1% Formic Acid or a combination thereof as mobile phases. Samples were liquid loaded in methanol (volume 1.0 – 5.0 mL) and a DIAD was employed with monitoring at 220 nm alongside an ELSD.

Method: Flow rate 25 mL/min. A = MeCN + 0.1% Formic Acid, B = H_2O + 0.1% Formic Acid. Gradient elution: t = 0 min (20:80, A:B), t = 15 min (60:40, A:B), t = 20 min (60:40, A:B).Total run time 30 min.

Reagents

N-(3-Dimethylaminopropyl)-*N'*-ethylcarbodiimide hydrochloride, sodium hydride in mineral oil, cyclopropylamine, triphenylphosphine, (+)-cinchonine, 6-fluoro-3-(4-piperidinyl)benzisoxazole, 4-fluorobenzylamine, cholesterol, trichloroisocyanuric acid, hexafluoroantimonic acid, pyridinium poly(hydrogenfluoride), *N*-chlorosuccinimide, *N,N,N*-disopropylethylamine, 4-iodoaniline, 4-fluoroaniline, aniline and 4-methylaniline, were all purchased from Sigma Aldrich and used without further purification. 4-Nitrobenzenesulfonyl chloride, diisopropyl azodicarboxylate, bis(trifluoroacetoxy)iodobenzene, *N*-(*tert*-butoxycarbonyl)-*p*-toluenesulfonamate, 3-amino-5-methylisoxazole, *N*-bromo succinimide, 4-methylthiazolecarboxaldehyde, (E)-crotonic acid, (E)-crotonyl chloride, 1-(4-nitrophenyl)piperazine and nortropinone hydrochloride were all purchased from Fluorochem. Morpholine, triethylamine, pyridine, potassium carbonate, 4-(dimethylamino)pyridine, HCl in ether (2M), 4-penten-1-ol, *tert*-butyldiphenylsilyl chloride, (Z)-2-hexen-1-ol, (Z)-4-hexen-1-ol, 3-pyrroline, L-carvone and tri-*O*-acetyl-D-glucal were all purchased from Alfa-Aesar. 4-Toluenesulfonyl chloride, isopropyltriphenylphosphonium iodide, isoindoline and 4-nitrobenzyl bromide were all purchased from Acros. Benzyl alcohol was purchased from Honeywell. 4-Trifluoromethylaniline and acetyl chloride were purchased from Apollo Scientific. Enilconazole, 8-oxabicyclo[3.2.1]oct-6-en-3-one and 2-(3-chloro-4-hydroxyphenyl)acetic acid were supplied by GSK.

Analysis

NMR spectra were recorded on Bruker Nano 400 or Bruker Advance III HD 500 cryo spectrometers. Chemical shifts (δ) are quoted in parts per million (ppm), referenced to the residual solvent peak (^{1}H and ^{13}C NMR) and coupling constants (*J*) are given in Hz. Multiplicities are abbreviated as: s (singlet), d (doublet), t (triplet), q (quartet), m (multiplet) or combinations thereof. NMR shifts for novel compounds have been assigned with the use of the appropriate 2D NMR experiments, such as COSY, HSQC and HMBC. High resolution mass spectra (HRMS) were recorded on a VG Analytical

Autospec spectrometer by Electron Ionisation (EI) or Chemical Ionisation (CI) or on a Bruker micrOTOF instrument by Electrospray Ionisation (ESI). Infrared spectra were recorded using a Perkin Elmer Spectrum Two FT-IR spectrometer.

Electrochemical techniques

All cyclic voltammetric (CV) and chronopotentiometric measurements were performed at room temperature using an Autolab M101 or a Tenma PSU (purchased from Farnell) and the ElectraSyn 2.0 (purchased from IKA).

Warning: Use of HF reagents

The hazards of hydrogen fluoride solutions are well categorised and can be viewed in this SDS (https://www.sigmaaldrich.com/catalog/product/aldrich/184225). Therefore, personal protection is of utmost importance. It is advised to wear two pairs of nitrile gloves when handling, and if the gloves come into contact with HF, they are removed immediately, the area affected washed thoroughly with Hexafluorine solutionTM (http://www.medicalcare.se/diphoterine-and-hexafluorine), calcium glucanoate gel is applied to the area and medical attention is sought. It is advised that Hexafluorine solution^{TM} and calcium glucanoate gel is kept nearby for personal use.

Synthesis of standard: all products observed during optimization

(Z)-N-benzyl-4-fluoro-N-(hex-3-en-1-yl)aniline, 1a-cis

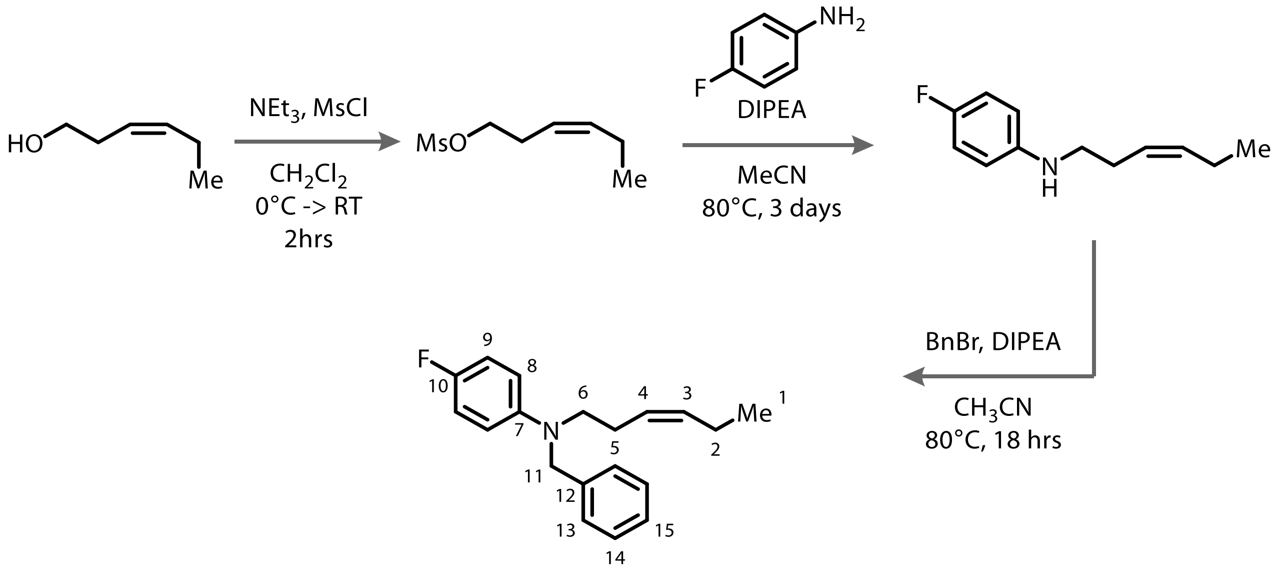

To a round-bottomed flask charged with a stirrer bar was added (*Z*)-3-hexen-1-ol (1 eq., 10 mmol, 1.18 mL), CH_2Cl_2 (50 mL) and methanesulfonyl chloride (1.5 eq., 15 mmol, 1.15 mL). After the solution was cooled to 0 °C, triethylamine (2.0 eq., 20 mmol, 2.75 mL) was added dropwise as a colourless precipitate and effervescence was observed. After addition, the solution was allowed to reach room temperature and left to stir for 2 hours. Saturated aqueous K_2CO_3 solution (100 mL) was added, and the organic phase was extracted using CH_2Cl_2 (3 x 50 mL). This mixture was then washed with brine, dried with $MgSO_4$, concentrated in *vacuo,* and used for the next step without further purification.

To a round-bottomed flask charged with a stirrer bar, was added the mesylated alcohol (1 eq., 10 mmol), *p*-fluoroaniline (2 eq., 20 mmol, 2 mL), DIPEA (5 eq., 50 mmol, 8.9 mL) and acetonitrile (20 mL). This reaction mixture was heated to reflux and allowed to stir for 3 days. The acetonitrile was then removed in *vacuo* and EtOAc (50 mL) and water (50 mL) was added to the remaining reaction mixture. The organic phase was extracted with EtOAc (2 x 50 mL), dried with $MgSO_4$, concentrated in *vacuo* and the mono alkylated aniline was purified using silica-gel column chromatography.

To a round-bottomed flask charged with a stirrer bar was added the mono alkylated aniline (5 mmol), CH_3CN (20 mL), benzyl bromide (1.2 eq., 6 mmol, 1.43 mL) and *N*-diisopropylethylamine (5 eq., 25 mmol, 4.6 mL). This reaction mixture was heated to

reflux and allowed to stir overnight. The solvent was then removed in *vacuo* and EtOAc (50 mL) was added. The organic phase was extracted with EtOAc (2 x 50 mL), dried with MgSO4, and concentrated in *vacuo*. The benzylated amine was purified by silica-gel column chromatography (2% EtOAc:Hexane) to afford **1a-*cis*** as a colourless oil (1.20 g, 85%).

R_f = 0.15 (2% EtOAc:Hexane)

^{1}H NMR (400 MHz, $CDCl_3$): δ = 7.37 – 7.19 (5H, m, H^{13-15}), 6.89 (2H, t, *J* = 8.7 Hz, H^8), 6.70 – 6.56 (2H, m, H^9), 5.53 – 5.42 (1H, m, H^4), 5.39 – 5.28 (1H, m, H^3), 4.50 (2H, s, H^{11}), 3.42 – 3.29 (2H, m, H^6), 2.37 – 2.32 (2H, m, H^5), 2.02 (2H, pd, *J* = 7.5, 1.5 Hz, H^2), 0.94 (3H, t, *J* = 7.5 Hz, H^1).

^{19}F NMR (376 MHz, $CDCl_3$): δ = -129.34 – -129.59 (1F, m, F^{10}).

^{13}C {^{1}H} NMR (100 MHz, $CDCl_3$): δ = 146.5 (d, *J* = 250.7 Hz, C^{10}), 145.2 (C^7), 139.0 (C^{12}), 134.0 (C^4), 128.7 (C^{13}), 127.0 (C^{15}), 126.8 (C^{14}), 125.4 (C^3), 115.7 (d, *J* = 21.8 Hz, C^9),113.6 (d, *J* = 7.3 Hz, C^8), 55.3 (C^6), 51.8 (C^{11}), 25.2 (C^5), 20.8 (C^2), 14.5 (C^1).

HRMS (EI) calc: $[M]^+$ ($C_{19}H_{22}FN$) 283.1731; measured: 283.1728 = 1.06 ppm difference.

IR (neat) v_{max}/ cm^{-1}: 1608, 1510, 1415, 1356, 1229, 1205, 1162, 1072.

(E)-N-benzyl-4-fluoro-N-(hex-3-en-1-yl)aniline, 1a-trans

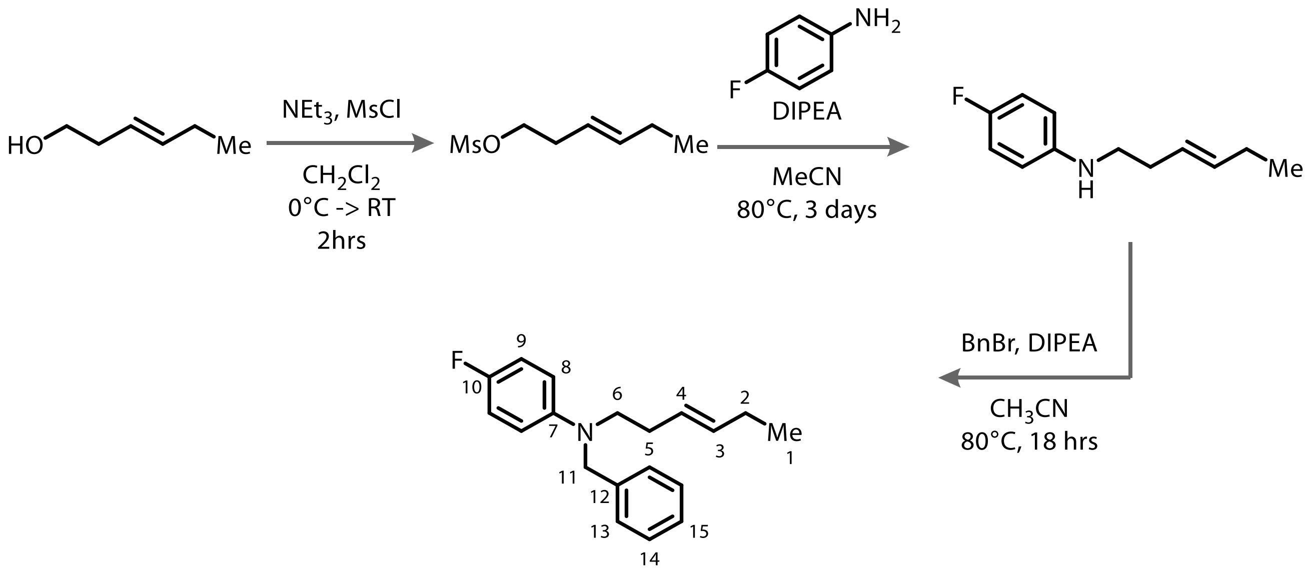

To a round-bottomed flask charged with a stirrer bar was added (E)-3-hexen-1-ol (1 eq., 10 mmol, 1.18 mL), CH_2Cl_2 (50 mL) and methanesulfonyl chloride (1.5 eq., 15 mmol, 1.15 mL). After the solution was cooled to 0ºC, triethylamine (2 eq., 20 mmol, 2.75 mL) was added dropwise as a colourless precipitate and effervescence was observed. After addition, the solution was allowed to reach room temperature and left to stir for 2 hours. Saturated aqueous K_2CO_3 solution (100 mL) was added, and the organic phase was extracted using CH_2Cl_2 (3 x 50 mL). This mixture was then washed with brine, dried with $MgSO_4$, concentrated in *vacuo,* and used for the next step without further purification.

To a round-bottomed flask charged with a stirrer bar, was added the mesylated alcohol (1 eq., 10 mmol), *p*-fluoroaniline (2 eq., 20 mmol, 2 mL), DIPEA (5 eq., 50 mmol, 8.9 mL) and acetonitrile (20 mL). This reaction mixture was heated to reflux and allowed to stir for 3 days. The acetonitrile was then removed in *vacuo* and EtOAc (50 mL) and water (50 mL) was added to the remaining reaction mixture. The organic phase was extracted with EtOAc (2 x 50 mL), dried with $MgSO_4$, concentrated in *vacuo* and the mono alkylated aniline was purified using silica-gel column chromatography.

To a round-bottomed flask charged with a stirrer bar was added the mono alkylated aniline (5 mmol), CH_3CN (20 mL), benzyl bromide (1.2 eq., 6 mmol, 1.43 mL) and *N*-diisopropylethylamine (5 eq., 25 mmol, 4.6 mL). This reaction mixture was heated to reflux and allowed to stir overnight. The solvent was then removed in *vacuo* and EtOAc

(50 mL) was added. The organic phase was extracted with EtOAc (2 x 50 mL), dried with MgSO4, and concentrated in *vacuo*. The benzylated amine was purified by silica-gel column chromatography (2% EtOAc:Hexane) to afford **1a-*trans*** as a colourless oil (0.95 g, 67%).

R_f = 0.15 (2% EtOAc:Hexane)

^{1}H NMR (400 MHz, $CDCl_3$): δ = 7.34 – 7.21 (5H, m, $H^{13\text{-}15}$), 6.95 – 6.85 (2H, m, H^8), 6.65 – 6.58 (2H, m, H^9), 5.61 – 5.52 (1H, m, H^4), 5.46 – 5.36 (1H, m, H^3), 4.51 (2H, s, H^{11}), 3.46 – 3.36 (2H, m, H^6), 2.41 – 2.27 (2H, m, H^5), 2.10 – 1.96 (2H, m, H^2), 0.99 (3H, t, *J* = 7.5 Hz, H^1).

^{19}F NMR (376 MHz, $CDCl_3$): δ = -129.46 – -129.81 (1F, m, F^{10}).

^{13}C {^{1}H} NMR (100 MHz, $CDCl_3$): δ = 155.2 (d, *J* = 249.2 Hz, C^{10}), 145.3 (C^7), 139.1 (C^{12}), 134.5 (C^4), 128.7 (C^{13}), 126.9 (C^{15}), 126.7 (C^{14}), 125.9 (C^3), 115.6 (d, *J* = 21.8 Hz, C^9),113.5 (d, *J* = 7.3 Hz, C^8), 55.3 (C^6), 52.2 (C^{11}), 30.5 (C^5), 25.8 (C^2), 13.9 (C^1).

HRMS (ESI) calc: $[M+H]^+$ ($C_{19}H_{22}FN$) 284.1809; measured: 284.1803 = 2.11 ppm difference.

IR (neat) ν_{max}/ cm^{-1}: 1616, 1511, 1407, 1355, 1232, 1209, 1157, 1051.

N-benzyl-N-((3S,4S)-3-chloro-4-fluorohexyl)-4-fluoroaniline, 1b

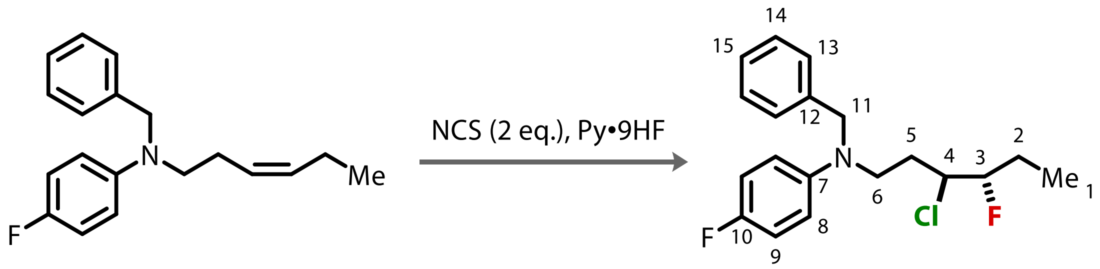

To a 100 mL HDPE vial was added, py•9HF (1 mL) and NCS (2 eq., 0.4 mmol, 27 mg). To this solution was added alkene (0.04 M, 1 eq., 0.2 mmol, 57 mg) in tetrahydrofuran over 10 minutes. After stirring overnight, the reaction mixture was quenched with 200 mL of cold (0 °C) saturated aqueous $NaHCO_3$ solution. This stirred for 1 hour until the aqueous layer measured pH 7. The mixture was extracted into CH_2Cl_2, dried with Na_2SO_4, filtered, and concentrated *in vacuo* and isolated using silica-gel column chromatography (2% EtOAc:Hexane) to afford **1b** as a colourless oil (23 mg, 33%).

R_f = 0.20 (4% EtOAc:Hexane)

^{1}H NMR (400 MHz, $CDCl_3$): δ = 7.35 – 7.30 (2H, m, H^{14}), 7.28 – 7.24 (1H, m, H^{15}), 7.24 – 7.19 (2H, m, H^{13}), 6.95 – 6.87 (2H, m, H^{8}), 6.71 – 6.64 (2H, m, H^{9}), 4.56 – 4.49 (2H, m, H^{11}), 4.47 – 4.31 (1H, m, H^{3}), 3.97 (1H, ddt, *J* = 21.8, 10.4, 3.2 Hz, H^{4}), 3.69 (1H, ddd, *J* = 14.9, 8.5, 4.4 Hz, H^{6}), 3.52 (1H, dt, *J* = 15.1, 7.8 Hz, H^{6}), 2.18 (1H, dddd, *J* = 14.4, 8.5, 7.3, 3.3 Hz, H^{2}), 2.06 (1H, dddd, *J* = 14.6, 10.5, 8.2, 4.3 Hz, H^{2}), 1.88 – 1.63 (2H, m, H^{5}), 0.98 (3H, t, *J* = 7.5 Hz).

^{19}F NMR (376 MHz, $CDCl_3$): δ = -128.39 – -128.47 (1F, m, F^{10}), -188.42 (1F, dddd, *J* = 46.4, 31.5, 21.9, 14.5 Hz, F^{3}).

^{13}C {^{1}H} NMR (100 MHz, $CDCl_3$): δ = 155.8 (d, *J* = 235.5 Hz, C^{10}), 145.1 (d, *J* = 1.6 Hz, C^{7}), 138.7 (C^{12}), 128.8 (C^{13}), 127.2 (C^{15}), 126.9 (C^{14}), 115.9 (d, *J* = 22.1 Hz, C^{9}), 114.3 (d, *J* = 7.2 Hz, C^{8}), 96.0 (d, *J* = 178.7 Hz, C^{3}), 60.7 (d, *J* = 22.5 Hz, C^{4}), 55.9 (C^{11}), 49.2 (C^{6}), 31.8 (d, *J* = 2.0 Hz, C^{5}), 24.8 (d, *J* = 21.7 Hz, C^{2}), 9.6 (d, *J* = 5.7 Hz, C^{1}).

HRMS (EI) calc: $[M]^+$ ($C_{19}H_{22}ClFN$) 337.1403; measured: 337.1402 = 0.30 ppm difference.

IR (neat) ν_{max}/ cm^{-1}: 1681, 1593, 1487, 1412, 1381, 1319, 1285, 1261, 1238, 1156, 1102, 1007.

N-benzyl-N-((3S,4R)-3-chloro-4-fluorohexyl)-4-fluoroaniline, 1d

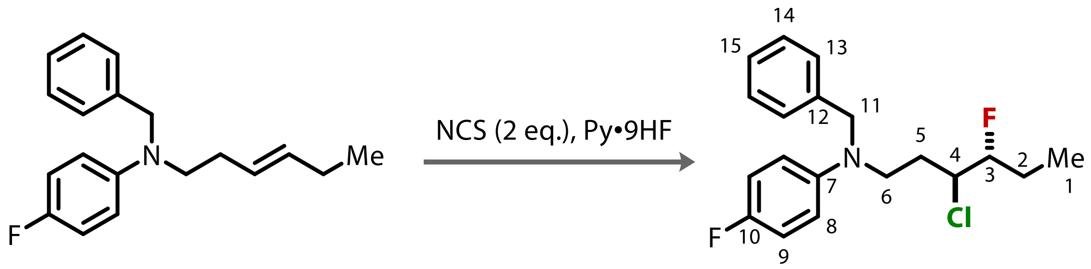

To a 100 mL HDPE vial was added, py•9HF (1 mL) and NCS (2 eq., 0.4 mmol, 27 mg). To this solution was added alkene (0.04 м, 1 eq., 0.2 mmol, 57 mg) in tetrahydrofuran over 10 minutes. After stirring overnight, the reaction mixture was quenched with 200 mL of cold (0 ºC) saturated aqueous $NaHCO_3$ solution. This stirred for 1 hour until the aqueous layer measured pH 7. The mixture was extracted into CH_2Cl_2, dried with Na_2SO_4, concentrated *in vacuo* and isolated using silica-gel column chromatography (2% EtOAc:Hexane) to produce **1c** as a colourless oil (23 mg, 33%).

R_f = 0.20 (4% EtOAc:Hexane)

^{1}H NMR (400 MHz, $CDCl_3$): δ = 7.35 – 7.27 (2H, m, H^{14}), 7.25 – 7.17 (1H, m, $H^{13,15}$), 6.90 (2H, dd, J = 9.2, 8.3 Hz, H^8), 6.71 – 6.62 (2H, m, H^9), 4.54 – 4.30 (3H, m, $H^{3,11}$), 3.95 (1H, tdd, J = 10.4, 6.4, 2.6 Hz, H^4), 3.70 (1H, ddd, J = 13.9, 9.1, 4.2 Hz, H^6), 3.51 (1H, ddd, J = 15.2, 8.9, 6.9 Hz, H^6), 2.33 – 2.23 (1H, m, H^5), 1.99 – 1.67 (3H, m, $H^{2,5}$), 1.00 (3H, t, J = 7.4 Hz, H^1).

^{19}F NMR (376 MHz, $CDCl_3$): δ = -128.66 (1F, tt, J = 8.5, 4.4 Hz, F^{10}), -183.77 (1F, dddd, J = 47.8, 31.9, 20.1, 10.6 Hz, F^3).

^{13}C {^{1}H} NMR (100 MHz, $CDCl_3$): δ = 155.6 (d, J = 228.0 Hz, C^{10}), 145.0 (C^7), 138.6 (C^{12}), 128.8 (C^{13}), 127.1 (C^{15}), 126.8 (C^{14}), 115.8 (d, J = 22.3 Hz, C^9), 114.1 (d, J = 6.6 Hz, C^8), 96.3 (d, J = 176.6 Hz, C^3), 60.3 (d, J = 25.6 Hz, C^4), 55.7 (C^{11}), 48.8 (C^6), 30.8 (d, J = 3.4 Hz, C^5), 25.0 (d, J = 22.3 Hz, C^2), 9.2 (d, J = 3.5 Hz, C^1).

HRMS (EI) calc: $[M]^+$ ($C_{19}H_{22}F_2{}^{35}ClN$) 337.4103; measured: 337.1401 = 0.59 ppm difference.

IR (neat) ν_{max}/ cm^{-1}: 1684, 1593, 1487, 1412, 1381, 1317, 1287, 1262, 1236, 1157, 1102, 1007.

N-benzyl-N-((3S,4S)-3,4-dichlorohexyl)-4-fluoroaniline, 1f

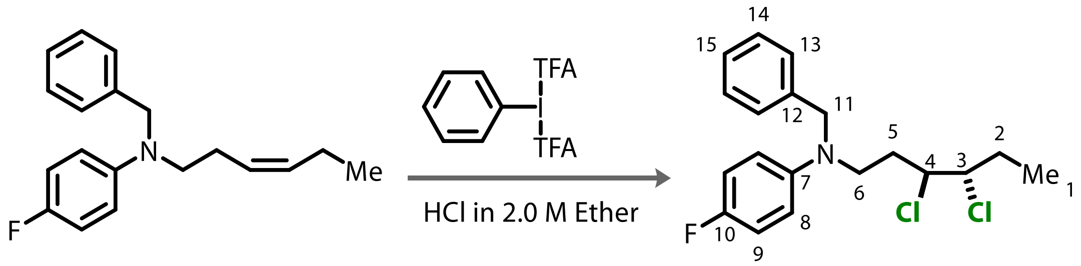

To a 5 mL round-bottomed flask was added bis(trifluoroacetoxy)iodobenzene (1.1 eq., 0.22 mmol, 90 mg), CH_2Cl_2 (1mL) and HCl in ether (2.0M, 10 eq., 1.0 mmol, 0.5 mL). After stirring for 5 minutes, (1 eq., 0.2 mmol, 58 mg) was added. After stirring overnight, the reaction mixture was quenched with 20 mL of cold (0 °C) saturated aqueous $NaHCO_3$ solution. This stirred for 1 hour until the aqueous layer measured pH 7. The mixture was extracted into CH_2Cl_2, dried with Na_2SO_4, concentrated *in vacuo* and isolated using silica-gel column chromatography (2% EtOAc:Hexane) to afford **1f** as a colourless oil (57 mg, 80%).

R_f = 0.33 (4% EtOAc:Hexane)

^{1}H NMR (500 MHz, $CDCl_3$): δ = 7.35 – 7.18 (5H, m, *$H^{13,14,15}$*), 6.97 – 6.86 (2H, m, *H^8*), 6.76 – 6.65 (2H, m, *H^9*), 4.50 (2H, d, *J* = 2.8 Hz, *H^{11}*), 4.11 (1H, dt, *J* = 10.4, 2.8 Hz, H^4), 3.89 (1H, ddd, *J* = 9.6, 4.0, 2.7 Hz, *H^3*), 3.65 (1H, ddd, *J* = 14.7, 8.5, 4.2 Hz, *H^6*), 3.49 (1H, dt, *J* = 15.0, 7.8 Hz, *H^6*), 2.27 (1H, dddd, *J* = 14.5, 8.5, 7.4, 2.8 Hz, *H^5*), 2.12 – 2.01 (1H, m, *H^5*), 2.00 – 1.90 (1H, m, *H^2*), 1.77 (1H, ddq, *J* = 14.5, 9.6, 7.3 Hz, *H^2*), 1.04 (t, *J* = 7.3 Hz, 2H).

^{19}F NMR (376 MHz, $CDCl_3$): δ = -128.29 (1F, m, *F^{10}*).

^{13}C {^{1}H} NMR (125 MHz, $CDCl_3$): δ = 155.9 (d, *J* = 241.3 Hz, *C^{10}*), 145.1 (*C^7*), 138.6 (*C^{12}*), 128.8 (*C^{13}*), 127.2 (*C^{15}*), 127.0 (*C^{14}*), 115.8 (d, *J* = 21.5 Hz, *C^9*), 114.5 (*C^8*), 67.5 (*C^4*), 62.9 (*C^3*), 55.9 (*C^{11}*), 49.3 (*C^6*), 32.3 (*C^5*), 27.8 (*C^2*), 11.5 (*C^1*).

HRMS (EI) calc: $[M]^+$ ($C_{19}H_{22}N^{35}Cl_2F$) 353.1108; measured: 353.1107 = 0.28 ppm difference.

IR (neat) ν_{max}/ cm^{-1}: 1638, 1608, 1510, 1453, 1356, 1229, 1163.

N-benzyl-N-((3S,4R)-3,4-dichlorohexyl)-4-fluoroaniline, 1h

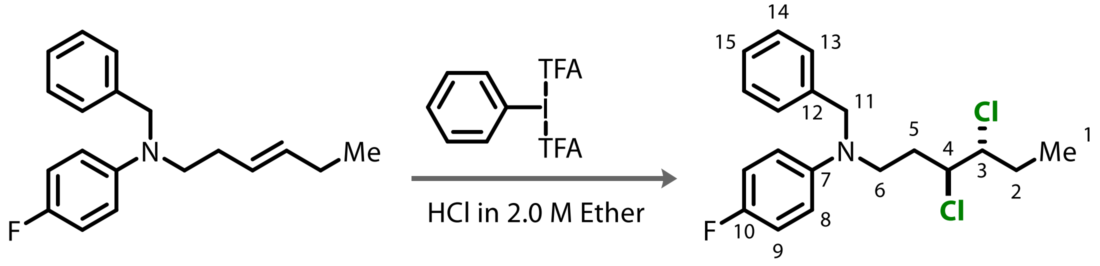

To a 5 mL round-bottomed flask was added bis(trifluoroacetoxy)iodobenzene (1.1 eq., 0.22 mmol, 90 mg), CH_2Cl_2 (1mL) and HCl in ether (2.0M, 10 eq., 1.0 mmol, 0.5 mL). After stirring for 5 minutes, (1 eq., 0.2 mmol, 58 mg) was added. After stirring overnight, the reaction mixture was quenched with 20 mL of cold (0 ºC) saturated aqueous $NaHCO_3$ solution. This stirred for 1 hour until the aqueous layer measured pH 7. The mixture was extracted into CH_2Cl_2, dried with Na_2SO_4, concentrated *in vacuo* and isolated using silica-gel column chromatography (2% EtOAc:Hexane) to afford **1h** as a colourless oil (57 mg, 80%).

R_f = 0.33 (4% EtOAc:Hexane)

^{1}H NMR (500 MHz, $CDCl_3$): δ = 7.36 – 7.21 (5H, m, $H^{13,14,15}$), 6.91 (2H, dd, J = 9.2 Hz, H^8), 6.68 (2H, dd, J = 9.1, 4.4 Hz, H^9), 4.51 (2H, s, H^{11}), 4.03 (1H, ddd, J = 9.6, 7.0, 2.3 Hz, H^4), 3.93 (1H, ddd, J = 8.9, 7.1, 3.1 Hz, H^3), 3.70 (1H, ddd, J = 14.7, 9.1, 4.3 Hz, H^6), 3.52 (1H, ddd, J = 15.0, 8.8, 6.8 Hz, H^6), 2.41 (2H, dddd, J = 14.4, 9.1, 6.8, 2.2 Hz, H^5), 2.15 – 1.95 (2H, m, $H^{5,2}$), 1.81 (1H, ddt, J = 14.5, 8.8, 7.3 Hz, H^2), 1.05 (3H, t, J = 7.3 Hz, H^1).

^{19}F NMR (376 MHz, $CDCl_3$): δ = -128.55 (1F, m, F^{10}).

^{13}C {^{1}H} NMR (125 MHz, $CDCl_3$): δ = 155.6 (d, J = 240. 6Hz, C^{10}), 144.9 (C^7), 138.6 (C^{12}), 128.8 (C^{13}), 127.2 (C^{15}), 126.9 (^{14n}), 115.8 (d, J = 21.5 Hz, C^9), 114.8 (C^8), 67.4 (C^4), 63.2 (C^3), 55.7 (C^{11}), 48.8 (C^6), 32.3 (C^5), 28.3 (C^2), 10.6 (C^1).

HRMS (EI) calc: $[M]^+$ ($C_{19}H_{22}F^{35}Cl_2N$) 353.1108; measured: 353.1106 = 0.57 ppm difference.

IR (neat) ν_{max}/ cm^{-1}: 1572, 1511, 1453, 1355, 1230, 1162.

N-benzyl-N-((3S,4S)-3,4-difluorohexyl)-4-fluoroaniline, 1g

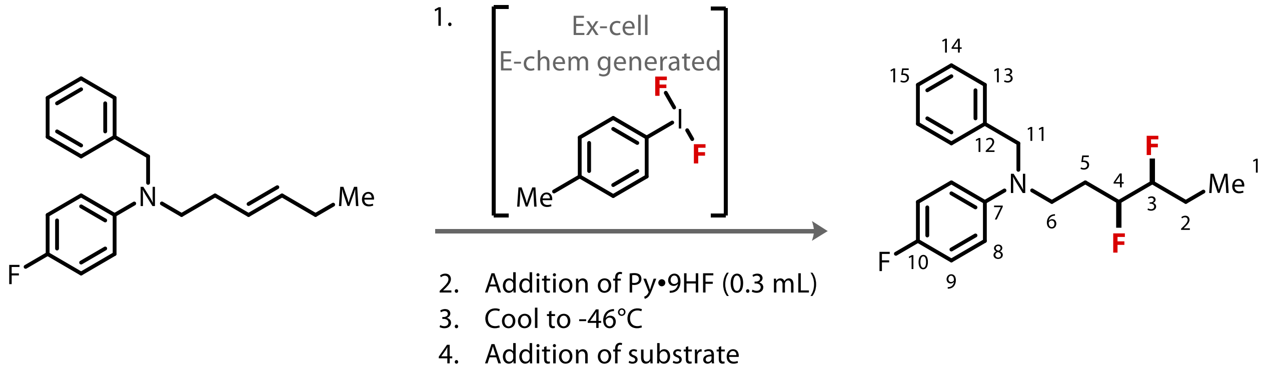

To each compartment of a PTFE divided cell equipped with a Nafion™ membrane and stirrer bars, CH_2Cl_2 (1.5 mL) and 5.6HF:amine stock solution (4.5 mL) were added. To the anodic compartment, *p*-iodotoluene (1 eq., 1.2 mmol, 262 mg) was then added. Each compartment was then capped and wrapped in parafilm. A platinum electrode was inserted into each compartment, and the reaction was subjected to electrolysis (13.4 mA, 2.2 F, 5 hrs). The electrodes were then removed, and 1.1 mL of this solution was added to a 100 mL HDPE vial, equipped with a stirrer bar. Additional py•9HF (0.3 mL) was then added to the reaction mixture. This vial was capped with a Suba-seal and a venting needle was placed through the Suba-seal and was cooled to -46 ºC. Alkene (1.0 м, 0.2 mmol, 58 mg) in CH_2Cl_2 was added then added. After stirring overnight, the reaction mixture was quenched with 200 mL of cold (0 ºC) saturated aqueous $NaHCO_3$ solution. This stirred for 1 hour until the aqueous layer measured pH 7. The mixture was extracted into CH_2Cl_2, dried with Na_2SO_4, concentrated *in vacuo* and isolated using silica-gel column chromatography (2% EtOAc:Hexane) to afford **1g** as a colourless oil (12 mg, 17%).

R_f = 0.30 (4% EtOAc:Hexane)

^{1}H NMR (400 MHz, $CDCl_3$): δ = δ = 7.34 – 7.18 (5H, m, $H^{13,14,15}$), 6.93 – 6.87 (2H, m, H^8), 6.69 – 6.64 (2H, m, H^9), 4.65 - 4.18 (4H, m, $H^{3,4,11}$), 3.65 – 3.45 (2H, m, H^6), 2.12 – 1.58 (4H, m, $H^{2,5}$), 1.00 (3H, J = 6.7 Hz, H^1).

^{19}F NMR (376 MHz, $CDCl_3$): δ = -128.53 – -128.63 (1F, m, F^{10}), -196.32 – -196.78 (1F, m, F^4), -198.70 – -199.23 (1F, m, F^3).

^{13}C {^{1}H} NMR (100 MHz, $CDCl_3$): δ = 155.7 (d, J = 232.6 Hz, C^{10}), 145.0 (C^7), 138.6 (C^{12}), 128.8 (C^{13}), 127.1 (C^{15}), 126.8 (C^{14}), 115.8 (d, J = 22.7 Hz, C^9), 114.2 (d, J = 8.9

Hz, *C*[8]), 94.8 (dd, *J* = 177.9, 21.9 Hz, *C*[4]), 91.1 (dd, *J* = 175.7, 21.0 Hz, *C*[3]), 55.7 (*C*[11]), 47.7 (*C*[6]), 28.6 (dd, *J* = 21.8, 4.3 Hz, *C*[5]), 23.8 (dd, *J* = 22.5, 5.2 Hz, *C*[2]), 9.5 (d, *J* = 5.1 Hz, *C*[1]).

HRMS (ESI) calc: $[M+H]^+$ ($C_{20}H_{25}F_3N$) 322.1704; measured: 322.1707 = 0.93 ppm difference.

IR (neat) ν_{max}/ cm^{-1}: 1587, 1371, 1325, 1254, 1172, 1124.

N-benzyl-N-((3S,4R)-3,4-difluorohexyl)-4-fluoroaniline, 1i

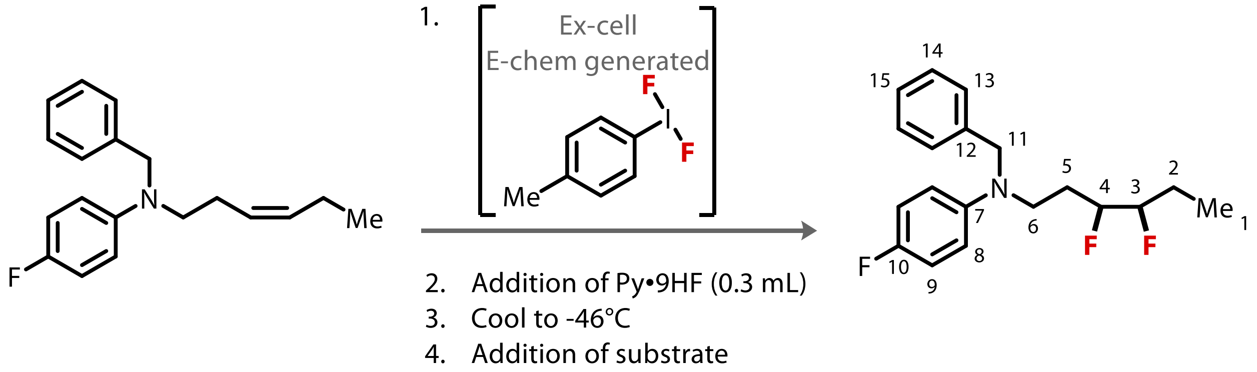

To each compartment of a PTFE divided cell equipped with a Nafion™ membrane and stirrer bars, CH_2Cl_2 (1.5 mL) and 5.6HF:amine stock solution (4.5 mL) were added. To the anodic compartment, *p*-iodotoluene (1 eq., 1.2 mmol, 262 mg) was then added. Each compartment was then capped and wrapped in parafilm. A platinum electrode was inserted into each compartment, and the reaction was subjected to electrolysis (13.4 mA, 2.2 F, 5 hrs). The electrodes were then removed, and 1.1 mL of this solution was added to a 100 mL HDPE vial, equipped with a stirrer bar. Additional py•9HF (0.3 mL) was then added to the reaction mixture. This vial was capped with a Suba-seal and a venting needle was placed through the Suba-seal and was cooled to -46 ºC. Alkene (1.0 м, 0.2 mmol, 58 mg) in CH_2Cl_2 was added then added. After stirring overnight, the reaction mixture was quenched with 200 mL of cold (0 ºC) saturated aqueous $NaHCO_3$ solution. This stirred for 1 hour until the aqueous layer measured pH 7. The mixture was extracted into CH_2Cl_2, dried with Na_2SO_4, concentrated *in vacuo* and isolated using silica-gel column chromatography (2% EtOAc:Hexane) to afford **1i** as a colourless oil (10 mg, 15%).

R_f = 0.30 (4% EtOAc:Hexane)

^{1}H NMR (400 MHz, $CDCl_3$): δ = 7.34 – 7.19 (5H, m, $H^{13,14,15}$), 6.93 – 6.87 (2H, m, H^8), 6.69 – 6.60 (2H, m, H^9), 4.61 – 4.42 (3H, m, $H^{4,11}$), 4.39 – 4.21 (1H, m, H^3), 3.63 – 3.44 (2H, m, H^6), 2.15 – 2.02 (1H, m, H^5), 1.98 – 1.59 (3H, m, $H^{2,5}$), 0.99 (3H, J = 6.7 Hz, H^1).

^{19}F NMR (376 MHz, $CDCl_3$): δ = -128.86 – -128.99 (1F, m, F^{10}), -193.71 – -194.17 (1F, m, F^4), 196.07 – -196.53 (1F, m, F^3).

^{13}C {^{1}H} NMR (100 MHz, $CDCl_3$): δ = 155.6 (d, *J* = 240.9 Hz, C^{10}), 145.0 (C^{7}), 138.7 (C^{12}), 128.8 (C^{13}), 127.1 (C^{15}), 126.8 (C^{14}), 115.8 (d, *J* = 20.8 Hz, C^{9}), 114.1 (C^{8}), 94.9 (dd, *J* = 173.7, 25.2 Hz, C^{4}), 91.1 (dd, *J* = 171.9, 25.1 Hz, C^{3}), 55.4 (C^{11}), 47.7 (C^{6}), 28.1 (C^{5}), 23.8 (dd, *J* = 21.5, 5.0 Hz, C^{2}), 9.5 (d, *J* = 4.5 Hz, C^{1}).

HRMS (ESI) calc: $[M+H]^+$ ($C_{20}H_{25}F_3N$) 322.1777; measured: 322.17067 = 3.10 ppm difference.

IR (neat) ν_{max}/ cm^{-1}: 1587, 1457, 1432, 1391, 1371, 1362, 1325, 1272, 1165, 1124.

Spectroscopic differences between chlorofluoride diastereomers by ^{19}F NMR

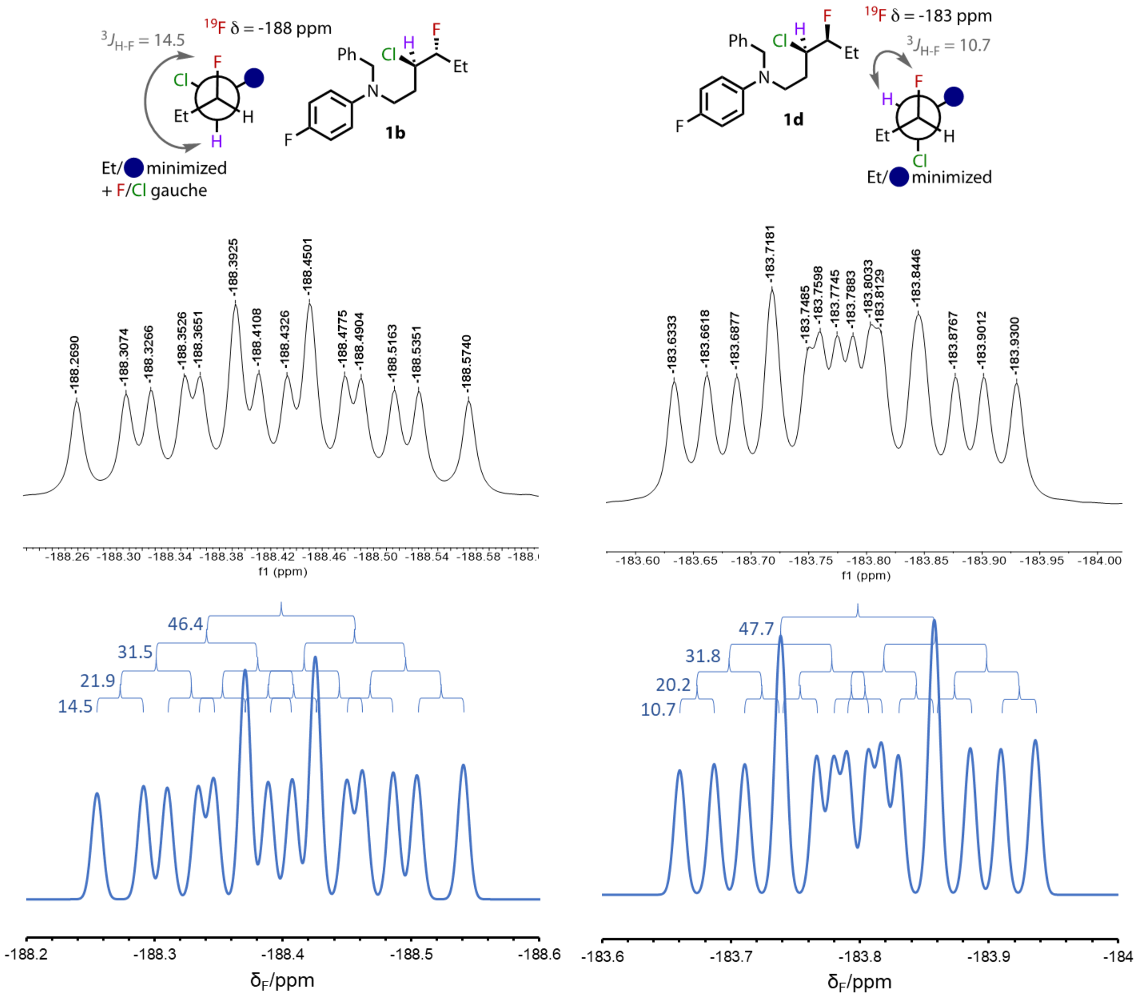

The product **1b** (from *anti*-chlorofluorination of **1a-*cis*** (*cis*-alkene isomer)) exhibits a ^{19}F NMR signal with chemical shift at −188.42 ppm splitting into a dddd pattern pattern, from which can be measured a two-bond coupling constant $^{2}J_{FH}$ 46.4 Hz and three three-bond coupling constants $^{3}J_{FH}$ 31.5, 21.9, 14.5 Hz. A PSYCHE NMR experiment revealed that coupling constant $^{3}J_{FH}$ 14.5 Hz corresponds to the coupling of the *F3* and *H4*.[1]

The product **1d** (from *anti*-chlorofluorination of **1a-*trans*** (*trans*-alkene isomer)) exhibits a more downfield ^{19}F NMR signal with chemical shift at −183.77 ppm and displays a different dddd splitting pattern, from which can be measured a two-bond coupling constant $^{2}J_{FH}$ 47.8 Hz and three three-bond coupling constant $^{3}J_{FH}$ 31.9, 20.1, 10.6 Hz.

The smallest $^{3}J_{FH}$ coupling constant is a diagnostic that differs between the *anti*- and *syn*-chlorofluorides. The non-negligible chemical shift difference between the ^{19}F environments in the *anti*- and *syn*-chlorofluorides suggests that ^{19}F NMR chemical shift for homoallylic amine systems can be used to distinguish between *anti*- and *syn*-chlorofluoride products. Multiplet structure simulations using the above coupling constants render multiplet structures shown above, which well resembles multiplets observed in experimentally recorded ^{19}F NMR spectra. Chemical shift difference is hereon used as the diastereodetermining diagnostic for homoallylic amine-derived chlorofluorination products.

Optimisation

To each compartment of a PTFE divided cell equipped with a Nafion™ membrane and stirrer bars, CH_2Cl_2 (1.5 mL) and 5.6HF:amine stock solution (4.5 mL) were added. To the anodic compartment, *p*-iodotoluene (1 eq., 1.2 mmol, 262 mg) was then added. Each compartment was then capped and wrapped in parafilm. A platinum electrode was inserted into each compartment, and the reaction was subjected to electrolysis (13.4 mA, 2.2 F, 5 hrs). The electrodes were then removed, and 1 mL of this solution was added to a 100 mL HDPE vial, equipped with a stirrer bar. This vial was capped with a Suba-seal and a venting needle was placed through the Suba-seal. Alkene **1a-*cis*** (1.0 M, 0.2 mmol, 58 mg) in CH_2Cl_2 was added first and then chloride source (1 eq., 0.2 mmol). After stirring overnight, the reaction mixture was quenched with 100 mL of cold (0 °C) saturated aqueous $NaHCO_3$ solution. This stirred for 1 hour until the aqueous layer measured pH 7. The mixture was extracted into CH_2Cl_2, dried with Na_2SO_4, filtered, and concentrated *in vacuo*. To the mixture was then added $CDCl_3$ (2 mL) and hexafluorobenzene (1 eq., 0.2 mmol, 23.2 µL), at which point the ^{19}F NMR was measured. The NMR yield was then measured by comparing the integration of the fluorine signal of the product (δ = -128.42 ppm) to that of the internal standard (δ = -161 ppm).

Variation of chloride source

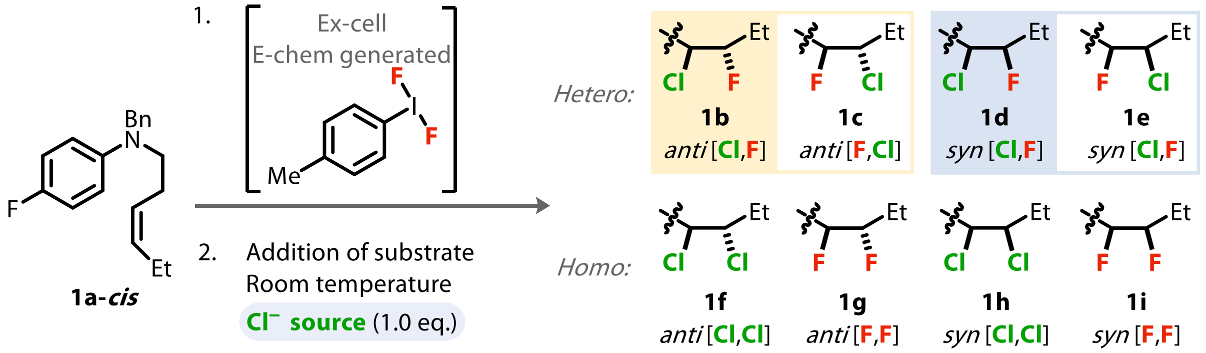

Entry	Cl⁻	1b	1c	1d	1e	1f	1g	1h	1i
1	TEAC	63	12	0	0	9	0	0	0
2	TBAC	51	8	1	0	6	0	0	0
3	$PhMe_3NCl$	53	9	1	0	6	0	0	0
4[a]	NaCl	25	4	0	0	10	0	0	5
5	NH_4Cl	66	10	1	0	22	0	0	0
6	$ZnCl_2$	44	7	0	0	33	0	0	0
7	$MgCl_2$	23	4	0	0	25	0	0	0

Table S1 Numbers given are ^{19}F NMR yields. Standard conditions: *p*-Iodotoluene (1 eq., 1.2 mmol) in 4.5 mL of 5.6 HF:amine and 1.5 mL CH_2Cl_2; electrolysis: 13 mA, 2.2 *F*, divided cell, Pt||Pt. After electrolysis, transfer 1 mL of anodic compartment to HDPE vessel, addition of 1a-*cis* (0.2 mmol), Cl^- source (1 eq., 0.2 mmol) and then overnight stirring. [a] 37% starting material remaining.

Variation of temperature

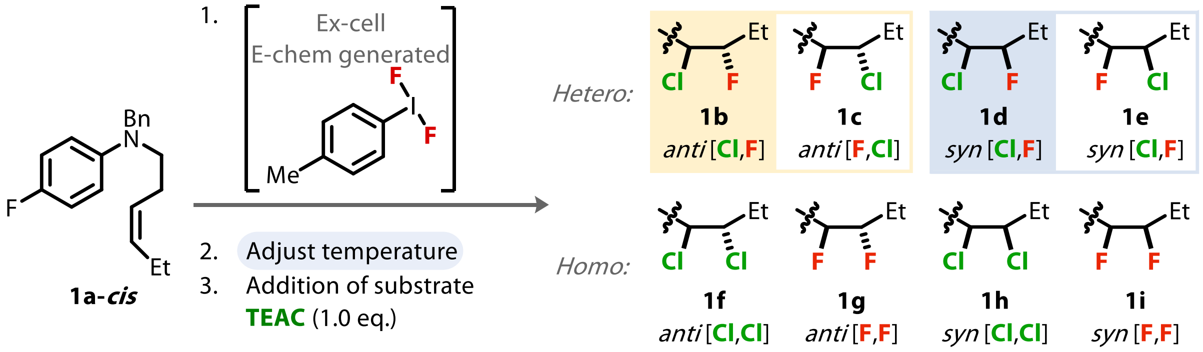

Table S2 Numbers given are ^{19}F NMR yields Standard conditions: *p*-Iodotoluene (1 eq., 1.2 mmol) in 4.5 mL of 5.6 HF:amine and 1.5 mL CH_2Cl_2; electrolysis: 13 mA, 2.2 *F*, divided cell, Pt||Pt. After electrolysis, transfer 1 mL of anodic compartment to HDPE vessel then adjustment of temperature, addition of 1a-*cis* (0.2 mmol), TEAC (1 eq., 0.2 mmol) and then overnight stirring. [a] TEAC (1 eq., 0.2 mmol, 0.2M) in CH_2Cl_2 addition *via* syringe pump over 5 hours.

Entry	Temperature	1b	1c	1d	1e	1f	1g	1h	1i
1	25	63	12	0	0	9	0	0	0
2	0	51	11	0	0	8	0	0	0
3	-20	71	9	0	0	7	0	0	0
4	-46	78	7	0	0	6	0	0	0
5	-78	37	3	0	0	3	0	0	0

Variation of Chloride addition rate

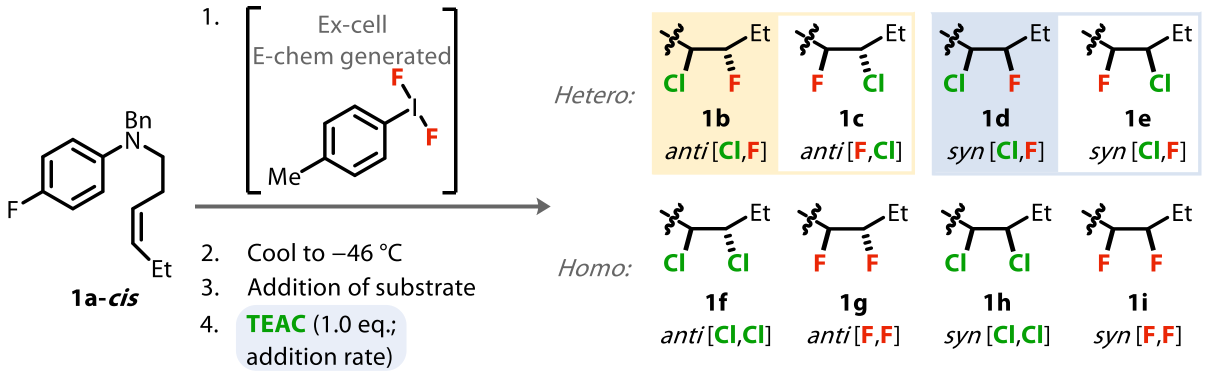

Entry	Addition rate/ (eq./h)	1b	1c	1d	1e	1f	1g	1h	1i
1	0.16	75	5	5	0	0	0	0	1
2	0.18	78	6	3	0	2	0	0	1
3	0.2	85	7	3	0	1	0	0	1
4	0.22	83	7	1	0	1	0	0	0
5	0.24	80	5	1	0	1	0	0	0

Table S3 Numbers given are ^{19}F NMR yields Standard conditions: *p*-Iodotoluene (1 eq., 1.2 mmol) in 4.5 mL of 5.6 HF:amine and 1.5 mL CH_2Cl_2; electrolysis: 13 mA, 2.2 *F*, divided cell, Pt||Pt. After electrolysis, transfer 1 mL of anodic compartment to HDPE vessel then adjustment of temperature, addition of 1a-*cis* (0.2 mmol), TEAC (1 eq., 0.2 mmol, 0.2M) in CH_2Cl_2 addition *via* syringe pump then overnight stirring.

Variation of HF:amine ratio

The HF:amine ratio was adjusted from 5.6 by adding pyridine•9HF. Adjusting to 6HF:amine requires addition of 0.1 mL pyridine•9HF. Adjusting to 6.6HF:amine requires addition of 0.35 mL. Adjusting to 7HF:amine requires addition of 0.55 mL pyridine•9HF. Adjusting to 7.3HF:amine requires addition of 0.8 mL pyridine•9HF. Adjusting to 7.5HF:amine requires addition of 1.0 mL pyridine•9HF.

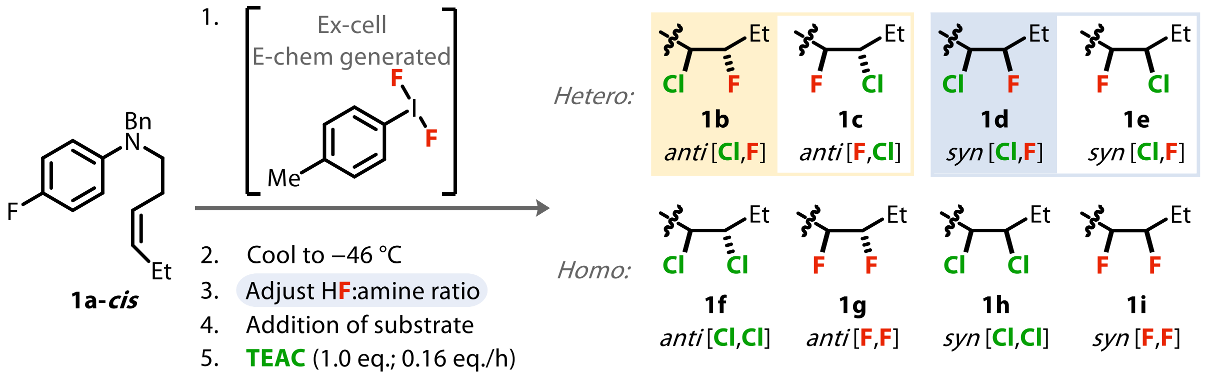

Entry	HF:amine	1b	1c	1d	1e	1f	1g	1h	1i
1	5.6	75	5	5	0	3	0	0	1
2	6	65	4	10	1	2	0	0	3
3	6.6	31	1	33	1	3	0	4	9
4	7	6	0	55	3	6	0	3	5
5	7.3	4	0	41	2	5	0	2	5
6	7.5	3	0	30	2	5	0	3	5
7[a]	7	75	5	3	0	3	0	0	0
8[b]	7	72	4	3	0	4	0	0	0

Table S4 Numbers given are ^{19}F NMR yields Standard conditions: *p*-Iodotoluene (1 eq., 1.2 mmol) in 4.5 mL of 5.6 HF:amine and 1.5 mL CH_2Cl_2; electrolysis: 13 mA, 2.2 *F*, divided cell, Pt||Pt. After electrolysis, transfer 1 mL of anodic compartment to HDPE vessel, adjustment of HF:amine *via* addition of more py•9HF, cool to -46 ºC, addition of 1a-*cis* (0.2 mmol), TEAC (1 eq., 0.2 mmol, 0.2 M) addition *via* a syringe pump (0.17 eq./ hr) and then overnight stirring. [a] TEAC added in portion rather than slow addition; [b] TEAC added in one portion before the substrate.

Variation of acid additive

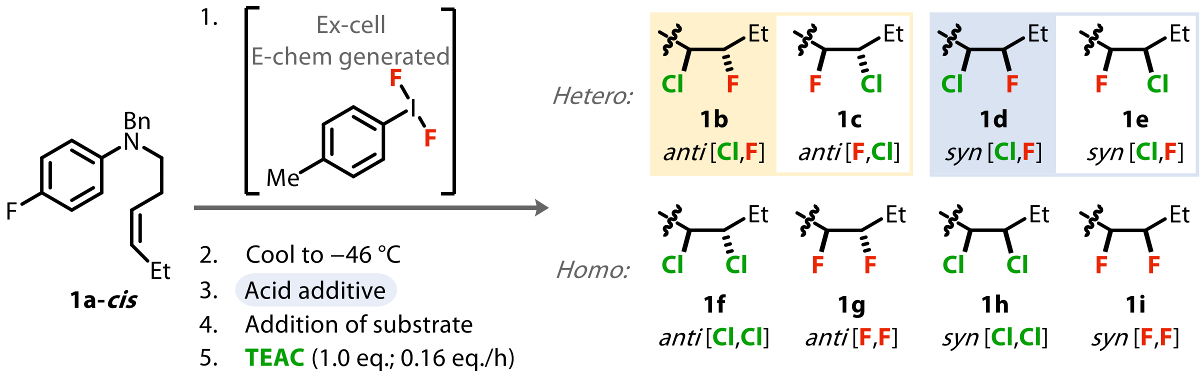

Entry	Acid additive	1b	1c	1d	1e	1f	1g	1h	1i
1	Adjust to 7HF:amine	6	0	55	3	6	0	3	5
2	TFA (0.5 mL)	46	4	27	3	4	0	7	5
3[a]	TFA (0.75 mL)	36	4	37	2	5	0	5	2
4[b]	$HBF_4 \cdot OEt_2$ (0.33 mL)	13	0	51	4	2	0	10	3
5[c]	$HBF_4 \cdot OEt_2$ (0.5 mL)	2	0	45	2	1	0	6	2
6[d]	Adjust to 7HF:amine and add 2.0 eq. TFA	1	0	24	2	0	0	0	1

Table S5 Numbers given are ^{19}F NMR yields Standard conditions: *p*-Iodotoluene (1 eq., 1.2 mmol) in 4.5 mL of 5.6 HF:amine and 1.5 mL CH_2Cl_2; electrolysis: 13 mA, 2.2 *F*, divided cell, Pt||Pt. After electrolysis, transfer 1 mL of anodic compartment to HDPE vessel, addition of acid additive, cool to -46 ºC, addition of 1a-*cis* (0.2 mmol), TEAC (1 eq., 0.2 mmol, 0.2 M) addition *via* a syringe pump (0.16 eq./ hr) and then overnight stirring. [a] 20% uncharacterizable side-products. [b] 9% starting material remaining. [c] 14% starting material remaining. 25% uncharacterizable side-products. [d] 48% uncharacterizable side-products.

Synthesis of substrates

General benzylation procedure 1

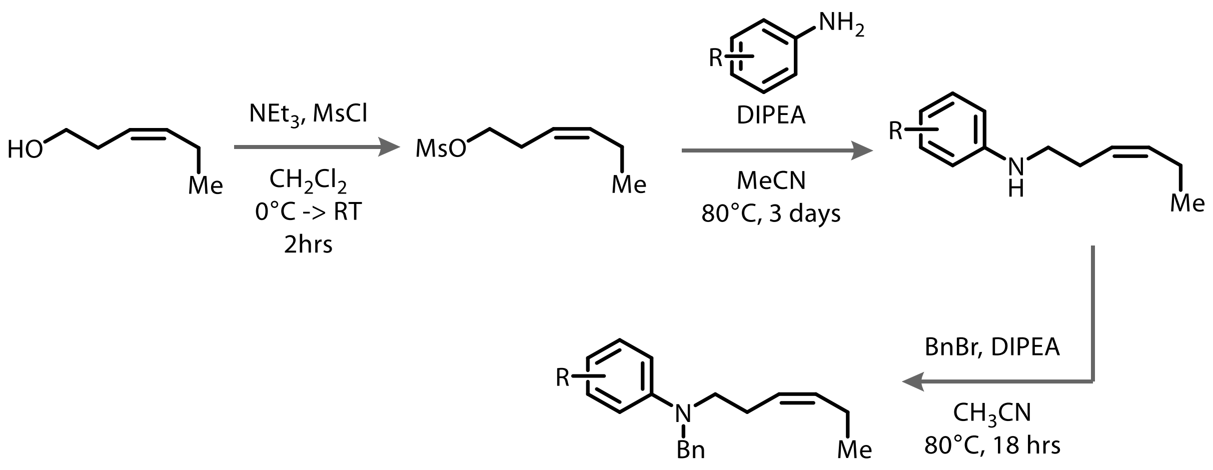

To a round-bottomed flask charged with a stirrer bar was added (Z)-3-hexen-1-ol (1 eq., 10 mmol, 1.18 mL), CH_2Cl_2 (50 mL) and methanesulfonyl chloride (1.5 eq., 15 mmol, 1.15 mL). After the solution was cooled to 0ºC, triethylamine (2 eq., 20 mmol, 2.75 mL) was added dropwise as a colourless precipitate and effervescence was observed. After addition, the solution was allowed to reach room temperature and left to stir for 2 hours. Saturated aqueous K_2CO_3 solution (100 mL) was added, and the organic phase was extracted using CH_2Cl_2 (3 x 50 mL). This mixture was then washed with brine, dried with $MgSO_4$, concentrated in *vacuo,* and used for the next step without further purification.

To a round-bottomed flask charged with a stirrer bar, was added the mesylated alcohol (1 eq., 10 mmol), aniline (2 eq., 20 mmol), DIPEA (5 eq., 50 mmol, 8.9 mL) and acetonitrile (20 mL). This reaction mixture was heated to reflux and allowed to stir for 3 days. The acetonitrile was then removed in *vacuo* and EtOAc (50 mL) and water (50 mL) was added to the remaining reaction mixture. The organic phase was extracted with EtOAc (2 x 50 mL), dried with $MgSO_4$, concentrated in *vacuo* and the mono alkylated aniline was purified using silica-gel column chromatography.

To a round-bottomed flask charged with a stirrer bar was added the mono alkylated aniline (5 mmol), CH_3CN (20 mL), benzyl bromide (1.2 eq., 6 mmol, 1.43 mL) and *N*-diisopropylethylamine (5 eq., 25 mmol, 4.6 mL). This reaction mixture was heated to reflux and allowed to stir overnight. The solvent was then removed in *vacuo* and EtOAc

(50 mL) was added. The organic phase was extracted with EtOAc (2 x 50 mL), dried with MgSO4, and concentrated in *vacuo*. The benzylated amine was purified using silica-gel column chromatography.

General mesylation procedure 2

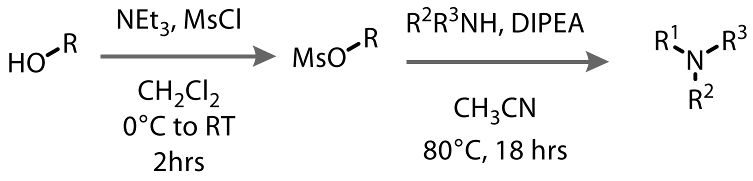

To a round-bottom flask charged with a stirrer bar was added alcohol (1 eq., 10 mmol, 1.18 mL), CH_2Cl_2 (50 mL) and methanesulfonyl chloride (1.5 eq., 15 mmol, 1.15 mL). After the solution was cooled to 0 ºC, triethylamine (2 eq., 20 mmol, 2.75 mL) was added dropwise as a colourless precipitate and effervescence was observed. After addition, the solution was warmed to room temperature and left to stir for 2 hours. Saturated aqueous K_2CO_3 solution (100 mL) was added, and the organic phase was extracted using CH_2Cl_2 (3 x 50 mL). This mixture was then washed with brine, dried with MgSO4, concentrated in *vacuo,* and used for the next step without further purification.

To a round-bottomed flask charged with a stirrer bar, was added the mesylated alcohol (1 eq., 5 mmol), amine (2 eq., 10 mmol), CH_3CN (10 mL) diisopropylethylamine (5 eq., 30 mmol, 5.2 mL). This reaction mixture was heated to reflux and allowed to stir overnight. The reaction mixture was concentrated in *vacuo* and EtOAC (30 mL) was added. The organic phase was extracted with EtOAc (2 x 50 mL), dried with MgSO4, concentrated in *vacuo* and the alkylated amine was purified using silica-gel column chromatography.

Characterisation of substrates

Terminal alkene substrates

4-Methyl-N-(pent-4-en-1-yl)benzenesulfonamide, 2a

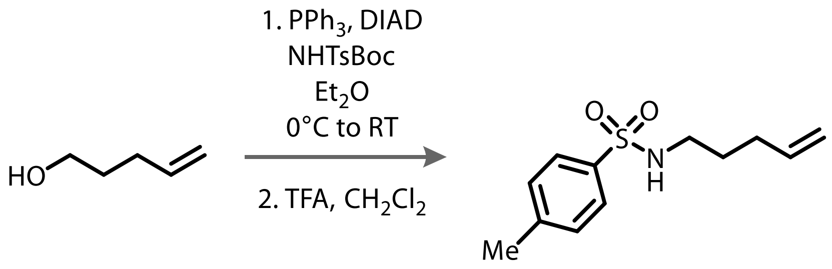

Under a nitrogen atmosphere to a Schlenk tube, was added triphenylphosphine (1 eq., 5 mmol, 1.31 g), Et_2O (20 mL), 4-penten-1-ol (1 eq., 5 mmol, 0.52 mL) and *N*-(tert-butoxycarbonyl)-*p*-toluenesulfonamide (1 eq., 5 mmol, 1.35 g). After the reaction mixture was cooled to 0 ºC, diisopropyl azodicarboxylate (1.1 eq., 5.5 mmol, 1.09 mL) was added dropwise, then a yellow solution was observed. After stirring overnight at room temperature, the white crystalline solid was filtered off, and the reaction mixture was concentrated *in vacuo*. To this yellow residue was added CH_2Cl_2 (10 mL) and trifluoroacetic acid (5 eq., 25 mmol, 1.91 mL). After stirring for 2 hours, saturated aqueous K_2CO_3 (200 mL) was added and the organic phase was extracted with CH_2Cl_2 (3 x 50 mL), dried with $MgSO_4$, concentrated *in vacuo,* and purified using silica-gel column chromatography (30% EtOAc:Hexane) to afford **2a** as a colourless oil (824 mg, 69%), which solidified over several weeks.

R_f = 0.25 (30% EtOAc:Hexane)

^{1}H NMR (500 MHz, $CDCl_3$): δ = 7.73 (2H, d, *J* = 8.2 Hz), 7.26 (2H, d, *J* = 8.2 Hz), 5.74 – 5.59 (1H, m), 5.12 – 4.83 (3H, m), 2.96 – 2.92 (2H, m), 2.40 (3H, s), 1.95 – 1.83 (2H, m), 1.55 – 1.47 (2H, m).

^{13}C {^{1}H} NMR (125 MHz, $CDCl_3$): δ = 143.2, 137.1, 136.9, 129.6, 127.2, 115.4, 42.6, 30.4, 28.3, 21.5.

These data are consistent with those previously reported.[2]

N-allyl-N-(4-methylisoxazol-3-yl)-4-nitrobenzenesulfonamide, 4a

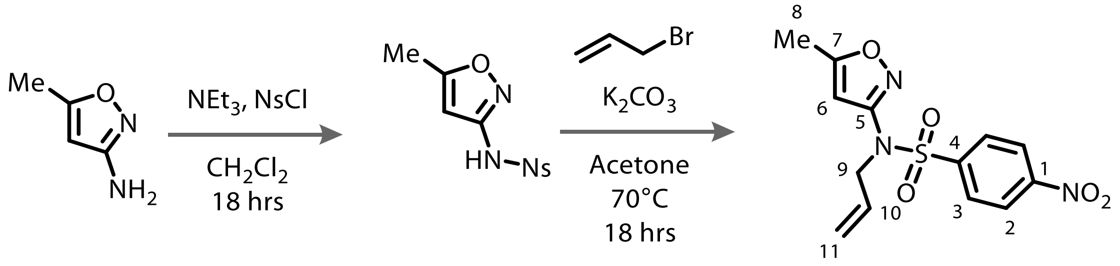

To a round-bottomed flask charged with a stirrer bar was added 3-amino-5-methylisoxazole (1 eq., 5 mmol, 490 mg), CH_2Cl_2 (30 mL) and 4- nitrobenzenesulfonyl chloride (1.1 eq., 5.5 mmol, 1.22 g). Triethylamine (1.5 eq., 7.5 mmol, 1.03 mL) was then added dropwise and the solution was left to stir overnight, after which, a dark colour was observed. The reaction mixed was washed with 1 м HCl solution (20 mL) and the organic phase was extracted with EtOAc (3 x 50 mL). After being dried with $MgSO_4$ and concentrated *in vacuo*, the crude nosylated isoxazole was used without any further purification.

To a round-bottomed flask charged with a stirrer bar was added crude nosylated isoxazole (1 eq., 5 mmol), acetone (30 mL), K_2CO_3 (5 eq., 25 mmol, 3.45 g) and allyl bromide (1.5 eq., 7.5 mmol, 0.65 mL). After stirring overnight at reflux, the reaction mixture was concentrated *in vacuo*, EtOAc (50 mL) and water (50 mL) were added. The organic phase was extracted with EtOAC (2 x 50 mL), dried with $MgSO_4$, concentrated *in vacuo,* and purified using silica-gel column chromatography (30% EtOAC:Hexane) to afford **4a** as a viscous oil (904 mg, 56%).

R_f = 0.20 (30% EtOAc:Hexane)

^{1}H NMR (500 MHz, $CDCl_3$): δ = 8.38 – 8.30 (2H, m, *H^3*), 8.00 – 7.94 (2H, m, *H^2*), 6.36 (1H, s, *H^7*), 5.88 – 5.79 (1H, m, *H^{10}*), 5.34 – 5.27 (1H, m, *H^{11}*), 5.22 – 5.17 (1H, m, *H^{11}*), 4.44 – 4.38 (2H, m, *H^9*), 2.40 (3H, s, *H^8*).

^{13}C {^{1}H} NMR (125 MHz, $CDCl_3$): δ = 171.2 (*C^5*), 159.4 (*C^6*), 150.6 (*C^1*), 144.2 (*C^4*), 131.3 (*C^{10}*), 128.8 (*C^2*), 124.6 (*C^3*), 119.8 (*C^{11}*), 98.5 (*C^7*), 52.0 (*C^9*), 12.9 (*C^8*).

HRMS (APCI) calc: $[M+H]^+$ ($C_{13}H_{14}N_3O_5S$) 324.0649; measured: 324.0656 = 2.46 ppm difference.

IR (neat) ν_{max}/ cm^{-1}: 1608, 1526, 1416, 1362, 1345, 1312, 1291, 1171, 1088.

(S)-quinolin-4-yl((1S,2S,4S,5R)-5-vinylquinuclidin-2-yl)methyl acetate, 11a

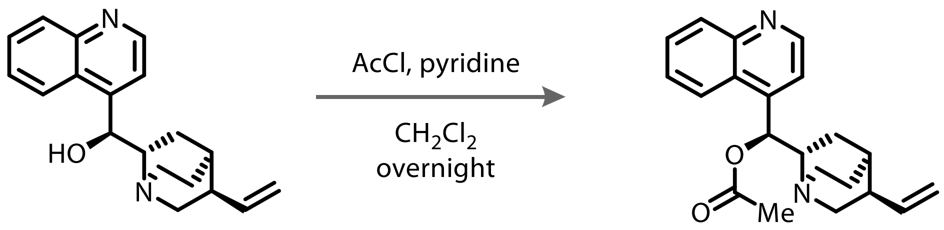

To a round-bottomed flask, acetyl chloride (1 eq., 10 mmol, 0.71 mL), (+)-cinchonine (1 eq., 10 mmol, 2.94 g), CH_2Cl_2 and pyridine (1 eq., 10 mmol, 0.81 mL). After being stirred overnight the reaction mixture was diluted with DCM (10 mL) and washed with H_2O (2 x 20 mL), $NaHCO_3$ (2 x 20 mL) and brine (10 mL). The organic phase was dried using MgSO4, concentrated *in* vacuo and product was purified using silica-gel column chromatography (5% IPA:EtOAc) to afford **11a** as a colourless oil (3.19 g, 95%).

R_f = 0.40 (5% IPA:EtOAc)

^{1}H NMR (500 MHz, $CDCl_3$): δ = 8.87 (1H, d, *J* = 4.5 Hz), 8.19 (1H, d, *J* = 8.3 Hz), 8.10 (1H, d, *J* = 8.3 Hz), 7.70 – 7.67 (1H, m), 7.59 – 7.55 (1H, m), 7.36 (1H, d, *J* = 4.5 Hz), 6.48 (1H, d, *J* = 7.2 Hz), 5.85 – 5.78 (1H, m), 4.98 (1H, d, *J* = 17.8 Hz), 4.97 (1H, d, *J* = 9.1 Hz), 3.41 – 3.35 (1H, m), 3.10 – 3.05 (1H, m), 3.02 – 2.95 (1H, m), 2.60 – 2.54 (2H, m), 2.28 – 2.22 (1H, m), 2.07 (3H, s), 1.89 – 1.78 (2H, m), 1.72 – 1.65 (1H, m), 1.56 – 1.47 (2H, m).

^{13}C {^{1}H} NMR (125 MHz, $CDCl_3$): δ = 170.0, 149.9, 148.3, 145.1, 141.6, 130.3, 129.0, 126.9, 126.2, 123.4, 118.9, 114.7, 74.2, 59.7, 56.4, 42.3, 39.8, 27.5, 27.4, 24.2, 21.1.

These data are consistent with those previously reported.[3]

Benzyl 2-(4-(allyloxy)-3-chlorophenyl)acetate, 13a

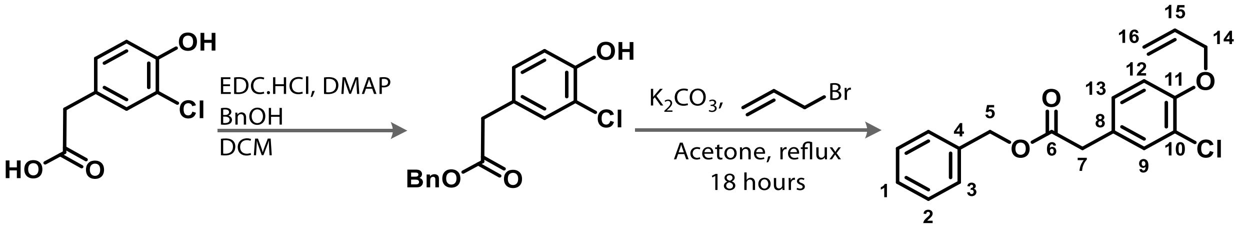

To a round bottom flask equipped with a stirrer bar, was added 2-(3-chloro-4-hydroxyphenyl)acetic acid (1 eq., 930 mg, 5 mmol), EDC•HCl (1.1 eq., 1.05 g, 5.5 mmol), DMAP (0.05 eq., 30 mg, 0.25 mmol) and CH_2Cl_2 (20 mL). After overnight stirring, the organic layer was extracted using EtOAc (3 x 30 mL), dried with $MgSO_4$, filtered, dried in *vacuo* to afford crude phenol product. This was purified using silica gel chromatography (30% EtOAc:Hexane).

To a round bottom flask equipped with a stirrer bar, was added, the phenol isolated previously (5 mmol), K_2CO_3 (5 eq., 3.54 g, 25 mmol), acetone (40 mL) and allyl bromide (3 eq., 1.21 mL, 15 mmol). This reaction mixture was then heated to reflux and left to stir overnight. Afterwards, the reaction mixture was concentrated *in vacuo* and extracted using CH_2Cl_2 (3 x 30 mL). After being dried with $MgSO_4$, the organic phase was dried *in vacuo* and was purified by silica gel column chromatography (20% EtOAc:Hexane) to yield **13a** as a colourless oil (679 mg, 43% over two steps).

R_f = 0.35 (20% EtOAc:Hexane)

^{1}H NMR (500 MHz, $CDCl_3$): δ = 7.40 – 7.29 (6H, m, $H^{1,2,3,9}$), 7.10 (1H, dd, J = 8.4, 2.3 Hz, H^{13}), 6.87 (1H, d, J = 8.5 Hz, H^{12}), 6.06 (1H, ddt, J = 17.2, 10.4, 5.1 Hz, H^{15}), 5.46 (1H, m, H^{16}), 5.36 – 5.26 (1H, m, H^{16}), 5.13 (2H, s, H^7), 4.60 (2H, dt, J = 5.1, 1.6 Hz, H^{14}), 3.58 (2H, s, H^5).

^{13}C {^{1}H} NMR (125 MHz, $CDCl_3$): δ = 171.3 (C^6), 153.4 (C^{11}), 135.8 (C^4), 132.8 (C^{15}), 131.3 (C^9), 128.7 (C^{10}), 128.6 (C^1), 128.5 (C^{13}), 128.3 (C^3), 127.3(C^2), 123.2 (C^8), 118.1 (C^{16}), 113.9 (C^{12}), 69.9 (C^{14}), 66.9 (C^7), 40.2 (C^5).

These data are consistent with those previously reported.[4]

Hex-5-en-1-yl-3,5-dinitrobenzoate, 15a

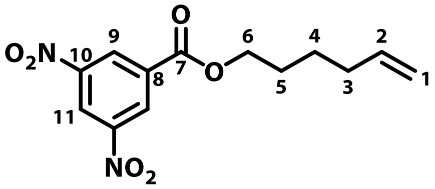

To a round-bottomed flask charged with a stirrer bar was added 5-hexen-1-ol (1 eq., 5 mmol, 0.59 mL), Et3N (1.5 eq., 7.5 mmol, 1.1 mL), and CH2Cl2 (28 mL) and the solution cooled to 0 ℃. 3,5-Dinitrobenzoyl chloride (1.1 eq., 5.5 mmol, 1.27 g) was then added, the solution allowed to warm to RT and stirred overnight. Sat $NaHCO_3$ was then added and the reaction stirred for 10 min. The resulting solution was then extracted with CH_2Cl_2 (3 × 50 mL) and the combined organic layers dried with MgSO4 and concentrated *in vacuo*. The product was purified by silica-gel column chromatography (25% EtOAc:Pentane) to afford **15a** as an off-white solid (1.216 g, 73%).

^{1}H NMR (400 MHz, $CDCl_3$): δ 9.22 (1H, t, *J* = 2.1 Hz, H^{11}), 9.15 (2H, d, *J* = 2.1 Hz, H^9), 5.87-5.77 (1H, tdd, *J* = 10.2, 6.8, 3.2 Hz, H^2), 5.08-4.99 (2H, ddq, *J* = 23.9, 17.1, 1.6 Hz, H^1), 4.46 (2H, t, *J* = 6.8 Hz, H^6), 2.19-2.13 (2H, qt, *J* = 7.3, 1.3 Hz, H^3), 1.89-1.82 (2H, m, H^5), 1.60-1.53 (2H, m, H^4).

^{13}C {^{1}H} NMR (100 MHz, $CDCl_3$): δ 162.7 (C^7), 148.8 (C^{10}), 138.1 (C^2), 134.3 (C^8), 129.6 (C^9), 122.5 (C^{11}), 115.4 (C^1), 67.1 (C^6), 33.3 (C^3), 28.1 (C^5), 25.3 (C^4).

HRMS (MALDI) m/z calc: [M^-] ($C_{13}H_{14}N_2O_6$) 294.0587, measured = 294.0852, 1.70 ppm difference

IR (neat) v_{max}/cm^{-1}: 3102, 2934, 1729, 1545, 1344, 1277, 1167

Internal alkene substrates

(Z)-N-benzyl-N-(hex-3-en-1-yl)-4-(trifluoromethyl)aniline, 16a

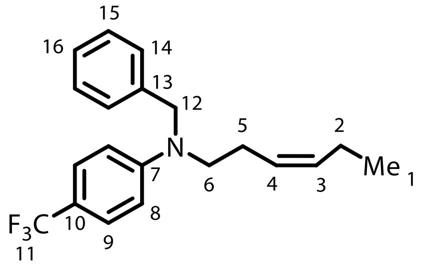

This substrate, **16a**, was synthesised from *p*-trifluoromethylaniline (20 mmol) using **benzylation procedure 1** and was purified by silica-gel column chromatography (2% EtOAc:Hexane) to afford **16a** as a colourless oil (715 mg, 43%).

R_f = 0.15 (2% EtOAc:Hexane)

^{1}H NMR (500 MHz, $CDCl_3$): δ = 7.44 – 7.39 (2H, m, *H^{14}*), 7.35 – 7.30 (2H, m, *H^{15}*), 7.28 – 7.23 (1H, m, *H^{16}*), 7.21 – 7.18 (2H, m, *H^9*), 6.72 – 6.68 (2H, m, *H^8*), 5.55 – 5.48 (1H, m, *H^4*), 5.39 – 5.32 (1H, m, *H^3*), 4.61 (2H, s, *H^{12}*), 3.52 – 3.44 (2H, m, *H^6*), 2.46 – 2.39 (2H, m, *H^5*), 2.09 – 2.00 (2H, m, *H^2*), 0.97 (3H, t, *J* = 7.3 Hz, *H^1*).

^{19}F NMR (376 MHz, $CDCl_3$): δ = -61.15 (3, m, *F^{11}*).

^{13}C {^{1}H} NMR (125 MHz, $CDCl_3$): δ = 150.6 (*C^7*), 138.0 (*C^4*), 134.5 (*C^{13}*), 128.9 (*C^{14}*), 127.2 (*C^{16}*), 126.7 (q, *J* = 3.7 Hz, *C^9*), 126.5 (*C^{15}*), 125.3 (q, *J* = 256.3 Hz, *C^{11}*), 124.9 (*C^3*), 117.7 (*q,* J = 35.6 Hz, *C^{10}*), 111.3 (*C^8*), 54.5 (*C^{12}*), 51.2 (*C^6*), 25.2 (*C^5*), 20.8 (*C^2*), 14.4 (*C^1*).

HRMS (EI) calc: $[M]^+$ ($C_{20}H_{22}F_3N$) 333.1699; measured: 333.1700 = 0.21 ppm difference.

IR (neat) ν_{max}/ cm^{-1}: 1615, 1571, 1530, 1494, 1452, 1399, 1358, 1326, 1247, 1196, 1158, 1106, 1073, 1027.

(Z)-N-benzyl-N-(hex-3-en-1-yl)-4-iodoaniline, 17a

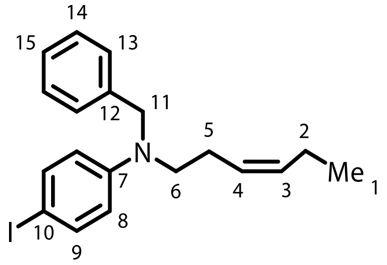

This substrate, **17a**, was synthesised from *p*-iodoaniline (20 mmol) using **benzylation procedure 1** and was purified by silica-gel column chromatography (2% EtOAc:Hexane) to afford **17a** a colourless oil (958 mg, 49%).

R_f = 0.15 (2% EtOAc:Hexane)

^{1}H NMR (500 MHz, $CDCl_3$): δ = 7.50 – 7.38 (2H, m, H^9), 7.34 – 7.17 (5H, m, H^{Ph}), 6.48 (2H, d, *J* = 8.5 Hz, H^8), 5.53 – 5.45 (1H, m, H^4), 5.32 (1H, dtt, *J* = 10.7, 7.4, 1.6 Hz, H^3), 4.52 (2H, s, H^{11}), 3.44 – 3.35 (2H, m, H^6), 2.38 (2H, q, *J* = 7.6 Hz, 1H, H^2), 2.02 (2H, pd, *J* = 7.5, 1.6 Hz, H^5), 0.95 (3H, t, *J* = 7.5 Hz, H^1).

^{13}C {^{1}H} NMR (125 MHz, $CDCl_3$): δ = 147.9 (C^{10}), 137.9 (C^8), 134.3 (C^4), 129.2 (C^{12}), 128.8 (C^{13}), 128.6 (C^7), 127.1 (C^{15}), 126.7 (C^{14}), 125.1 (C^3), 114.7 (C^9), 54.7 (C^{11}), 51.3 (C^6), 25.1 (C^5), 20.8 (C^2), 14.5 (C^1)

HRMS (EI) calc: $[M]^+$ ($C_{29}H_{22}NI$) 391.0791; measured: 391.0793 = 0.51 ppm difference.

IR (neat) ν_{max}/ cm^{-1}: 159, 1498, 1471, 1355, 1317, 1278, 1235.

(Z)-N-benzyl-N-(hex-3-en-1-yl)aniline, 18a

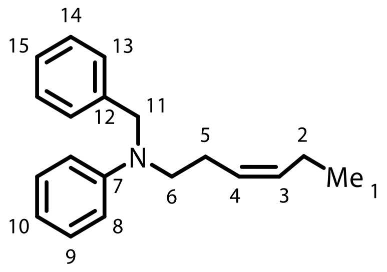

This substrate, **18a**, was synthesised from aniline (20 mmol) using the **benzylation procedure 1** and was purified by silica-gel column chromatography (2% EtOAc:Hexane) to afford **18a** as a colourless oil (675 mg, 51%).

R_f = 0.15 (2% EtOAc:Hexane)

^{1}H NMR (500 MHz, $CDCl_3$): δ = 7.41 – 7.15 (7H, m, $H^{9,13,14,15}$), 6.73 – 6.65 (3H, m, $H^{8,10}$), 5.52 – 5.30 (2H, m, $H^{3,4}$), 4.56 (2H, s, H^{11}), 3.45 – 3.39 (2H, m, H^{6}), 2.48 – 2.33 (2H, m, H^{5}), 2.12 – 1.98 (2H, m, H^{2}), 0.96 (3H, t, *J* = 7.5 Hz, H^{1}).

^{13}C {^{1}H} NMR (125 MHz, $CDCl_3$): δ = 148.5 (C^7), 139.1 (C^{12}), 134.0 (C^4), 129.4 (C^9), 128.7 (C^{13}), 126.9 (C^{15}), 126.7 (C^{14}), 125.5 (C^3), 116.2 (C^{10}), 112.3 (C^8), 54.6 (C^{11}), 51.2 (C^6), 25.2 (C^5), 20.8 (C^2), 14.5 (C^1).

HRMS (ESI) calc: $[M+H]^+$ ($C_{19}H_{24}N$) 266.1903; measured: 266.1911 = 3.20 ppm difference.

IR (neat) ν_{max}/ cm^{-1}: 1703, 1597, 1504, 1451, 1356, 1236, 1205, 1163, 1078.

(Z)-N-benzyl-N-(hex-3-en-1-yl)-4-methylaniline, 19a

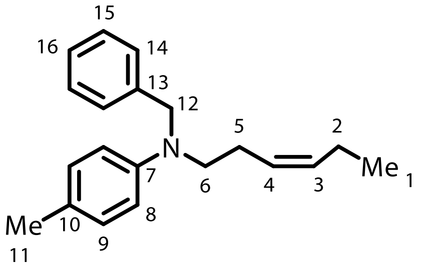

This substrate, **19a**, was synthesised from *p*-methylaniline (20 mmol) using **benzylation procedure 1** and was purified by silica-gel column chromatography (2% EtOAc:Hexane) to afford **19a** as colourless oil (530 mg, 38%).

R_f = 0.15 (2% EtOAc:Hexane)

^{1}H NMR (400 MHz, $CDCl_3$): δ = 7.45 – 7.18 (5H, m, H^{Ph}), 7.07 – 6.93 (2H, m, H^9), 6.67 – 6.53 (2H, m, H^8), 5.54 – 5.28 (2H, m, $H^{3,4}$), 4.53 (2H, s, H^{12}), 3.44 – 3.34 (2H, m, H^6), 2.44 – 2.34 (2H, m, H^5), 2.24 (3H, s, H^{11}), 2.13 – 1.90 (2H, m, H^2), 0.96 (3H, t, *J* = 7.5 Hz, H^1).

^{13}C {^{1}H} NMR (100 MHz, $CDCl_3$): δ = 146.4 (C^{10}), 139.4 (C^{13}), 133.9 (C^4), 129.9 (C^9), 128.6 (C^{14}), 126.8 (C^{16}), 126.7 (C^{15}),125.6 (C^3), 125.4 (C^7), 112.5 (C^8), 54.8 (C^{12}), 51.3 (C^6), 25.2 (C^5), 20.8 (C^2), 20.3 (C^{11}), 14.5 (C^1).

HRMS (EI) calc: $[M]^+$ ($C_{20}H_{25}N$) 279.1982; measured: 279.1981 = 0.36 ppm difference.

IR (neat) ν_{max}/ cm^{-1}: 1703, 1618, 1519, 1451, 1389, 1356, 1190, 1165, 1075.

(E)-4-fluoro-N-(hex-3-en-1-yl)aniline, 20a

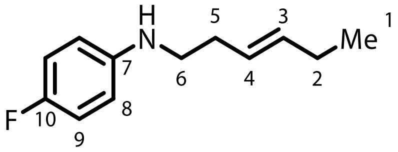

This substrate, **20a**, was synthesised from (E)-3-hexen-1-ol (1 eq., 10 mmol, 1.18 mL) and 4-fluoroaniline (20 mmol) using **mesylation procedure 2** and was purified by silica-gel column chromatography (20% EtOAc:Hexane) to afford **20a** as a yellow oil (1.31 g, 68%).

R_f = 0.45 (20% EtOAc:Hexane)

^{1}H NMR (400 MHz, $CDCl_3$): δ = 6.92 – 6.85 (2H, m, *H^8*), 6.59 – 6.53 (2H, m, *H^9*), 5.64 – 5.55 (1H, m, *H^4*), 5.44 – 5.35 (1H, m, *H^3*), 3.09 (2H, t, *J* = 6.7 Hz, *H^6*), 2.36 – 2.28 (2H, m, *H^5*), 2.11 – 1.97 (2H, m, *H^2*), 0.99 (3H, t, *J* = 7.5 Hz, *H^1*).

^{19}F NMR (376 MHz, $CDCl_3$): δ = -128.14 – -128.22 (1F, m, *F^{10}*).

^{13}C {^{1}H} NMR (100 MHz, $CDCl_3$): δ = 155.9 (d, *J* = 230.1 Hz, *C^{10}*), 144.8 (*C^7*), 134.7 (*C^4*), 125.6 (*C^3*), 115.8 (d, *J* = 22.5 Hz, *C^9*), 113.8 (d, *J* = 7.4 Hz, *C^8*), 44.3 (*C^6*), 27.2 (*C^5*), 20.8 (*C^2*), 14.5 (*C^1*).

HRMS (ESI) calc: $[M+H]^+$ ($C_{12}H_{17}FN$) 194.1340; measured: 194.1348 = 4.20 ppm difference.

IR (neat) ν_{max}/ cm^{-1}: 3405, 1612, 1509, 1315, 1250, 1216, 1108, 1015.

4-(Trifluoromethyl)phenyl (E)-octadec-9-enoate, 23a

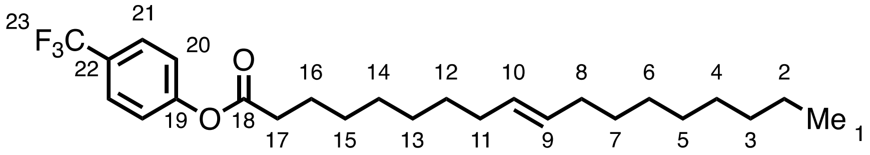

^{1}H NMR (400 MHz, $CDCl_3$): δ = 7.67 – 7.63 (2H, m, H^{21}), 7.25 – 7.20 (2H, m, H^{20}), 5.44 – 5.34 (2H, m, $H^{9,10}$), 2.61 – 2.56 (2H, m, H^{17}), 2.13 – 1.94 (4H, m, $H^{8,11}$), 1.80 – 1.72 (2H, m, H^{16}), 1.61 – 1.19 (20H, m, $H^{2,3,4,5,6,7,12,13,14,15}$), 0.88 (3H, t, *J* = 6.7 Hz, H^{1}).

^{13}C {^{1}H} NMR (100 MHz, $CDCl_3$): δ = 171.9 (C^{18}), 153.4 (C^{19}), 130.7 ($C^{9\ or\ 10}$), 130.3 ($C^{9\ or\ 10}$), 126.0 (q, *J* = 33.8 Hz, C^{22}), 126.9 (q, *J* = 3.8 Hz, C^{21}), 122.7 (q, *J* = 271.9 Hz, C^{23}), 122.2 (C^{20}), 34.5 (C^{17}), 32.8 ($C^{8\ or\ 11}$), 32.7 ($C^{8\ or\ 11}$), 32.1, 29.8, 29.7, 29.6, 29.5, 29.3, 29.2, 29.2, 29.1, 25.0 (C^{16}), 22.8, 14.3 (C^{1}).

HRMS (MALDI) calc: $[M+Na]^+$ ($C_{26}H_{34}O_2F_3$) 435; measured: 449.3.

5-Methyl-4-(2-methylprop-1-en-1-yl)thiazole, 24a

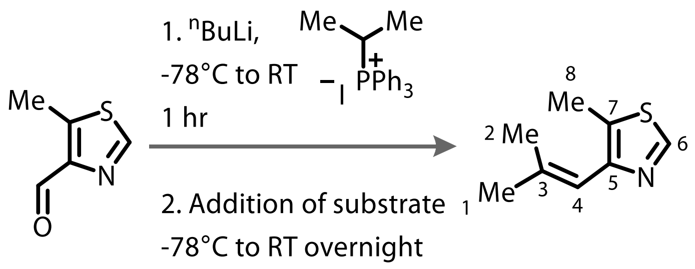

To a flame-dried flask, under nitrogen, at -78ºC, was added isopropyltriphenylphosphonium iodide (1.2 eq., 6 mmol, 2.59 g) and THF (40 mL). To the stirred solution was added nBuLi (1.4 eq., 7 mmol, 4.58 mL, 1.53 M solution in hexane) dropwise. The reaction was stirred for one hour at room temperature before being cooled to -78ºC. 4-Methyl-5-thiazolecarboxaldehyde (1 eq., 5 mmol, 635 mg) in THF (0.5 M) was then added dropwise. The flask was left to stir at room temperature until TLC analysis indicated full consumption of the aldehyde. The reaction was quenched by the addition of brine and EtOAc (30 mL) was added. The organic phase was extracted with EtOAc (2 x 30 mL), washed with brine (20 mL), dried with MgSO4, and concentrated *in vacuo* to afford the crude product. The crude product was purified by silica-gel flash column chromatography (10% EtOAc:Hexane) to afford **24a** as an orange oil (527 mg, 69%).

R_f = 0.30 (10% EtOAc:Hexane)

^{1}H NMR (500 MHz, $CDCl_3$): δ = 8.59 (1H, s, H^6), 6.24 (1H, s, H^4), 2.40 (3H, s, H^8), 1.94 (3H, s, H^2), 1.86 (3H, s, H^1).

^{13}C {^{1}H} NMR (125 MHz, $CDCl_3$): δ = 149.6 (C^7), 149.5 (C^6), 137.9 (C^5), 128.3 (C^3), 114.9 (C^4), 27.1 (C^2), 20.0 (C^1), 15.6 (C^8).

HRMS (APCI) calc: $[M+H]^+$ ($C_8H_{12}NS$) 154.0685; measured: 154.0683 = 1.30 ppm difference.

IR (neat) v_{max}/ cm^{-1}: 1610, 1524, 1439, 1409, 1375, 1316, 1220, 1168, 1078

(Z)-N,N-dibenzylhex-2-en-1-amine, 25a

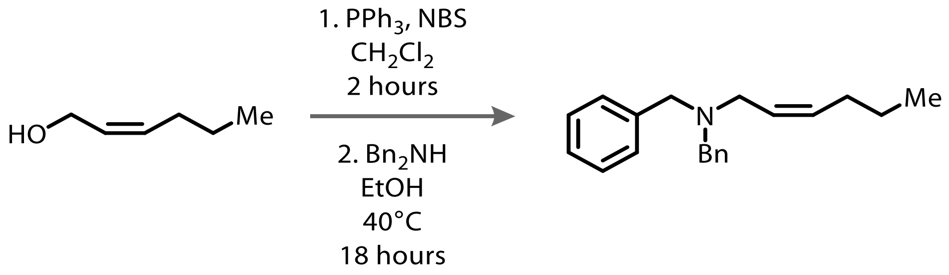

To a round-bottomed flask charged with a stirrer bar was added (Z)-2-hexen-1-ol (1 eq., 10 mmol, 1.18 mL), CH_2Cl_2 (50 mL) and triphenylphosphine (1 eq., 10 mmol, 2.62 g). After the solution was cooled to 0ºC, N-bromo succinimide (1 eq., 10 mmol, 1.78 g) was added portion wise and effervescence was observed. After addition, the solution was allowed to reach room temperature and left to stir for 2 hours. Dibenzylamine (3 eq., 30 mmol, 5.88 mL) and EtOH (50 mL) and the reaction mixture was left to stir overnight at 40ºC. The reaction mixture was then concentrated *in vacuo* and cold Et_2O (20 mL) was added. The filtrate was collected *via* gravity filtration and concentrated *in vacuo*. The crude mixture was purified by silica-gel column chromatography (10% EtOAc:Hexane) to afford **25a** as a colourless oil (1.21 g, 43%).

R_f = 0.50 (10% EtOAc:Hexane)

^{1}H NMR (500 MHz, $CDCl_3$): δ = 7.45 – 7.20 (10H, m), 5.62 – 5.49 (2H, m), 3.58 (4H, s), 3.10 (2H, m), 1.98 (2H, m), 1.38 (2H, m), 0.91 (3H, t, *J* = 6.9 Hz)

^{13}C {^{1}H} NMR (125 MHz, $CDCl_3$): δ = 139.9, 132.8, 128.9, 128.2, 127.1, 126.4, 57.9, 50.1, 29.6, 22.8, 13.8.

These data are consistent with those previously reported.[5]

(Z)-N,N-dibenzylhex-3-en-1-amine, 26a-*cis*

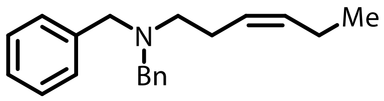

This substrate, **26a-*cis***, was synthesised from (Z)-3-hexen-1-ol (1 eq., 10 mmol, 1.18 mL) and dibenzylamine (20 mmol) using the **mesylation procedure 2** and was purified by silica-gel column chromatography (10% EtOAc:Hexane) to afford **26a-*cis*** as a colourless oil (980 mg, 35%).

R_f = 0.50 (10% EtOAc:Hexane)

^{1}H NMR (500 MHz, $CDCl_3$): δ = 7.40 – 7.20 (10H, m), 5.41 – 5.35 (1H, m), 5.32 – 5.25 (1H, m), 3.59 (4H, s), 2.47 – 2.25 (2H, t, *J* = 7.4 Hz), 2.49 – 2.45 (2H, m), 2.03 – 1.96 (2H, m), 0.91 (3H, t, *J* = 8.7 Hz).

^{13}C {^{1}H} NMR (125 MHz, $CDCl_3$): δ = 140.0, 132.6, 128.9, 128.3, 127.0, 126.9, 58.3, 53.4, 25.0, 20.7, 14.5.

These data are consistent with those previously reported.[5]

(Z)-N,N-dibenzylhex-4-en-1-amine, 27a

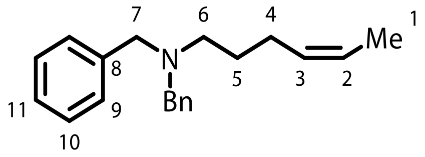

This substrate, **27a**, was synthesised from (Z)-4-hexen-1-ol (1 eq., 10 mmol, 1.18 mL) and dibenzylamine (20 mmol) using **mesylation procedure 2** and was purified by silica-gel column chromatography (10% EtOAc:Hexane) to afford **27a** as a colourless oil (1.59 g, 57%).

R_f = 0.50 (10% EtOAc:Hexane)

^{1}H NMR (500 MHz, $CDCl_3$): δ = 7.42 – 7.18 (10H, m), 5.47 – 5.26 (2H, m), 3.57 (4H, s), 2.48 – 2.42 (2H, m), 2.07 – 1.99 (2H, m), 1.62 – 1.54 (5H, m).

^{13}C {^{1}H} NMR (125 MHz, $CDCl_3$): δ = 140.1, 130.6, 128.9, 128.3, 126.9, 124.0, 58.4, 53.3, 27.2, 24.8, 12.9.

These data are consistent with those previously reported.[5]

(Z)-tert-butyl(hex-3-en-1-yloxy)diphenylsilane, 29a

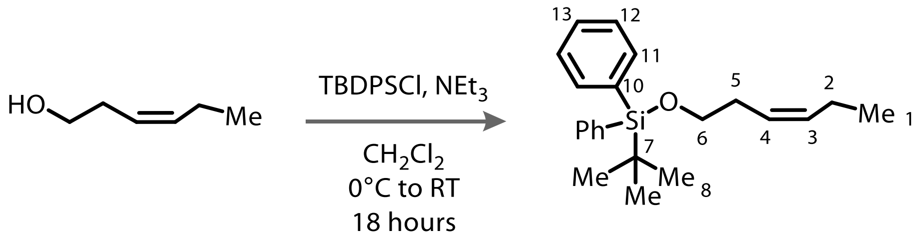

To a round-bottomed flask charged with a stirrer bar was added (Z)-3-hexen-1-ol (1 eq., 10 mmol, 1.18 mL), CH_2Cl_2 (50 mL) and tert-butyldiphenylsilyl chloride (1.5 eq., 15 mmol, 4.03 mL). After the solution was cooled to 0ºC, triethylamine (2 eq., 20 mmol, 2.75 mL) was added dropwise and effervescence was observed. After addition, the solution was allowed to reach room temperature and left to stir for 2 hours. Saturated aqueous K_2CO_3 solution (100 mL) was added, and the organic phase was extracted using CH_2Cl_2 (3 x 50 mL). This mixture was then washed with brine, dried with MgSO4, concentrated *in vacuo,* and purified by silica-gel column chromatography (5% EtOAc:Hexane) to afford **29a** as a colourless oil (3 g, 89%).

R_f = 0.60 (5% EtOAc:Hexane)

^{1}H NMR (500 MHz, $CDCl_3$): δ = 7.80 – 7.28 (10H, m), 5.48 – 5.27 (2H, m), 3.68 – 3.58 (2H, m), 2.35 – 2.28 (2H, m), 2.07 – 1.95 (2H, m), 1.15 – 1.02 (2H, m), 0.97 (9H, t, *J* = 6.8 Hz).

^{13}C {^{1}H} NMR (125 MHz, $CDCl_3$): δ = 135.6, 134.0, 133.4, 129.1, 127.4, 124.8, 77.3, 77.4, 77.1, 76.7, 63.9, 30.6, 26.1, 20.6, 19.2, 14.3.

These data are consistent with those previously reported.[6]

4-Fluorophenyl (E)-but-2-enoate, 30a

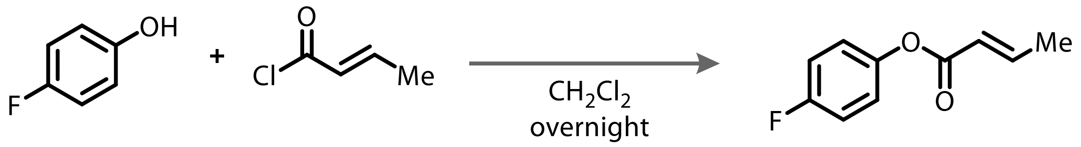

To a round-bottomed flask, (E)-crotonyl chloride (1 eq., 10 mmol, 1.05 g), 4-fluorophenol (1 eq., 10 mmol, 1.96 mL) and CH_2Cl_2 were added. After being stirred overnight the reaction mixture was diluted with DCM (10 mL) and washed with H_2O (2 x 20 mL), $NaHCO_3$ (2 x 20 mL) and brine (10 mL). The organic phase was dried using MgSO4, concentrated *in* vacuo and product was purified using silica-gel column chromatography (25% EtOAc:Hexane) to afford **30a** as a colourless oil (1.35 g, 75 %).

R_f = 0.25 (25% EtOAc:Hexane)

^{1}H NMR (500 MHz, $CDCl_3$): δ = 7.28 – 7.03 (4H, m), 6.11 – 5.91 (1H, m), 5.27 (1H, d, *J* = 14.2 Hz), 1.91 (3H, d, *J* = 6.7 Hz).

^{19}F NMR (376 MHz, $CDCl_3$): δ = -115.58 – -115.63 (1F, m).

^{13}C {^{1}H} NMR (125 MHz, $CDCl_3$): δ = 170.0, 160.1, 146.4 (d, *J* = 230.1 Hz), 130.9 , 122.7 (d, *J* = 22.3 Hz), 119.2, 115.9 (d, *J* = 7.2 Hz), 38.7.

These data are consistent with those previously reported.[2]

(E)-N,N-dibenzylbut-2-enamide, 31a

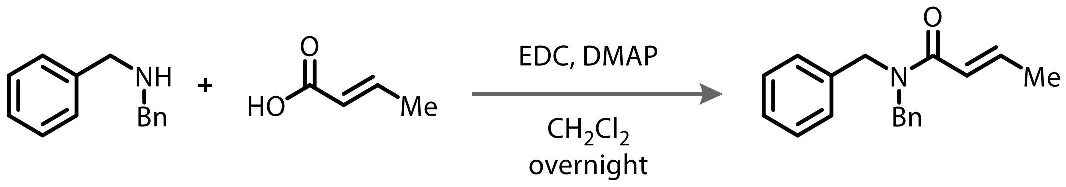

To a round-bottomed flask, (E)-crotonic acid (1 eq., 10 mmol, 870 mg), dibenzylamine (1 eq., 10 mmol, 1.96 mL), CH_2Cl_2 (40 mL), EDC•HCl (1.1 eq., 11 mmol, 2.12 g) and DMAP (0.25 eq., 2.5 mmol, 305 mg) were added. The solution was stirred overnight and then the reaction mixture was diluted with DCM (10 mL) and washed with H_2O (2 x 20 mL), $NaHCO_3$ (2 x 20 mL) and brine (10 mL). The organic phase was dried using MgSO4, concentrated *in* vacuo and product was purified using silica-gel column chromatography (30% EtOAc:Hexane) to afford **31a** a colourless solid (1.35 g, 51 %).

R_f = 0.35 (30% EtOAc:Hexane)

^{1}H NMR (500 MHz, $CDCl_3$): δ = 7.42 – 7.16 (10H, m), 7.07 (1H, dq, *J* = 14.9, 6.9 Hz), 6.30 (1H, dq, *J* = 14.9, 1.7 Hz), 4.63 (2H, s), 4.50 (2H, s), 1.87 (3H, dd, *J* = 6.9, 1.7 Hz).

^{13}C {^{1}H} NMR (125 MHz, $CDCl_3$): δ = 167.5, 143.3, 137.6, 136.7, 128.7, 128.6, 128.2, 127.5, 127.5, 126.6, 121.9, 49.9, 48.4, 18.4.

These data are consistent with those previously reported.[7]

1-Tosyl-2,5-dihydro-1H-pyrrole, 32a

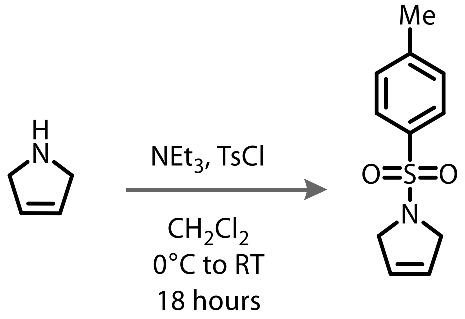

To a round-bottomed flask charged with a stirrer bar was added 3-pyrroline (1 eq., 10 mmol, 1.18 mL), CH_2Cl_2 (50 mL) and *p*-toluenesulfonyl chloride (1.5 eq., 15 mmol, 2.85 g). After the solution was cooled to 0ºC, triethylamine (2 eq., 20 mmol, 2.75 mL) was added dropwise and effervescence was observed. After addition, the solution was allowed to reach room temperature and left to stir overnight. 1 м HCl solution (100 mL) was added, and the organic phase was extracted using CH_2Cl_2 (3 x 50 mL). This mixture was then washed with brine, $MgSO_4$, concentrated *in vacuo* and purified by silica-gel column chromatography (20% EtOAc:Hexane) to afford **32a** as a colourless oil (2 g, 90%).

R_f = 0.30 (20% EtOAc:Hexane)

^{1}H NMR (500 MHz, $CDCl_3$): δ = 7.73 (2H, d, *J* = 8.3 Hz), 7.31 (2H, d, *J* = 8.3 Hz), 5.65 (2H, s), 4.14 (4H, s), 2.45 (3H, s).

^{13}C {^{1}H} NMR (125 MHz, $CDCl_3$): δ = 143.2, 134.4, 129.5, 125.3, 54.9, 21.4.

These data are consistent with those previously reported.[8]

tert-Butyl-(Z)-9-(hex-3-en-1-yl)-3,9-diazaspiro[5.5]undecane-3-carboxylate, 34a

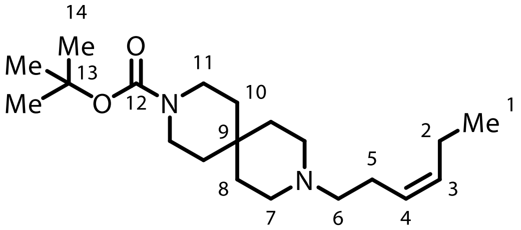

Synthesised from (*Z*)-hex-3-en-1-ol (1 eq., 5 mmol, 0.60 mL) and *tert*-butyl 3,9-diazaspiro[5.5]undecane-3-carboxylate (1 eq., 5 mmol, 1.75 g) using the **general mesylation procedure 2**. The product was purified by silica-gel column chromatography (10% IPA: $CHCl_3$) to afford **34a** as a yellow/orange oil (1.001 g, 59%).

^{1}H NMR (400 MHz, $CDCl_3$): δ 5.43-5.36 (1H, dtt, *J* = 10.7, 7.3, 1.3, H^3), 5.32-5.25 (1H, dtt, *J* = 10.7, 7.3, 1.3 Hz, H^4), 3.34 (4H, m, H^7), 2.40 (4H, t, *J* = 4.8 Hz, H^{11}), 2.37-2.33 (2H, m, H^6), 2.25-2.19 (2H, m, H^5), 2.06-1.98 (2H, dp, *J* = 7.5, 1.3 Hz, H^2), 1.52 (4H, m, H^8), 1.43 (9H, s, H^{14}), 1.40 (4H, m, H^{10}), 0.93 (3H, t, *J* = 7.5 Hz, H^1).

^{13}C {^{1}H} NMR (100 MHz, $CDCl_3$): δ 155.1 (C^{12}), 133.0 (C^3), 126.4 (C^4), 79.3 (C^{13}), 58.9 (C^6), 49.2 (C^{11}), 39.5 (C^7), 35.6 (C^{10}), 35.4 (C^8), 29.7 (C^9), 28.6 (C^{14}), 25.1 (C^5), 20.7 (C^2), 14.4 (C^1).

HRMS (ESI) m/z calc: [M+H$^+$] ($C_{20}H_{36}N_2O_2$) 337.2850, measured = 337.2850, 0.00 ppm difference.

IR (neat) v_{max}/cm^{-1}: 2928, 1675, 1427, 1366, 1162.

(E)-hex-3-en-1-yl 3,5-dinitrobenzoate, 36a

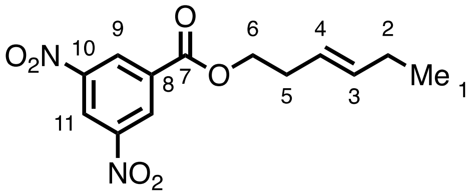

^{1}H NMR (400 MHz, CDCl$_3$): δ = 9.25 – 9.22 (1H, m, H^{11}), 9.17 – 9.15 (1H, m, H^9), 5.69 – 5.62 (1H, m, H^4), 5.47 – 5.39 (1H, m, H^3), 4.45 (2H, t, J = 7.1 Hz, H^6), 2.60 – 2.49 (1H, m, H^5), 2.10 – 2.01 (1H, m, H^2), 0.97 (3H, t, J = 6.7 Hz, H^1).

^{13}C {^{1}H} NMR (100 MHz, CDCl$_3$): δ = 162.6 (C^7), 148.8 (C^{10}), 136.2 (C^4), 134.3 (C^{10}), 129.5 (C^9), 123.4 (C^3), 122.4 (C^{11}), 66.5 (C^6), 32.0 (C^5), 25.8 (C^2), 13.8 (C^1).

HRMS (MALDI) m/z calc: [M$^-$] ($C_{13}H_{14}N_2O_6$) 294.0857, measured = 294.0855, 0.68 ppm difference.

(*Z*)-2-(1-(Hex-3-en-1-yl)piperidin-4-yl)-4,6-dimethoxypyrimidine, 37a

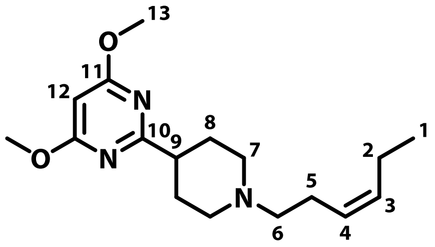

Synthesised from (*Z*)-hex-3-en-1-ol (1 eq., 5 mmol, 0.60 mL) and 4,6-Dimethoxy-2-(piperidin-4-yl)pyrimidine (1 eq., 5 mmol, 1.15 g) using the **general mesylation procedure 2**. The product was purified by silica-gel column chromatography (10% IPA: $CHCl_3$ + 1% Et_3N) to afford **37a** as an orange oil (734 mg, 48%).

^{1}H NMR (400 MHz, $CDCl_3$): δ 5.84 (1H, s, H^{12}), 5.45-5.39 (1H, m, H^3), 5.35-5.29 (1H, m, H^4), 3.90 (6H, s, H^{13}), 3.03 (2H, dt, *J*= 7.5, 1.5 Hz, H^7), 2.70-2.62 (1H, m, H^9), 2.41-2.37 (2H, m, H^6), 2.30-2.25 (2H, m, H^5), 2.09-2.02 (4H, m, H^2, $H^{7'}$), 2.00-1.96 (4H, m, H^8), 0.96 (3H, t, *J* = 7.5 Hz, H^1).

^{13}C {^{1}H} NMR (100 MHz, $CDCl_3$): δ 172.8 (C^{10}), 171.5 (C^{11}), 133.0 (C^3), 126.6 (C^4), 87.0 (C^{12}), 59.0 (C^6), 53.9 (C^{13}), 53.8 (C^7), 45.1 (C^9), 30.8 (C^8), 25.2 (C^5), 20.7 (C^2), 14.5 (C^1).

HRMS (ESI) m/z calc: [M+H$^+$] ($C_{17}H_{27}N_3O_2$) 306.2176, measured = 306.2175, 0.32 ppm difference.

IR (neat) ν_{max}/cm^{-1}: 2950, 1585, 1566, 1371, 1187.

4-fluorobenzyl (Z)-hex-3-enoate, 38a-cis

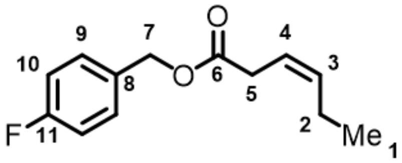

In a 100-mL RBF, (*Z*)-hex-3-en-1-ol (2.36 mL, 20 mmol, 1.0 eq.) was dissolved in MeCN/H_2O (1:1 v/v, 40 mL) and PIDA (14.2 g, 44 mmol, 2.2 eq.) and TEMPO (625 mg, 4.0 mmol, 0.2 eq.) were added sequentially. The reaction mixture was stirred at room temperature for 3 h and then diluted with water. Then, aqueous 3 м NaOH (50 mL) was added and EtOAc (50 mL). The aqueous layer was collected, acidified to pH 2 with concentrated aqueous HCl, extracted with EtOAc, the organic layer collected, dried over $MgSO_4$, filtered and concentrated under vacuum to afford (*Z*)-hex-3-enoic acid as a brown residue which was used in the next step without further purification. In a 250-mL RBF, (*Z*)-hex-3-enoic acid was dissolved in toluene (100 mL) and 4-fluorobenzyl alcohol (4.3 mL, 40 mmol, 2 eq.) and *p*-TsOH (761 mg, 4.0 mmol, 0.2 eq.) were added sequentially. The RBF was equipped with a Dean-Stark apparatus and the reaction mixture was stirred at 150 °C overnight. After cooling to room temperature, the reaction mixture was concentrated under vacuum to afford a brown residue which was purified by column chromatography on silica gel (5% EtOAc/pentane) to afford **38a-cis** as a pale yellow oil (1.33 g, 33%).

^{1}H NMR (400 MHz, $CDCl_3$) 7.33 (dd, *J* = 8.5, 5.5 Hz, 2H, *H10*), 7.04 (t, *J* = 8.7 Hz, 2H, *H9*), 5.55 (m, 2H, *H3*, *H4*), 5.09 (s, 2H, *H7*), 3.13 (d, *J* = 6.7 Hz, 2H, *H5*), 2.05 (t, *J* = 7.3 Hz, 2H, *H2*), 0.96 (t, *J* = 7.5 Hz, 3H, *H1*).

^{19}F NMR (376 MHz, $CDCl_3$) δ -113.58 (tt, *J* = 8.7, 5.3 Hz, *F11*).

^{13}C NMR (101 MHz, $CDCl_3$) δ 171.97 (*C6*), 162.78 (d, *J* = 246.9 Hz, *C11*), 135.50, 131.91 (d, *J* = 3.1 Hz, *C8*), 130.34 (d, *J* = 8.2 Hz, *C9*), 120.00, 115.62 (d, *J* = 21.5 Hz, *C10*), 65.83 (*C7*), 32.98 (*C5*), 20.87 (*C2*), 14.03 (*C1*).

HRMS (EI) calc: $[M]^+$ ($C_{13}H_{15}FO_2$) 222.1051; measured: 222.1046 = 2.25 ppm difference.

4-fluorobenzyl (*E*)-hex-3-enoate, 38a-trans

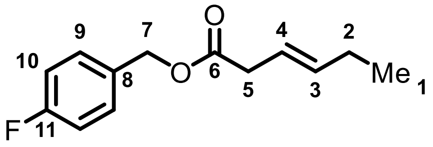

In a 100-mL RBF, (*Z*)-hex-3-en-1-ol (2.36 mL, 20 mmol, 1.0 eq.) was dissolved in MeCN/H_2O (1:1 v/v, 40 mL) and PIDA (14.2 g, 44 mmol, 2.2 eq.) and TEMPO (625 mg, 4.0 mmol, 0.2 eq.) were added sequentially. The reaction mixture was stirred at room temperature for 3 h and then diluted with water. Then, aqueous 3 м NaOH (50 mL) was added and EtOAc (50 mL). The aqueous layer was collected, acidified to pH 2 with concentrated aqueous HCl, extracted with EtOAc, the organic layer collected, dried over $MgSO_4$, filtered and concentrated under vacuum to afford (*Z*)-hex-3-enoic acid as a brown residue which was used in the next step without further purification. In a 250-mL RBF, (*Z*)-hex-3-enoic acid was dissolved in toluene (100 mL) and 4-fluorobenzyl alcohol (4.3 mL, 40 mmol, 2 eq.) and *p*-TsOH (761 mg, 4.0 mmol, 0.2 eq.) were added sequentially. The RBF was equipped with a Dean-Stark apparatus and the reaction mixture was stirred at 150 °C overnight. After cooling to room temperature, the reaction mixture was concentrated under vacuum to afford a brown residue which was purified by column chromatography on silica gel (5% EtOAc/pentane) to afford **38a-trans** as a pale yellow oil (1.56 g, 35%).

^{1}H NMR (400 MHz, $CDCl_3$) 7.32 (dd, *J* = 8.3, 5.5 Hz, 2H, *H10*), 7.03 (t, *J* = 8.7 Hz, 2H, *H9*), 5.60 (dt, *J* = 15.4, 6.0 Hz, 1H, *H3*), 5.50 (dt, *J* = 15.5, 6.6 Hz, 1H, *H4*), 5.07 (s, 2H, *H7*), 3.05 (d, *J* = 6.6 Hz, 2H, *H5*), 2.03 (q, *J* = 7.4 Hz, 2H, *H2*), 0.97 (t, *J* = 7.4 Hz, 3H, *H1*).

^{19}F NMR (376 MHz, $CDCl_3$) -113.61 (tt, *J* = 8.7, 5.3 Hz, *F11*)

^{13}C NMR (101 MHz, $CDCl_3$) 172.12 (*C6*), 162.72 (d, *J* = 246.8 Hz, *C11*), 136.73 (*C3*), 131.90 (d, *J* = 3.2 Hz, *C8*), 130.29 (d, *J* = 8.2 Hz, *C9*), 120.37 (*C4*), 115.56 (d, *J* = 21.6 Hz, *C10*), 65.70 (*C7*), 38.10 (*C5*), 25.60 (*C2*), 13.53 (*C1*).

HRMS (EI) calc: $[M]^+$ ($C_{13}H_{15}FO_2$) 222.1051; measured: 222.1046 = 2.25 ppm difference.

(Z)-2-(hex-3-en-1-yl)isoindoline, 40a-cis

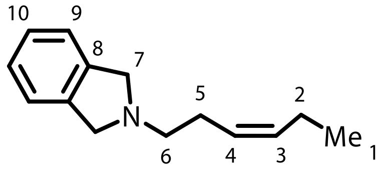

40a-*cis* was synthesised from (Z)-3-hexen-1-ol (1 eq., 1 mmol, 0.12 mL) and isoindoline (2 eq., 2 mmol, 0.24 mL) using **mesylation procedure 2** and was purified by silica-gel column chromatography (5% IPA:EtOAc) to afford **40a-*cis*** as a brown oil (161 mg, 81%).

R_f = 0.40 (5% IPA:EtOAc)

^{1}H NMR (500 MHz, $CDCl_3$): δ = 7.22 – 7.16 (4H, m, $H^{9,10}$), 5.51 – 5.37 (2H, m, $H^{3,4}$), 3.97 (4H, s, H^7), 2.77 (2H, dd, *J* = 8.3, 7.0 Hz, H^6), 2.42 – 2.31 (2H, m, H^5), 2.11 (2H, pd, *J* = 7.4, 1.3 Hz, H^2), 0.99 (3H, t, *J* = 7.5 Hz, H^1).

^{13}C {^{1}H} NMR (125 MHz, $CDCl_3$): δ = 140.0 (C^8), 133.3 (C^4), 126.9 (C^{10}), 126.3 (C^3), 122.4 (C^9), 59.2 (C^7), 56.2 (C^6), 27.0 (C^5), 20.8 (C^2), 14.5 (C^1).

HRMS (ESI) calc: $[M+H]^+$ ($C_{14}H_{20}NO$) 170.1539; measured: 170.1545 = 3.20 ppm difference.

IR (neat) v_{max}/ cm^{-1}: 1693, 1665, 1578, 1517, 1478, 1462, 1378.

(E)-2-(hex-3-en-1-yl)isoindoline, 40a-trans

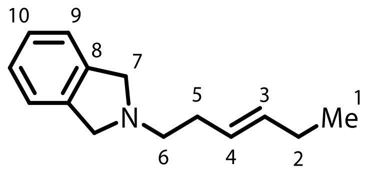

40a-*trans* was synthesised from (*E*)-3-hexen-1-ol (1 eq., 1 mmol, 0.12 mL) and isoindoline (2 eq., 2 mmol, 0.24 mL) using **mesylation procedure 2** and was purified by silica-gel column chromatography (5% IPA:EtOAc) to afford **40a-*trans*** as a brown oil (105 mg, 57%).

R_f = 0.40 (5% IPA:EtOAc)

^{1}H NMR (500 MHz, $CDCl_3$): δ = 7.21 – 7.16 (4H, m, $H^{9,10}$), 5.61 – 5.44 (2H, m, $H^{3,4}$), 3.95 (4H, s, H^7), 2.80 – 2.75 (2H, m, H^6), 2.34 – 2.27 (2H, m, H^5), 2.03 (2H, pd, *J* = 7.4, 1.3 Hz, H^2), 0.98 (3H, t, *J* = 7.5 Hz, H^1).

^{13}C {^{1}H} NMR (125 MHz, $CDCl_3$): δ = 140.1 (C^8), 133.7 (C^4), 126.8 (C^{10}), 126.7 (C^3), 122.4 (C^9), 59.2 (C^7), 56.4 (C^6), 32.4 (C^5), 25.8 (C^2), 14.0 (C^1).

HRMS (ESI) calc: $[M+H]^+$ ($C_{14}H_{20}NO$) 202.1590; measured: 202.1591 = 0.49 ppm difference.

IR (neat) ν_{max}/ cm^{-1}: 1699, 1631, 1515, 1501, 1493, 1461, 1417, 1378, 1271.

(Z)-6-fluoro-3-(1-(hex-3-en-1-yl)piperidin-4-yl)benzo[d]isoxazole, 41a

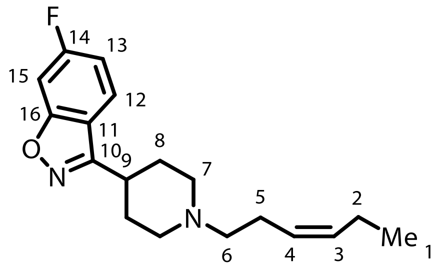

41a, was synthesised from (Z)-3-hexen-1-ol (1 eq., 2 mmol, 0.24 mL) and 6-fluoro-3-(4-piperidinyl)benzisoxazole (1 eq., 2 mmol, 440 mg) using **mesylation procedure 2** and was purified by silica-gel column chromatography (5% IPA:EtOAc) to afford **41a** as a colourless oil (204 mg, 32%).

R_f = 0.45 (5% IPA:EtOAc)

^{1}H NMR (500 MHz, $CDCl_3$): δ = 7.71 (1H, dd, *J* = 8.4, 5.1 Hz, H^{12}), 7.24 (1H, dd, *J* = 8.5, 2.1 Hz, H^{15}), 7.05 (1H, td, *J* = 8.8, 2.2 Hz, H^{13}), 5.58 – 5.50 (1H, m, H^4), 5.44 – 5.36 (1H, m, H^3), 3.18 – 3.02 (3H, m, $H^{7,9}$), 2.51 – 2.41 (2H, m, H^6), 2.31 – 1.96 (10H, m, $H^{2,5,7,8}$), 0.96 (3H, t, *J* = 7.4 Hz, H^1).

^{19}F NMR (376 MHz, $CDCl_3$): δ = -109.45 – -109.85 (1F, m, F^{14}).

^{13}C {^{1}H} NMR (125 MHz, $CDCl_3$): δ = 164.2 (d, *J* = 250.2 Hz, C^{14}), 164.0 (d, *J* = 14.2 Hz, C^{16}), 161.2 (C^{10}), 133.7 (C^4), 126.7 (C^3), 122.8 (d, *J* = 11.2 Hz, C^{12}), 117.4 (C^{11}), 112.5 (d, *J* = 26.7 Hz, C^{13}), 97.6 (d, *J* = 26.6 Hz, C^{15}), 59.1 (C^6), 53.6 (C^7), 34.8 (C^9), 30.7 (C^8), 30.4 (C^5), 25.8 (C^2), 14.0 (C^1).

HRMS (APCI) calc: $[M+H]^+$ ($C_{18}H_{24}O_2NF$) 303.1867; measured: 303.1853 = 4.62 ppm difference.

IR (neat) ν_{max}/ cm^{-1}: 1613, 1495, 1313, 1273, 1152, 1122.

(Z)-4-(hex-3-en-1-yl)morpholine, 42a

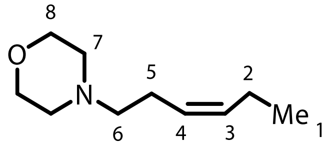

This substrate, **42a**, was synthesised from (Z)-3-hexen-1-ol (1 eq., 5 mmol, 0.60 mL) and morpholine (2 eq., 10 mmol, 0.87 mL) using **mesylation procedure 2** and was purified by silica-gel column chromatography (5% IPA:EtOAc) to afford **42a** as a pale yellow oil (426 mg, 51%).

R_f = 0.40 (5% IPA:EtOAc)

^{1}H NMR (500 MHz, $CDCl_3$): δ = 5.54 – 5.47 (1H, m, H^4), 5.41 – 5.34 (1H, m, H^3), 3.76 – 3.68 (4H, m, H^7), 2.52 – 2.42 (4H, m, H^8), 2.41 – 2.34 (2H, m, H^6), 2.22 – 2.15 (2H, m, H^5), 2.04 – 1.96 (2H, m, H^2), 0.96 (3H, t, *J* = 7.6 Hz, H^1).

^{13}C {^{1}H} NMR (125 MHz, $CDCl_3$): δ = 133.7 (C^4), 126.5 (C^3), 67.1 (C^7), 59.2 (C^6), 53.8 (C^8), 30.0 (C^5), 25.8 (C^2), 14.0 (C^1).

HRMS (ESI) calc: $[M+H]^+$ ($C_{11}H_{20}NO$) 170.1539; measured: 170.1542 = 1.76 ppm difference.

IR (neat) v_{max}/ cm^{-1}: 1453, 1394, 1379, 1280, 1252, 1247, 1151.

(Z)-1-(hex-3-en-1-yl)-4-(4-nitrophenyl)piperazine. 43a

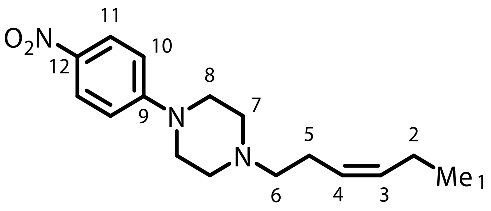

43a, was synthesised from (Z)-3-hexen-1-ol (1 eq., 5 mmol, 0.60 mL) and 1-(4-Nitrophenyl)piperazine (1 eq., 5 mmol, 1.4 g) using **mesylation procedure 2** and was purified by silica-gel column chromatography (5% IPA:EtOAc) to afford **43a** as a yellow oil (1.2 g, 83%).

R_f = 0.35 (5% IPA:EtOAc)

^{1}H NMR (500 MHz, $CDCl_3$): δ = 8.15 – 8.10 (2H, m, H^{11}), 6.85 – 6.79 (2H, m, H^{10}), 5.51 – 5.42 (1H, m, H^4), 5.38 – 5.30 (1H, m, H^3), 3.50 – 3.38 (4H, m, H^8), 2.66 – 2.55 (4H, m, H^7), 2.47 – 2.41 (2H, m, H^6), 2.34 – 2.24 (2H, m, H^5), 2.11 – 2.02 (2H, m, H^2), 0.98 (3H, t, J = 7.5 Hz, H^1).

^{13}C {^{1}H} NMR (125 MHz, $CDCl_3$): δ = 155.0 (C^{12}), 138.5 (C^9), 133.3 (C^4), 126.1 (C^{11}), 126.1 (C^3), 112.7 (C^{10}), 58.4 (C^8), 52.8 (C^7), 47.2 (C^6), 25.0 (C^5), 20.8 (C^2), 14.5 (C^1).

HRMS (APCI) calc: $[M+H]^+$ ($C_{16}H_{24}O_2N_3$) 290.1863; measured: 290.1850 = 4.48 ppm difference.

IR (neat) ν_{max}/ cm^{-1}: 1609, 1593, 1508, 1449, 1405, 1329, 1242, 1060.

(1R,5S)-8-((E)-hex-3-en-1-yl)-8-azabicyclo[3.2.1]octan-3-one, 44a

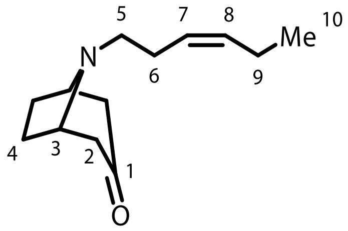

This substrate, **44a**, was synthesised from (Z)-3-hexen-1-ol (1 eq., 5 mmol, 0.60 mL) and nortropinone hydrochloride (1 eq., 5 mmol, 805 mg) using **mesylation procedure 2** and was purified by silica-gel column chromatography (5% IPA:EtOAc) to afford **44a** as a colourless oil (560 mg, 54%).

R_f = 0.35 (5% IPA:EtOAc)

^{1}H NMR (500 MHz, $CDCl_3$): δ = 5.59 – 5.51 (1H, m, H^7), 5.48 – 5.39 (1H, m, H^8), 3.62 – 3.52 (2H, m, H^3), 2.73 – 2.59 (4H, m, $H^{2,5}$), 2.31 – 2.22 (2H, m, H^6), 2.22 – 2.15 (2H, m, H^2), 2.07 – 1.97 (4H, m, $H^{4,9}$), 1.65 – 1.54 (2H, m, H^4), 0.97 (3H, t, *J* = 6.9 Hz, H^{10}).

^{13}C {^{1}H} NMR (125 MHz, $CDCl_3$): δ = 210.3 (C^1), 133.9 (C^7), 126.5 (C^8), 58.7 (C^5), 50.5 (C^3), 47.3 (C^2), 32.6 (C^6), 28.0 (C^4), 25.8 (C^9), 14.0 (C^{10}).

HRMS (APCI) calc: $[M+H]^+$ ($C_{13}H_{22}NO$) 208.1696; measured: 208.1693 = 1.44 ppm difference.

IR (neat) ν_{max}/ cm^{-1}: 1713, 1450, 1393, 1381 ,1237, 1056.

(*E*)-*N*,*N*-Dibenzylhex-3-en-1-amine, 26a-*trans*

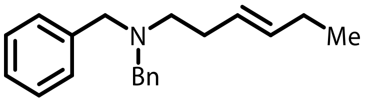

26a was synthesised from trans hex-3-en-1-ol (20 mmol) using **mesylation procedure 2** and was purified by silica-gel column chromatography (5% Et_2O:Hexane) to yield a colourless oil (650 mg, 50%).

R_f = 0.4 (5% Et_2O:Hexane)

^{1}H NMR (500 MHz, $CDCl_3$): δ = 7.41 – 7.19 (10H, m), 5.51 – 5.41 (1H, m), 5.39 – 5.29 (1H, m), 3.58 (4H, s), 2.47 – 2.25 (2H, t, *J* = 7.4 Hz), 2.25 – 2.18 (2H, m), 2.04 – 1.94 (2H, m), 0.96 (3H, t, *J* = 8.7 Hz).

^{13}C {^{1}H} NMR (125 MHz, $CDCl_3$): δ = 140.1, 133.1, 128.9, 128.2, 127.3, 126.9, 58.3, 53.6, 30.5, 25.8, 14.0.

These data are consistent with those previously reported.[5]

(E)-N,N-dibenzyloct-3-en-1-amine, 45a

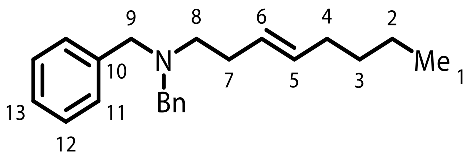

45a was synthesised from (E)-3-octen-1-ol (1 eq., 5 mmol, 0.75mL) and dibenzylamine (1 eq., 5 mmol, 985 mg) using **mesylation procedure 2** and was purified by silica-gel column chromatography (4% EtOAC:Hexane) to afford **45a** as a colourless oil (660 mg, 43%).

R_f = 0.25 (4% EtOAC:Hexane)

^{1}H NMR (500 MHz, $CDCl_3$): δ = 7.41 – 7.29 (8H, m, $H^{11,12}$), 7.25 – 7.20 (2H, m, H^{13}), 5.42 – 5.27 (2H, m, $H^{5.6}$), 3.60 (4H, m, H^9), 2.51 – 2.42 (2H, m, H^8), 2.31 – 2.19 (2H, m, H^7), 2.04 – 1.94 (2H, m, H^4), 1.34 – 1.23 (4H, m, $H^{2,3}$), 0.87 (3H, t, *J* = 6.8 Hz, C^1).

^{13}C {^{1}H} NMR (125 MHz, $CDCl_3$): δ = 140.0 (C^{10}), 131.0 (C^6), 128.9 (C^{11}), 128.3 (C^{12}), 127.4 (C^{13}), 126.9 (C^5), 58.3 (C^9), 53.4 (C^8), 32.0 (C^7), 27.2 (C^4), 25.1 (C^3), 22.5 (C^2), 14.1 (C^1).

HRMS (EI) calc: $[M]^+$ ($C_{22}H_{29}N$) 307.2300; measured: 307.2298 = 0.67 ppm difference.

IR (neat) v_{max}/ cm^{-1}: 1525, 1493, 1448, 1393, 1379, 1372, 1027, 1016.

(E)-N,N-dibenzylnon-3-en-1-amine, 46a

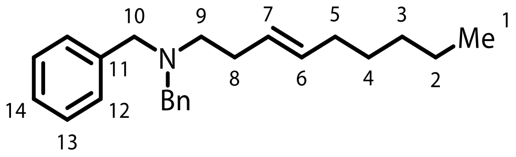

46a was synthesised from (E)-3-nonen-1-ol (1 eq., 5 mmol, 0.82mL) and dibenzylamine (1 eq., 5 mmol, 985 mg) using **mesylation procedure 2** and was purified by silica-gel column chromatography (4% EtOAC:Hexane) to afford **46a** a colourless oil (563 mg, 35%).

R_f = 0.25 (4% EtOAC:Hexane)

^{1}H NMR (500 MHz, CDCl$_3$): δ = 7.46 -7.41 (4H, m, H^{12}), 7.38 – 7.33 (4H, m, H^{13}), 7.26 – 7.25 (2H, m, H^{14}), 5.47 – 5.35 (2H, m, $H^{6.7}$), 3.65 (4H, s, H^{10}), 2.54 – 2.49 (2H, m, H^9), 2.35 – 2.28 (2H, m, H^8), 2.05 – 1.99 (2H, m, H^5), 1.40 – 1.25 (6H, m, $H^{2,3,4}$), 0.93 (3H, t, J = 6.7 Hz, H^1).

^{13}C {^{1}H} NMR (125 MHz, CDCl$_3$): δ = 140.0 (C^{11}), 131.0 (C^7), 128.9 (C^{12}), 128.3 (C^{13}), 127.4 (C^{14}), 126.9 (C^6), 58.3 (C^{10}), 53.3 (C^9), 31.6 (C^8), 29.5 (C^5), 27.4 (C^4), 25.1 (C^3), 22.7 (C^2), 14.2 (C^1).

HRMS (ESI) calc: $[M+H]^+$ ($C_{23}H_{32}N$) 322.2529; measured: 322.2533 = 1.30 ppm difference.

IR (neat) ν_{max}/ cm^{-1}: 1651, 1601, 1493, 1453, 1365, 1126, 1027.

(E)-N-(4-fluorobenzyl)-N-((E)-hex-3-en-1-yl)hex-3-en-1-amine, 47a

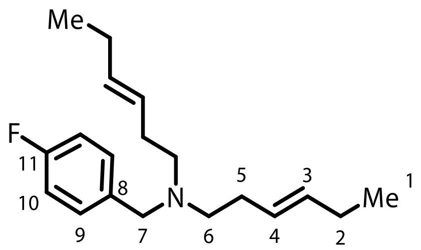

This substrate, **47a**, was synthesised from (E)-3-hexen-1-ol (2 eq., 20 mmol, 2.4 mL) and 4-fluorobenzylamine (1 eq., 10 mmol, 1.15 mL) using **mesylation procedure 2** and was purified by silica-gel column chromatography (10% EtOAc:Hexane) to afford **47a** as a colourless oil (491 mg, 17%).

R_f = 0.55 (10% EtOAc:Hexane)

^{1}H NMR (500 MHz, $CDCl_3$): δ = 7.34 – 7.28 (2H, m, H^{10}), 7.01 – 6.96 (2H, m, H^9), 5.44 – 5.25 (4H, m, $H^{3,4}$), 3.60 (2H, s, H^7), 2.53 – 2.46 (4H, m, H^6), 2.26 – 2.17 (4H, m, H^5), 2.06 – 1.97 (4H, m, H^2), 0.94 (6H, t, J = 7.5 Hz, H^1).

^{19}F NMR (376 MHz, $CDCl_3$): δ = -116.33 – -116.78 (1F, m, F^{11}).

^{13}C {^{1}H} NMR (125 MHz, $CDCl_3$): δ = 171.3 (C^8), 161.9 (d, J = 234.7 Hz, C^{11}), 133.0 (C^4), 130.5 (C^9), 130.4 (C^8), 126.6 (C^3), 115.1 (d, J = 20.2 Hz, C^{10}), 57.7 (C^7), 53.4 (C^6), 24.8 (C^4), 20.8 (C^2), 14.5 (C^1).

HRMS (APCI) calc: $[M+H]^+$ ($C_{19}H_{29}FN$) 290.2279; measured: 290.2267 = 4.14 ppm difference.

IR (neat) ν_{max}/ cm^{-1}: 1602, 1459, 1366, 1303, 1221, 1152, 1088, 1015.

Synthesis of products

Electrochemical synthesis of *p*-TolIF$_2$

To each compartment of PTFE divided cell equipped with a Nafion™ membrane and stirrer bars, CH_2Cl_2 (1.5 mL) and 5.6 HF:amine stock solution (4.5 mL) were added. To the anodic compartment, *p*-iodotoluene (2 eq., 1.2 mmol, 262 mg) was then added. Each compartment was then capped and wrapped in parafilm. A platinum electrode was inserted into each compartment, and the reaction was subjected to electrolysis (17 mA, 2.2 *F*, 4.2 h). The electrodes were removed and the anodic compartment mixture was used for each chlorofluorination procedure below, as well as mechanistic investigations (*vide infra*).

Chlorofluorination procedure 1 (for terminal alkenes)

To a 50 mL HDPE vial was added *p*-TolIF$_2$ (1.0 eq., 0.6 mmol, 3 mL of anodic compartment reaction mixture, withdrawn directly from divided cell via syringe), and CH_2Cl_2 (3 mL). This vial was capped with a Suba-seal, a venting needle placed through the Suba-seal, and the vial then cooled to −46 °C. Alkene substrate (0.6 м, 1.0 eq., 0.6 mmol) in CH_2Cl_2 (1 mL) was added and then tetraethylammonium chloride (TEAC) (0.2 м, 1.0 eq., 0.6 mmol, 100 mg) in CH_2Cl_2 (3 mL) was added *via* syringe pump (0.16 eq./min; 0.48 mL/min). After stirring overnight, the reaction mixture was quenched with 300 mL of cold (0 °C) saturated aqueous $NaHCO_3$ solution until the aqueous layer measured pH 7, and then the reaction mixture was stirred for 1 hour. The mixture was extracted into CH_2Cl_2, dried with Na_2SO_4, filtered, and concentrated *in vacuo*. The product was then purified *via* silica-gel column chromatography.

Anti-chlorofluorination procedure 2 (Anti: internal alkenes)

To a 50 mL HDPE vial was added *p*-TolIF$_2$ (1.0 eq., 0.6 mmol, 3 mL of anodic compartment reaction mixture, withdrawn directly from divided cell via syringe), and CH_2Cl_2 (3 mL). This vial was capped with a Suba-seal and a venting needle was placed through the Suba-seal, and then cooled to −46 °C. Alkene substrate (0.6 м, 1.0 eq., 0.6 mmol) in CH_2Cl_2 (1 mL) was added and then tetraethylammonium chloride (TEAC) (0.2 м, 1.0 eq., 0.6 mmol, 100 mg) in CH_2Cl_2 (3 mL) was added *via* syringe pump (0.16 eq./h; 0.48 mL/h). After stirring overnight, the reaction mixture was quenched with 300 mL of cold (0 °C) saturated aqueous $NaHCO_3$ solution until the aqueous layer

measured pH 7, and then the reaction mixture was stirred for 1 hour. The mixture was extracted into CH_2Cl_2, dried with Na_2SO_4, filtered, and concentrated *in vacuo*. The product was then purified *via* silica-gel column chromatography.

Syn-chlorofluorination procedure 3 (Syn: internal alkenes)

To a 50 mL HDPE vial was added *p*-TolIF$_2$ (1.0 eq., 0.6 mmol, 3 mL of anodic compartment reaction mixture, withdrawn directly from divided cell via syringe), CH_2Cl_2 (3 mL) and pyridinium poly(hydrogenfluoride) (1.5 mL unless specified otherwise). This vial was capped with a Suba-seal and a venting needle was placed through the Suba-seal, and then cooled to −46 ˚C. Alkene substrate (0.6 ᴍ, 1.0 eq., 0.6 mmol) in CH_2Cl_2 (1 mL) was added and then tetraethylammonium chloride (TEAC) (0.2 ᴍ, 1.0 eq., 0.6 mmol, 100 mg) in CH_2Cl_2 (3 mL) was added *via* syringe pump (0.16 eq./ h; 0.48 mL/h). After stirring overnight, the reaction mixture was quenched with 400 mL of cold (0 °C) saturated aqueous $NaHCO_3$ solution until the aqueous layer measured pH 7, and then the reaction mixture was stirred for 1 hour. The mixture was extracted into CH_2Cl_2, dried with Na_2SO_4, filtered, and concentrated *in vacuo*. The product was then purified *via* silica-gel column chromatography.

Anti-chlorofluorination procedure 4 (Anti: PIFA instead of electrochemically generated tol-IF$_2$)

To a 50 mL HDPE vial was added bis(trifluoroacetoxy)iodobenzene (**PIFA**) (1 eq., 0.2 mmol, 86 mg), 5.6HF:amine stock solution (0.75 mL) and CH_2Cl_2 (0.25 mL) (unless otherwise stated). This vial was capped with a Suba-seal, a venting needle placed through the Suba-seal, and the vial then cooled to −46 °C. Alkene substrate (0.6 ᴍ, 1.0 eq., 0.2 mmol) in CH_2Cl_2 (0.33 mL) was added and then tetraethylammonium chloride (TEAC) (0.2 ᴍ, 1.0 eq., 0.2 mmol, 33 mg) in CH_2Cl_2 (1 mL) was added *via* syringe pump (0.16 eq./min; 0.16 mL/min). After stirring overnight, the reaction mixture was quenched with 300 mL of cold (0 °C) saturated aqueous $NaHCO_3$ solution until the aqueous layer measured pH 7, and then the reaction mixture was stirred for 1 hour. The mixture was extracted into CH_2Cl_2, dried with Na_2SO_4, filtered, and concentrated *in vacuo*. The product was then purified *via* silica-gel column chromatography. The residue was dissolved in $CDCl_3$ (2 mL), to this solution was added hexafluorobenzene (23.2 μL, 1.0 eq.) and a ^{19}F NMR spectrum of the sample was recorded to measure a ^{19}F NMR yield.

Syn-chlorofluorination procedure 5 (Syn: PIFA instead of electrochemically generated $TolIF_2$)

To a 50 mL HDPE vial was added bis(trifluoroacetoxy)iodobenzene (**PIFA**) (1 eq., 0.2 mmol, 86 mg), 5.6HF:amine stock solution (0.75 mL) and CH_2Cl_2 (0.25 mL) (unless otherwise stated) and pyridinium poly(hydrogenfluoride) (0.5 mL unless specified otherwise). This vial was capped with a Suba-seal and a venting needle was placed through the Suba-seal, and then cooled to −46 ˚C. Alkene substrate (0.6 м, 1.0 eq., 0.6 mmol) in CH_2Cl_2 (1 mL) was added and then tetraethylammonium chloride (TEAC) (0.2 м, 1.0 eq., 0.6 mmol, 100 mg) in CH_2Cl_2 (3 mL) was added *via* syringe pump (0.16 eq./ h; 0.48 mL/h). After stirring overnight, the reaction mixture was quenched with 400 mL of cold (0 °C) saturated aqueous $NaHCO_3$ solution until the aqueous layer measured pH 7, and then the reaction mixture was stirred for 1 hour. The mixture was extracted into CH_2Cl_2, dried with Na_2SO_4, filtered, and concentrated *in vacuo*. The residue was dissolved in $CDCl_3$ (2 mL), to this solution was added hexafluorobenzene (23.2 μL, 1.0 eq.) and a ^{19}F NMR spectrum of the sample was recorded to measure a ^{19}F NMR yield.

Characterisation of products

Terminal chlorofluorination products

N-(5-chloro-4-fluoropentyl)-4-methylbenzenesulfonamide, 2j

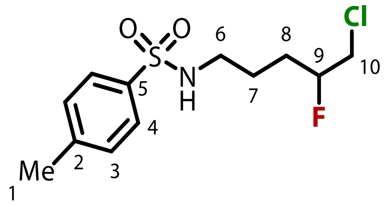

Product **2j** was synthesised from **2a** using **chlorofluorination procedure 1** and purified using silica-gel chromatography (20% EtOAc:Hexane) to yield **2j** as a viscous colourless oil (141 mg, 80%, rr = >19:1).

R_f = 0.2 (30% EtOAc:Hexane)

^{1}H NMR (500 MHz, $CDCl_3$): δ = 7.74 (2H, d, *J* = 7.74 Hz, *H^4*), 7.31 (2H, d, *J* = 7.31 Hz, *H^3*), 4.74 (1H, t, *J* = 6.1 Hz, *NH*), 4.66 – 4.51 (1H, m, *H^9*), 3.59 – 3.53 (2H, m, *H^{10}*), 3.04 – 2.93 (2H, m, *H^6*), 2.43 (3H, s, *H^1*), 1.78 – 1.55 (4H, m, *$H^{7,\ 8}$*).

^{19}F NMR (376 MHz, $CDCl_3$): δ = -182.39 – -182.79 (1F, m, *F^9*).

^{13}C {^{1}H} NMR (125 MHz, $CDCl_3$): δ = 143.7 (*C^5*), 136.9 (*C^2*), 129.9 (*C^3*), 127.2 (*C^4*), 91.9 (d, *J* = 175.2 Hz, *C^9*), 45.7 (d, *J* = 25.1 Hz, *C^{10}*), 42.8 (*C^6*), 29.5 (d, *J* = 21.0 Hz, *C^8*), 25.2 (d, *J* = 3.5 Hz, *C^7*), 21.7 (*C^1*).

HRMS (APCI) calc: $[M+H]^+$ ($C_{12}H_{18}O_2{}^{35}ClFSN$) 294.0725; measured: 294.0714 = 3.74 ppm difference.

IR (neat) ν_{max}/ cm^{-1}: 3276, 1596, 1476, 1426, 1316, 1153, 1062, 1027.

N-(3-chloro-2-fluoropropyl)-N-(4-methylisoxazol-3-yl)-4-nitrobenzenesulfonamide, 2j

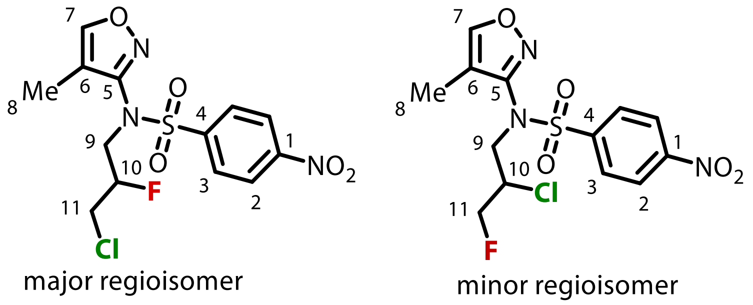

Product **4j** was synthesised from **4a** using **chlorofluorination procedure 1**. The product was purified using silica-gel chromatography (35% EtOAc:Hexane) to afford **4j** as a colourless oil (174 mg, 77%, rr = 5:1)

R_f = 0.15 (35% EtOAc:Hexane)

^{1}H NMR (500 MHz, $CDCl_3$): Major regioisomer δ = 8.35 (2H, d, *J* = 8.5 Hz, H^2), 7.98 (2H, d, *J* = 8.6 Hz, H^3), 6.40 (1H, q, *J* = 0.9 Hz, H^7), 5.11 – 4.91 (1H, m, H^{10}), 4.27 (1H, ddd, *J* = 14.9, 13.9, 7.5 Hz, H^9), 4.00 (1H, ddd, *J* = 26.0, 14.9, 3.6 Hz, H^9), 3.81 – 3.62 (2H, m, H^{11}), 2.42 (3H, d, *J* = 0.9 Hz, H^8). Minor regioisomer δ = 8.35 (2H, d, *J* = 8.5 Hz, H^2), 7.94 (2H, d, *J* = 8.6 Hz, H^3), 6.42 (1H, q, *J* = 0.9 Hz, H^7), 4.73 – 4.50 (2H, m, H^{11}), 4.48 – 4.35 (1H, m, H^{10}), 4.18 – 4.06 (2H, m, H^9), 2.43 (3H, q, *J* = 0.9 Hz, H^8).

^{19}F NMR (376 MHz, $CDCl_3$): Major regioisomer δ = -186.02 – -186.47 (1F, m, F^{10}). Minor regioisomer δ = -223.42 (td, *J* = 46.7, 19.3 Hz, F^{11}).

^{13}C {^{1}H} NMR (125 MHz, $CDCl_3$): Major regioisomer δ = 171.8 (C^5), 159.4 (C^7), 150.4 (C^1), 143.4 (C^4), 128.8 (C^2), 124.7 (C^3), 98.3 (C^7), 89.0 (d, *J* = 180.5 Hz, C^{10}), 50.6 (d, *J* = 24.8 Hz, C^{10}), 43.6 (d, *J* = 22.5 Hz, C^{11}), 12.9 (C^8). Minor regioisomer δ = 171.9 (C^5), 159.2 (C^7), 150.8 (C^1), 143.0 (C^4), 128.7 (C^2), 124.8 (C^3), 98.7 (C^7), 83.2 (d, *J* = 178.2 Hz, C^{11}), 55.0 (d, *J* = 20.4 Hz, C^{10}), 51.1 (d, *J* = 4.9 Hz, C^9), 12.9 (C^8).

HRMS (ESI) calc: $[M+Na]^+$ ($C_{13}H_{13}{}^{35}ClFN_3NaO_5S$) 400.0141; measured: 400.0151 = 2.50 ppm difference.

IR (neat) ν_{max}/ cm^{-1}: 1602, 1533, 1477, 1448, 1351, 1313, 1177, 1088, 1066, 1011.

N-(3-chloro-2-fluoropropyl)-N-(4-fluoro-2-methylphenyl)-4-nitrobenzenesulfonamide, 3j

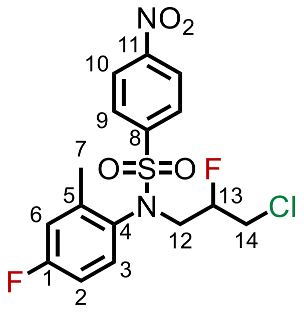

Product **3j** was synthesised from **3a** using **chlorofluorination procedure 1** on a 1 mmol scale. The crude product was then purified via silica gel flash column chromatography (2:8 EtOAc:Hexane) to afford **3j** as a colourless oil that solidified to form an off-white solid on standing (0.233 g, 55%, rr >19:1). NMR data suggest two inseparable diastereomers resulting from a rotameric centre in solution.

R_f = 0.2 (2:8 EtOAc:Hexane)

^{1}H NMR (400 MHz, $CDCl_3$): Major diastereomer δ = 8.34 (2H, d, J = 8.8 Hz, H^{10}), 7.87 (2H, d, J = 8.8 Hz, H^9), 7.07 – 6.97 (1H, m, H^6), 6.84 – 6.76 (1H, m, H^2), 6.56 (1H, dd, J = 8.7, 5.2 Hz, H^3), 4.89 – 4.72 (1H, m, H^{13}), 4.21 – 3.87 (1H, m, H^{14}), 3.86 – 3.56 (3H, m, $H^{12,14}$), 2.38 (3H, s, H^7). Minor diastereomer δ = 8.35 (2H, d, J = 8.8 Hz, H^{10}), 7.85 (2H, d, J = 8.7 Hz, H^9), 7.07 – 6.97 (1H, m, H^6), 6.84 – 6.76 (1H, m, H^2), 6.65 (1H, dd, J = 8.8, 5.3 Hz, H^3), 4.73 – 4.59 (1H, m, H^{13}), 4.21 – 3.87 (1H, m, H^{14}), 3.86 – 3.56 (3H, m, $H^{12,14}$), 2.28 (3H, s, H^7).

^{13}C NMR (101 MHz, $CDCl_3$): Major diastereomer δ = 162.5 (1C, d, J = 250.4 Hz, C^1), 150.4 (1C, C^{11}), 144.4 (1C, C^8), 142.6 (1C, d, J = 8.7 Hz, C^5), 133.3 (1C, C^4), 130.4 (1C, d, J = 9.3 Hz, C^3), 129.4 (2C, C^9), 124.3 (2C, C^{10}), 118.9 (1C, d, J = 22.6 Hz, C^6), 114.0 (1C, d, J = 22.8 Hz, C^2), 89.9 (1C, d, J = 181.3 Hz, C^{13}), 53.6 (1C, d, J = 23.0 Hz, C^{14}), 43.2 (1C, d, J = 24.2 Hz, C^{12}), 18.6 (1C, C^7). Minor diastereomer δ = 162.4 (1C, d, J = 250.0 Hz, C^1), 150.5 (1C, C^{11}), 144.0 (1C, C^8), 142.4 (1C, d, J = 8.7 Hz, C^5), 133.3 (1C, d, J = 2.9 Hz, C^4), 130.3 (1C, d, J = 9.1 Hz, C^3), 129.3 (2C, C^9), 124.5 (2C, C^{10}), 118.7 (1C, d, J = 23.3 Hz, C^6), 114.1 (1C, d, J = 22.8 Hz, C^2), 89.2 (1C, d, J = 182.4 Hz, C^{13}), 53.7 (1C, d, J = 22.8 Hz, C^{14}), 43.1 (1C, d, J = 24.9 Hz, C^{12}), 18.6 (1C, C^7).

^{19}F NMR (377 MHz, $CDCl_3$): Major diastereomer δ = -111.10 (1F, ddd, *J* = 9.2, 7.6, 5.4 Hz), -184.66 – -185.59 (1F, m). Minor diastereomer δ = -111.20 (1F, ddd, *J* = 9.2, 7.6, 5.4 Hz), -184.66 – -185.59 (1F, m).

IR (neat) V_{max} / cm^{-1} = 1529, 1495, 1349, 1313, 1166, 1089, 856, 737, 688, 808.

HRMS (APCI) calc: $[M+H]^+$ ($C_{16}H_{15}O_4N_2{}^{35}ClF_2S$) 405.0482; measured: 405.0475 = 1.73 ppm difference.

4-(3-chloro-2-fluoropropyl)-N,N-dimethylaniline 5j

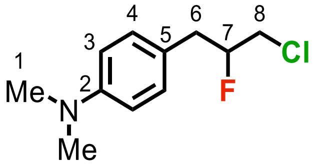

Product **5j** was synthesised from 4-allyl-*N,N*-dimethylaniline using **chlorofluorination procedure 1** on a 1 mmol scale. The crude product was then purified via silica gel flash column chromatography (1:9 EtOAc:Hexane) to afford **5j** as a light orange oil (0.106 g, 49%, rr >19:1).

R_f = 0.3 (1:9 EtOAc:Hexane).

^{1}H NMR (400 MHz, $CDCl_3$): δ = 7.10 (2H, d, *J* = 9.4 Hz, H^3), 6.70 (2H, d, *J* = 8.7 Hz, H^2), 4.80 (1H, app. dtdd, *J* = 47.1, 6.3, 5.4, 4.3 Hz, H^7), 3.68 – 3.51 (2H, m, H^8), 2.99 (2H, dd, *J* = 18.7, 6.4 Hz, H^6), 2.93 (3H, s, H^1)

^{13}C NMR (101 MHz, $CDCl_3$): δ = 149.9 (1C, C^2), 130.2 (2C, C^3), 123.2 (1C, d, *J* = 6.6 Hz, C^5), 113.0 (2C, C^4), 92.9 (1C, d, *J* = 178.2 Hz, C^7), 45.1 (1C, d, *J* = 24.9 Hz, C^8), 40.8 (2C, C^1), 37.6 (1C, d, *J* = 21.1 Hz, C^6).

^{19}F NMR (377 MHz, $CDCl_3$): δ = -179.36 (1F, dp, *J* = 47.1, 19.9 Hz).

IR (neat) v_{max}/ cm^{-1}: 2916 (br), 1615, 1522, 1348, 1227, 1165, 1031, 947, 807

HRMS (EI) calc: $[M]^{+\cdot}$ ($C_{11}H_{15}N^{35}ClF$) 215.0872; measured: 215.0870 = 0.93 ppm difference.

1-(2-chloro-3-fluoropropyl)-4-fluorobenzene, 6j

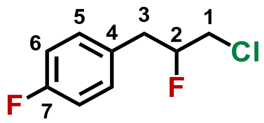

Product **6j** was synthesised from 1-allyl-4-fluorobenzene using **chlorofluorination procedure 1** on a 1 mmol scale. The crude product was then purified via silica gel flash column chromatography (1:19 EtOAc:Hexane) to afford **6j** as a colourless oil (0.145 g, 77%, rr >19:1).

R_f = 0.3 (1:19 EtOAc:Hexane).

^{1}H NMR (400 MHz, $CDCl_3$): δ = 7.24 – 7.19 (2H, m, H^6), 7.01 (2H, t, J = 8.7 Hz, H^5), 4.82 (1H, app. dddt, J = 46.8, 6.4, 5.6, 5.0 Hz, H^2), 3.67 – 3.51 (2H, m, H^1), 3.10 – 3.01 (2H, m, H^3).

^{13}C NMR (101 MHz, $CDCl_3$): δ = 162.2 (1C, d, J = 245.2 Hz, C^7), 131.3 (dd, J = 5.1, 3.2 Hz, C^4), 131.1 (2C d, J = 7.7 Hz, C^5), 115.7 (2C, d, J = 21.2 Hz, C^6), 92.3 (1C, d, J = 178.2 Hz, C^2), 44.7 (1C, d, J = 26.0 Hz, C^1), 37.7 (1C, d, J = 21.2 Hz, C^3).

^{19}F NMR (376 MHz, $CDCl_3$): δ = -115.78 (1F, tt, J = 8.9, 5.2 Hz), -179.86 – -180.25 (1F, m).

IR (neat) v_{max}/ cm^{-1}: 3357 (br), 2976, 2936, 1718, 1659, 1432, 1278, 1218, 838.

HRMS (EI) calc: $[M]^{+\cdot}$ ($C_9H_9{}^{35}ClF_2$) 190.0354; measured: 190.0355 = 0.53 ppm difference.

1-(3-chloro-2-fluoropropyl)piperidine, 7j

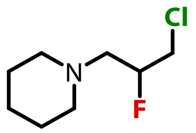

Product **7j** was synthesised from *N*-allyl piperidine using **chlorofluorination procedure 1** and purified using silica-gel chromatography (10% IPA:EtOAc) to afford **7j** as a viscous yellow oil (40 mg, 37%, rr >19:1).

R_f = 0.15 (10% EtOAc:IPA)

^{1}H NMR (500 MHz, $CDCl_3$): 4.92 – 4.75 (1H, m), 3.81 – 3.74 (1H, m), 3.70 – 3.65 (1H, m), 2.70 – 2.61 (2H, m), 2.57 – 2.46 (4H, m), 1.65 – 1. 51 (4H, m), 1.49 – 1.41 (2H, m).

^{19}F NMR (376 MHz, $CDCl_3$): -182.87 – -182.95 (1F, m).

^{13}C {^{1}H} NMR (125 MHz, $CDCl_3$): 90.7 (d, *J* = 177.8 Hz), 60.5 (d, *J* = 21.5 Hz), 55.3, 44.8 (d, *J* = 24.1 Hz), 26.0, 24.1.

These data are consistent with those previously reported.[9]

11-chloro-10-fluoroundecan-1-ol, 8j

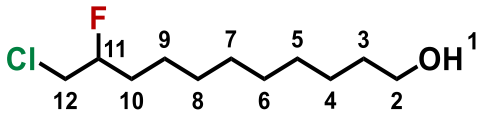

Product **8j** was synthesised from 10-undecen-1-ol using **chlorofluorination procedure 1** on a 1 mmol scale. The crude product was then purified via silica gel flash column chromatography (2:8 EtOAc:Hexane) to afford **8j** as a white solid (0.150 g, 67%, rr >19:1).

R_f = 0.2 (2:8 EtOAc:Hexane)

^{1}H NMR (400 MHz, $CDCl_3$): δ = 4.64 (1H, app. ddt, *J* = 48.0, 12.9, 4.9 Hz, H^{11}), 3.69 – 3.56 (4H, m, $H^{2, 12}$), 1.79 – 1.61 (2H, m), 1.61 – 1.52 (2H, m), 1.31 (12H, br s)

^{19}F NMR (376 MHz, $CDCl_3$): δ = -181.73 (1F, dddd, *J* = 48.2, 37.6, 28.6, 18.7 Hz)

IR (neat) $ν_{max}$/ cm^{-1}: 3421 (br), 2925, 2851, 1466, 1354, 1058, 836, 739.

MS (ACPI) m/z: 207 (31%, M - OH), 205 (100), 169 (40) 151 (92), 138 (50), 95 (96).

2-(11-chloro-10-fluoroundecyl)isoindoline-1,3-dione, 9j

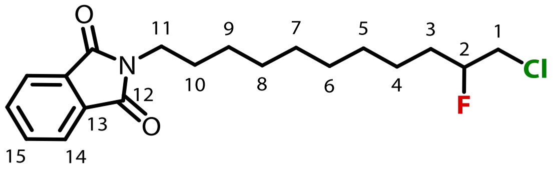

Product **9j** was synthesised using **chlorofluorination procedure 1** and purified using silica-gel chromatography (201 mg, 95%, rr >19:1).

^{1}H NMR (500 MHz, CDCl$_3$): δ = 7.82 (2H, dd, *J* = 5.4, 3.1 Hz, H^{15}), 7.69 (2H, dd, *J* = 5.5, 3.0 Hz, H^{14}), 4.61 (1H, dddd, *J* = 48.0, 9.8, 8.3, 4.8 Hz, H^{2}), 3.65 (3H, t, *J* = 7.3 Hz, H^{11}), 3.59 (2H, ddd, *J* = 19.9, 5.0, 3.2 Hz, H^{1}), 1.80 – 1.56 (4H, m, $H^{3\text{-}10}$), 1.38 – 1.19 (12H, m, $H^{3\text{-}10}$).

^{13}C NMR (151 MHz, CDCl$_3$): δ = 168.5 (C^{12}), 133.9 (C^{14}), 132.2 (C^{13}), 123.2 (C^{15}), 92.5 (d, *J* = 175.7 Hz, C^{2}), 45.9 (d, *J* = 25.2 Hz, C^{1}), 38.1 (C^{11}), 32.5 (d, *J* = 25.2 Hz, C^{3}), 29.4 (d, *J* = 1.4 Hz, C^{4}), 29.3 ($C^{5\text{-}10}$), 29.2 ($C^{5\text{-}10}$), 28.6 ($C^{5\text{-}10}$), 26.9 ($C^{5\text{-}10}$), 24.7 ($C^{5\text{-}10}$), 24.7 ($C^{5\text{-}10}$).

^{19}F NMR (376 MHz, CDCl$_3$): δ = -181.52 – -181.98 (1F, m, F^{2}).

HRMS (ESI) calc: $[M]^+$ ($C_9H_{25}{}^{35}ClFNO_2$) 354.1631; measured: 354.1618 = 3.67 ppm difference.

IR (neat) ν_{max}/ cm^{-1}: 2922, 2850, 1721, 1701, 1615, 1397, 1053, 718, 710, 530.

1-(2-chloro-1-fluoroethyl)-3-nitrobenzene, 10j

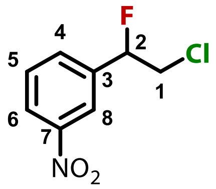

Product **10j** was synthesised from 3-nitrostyrene using **chlorofluorination procedure 1** and purified using silica-gel chromatography (201 mg, 95%, rr >19:1).

^{1}H NMR (400 MHz, CDCl$_3$): 8.31 – 8.24 (2H, m, *H^{6,8}*), 7.77 – 7.69 (1H, m, *H^{5}*), 7.66 – 7.59 (1H, m, *H^{4}*), 5.83 – 5.64 (1H, m, *H^{2}*), 3.69 – 3.78 (2H, m, *H^{1}*).

^{19}F NMR (371 MHz, CDCl$_3$): -179.95 (1F, dt, *J* = 46.1, 19.5 Hz, *F^{2}*).

^{13}C NMR (151 MHz, CDCl$_3$): 148.5 (*C^{7}*), 138.8 (d, *J* = 20.2 Hz, *C^{3}*), 131.9 (d, *J* = 7.5 Hz, *C^{8}*), 130.0 (*C^{6}*), 124.2 (*C^{5}*), 121.1 (d, *J* = 7.1 Hz, *C^{4}*), 91.6 (d, *J* = 181.65 Hz, *C^{2}*), 46.2 (d, *J* = 27.7 Hz, *C^{1}*).

HRMS (EI) calc: $[M]^+$ ($C_8H_7{}^{35}ClFNO_2$) 203.0144; measured: 203.0143 = 0.49 ppm difference.

IR (neat) ν_{max}/ cm^{-1}: 1530, 1406, 1393, 1249, 1057, 1066, 1027

(S)-((1S,2S,4S,5R)-5-((R)-1-chloro-2-fluoroethyl)quinuclidin-2-yl)(quinolin-4-yl)methyl acetate, 11k

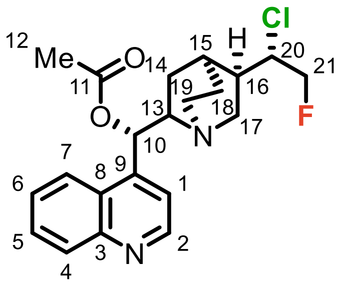

Product **11k** was synthesised from **11a** using ***anti*-chlorofluorination procedure 2** and purified using silica-gel chromatography (95% EtOAc:Hexane) to afford **11k** as a viscous colourless oil (141 mg, 60%, rr >19:1). Diastereomer assignment of C20 in relation to the neighbouring C16 stereocentre has been estimated from consideration of the Newman projections of either diastereomer and a $^3J_{H16\text{-}H20}$ = 10.7 Hz coupling constant, which indicates a φ(HCCH) = ~150°.

R$_f$ = 0.1 (95% EtOAc:Hexane)

^{1}H NMR (500 MHz, CDCl$_3$): δ = 8.87 (1H, d, *J* = 4.5 Hz, H^2), 8.19 – 8.09 (2H, m, $H^{4,7}$), 7.72 (1H, ddd, *J* = 8.4, 6.9, 1.3 Hz, H^5), 7.61 (1H, ddd, *J* = 8.4, 6.9, 1.4 Hz, H^6), 7.36 (1H, d, *J* = 4.5 Hz, H^1), 6.62 (1H, d, *J* = 6.2 Hz, H^{10}), 4.71 – 4.55 (2H, m, H^{21}), 4.35 (1H, ddt, *J* = 19.5, 10.7, 4.5 Hz, H^{20}), 3.32 – 3.22 (1H, m, H^{13}), 3.06 (1H, ddd, *J* = 14.3, 7.7, 2.3 Hz, H^{19}), 2.98 (1H, ddd, *J* = 14.3, 9.7, 1.3 Hz, H^{19}), 2.80 (1H, dddd, *J* = 13.3, 8.7, 4.3, 2.4 Hz, H^{17}), 2.76 – 2.66 (1H, m, H^{17}), 2.17 (3H, s, H^{12}), 2.02 – 1.93 (2H, m, $H^{15,16}$), 1.80 – 1.72 (1H, m, H^{18}), 1.57 – 1.42 (3H, m, $H^{14,18}$).

^{19}F NMR (376 MHz, CDCl$_3$): δ = -220.28 (1F, td, *J* = 47.1, 19.4, F^{21}).

^{13}C {^{1}H} NMR (125 MHz, CDCl$_3$): δ = 169.9, 150.0 (C^2), 148.6, 145.1, 130.6 (C^4), 129.4 (C^5), 127.1 (C^6), 125.8 (*C*), 123.3 (C^7), 118.2(C^1), 84.9 (d, *J* = 175.8 Hz, C^{21}), 74.1 (C^{10}), 62.4 (d, *J* = 19.6 Hz, C^{20}), 59.1 (C^{13}), 49.9 (C^{17}), 48.9 (C^{19}), 39.4 (C^{16}), 26.7 (C^{14}), 25.3 (C^{16}), 22.9 (C^{18}), 21.3 (C^{12}).

HRMS (ESI) calc: $[M+H]^+$ ($C_{21}H_{25}N_2O_2{}^{35}ClF$) 391.1586; measured: 391.1586 = 0.76 ppm difference.

IR (neat) ν_{max}/ cm^{-1}: 1591, 1568, 1460, 1329, 1280 ,1050.

1-(2-(3-chloro-2-fluoropropoxy)-2-(2,4-dichlorophenyl)ethyl)-1H-imidazole, 12j

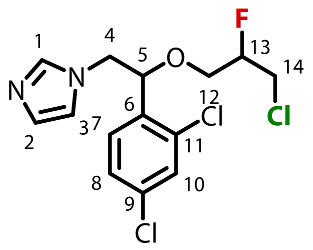

Product **12j** was synthesised from enilconazole using **chlorofluorination procedure 1** and purified using silica-gel chromatography (5% IPA:EtOAc) to afford **12j** as a colourless oil (158 mg, 87%, rr = 11:1).

R_f = 0.45 (5% IPA:EtOAc)

^{1}H NMR (500 MHz, $CDCl_3$): δ = 7.52 – 7.42 (2H, m, $H^{1,10}$), 7.34 – 7.24 (2H, m, $H^{2,3}$), 7.05 (1H, s, H^7), 6.94 (1H, s, H^8), 4.50 – 4.95 (1H, m, H^{14}), 4.82 – 4.63 (1H, m, H^{13}), 4.28 – 4.20 (1H, m, H^4), 4.10 – 4.02 (1H, m, H^4), 3.72 – 3.50 (4H, m, $H^{5,12,14}$).

^{19}F NMR (376 MHz, $CDCl_3$): δ = -186.75 – -187.75 (1F, m, F^{13}).

^{13}C {^{1}H} NMR (125 MHz, $CDCl_3$): δ = 137.9 (C^1), 135.3 (C^6), 133.4 (C^{11}), 133.3 (C^9), 129.8 (C^7), 129.3 (C^{10}), 128.4 (C^8), 128.2 (C^2), 119.9 (C^3), 90.4 (d, J = 180.1 Hz, C^{13}), 78.7 (C^4), 68.7 (d, J = 22.4 Hz, C^{14}), 51.4 (C^5), 41.9 (d, J = 26.7 Hz, C^{12}).

HRMS (APCI) calc: $[M+H]^+$ ($C_{14}H_{15}{}^{35}Cl_3FN_2O$) 351.0229; measured: 351.0226 = 0.85 ppm difference.

IR (neat) ν_{max}/ cm^{-1}: 1588, 1561, 1504, 1469, 1435, 1381, 1285, 1263, 1106, 1032.

Benzyl 2-(3-chloro-4-(3-chloro-2-fluoropropoxy)phenyl)acetate, 13j

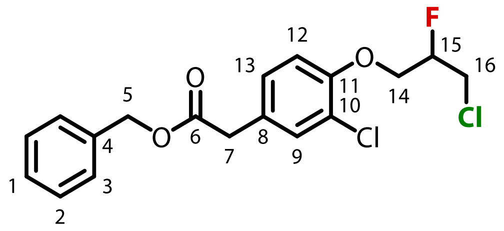

Product **13j** was synthesised from **13a** using **chlorofluorination procedure 1** and purified using silica-gel chromatography (30% EtOAc:Hexane) to afford **13j** as a colourless oil (133 mg, 60%, rr = 14:1).

R_f = 0.25 (30% EtOAc:Hexane)

^{1}H NMR (400 MHz, $CDCl_3$): δ = 7.43 – 7.28 (6H, m, $H^{1-3,9}$), 7.17 – 7.08 (1H, m, H^{13}), 6.89 (1H, d, *J* = 8.4 Hz, H^{12}), 5.12 (2H, s, H^{7}), 5.02 (1H, dddd, *J* = 46.1, 9.6, 5.2, 4.5 Hz, H^{15}), 4.35 – 4.24 (2H, m, H^{14}), 4.05 – 3.80 (2H, m, H^{16}), 3.59 (2H, s, H^{5}).

^{19}F NMR (376 MHz, $CDCl_3$): δ = -188.80 (1F, dtt, *J* = 46.0, 19.5, 17.3 Hz, F^{15}).

^{13}C {^{1}H} NMR (100 MHz, $CDCl_3$): δ = 171.2 (C^{6}), 153.0 (C^{11}), 135.8 (C^{4}), 131.4 (C^{9}), 128.8 (C^{3}), 128.7 (C^{13}), 128.5 (C^{8}), 128.4(C^{1}), 128.3 (C^{10}), 123.5 (C^{2}), 114.1 (C^{12}), 89.8 (d, *J* = 178.7 Hz, C^{15}), 67.8 (d, *J* = 27.2 Hz, C^{14}), 67.0 (C^{7}), 42.4 (d, *J* = 25.8 Hz, C^{16}), 40.2 (C^{5}).

HRMS (EI) calc: $[M]^+$ ($C_{18}H_{17}{}^{35}Cl_2FO_3$) 370.0533; measured: 370.0535 = 0.54 ppm difference.

IR (neat) ν_{max}/ cm^{-1}: 1733, 1608, 1500, 1453, 1409, 1393, 1379, 1257, 1131, 1068.

(4S)-4-(1-chloro-2-fluoropropan-2-yl)-2-methylcyclohex-2-en-1-one, 14j

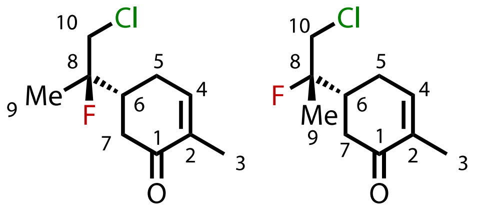

1st diasteromer 2nd diasteromer

Product **14j** was synthesised from L-carvone using ***anti*-chlorofluorination procedure 2** and purified using silica-gel chromatography (10% EtOAc:Hexane) to afford **14j** as a colourless oil (149 mg, 85%, rr >19:1). 1:1 mixture of diastereomers.

R_f = 0.25 (10% EtOAc:Hexane)

^{1}H NMR (500 MHz, $CDCl_3$): First diastereomer δ = 6.79 – 6.72 (1H, m, *H^4*), 3.68 – 3.53 (2H, m, *H^{10}*), 2.66 – 2.21 (5H, m, *$H^{5,6,7}$*), 1.80 – 1.77 (3H, m, *H^3*), 1.44 (3H, d, *J* = 21.7 Hz, *H^9*). Second diastereomer δ = 6.79 – 6.72 (1H, m, *H^4*), 3.68 – 3.53 (2H, m, *H^{10}*), 2.66 – 2.21 (5H, m, *$H^{5,6,7}$*), 1.80 – 1.77 (3H, m, *H^3*), 1.43 (3H, d, *J* = 21.7 Hz, *H^9*).

^{19}F NMR (376 MHz, $CDCl_3$): First diastereomer δ = -155.18 (qdt, *J* = 21.7, 17.9, 14.6 Hz). Second diastereomer δ = -155.56 (qdt, *J* = 21.8, 17.3, 14.3 Hz).

^{13}C {^{1}H} NMR (125 MHz, $CDCl_3$): δ = First diastereomer δ = 198.6 (*C^1*), 144.1 (*C^4*), 135.8 (*C^2*), 96.1 (d, *J* = 230.1 Hz, *C^8*), 47.9 (d, *J* = 31.3 Hz, *C^{10}*), 40.2 (d, *J* = 21.8 Hz, *C^5*), 38.7 (*C^7*), 26.7 (d, *J* = 3.2 Hz, *C^6*), 20.4 (d, *J* = 22.3 Hz, *C^9*), 15.7 (*C^3*). Second diastereomer δ = 198.4 (*C^1*), 143.6 (*C^4*), 135.7 (*C^2*), 95.9 (d, *J* = 229.9 Hz, *C^8*), 47.9 (d, *J* = 31.3 Hz, *C^{10}*), 40.2 (d, *J* = 22.3 Hz, *C^5*), 38.1 (*C^7*), 26.0 (d, *J* = 4.1 Hz, *C^6*), 20.4 (d, *J* = 23.9 Hz, *C^9*), 15.7 (*C^3*).

HRMS (ESI) calc: $[M+H]^+$ ($C_{10}H_{15}{}^{35}ClFO$) 205.0790; measured: 205.0786 = 2.00 ppm difference.

IR (neat) ν_{max}/cm^{-1}: 2920, 1724, 1516, 1451, 1229, 1063

6-chloro-5-fluorohexyl 3,5-dinitrobenzoate, 15j

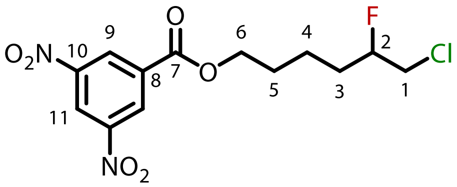

Product **15j** was synthesised from **15a** (0.6 mmol, 177 mg) using ***anti*-chlorofluorination procedure 1**. The product was purified by column chromatography (10-30% EtOAc in pentane) to afford **15j** as an orange oil (171 mg, 82%, rr >19:1).

15j was also prepared from **15a** using ***anti*-chlorofluorination procedure 4** to give **15j** (69% NMR yield, rr >19:1).

^{1}H NMR (400 MHz, CDCl$_3$) δ 9.22 (1H, t, J = 2.1 Hz, H^{11}), 9.15 (d, J = 2.1 Hz, 2H, H^9), 4.79 – 4.57 (1H, m, H^2), 4.48 (2H, t, J = 6.6 Hz, H^6), 3.64 (2H, dd, J = 19.0, 5.1 Hz, H^1), 1.97 – 1.58 (6H, m, H^3, H^4, H^5).

^{13}C {^{1}H} NMR (100 MHz, CDCl$_3$) δ 162.7 (C^7), 148.8 (C^{10}), 134.1 (C^2), 129.5 (C^9), 122.5 (C^{11}), 92.1 (d, J = 175.3 Hz, C^2), 66.7 (C6), 45.6 (d, J = 26.0 Hz, (C1)), 32.1 (d, J = 20.8 Hz, C^3), 28.4 (C^5), 21.5 (C^4).

^{19}F NMR (377 MHz, CDCl$_3$) δ (major regioisomer) -182.2 (ddq, J = 48.0, 29.5, 18.6 Hz), (minor regioisomer) -230.4 (tdd, J = 47.5, 20.7, 13.5 Hz).

HRMS (MALDI) m/z calc: [M^-] ($C_{13}H_{14}{}^{35}ClFN_2O_6$) 348.0530, measured = 348.0537, 2.01 ppm difference.

IR (neat) v_{max}/cm^{-1}: 3018, 2975, 1731, 1548, 1345, 1214, 1046.

Internal alkene *anti*-chlorofluorination products

N-benzyl-N-((3S,4S)-3-chloro-4-fluorohexyl)-4-fluoroaniline, 1b

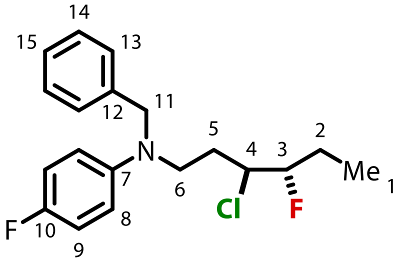

Product **1b** was synthesised from **1a-*cis*** using ***anti*-chlorofluorination procedure 2** and purified using silica-gel chromatography (2% EtOAc:Hexane) to afford **1b** as a colourless oil (171 mg, 85%, dr >19:1, rr = 13:1).

1b was also prepared from **1a-*cis*** using ***anti*-chlorofluorination procedure 4** to give **1b** (94% NMR yield, dr > 19:1, rr 12:1). The reaction was repeated, where **1b** was also prepared from **1a-*cis*** using ***anti*-chlorofluorination procedure 4** but a CH_2Cl_2 volume of 1.25 mL was used to afford **1d** (73% NMR yield, dr >19:1, rr = 11:1).

$\mathbf{R_f}$ = 0.10 (2% EtOAc:Hexane)

^{1}H NMR (400 MHz, $CDCl_3$): δ = 7.35 – 7.30 (2H, m, H^{14}), 7.28 – 7.24 (1H, m, H^{15}), 7.24 – 7.19 (2H, m, H^{13}), 6.95 – 6.87 (2H, m, H^8), 6.71 – 6.64 (2H, m, H^9), 4.56 – 4.49 (2H, m, H^{11}), 4.47 – 4.31 (1H, m, H^3), 3.97 (1H, ddt, J = 21.8, 10.4, 3.2 Hz, H^4), 3.69 (1H, ddd, J = 14.9, 8.5, 4.4 Hz, H^6), 3.52 (1H, dt, J = 15.1, 7.8 Hz, H^6), 2.18 (1H, dddd, J = 14.4, 8.5, 7.3, 3.3 Hz, H^2), 2.06 (1H, dddd, J = 14.6, 10.5, 8.2, 4.3 Hz, H^2), 1.88 – 1.63 (2H, m, H^5), 0.98 (3H, t, J = 7.5 Hz).

^{19}F NMR (376 MHz, $CDCl_3$): δ = -128.39 – -128.47 (1F, m, F^{10}), -188.42 (1F, dddd, J = 46.4, 31.5, 21.9, 14.5 Hz, F^3).

^{13}C {^{1}H} NMR (100 MHz, $CDCl_3$): δ = 155.8 (d, J = 235.5 Hz, C^{10}), 145.1 (d, J = 1.6 Hz, C^7), 138.7 (C^{12}), 128.8 (C^{13}), 127.2 (C^{15}), 126.9 (C^{14}), 115.9 (d, J = 22.1 Hz, C^9), 114.3 (d, J = 7.2 Hz, C^8), 96.0 (d, J = 178.7 Hz, C^3), 60.7 (d, J = 22.5 Hz, C^4), 55.9 (C^{11}), 49.2 (C^6), 31.8 (d, J = 2.0 Hz, C^5), 24.8 (d, J = 21.7 Hz, C^2), 9.6 (d, J = 5.7 Hz, C^1).

HRMS (EI) calc: $[M]^+$ ($C_{19}H_{22}{}^{35}ClF_2N$) 337.1403; measured: 337.1402 = 0.30 ppm difference.

IR (neat) ν_{max}/ cm^{-1}: 1681, 1593, 1487, 1412, 1381, 1319, 1285, 1261, 1238, 1156, 1102, 1007.

***N*-benzyl-*N*-((3S,4S)-3-chloro-4-fluorohexyl)-4-(trifluoromethyl)aniline, 16b**

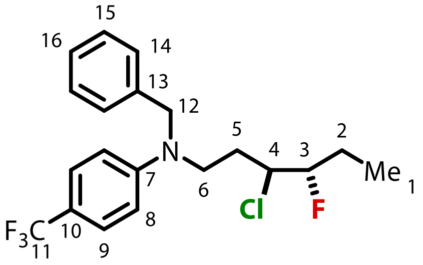

Product **16b** was synthesised from **16a** using ***anti*-chlorofluorination procedure 2** and purified using silica-gel chromatography (2% EtOAc:Hexane) to afford **16b** as a colourless oil (195 mg, 84%, dr >19:1, rr = 9:1).

R_f = 0.10 (2% EtOAc:Hexane)

^{1}H NMR (500 MHz, $CDCl_3$): δ = 7.44 (2H, d, J = 8.8 Hz, H^9), 7.38 – 7.32 (2H, m, H^{15}), 7.31 – 7.26 (1H, m, H^{16}), 7.23 – 7.18 (2H, m, H^{14}), 6.79 (2H, d, J = 8.7 Hz, H^8), 4.70 – 4.60 (2H, m, H^{12}), 4.44 (1H, dddd, J = 47.0, 8.7, 4.4, 3.0 Hz, H^3), 3.97 (1H, m, H^4), 3.84 (1H, ddd, J = 15.2, 9.1, 4.3 Hz, H^6), 3.64 (1H, ddd, J = 15.4, 8.8, 7.0 Hz, H^6), 2.25 (1H, m,H^2), 2.13 (1H, m, H^2), 1.91 – 1.65 (2H, m, H^5), 1.01 (3H, t, J = 7.5 Hz, H^1).

^{19}F NMR (376 MHz, $CDCl_3$): δ = -60.79 (3F, s, F^{11}), -188.26 (1F, dddd, J = 46.5, 31.5, 21.5, 14.4 Hz, F^3)

^{13}C {^{1}H} NMR (125 MHz, $CDCl_3$): δ = 150.5 (C^7), 137.7 (C^{13}), 129.0 (C^{14}), 127.4 (C^{16}), 126.8 (q, J = 4.1 Hz, C^9), 126.5 (C^{15}), 125.2 (q, J = 125.2, C^{11}), 118.2 (q, J = 33.1 Hz, C^{10}), 111.5 (C^8), 96.0 (d, J = 178.7 Hz, C^3), 60.5 (d, J = 22.4 Hz, C^4), 54.6 (C^{12}), 48.7 (C^6), 31.6 (d, J = 2.3 Hz, C^5), 24.8 (d, J = 21.6 Hz, C^2), 9.6 (d, J = 5.7 Hz, C^1).

HRMS (EI) calc: $[M]^+$ ($C_{20}H_{22}{}^{35}ClF_4N$) 387.1371; measured: 387.1370 = 0.26 ppm difference.

IR (neat) ν_{max}/ cm^{-1}: 1699, 1615, 1530, 1494, 1453, 1403, 1325, 1273, 1249, 1199, 1159, 1107, 1067.

N-benzyl-N-((3S,4S)-3-chloro-4-fluorohexyl)-4-iodoaniline, 17b

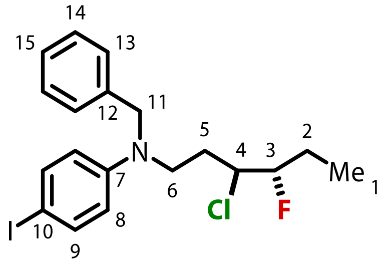

Product **17b** was synthesised from **17a** using ***anti*-chlorofluorination procedure 2** and purified using silica-gel chromatography (2% EtOAc:Hexane) to afford **17b** as a colourless oil (184 mg, 69%, dr >19:1, rr = 10:1).

R_f = 0.10 (2% EtOAc:Hexane)

^{1}H NMR (400 MHz, $CDCl_3$): δ = 7.43 (2H, d, *J* = 9.4 Hz, H^9), 7.35 – 7.16 (5H, m, $H^{13,14,15}$), 6.51 (2H, d, *J* = 9.4 Hz, H^8), 4.59 – 4.52 (2H, m, H^{11}), 4.40 (1H, dddd, *J* = 47.0, 8.6, 4.4, 3.1 Hz, H^3), 3.94 (1H, m, H^4), 3.73 (1H, ddd, *J* = 15.0, 8.8, 4.3 Hz, H^6), 3.55 (1H, ddd, *J* = 15.3, 8.5, 7.2 Hz, H^6), 2.25 – 2.02 (2H, m, H^5), 1.88 – 1.64 (2H, m, H^2), 0.99 (3H, t, *J* = 7.5 Hz, H^1).

^{19}F NMR (376 MHz, $CDCl_3$): δ = -188.29 (1F, dddd, *J* = 46.6, 31.5, 21.5, 14.4 Hz, F^3).

^{13}C {^{1}H} NMR (100 MHz, $CDCl_3$): δ = 147.9 (C^{10}), 138.1 (C^{12}), 138.0 (C^9), 128.9 (C^{13}), 127.3 (C^{15}), 126.6(C^{14}), 114.9 (C^8), 114.8 (C^7), 96.0 (d, *J* = 179.3 Hz, C^3), 60.6 (d, *J* = 21.7 Hz, C^4), 54.8 (C^{11}), 48.7 (C^6), 31.7 (d, *J* = 2.0 Hz, C^5), 24.8 (d, *J* = 21.4 Hz, C^2), 9.6 (d, *J* = 5.2 Hz, C^1).

HRMS (EI) calc: $[M]^+$ ($C_{19}H_{22}{}^{35}ClFIN$) 445.0464; measured: 445.0460 = 0.90 ppm difference.

IR (neat) v_{max}/ cm^{-1}: 1584, 1495, 1451, 1397, 1382, 1329, 1232, 1014.

N-benzyl-N-((3S,4S)-3-chloro-4-fluorohexyl)aniline, 18b

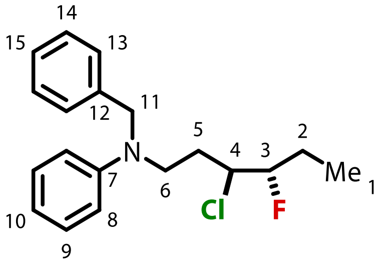

Product **18b** was synthesised from **18a** using ***anti*-chlorofluorination procedure 2** and purified using silica-gel chromatography (2% EtOAc:Hexane) to afford **18b** as a colourless oil (162 mg, 85%, dr >19:1, rr = 13:1).

R_f = 0.10 (2% EtOAc:Hexane).

^{1}H NMR (400 MHz, $CDCl_3$): δ = 7.37 – 7.19 (8H, m, $H^{13,14,15,Ph}$), 6.79 – 6.70 (2H, m, H^{Ph}), 4.65 – 4.54 (2H, m, H^{11}), 4.41 (1H, dddd, *J* = 47.0, 8.6, 4.4, 3.1 Hz, H^3), 3.98 (1H, m, H^4), 3.77 (1H, ddd, *J* = 14.9, 8.7, 4.4 Hz, H^6), 3.58 (1H, ddd, *J* = 15.2, 8.4, 7.2 Hz, H^6), 2.23 (1H, dddd, *J* = 14.4, 8.7, 7.2, 3.2 Hz, H^5), 2.10 (1H, dddd, *J* = 14.5, 10.6, 8.3, 4.3 Hz, H^5), 1.89 – 1.64 (2H, m, H^2), 0.99 (3H, t, *J* = 7.5 Hz).

^{19}F NMR (376 MHz, $CDCl_3$): δ = -188.41 (1F, dddd, *J* = 46.5, 31.5, 21.8, 14.5 Hz, F^3).

^{13}C {^{1}H} NMR (100 MHz, $CDCl_3$): δ = 148.4 (C^7), 138.8 (C^{12}), 129.5 (C^8), 128.8 (C^{13}), 127.1 (C^{15}), 126.8 (C^{14}), 116.9 (C^{10}), 112.6 (C^9), 96.1 (d, *J* = 178.6 Hz, C^3), 60.8 (d, *J* = 22.5 Hz, C^4), 55.0 (C^{11}), 48.6 (C^6), 31.9 (d, *J* = 2.0 Hz, C^5), 22.3 (d, *J* = 22.3 Hz, C^2), 9.6 (d, *J* = 5.2 Hz, C^1).

HRMS (APCI) calc: $[M]^+$ ($C_{19}H_{23}{}^{35}ClFN$) 319.1498; measured: 319.1495 = 0.94 ppm difference.

IR (neat) ν_{max}/ cm^{-1}: 1698, 1589, 1453, 1393, 1380, 1317, 1293, 1252, 1203, 1183, 1117, 1058.

N-benzyl-N-((3S,4S)-3-chloro-4-fluorohexyl)-4-methylaniline, 19b

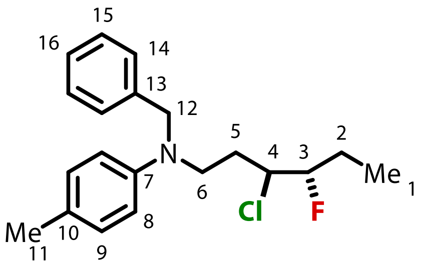

Product **19b** was synthesised from **19a** using ***anti*-chlorofluorination procedure 2** and purified using silica-gel chromatography (4% EtOAc:Hexane) to afford **19b** as a colourless oil (124 mg, 62 %, dr >19:1, rr = 13.5:1).

R_f = 0.25 (4% EtOAc:Hexane)

^{1}H NMR (500 MHz, $CDCl_3$): δ = 7.37 – 7.30 (2H, m, H^{Bn}), 7.29 – 7.23 (3H, m, H^{Bn}), 7.07 – 7.02 (2H, m, H^9), 6.74 – 6.67 (2H, m, H^8), 4.59 – 4.54 (2H, m, H^{12}), 4.49 – 4.31 (1H, m, H^3), 4.06 – 3.93 (1H, m, H^4), 3.78 – 3.68 (1H, m, H^6), 3.63 – 3.52 (1H, m, H^6), 2.30 – 2.18 (4H, m, $H^{5,11}$), 2.13 – 2.04 (1H, m, H^5), 1.90 – 1.63 (2H, m, H^2), 1.00 (3H, t, *J* = 7.5 Hz).

^{19}F NMR (376 MHz, $CDCl_3$): δ = -187.95 – -118.89 (1F, m, F^3).

^{13}C {^{1}H} NMR (125 MHz, $CDCl_3$): δ = 146.3 (C^{10}), 139.1 (C^{13}), 130.0 (C^9), 128.7 (C^{14}), 127.0 (C^{16}), 126.2 (C^{15}), 123.8 (C^8), 113.1 (C^7), 96.1 (d, *J* = 180.9 Hz, C^3), 60.8 (d, *J* = 22.9 Hz, C^4), 55.4 (C^{12}), 48.6 (C^6), 31.9 (d, *J* = 2.0 Hz, C^5), 24.9 (d, *J* = 21.5 Hz, C^2), 20.3 (C^{11}), 9.5 (d, *J* = 5.6 Hz, C^1).

HRMS (APCI) calc: $[M+H]^+$ ($C_{20}H_{26}{}^{35}ClFN$) 334.1732; measured: 334.1730 = 0.59 ppm difference.

IR (neat) ν_{max}/ cm^{-1}: 1657, 1387, 1312, 1305, 1284, 1251, 1181, 1118, 1053, 1031.

***N*-((3S,4R)-3-chloro-4-fluorohexyl)-4-fluoroaniline, 20d**

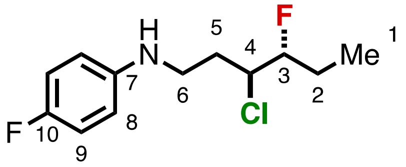

Product **20d** was synthesised from **20a** using ***anti*-chlorofluorination procedure 2** and purified using silica-gel chromatography (30% EtOAc:Hexane) to afford **20d** as a viscous yellow oil (86 mg, 58%, rr = 11:1).

R_f = 0.25 (30% EtOAc:Hexane)

^{1}H NMR (500 MHz, CDCl3): δ = 6.90 (2H, t, *J* = 8.7 Hz, *H^8*), 6.64 – 6.52 (2H, m, *H^9*), 4.45 (1H, dddd, *J* = 47.7, 8.3, 6.5, 3.2 Hz, *H^3*), 4.06 (1H, m, *H^4*), 3.40 (1H, ddd, *J* = 12.5, 7.6, 4.6 Hz, *H^6*), 3.32 (1H, m, *H^6*), 2.24 (1H, dddd, *J* = 14.5, 7.7, 2.7, 1.4 Hz, *H^5*), 1.97 – 1.66 (3H, m, *$H^{5,2}$*), 1.03 (3H, t, *J* = 7.4 Hz, *H^1*).

^{19}F NMR (376 MHz, $CDCl_3$): δ = -127.63 (1F, tt, *J* = 8.6, 4.4 Hz, *F^{10}*), -183.85 (1F, dddd, *J* = 47.9, 33.8, 19.8, 10.7 Hz, *F^3*).

^{13}C {^{1}H} NMR (125 MHz, $CDCl_3$): δ = 156.1 (d, *J* = 231.1 Hz, *C^{10}*), 144.2 (d, *J* = 2.0 Hz, *C^7*), 115.9 (d, *J* = 22.4 Hz, *C^9*), 113.9 (d, *J* = 7.3 Hz, *C^8*), 96.3 (d, *J* = 176.2 Hz, *C^3*), 60.1 (d, *J* = 24.1 Hz, *C^4*), 41.5 (*C^6*), 33.0 (d, *J* = 4.0 Hz, *C^5*), 25.0 (d, *J* = 23.3 Hz, *C^2*), 9.2 (d, *J* = 4.2 Hz, *C^1*).

HRMS (ESI) calc: $[M+H]^+$ ($C_{12}H_{17}F_2{}^{35}ClN$) 248.1012; measured: 248.1017 = 2.00 ppm difference.

IR (neat) ν_{max}/ cm^{-1}: 1612, 1511, 1462, 1435, 1406, 1393, 1382, 1255, 1156.

N-benzyl-N-((3S,4R)-3-chloro-4-fluorohexyl)-4-fluoroaniline, 21d

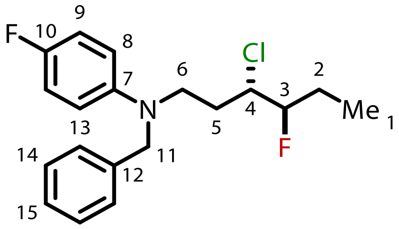

Product **21d** was synthesised from **1a-*trans*** (0.6 mmol, 170 mg) using ***anti*-chlorofluorination procedure 2**. The product was purified by column chromatography (3% EtOAc in pentane) to afford **21d** as a yellow oil (132 mg, 67%, rr 11:1).

21d was also prepared from **1a-*trans*** using ***anti*-chlorofluorination procedure 4** using 1.25 mL of CH_2Cl_2 to give **21d** (56% NMR yield, dr > 19:1, rr 11:1).

^{1}H NMR (500 MHz, $CDCl_3$) δ 7.35 – 7.27 (2H, m, H^{14}), 7.27 – 7.18 (3H, m, H^{13}, H^{15}), 6.94 – 6.85 (2H, m, H^8), 6.71 – 6.62 (2H, m, H^9), 4.49 (2H, s, H^{11}), 4.40 (1H, dddd, J = 47.7, 8.1, 6.4, 3.2 Hz, H^3), 3.94 (1H, tdd, J = 10.4, 6.4, 2.6 Hz, H^4), 3.70 (1H, ddd, J = 14.0, 9.2, 4.2 Hz, H^6), 3.51 (1H, ddd, J = 15.2, 8.8, 6.9 Hz, H^6), 2.28 (1H, ddddd, J = 14.5, 9.4, 6.9, 2.6, 1.4 Hz, H^5), 1.92 (1H, dddd, J = 14.4, 10.1, 8.9, 4.2 Hz, $H^{5'}$), 1.87 – 1.66 (2H, m, H^2), 1.00 (3H, t, J = 7.4 Hz, H^1).

^{13}C {^{1}H} NMR (101 MHz, $CDCl_3$) δ 155.5 (d, J = 235.8 Hz, C^{10}), 144.9 (d, J = 1.8 Hz, C^7), 138.5 (C^{12}), 128.6 (C^{13}), 127.0 (C^{15}), 126.7 (C^{14}), 115.7 (d, J = 22.0 Hz, C^9), 114.0 (d, J = 7.2 Hz, C^8), 96.2 (d, J = 177.1 Hz, C^3), 60.1 (d, J = 24.8 Hz, C^4), 55.5 (C^{11}), 48.7 (C^6), 30.7 (d, J = 3.7 Hz, C^5), 24.8 (d, J = 21.4 Hz, C^2), 9.1 (d, J = 4.1 Hz, C^1).

^{19}F NMR (377 MHz, $CDCl_3$) δ (major regioisomer) -128.69 (tt, J = 8.5, 4.3 Hz), -183.83 (dddd, J = 47.9, 32.2, 20.2, 10.7 Hz). (minor regioisomer) -128.76 (tt, J = 8.4, 4.3 Hz), -181.98 – -182.43 (m).

HRMS (ESI) m/z calc: [M+H$^+$] ($C_{19}H_{22}ClF_2N$) 338.1482, measured = 338.1479, 0.88 ppm difference.

IR (neat) ν_{max}/cm^{-1}: 2972, 2881, 1379, 1087, 1045, 879.

(3R,4R)-N-benzhydryl-N-benzyl-3-chloro-4-fluorohexan-1-amine 22b

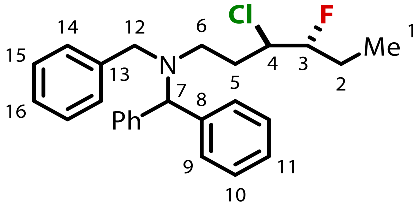

Product **22b** was synthesised using ***anti*-chlorofluorination procedure 2** and purified using silica-gel chromatography (198 mg, 81%, dr >19:1, rr = 16:1).

^{1}H NMR (400 MHz, CDCl$_3$): δ = 7.48 – 7.23 (15H, m, H^{Ar}), 4.92 (1H, s, H^{7}), 4.23 – 4.05 (1H, m, H^{3}), 3.92 – 3.79 (1H, m, H^{4}), 3.71 – 3.59 (2H, m, H^{12}), 2.79 – 2.62 (2H, m, H^{6}), 2.05 – 1.87 (2H, m, H^{5}), 1.80 – 1.51 (2H, m, H^{2}), 0.94 (3H, t, *J* = 7.4 Hz, H^{1}).

^{19}F NMR (376 MHz, CDCl$_3$): δ = -189.88 (1F, dddd, *J* = 46.5, 32.2, 22.8, 14.0 Hz, F^{3})

^{13}C NMR (100 MHz, CDCl$_3$): δ = 141.9 (C^{Ar}), 139.9 (C^{Ar}), 128.9 (C^{Ar}), 128.8 (C^{Ar}), 128.5 (C^{Ar}), 127.9 (C^{Ar}), 127.4 (C^{Ar}), 127.2 (C^{Ar}), 95.6 (d, *J* = 180.1 Hz, C^{3}), 70.3 (C^{7}), 60.9 (d, *J* = 25.3 Hz, C^{4}), 55.3 (C^{12}), 47.5 (C^{6}), 31.6 (d, *J* = 3.2 Hz, C^{5}), 24.9 (d, *J* = 24.1 Hz, C^{2}), 9.5 (C^{1}).

HRMS (EI) calc: $[M+H]^+$ ($C_{26}H_{30}{}^{35}ClFN$) 410.2045; measured: 410.2032 = 3.17 ppm difference.

IR (neat) ν_{max}/ cm^{-1}: 1649, 1599, 1492, 1452, 1277, 1076, 1027

4-(trifluoromethyl)phenyl (9R,10S)-9-chloro-10-fluorooctadecanoate, 23d

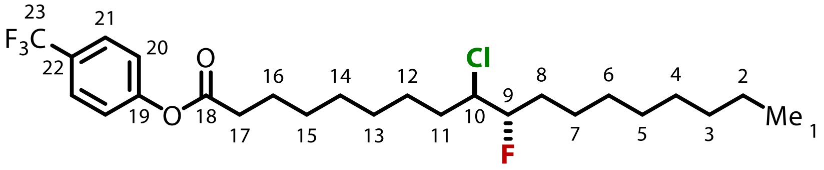

Product **23d** was synthesised from **23a** using ***anti*-chlorofluorination procedure 2** and purified using silica-gel chromatography (5% EtOAc:Hexane) to afford **23**d as a colourless oil (230 mg, 81%, dr >19:1, rr = 1:1).

R_f = 0.30 (5% EtOAc:Hexane)

^{1}H NMR (400 MHz, $CDCl_3$): δ = 7.68 – 7.63 (2H, m, H^9), 7.24 – 7.17 (2H, m, H^{10}), 4.56 – 4.36 (1H, m, H^9), 3.96 – 3.85 (1H, m, H^{10}), 2.61 – 2.55 (2H, m, H^7), 1.93 – 1.22 (2H, m, $H^{2,3,4,5,6,7,8,11,12,13,14,15,16}$), 0.96 – 0.80 (3H, m, H^1).

^{19}F NMR (376 MHz, $CDCl_3$): δ = -62.11 (3F, m, F^{23}), -181.71 – -182.26 (1F, m, F^9).

^{13}C {^{1}H} NMR (100 MHz, $CDCl_3$): δ = 171.8 (C^{18}), 153.4 (C^{19}), 128.1 (q, J = 33.1 Hz, C^{22}), 126.9 (q, J = 3.7 Hz, C^{20}), 123.9 (q, J = 271.9 Hz, C^{23}), 122.2 (C^{20}), 95.4 (d, J = 174.9 Hz, C^9), 63.2 (d, J = 23.9 Hz, C^{10}), 34.4 (C^{17}), 33.4 (d, J = 4.5 Hz, C^{11}), 32.0 (C^3), 31.7 (d, J = 21.4 Hz, C^8), 29.5 ($C^{3,13,14}$), 29.5 ($C^{3,13,14}$), 29.4 ($C^{3,13,14}$), 29.3 (C^6), 29.2 (C^{12}), 29.1 (C^{15}), 26.3 (C^5), 25.0 (d, J = 4.0 Hz, C^7), 24.9 (C^{16}), 22.8 (C^2), 14.2 (C^1).

HRMS (EI) calc: $[M]^+$ (503.2310); measured: 503.2316 = 1.19 ppm difference.

IR (neat) $ν_{max}$/ cm^{-1}: 1764, 1613, 1513, 1323, 1209, 1166, 1125, 1103, 1063

(R)-4-(2-chloro-1-fluoro-2-methylpropyl)-5-methylthiazole, 24b

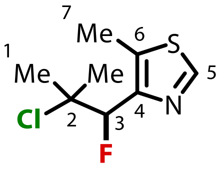

major

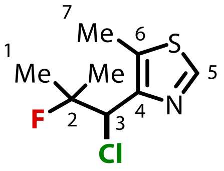

minor

Product **24b** was synthesised from **24a** using ***anti*-chlorofluorination procedure 2** and purified using silica-gel chromatography (30% EtOAc:Hexane) to afford **24b** as a colourless oil (103 mg, 83%, rr = 2:1).

R_f = 0.25 (30% EtOAc:Hexane)

^{1}H NMR (500 MHz, $CDCl_3$): Major regioisomer δ = 8.73 (1H, s, H^5), 5.64 (1H, d, J = 43.9 Hz, H^3), 2.46 (3H, s, H^7), 1.65 (3H, s, H^1), 1.54 (3H, s, H^1). Minor regioisomer δ = 8.70 (1H, s, H^5), 5.16 (1H, d, J = 17.8 Hz, H^3), 2.42 (3H, s, H^7), 1.51 (3H, d, J = 21.4, H^1), 1.39 (3H, d, J = 21.4 Hz, H^1).

^{19}F NMR (376 MHz, $CDCl_3$): Major regioisomer δ = -161.82 (1F, d, J = 43.9 Hz, F^3) Minor regioisomer δ = -145.56 – -145.99 (1F, m, F^2).

^{13}C {^{1}H} NMR (125 MHz, $CDCl_3$): Major regioisomer δ = 153.7 (C^5), 152.1 (d, J = 5.3 Hz, C^6), 126.0 (d, J = 24.7 Hz, C^4), 92.6 (d, J = 180.7 Hz, C^3), 69.3 (d, J = 24.3 Hz, C^2), 28.5 (d, J = 2.4 Hz, C^1), 28.4 (d, J = 2.4 Hz, C^1), 15.9 (C^7). Minor regioisomer δ = 153.9 (C^5), 150.2 (C^6), 130.0 (C^4), 96.0 (d, J = 179.6 Hz, C^2), 60.7 (d, J = 25.9 Hz, C^3), 24.7 (d, J = 23.7 Hz, C^1), 24.6 (d, J = 23.7 Hz, C^1), 15.2 (C^7).

HRMS (APCI) calc: $[M+H]^+$ ($C_8H_{12}{}^{35}ClFNS$) 208.0358; measured: 208.0359 = 0.48 ppm difference.

IR (neat) v_{max}/ cm^{-1}: 1660, 1537, 1455, 1415, 1388, 1372, 1316, 1121, 1018.

(2S,3S)-N,N-dibenzyl-2-chloro-3-fluorohexan-1-amine, 25b

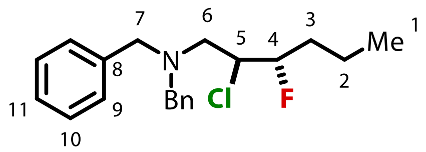

Product **25b** was synthesised from **25a** using ***anti*-chlorofluorination procedure 2** and purified using silica-gel chromatography (2% EtOAc:Hexane) to afford **25b** a colourless oil (141 mg, 71%, rr and dr >19:1).

R_f = 0.15 (2% EtOAc:Hexane)

^{1}H NMR (400 MHz, CDCl$_3$): δ = 7.36 – 7.25 (10H, m, H^{Ar}), 4.78 (1H, dddd, J = 46.7, 8.7, 4.8, 1.9 Hz, H^{4}), 3.72 – 3.59 (5H, m, $H^{7,5}$), 3.07 (1H, dd, J = 13.6, 8.5 Hz, H^{6}), 2.83 (1H, ddd, J = 13.6, 6.1, 1.1 Hz, H^{6}), 1.86 – 1.73 (1H, m, H^{3}), 1.52 – 1.23 (3H, m, $H^{3,2}$), 0.93 (3H, t, J = 7.2 Hz, H^{1}).

^{19}F NMR (376 MHz, CDCl$_3$): δ = -194.23 (1F, dddd, J = 46.8, 29.4, 26.1, 13.1 Hz, F^{4}).

^{13}C {^{1}H} NMR (100 MHz, CDCl$_3$): δ = 139.0 (C^{8}), 129.1 (C^{10}), 128.5 (C^{9}), 127.4 (C^{11}), 90.9 (d, J = 179.8 Hz, C^{4}), 60.9 (d, J = 20.0 Hz, C^{5}), 59.7 (C^{7}), 57.8 (d, J = 3.3 Hz, C^{6}), 34.1 (d, J = 20.4 Hz, C^{3}), 18.4 (d, J = 5.6 Hz, C^{2}), 13.9 (C^{1}).

HRMS (ESI) calc: $[M+H]^+$ ($C_{20}H_{26}{}^{35}ClFN$) 334.1732; measured 334.1737 = 1.49 ppm difference.

IR (neat) ν_{max}/ cm^{-1}: 1703, 1601, 1494, 1452, 1367, 1308, 1247, 1124, 1070.

N,N-dibenzyl-3-chloro-4-fluorohexan-1-amine, 26b

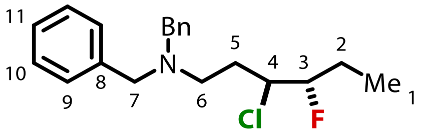

Product **26b** was synthesised from **26a** using ***anti*-chlorofluorination procedure 2** and purified using silica-gel chromatography (2% EtOAc:Hexane) to afford **26b** as a colourless oil (164 mg, 82%, rr and dr >19:1).

R_f = 0.15 (2% EtOAc:Hexane)

^{1}H NMR (400 MHz, $CDCl_3$): 7.37 – 7.23 (10H, m, $H^{9,10,11}$), 4.23 – 4.00 (2H, m, $H^{3,4}$), 3.57 (4H, s, H^7), 2.71 – 2.57 (2H, m, H^6), 2.05 – 1.60 (4H, m, $H^{2,5}$), 0.93 (3H, t, J = 6.7 Hz, H^1).

^{19}F NMR (376 MHz, $CDCl_3$): -191.15 (dddd, J = 46.5, 31.6, 24.3, 13.5 Hz)

^{13}C {^{1}H} NMR (100 MHz, $CDCl_3$): 139.5 (C^8), 129.0 (C^9), 128.4 (C^{10}), 127.2 (C^{11}), 95.1 (d, J = 178.0 Hz, C^3), 60.7 (d, J = 21.5 Hz, C^4), 58.9 (C^7), 50.5 (C^6), 32.3 (d, J = 3.3 Hz, C^5), 25.1 (d, J = 20.3 Hz, C^2), 9.6 (d, J = 5.8 Hz, C^1).

HRMS (APCI) calc: $(M+H)^+$ ($C_{20}H_{26}{}^{35}ClFN$) 334.1732; measured: 334.1720 = 3.59 ppm difference.

IR (neat) ν_{max}/ cm^{-1}: 1602, 1494, 1453, 1377, 1119, 1071, 1028.

(4S,5S)-N,N-dibenzyl-4-chloro-5-fluorohexan-1-amine, 27b

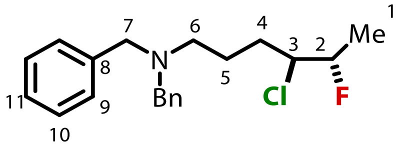

Product **27b** was synthesised from **27a** using ***anti*-chlorofluorination procedure 2** and purified using silica-gel chromatography (2% EtOAc:Hexane) to afford **27b** a colourless oil (150 mg, 75%, rr and dr >19:1).

R_f = 0.15 (2% EtOAc:Hexane)

^{1}H NMR (500 MHz, $CDCl_3$): δ = 7.39 – 7.21 (10H, m, H^{Bn}), 4.63 (1H, dqd, J = 46.8, 6.3, 3.6 Hz, H^2), 3.72 – 3.47 (5H, m, $H^{3,7}$), 2.51 – 2.38 (2H, m, H^6), 1.90 – 1.58 (4H, m, $H^{4,5}$), 1.36 (3H, dd, J = 23.9, 6.2 Hz, H^1).

^{19}F NMR (376 MHz, $CDCl_3$): δ = -179.74 (1F, dqd, J = 47.5, 23.7, 19.3 Hz, F^2)

^{13}C {^{1}H} NMR (125 MHz, $CDCl_3$): δ = 139.9 (C^8), 129.0 (C^9), 128.3 (C^{10}), 127.0 (C^{11}), 91.1 (d, J = 174.8 Hz, C^2), 63.9 (d, J = 22.3 Hz, C^3), 58.6 (C^7), 52.2 (C^6), 31.1 (d, J = 2.6 Hz, C^4), 23.9 (C^5), 17.5 (C^1).

HRMS (ESI) calc: $[M+H]^+$ ($C_{20}H_{26}N^{35}ClF$) 334.1732; measured: 334.1748 = 4.70 ppm difference.

IR (neat) ν_{max}/ cm^{-1}: 1601, 1493, 1452, 1381, 1249, 1125, 1070, 1031.

(3S,5S,6S,8S,9S,10R,13R,14S)-5-chloro-6-fluoro-10,13-dimethyl-17-((R)-6-methylheptan-2-yl)hexadecahydro-1H-cyclopenta[a]phenanthren-3-ol, 28b

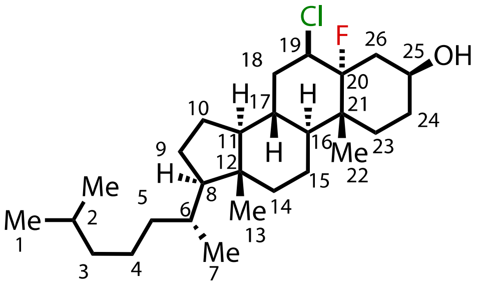

Product **28b** was synthesised cholesterol using ***anti*-chlorofluorination procedure 2** and purified using silica-gel chromatography (30% EtOAc:Hexane) to afford **28b** as a colourless oil (193 mg, 73%, dr and rr >19:1).

R_f = 0.15 (30% EtOAc:Hexane)

^{1}H NMR (500 MHz, $CDCl_3$): δ = 4.46 – 4.34 (1H, m, H^{19}), 4.11 – 4.03 (1H, m, H^{25}), 2.52 – 2.44 (1H, m, H^{26}), 2.14 – 2.07 (1H, m, H^{18}), 2.04 – 1.94 (2H, m, H^{9}), 1.91 – 1.79 (3H, m, $H^{14,24,26}$), 1.70 – 1.69 (1H, m, H^{23}), 1.61 – 1.48 (4H, m, $H^{2,15,17,23}$), 1.47 – 1.42 (2H, m, H^{24}), 1.39 – 1.19 (7H, m, $H^{3,5,6,10,14,15,18}$), 1.18 – 1.03 (8H, m, $H^{3,4,5,8,10,11,16}$), 0.99 (3H, s, H^{22}), 0.88 (3H, d, J = 6.5 Hz, H^{7}), 0.85 (3H, d, J = 6.4 Hz, H^{1}), 0.84 (3H, d, J = 6.4 Hz, H^{1}), 0.64 (3H, s, H^{13}).

^{19}F NMR (376 MHz, $CDCl_3$): δ = -152.84 (1F, m, F^{20}).

^{13}C {^{1}H} NMR (125 MHz, $CDCl_3$): δ = 101.0 (d, J = 183.1 Hz, C^{20}), 66.0 (C^{25}), 62.9 (d, J = 23.1 Hz, C^{19}), 56.2 (C^{8}), 56.0 (C^{11}), 43.4 (d, J = 5.3 Hz, C^{16}), 42.9 (C^{12}), 42.5 (d, J = 18.1 Hz, C^{21}), 39.7 (C^{15}), 39.6 (d, J = 17.9 Hz, C^{26}), 39.6 (C^{10}), 36.2 (C^{5}), 35.8 (C^{6}), 35.5 (C^{17}), 30.9 (C^{9}), 30.7 (C^{14}), 28.2 (C^{17}), 28.1 (C^{24}), 27.1 (C^{23}), 25.6 (C^{18}), 24.3(C^{5}), 23.9 (C^{3}), 22.9 (C^{1}), 22.7 (C^{1}), 21.1 (C^{4}), 18.7 (C^{7}), 17.1 (d, J = 6.3 Hz, C^{22}), 12.1 (C^{13}).

HRMS (MALDI) calc: $[M+Na]^+$ ($C_{27}H_{46}{}^{35}ClFONa$) 463.3113; measured: 463.3119 = 1.30 ppm difference.

IR (neat) ν_{max}/ cm^{-1}: 2923, 2854, 1458, 1376, 1045.

Tert-butyl(((3S,4S)-3-chloro-4-fluorohexyl)oxy)diphenylsilane, 29b

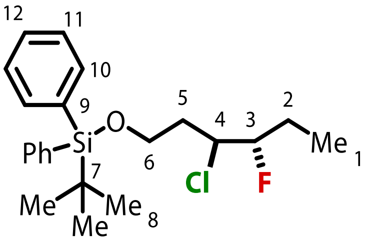

major

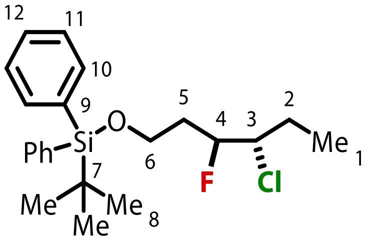

minor

Product **29b** was synthesised from **29a** using ***anti*-chlorofluorination procedure 2**, except the TEAC was added over a period of only 15 min, and the reaction was quenched immediately with no overnight stir. It was purified using silica-gel chromatography (5% EtOAc:Hexane) to afford **29b** as a colourless oil (167 mg, 71%, dr >19:1, rr = 1.5:1).

R_f = 0.50 (5% EtOAc:Hexane)

^{1}H NMR (500 MHz, $CDCl_3$): Major regioisomer δ = 7.71 – 7.63 (4H, m, H^{12}), 7.48 – 7.36 (6H, m, $H^{11,13}$), 4.48 (1H, dddd, J = 47.0, 8.1, 4.6, 3.1 Hz, H^3), 4.36 – 4.25 (1H, m, H^4), 3.96 – 3.74 (2H, m, H^6), 2.15 – 1.66 (4H, m, $H^{2,5}$), 1.06 (9H, s, H^8), 1.03 (3H, t, J = 7.6 Hz, H^1). Minor regioisomer δ = 7.71 – 7.63 (4H, m, H^{12}), 7.48 – 7.36 (6H, m, $H^{11,13}$), 4.99 – 4.82 (1H, m, H^4), 3.96 – 3.74 (2H, m, $H^{3,6}$), 2.15 – 1.66 (4H, m, $H^{2,5}$), 1.09 (3H, t, J = 6.9 Hz, H^1), 1.06 (9H, s, H^8).

^{19}F NMR (376 MHz, $CDCl_3$): Major regioisomer δ = -189.57 (dddd, J = 47.0, 30.2, 23.3, 14.6 Hz). Major regioisomer δ = -192.31 (dddd, J = 47.5, 32.0, 22.3, 15.4 Hz).

^{13}C {^{1}H} NMR (125 MHz, $CDCl_3$): Major regioisomer δ = 135.6 (C^{12}), 133.7 (C^9), 129.9 (C^{Ar}), 127.9 (C^{Ar}), 95.9 (d, J = 177.2 Hz, C^3), 65.3 (d, J = 21.8 Hz, C^4), 60.3 (C^6), 37.1 (d, J = 2.7 Hz, C^5), 27.0 (C^8), 25.1 (d, J = 20.2 Hz, C^2), 19.4 (C^7), 9.5 (d, J = 5.9 Hz, C^1). Minor regioisomer δ = 135.7 (C^{12}), 133.5 (C^9), 129.9 (C^{Ar}), 127.9 (C^{Ar}), 91.1 (d, J = 176.9 Hz, C^4), 59.6 (d, J = 21.6 Hz, C^3), 59.6 (d, J = 5.9 Hz, C^6), 34.9 (d, J = 21.4 Hz, C^5), 27.6 (d, J = 3.3 Hz, C^2), 27.0 (C^8), 19.4 (C^7), 11.4 (C^1).

HRMS (APCI) calc: $[M+H]^+$ ($C_{22}H_{31}O^{35}ClFSi$) 393.1811; measured: 393.1794 = 4.32 ppm difference.

IR (neat) ν_{max}/ cm^{-1}: 1588, 1472, 1427, 1388, 1362, 1262, 1107, 1007.

1-chloro-2-fluoropropyl 4-fluorobenzoate, 30d

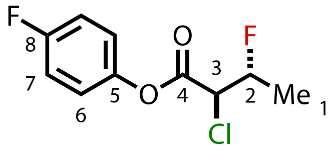

Product **30d** was synthesised from **30a** using ***anti*-chlorofluorination procedure 2**, except that the reaction mixture was stirred at room temperature for 48 hours following TEAC addition. The product was purified using silica-gel chromatography (25% EtOAc:Hexane) to afford **30d** as a colourless oil (90 mg, 70%, dr and rr >19:1).

R_f = 0.20 (25% EtOAc:Hexane)

^{1}H NMR (400 MHz, $CDCl_3$): δ = 7.13 – 7.05 (4H, m, $H^{6,7}$), 5.22 – 5.03 (1H, m, H^2), 4.48 (1H, dd, J = 7.5, 6.8 Hz, H^3), 1.60 (3H, dd, J = 24.2, 6.8 Hz, H^1).

^{19}F NMR (376 MHz, $CDCl_3$): δ = -115.66 – -115.75 (1F, m, F^8), -172.58 (1F, dqd, J = 46.0, 24.3, 6.9 Hz, F^2).

^{13}C {^{1}H} NMR (100 MHz, $CDCl_3$): δ = 166.0 (d, J = 4.5 Hz, C^4), 160.9 (d, J = 249.5 Hz, C^8), 140.0 (C^5), 122.7 (d, J = 9.2 Hz, C^6), 116.5 (d, J = 24.9 Hz, C^7), 89.7 (d, J = 170.3 Hz, C^2), 57.9 (d, J = 29.0 Hz, C^3), 17.5 (d, J = 21.4 Hz, C^1).

HRMS (APCI) calc: $[M+H]^+$ ($C_{10}H_{10}{}^{35}ClF_2O_2$) 235.0332; measured: 235.0335 = 1.28 ppm difference.

IR (neat) ν_{max}/ cm^{-1}: 1766, 1632, 1599, 1502, 1453, 1387, 1290, 1232, 1184, 1144, 1104, 1089, 1074, 1059.

(2S,3R)-N,N-dibenzyl-2-chloro-3-fluorobutanamide, 31d

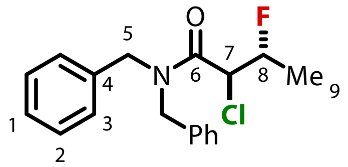

Product **31d** was synthesised from **31a** using ***anti*-chlorofluorination procedure 2**, except that the reaction mixture was stirred at room temperature for 48 hours following TEAC addition. The product was then purified using silica-gel chromatography (50% EtOAc:Hexane) to afford **31d** a colourless oil (105 mg, 55%, dr and rr >19:1).

R_f = 0.30 (50% EtOAc:Hexane)

^{1}H NMR (500 MHz, $CDCl_3$): δ = 7.43 – 7.11 (10H, m, H^{Ar}), 4.96 (1H, d, J = 14.9 Hz, H^8), 4.72 – 4.25 (5H, m, $H^{7,5}$), 1.57 (3H, dd, J = 24.6, 6.4 Hz, H^9).

^{19}F NMR (376 MHz, $CDCl_3$): δ = -170.19 – -170.59 (1F, m, F^8).

^{13}C {^{1}H} NMR (125 MHz, $CDCl_3$): δ = 167.7 (d, J = 167.7 Hz, C^6), 136.5 (C^4), 135.6 (C^4), 129.3 (C^3), 128.9 (C^3), 128.2 (C^1), 128.1 (C^1), 127.8 (C^2), 126.5 (C^2), 89.8 (d, J = 170.8 Hz, C^8), 54.4 (d, J = 32.2 Hz, C^7), 50.0 (C^5), 48.9 (C^5), 17.9 (d, J = 21.5 Hz, C^9).

HRMS (ESI) calc: $[M+H]^+$ ($C_{18}H_{20}{}^{35}ClFNO$) 320.1212; measured: 320.1208 = 1.20 ppm difference.

IR (neat) ν_{max}/ cm^{-1}: 1660, 1495, 1444, 1357, 1197, 1073, 1029.

(3S,4S)-3-chloro-4-fluoro-1-tosylpyrrolidine, 32b

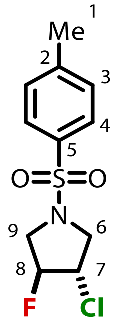

Product **32b** was synthesised from **32a** using ***anti*-chlorofluorination procedure 2** and purified using silica-gel chromatography (25% EtOAc:Hexane) to afford **32b** as a colourless solid (149 mg, 90%, dr >19:1).

R_f = 0.40 (25% EtOAc:Hexane)

^{1}H NMR (500 MHz, $CDCl_3$): δ = 7.70 (2H, d, *J* = 8.1 Hz, H^4), 7.31 (2H, d, *J* = 8.1 Hz, H^3), 4.99 (1H, d, *J* = 50.7, 3.5, 1.3 Hz, H^8), 4.27 (1H, d, *J* = 10.3, 4.4, 1.3 Hz, H^7), 3.80 – 3.55 (4H, m, $H^{6,9}$), 2.41 (3H, s, H^1).

^{19}F NMR (376 MHz, $CDCl_3$): δ = -169.76 – -170.13 (1F, m, F^8).

^{13}C {^{1}H} NMR (125 MHz, $CDCl_3$): δ = 114.1 (C^2), 133.6 (C^5), 129.8 (C^4), 127.6 (C^3), 95.3 (d, *J* = 187.0 Hz, C^8), 57.1 (d, *J* = 28.8 Hz, C^7), 54.1 (C^6), 51.4 (d, *J* = 22.6 Hz, C^9), 21.6 (C^1).

HRMS (APCI) calc: $[M+H]^+$ ($C_{11}H_{14}O_2{}^{35}ClFNS$) 278.0412; measured: 278.0401 = 3.96 ppm difference.

IR (neat) ν_{max}/ cm^{-1}: 1597, 1461, 1343, 1222, 1159, 1091, 1056, 1031, 1005.

(3S,4S)-3-Chloro-4-fluorohexan-1-ol, 33b

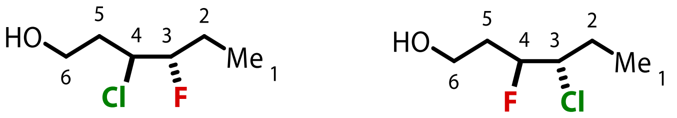

major regioisomer minor regioisomer

Product **33b** was synthesised from (*Z*)-hexen-3-ol using ***anti*-chlorofluorination procedure 2** and purified using silica-gel chromatography (20% EtOAc:Hexane) to afford **33b** as a colourless oil (70 mg, 76%, dr >19:1, rr = 3:1).

R_f = 0.20 (20% EtOAc:Hexane)

^{1}H NMR (500 MHz, $CDCl_3$): Major regioisomer δ = 4.47 (1H, dddd, *J* = 47.0, 8.4, 4.6, 2.9 Hz, H^3), 4.19 (1H, dddd, *J* = 23.3, 9.9, 4.2, 3.0 Hz, H^4), 3.90 – 3.81 (2H, m, H^6), 2.17 – 1.68 (4H, m, $H^{2,5}$), 1.01 (3H, t, *J* = 7.5 Hz, H^1). Minor regioisomer δ = 4.89 – 4.74 (1H, m, H^4), 3.90 – 3.81 (2H, m, $H^{3,6}$), 2.17 – 1.68 (4H, m, $H^{2,5}$), 1.08 (3H, t, *J* = 7.5 Hz, H^1).

^{19}F NMR (376 MHz, $CDCl_3$): Major regioisomer δ = -189.69 (dddd, *J* = 47.0, 30.8, 23.3, 14.2 Hz). Minor regioisomer δ = -191.69 (dddd, *J* = 47.4, 35.9, 21.9, 13.7 Hz)

^{13}C {^{1}H} NMR (125 MHz, $CDCl_3$): Major regioisomer δ = 95.9 (d, *J* = 180.1 Hz, C^3), 59.6 (d, *J* = 21.6 Hz, C^4), 59.3 (C^6), 36.8 (d, *J* = 2.63 Hz, C^5), 25.1 (d, *J* = 21.8 Hz, C^2), 9.5 (d, *J* = 5.8 Hz, C^1). Minor regioisomer δ = 91.7 (d, *J* = 177.2 Hz, C^4), 65.2 (d, *J* = 22.8 Hz, C^3), 58.9 (d, *J* = 4.4 Hz, C^6), 34.7 (d, *J* = 20.6 Hz, C^5), 27.5 (d, *J* = 2.9 Hz, C^2), 11.4 (s, C^1).

HRMS (APCI) calc: $[M-F]^+$ ($C_6H_{12}O^{35}Cl$) 135.0571; measured: 135.0567 = 2.96 ppm difference.

IR (neat) ν_{max}/ cm^{-1}: 3346, 1710, 1461, 1343, 1293, 1182, 1048.

tert-butyl 9-((3R,4R)-3-chloro-4-fluorohexyl)-3,9-diazaspiro[5.5]undecane-3-carboxylate, 34b

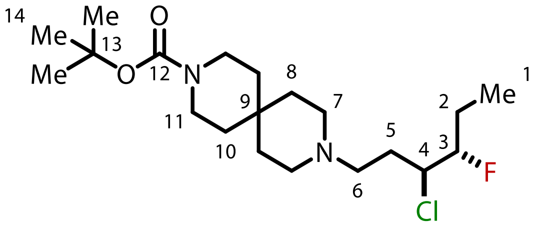

Product **34b** was synthesised from **34a** (202 mg, 0.6 mmol, 1 eq) using ***anti*-chlorofluorination procedure 2** and purified by column chromatography (10-30% IPA in pentane) to afford **34b** as an orange oil (155 mg, 66%, rr >19:1).

^{1}H NMR (500 MHz, CDCl$_3$) δ 4.48 (1H, dddd, J = 47.1, 8.6, 4.3, 3.2 Hz, H^3), 4.09 (1H, m, H^4), 3.38 (4H, dt, J = 5.9, 2.0, H^7), 2.58 (2H, tdd, J = 15.7, 10.4, 5.3 Hz, H^6), 2.45 (4H, m, H^{11}), 2.02 (2H, m, H^5), 1.84 (2H, m, H^2), 1.55 (4H, dt, J = 5.1, 1.7 Hz, H^8) 1.47 (9H, s, H^{14}), 1.43 (4H, m, H^{10}), 1.03 (3H, t, J = 7.5 Hz, H^1).

^{19}F NMR (377 MHz, CDCl$_3$) δ -188.86 (dddd, *J* = 46.8, 31.3, 21.8, 14.9 Hz).

^{13}C {^{1}H} NMR (101 MHz, CDCl$_3$) δ 155.2 (C^{12}), 95.8 (d, *J* = 178.2 Hz, C^3), 79.4 (C^{13}), 64.6 (C^9), 61.2 (d, *J* = 22.1 Hz, C^4), 55.6 (C^6), 49.3 (C^{11}), 39.1 (C^7), 35.5 (C^8), 31.5 (d, *J* = 2.5 Hz, H^5), 29.7 (C^{10}), 28.6 (C^{14}), 24.8 (d, *J* = 21.4 Hz, C^2), 9.6 (d, *J* = 5.5 Hz, C^1).

HRMS (ESI) m/z calc: [M+H$^+$] ($C_{20}H_{36}{}^{35}ClFN_2O_2$) 391.2522, measured = 391.2515, 1.78 ppm difference.

IR (neat) ν_{max}/cm^{-1}: 2978, 2930, 1686, 1428, 1364, 1162.

***3,4,6-tri-O-acetyl-2-deoxy-2-chloro-α-D-mannopyranosyl fluoride,* α-*anti*-35b, and 3,4,6-*tri-O-acetyl-2-deoxy-2-chloro-α-D-glucopyranosyl fluoride,* α-*syn*-35b, and *3,4,6-tri-O-acetyl-2-deoxy-2-chloro-β-D-glucopyranosyl fluoride,* β-*anti*-35b**

3,4,6-tri-*O*-acetyl-D-glucal was subjected to ***anti*-chlorofluorination procedure 1**. Before purification, hexafluorobenzene (23.2 μL) was added and ^{19}F NMR yields of **α-*anti*-35b**, **α-*syn*-35b** and **β-*anti*-35b** were measured as 34%, 20% and 31% respectively. The crude mixture was purified by reverse phase HPLC to afford the three diastereomers in sufficient purity for full characterisation.

***3,4,6-tri-O-acetyl-2-deoxy-2-chloro-α-D-mannopyranosyl fluoride,* α-*anti*-35b**

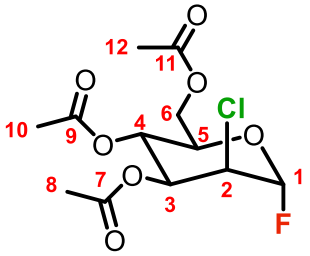

^{1}H NMR (400 MHz, CDCl$_3$) 5.74 (1H, dd, $^2J_{FH}$ = 50.0, $^3J_{HF}$ 1.7 Hz, ***H1***), 5.47 (1H, t, *J* = 10.0 Hz, ***H4***), 5.35 (ddd, *J* = 10.0, 3.8, 1.2 Hz, 1H, ***H3***), 4.54 (dt, *J* = 3.9, 1.9 Hz, 1H, ***H2***), 4.28-4.13 (m, 3H, ***H6***, ***H5***), 2.11 (2xs, 6H, ***H10***, ***H12***), 2.07 (s, 3H, ***H11***).

^{13}C NMR (101 MHz, CDCl$_3$) 170.8 (***C9***), 170.1 (***C7***), 169.5 (***C8***), 106.8 (d, $^1J_{CF}$ = 225.8 Hz, ***C1***), 71.4 (d, $^3J_{CF}$ = 3.2 Hz, ***C5***), 68.8 (***C3***), 64.4 (***C4***), 61.7 (***C6***), 55.5 (d, $^2J_{CF}$ = 37.5 Hz, ***C2***), 20.8 (2xs, **C8** or **C10** or **C12**), 20.7 (**C8** or **C10** or **C12**)

^{19}F NMR (377 MHz, CDCl$_3$) −127.37 (d, $^2J_{HF}$ = 49.9 Hz)

HRMS (MALDI) calc: $[M+Na]^+$ ($C_{12}H_{16}O_7{}^{35}ClF$) 349.0461; measured: 349.0456 = 1.43 ppm difference.

IR (neat) ν_{max}/cm^{-1}: 1741, 1433, 1368, 1219, 1168, 1089, 1066, 1047, 1013, 970, 912, 802, 751

3,4,6-tri-O-acetyl-2-deoxy-2-chloro-α-D-glucopyranosyl fluoride, α-*syn*-35b

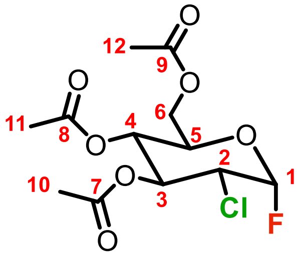

^{1}H NMR (MHz, CDCl$_3$) 5.71 (1H, dd, $^{2}J_{HF}$ = 51.2, $^{3}J_{HH}$ = 2.5 Hz, ***H1***), 5.47 (1H, dd, *J* 10.7, 9.5 Hz, ***H3***), 5.14-5.06 (1H, m, ***H4***), 4.30 (1H, dd, *J* 12.4, 4.3 Hz, ***H6***), 4.24 (1H, ddd, *J* = 10.5, 4.3, 2.0 Hz, ***H5),*** 4.14 (1H, dd, *J* 12.4, 2.0 Hz, ***H6'***), 3.95 (ddd, *J* = 24.3, 10.7, 2.5 Hz, 1H, ***H2***), 2.10 (3H, s), 2.09 (2×s, 6H, ***H10***, ***H12***), 2.05 (3H, s, ***H11***).

^{13}C NMR (MHz, CDCl$_3$) 170.6 (***C9***), 169.9 (***C7***), 169.6 (***C8***), 105.5 (d, $^{1}J_{CF}$ = 231.0 Hz, ***C1***), 71.5 (***C5***), 70.2 (d, $^{3}J_{CF}$ = 4.8 Hz, ***C3***), 68.0 (***C4***), 61.3 (***C6***), 56.1 (d, $^{2}J_{CF}$ = 27.5 Hz, ***C2***), 20.8 (***C10*** or ***C11*** or ***C12***), 20.7 (2×s, ***C10*** or ***C11*** or ***C12***).

^{19}F NMR (MHz, CDCl$_3$) −147.23 (dd, $^{2}J_{HF}$ = 51.3, $^{3}J_{HF}$ = 24.3 Hz).

HRMS (MALDI) calc: [M+Na]$^{+}$ ($C_{12}H_{16}O_{7}{}^{35}ClF$) 349.0461; measured: 349.0455 = 1.72 ppm difference.

IR (neat) ν_{max}/cm^{-1}: 1743, 1434, 1367, 1215, 1151, 1085, 1030, 958, 920, 898, 829, 742, 675

3,4,6-tri-O-acetyl-2-deoxy-2-chloro-β-D-glucopyranosyl fluoride, β-*anti*-35b

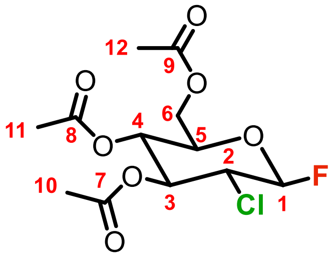

^{1}H NMR (MHz, CDCl$_3$) 5.37 (dd, $^2J_{HF}$ = 50.8, $^3J_{HH}$ 7.5 Hz, 1H, ***H1***), 5.31 (ddd, *J* = 10.1, 9.2, 1.0 Hz, 1H, ***H3***), 5.07 (dd, *J* = 10.0, 9.2 Hz, 1H, ***H4***), 4.29 (ddd, *J* = 12.5, 4.9, 1.0 Hz, 1H, ***H6***), 4.19 (dd, *J* = 12.5, 2.4 Hz, 1H, ***H6'***), 3.91 – 3.81 (m, 2H, ***H2, H5***), 2.10 (2×s, 6H, ***H10, H12***), 2.04 (s, 3H, ***H11***)

^{13}C NMR (MHz, CDCl$_3$) 170.7 (***C9***), 169.9 (***C7***), 169.5 (***C8***), 107.9 (d, *J* = 219.1 Hz, ***C1***), 73.5 (d, $^3J_{CF}$ = 8.8 Hz, ***C3***), 72.2 (d, $^3J_{CF}$ = 5.6 Hz, ***C5***), 68.28 (C4), 61.6 (C6), 57.7 (d, $^2J_{CF}$ = 25.4 Hz, ***C2***), 20.8 (***C10*** or ***C11*** or ***C12***), 20.6 (***C10*** or ***C11*** or ***C12***)

^{19}F NMR (MHz, CDCl$_3$) −137.40 (dd, $^2J_{HF}$ = 50.9, $^3J_{HF}$ = 10.3 Hz).

HRMS (MALDI) calc: $[M+Na]^+$ ($C_{12}H_{16}O_7{}^{35}ClF$) 349.0461; measured: 349.0461 = 0.00 ppm difference.

IR (neat) ν_{max}/cm^{-1}: 1745, 1433, 1367, 1215, 1091, 1045, 962, 904, 819, 734, 667

***(3R,4R)-3-chloro-4-fluorohexyl 3,5-dinitrobenzoate,* 36d**

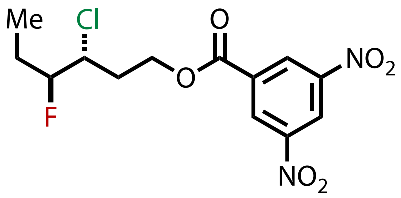

Product **36d** was synthesised from **36a** using ***anti*-chlorofluorination procedure 2** and purified using silica-gel chromatography (15% EtOAc:Hexane) to afford **36d** as a colourless oil (154 mg, 74%, dr >19:1, rr = 6:1).

R_f = 0.35 (30% EtOAc:Hexane)

^{1}H NMR (400 MHz, $CDCl_3$): δ = 9.23 – 9.21 (1H, m, H^{11}), 9.16 – 9.13 (2H, m, H^9), 4.77 – 4.61 (2H, m, H^6), 4.58 – 4.40 (1H, m, H^3), 4.11 – 4.02 (1H, m, H^4), 2.58 – 2.44 (1H, m, H^5), 2.26 – 2.13 (1H, m, H^5), 1.97 – 1.68 (2H, m, H^2), 1.05 (3H, t, J = 6.7 Hz, H^1).

^{19}F NMR (376 MHz, $CDCl_3$): δ = -184.08 (1F, dddd, J = 47.8, 34.0, 19.4, 10.1 Hz, F^3).

^{13}C {^{1}H} NMR (100 MHz, $CDCl_3$): δ = 162.5 (C^7), 148.8 (C^{10}), 133.8 (C^8), 129.6 (C^9), 122.6 (C^{11}), 96.1 (d, J = 177.1 Hz, C^3), 63.7 (C^6), 58.3 (d, J = 22.1 Hz, C^4), 32.3 (d, J = 4.1 Hz, C^5), 25.1 (d, J = 21.6 Hz, C^2), 9.2 (d, J = 3.9 Hz, C^1).

HRMS (EI) calc: $[M]^+$ (348.0519); measured: 348.0520 = 0.29 ppm difference.

IR (neat) ν_{max}/ cm^{-1}: 1730, 1629, 1598, 1451, 1275, 1163, 1075

2-(1-((3R,4R)-3-chloro-4-fluorohexyl)piperidin-4-yl)-4,6-dimethoxypyrimidine, 37b

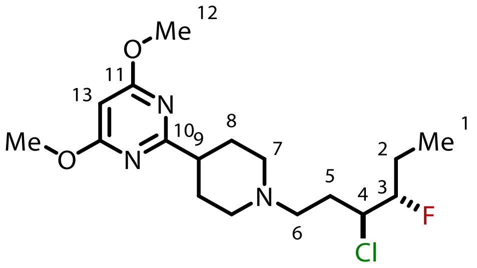

Product **37b** was synthesized from **37a** using ***anti*-chlorofluorination procedure 2** and purified by column chromatography (0-10% IPA in $CHCl_3$) to afford **37b** as an orange oil (143 mg, 66%, rr >19:1).

^{1}H NMR (500 MHz, CDCl3) δ 5.85 (1H, s, H^{13}), 4.47 (1H, dddd, J = 47.1, 8.6, 4.3, 3.2 Hz, H^3), 4.08 (1H, ddt, *J* = 22.0, 9.7, 3.9 Hz, H^4), 3.91 (6H, s, H^{12}), 3.00 (2H, m, H^7), 2.68 (1H, tt, J = 10.1, 4.6 Hz, H^9), 2.59 (2H, m, H^6), 2.21-2.07 (3H, m, H^5, $H^{7'}$), 1.99 (5H, m, $H^{5'}$, H^8, $H^{8'}$), 1.90-1.69 (2H, m, H^2), 1.02 (3H, t, J = 7.5 Hz).

^{13}C {^{1}H} NMR (101 MHz, $CDCl_3$) δ 172.7 (C^{10}), 171.5 (C^{11}), 95.8 (d, *J* = 178.2 Hz, C^3), 86.9 (C^{13}), 61.2 (d, *J* = 21.9 Hz, C^4), 55.6 (C^6), 54.3 (C^5), 53.9 (C^{12}), 53.5 (d, *J* = 17.3 Hz, C^7), 44.7 (C^9), 31.5 (C^8), 24.9 (d, *J* = 21.4 Hz, C^2), 9.6 (d, *J* = 5.6 Hz, C^1).

^{19}F NMR (377 MHz, $CDCl_3$) δ -188.9 (dddd, J = 46.8, 31.5, 21.9, 14.9 Hz).

HRMS (ESI) m/z calc: [M+H$^+$] ($C_{17}H_{27}{}^{35}ClFN_3O_2$) 360.1849, measured = 360.1843, 1.66 ppm difference

IR (neat) ν_{max}/cm^{-1}: 2881, 1379, 1328, 1087, 1045, 879.

4-fluorobenzyl (3R,4R)-3-chloro-4-fluorohexanoate, 38b

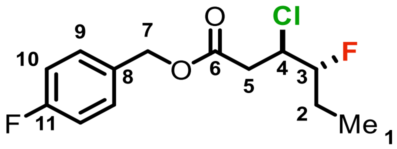

Product **38b** was synthesized from **38a-*cis*** using ***anti*-chlorofluorination procedure 2** on a 0.6 mmol scale, after work-up the residue was dissolved in $CDCl_3$ (2 mL), to the solution was added hexafluorobenzene (23.2 µL, 0.33 eq.) and a ^{19}F NMR spectrum was recorded for the sample, from which a 45% (rr > 19:1) ^{19}F NMR yield was measured. ^{1}H and ^{13}C NMR data were recorded for the reaction mixture and reported below for **38b**.

^{19}F NMR (377 MHz, $CDCl_3$) −190.95 (dddd, *J* 47.1, 30.6, 23.4, 13.8 Hz)

HRMS (EI) calc: $[M]^+$ ($C_{13}H_{15}{}^{35}ClF_2O_2$) 276.0723; measured: 276.0719 = 1.45 ppm difference.

These data are consistent with the assigned *anti*-diastereomer by comparison with chemical shift and coupling constant data of **1b**.

4-fluorobenzyl (3R,4R)-3-chloro-4-fluorohexanoate, 38d

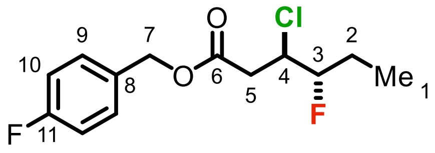

Product **38d** was synthesized from **38a-*trans*** using ***anti*-chlorofluorination procedure 2** on a 0.6 mmol scale, after work-up the residue was dissolved in $CDCl_3$ (2 mL), to the solution was added hexafluorobenzene (23.2 μL, 0.33 eq.) and a ^{19}F NMR spectrum was recorded for the sample, from which a 20% (rr > 19:1) ^{19}F NMR yield was measured. ^{1}H and ^{13}C NMR data were recorded for the reaction mixture and reported below for **38d**.

^{1}H NMR (400 MHz, $CDCl_3$) δ 7.39 – 7.30 (m, 2H, *H9*), 7.05 (app. t, *J* = 8.7 Hz, 2H, *H10*), 5.14 (s, 2H, *H7*), 4.45 (dddd, *J* = 47.4, 8.2, 7.1, 3.1 Hz, 1H, *H3*), 4.32 (tdd, *J* = 9.6, 7.1, 3.9 Hz, 1H), 3.02 (ddd, *J* = 16.4, 3.8, 0.9 Hz, 1H, *H5*), 2.76 (dd, *J* = 16.3, 9.5 Hz, 1H, *H5'*), 1.97 – 1.65 (m, 2H, *H2*), 1.03 (t, *J* = 7.4 Hz, 3H).

^{13}C NMR (101 MHz, $CDCl_3$) δ 169.9 (*C6*), 162.9 (d, *J* = 247.1 Hz, *C11*), 131.5 (d, *J* = 3.2 Hz, *C8*), 130.5 (d, *J* = 8.2 Hz, *C9*), 115.7 (d, *J* = 21.6 Hz, *C10*). 95.8 (d, *J* = 177.4 Hz, *C3*) 66.3 (*C7*), 56.5 (d, *J* = 25.4 Hz, *C4*), 39.3 (d, *J* = 4.7 Hz, *C5*), 25.0 (d, *J* = 21.2 Hz, *C2*), 9.1 (d, *J* = 4.2 Hz, *C1*).

^{19}F NMR (377 MHz, $CDCl_3$) −184.02 (dddd, *J* 47.4, 33.5, 19.9, 9.9 Hz)

HRMS (EI) calc: $[M]^+$ ($C_{13}H_{15}{}^{35}ClF_2O_2$) 276.0723; measured: 276.0719 = 1.45 ppm difference.

6-Chloro-7-fluoro-8-oxabicyclo[3.2.1]octan-3-one, 39b

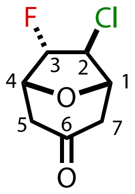

To each compartment of a larger-scale 3D printed divided cell, equipped with magnetic stirrer bars and Nafion™ membranes, was added CH_2Cl_2 (9 mL) and 5.6 HF stock solution (27 mL) were added. To the anodic compartment was added *p*-iodotoluene (1.1 eq., 22 mmol, 6.10 g). The compartments were capped, and platinum electrodes were inserted through each lid, and the mixture was subjected to electrolysis (30.0 mA, 2.2 *F*, 41 hrs). Then, to a 1 L HDPE vessel was added the anodic compartment reaction mixture, and CH_2Cl_2 (250 mL). This vial was capped with a Suba-seal and a venting needle was placed through the Suba-seal. Once the reaction mixture was cooled to -46 ºC, 8-oxabicyclo[3.2.1]oct-6-en-3-one (0.33 м, 21 mmol) in CH_2Cl_2 was added in one portion and tetraethylammonium chloride (1.0 м, 1 eq., 21.6 mmol, 3.56 g) in CH_2Cl_2 was added *via* syringe pump (0.16 eq./h). After stirring overnight, the reaction mixture was quenched with 2 L of cold (0 ºC) saturated aqueous K_2CO_3 solution. This stirred for 1 hour until the aqueous layer measured pH 7. The mixture was extracted into CH_2Cl_2, dried with Na_2SO_4, filtered, and concentrated *in vacuo*. The crude mixture was dissolved in EtOAc (50 mL) and washed with water (3 x 50 mL) to reveal a dark brown crude oil. This was placed in a -20 ºC freezer and after several hours, crystals had formed. These were washed with pentane and dried in *vacuo* to afford **39b** as a dark brown solid (3.10 g, 83%).

$\mathbf{R_f}$ = 0.15 (10% EtOAc:IPA)

^{1}H NMR (400 MHz, CDCl$_3$): 5.36 (dd, *J* = 53.6 Hz, 6.2 Hz, H^3), 5.00 – 4.91 (1H, m, H^4), 4.62 (d, *J* = 6.2 Hz, H^1), 4.19 (d, *J* = 18.3 Hz, H^2), 2.85 – 2.79 (1H, m, H^7), 2.73 – 2.61 (2H, m, H^5), 2.59 – 2.54 (1H, m, H^7).

^{19}F NMR (376 MHz, CDCl$_3$): -184.37 – -184.64 (1F, m, F^3).

^{13}C {^{1}H} NMR (100 MHz, $CDCl_3$): 202.6 (*C^6*), 100.3 (d, *J* = 198.3 Hz, *C^3*), 81.8 (d, *J* = 5.5 Hz, *C^1*), 76.1 (d, *J* = 19.9 Hz, *C^4*), 63.5 (d, *J* = 29.8 Hz, *C^2*), 46.2 (*C^7*), 42.5 (d, *J* = 8.4 Hz, *C^5*).

HRMS (APCI) calc: $[M+H]^+$ ($C_7H_9F^{35}ClO_2$) 179.0270; measured: 179.0273 = 1.68 ppm difference.

IR (neat) ν_{max}/ cm^{-1}: 1714, 1596, 1398, 1349, 1197, 1143, 1095, 1059, 1022

Internal alkene *syn*-chlorofluorination products

N-benzyl-N-((3S,4R)-3-chloro-4-fluorohexyl)-4-(trifluoromethyl)aniline, 16d

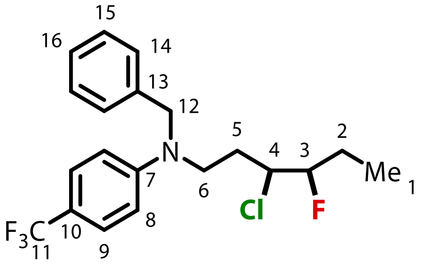

Product **16d** was synthesised **16a** using ***syn-chlorofluorination*** **procedure 3** and purified using silica-gel chromatography (2% EtOAc:Hexane) to afford **16d** as a viscous colourless oil (165 mg, 62%, dr >19:1, rr = 11:1).

R_f = 0.20 (4% EtOAc:Hexane)

^{1}H NMR (500 MHz, $CDCl_3$): δ = 7.45 - 7.41 (5H, m, $H^{14,15,16}$), 7.25 – 7.17 (2H, m, H^9), 6.78 – 6.88 (2H, m, H^8), 4.67 – 4.58 (2H, m, H^{12}), 4.51 – 4.35 (1H, m, H^3), 3.98 – 3.90 (1H, m, H^4), 3.87 – 3.79 (1H, m, H^6), 3.64 – 3.57 (1H, m, H^6), 2.39 – 2.29 (1H, m, H^5), 2.04 – 1.95 (1H, m, H^5), 1.91 – 1.68 (2H, m, H^2), 1.03 (3H, t, J = 7.4 Hz, H^1).

^{19}F NMR (376 MHz, $CDCl_3$): δ = -60.85 (1F, s, F^{11}), -183.38 - -183.90 (1F, m, F^3).

^{13}C {^{1}H} NMR (125 MHz, $CDCl_3$): δ = 150.5 (C^7), 137.7 (C^{13}), 129.0 (C^{14}), 127.4 (C^{16}), 126.8 (q, J = 3.8 Hz, C^9), 126.5 (C^{15}), 125.0 (q, J = 270.1 Hz, C^{11}), 118.3 (q, J = 32.5 Hz, C^{10}), 111.5 (C^8), 96.1 (d, J = 177.1 Hz, C^3), 60.0 (d, J = 26.7 Hz, C^4), 54.5 (C^6), 48.3 (C^{12}), 30.7 (d, J = 3.6 Hz, C^5), 25.0 (d, J = 19.9 Hz, C^2), 9.2 (d, J = 3.9 Hz, C^1).

HRMS (EI) calc: $[M-H]^+$ ($C_{20}H_{21}{}^{35}ClF_4N$) 386.1293; measured: 386.1294 = 0.26 ppm difference.

IR (neat) ν_{max}/ cm^{-1}: 1615, 1530, 1517, 1498, 1328, 1257, 1217, 1118.

N-benzyl-N-((3S,4R)-3-chloro-4-fluorohexyl)-4-iodoaniline, 17d

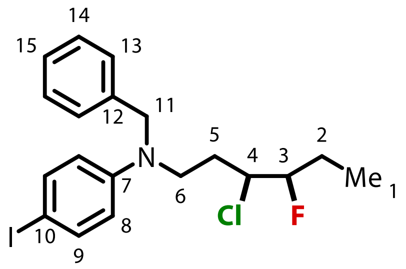

Product **17d** was synthesised **17a** using ***syn*-chlorofluorination procedure 3** and purified using silica-gel chromatography (2% EtOAc:Hexane) to afford **17d** as a viscous colourless oil (183 mg, 62%, dr >19:1, rr = 12:1).

R_f = 0.20 (4% EtOAc:Hexane)

^{1}H NMR (500 MHz, $CDCl_3$): δ = 7.45 – 7.41 (2H, m, H^8), 7.35 – 7.16 (5H, m, $H^{13,14,15}$). 6.51 (2H, d, J = 9.0 Hz, 1H, H^9), 4.54 (2H, d, H^{11}), 4.48 – 4.34 (1H, m, H^3), 3.92 (1H, m, H^4), 3.77 – 3.69 (1H, m, H^6), 3.58 – 3.49 (1H, m, H^6), 2.35 – 2.25 (1H, m, H^5), 1.98 – 1.90 (1H, m, H^5), 1.88 – 1.68 (2H, m, H^2), 1.02 (3H, t, J = 7.4 Hz, H^1).

^{19}F NMR (376 MHz, $CDCl_3$): δ = -183.47 – -183.86 (1F, m, F^3)

^{13}C {^{1}H} NMR (125 MHz, $CDCl_3$): δ = 147.8 (C^{10}), 138.1 (C^{12}), 138.0 (C^8) 128.9 (C^{13}), 127.2 (C^{15}), 126.6 (C^{14}), 114.8 (C^9), 114.8 (C^7), 96.2 (d, J = 175.6 Hz, C^3), 60.1 (d, J = 24.9 Hz, C^4), 54.6 (C^6), 48.3 (C^{11}), 30.7 (d, J = 4.5 Hz, C^5), 25.0 (d, J = 21.2 Hz, C^2), 9.2 (d, J = 4.1 Hz, C^1).

HRMS (APCI) calc: $[M]^+$ ($C_{19}H_{22}{}^{35}ClFIN$) 445.0542; measured: 445.0544 = 0.49 ppm difference.

IR (neat) v_{max}/ cm^{-1}: 1585, 1495, 1452, 1393, 1383, 1329, 1236, 1007.

N-benzyl-N-((3S,4R)-3-chloro-4-fluorohexyl)-4-fluoroaniline, 1d

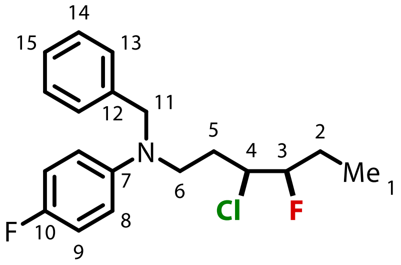

Product **1d** was synthesised **1a-*cis*** using ***syn*-chlorofluorination procedure 3** and purified using silica-gel chromatography (2% EtOAc:Hexane) to afford **1d** as a viscous colourless oil (152 mg, 55%, dr >19:1, rr = 13:1).

1d was also prepared from **1a-*cis*** using ***syn*-chlorofluorination procedure 5** to give **1b** (59% NMR yield, dr > 3.5:1, rr = 14:1). The reaction was repeated, where **1d** was also prepared from **1a-*cis*** using ***syn*-chlorofluorination procedure 5** but a CH_2Cl_2 volume of 0.58 mL was used to afford **1d** (34% NMR yield, dr 1.7:1, rr = 9:1).

R_f = 0.20 (4% EtOAc:Hexane)

^{1}H NMR (400 MHz, $CDCl_3$): δ = 7.35 – 7.27 (2H, m, H^{14}), 7.25 – 7.17 (3H, m, $H^{13,15}$), 6.90 (2H, dd, J = 9.2, 8.3 Hz, H^8), 6.71 – 6.62 (2H, m, H^9), 4.54 – 4.30 (3H, m, $H^{3,11}$), 3.95 (1H, m, H^4), 3.70 (1H, ddd, J = 13.9, 9.1, 4.2 Hz, H^6), 3.51 (1H, ddd, J = 15.2, 8.9, 6.9 Hz, H^6), 2.33 – 2.23 (1H, m, H^5), 1.99 – 1.67 (3H, m, $H^{2,5}$), 1.00 (3H, t, J = 7.4 Hz, H^1).

^{19}F NMR (376 MHz, $CDCl_3$): δ = -128.61 – -128.76 (1F, m, F^{10}), -183.47 – -184.12 (1F, m, F^3).

^{13}C {^{1}H} NMR (100 MHz, $CDCl_3$): δ = 155.9 (d, J = 228.0 Hz, C^{10}), 145.0 (C^7), 138.6 (C^{12}), 128.8 (C^{13}), 127.1 (C^{15}), 126.8 (C^{14}), 115.8 (d, J = 22.3 Hz, C^9), 114.1 (d, J = 6.6 Hz, C^8), 96.1 (d, J = 176.6 Hz, C^3), 60.3 (d, J = 25.6 Hz, C^4), 55.6 (C^{11}), 48.8 (C^6), 30.8 (d, J = 3.4 Hz, C^5), 24.9 (d, J = 22.3 Hz, C^2), 9.2 (d, J = 3.5 Hz, C^1).

HRMS (EI) calc: $[M]^+$ ($C_{19}H_{22}F_2{}^{35}ClN$) 337.4103; measured: 337.1401 = 0.59 ppm difference.

IR (neat) ν_{max}/ cm^{-1}: 1578, 1491, 1445, 1372, 1312, 1278, 1257, 1231, 1006.

***N*-benzyl-*N*-((3S,4R)-3-chloro-4-fluorohexyl)aniline, 18d**

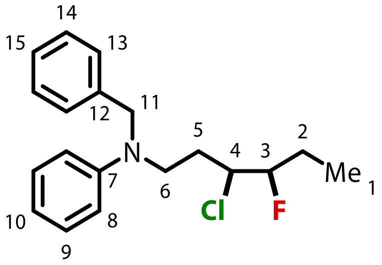

Product **18d** was synthesised from **18a** using ***syn*-chlorofluorination procedure 3** and purified using silica-gel chromatography (2% EtOAc:Hexane) to afford **18d** as a viscous colourless oil (80 mg, 59%, dr >19:1, rr = 16:1).

R_f = 0.25 (4% EtOAc:Hexane)

^{1}H NMR (400 MHz, $CDCl_3$): δ = 7.39 – 7.21 (8H, m, $H^{9,10,13,14,15}$), 6.87 – 6.68 (2H, m, H^8), 4.70 – 4.36 (3H, m, $H^{3,11}$), 4.06 – 3.91 (1H, m, H^4), 3.85 – 3.54 (2H, m, H^6), 2.45 – 2.27 (1H, m, H^5), 2.05 – 1.92 (1H, m, H^5), 1.90 – 1.72 (2H, m, H^2), 1.04 (3H, t, *J* = 6.7 Hz, H^1).

^{19}F NMR (376 MHz, $CDCl_3$): δ = -183.61 (1F, dddd, *J* = 47.7, 32.0, 20.3, 10.5 Hz, F^3).

^{13}C {^{1}H} NMR (100 MHz, $CDCl_3$): δ = 148.4 (C^7), 138.9 (C^{12}), 129.5 (C^8), 128.8 (C^{14}), 127.3 (C^{15}), 126.8 (C^{13}), 116.9 (C^{10}), 112.7 (C^9), 96.4 (d, *J* = 176.6 Hz, C^3), 60.4 (d, *J* = 22.6 Hz, C^4), 54.9 (C^{11}), 48.2 (C^6), 30.7 (C^5), 24.9 (d, *J* = 21.6 Hz, C^2), 9.3 (d, *J* = 4.1 Hz, C^1).

HRMS (EI) calc: $[M]^+$ ($C_{19}H_{23}{}^{35}ClFN$) 319.1498; measured: 319.1495 = 0.94 ppm difference.

IR (neat) ν_{max}/ cm^{-1}: 1597, 1504, 1453, 1378, 1356, 1295, 1237, 1205, 1162, 1074, 1051.

N-benzyl-N-((3S,4R)-3-chloro-4-fluorohexyl)-4-methylaniline, 19d

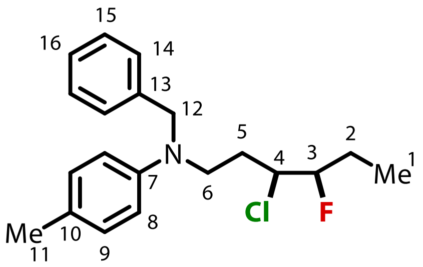

Product **19d** was synthesised from **19a** using ***syn*-chlorofluorination procedure 3** and purified using silica-gel chromatography (2% EtOAc:Hexane) to afford **19d** as a viscous colourless oil (87 mg, 44%, dr >19:1, rr = 14:1).

R_f = 0.25 (EtOAc:Hexane)

^{1}H NMR (500 MHz, $CDCl_3$): δ = 7.41 – 7.34 (2H, m, H^{14}), 7.32 – 7.27 (3H, m, $H^{15,16}$), 7.11 – 7.05 (2H, m, H^9), 6.79 – 6.68 (2H, m, H^8), 4.64 – 4.55 (2H, m, H^{12}), 4.53 – 4.39 (1H, m, H^3), 4.07 – 3.97 (1H, m, H^4), 3.82 – 3.70 (1H, m, H^6), 3.63 – 3.54 (1H, m, H^6), 2.41 – 2.28 (4H, m, $H^{5,11}$), 2.03 – 1.94 (1H, m, H^5), 1.92 – 1.71 (2H, m, H^2), 1.07 (3H, t, J = 6.7 Hz, H^1).

^{19}F NMR (376 MHz, $CDCl_3$): δ = -183.62 (dddd, J = 47.7, 31.9, 20.8, 10.8 Hz).

^{13}C {^{1}H} NMR (125 MHz, $CDCl_3$): δ = 146.2 (C^{10}), 139.0 (C^{13}), 130.0 (C^9), 128.7 (C^{14}), 126.9 (C^{16}), 126.8 (C^{15}), 126.0 (C^7), 96.2 (d, J = 183.1 Hz, C^3), 60.4 (d, J = 22.3 Hz, C^4), 55.1 (C^{12}), 48.4 (C^6), 30.8 (d, J = 3.6 Hz, C^5), 24.9 (d, J = 22.3 Hz, C^2), 20.3 (C^{11}), 9.3 (d, J = 4.1 Hz, C^1).

HRMS (EI) calc: $[M+H]^+$ ($C_{20}H_{26}{}^{35}ClFN$) 334.1732; measured: 334.1739 = 2.09 ppm difference.

IR (neat) $ν_{max}$/ cm^{-1}: 1655, 1381, 1312, 1305, 1272, 1268, 1172, 1118, 1048, 1030.

2-((3S,4R)-3-Chloro-4-fluorohexyl)isoindoline, 40d

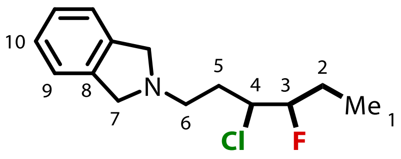

Product **40d** was synthesised from **40a-*cis*** using ***syn*-chlorofluorination procedure 3** (Py•9HF (2.1 mL) was added after electrolysis) and purified using silica-gel chromatography (5% IPA:EtOAc) to afford **40d** as a viscous brown oil (107 mg, 70%, dr = 15:1, rr = 14:1).

R_f = 0.40 (5% IPA:EtOAc)

^{1}H NMR (400 MHz, CDCl3): δ = 7.20 (4H, s, $H^{9,10}$), 4.54 – 4.40 (1H, m, H^3), 4.19 – 4.11 (1H, m, H^4), 4.02 – 3.93 (4H, m, H^7), 3.02 (1H, m, H^6), 2.94 (1H, ddd, *J* = 12.0, 7.7, 4.2 Hz, H^6), 2.30 – 2.22 (1H, m, H^5), 1.94 – 1.70 (3H, m, $H^{2,5}$), 1.04 (3H, t, *J* = 7.4 Hz, H^1).

^{19}F NMR (376 MHz, $CDCl_3$): δ = -184.11 – -184.58 (1F, m, F^3).

^{13}C {^{1}H} NMR (100 MHz, $CDCl_3$): δ = 139.8 (C^8), 127.0 (C^{10}), 122.4 (C^9), 96.6 (d, *J* = 176.5 Hz, C^3), 60.2 (d, *J* = 24.5, Hz, C^4), 59.2 (C^7), 52.5 (C^6), 32.5 (d, *J* = 4.1 Hz, C^5), 25.1 (d, *J* = 21.4 Hz, C^2), 9.4 (d, *J* = 4.1 Hz, C^1).

HRMS (ESI) calc: $[M+H]^+$ ($C_{14}H_{20}{}^{35}ClFN$) 256.1263; measured: 256.1263 = 0.20 ppm difference.

IR (neat) ν_{max}/ cm^{-1}: 1693, 1494, 1447, 1416, 1349, 1273, 1122, 1073.

3-(1-((3S,4R)-3-chloro-4-fluorohexyl)piperidin-4-yl)-6-fluorobenzo[d]isoxazole, 41d

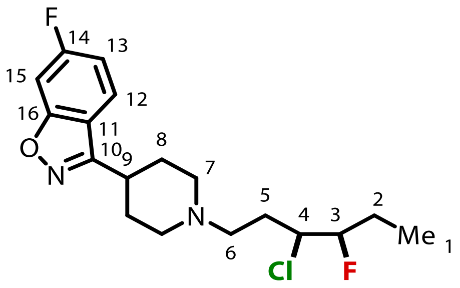

Product **41d** was synthesised from **41a** using ***syn*-chlorofluorination procedure 3** (Py•9HF (2.1 mL) was added after electrolysis) and purified using silica-gel chromatography (30% EtOAc:Hexane) to afford **41d** as a viscous colourless oil (120 mg, 56%, dr and rr >19:1).

R_f = 0.25 (30% EtOAc:Hexane)

^{1}H NMR (400 MHz, $CDCl_3$): δ = 7.70 (1H, dd, *J* = 8.7, 5.1 Hz, H^{12}), 7.24 (1H, dd, *J* = 8.5, 2.1 Hz, H^{15}), 7.05 (1H, td, *J* = 8.8, 2.2 Hz, H^{13}), 4.56 – 4.37 (1H, m, H^{3}), 4.14 – 4.03 (1H, m, $H^{4,9}$), 3.14 – 3.02 (3H, m, $H^{6,9}$), 2.69 – 2.58 (2H, m, H^{7}), 2.34 – 2.03 (7H, m, $H^{5,7,8}$), 1.92 – 1.72 (3H, m, $H^{2,5}$), 1.04 (3H, t, *J* = 7.6 Hz, H^{1}).

^{19}F NMR (376 MHz, $CDCl_3$): δ = -109.54 – -109.74 (1F, m, F^{14}), -183.89 – -184.52 (1F, m, F^{3}).

^{13}C {^{1}H} NMR (100 MHz, $CDCl_3$): δ = 164.2 (d, *J* = 250.9 Hz, C^{14}), 164.0 (d, *J* = 11.1 Hz, C^{16}), 161.2 (C^{11}), 122.7 (d, *J* = 12.5 Hz, C^{12}), 117.4 (C^{10}), 112.5 (d, *J* = 24.5 Hz, C^{13}), 97.6 (d, *J* = 21.6 Hz, C^{15}), 96.6 (d, *J* = 176.6 Hz, C^{3}), 60.7 (d, *J* = 24.4 Hz, C^{4}), 55.2 (C^{6}), 54.4 (C^{9}), 53.1 (C^{7}), 34.6 (C^{8}), 30.7 (C^{5}), 24.9 (d, *J* = 20.7 Hz, C^{2}), 9.4 (d, *J* = 4.3 Hz, C^{1}).

HRMS (APCI) calc: $[M+H]^+$ ($C_{18}H_{24}{}^{35}ClF_2N_2O$) 357.1540; measured: 357.1535 = 1.40 ppm difference.

IR (neat) ν_{max}/ cm^{-1}: 1615, 1494, 1447, 1416, 1349, 1273, 1122.

2-(1-((3R,4S)-3-chloro-4-fluorohexyl)piperidin-4-yl)-4,6-dimethoxypyrimidine, 37d

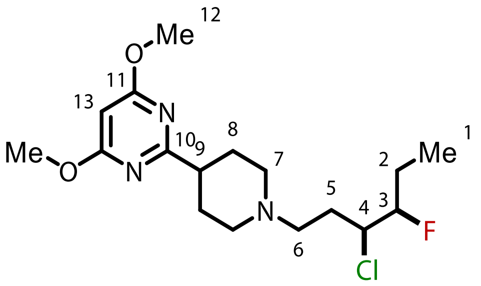

Product **37d** was synthesized from **37a** using ***syn*-chlorofluorination procedure 3** and purified by column chromatography (0-10% IPA in $CHCl_3$) to afford **37d** as an orange oil (69 mg, 32%, dr 3.8:1, rr 1.0:0).

^{1}H NMR (500 MHz, $CDCl_3$) δ 5.83 (1H, s, H^{13}), 4.54-4.37 (1H, m, H^3), 4.15-4.00 (1H, m, H^4), 3.91 (6H, s, H^{12}), 2.99 (2H, t, J = 10.7 Hz, H^7), 2.66 (1H, tt, J = 10.8, 4.7 Hz, H^9), 2.57 (2H, m, H^6), 2.10 (3H, m, $H^{7'}$, $H^{8'}$), 1.97 (4H, m, H^5, H^8, $H^{8'}$), 1.81 (3H, m H^2, $H^{5'}$), 1.03 (3H, t, J = 7.5 Hz, H^1).

^{19}F NMR (377 MHz, $CDCl_3$) δ (major regioisomer) -184.2 (1F, dddd, J = 47.6, 32.3, 21.1, 11.7 Hz), (minor regioisomer) -188.9 (1F, dddd, J = 46.9, 31.5, 22.0, 14.8 Hz).

^{13}C {^{1}H} NMR (101 MHz, $CDCl_3$) δ 172.7 (C^{10}), 171.5 (C^{11}), 97.3 - 95.9 (d, J = 176.6, C^3), 86.9 (C^{13}), 61.1-60.9 (d, J = 24.1, C^4), 55.4 (C^6), 54.5 (C^5), 53.9 (C^{12}), 53.3 (C^7), 44.9 (C^9), 30.8 (C^8), 24.8 (d, J = 21.4 Hz, C^2), 9.4 (d, J = 4.1 Hz, C^1).

HRMS (EZ) m/z calc: [M-Me$^+$] ($C_{17}H_{27}{}^{35}ClFN_3O_2$) 344.1536, measured = 344.1536, 0.00 ppm difference.

IR (neat) v_{max}/cm^{-1}: 2880, 1579, 1461, 1374, 1213, 1163.

4-((3S,4R)-3-Chloro-4-fluorohexyl)morpholine, 42d

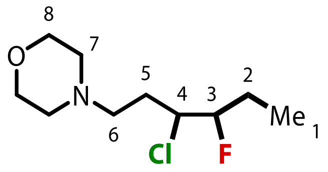

Product **42d** was synthesised from **42a** using ***syn*-chlorofluorination procedure 3** (Py•9HF (2.1 mL) was added after electrolysis) and purified using silica-gel chromatography (1% IPA:EtOAc) to afford **42d** as a viscous oil (84 mg, 63%, dr = 12:1, rr = 14:1).

R_f = 0.35 (1% IPA:EtOAc)

^{1}H NMR (400 MHz, $CDCl_3$): δ = 4.52 – 4.35 (1H, m, H^3), 4.10 – 4.02 (1H, m, H^4), 3.76 – 3.66 (4H, m, H^7), 2.58 – 2.38 (6H, m, $H^{6,8}$), 2.20 – 2.10 (1H, m, H^5), 1.94 – 1.68 (3H, m, $H^{2,5}$), 1.03 (3H, t, J = 7.81 Hz, H^1).

^{19}F NMR (376 MHz, $CDCl_3$): δ = -183.83 – -184.28 (1F, m, F^3).

^{13}C {^{1}H} NMR (100 MHz, $CDCl_3$): δ = 96.4 (d, J = 176.5 Hz, C^3), 67.1 (C^7), 60.4 (d, J = 24.6 Hz, C^4), 55.3 (C^6), 53.9 (C^8), 30.4 (d, J = 4.2 Hz, C^5), 24.9 (d, J = 21.4 Hz, C^2), 9.3 (d, J = 4.2 Hz, C^1).

HRMS (EI) calc: $[M-H]^+$ ($C_{10}H_{18}{}^{35}ClFNO$) 222.1055; measured: 222.1056 = 0.45 ppm difference.

IR (neat) ν_{max}/ cm^{-1}: 1750, 1407, 1350, 1233, 1116

1-((3S,4R)-3-Chloro-4-fluorohexyl)-4-(4-nitrophenyl)piperazine, 43d

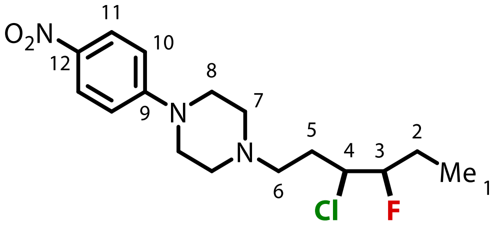

Product **43d** was synthesised from **43a** using ***syn*-chlorofluorination procedure 3** (Py•9HF (2.1 mL) was added after electrolysis) and purified using silica-gel chromatography (4% IPA:EtOAc) to afford **43d** as a viscous yellow oil (132 mg, 64%, dr and rr >19:1).

R_f = 0.40 (4% IPA:EtOAc)

^{1}H NMR (400 MHz, CDCl$_3$): δ = 8.15 – 8.10 (2H, m, H^{10}), 6.85 – 6.79 (2H, m, H^{11}), 4.45 (1H, dddd, J = 47.6, 8.1, 6.6, 3.3 Hz, H^3), 4.12 – 4.03 (1H, m, H^4), 3.48 – 3.37 (4H, m, H^8), 2.69 – 2.53 (6H, m, $H^{6,7}$), 2.25 – 2.14 (1H, m, H^5), 1.96 – 1.70 (3H, m, $H^{2,5}$), 1.04 (3H, t, J = 6.7 Hz, H^1).

^{19}F NMR (376 MHz, CDCl$_3$): δ = -183.69 – -184.04 (1F, m, F^3).

^{13}C {^{1}H} NMR (100 MHz, CDCl$_3$): δ = 154.9 (C^{12}), 138.5 (C^9), 126.0 (C^{11}), 112.7 (C^{10}), 95.8 (d, J = 170.6 Hz, C^3), 60.7 (d, J = 22.4 Hz, C^4), 54.9 (C^6), 52.8 (C^7), 47.1 (C^8), 31.3 (d, J = 2.6 Hz, C^5), 24.8 (d, J = 21.4 Hz, C^2), 9.6 (d, J = 4.5 Hz, C^1).

HRMS (APCI) calc: $[M+H]^+$ ($C_{16}H_{24}N_3O_2{}^{35}ClF$) 344.1536; measured: 344.1522 = 4.07 ppm difference.

IR (neat) ν_{max}/ cm^{-1}: 1595, 1459, 1326, 1243, 1203, 1114.

(1R,5S)-8-((3R,4R)-3-chloro-4-fluorohexyl)-8-azabicyclo[3.2.1]octan-3-one, 44d

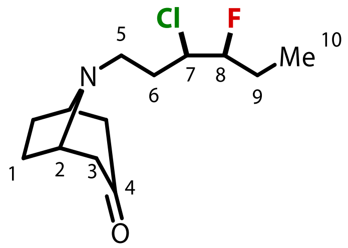

Product **44d** was synthesised from **44a** using ***syn*-chlorofluorination procedure 3** (Py•9HF (2.1 mL) was added after electrolysis) and purified using silica-gel chromatography (25% EtOAc:Hexane) to afford **44d** as a viscous colourless oil (90 mg, 57%, dr and rr >19:1).

R_f = 0.25 (25% EtOAc:Hexane)

^{1}H NMR (500 MHz, $CDCl_3$): 4.57 – 4.38 (1H, m, H^8), 4.28 – 4.11 (1H, m, H^7), 3.55 – 3.46 (2H, m, H^2), 2.88 – 2.79 (1H, m, H^5), 2.78 – 2.56 (3H, m, $H^{3,5}$), 2.24 – 2.11 (3H, m, $H^{3,6}$), 2.07 – 1.99 (2H, m, H^1), 1.93 – 1.72 (3H, m, $H^{6,9}$), 1.64 – 1.54 (2H, m, H^1), 1.04 (3H, t, J = 7.3 Hz, H^{10}).

^{19}F NMR (376 MHz, $CDCl_3$): -183.93 – -184.28 (1F, m, F^8).

^{13}C {^{1}H} NMR (125 MHz, $CDCl_3$): 210.0 (C^4), 96.6 (d, J = 179.6 Hz, C^8), 60.4 (d, J = 25.3 Hz, C^7), 59.2 (C^2), 58.9 (C^2), 48.0 (C^3), 47.7 (C^3), 47.1 (C^5), 32.8 (d, J = 4.1 Hz, C^6), 28.3 (C^1), 27.8 (C^1), 25.0 (d, J = 22.1 Hz, C^9), 9.3 (d, J = 4.3 Hz, C^{10}).

HRMS (APCI) calc: $[M+H]^+$ ($C_{13}H_{22}{}^{35}ClFNO$) 262.1368; measured: 262.1371 = 1.14 ppm difference.

IR (neat) ν_{max}/ cm^{-1}: 1459, 1358, 1300, 1274, 1176, 1069, 1034.

N,N-dibenzyl-3-chloro-4-fluorohexan-1-amine, 26b

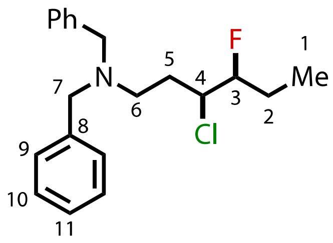

Product **26b** was synthesised from **26a-*trans*** using ***syn*-chlorofluorination procedure 3** (Py•9HF (2.1 mL) was added after electrolysis) and purified using silica-gel chromatography (2% EtOAc:Hexane) to afford **26b** as a viscous colourless oil (84 mg, 42%, dr and rr = 14:1).

R_f = 0.15 (2% EtOAc:Hexane)

^{1}H NMR (500 MHz, $CDCl_3$): 7.37 – 7.23 (10H, m, H^{Bn}), 4.23 – 4.00 (2H, m, $H^{3,4}$), 3.57 (4H, s, H^7), 2.71 – 2.57 (2H, m, H^6), 2.05 – 1.60 (4H, m, $H^{2,5}$), 0.93 (3H, t, *J* = 6.7 Hz, H^1).

^{19}F NMR (376 MHz, $CDCl_3$): -191.15 (dddd, *J* = 46.5, 31.6, 24.3, 13.5 Hz)

^{13}C {^{1}H} NMR (125 MHz, $CDCl_3$): 139.5 (C^8), 129.0 (C^9), 128.4 (C^{10}), 127.2 (C^{11}), 95.1 (d, *J* = 178.0 Hz, C^3), 60.7 (d, *J* = 21.5 Hz, C^4), 58.9 (C^7), 50.5 (C^6), 32.3 (d, *J* = 3.3 Hz, C^5), 25.1 (d, *J* = 20.3 Hz, C^2), 9.6 (d, *J* = 5.8 Hz, C^1).if

HRMS (APCI) calc: $(M+H)^+$ ($C_{20}H_{26}{}^{35}ClFN$) 334.1732; measured: 334.1720 = 3.59 ppm difference.

IR (neat) ν_{max}/ cm^{-1}: 1602, 1494, 1453, 1377, 1119, 1071, 1028.

(3R,4R)-N,N-dibenzyl-3-chloro-4-fluorooctan-1-amine, 45b

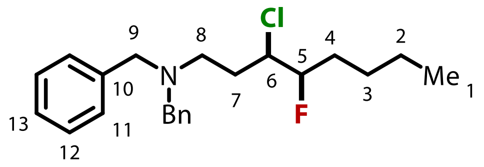

Product **45b** was synthesised from **45a** using ***syn*-chlorofluorination procedure 3** (Py•9HF (2.1 mL) was added after electrolysis) and purified using silica-gel chromatography (2% EtOAc:Hexane) to afford **45b** as a colourless oil (95 mg, 44%, dr and rr >19:1).

R_f = 0.15 (2% EtOAc:Hexane)

^{1}H NMR (400 MHz, $CDCl_3$): 7.41 – 7.22 (10H, m, $H^{11,12,13}$), 4.36 – 4.18 (1H, m, H^5), 4.13 – 4.10 (1H, m, H^6), 3.59 (4H, s, H^9), 2.75 – 2.59 (2H, m, H^8), 2.07 – 1.89 (2H, m, H^7), 1.83 – 1.69 (1H, m, H^4), 1.64 – 1.25 (5H, m, $H^{2,3,4}$), 0.93 (3H, t, J = 6.7 Hz, H^1).

^{19}F NMR (376 MHz, $CDCl_3$): -189.49 – -189.93 (1F, m, F^5).

^{13}C {^{1}H} NMR (100 MHz, $CDCl_3$): 139.5 (C^{10}), 129.0 ($C^{11\text{ or }12}$), 128.4 ($C^{11\text{ or }12}$), 127.2 (C^{13}), 93.9 (d, J = 177.5 Hz, C^5), 61.1 (d, J = 21.1 Hz, C^6), 58.8 (C^9), 50.5 (C^8), 31.8 (d, J = 3.1 Hz, C^7), 31.7 (d, J = 20.5 Hz, C^4), 24.9 (d, J = 4.2 Hz, C^3), 22.6 (C^2), 14.1 (C^1).

HRMS (APCI) calc: $[M+H]^+$ ($C_{22}H_{30}{}^{35}ClFN$) 362.2045; measured: 362.2030 = 4.14 ppm difference.

IR (neat) v_{max}/ cm^{-1}: 1689, 1615, 1530, 1453, 1404, 1394, 1328, 1197, 1056.

(3R,4R)-N,N-dibenzyl-3-chloro-4-fluorononan-1-amine, 46b

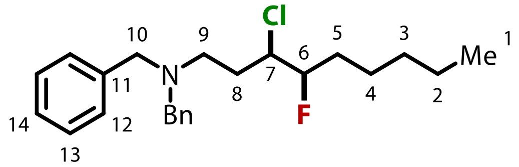

Product **46b** was synthesised from **46a** using ***syn*-chlorofluorination procedure 3** (Py•9HF (2.1 mL) was added after electrolysis) and purified using silica-gel chromatography (4% EtOAc:Hexane) to afford **46b** a colourless oil (97 mg, 43%, dr and rr >19:1).

R_f = 0.15 (2% EtOAc:Hexane)

^{1}H NMR (400 MHz, $CDCl_3$): 7.37 – 7.23 (10H, m, $H^{12,13,14}$), 4.37 – 4.18 (1H, m, H^6), 4.12 – 3.99 (1H, m, H^5), 3.58 (4H, s, H^{10}), 2.74 – 2.58 (2H, m, H^9), 2.08 – 1.89 (2H, m, H^8), 1.82 – 1.22 (8H, m, $H^{2,3,4,5}$), 0.94 (3H, t, J = 6.7 Hz, H^1).

^{19}F NMR (376 MHz, $CDCl_3$): -189.46 – -189.95 (1F, m, F^6).

^{13}C {^{1}H} NMR (100 MHz, $CDCl_3$): 139.5 (C^{11}), 129.0 (C^{13}), 128.4 (C^{12}), 127.2 (C^{14}), 94.0 (d, J = 177.9 Hz, C^6), 61.1 (d, J = 22.1 Hz, C^7), 58.9 (C^{10}), 50.5 (C^9), 32.2 (d, J = 2.1 Hz, C^8), 31.5 (d, J = 20.7 Hz, C^5), 28.9 (C^3), 27.3 (d, J = 4.4 Hz, C^4), 22.6 (C^2), 14.1 (C^1).

HRMS (APCI) calc: $[M+H]^+$ ($C_{23}H_{32}N^{35}ClF$) 376.2202; measured: 376.2186 = 4.25 ppm difference.

IR (neat) ν_{max}/ cm^{-1}: 1512, 1494, 1453, 1366, 1312, 1124, 1071.

(E)-N-((3S,4S)-3-chloro-4-fluorohexyl)-N-(4-fluorobenzyl)hex-3-en-1-amine, 47b

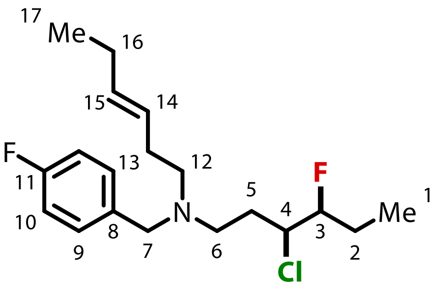

Product **47b** was synthesised from **47a** using ***syn*-chlorofluorination procedure 3** (Py•9HF (2.1 mL) was added after electrolysis) and purified using silica-gel chromatography (10% EtOAc:Hexane) to afford **47b** as a viscous colourless oil (154 mg, 39%, dr = 16:1, rr = 12:1).

R_f = 0.30 (10% EtOAc:Hexane)

^{1}H NMR (500 MHz, $CDCl_3$): δ = 7.34 – 7.28 (2H, m, H^{10}), 7.04 – 6.96 (2H, m, H^9), 5.47 – 5.38 (1H, m, H^{14}), 5.32 – 5.22 (1H, m, H^{15}), 4.42 – 4.26 (1H, m, H^3), 4.12 – 4.02 (1H, m, H^4), 3.67 – 3.51 (2H, m, H^7), 2.81 – 2.63 (2H, m, H^6), 2.57 – 2.44 (2H, m, H^{12}), 2.30 – 2.19 (2H, m, H^5), 2.07 – 1.61 (6H, m, $H^{2,13,16}$), 1.01 – 0.89 (6H, m, $H^{1,17}$).

^{19}F NMR (376 MHz, $CDCl_3$): δ = -114.41 – -117.06 (1F, m, F^{11}), -188.28 – -191.91 (1F, m, F^3).

^{13}C {^{1}H} NMR (125 MHz, $CDCl_3$): δ = 162.1 (d, J = 234.1 Hz, C^{11}), 133.4 (C^{14}), 130.7 (C^9), 130.5 (C^8), 126.3 (C^{15}), 115.3 (d, J = 19.6 Hz, C^{10}), 95.6 (d, J = 178.2 Hz, C^3), 60.7 (d, J = 22.3 Hz, C^4), 58.0 (C^7), 53.6 (C^{12}), 50.4 (C^6), 31.9 (C^5), 25.0 (d, J = 20.6 Hz, C^2), 24.6 (C^{13}), 20.8 (C^{16}), 14.4 (C^{17}), 9.6 (d, J = 6.1 Hz, C^1).

HRMS (APCI) calc: $[M+H]^+$ ($C_{19}H_{29}{}^{35}ClF_2N$) 344.1951; measured: 344.1938 = 3.78 ppm difference.

IR (neat) ν_{max}/ cm^{-1}: 1603, 1508, 1460, 1370, 1222, 1153, 1090, 1015.

2-((3S,4S)-3-Chloro-4-fluorohexyl)isoindoline, 40b

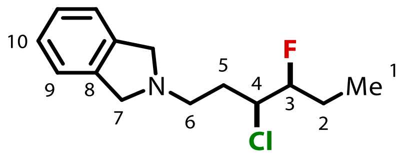

Product **40b** was synthesised from **40a-*trans*** using ***syn*-chlorofluorination procedure 3** (Py•9HF (2.1 mL) was added after electrolysis) and purified using silica-gel chromatography (5% IPA:EtOAc) to afford **40b** as a viscous brown oil (55 mg, 38%, dr >19:1. rr = 17:1).

R_f = 0.40 (5% IPA:EtOAc)

^{1}H NMR (500 MHz, CDCl$_3$): δ = 7.20 (4H, s, $H^{9,10}$), 4.57 – 4.43 (1H, m, H^3), 4.25 – 4.15 (1H, m, H^4), 4.00 – 3.90 (4H, m, H^7), 2.99 (1H, m, H^6), 2.92 (1H, ddd, *J* = 11.9, 7.0, 5.0 Hz, H^6), 2.17 – 2.00 (2H, m, H^5), 1.93 – 1.68 (2H, m, H^2), 1.03 (3H, t, *J* = 7.5 Hz, H^1).

^{19}F NMR (376 MHz, CDCl$_3$): δ = -189.17 – -189.58 (1F, m, F^3).

^{13}C {^{1}H} NMR (125 MHz, CDCl$_3$): δ = 140.0 (C^8), 126.9 (C^{10}), 122.4 (C^9), 95.9 (d, *J* = 178.3 Hz, C^3), 60.7 (d, *J* = 21.9 Hz, C^4), 59.2 (C^6), 52.7 (C^7), 33.4 (d, *J* = 2.5 Hz, C^5), 25.0 (d, *J* = 21.4 Hz, C^2), 9.7 (d, *J* = 5.5 Hz, C^1).

HRMS (EI) calc: [M-H] + ($C_{14}H_{18}{}^{35}ClFN$) 254.1106; measured: 254.1108 = 0.79 ppm difference.

IR (neat) ν_{max}/ cm^{-1}: 1687, 1453, 1406, 1393, 1382, 1251, 1081, 1073.

4-fluorobenzyl (3R,4S)-3-chloro-4-fluorohexanoate, 38d

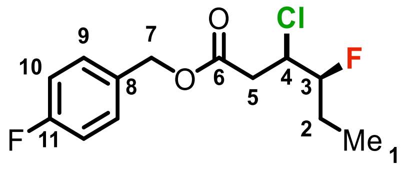

Product **38d** was synthesized from **38a-*cis*** using ***syn*-chlorofluorination procedure 2** on a 0.6 mmol scale, after work-up the residue was dissolved in $CDCl_3$ (2 mL), to the solution was added hexafluorobenzene (23.2 μL, 0.33 eq.) and a ^{19}F NMR spectrum was recorded for the sample, from which a 28% (rr >19:1) ^{19}F NMR yield was measured. ^{1}H and ^{13}C NMR data were recorded for the reaction mixture and reported below for **38d**.

^{19}F NMR (377 MHz, $CDCl_3$) −184.07 (dddd, *J* 47.0, 33.5, 20.2, 10.1 Hz)

HRMS (EI) calc: $[M]^+$ ($C_{13}H_{15}{}^{35}ClF_2O_2$) 276.0723; measured: 276.0719 = 1.45 ppm difference.

These data are consistent with the data recorded for **38d** from ***anti*-chlorofluorination procedure 2** of **38a-*trans***.

X-ray crystallography

X-ray diffraction experiments on **32b** were carried out at 100(2) K on a Bruker D8 Venture diffractometer using Mo-K_{α} radiation (λ = 0.71073 Å). Data collections were performed using a Bruker CPAD detector. Intensities were integrated in SAINT[10] and absorption corrections based on equivalent reflections were applied using SADABS.[11] The structure was solved using ShelXT[12] and refined by full matrix least squares against F^2 in ShelXL[1314] using Olex2.[15] All the non-hydrogen atoms were refined anisotropically. While all the hydrogen atoms were located geometrically apart from the protons on C7 which were disordered and have been modelled in two positions using AFIX 123. Each component has a constrained occupancy of 0.5. All hydrogen atoms were refined using a riding model. The substituted pyrrole group is disordered and has been modelled in two positions with a refined occupancy ratio of 0.845:0.155(3). Equivalent atom distances in the major and minor component (e.g., C9-Cl1 and C9A-Cl1A) have been restrained to be the same (SADI) and due to the proximity equivalent atoms in the major and minor component have been restrained to have the same ADP (SIMU). Crystal structure and refinement data are given in **Table S6**. Crystallographic data for this compound has been deposited with the Cambridge Crystallographic Data Centre as supplementary publication CCDC 2235848. Copies of the data can be obtained free of charge on application to CCDC, 12 Union Road, Cambridge, CB2 1EZ, UK [fax (+44) 1223 336033, e-mail: deposit@ccdc.cam.ac.uk].

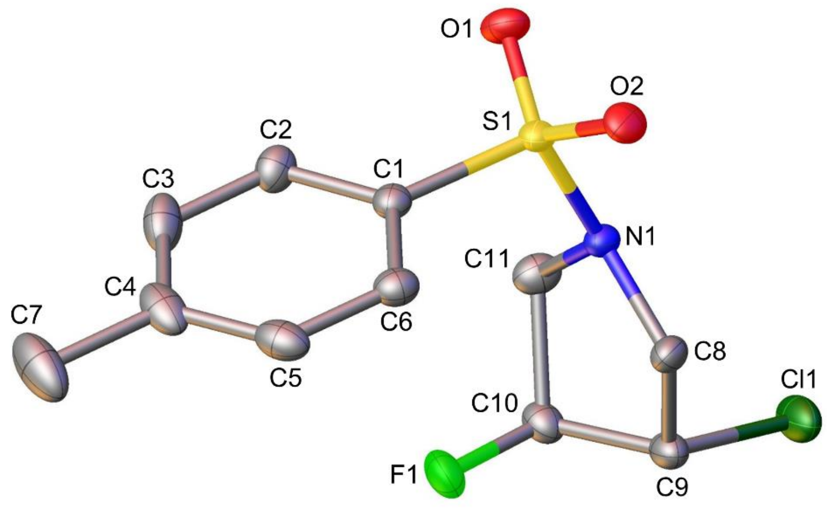

Figure S1 Crystal structure of 32b with the anisotropic displacement parameters depicted at the 50% probability level. Disorder and hydrogens omitted for clarity.

Table S6 Crystal data and structure refinement for 32b.

CCDC number	2235848
Empirical formula	$C_{11}H_{13}ClFNO_2S$
Formula weight	277.73
Temperature/K	100.0
Crystal system	monoclinic
Space group	$P2_1/c$
a/Å	9.7517(4)
b/Å	16.9976(8)
c/Å	7.8143(3)
α/°	90
β/°	110.7500(10)
γ/°	90
Volume/Å^3	1211.25(9)
Z	4
ρ_{calc}g/cm^3	1.523
μ/mm^{-1}	0.489
F (000)	576.0
Crystal size/mm^3	0.346 × 0.22 × 0.1
Radiation	MoKα (λ = 0.71073)
2Θ range for data collection/°	4.466 to 54.968
Index ranges	-12 ≤ h ≤ 12, -22 ≤ k ≤ 22, -10 ≤ l ≤ 10
Reflections collected	53497
Independent reflections	2773 [R_{int} = 0.0382, R_{sigma} = 0.0156]
Data/restraints/parameters	2773/212/218
Goodness-of-fit on F^2	1.113
Final R indexes [I>=2σ (I)]	R_1 = 0.0304, wR_2 = 0.0738
Final R indexes [all data]	R_1 = 0.0352, wR_2 = 0.0761
Largest diff. peak/hole / e Å^{-3}	0.36/-0.39

Benchmarking experiments

Comparison against current state of the art methods

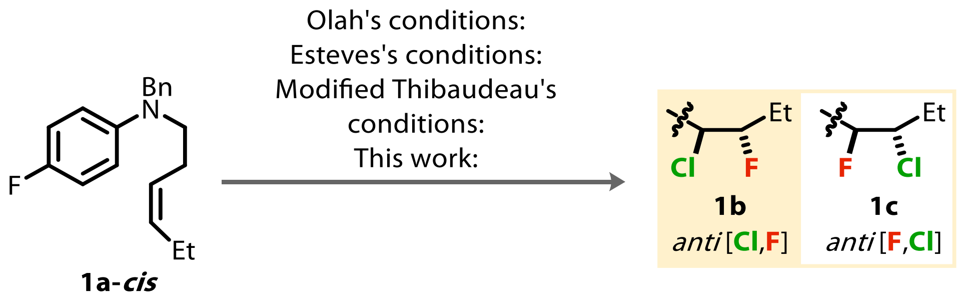

Olah's NCS conditions[16]:

To a 100 mL HDPE vial was added, py•9HF (1 mL) and NCS (2.0 eq., 0.4 mmol, 27 mg). To this solution was added alkene (0.04 M, 1.0 eq., 0.2 mmol) in tetrahydrofuran (5 mL) over 10 minutes. After stirring overnight, the reaction mixture was quenched with 100 mL of cold (0 ºC) saturated aqueous $NaHCO_3$ solution. This stirred for 1 hour until the aqueous layer measured pH 7. The mixture was extracted into CH_2Cl_2, dried with Na_2SO_4, filtered, and concentrated *in vacuo*. To the mixture was then added $CDCl_3$ (2 mL) and hexafluorobenzene (1.0 eq., 0.2 mmol, 23.2 µL), at which point the ^{19}F NMR was measured. The NMR yield was then measured by comparing the integration of the fluorine signal of the product to that of the internal standard (δ = -161 ppm).

Esteves's TCCA conditions[17]:

To a 100 mL HDPE vial equipped with a Suba-seal under N_2 atmosphere was added py•9HF (0.1 mL) and TCCA (0.33 eq., 0.066 mmol, 15 mg). After cooling this solution to 0 ºC, alkene (0.2 M, 1 eq., 0.2 mmol, 57 mg) in CH_2Cl_2. After stirring overnight, the reaction mixture was quenched with 100 mL of cold (0 ºC) saturated aqueous $NaHCO_3$ solution. This stirred for 1 hour until the aqueous layer measured pH 7. The mixture was extracted into CH_2Cl_2, dried with Na_2SO_4, filtered, and concentrated *in vacuo*. To the mixture was then added $CDCl_3$ (2 mL) and hexafluorobenzene (1 eq., 0.2 mmol, 23.2 µL), at which point the ^{19}F NMR was measured. The NMR yield was then measured by comparing the integration of the fluorine signal of the product to that of the internal standard (δ = -161 ppm).

Modified Thibaudeau's HF/SbF$_5$ reaction conditions[9]:

To a 100 mL HDPE vial was added, fluoroantimonic acid (0.3 mL, purchased from Aldrich and used without further purification) and NCS (3 eq., 0.6 mmol, 50 mg). After stirring at -20 ºC, alkene (1 eq., 0.2 mmol, 57 mg) was added. After stirring overnight, the reaction mixture was quenched with 100 mL of cold (0 ºC) saturated aqueous Na_2CO_3 solution. This stirred for 1 hour until the aqueous layer measured pH > 7. The mixture was extracted into CH_2Cl_2, dried with Na_2SO_4, filtered, and concentrated *in vacuo*. To the mixture was then added $CDCl_3$ (2 mL) and hexafluorobenzene (1 eq., 0.2 mmol, 23.2 μL), at which point the ^{19}F NMR was measured. The NMR yield was then measured by comparing the integration of the fluorine signal of the product that of the internal standard (δ = -161 ppm).

Table S7 NMR yields for previously reported literature methods for the chlorofluorination of alkenes used as substrates in this work's scope.

Compound	NMR yield/%			
	Olah	Esteves	Modified Thibaudeau	This work
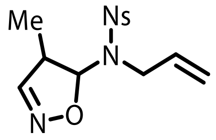 **8a**	<5%)	<5%	<5%	77 (rr 5:1)
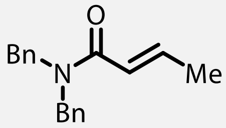 **4a**	21%	16%	<5%	55 (rr > 19:1)
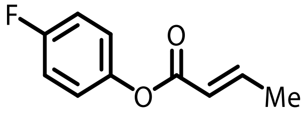 **3a**	23%	19%	<5%	70 (rr > 19:1)
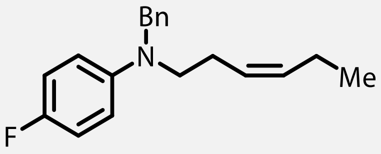 **1a-*cis***	33 (rr 6:1)	37 (rr 6:1)	<5%	85 (rr 12:1)

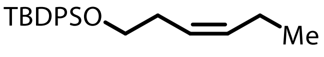 **5a**	0%	0%	0%	71 (1.5:1)
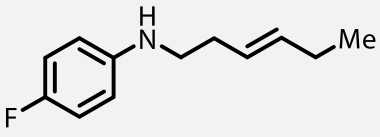 **6a**	21 (rr 4.5 :1)	17 (rr 4.5:1)	<5%	66 (11:1)

Variation of oxidant used in the formation of *p*-TolIF$_2$

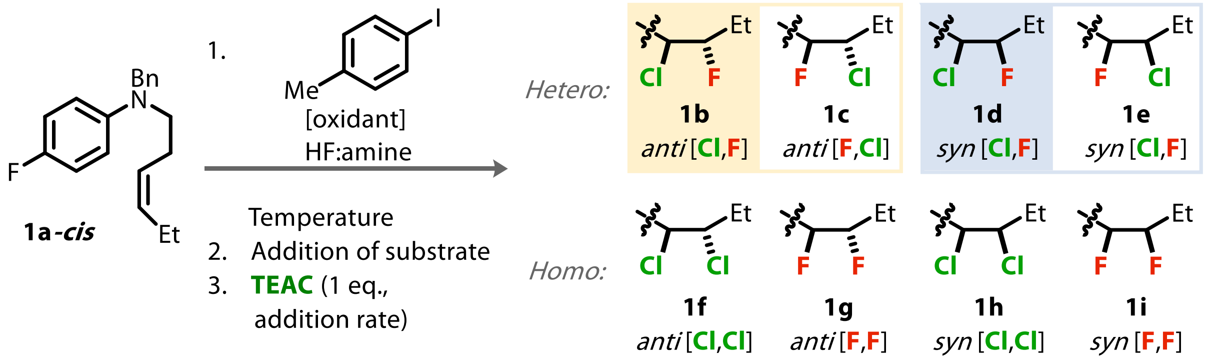

To a 100 mL HDPE vial was added, *p*-iodotoluene (0.2 eq., 0.04 mmol, 9 mg), external oxidant (if 70% *m*CPBA used (1.5 eq., 0.3 mmol, 76 mg) or if Selectfluor™ used (1.5 eq., 0.3 mmol, 106 mg). To this solution was added alkene **1a-*cis*** (1 eq., 0.2 mmol, 57 mg) and then TEAC (1 eq., 0.2 mmol, 34 mg). This vial was capped with a Suba-seal and a venting needle was placed through the Suba-seal. After stirring overnight, the reaction mixture was quenched with 100 mL of cold (0 ºC) saturated aqueous Na_2CO_3 solution. This stirred for 1 hour until the aqueous layer measured pH > 7. The mixture was extracted into CH_2Cl_2, dried with Na_2SO_4, filtered, and concentrated *in vacuo*. To the mixture was then added $CDCl_3$ (2 mL) and hexafluorobenzene (1 eq., 0.2 mmol, 23.2 µL), at which point the ^{19}F NMR was measured. The NMR yield was then measured by comparing the integration of the fluorine signal of the product (δ = -128.42 ppm) to that of the internal standard (δ = -161 ppm).

Table S8 **[a]** Our *anti*-protocol reaction conditions; **[b]** our *syn*-protocol reaction conditions. Unless specified otherwise; reactions with *m*CPBA standard conditions: 4-iodotoluene (0.2 eq.), *m*CPBA (1.5 eq.), CH_2Cl_2 (4 mL), py•9HF (0.5 mL). Then addition of **1a**-*cis* (1 eq., 0.2 mmol) and TEAC (1 eq., 0.2 mmol). Reactions with Selectfluor™ standard conditions: *p*-iodotoluene (0.2 eq.), Selectfluor™ (1.5 eq.), DCE (0.5 mL), HF source (0.5 mL). Then addition of **1a**-*cis* (1 eq., 0.2 mmol) and TEAC (1 eq., 0.2 mmol). **[c]** debenzylation of substrate; **[d]** several uncharacterizable side-products; **[e]** *p*-iodotoluene (1 eq., 0.2 mmol) and 4-hour pre-stir before addition of substrate.

Entry	Oxidant	HF:amine	Temp/ ºC	Addition rate	1b	1c	1d	1e	1f	1g	1h	1i	1a
1[a]	e-chem	5.6HF:amine	-46	0.20 eq./h	85	7	3	0	1	0	0	1	0
2[b]	e-chem	7HF:amine	-46	0.16 eq./h	6	0	55	3	6	3	0	5	0
3[c]	*m*CPBA	py•9HF	25	1 portion	1	0	0	0	0	0	0	1	<5
4	Selectfluor™	4.5HF:amine	25	1 portion	31	5	2	2	13	1	0	2	22
5	Selectfluor™	4.5HF:amine	-46	1 portion	3	0	0	0	1	0	0	0	95
6	Selectfluor™	4.5HF:amine	-46	0.20 eq./h	13	1	0	0	2	0	0	0	75
7	Selectfluor™	py•9HF	-46	0.20 eq./h	8	0	0	0	0	0	0	0	87
8[d][e]	Selectfluor™	4.5HF:amine	25	1 portion	41	8	2	0	4	0	0	0	0
9[d][e]	*m*CPBA	py•9HF	25	1 portion	5	1	1	0	8	1	0	5	12
10	*m*CPBA	5.6HF:amine	-46 ºC	0.16 eq./h	23	3	0	0	0	0	0	0	0
11	*m*CPBA	7.0HF:amine	-46 ºC	0.16 eq./h	8	0	24	4	0	0	0	0	1
12	Selectfluor™	5.6HF:amine	-46	0.16 eq./h	47	15	1	0	0	0	0	0	0
13	Selectfluor™	7.0HF:amine	-46	0.16 eq./h	5	1	40	8	0	0	2	0	0

Anti-chlorofluorination using electrophilic fluorine source and nucleophilic chloride source (F^+/Cl^-)

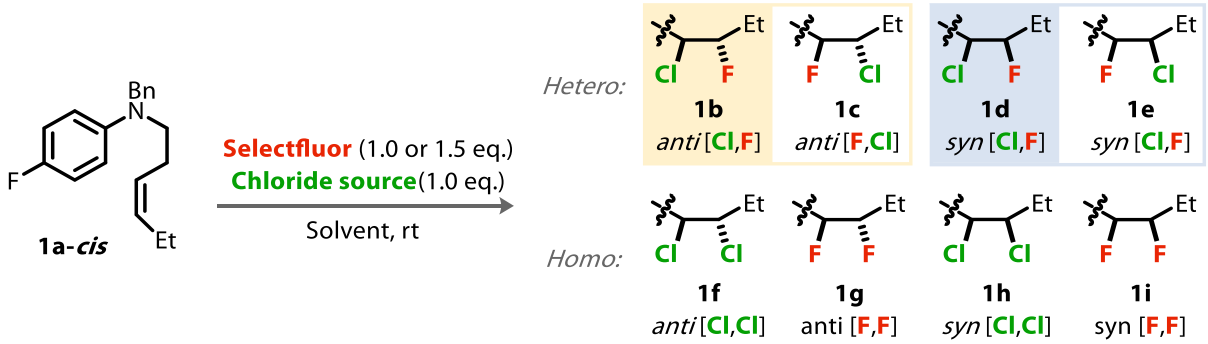

To a 100 mL HDPE vial were added Selectfluor (1.0 or 1.5 eq., 0.2 mmol or 0.3 mmol, 70.9 mg or 106 mg) and solvent (total 2 mL). The vial was capped with a Suba-seal and a venting needle placed through the Suba seal. Alkene **1a-*cis*** (1.0 eq., 0.2 mmol, 57 mg, 0.6 м) in solvent (0.3 mL) and chloride source (1.0 eq., 0.2 mmol) in solvent were each added in one portion. After stirring overnight, the reaction mixtures were diluted in the same solvent in which the reaction is carried out and concentrated under vacuum. To the residue was then added $CDCl_3$ (2 mL) and hexafluorobenzene (1 eq., 0.2 mmol, 23.2 µL), at which point the ^{19}F NMR was measured. The NMR yield was then measured by comparing the integration of the fluorine signal of the product (δ = -128.42 ppm) to that of the internal standard (δ = -161 ppm).

Table S9 ^{19}F NMR yield data for attempted electrophilic fluorine/nucleophilic chloride approaches towards alkene 1,2-chlorofluorination of model substrate *cis*-**1a**.

Equiv. of Selectfluor	Solvent	Cl^- source	1b	1c	1d	1e	1f	1g	1h	1i
1.0	5.6HF:amine/CH_2Cl_2[a]	TEAC[b]	12	3	0	0	0	0	0	0
1.5	5.6HF:amine/CH_2Cl_2[a]	TEAC[b]	21	5	0	0	0	0	0	0
1.0	CH_2Cl_2	TEAC	0	0	0	0	0	0	0	0
1.5	CH_2Cl_2	TEAC	0	0	0	0	0	0	0	0
1.0	THF	aq. HCl[c]	0	0	0	0	0	0	0	0
1.5	THF	aq. HCl[c]	0	0	0	0	0	0	0	0

[a]0.75 mL 5.6HF:amine stock solution and 1.25 mL CH_2Cl_2 were added. [b]TEAC (34 mg) was added as a 0.2 м solution in CH_2Cl_2. [c]Aqueous 1 м HCl (0.2 mL) was added neat and not dissolved in THF solvent before addition.

Studies into identity of active iodane under *anti*-chlorofluorination regime

Synthesis of *p*TolICl$_2$

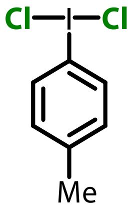

The hypervalent iodine oxidant, **201a**, was synthesised according to a literature procedure[18] using *p*-iodotoluene (5 mmol) to yield a colourless solid (682 g, 57%).

^{1}H NMR (300 MHz, CDCl$_3$): δ = 8.15 – 8.10 (2H, m), 7.35 – 7.28 (2H,m), 2.41 (3H, s).

^{13}C NMR (101 MHz, CDCl$_3$): δ = 143.7, 133.8, 131.7, 122.5, 21.4.

This data is consistent with those previously reported. [18]

Use of pre-formed *p*-TolICl$_2$ in the reaction

To a 100 mL HDPE vial was added, HF:amine stock solution (0.75 mL of 5.6HF:amine or 1.25 mL of 7HF:amine) and alkene **1a-*cis*** (1.0 м, 1 eq., 0.2 mmol, 57 mg) in CH_2Cl_2. This vial was capped with a Suba-seal, a venting needle was placed through the Suba-seal and cooled to –46 °C. To this solution was added *p*-TolICl$_2$ (0.8 м, 1 eq., 0.2 mmol, 57 mg) in CH_2Cl_2 (1 mL). After stirring overnight, the reaction mixture was quenched with 100 mL of cold (0 ºC) saturated aqueous $NaHCO_3$ solution. This stirred for 1 hour until the aqueous layer measured pH 7. The mixture was extracted into CH_2Cl_2, dried with Na_2SO_4, filtered and concentrated *in vacuo*. To the mixture was then added $CDCl_3$ (2 mL) and hexafluorobenzene (1 eq., 0.2 mmol, 23.2 μL), at which point the ^{19}F NMR was measured. The NMR yield was then measured by comparing the integration of the fluorine signal of the product (δ = -128.42 ppm) to that of the internal standard (δ = -161 ppm).

Table S10 ^{19}F NMR yields for reactions utilising *p*-TolICl$_2$ as sole oxidant.

**HF:amine ratio**	**1b**	**1c**	**1d**	**1e**	**1f**	**1g**	**1h**	**1i**
5.6	<5	<5	0	0	0	0	0	0
5.6[a]	13	1	0	0	9	0	0	0
7[b]	59	5	0	0	0	0	0	0

[a]The order of addition was swapped so that *p*-TolICl$_2$ was added to 5.6HF:amine/CH_2Cl_2 at −46 °C and stirred for 10 min prior to addition of **1a-*cis***.
[b]The order of addition was swapped so that *p*-TolICl$_2$ was added to 7HF:amine/CH_2Cl_2 at −46 °C and stirred for 10 min prior to addition of **1a-*cis***.

Use of pre-formed *p*-TolIF$_2$ and *p*-TolICl$_2$ in the reaction

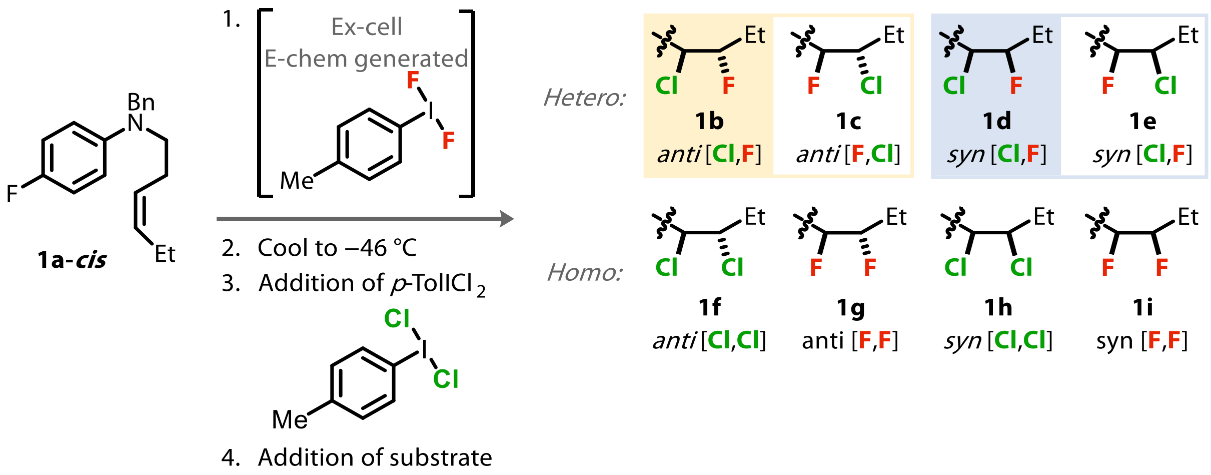

To a 100 mL HDPE vial was added *p*-TolIF$_2$ (0.5 eq., 0.1 mmol, 0.5 mL of anodic compartment reaction mixture, withdrawn directly from divided cell via syringe), 5.6HF:amine stock solution (0.38 mL) and CH_2Cl_2 (1.13 mL). This vial was capped with a Suba-seal and a venting needle was placed through the Suba-seal, and then cooled to −46 °C. *p*-TolICl$_2$ (0.1 м, 0.5 eq., 0.1 mmol, 29 mg) in CH_2Cl_2 (1 mL) was added in one portion. After stirring for 10 min, **1a-*cis*** (0.6 м, 1.0 eq., 0.2 mmol) in CH_2Cl_2 (0.3 mL) was added. After stirring overnight, the reaction mixture was quenched with 300 mL of cold (0 °C) saturated aqueous $NaHCO_3$ solution until the aqueous layer measured pH 7, and then the reaction mixture was stirred for 1 hour. The mixture was extracted into CH_2Cl_2, dried with Na_2SO_4, filtered and concentrated *in vacuo*. To the residue was then added $CDCl_3$ (2 mL) and hexafluorobenzene (1 eq., 0.2 mmol, 23.2 μL), at which point the ^{19}F NMR was measured. The NMR yield was then measured by comparing the integration of the fluorine signal of the product (δ = -128.42 ppm) to that of the internal standard (δ = -161 ppm).

Table S11 ^{19}F NMR yields (relative to C_6F_6) for reactions utilising *p*-TolIF$_2$ and *p*-TolICl$_2$ as oxidant combination.

HF:amine ratio	1b	1c	1d	1e	1f	1g	1h	1i
5.6	58	7	0	0	0	0	0	0
7	40	3	5	0	0	0	0	0

Use of pre-formed p-Tol-ICl2 in presence of Tol-I

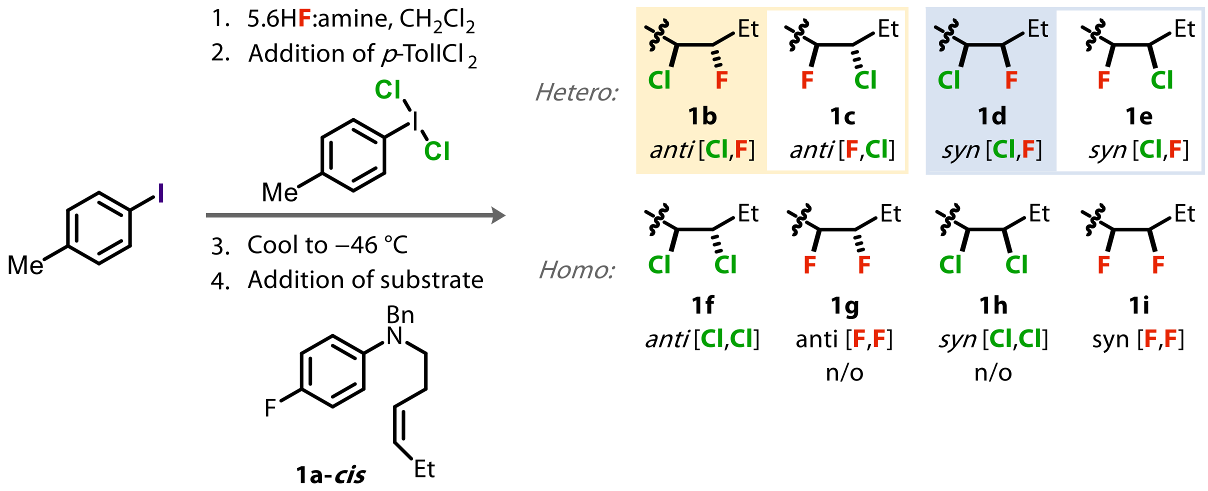

To a 100 mL HDPE vial was added *p*-TolI (0.5 eq., 0.1 mmol, 44 mg), 5.6HF:amine stock solution (0.75 mL) and CH_2Cl_2 (1.25 mL). This vial was capped with a Suba-seal and a venting needle was placed through the Suba-seal, and then cooled to −46 °C. *p*-TolICl2 (0.1 м, 0.5 eq., 0.1 mmol, 29 mg) in CH_2Cl_2 (1 mL) was added in one portion. After stirring for 15 min, **1a-*cis*** (0.6 м, 1.0 eq., 0.2 mmol) in CH_2Cl_2 (0.3 mL) was added. After stirring for 4 h, the reaction mixture was quenched with 300 mL of cold (0 °C) saturated aqueous $NaHCO_3$ solution until the aqueous layer measured pH 7, and then the reaction mixture was stirred for 1 hour. The mixture was extracted into CH_2Cl_2, dried with Na_2SO_4, filtered and concentrated *in vacuo*. To the residue was then added $CDCl_3$ (2 mL) and hexafluorobenzene (1 eq., 0.2 mmol, 23.2 μL), at which point the ^{19}F NMR was measured. The NMR yield was then measured by comparing the integration of the fluorine signal of the product (δ = -128.42 ppm) to that of the internal standard (δ = -161 ppm).

Table S12 ^{19}F NMR yields (relative to C_6F_6) with respect to *p*-TolICl2 as limiting reagent, where only 0.5 eq. *p*-TolIC2 was used.

1a-*cis*	1b	1c	1d	1e	1f	1g	1h	1i
27	20	4	0	0	0	0	0	0

Reaction monitoring of *anti*-chlorofluorination

Reaction monitoring of *p*-Tol-IF_2 in presence of alkene and chloride

To one compartment of PTFE divided cell equipped with a Nafion™ membrane and stirrer bars, CH_2Cl_2 (1.5 mL) and 5.6 HF:amine stock solution (4.5 mL) were added. To the other compartment of the PTFE divided cell was added CD_2Cl_2 (1.5 mL) and 5.6HF:amine stock solution. To the anodic compartment containing CD_2Cl_2, p-iodotoluene (2 eq., 1.2 mmol, 262 mg) was then added. Each compartment was then capped and wrapped in parafilm. A platinum electrode was inserted into each compartment, and the reaction was subjected to electrolysis (17 mA, 2.2 *F*, 4.2 h). The electrodes were removed and the anodic compartment mixture (3 mL) was transferred to a 100 mL HDPE vial equipped with a stirrer bar, diluted with further CD_2Cl_2 (3 mL), dosed with ethyl fluoroacetate (29 μL) as internal standard (for monitoring by ^{1}H and ^{19}F NMR), capped with a Suba seal equipped with a venting needle and cooled to −46 °C whilst stirring.

After cooling to −46 °C for 10 min, an aliquot (30 μL) of the mixture in the HDPE vial was removed and transferred to a NMR tube equipped with PTFE liner. ^{1}H and ^{19}F NMR spectra were recorded at −46 °C in a 300 MHz NMR spectrometer whose probe temperature was also set to −46 °C. A NMR sample containing CD_2Cl_2 and C_6F_6 was used once to set the shimming parameters for subsequent samples.

Substrate *cis*-**1a** (1.0 eq., 170 mg, 0.6 mmol, 0.6 M in CD_2Cl_2) was added to the mixture. After 15 min, an aliquot (30 μL) of the mixture in the HDPE vial was removed, and its ^{1}H and ^{19}F NMR spectra were recorded at a NMR probe temperature of −46 °C.

Tetraethylammonium chloride (0.25 eq., 25 mg, 0.15 mmol, 0.2 M in CD_2Cl_2) was added to the mixture in 30 min intervals. 15 min after each addition of tetraethylammonium chloride, an aliquot (30 μL) of the mixture in the HDPE vial was removed via needle and syringe and transferred to a NMR tube equipped with PTFE liner, and its ^{1}H and ^{19}F NMR spectra were recorded at a NMR probe temperature of −46 °C.

To a 100 mL HDPE vial was added ethyl fluoroacetate (1.5 eq., 29 μL, 0.3 mmol), substrate *cis*-**1a** (57 mg, 0.2 mmol, 0.6 M in CD_2Cl_2), CD_2Cl_2 (1.25 mL) and 5.6HF:amine (0.75 mL). An aliquot (30 μL) of the mixture in the HDPE vial was removed via needle and syringe and transferred to a NMR tube equipped with PTFE liner, and its ^{1}H and ^{19}F NMR spectra were recorded at a NMR probe temperature of −46 °C.

To a 100 mL HDPE vial was added ethyl fluoroacetate (1.5 eq.), 4-iodotoluene (44 mg), CD_2Cl_2 (1.25 mL) and 5.6HF:amine (0.75 mL). An aliquot (30 μL) of the mixture in the HDPE vial was removed via needle and syringe and transferred to a NMR tube equipped with PTFE liner, and its ^{1}H and ^{19}F NMR spectra were recorded at a NMR probe temperature of −46 °C.

^{1}H NMR spectrum of 4-iodotoluene at −46 °C

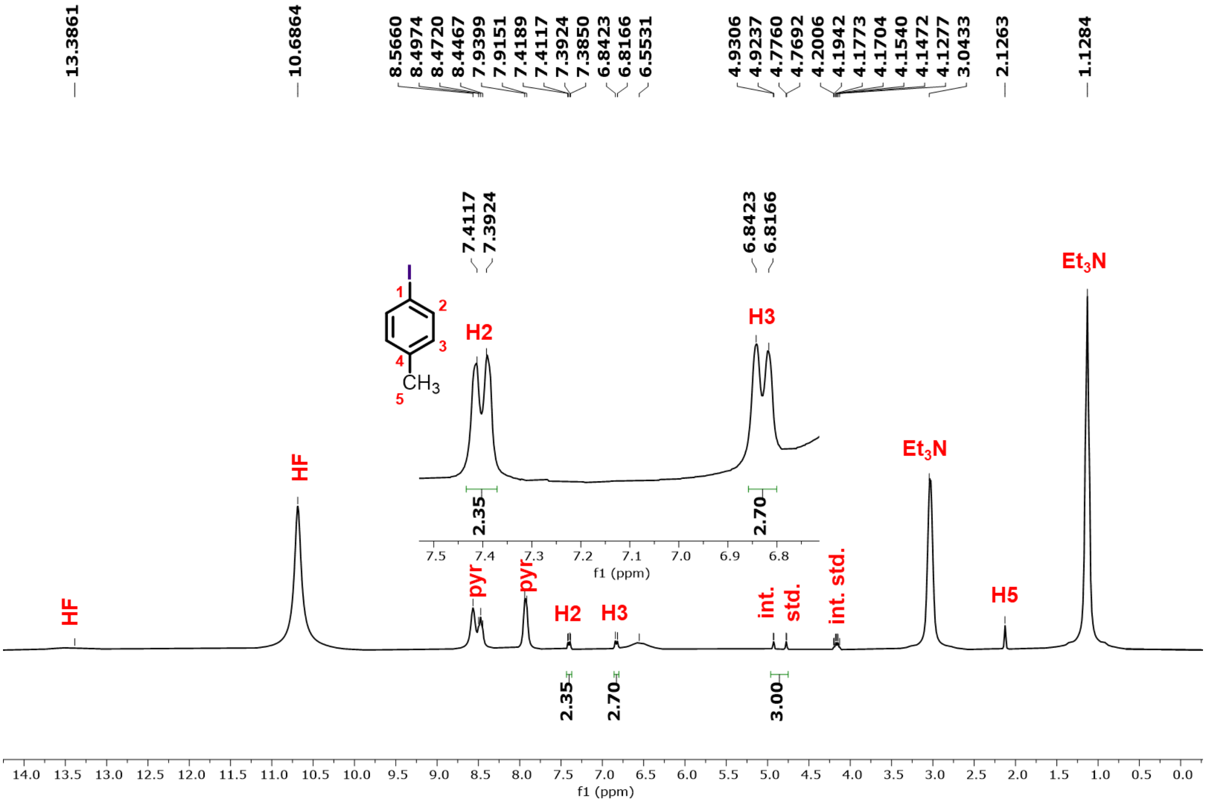

Figure S2 ^{1}H NMR spectrum of 4-iodotoluene in 5.6HF:amine/CD_2Cl_2 at −46 °C.

^{1}H NMR spectrum of *p*-Tol-IF_2 at −46 °C

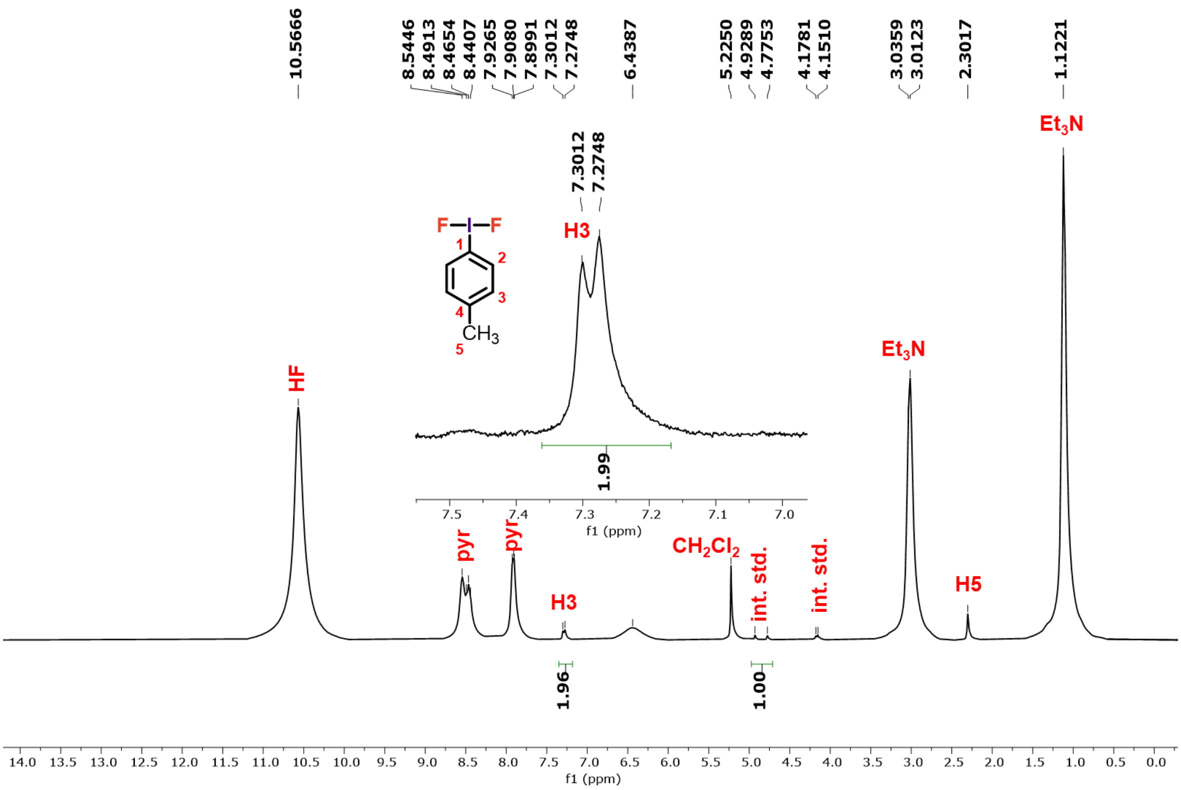

Figure S3 ^{1}H NMR spectrum of *p*-Tol-IF_2 in 5.6HF:amine/CD_2Cl_2 at −46 °C.

^{19}F NMR spectrum of *p*-Tol-IF_2 at −46 °C

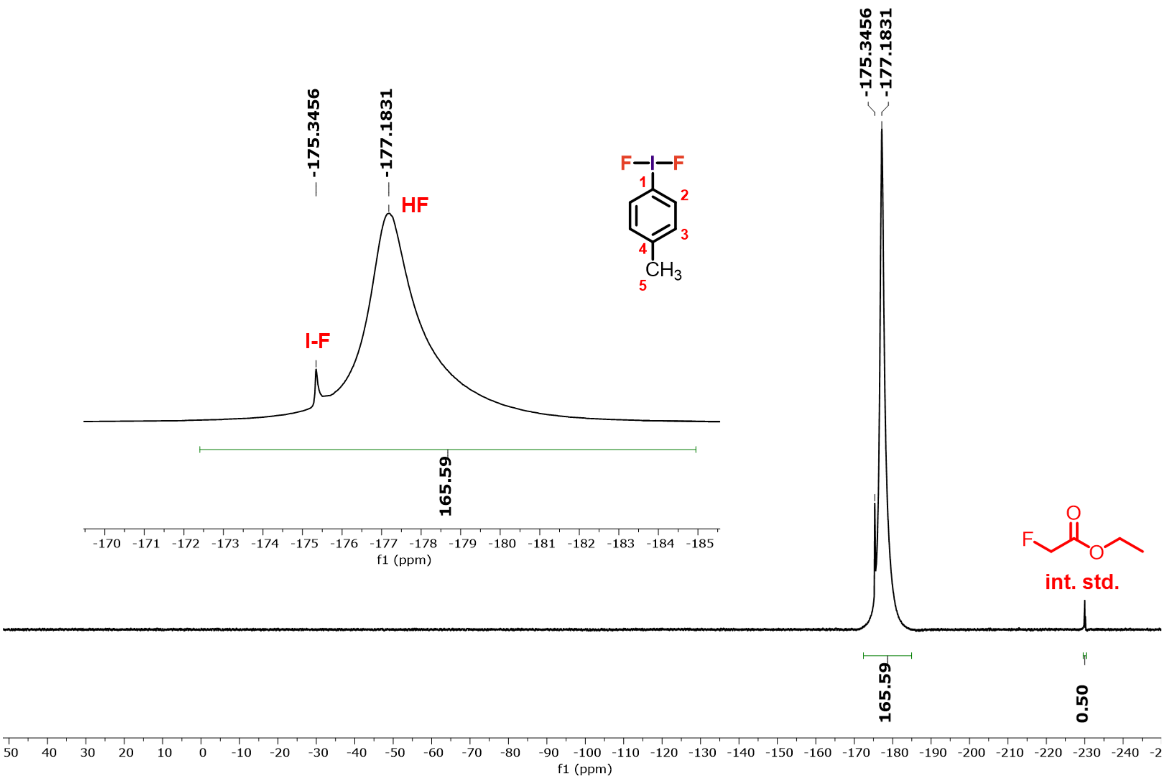

Figure S4 ^{19}F NMR spectrum of *p*-Tol-IF_2 in 5.6HF:amine/CD_2Cl_2 at −46 °C.

^{1}H NMR spectrum of *p*-Tol-IF$_2$ + alkene *cis*-1a at −46 °C

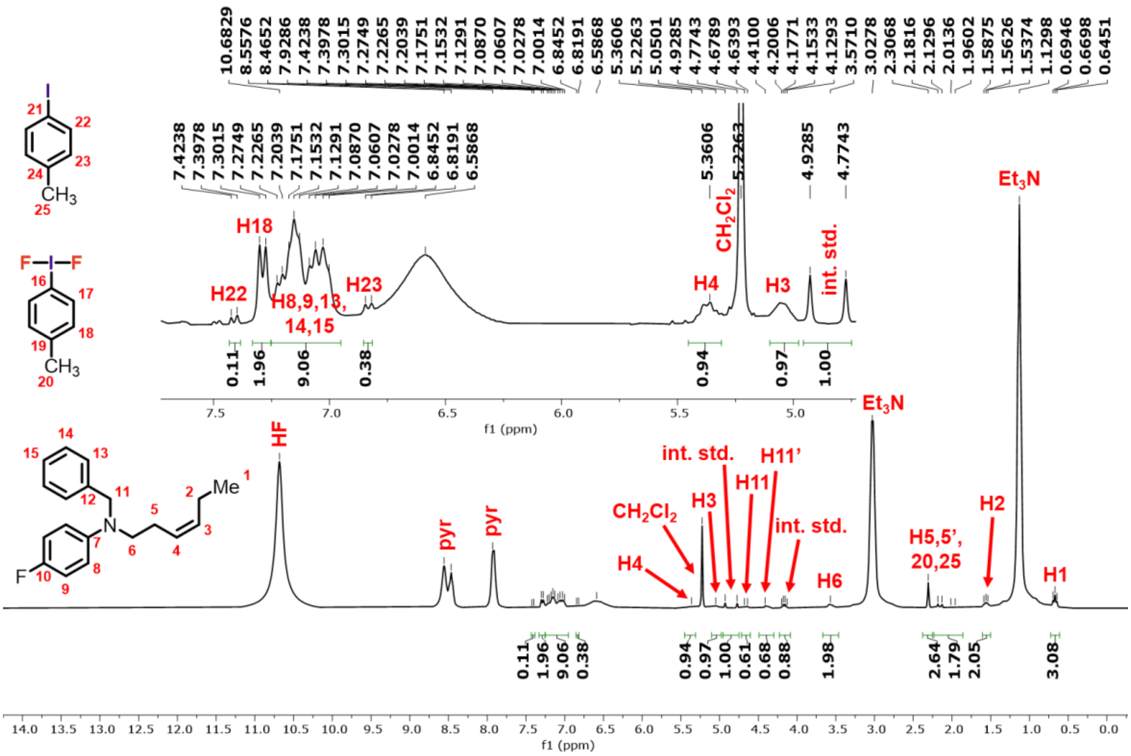

Figure S5 ^{1}H NMR spectrum of *p*-Tol-IF$_2$ + 1.0 eq. *cis*-**1a** in 5.6HF:amine/CD$_2$Cl$_2$ at −46 °C.

^{19}F NMR spectrum of *p*-Tol-IF$_2$ + alkene *cis*-1a at −46 °C

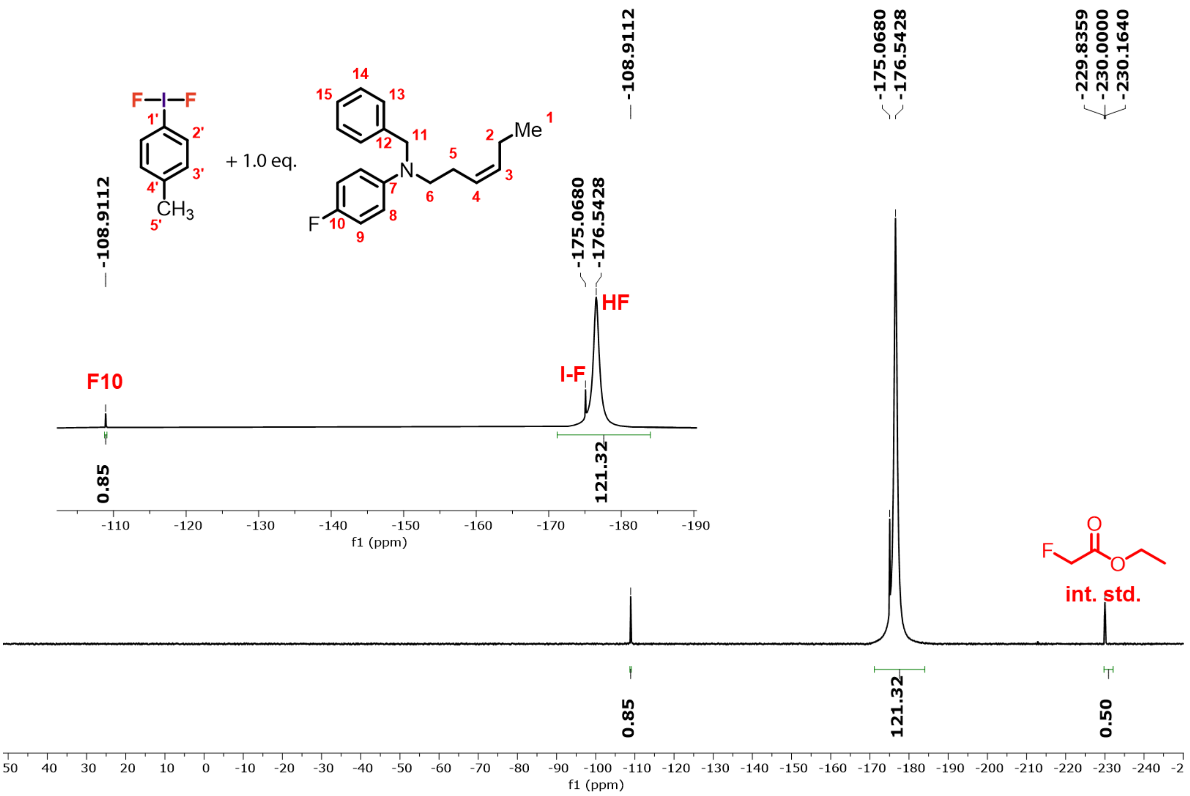

Figure S6 ^{19}F NMR spectrum of *p*-Tol-IF$_2$ + 1.0 eq. *cis*-1a in 5.6HF:amine/CD$_2$Cl$_2$ at −46 °C.

^{1}H NMR spectrum of alkene *cis*-1a at −46 °C

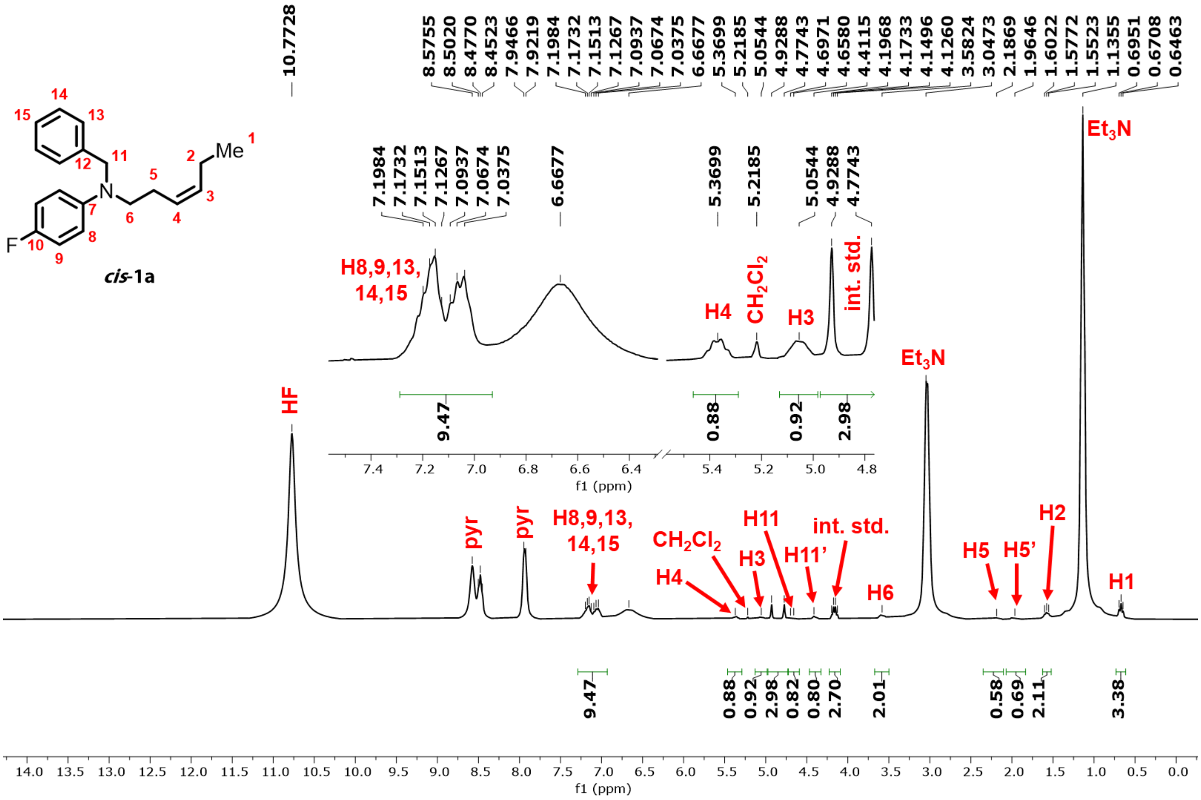

Figure S7 ^{1}H NMR spectrum *cis*-**1a** in 5.6HF:amine/CD_2Cl_2 at −46 °C.

^{19}F NMR spectrum of alkene *cis*-1a at −46 °C

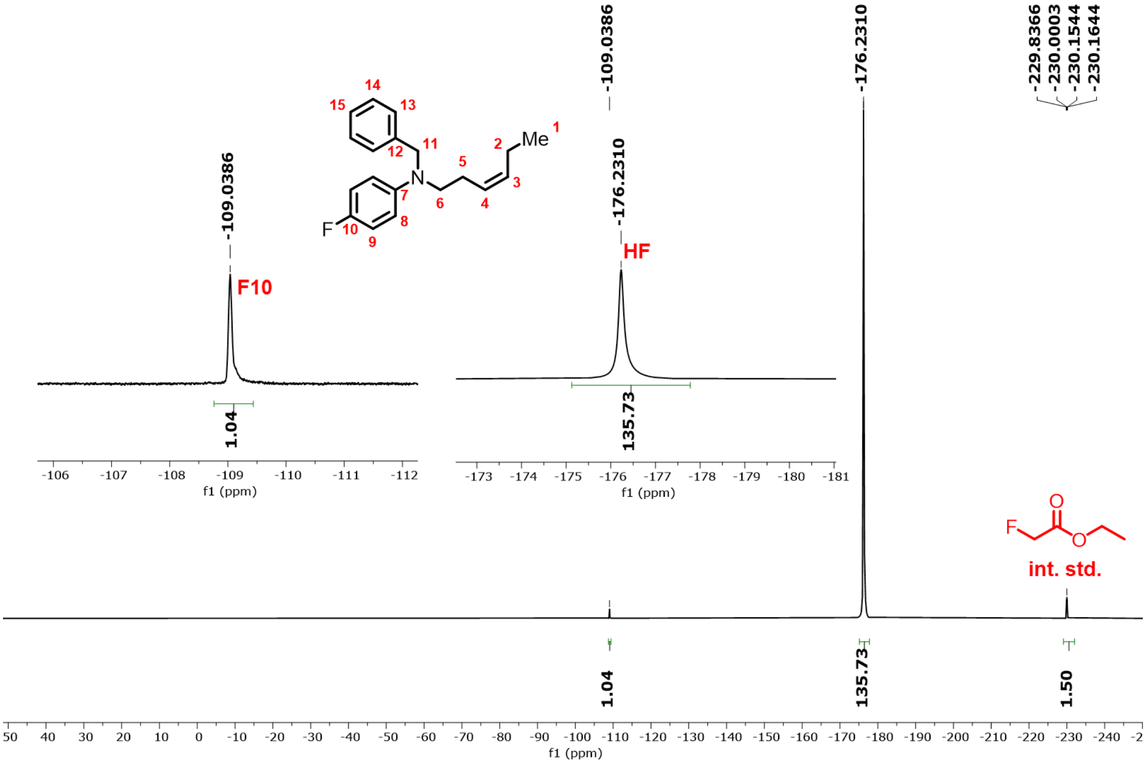

Figure S8 ^{19}F NMR spectrum of *cis*-**1a** in 5.6HF:amine/CD_2Cl_2 at −46 °C.

^{1}H NMR spectrum of *p*-Tol-IF2 + alkene *cis*-1a + 0.25 eq. chloride at −46 °C

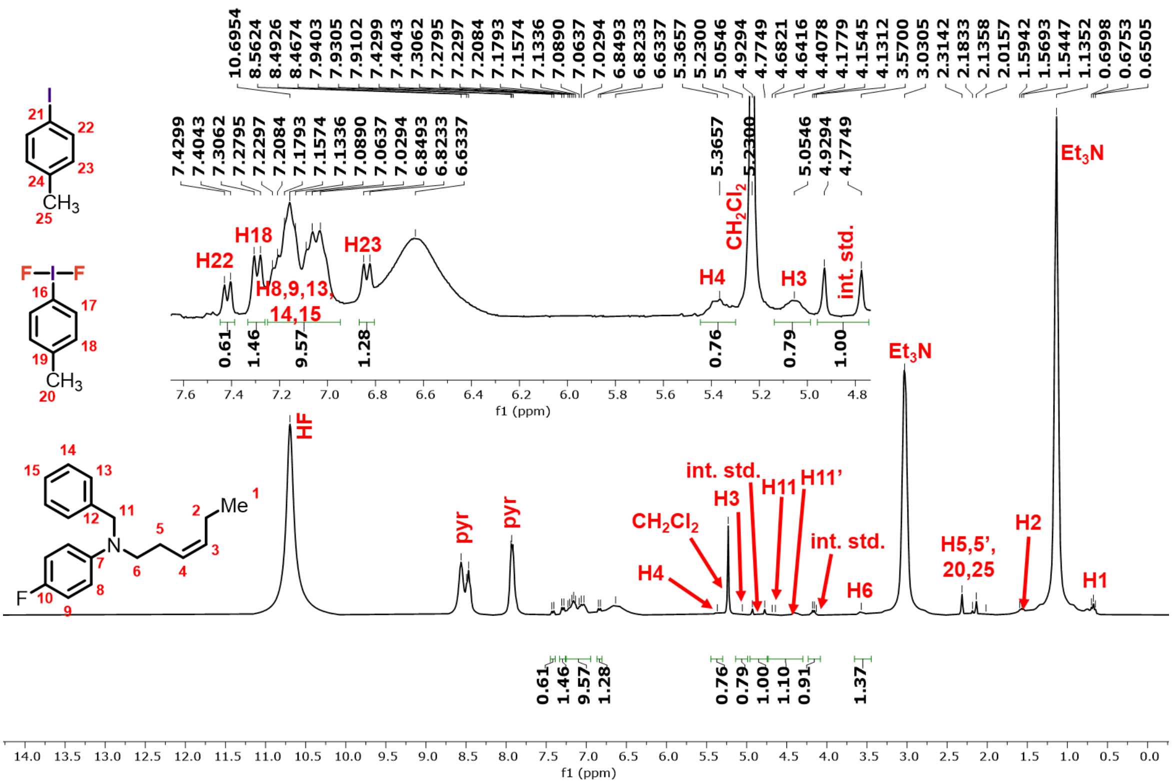

Figure S9 ^{1}H NMR spectrum *p*-Tol-IF2 + *cis*-**1a** + Et4NCl (0.25 eq.) in 5.6HF:amine/CD2Cl2 at −46 °C.

^{19}F NMR spectrum of *p*-Tol-IF2 + alkene *cis*-1a + 0.25 eq. chloride at −46 °C

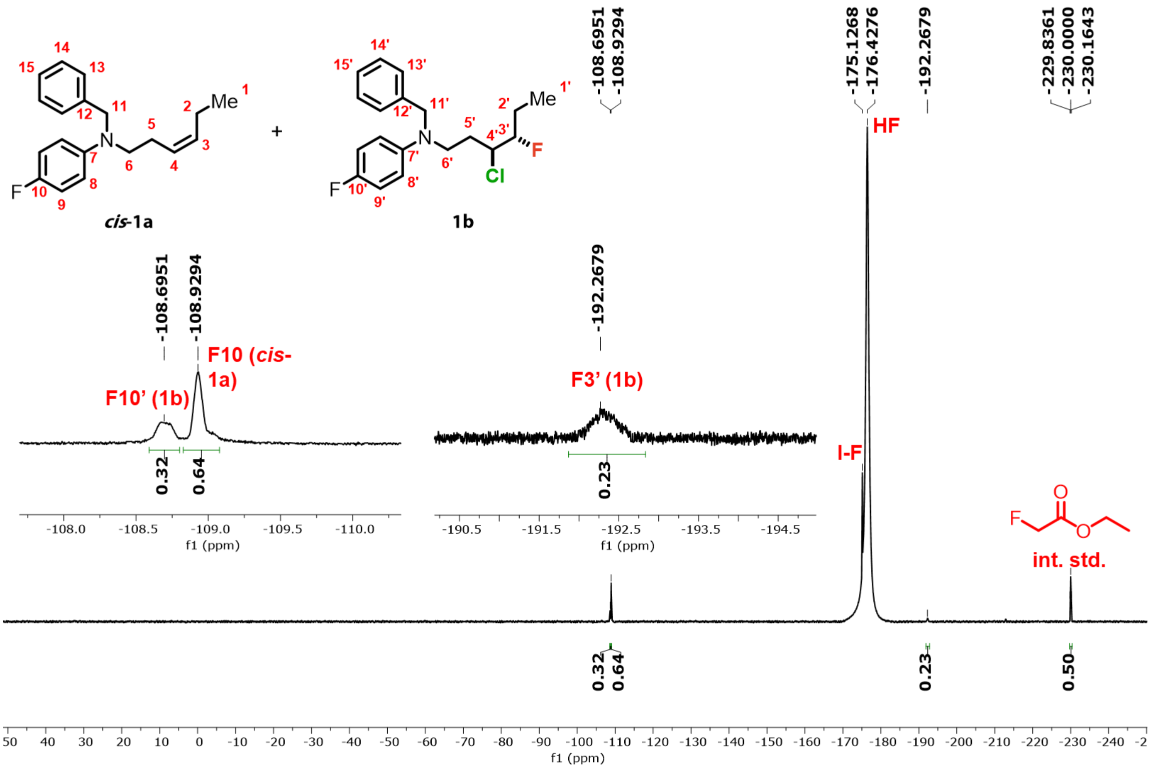

Figure S10 ^{19}F NMR spectrum *p*-Tol-IF2 + *cis*-1a + Et4NCl (0.25 eq.) in 5.6HF:amine/CD2Cl2 at −46 °C.

^{1}H NMR spectrum of *p*-Tol-IF₂ + alkene *cis*-1a + 0.5 eq. chloride at −46 °C

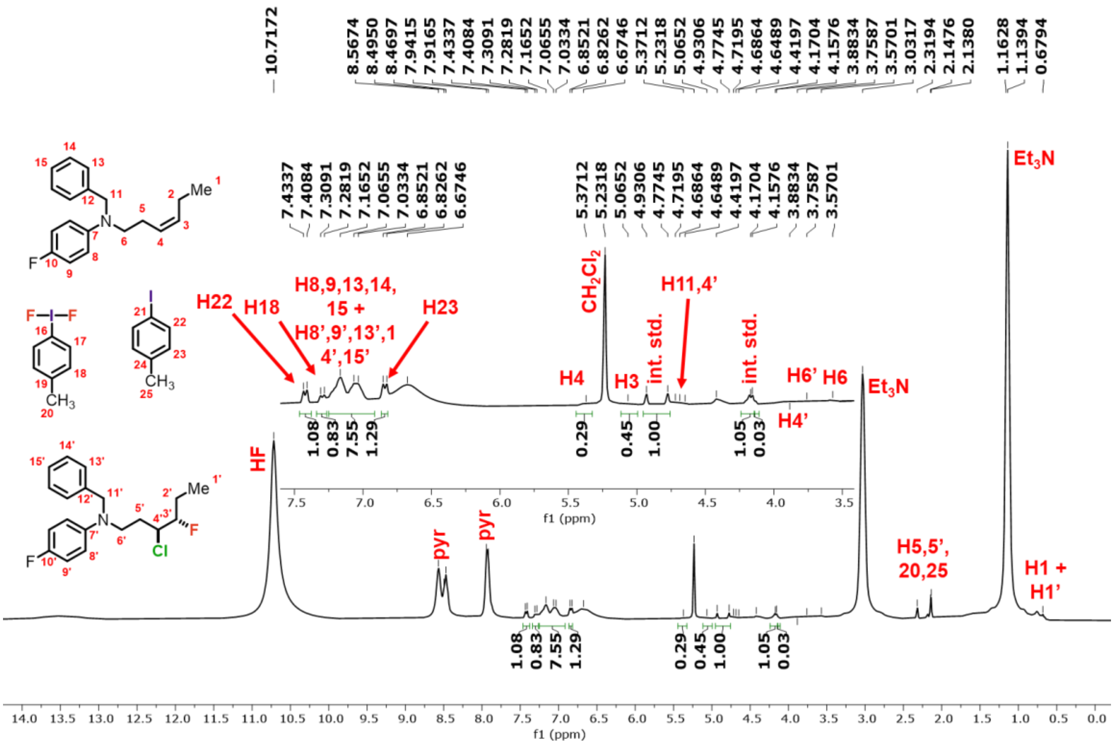

Figure S11 ^{1}H NMR spectrum *p*-Tol-IF$_2$ + *cis*-**1a** + Et$_4$NCl (0.5 eq.) in 5.6HF:amine/CD$_2$Cl$_2$ at −46 °C.

^{19}F NMR spectrum of *p*-Tol-IF₂ + alkene *cis*-1a + 0.5 eq. chloride at −46 °C

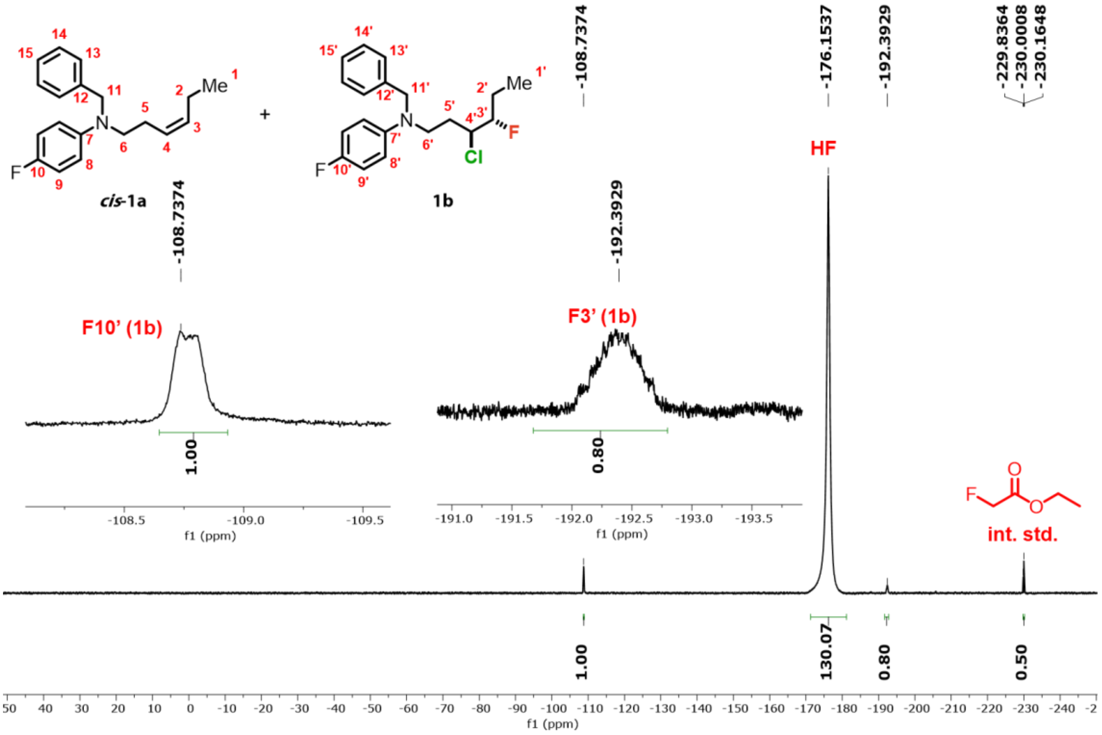

Figure S12 ^{19}F NMR spectrum *p*-Tol-IF$_2$ + *cis*-**1a** + Et$_4$NCl (0.5 eq.) in 5.6HF:amine/CD$_2$Cl$_2$ at −46 °C.

^{1}H NMR spectrum of *p*-Tol-IF_2 + alkene *cis*-1a + 0.75 eq. chloride at −46 °C

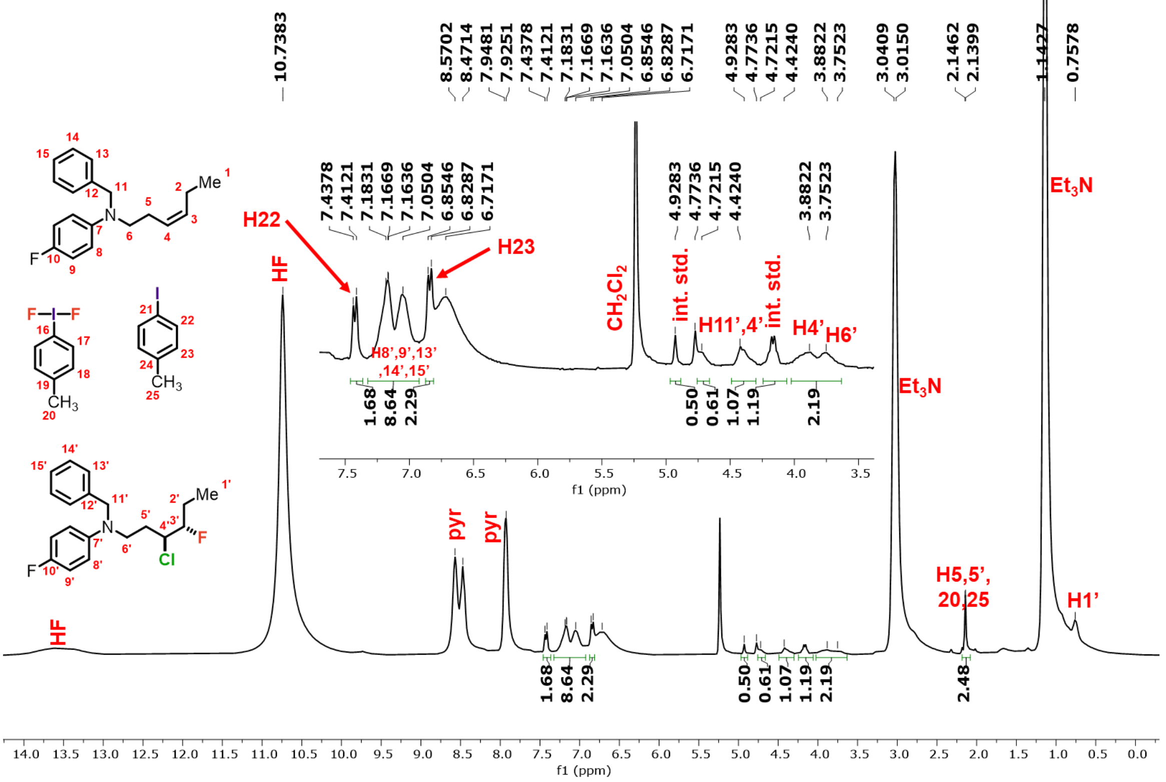

Figure S13 ^{1}H NMR spectrum *p*-Tol-IF_2 + *cis*-1a + Et_4NCl (0.75 eq.) in 5.6HF:amine/CD_2Cl_2 at −46 °C.

^{19}F NMR spectrum of *p*-Tol-IF_2 + alkene *cis*-1a + 0.75 eq. chloride at −46 °C

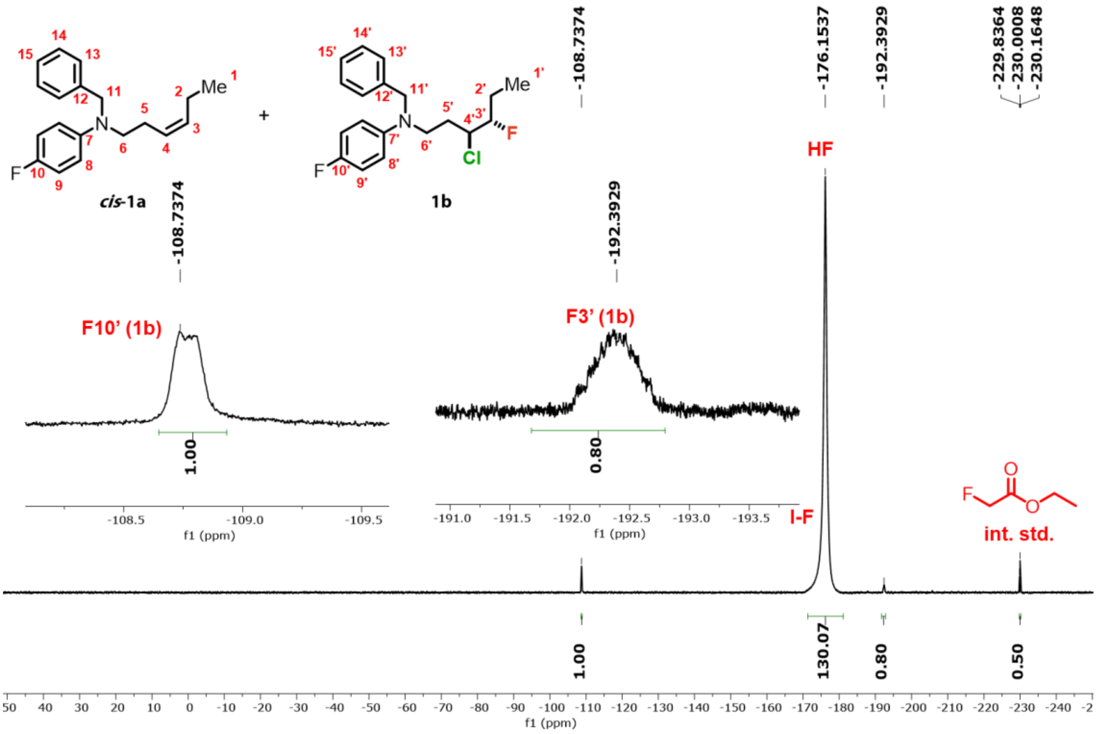

Figure S14 ^{19}F NMR spectrum *p*-Tol-IF_2 + *cis*-1a + Et_4NCl (0.75 eq.) in 5.6HF:amine/CD_2Cl_2 at −46 °C.

^{1}H NMR spectrum of *p*-Tol-IF$_2$ + alkene *cis*-1a + 1.0 eq. chloride at −46 °C

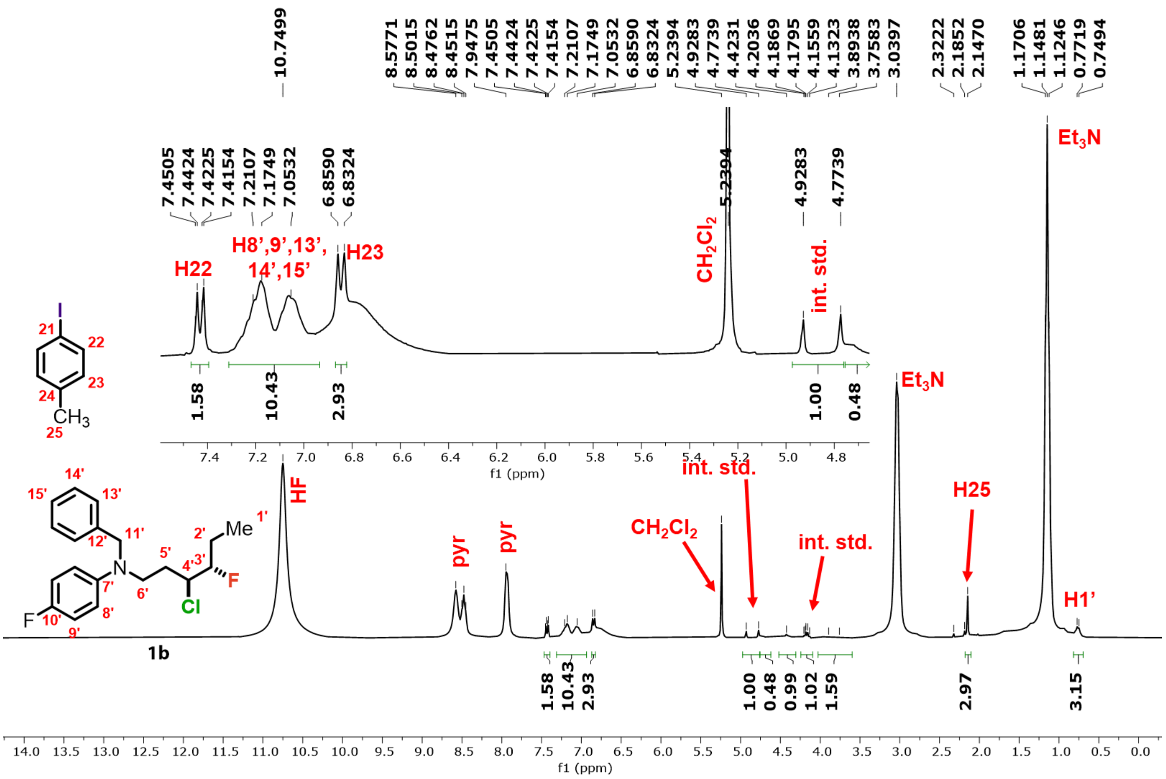

Figure S15 ^{1}H NMR spectrum *p*-Tol-IF$_2$ + *cis*-1a + Et$_4$NCl (1.0 eq.) in 5.6HF:amine/CD$_2$Cl$_2$ at −46 °C.

^{19}F NMR spectrum of *p*-Tol-IF$_2$ + alkene *cis*-1a + 1.0 eq. chloride at −46 °C

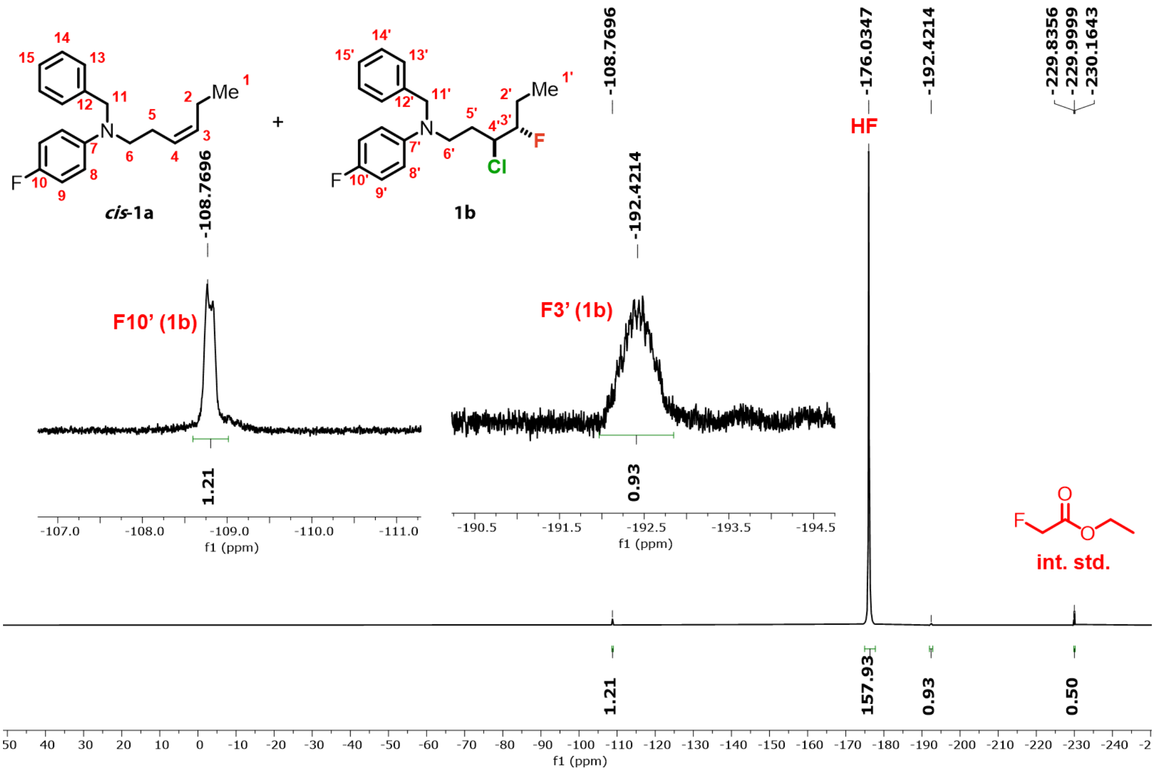

Figure S16 ^{19}F NMR spectrum *p*-Tol-IF$_2$ + *cis*-**1a** + Et$_4$NCl (1.0 eq.) in 5.6HF:amine/CD$_2$Cl$_2$ at −46 °C.

Stacked ^{1}H NMR spectra of *p*-Tol-IF$_2$ + *cis*-1a + Et$_4$NCl at −46 °C

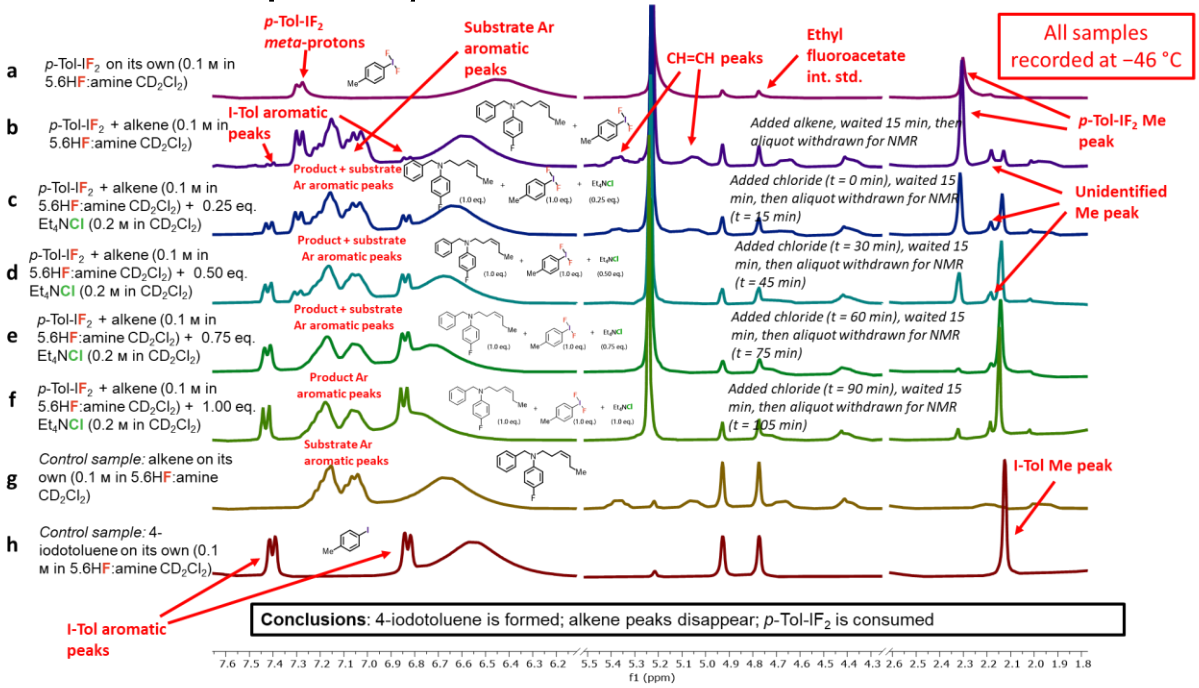

Figure S17 Stacked ^{1}H NMR spectra for reaction monitoring of *anti*-chlorofluorination of *cis*-**1a** using *p*-Tol-IF$_2$ and Et$_4$NCl. **(a)** ^{1}H NMR spectrum of *p*-Tol-IF$_2$ in 5.6HF:amine/CD$_2$Cl$_2$; **(b)** ^{1}H NMR spectrum of *p*-Tol-IF$_2$ + 1.0 eq. *cis*-**1a** in 5.6HF:amine/CD$_2$Cl$_2$; **(c-f)** ^{1}H NMR spectra of *p*-Tol-IF$_2$ + 1.0 eq. *cis*-**1a** + Et$_4$NCl (0.25-1.0 eq.) in 5.6HF:amine/CD$_2$Cl$_2$; **(g)** ^{1}H NMR spectrum of *cis*-**1a** in 5.6HF:amine/CD$_2$Cl$_2$; **(h)** ^{1}H NMR spectrum of *p*-Tol-I in 5.6HF:amine/CD$_2$Cl$_2$. All ^{1}H NMR spectra were recorded at −46 °C using ethyl fluoroacetate as internal standard.

Stacked ^{19}F NMR spectra of *p*-Tol-IF$_2$ + *cis*-1a + Et$_4$NCl at −46 °C

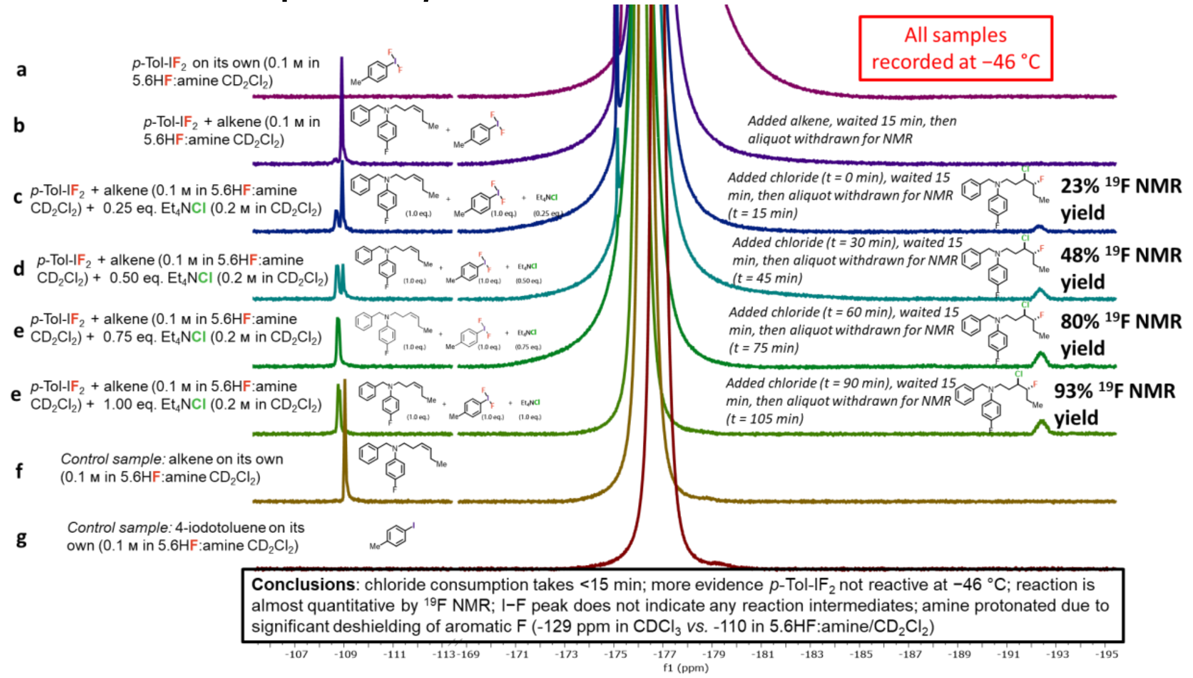

Figure S18 Stacked ^{19}F NMR spectra for reaction monitoring of *anti*-chlorofluorination of *cis*-**1a** using *p*-Tol-IF$_2$ and Et$_4$NCl. **(a)** ^{19}F NMR spectrum of *p*-Tol-IF$_2$ in 5.6HF:amine/CD$_2$Cl$_2$; **(b)** ^{19}F NMR spectrum of *p*-Tol-IF$_2$ + 1.0 eq. *cis*-**1a** in 5.6HF:amine/CD$_2$Cl$_2$; **(c-f)** ^{19}F NMR spectra of *p*-Tol-IF$_2$ + 1.0 eq. *cis*-**1a** + Et$_4$NCl (0.25-1.0 eq.) in 5.6HF:amine/CD$_2$Cl$_2$; **(g)** ^{19}F NMR spectrum of *cis*-**1a** in 5.6HF:amine/CD$_2$Cl$_2$; **(h)** ^{19}F NMR spectrum of *p*-Tol-I in 5.6HF:amine/CD$_2$Cl$_2$. All ^{19}F NMR spectra were recorded at −46 °C using ethyl fluoroacetate as internal standard.

Reaction monitoring of *p*-Tol-IF_2 in presence of chloride

To one compartment of PTFE divided cell equipped with a Nafion™ membrane and stirrer bars, CH_2Cl_2 (1.5 mL) and 5.6 HF:amine stock solution (4.5 mL) were added. To the other compartment of the PTFE divided cell was added CD_2Cl_2 (1.5 mL) and 5.6HF:amine stock solution. To the anodic compartment containing CD_2Cl_2, p-iodotoluene (2 eq., 1.2 mmol, 262 mg) was then added. Each compartment was then capped and wrapped in parafilm. A platinum electrode was inserted into each compartment, and the reaction was subjected to electrolysis (17 mA, 2.2 F, 4.2 h). The electrodes were removed and the anodic compartment mixture (3 mL) was transferred to a 100 mL HDPE vial equipped with a stirrer bar, diluted with further CD_2Cl_2 (3 mL), dosed with ethyl fluoroacetate (29 µL) as internal standard (for monitoring by ^{1}H and ^{19}F NMR), capped with a Suba seal equipped with a venting needle and cooled to −46 °C whilst stirring.

After cooling to −46 °C for 10 min, an aliquot (30 µL) of the mixture in the HDPE vial was removed and transferred to a NMR tube equipped with PTFE liner. ^{1}H and ^{19}F NMR spectra were recorded at −46 °C in a 300 MHz NMR spectrometer whose probe temperature was also set to −46 °C. An NMR sample containing CD_2Cl_2 and C_6F_6 was used once to set the shimming parameters for subsequent samples.

Tetraethylammonium chloride (0.25 eq., 25 mg, 0.15 mmol, 0.2 m in CD_2Cl_2) was added to the mixture in 30 min intervals. 15 min after each addition of tetraethylammonium chloride, an aliquot (30 µL) of the mixture in the HDPE vial was removed. In total, 2.0 eq. TEAC was added to the mixture of *p*-Tol-IF_2

^{1}H NMR spectrum of *p*-Tol-IF_2 at −46 °C

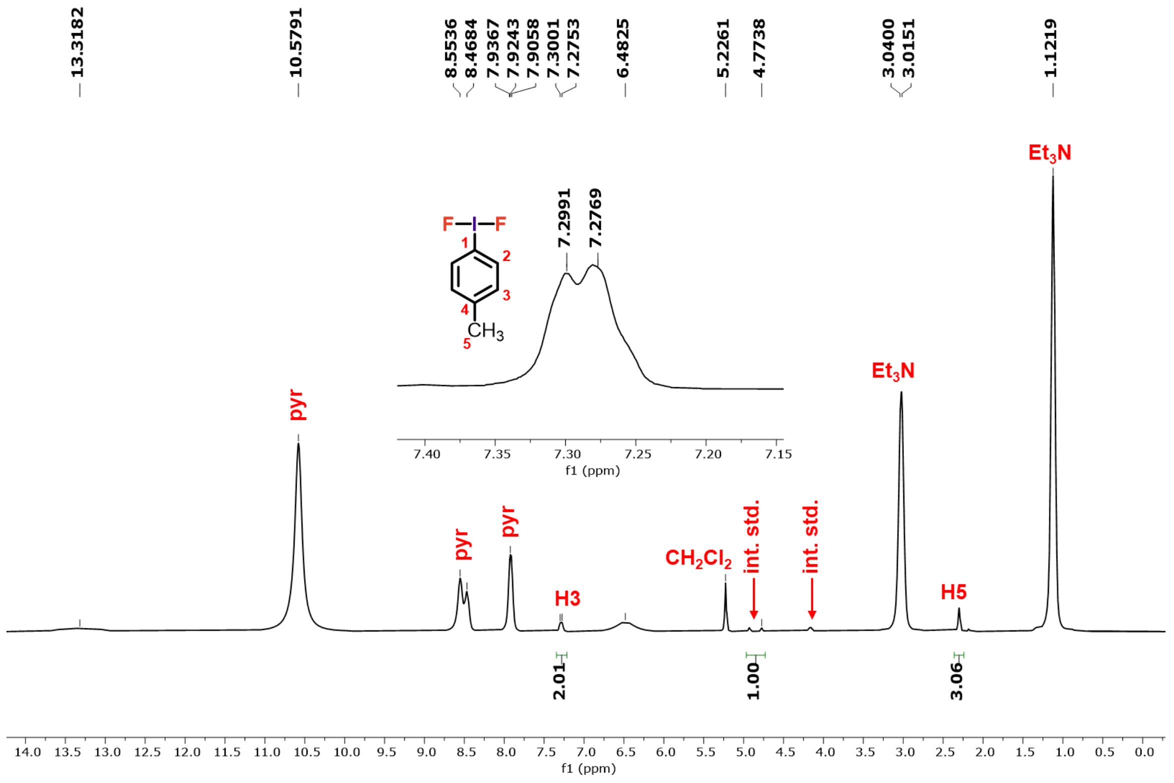

Figure S19 ^{1}H NMR spectrum of *p*-Tol-IF_2 in 5.6HF:amine/CD_2Cl_2 at −46 °C.

^{1}H NMR spectrum of *p*-Tol-IF$_2$ + 0.25 eq. Et_4NCl at −46 °C

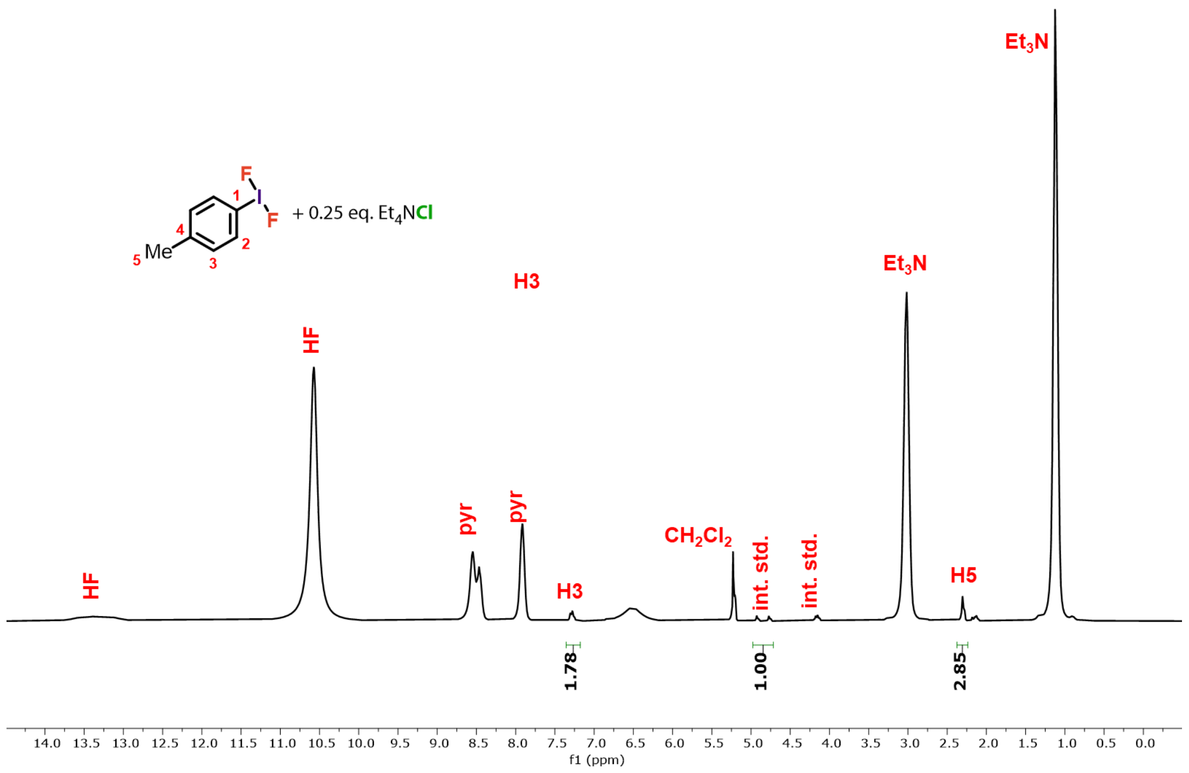

Figure S20 ^{1}H NMR spectrum of *p*-Tol-IF$_2$ + Et_4NCl (0.25 eq.) in 5.6HF:amine/CD_2Cl_2 at −46 °C. Zoomed-in region shows integrals of iodane peaks relative to each other. Full spectrum shows integrals of iodane peaks relative to ethyl fluoroacetate internal standard.

^{1}H NMR spectrum of *p*-Tol-IF$_2$ + 0.5 eq. Et$_4$NCl at −46 °C

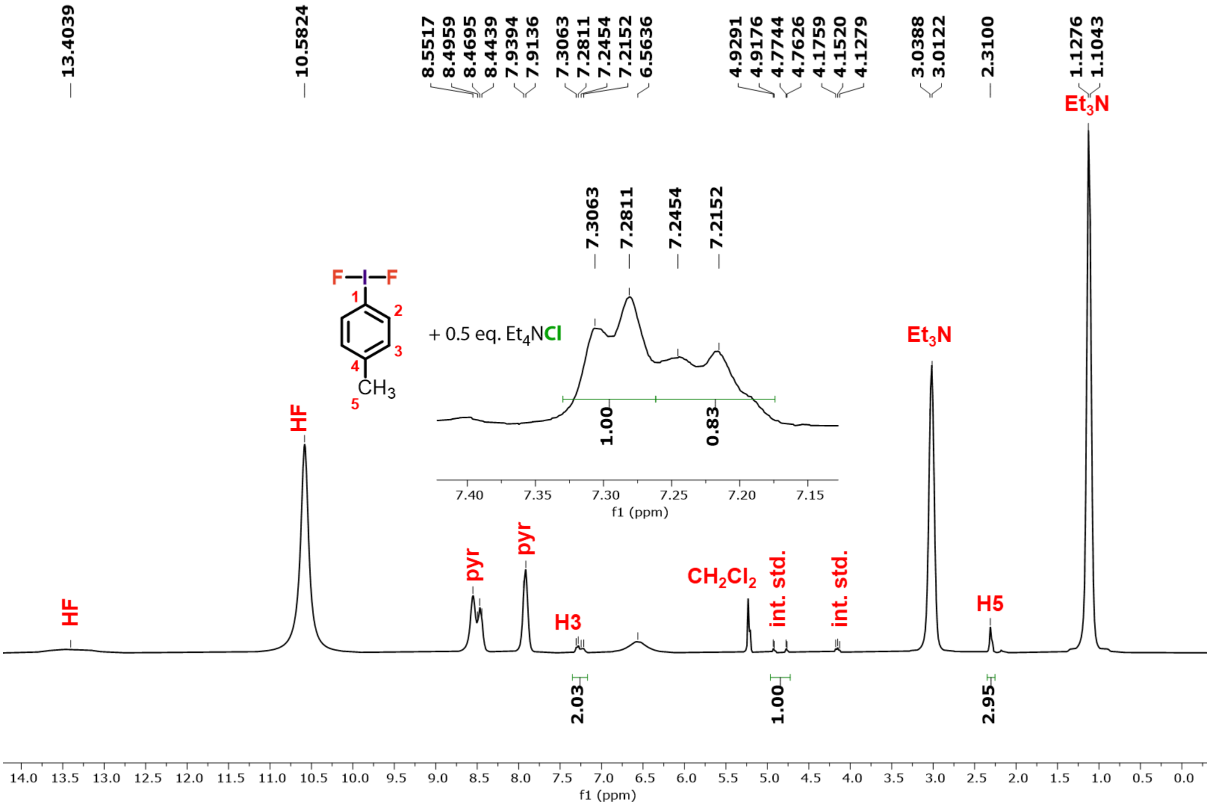

Figure S21 ^{1}H NMR spectrum of *p*-Tol-IF$_2$ + Et$_4$NCl (0.5 eq.) in 5.6HF:amine/CD$_2$Cl$_2$ at −46 °C. Zoomed-in region shows integrals of iodane peaks relative to each other. Full spectrum shows integrals of iodane peaks relative to ethyl fluoroacetate internal standard.

^{1}H NMR spectrum of *p*-Tol-IF$_2$ + 0.75 eq. Et$_4$NCl at −46 °C

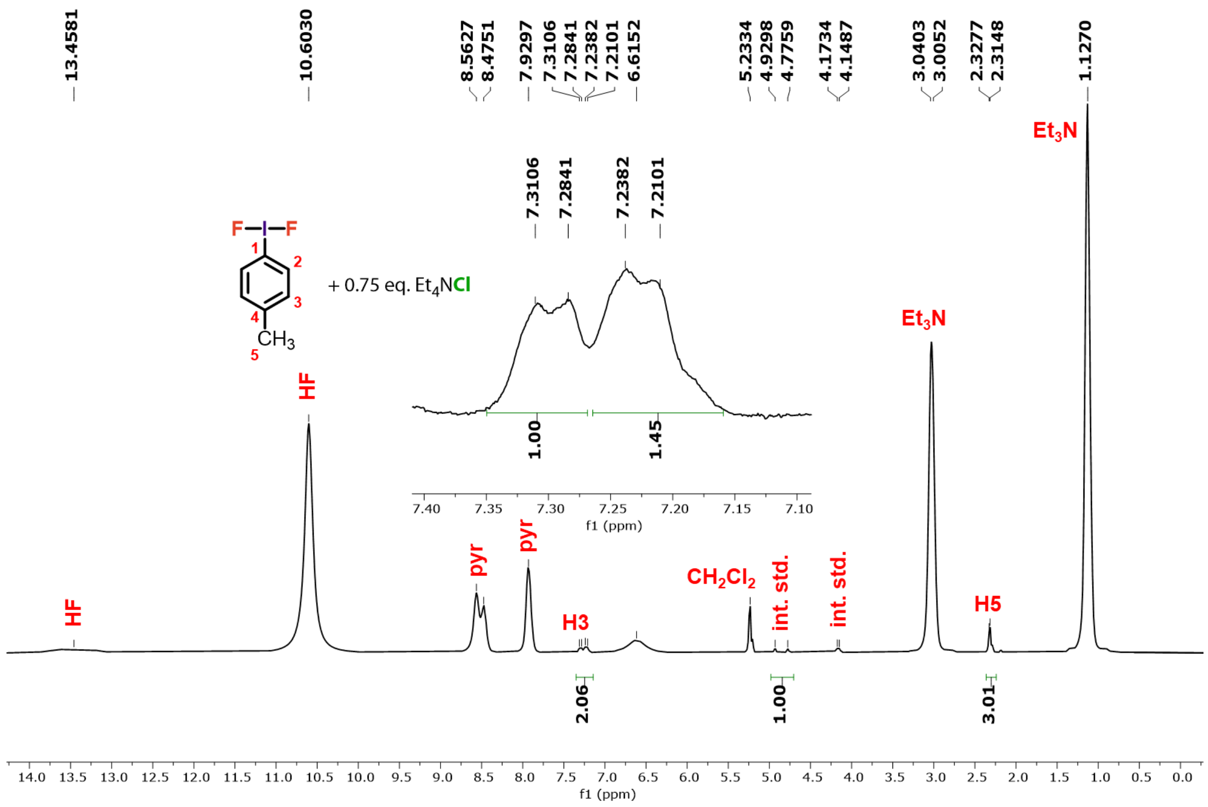

Figure S22 ^{1}H NMR spectrum of *p*-Tol-IF$_2$ + Et$_4$NCl (0.75 eq.) in 5.6HF:amine/CD$_2$Cl$_2$ at −46 °C. Zoomed-in region shows integrals of iodane peaks relative to each other. Full spectrum shows integrals of iodane peaks relative to ethyl fluoroacetate internal standard.

^{1}H NMR spectrum of *p*-Tol-IF_2 + 1.0 eq. Et_4NCl at −46 °C

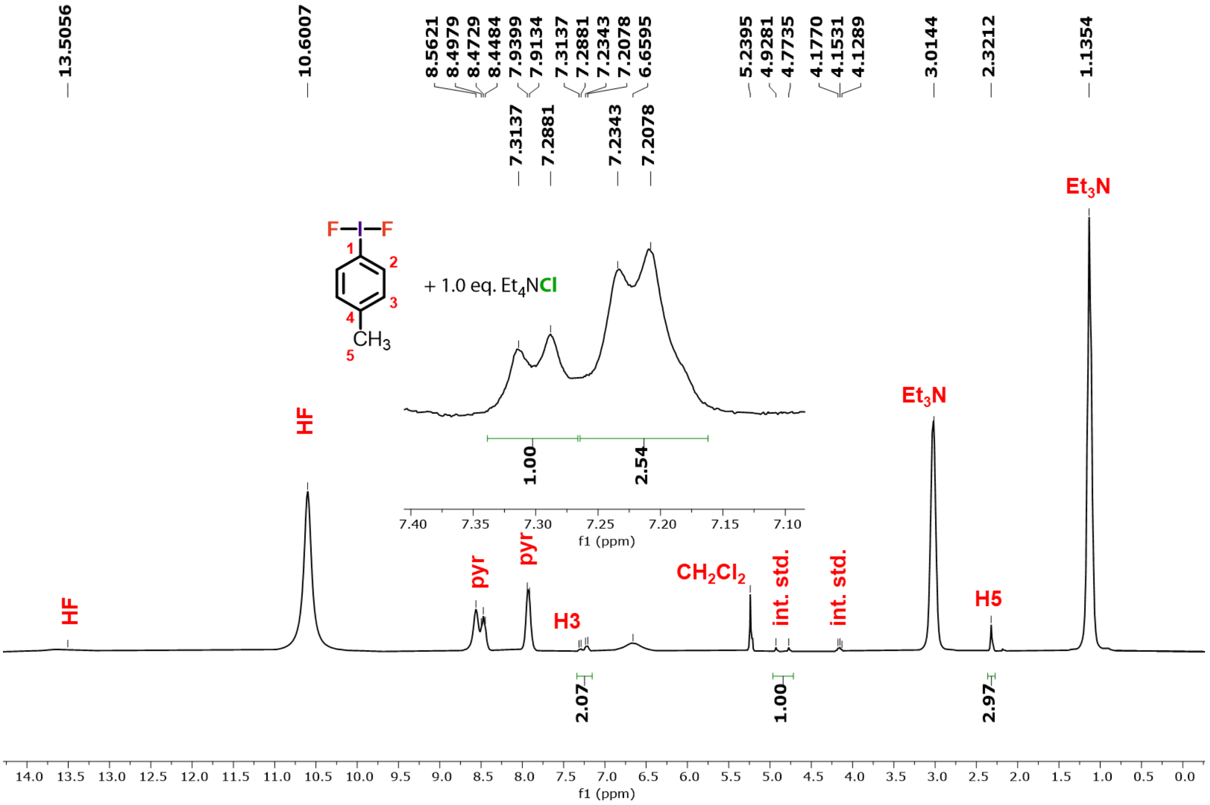

Figure S23 ^{1}H NMR spectrum of *p*-Tol-IF_2 + Et_4NCl (1.0 eq.) in 5.6HF:amine/CD_2Cl_2 at −46 °C. Zoomed-in region shows integrals of iodane peaks relative to each other. Full spectrum shows integrals of iodane peaks relative to ethyl fluoroacetate internal standard.

We do not report the ^{19}F NMR spectra for *p*-Tol-IF_2 + *n* eq. TEAC as we do the ^{1}H NMR spectra above. This is because the ^{19}F NMR signal attributed to the fluoride ligand of an iodane overlaps with the signal of the HF in the sample. Therefore, we are unable to measure any instructive integral data on these ^{19}F NMR signals.

^{1}H NMR of *p*-Tol-IF$_2$ and *p*-Tol-ICl$_2$ in 1:1 ratio

p-TolIF$_2$ (0.5 eq., 0.1 mmol, 0.5 mL of anodic compartment reaction mixture, withdrawn directly from divided cell via syringe) was transferred to a 100 mL HDPE vial equipped with a stirrer bar, was further diluted with CD_2Cl_2 (0.75 mL) and 5.6HF:amine stock solution (0.25 mL), dosed with ethyl fluoroacetate (29 μL, 0.3 mmol, 1.5 eq. wrt to total iodane in mixture) as internal standard (for monitoring by ^{1}H and ^{19}F NMR), capped with a Suba seal equipped with a venting needle and cooled to −46 °C whilst stirring. After fully cooling to −46 °C, *p*-Tol-ICl$_2$ (29 mg, 0.1 mmol, 0.1 M) in CD_2Cl_2 (1 mL) was added by syringe in one portion. After stirring for 5 min, an aliquot (30 μL) of the mixture in the HDPE vial was removed to record a ^{1}H NMR spectrum.

^{1}H NMR spectrum of *p*-Tol-IF$_2$ + *p*-TolICl$_2$ (1:1) at −46 °C

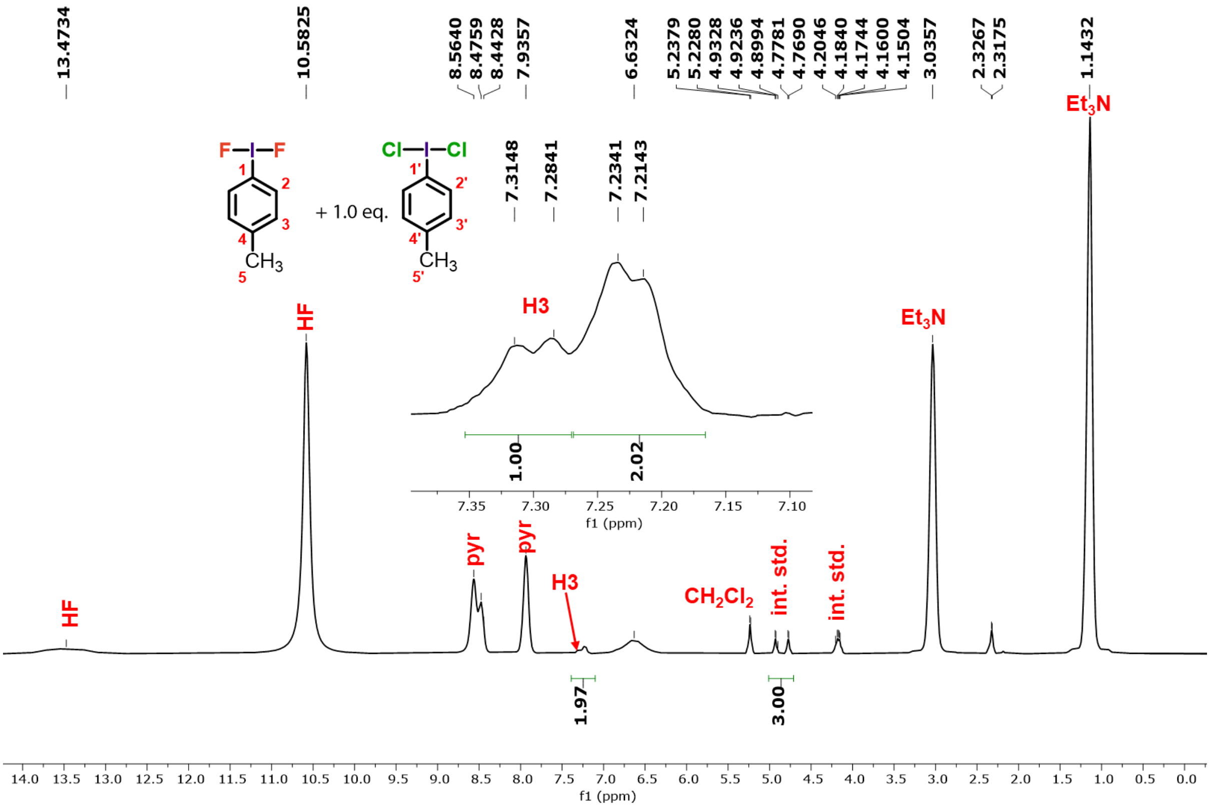

Figure S24 ^{1}H NMR spectrum of *p*-Tol-IF$_2$ (0.1 mmol, 1.0 eq.) and *p*-Tol-ICl$_2$ (0.1 mmol, 1.0 eq.) in 5.6HF:amine/CD_2Cl_2 at −46 °C. Total iodane in mixture is 0.2 mmol relative to internal standard.

^{1}H NMR of *p*-Tol-ICl_2

To a 100 mL HDPE vial equipped with a stirrer bar was added ethyl fluoroacetate (29 μL, 0.3 mmol, 1.5 eq.), CD_2Cl_2 (1.25 mL) and 5.6HF:amine stock solution (0.75 mL). The vial was capped with a Suba seal equipped with a venting needle and cooled to −46 °C whilst stirring. Then *p*-Tol-ICl_2 (58 mg, 0.2 mmol, 0.2 M) in CD_2Cl_2 (1 mL) was added by syringe in one portion. After stirring for 5 min, an aliquot (30 μL) of the mixture in the HDPE vial was removed to record a ^{1}H NMR spectrum.

^{1}H NMR spectrum of *p*-Tol-ICl_2 at −46 °C

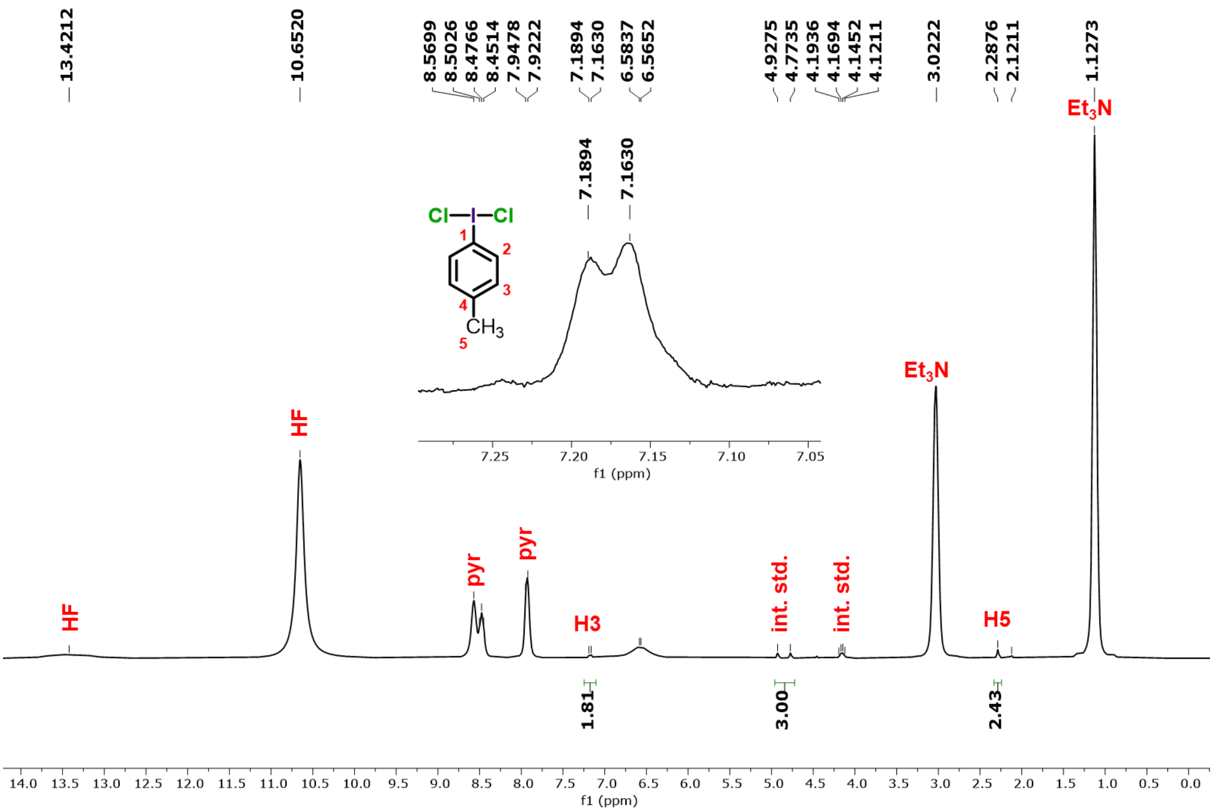

Figure S25 ^{1}H NMR spectrum of *p*-Tol-ICl_2 in 5.6HF:amine/CD_2Cl_2 at −46 °C.

Stacked ^{1}H NMR spectra of *p*-Tol-IF_2, *p*-Tol-ICl_2, *p*-Tol-IF_2 + 1.0 eq. Et_4NCl and *p*-Tol-IF_2 + 1.0 eq. *p*-Tol-ICl_2

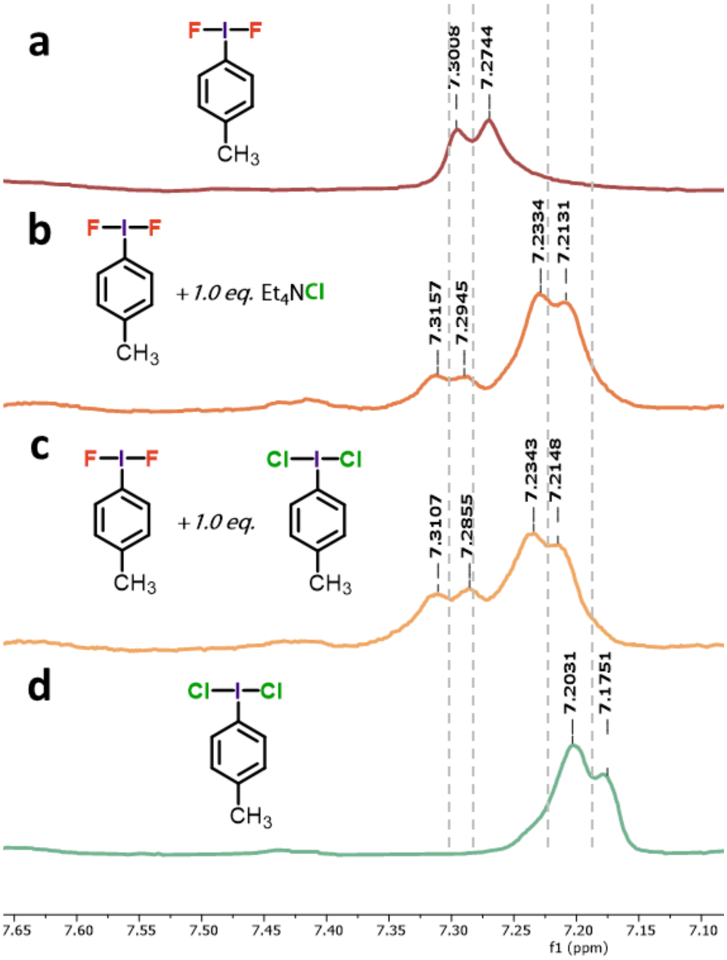

Figure S26 (a) ^{1}H NMR spectrum of *p*-Tol-IF_2 recorded in 5.6HF:amine/CD_2Cl_2; **(b)** ^{1}H NMR spectrum of *p*-Tol-IF_2 + Et_4NCl (1.0 eq.) recorded in 5.6HF:amine/CD_2Cl_2; **(c)** ^{1}H NMR spectrum of *p*-Tol-IF_2 + *p*-Tol-ICl_2 (1.0 eq.) recorded in 5.6HF:amine/CD_2Cl_2; **(d)** ^{1}H NMR spectrum of *p*-Tol-ICl_2 recorded in 5.6HF:amine/CD_2Cl_2. All spectra were recorded at -46 °C using ethyl fluoroacetate as internal standard.

Stacked ^{1}H NMR spectra of *p*-Tol-IF_2 + Et_4NCl (0-1.0 eq.)

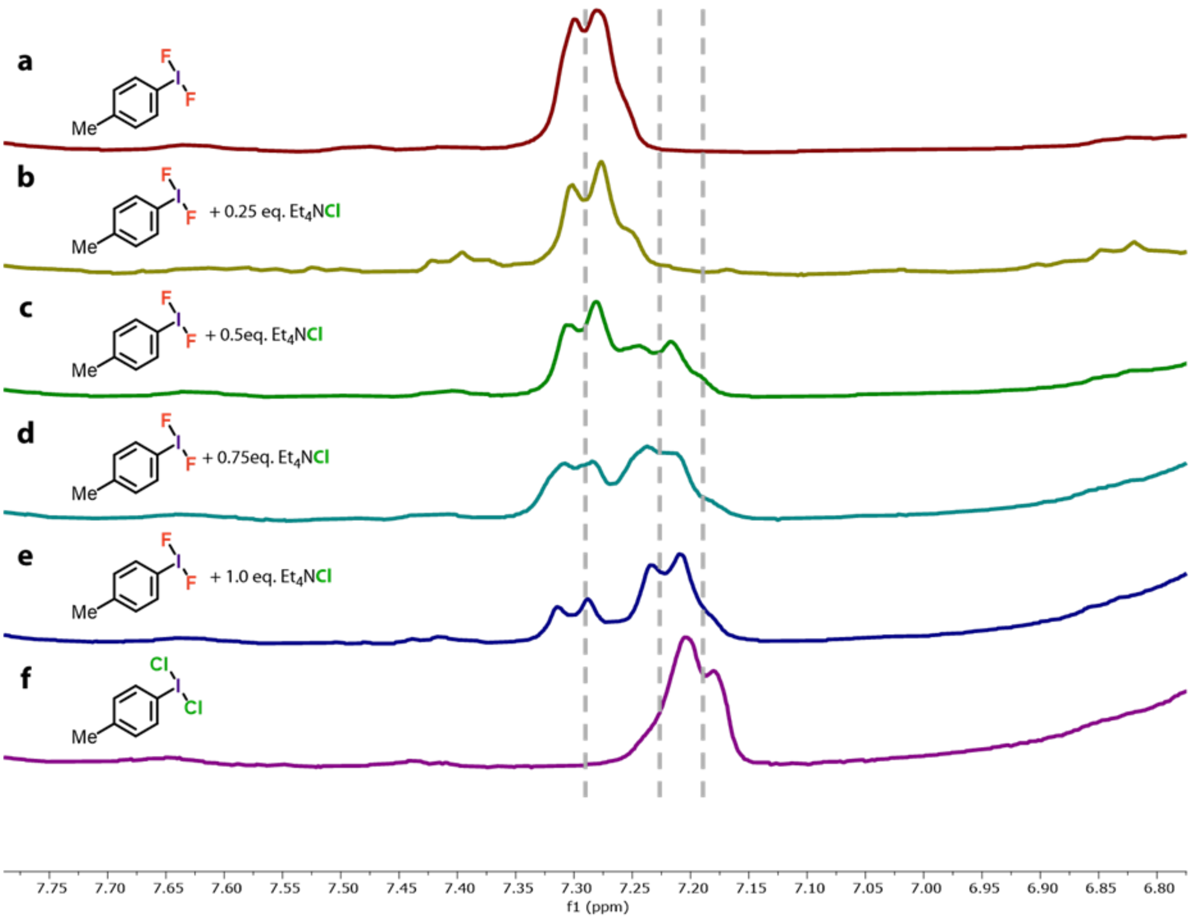

Figure S27 (a) ^{1}H NMR spectrum of *p*-Tol-IF_2 in 5.6HF:amine/CD_2Cl_2; **(b-e)** ^{1}H NMR spectra of *IF_2* + Et_4NCl (0.25-1.0 eq.) in 5.6HF:amine/CD_2Cl_2; **(f)** ^{1}H NMR spectrum of *p*-Tol-ICl_2 in 5.6HF:amine/CD_2Cl_2 All ^{1}H NMR spectra were recorded at −46 °C using ethyl fluoroacetate as internal standard.

Mimicking HF equivalents *syn*-chlorofluorination regime with 5.6HF:amine

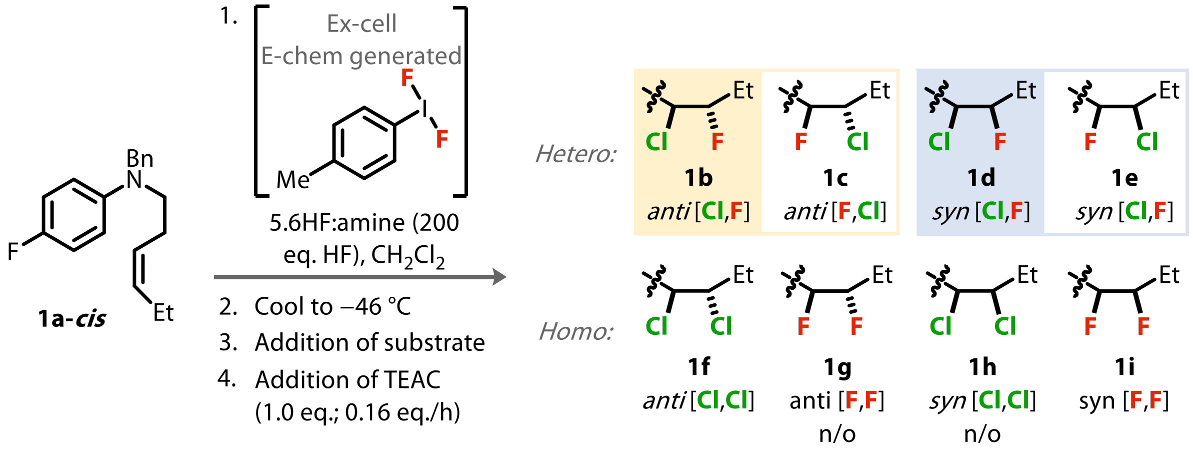

To a 50 mL HDPE vial were added *p*-TolIF$_2$ (1.0 eq., 0.2 mmol, 1 mL of anodic compartment reaction mixture, withdrawn directly from divided cell via syringe), CH_2Cl_2 (3 mL) and additional 5.6HF:amine stock solution (0.75 mL). This vial was capped with a Suba-seal, a venting needle placed through the Suba-seal, and the vial then cooled to −46 °C. **1a-*cis*** (0.6 м, 1.0 eq., 0.2 mmol, 57 mg) in CH_2Cl_2 (0.33 mL) was added and then tetraethylammonium chloride (TEAC) (0.2 м, 1.0 eq., 0.2 mmol, 33 mg) in CH_2Cl_2 (1 mL) was added *via* syringe pump (0.16 eq./min; 0.16 mL/min). After stirring overnight, the reaction mixture was quenched with 300 mL of cold (0 °C) saturated aqueous $NaHCO_3$ solution until the aqueous layer measured pH 7, and then the reaction mixture was stirred for 1 hour. The mixture was extracted into CH_2Cl_2, dried with Na_2SO_4, filtered, and concentrated *in vacuo*. The product was then purified *via* silica-gel column chromatography. The residue was dissolved in $CDCl_3$ (2 mL), to this solution was added hexafluorobenzene (23.2 µL, 1.0 eq.) and a ^{19}F NMR spectrum of the sample was recorded to measure a ^{19}F NMR yield.

Table S13 NMR yields measured for chlorofluorination of 1a-*cis* with the number of HF equivalents increased to approximately 200 with 5.6HF:amine stock solution.

1b	1c	1d	1e	1f	1g	1h	1i
40	3	0	0	0	0	0	7

Variation of HF:amine ratio in Olah conditions

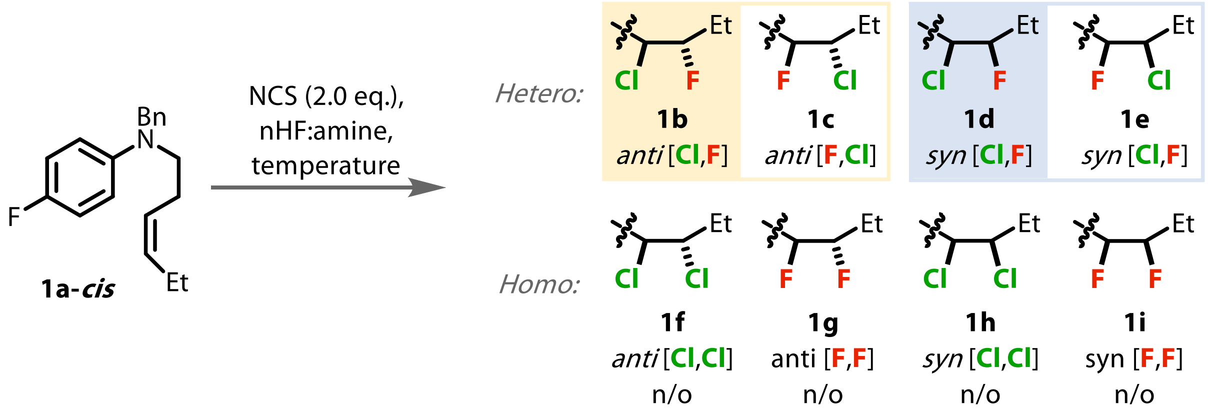

To a 100 mL HDPE vial was added, HF:amine (1 mL) and NCS (2.0 eq., 0.4 mmol, 27 mg). To this solution was added **1a-*cis*** (0.04 м, 1.0 eq., 0.2 mmol, 57 mg) in tetrahydrofuran (5 mL) over 10 minutes. After stirring overnight, the reaction mixture was quenched with 100 mL of cold (0 ºC) saturated aqueous $NaHCO_3$ solution. This stirred for 1 hour until the aqueous layer measured pH 7. The mixture was extracted into CH_2Cl_2, dried with Na_2SO_4, filtered, and concentrated *in vacuo*. To the mixture was then added $CDCl_3$ (2 mL) and hexafluorobenzene (1.0 eq., 0.2 mmol, 23.2 µL), at which point the ^{19}F NMR was measured. The NMR yield was then measured by comparing the integration of the fluorine signal of the product to that of the internal standard (δ = -161 ppm).

Table S14 Product NMR yields of Olah's chlorofluorination varying the HF:amine source.

Entry	HF:amine	HF equivalents	Temperature/ −46 °C	1b + 1c /% (rr)	1d + 1e /%
1	Pyr•9HF	54	25	33 (6:1)	0
2	Pyr•9HF	54	−46	39 (10:1)	0
3	Pyr•9HF	108	25	0	0
4	Pyr•9HF	108	−46	0	0
5[a]	Pyr•9HF	192	25	41 (5.8:1)	0
6[a]	Pyr•9HF	192	−46	38 (18:1)	0
7	Pyr•9HF	405	25	48 (11:1)	0
8	Pyr•9HF	405	−46	0	0
9	7HF:amine	204	−46	<5	0
10	5.6HF:amine	108	25	0	0
11	5.6HF:amine	108	−46	0	0

[a] CH_2Cl_2 (5 mL) was used instead of THF.

The regioselectivies observed under Olah's conditions with different HF:amine ratios are variable and indicate that this work's *anti*-chlorofluorination reaction either does

not proceed via a discrete chloronium intermediate or other interactions occur in the transition state for the nucleophilic addition that are absent in electrophilic chlorine/nucleophilic fluoride approaches.

Studies into chloride nucleophilicity in 5.6HF:amine and 7HF:amine

Mass Percentage of HF in Stock Reagents

Mass percentage of HF in stock reagents was determined via a known literature procedure.[20] A solution of KOH (1.96 g) in deuterated water was produced (10 ml, 3.5 м). A 0.5 ml aliquot of this solution was added to a 25 ml-HDPE vial equipped with a stir-bar. The aliquot was then cooled to 0 °C before adding 25 μL of the HF reagent stock solution and stirring for five minutes, followed by 15 μL of ethyl trifluoroacetate. The solution was then transferred to a fluorinated ethylene propylene (FEP) NMR tube liner, which was inserted into an NMR tube charged with 0.1 ml trichlorofluoromethane. ^{1}H and ^{19}F NMR were carried out to determine the mass percentage.

Fluoride Concentration Calibration Curve and Measurements

a) Synthesis of Ag/AgOTf Reference Electrode and Ag|AgF ion ISE

The method for determining the fluoride concentration within all the HF mixtures was adapted from one that was used to measure fluoride concentrations in organic media.[21] The open circuit potential (V_{ocp}) was measured using the Ag/AgOTf double-junction reference electrode described in the literature to prevent the precipitation of fluoride. A diagram for this reference electrode is shown in **Figure S28**. When the reference electrode is not in use, it is stored in a 0.1 м solution of tetraethylammonium perchlorate (TEAP) in propylene carbonate.

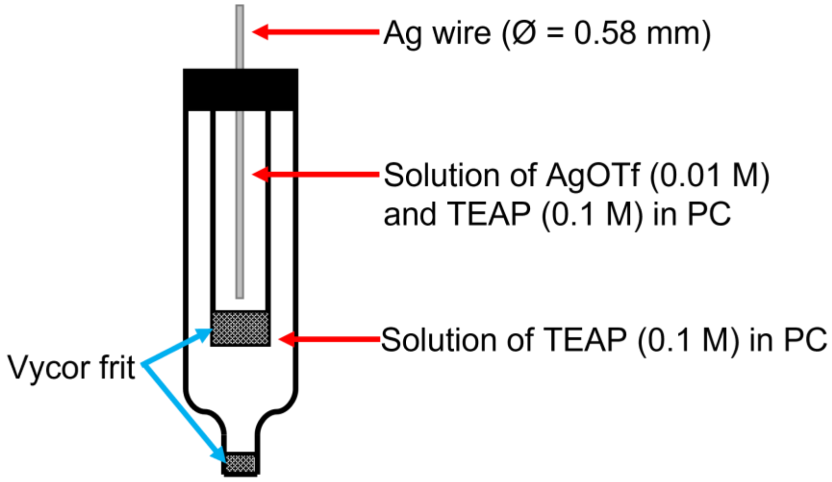

Figure S28 A diagram of the Ag/AgOTf reference electrode that was used for all the fluoride measurements.

For the fluoride ion-selective electrode (ISE), silver wire was submerged in Olah's reagent for five days. The wire was held in place by pushing it through a suba-seal, which was able to cover the top of a 25 ml-HDPE vial containing the Olah's reagent. After five days, a brown-black precipitate was present on the surface of the wire (**Figure S29**), indicating the synthesis of silver fluoride upon the surface of the wire.

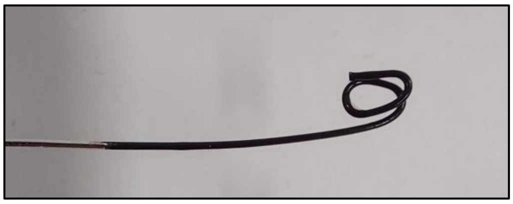

Figure S29 The Ag|AgF ISE synthesised by subjecting silver wire to Olah's reagent for five days.

b) Calibration Curve for Fluoride Measurements

The calibration curve was determined for fluoride concentrations in the range 10^{-2} M to 10^{-4} M, as this has been determined to be the range that the ISE can function successfully.[21] The calibration curve is shown in **Figure S30**. This was produced by diluting a 10^{-2} M solution of tetramethylammonium with a 0.1 M solution of tetraethylammonium perchlorate in PC, with aliquots of a 0.1 M TEAP in PC solution being added between each open circuit potential measurement.

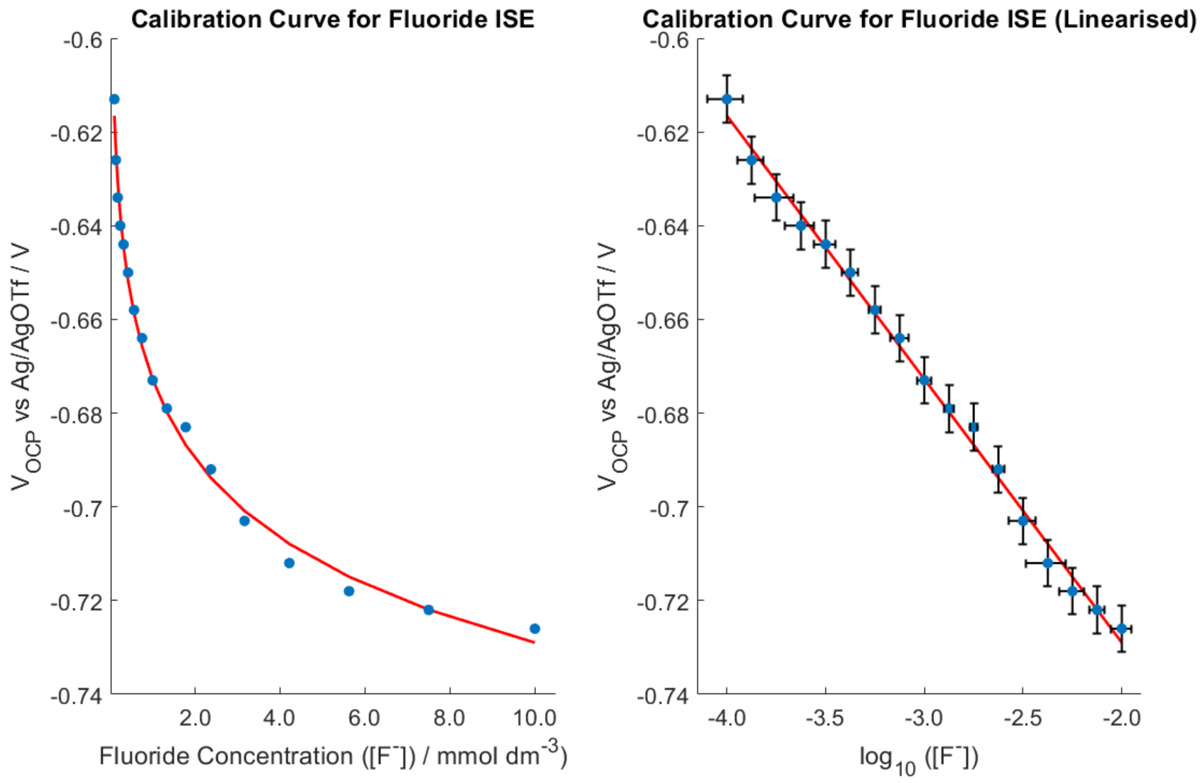

Figure S30 The calibration curve for the fluoride measurements when considering solutions with concentrations between 10-4 M and 10-2 M. **a**) The collected data along with the curve of best fit. **b**) The linearised form of the same data, where the equation of the line is given in **Equation 1.4**. N = 3 for all data points. Data are presented as the arithmetic mean value ± error values derived through combination of errors fitted into **Equation 1.4**. Error arises from the measurement of the V_{OCP} (± 0.0005 V) and the error in fluoride anion concentration (± 0.00008 M).

Data fitting was performed via MATLAB software using the function described in **Equation 1.4**, where [F⁻] refers to the concentration of fluoride within the solution.

$$V_{\mathrm{OCP}} = -0.8415 - 0.0562 \log_{10}[\mathrm{F}^-] \qquad \textbf{Equation 1.4}$$

c) Measuring Fluoride Concentrations Involving HF Solutions

To measure the fluoride concentrations, a 150 µL aliquot of the HF cluster was mixed with 5 mL of PC for five minutes. The open circuit potential vs the Ag/AgOTf reference electrode was then measured using the AutoLab M101. The open circuit potentials measured were then converted into fluoride concentrations, and then scaled so that the concentration of fluoride in the neat HF clusters could be determined.

General procedure for NMR kinetics

A HF•amine (either 5.6 HF•amine (7.5 mL) or 7.0 HF•amine (7.5 mL)) mixture was first prepared in a cooled 20 mL HDPE vial and was allowed to warm up to room temperature. Then a pre-prepared 1.0 M solution of substrate (2.5 mL, 2.5 mmol) in CH_2Cl_2 was added to the HF•amine solution at room temperature. Aliquots of the reaction mixture were initially taken every five minutes for the first thirty minutes, followed by an aliquot for every 30 minutes for the next 90 minutes, followed by aliquots taken every hour if required. The internal standard (5% tetramethylsilane (TMS) in CH_2Cl_2) is separate from the reaction mixture and is achieved using a coaxial system (illustrated in **Figure S31** below). ^{1}H NMR were recorded at each time point, and the rate constant was calculated from the change in the integrated area of a signal associated with both the substrate and the product for a given reaction. For the reaction of 4-nitrobenzyl bromide with fluoride anions, the benzylic chemical shifts of both the substrate and the fluorinated product were monitored throughout the reaction to determine the rate constant.

When determining the rate of reaction involving butyl mesylate at room temperature, tetraethylammonium chloride (TEAC; 414 mg, 2.5 mmol, 1 eq.) was added to the HF mixture before adding the substrate to ensure a homogenous solution. Characteristic signals for butyl mesylate, 1-chlorobutane and 1-fluorobutane were analysed to determine the rate constant for this S_N2 reaction.

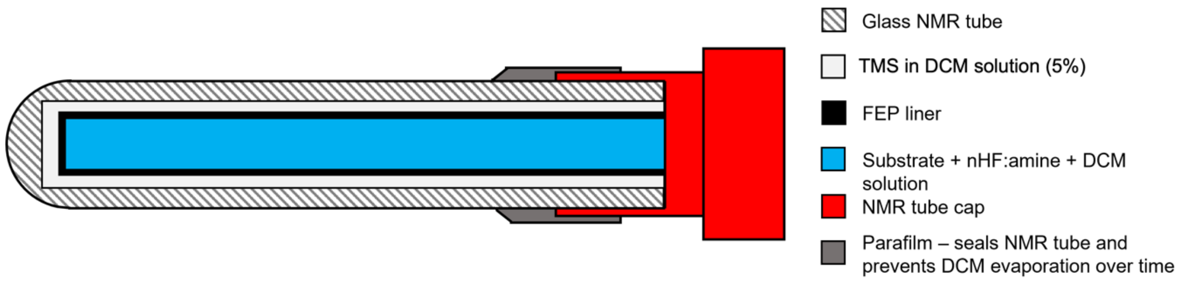

Figure S31 A cross section of an NMR tube used for monitoring the kinetics of a HF reaction.

The concentration of any given species at a given time is calculated using **Equation 1.5**

$$C_x = \frac{I_x}{I_{int}} * \frac{N_{int}}{N_x} * C_{int}$$ **Equation 1.5**

where C_x is the concentration of species x, I_x is the integrated area of the signal belonging to the compound of interest, I_{int} is the integrated area of the signal associated with the internal standard (5.0% TMS solution in DCM; chemical shift of 0.0ppm), N_{int} is the number of nuclei associated with the signal of the internal standard (N_{int} = 12), N_x is the number of nuclei associated with the signal of the desired species (N_x = 2 for substrates) and C_{int} is the concentration of the internal standard. The rate constant was calculated using a genetic algorithm provided by the software package COPASI.[22]

Substrate synthesis
Butyl mesylate

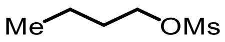

A solution of butan-1-ol (1.0 eq., 1.00 g, 13.4 mmol) in CH_2Cl_2 (10 mL) was cooled to 0 °C. To this mixture, triethylamine (1.5 eq., 2.79 mL, 20.0 mmol) was added, and the mixture was stirred for 10 minutes. Methanesulfonyl chloride (1.5 eq., 1.55 mL, 20.0 mmol) was then added dropwise to the cooled mixture. The reaction was then allowed to warm up to room temperature and was stirred for a further 90 minutes. The reaction mixture was then quenched with methanol (50 mL) and concentrated under vacuum. The resulting white solid was then dissolved in EtOAc (50 mL) and washed with aq. HCl (1 M, 50 mL), followed by saturated aq. $NaHCO_3$ solution (50 mL). The organic layer was collected, dried over $MgSO_4$, filtered and concentrated *in vacuo* to afford butyl mesylate as a yellow oil (1.55 g, 76%).

^{1}H NMR (400 MHz, $CDCl_3$): 4.18 (t, *J* = 6.51 Hz, 2H), 2.95 (s, 3H), 1.66 (m, 2H), 1.39 (m, 2H), 0.89 (t, *J* = 7.40 Hz, 3H).

^{13}C {^{1}H} NMR (101 MHz, $CDCl_3$): 69.9, 37.3, 31.0,18.7, 13.5.

These data are consistent with those previously reported.[23]

COPASI modelling of reaction of *n*-butyl mesylate in 5.6HF:amine

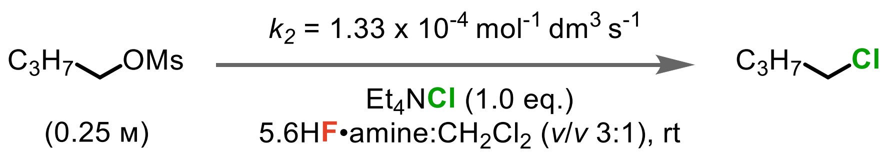

Scheme S1 S_N2 reaction of n-butyl mesylate with tetraethylammonium chloride (TEAC) in 5.6HF:amine, whose kinetic data are modelled with COPASI to output rate constants.

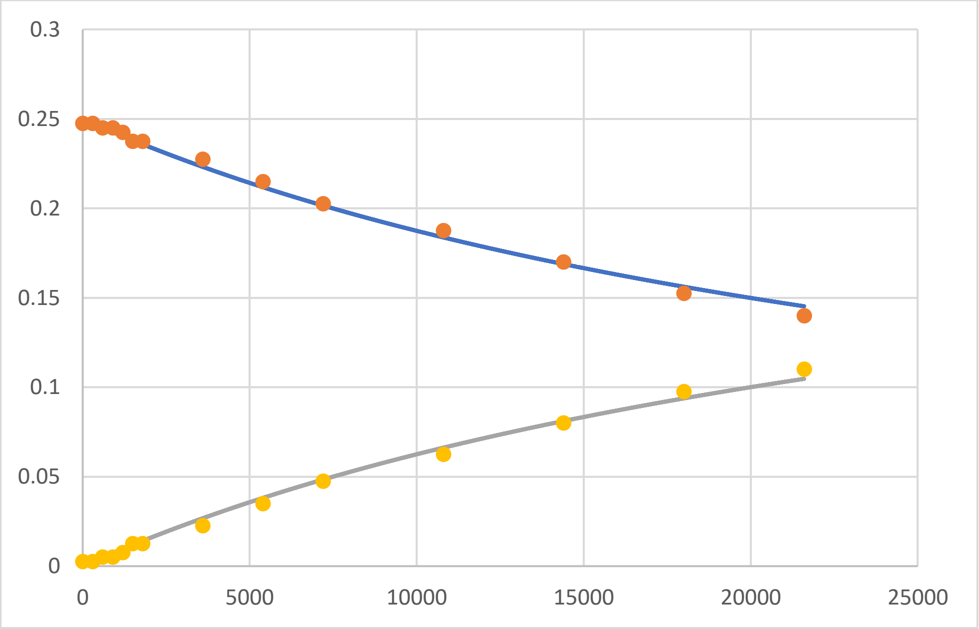

Figure S32 Plot of concentration of n-butyl mesylate (blue line) and 1-chlorobutane (grey line) against time for the reaction in **Scheme S1**.

COPASI modelling of reaction with *n*-butyl mesylate in 7HF:amine

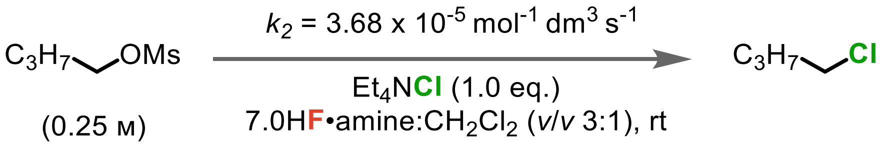

Scheme S2 S_N2 reaction of n-butyl mesylate in 7HF•amine, whose kinetic data are modelled with COPASI to output rate constants.

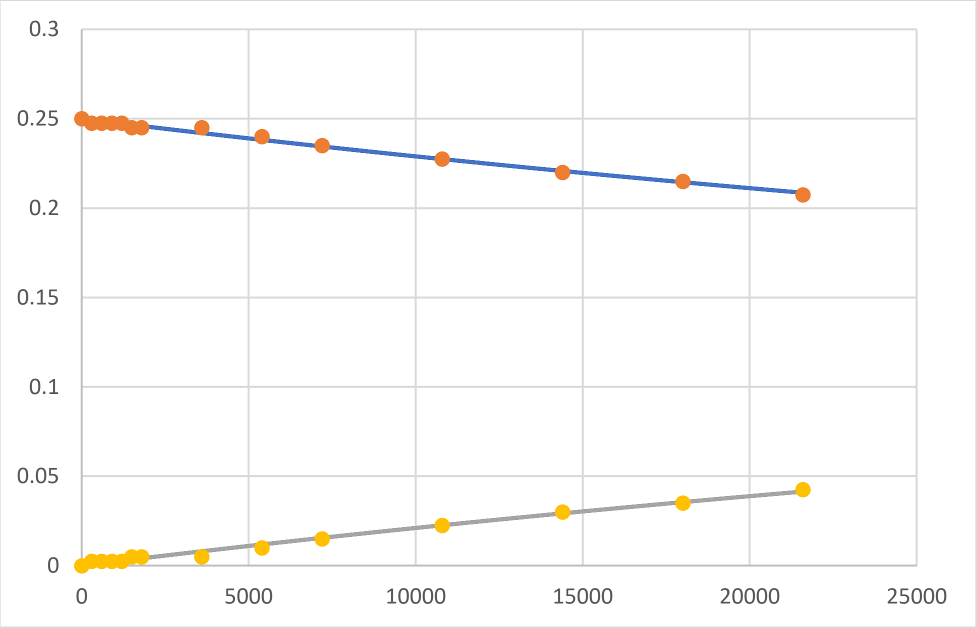

Figure S33 Plot of concentration of n-butyl mesylate (blue line) and 1-chorobutane (grey line) against time for the reaction in **Scheme S2.**

Back to top

COPASI modelling of reaction of 4-nitrobenzyl bromide in 5.6HF•amine

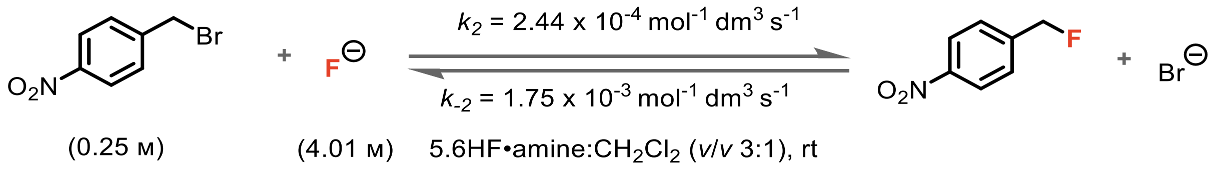

Scheme S3 S_N2 reaction of 4-nitrobenzyl bromide in 7.0 HF•amine, whose kinetic data are modelled with COPASI to output rate constants.

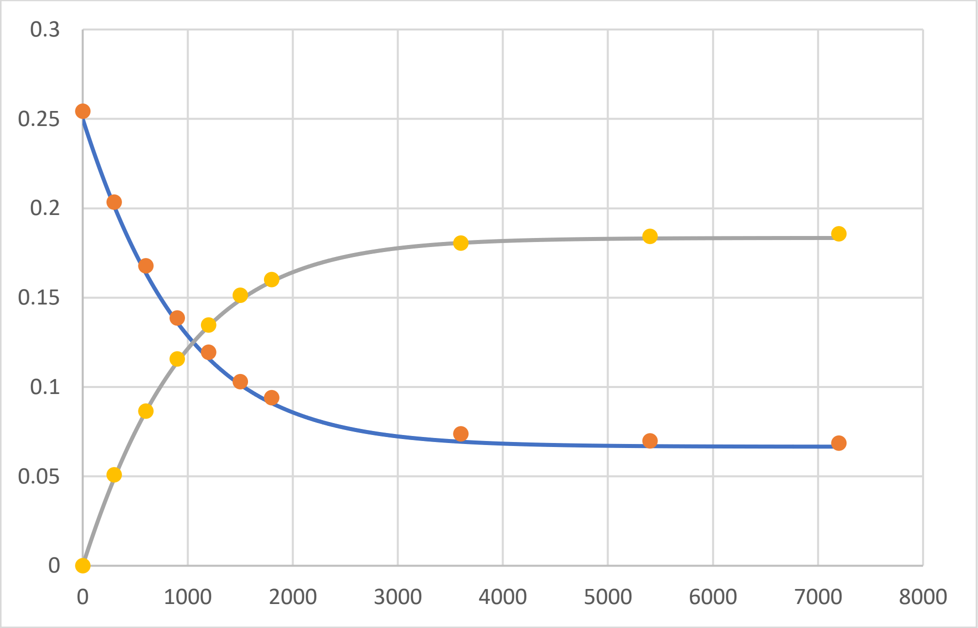

Figure S34 Plot of concentration of 4-nitrobenzyl bromide (blue line) and 4-nitrobenzyl fluoride (grey line) against time for the reaction in **Scheme S5**.

COPASI modelling of reaction with 4-nitrobenzyl bromide in 7HF•amine

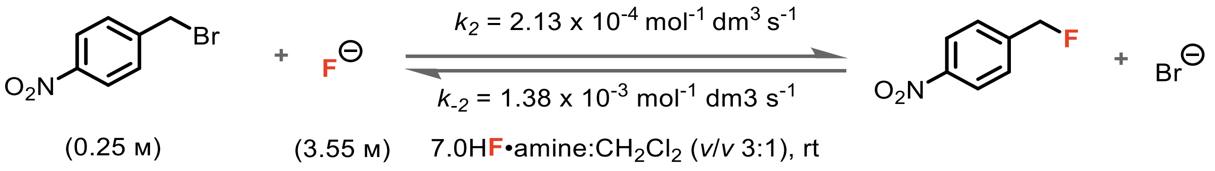

Scheme S4 S_N2 reaction of 4-nitrobenzyl bromide in 7HF•amine, whose kinetic data are modelled with COPASI to output rate constants.

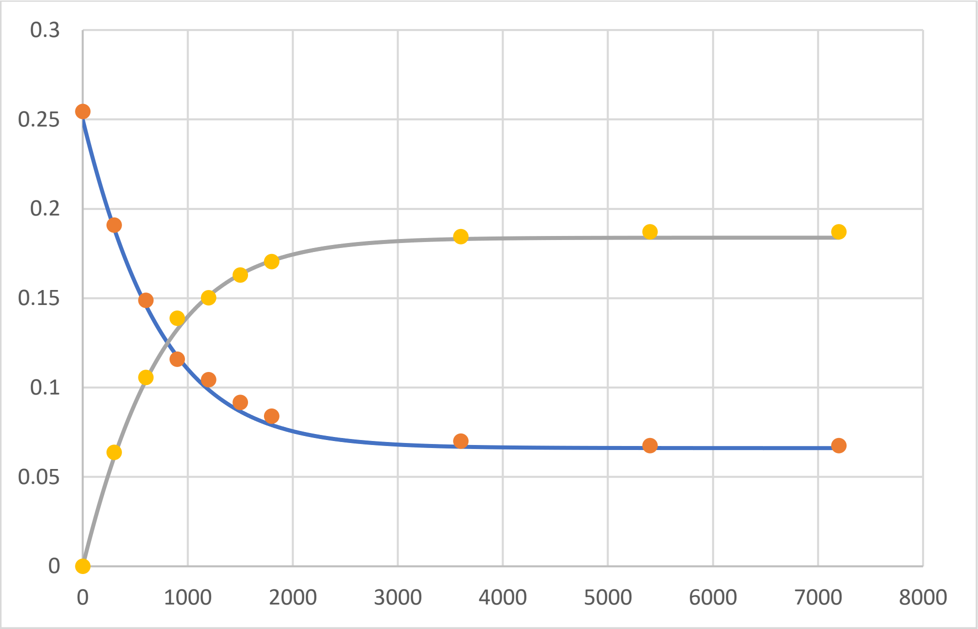

Figure S35 Plot of concentration of 4-nitrobenzyl bromide (blue line) and 4-nitrobenzyl fluoride (grey line) against time for the reaction in **Scheme S4**.

Mechanism that explains formation of three diastereomers of 35b

Proposed mechanism delivering three observed diastereomers

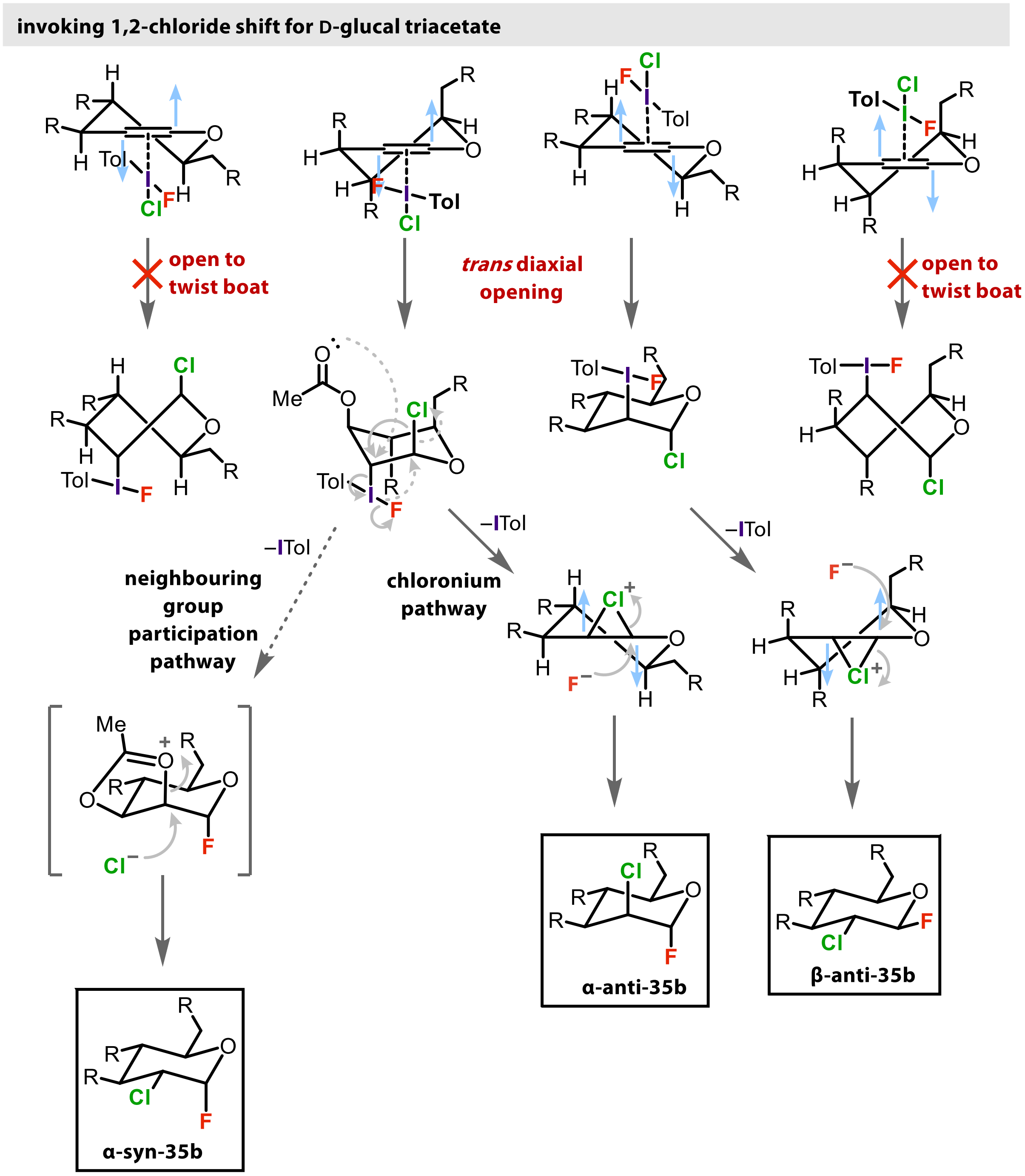

Figure S36 Mechanistic pathways that are consistent with the reaction outcome of the anti-chlorofluorination of substrate **35a**.

If the I(III)-alkene complex undergoes nucleophilic addition of chloride at the C1 carbon, then two diastereomers result from *trans*-diaxial ring opening. From only one of the diastereomers can **β-anti-35b** be produced from a formal 1,2-chloride shift via a chloronium, which is trapped out by fluoride. From the other diastereomer, two 1,2-chlorofluoride diastereomers can be produced if neighbouring group participation of

the C3 acetyl group is invoked. The acetyl group is poised to displace the I(III) moiety, whose fluoride ligand could feasibly displace the chlorine at C1. The chloride could then feasibly displace the C−O bond in the dioxolenium intermediate to give the observed **α-syn-35b** diastereomer. The 1,2-chloride shift pathway from the same 1,2-I^{III},Cl intermediate would give **α-anti-35b**. We propose that this mechanism involving a nucleophilic addition of chloride first followed by a formal 1,2-chloride shift via a chloronium is the only mechanism consistent with the outcome of the *anti*-chlorofluorination of **3,4,6-tri-*O*-acetyl-ᴅ-glucal** and is our proposed general mechanism for our reported *anti*-chlorofluorination of alkenes. Other mechanistic pathways cannot account for the formation of the **α-syn-35b** diastereomer.

Inconsistent mechanism: direct chloronium formation

Chloronium intermediate from D-glucal triacetate

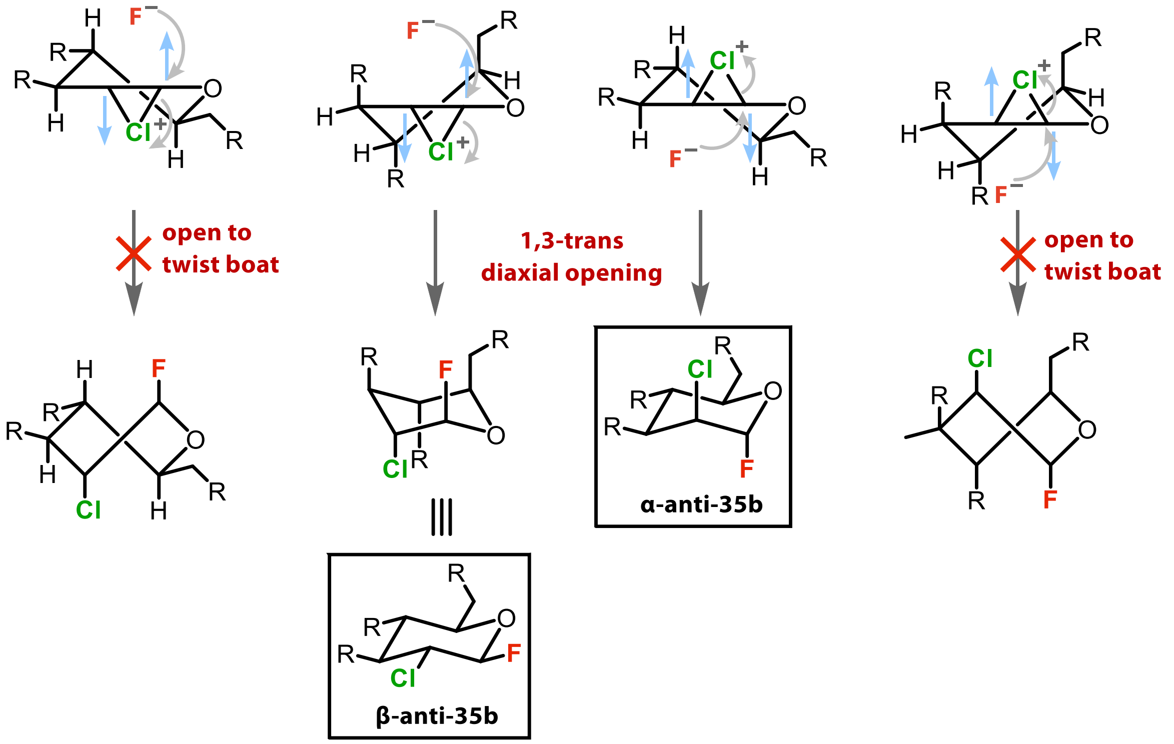

✘ **No opportunity for neighbouring group participation of C3 OAc group possible**

Figure S37 Mechanistic pathways that are inconsistent with the reaction outcome of the anti-chlorofluorination of substrate **35a**, as the direct chloronium formation does not afford the opportunity for neighbouring group participation of the C3 acetyl group.

If a discrete chloronium is formed, only two transition states for 1,3-*trans* diaxial opening with fluoride are possible. Anichmeric assistance of the C3 acetyl group could occur, but attack of chloride would lead to retention and reformation of **β-*anti*-35b**.

Inconsistent mechanism: *syn*-1,2-fluoro-λ3-iodanation

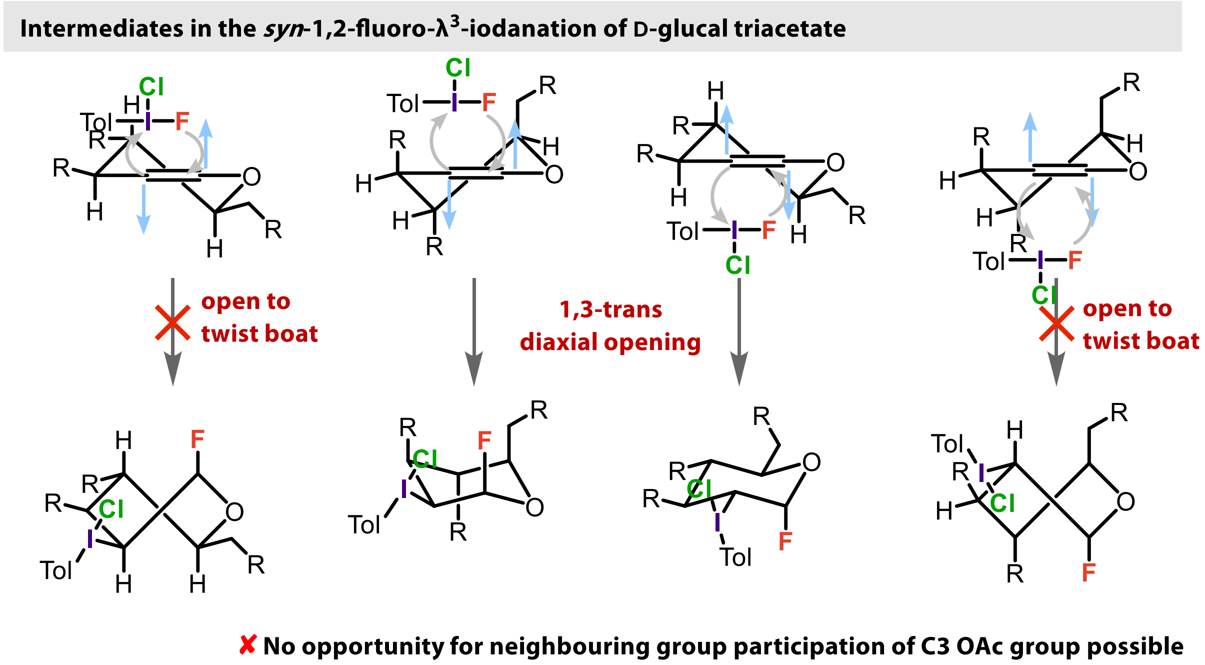

Figure S38 *Syn*-1,2-fluoro-λ3-iodanation mechanistic pathways that are inconsistent with the reaction outcome of the anti-chlorofluorination of substrate **35a**, as no intermediate places the C–I bond in the correct alignment for nucleophilic displacement by the C3 acetyl group for neighbouring group participation.

Inconsistent mechanism: *syn*-1,2-chloro-λ^3-iodanation

Intermediates in the *syn*-1,2-chloro-λ^3-iodanation of D-glucal triacetate

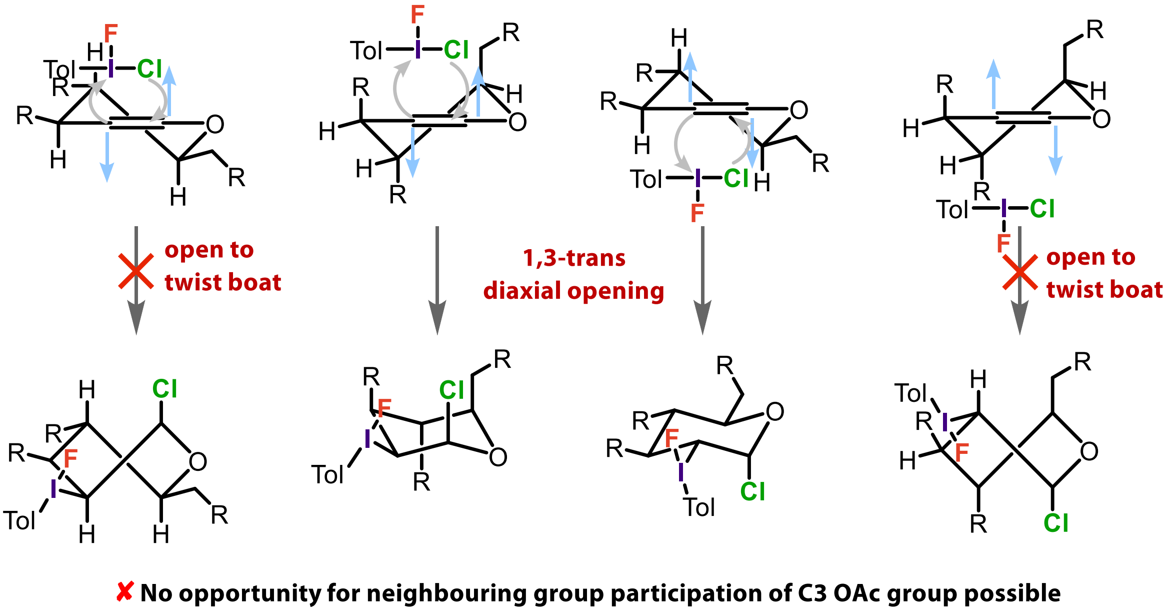

Figure S39 *Syn*-1,2-chloro-λ^3-iodanation mechanistic pathways that are inconsistent with the reaction outcome of the anti-chlorofluorination of substrate **35a**, as no intermediate places the C–I bond in the correct alignment for nucleophilic displacement by the C3 acetyl group for neighbouring group participation.

Variation of HF:amine in difluorination at −46 °C

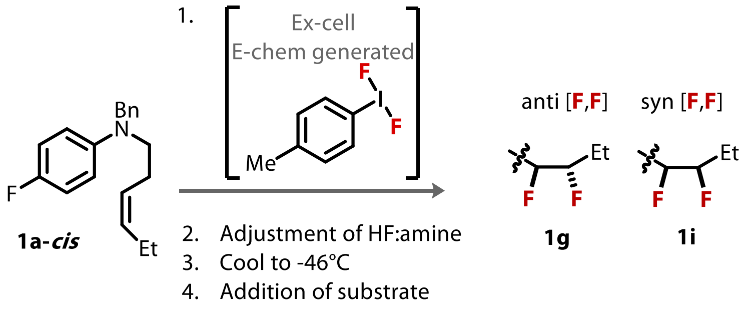

To each compartment of a PTFE divided cell equipped with a Nafion™ membrane and stirrer bars, CH_2Cl_2 (1.5 mL) and 5.6HF:amine stock solution (4.5 mL) were added. To the anodic compartment, *p*-iodotoluene (1 eq., 1.2 mmol, 262 mg) was then added. Each compartment was then capped and wrapped in parafilm. A platinum electrode was inserted into each compartment, and the reaction was subjected to electrolysis (13.4 mA, 2.2 F, 5 hrs). The electrodes were then removed, and 1 mL of this solution was added to a 100 mL HDPE vial, equipped with a stirrer bar. Additional py•9HF was added to adjust the HF:amine (additional py•9HF added: 0.1 mL (6HF:amine), 0.3 mL (6.6 HF:amine), 0.5 mL (7 HF:amine), 0.7 mL (7.3 HF:amine), 0.9 mL (7.5 HF:amine)). This vial was capped with a Suba-seal and a venting needle was placed through the Suba-seal and was cooled to -46 ºC. Alkene **1a-*cis*** (1.0 м, 0.2 mmol, 58 mg) in CH_2Cl_2 was added then added. After stirring overnight, the reaction mixture was quenched with 100 mL of cold (0 ºC) saturated aqueous $NaHCO_3$ solution. This stirred for 1 hour until the aqueous layer measured pH 7. The mixture was extracted into CH_2Cl_2, dried with Na_2SO_4, filtered and concentrated *in vacuo*. To the mixture was then added $CDCl_3$ (2 mL) and hexafluorobenzene (1 eq., 0.2 mmol, 23.2 µL), at which point the ^{19}F NMR was measured. The NMR yield was then measured by comparing the integration of the fluorine signal of the product (δ = -128.42 ppm) to that of the internal standard (δ = -161 ppm).

Table S15 Standard conditions: 4-iodotoluene (1 eq., 1.2 mmol) in 4.5 mL of 5.6 HF:amine and 1.5 mL CH_2Cl_2; electrolysis: 13 mA, 2.2 *F*, divided cell, Pt||Pt. After electrolysis, transfer 1 mL of anodic compartment to HDPE vessel, adjustment of HF:amine *via* addition of more py•9HF, cool to -46 °C, addition of 1a-*cis* (0.2 mmol), and then overnight stirring. Yields are determined by ^{19}F NMR using hexafluorobenzene as internal standard.

Entry	HF:amine	1g	1i	1a-*cis*
1	5.6	0	0	95
2	6	0	21	75
3	6.6	0	42	10
4	7	0	48	8
5	7.3	0	45	0
6	7.7	0	29	18

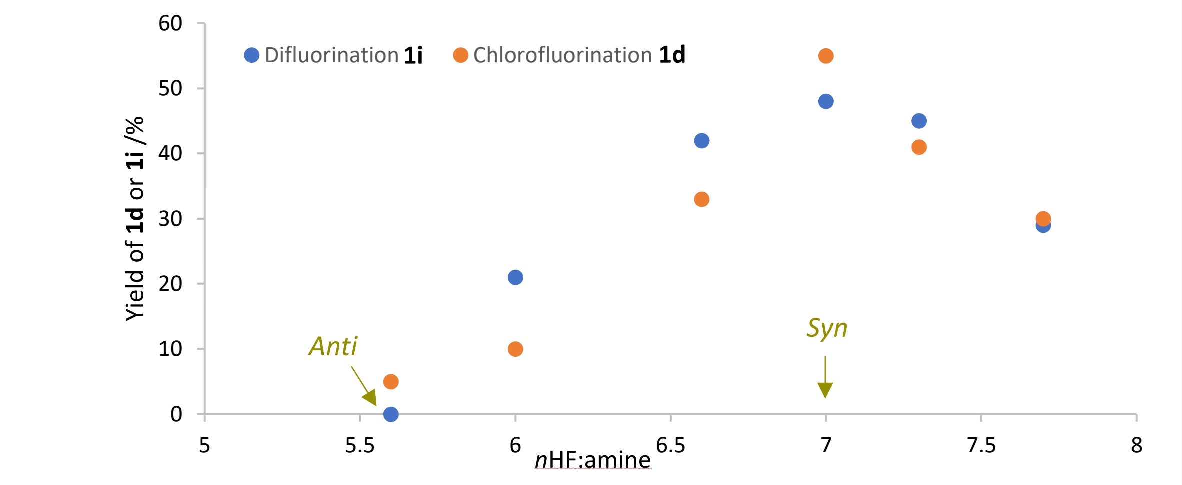

Figure S40 Comparison of NMR yields obtained from difluorination of **1a-*cis*** and chlorofluorination of **1a-*cis*** at −46 °C at HF:amine ratios 5.6, 6, 6.6, 7, 7.3 and 7.7. Yield of *syn*-difluorinated product **1i** under difluorination conditions follows a trend similar to the yield of the *syn*-chlorofluorinated product **1d** under the chlorofluorination conditions, where increasing the HF:amine ratio to 7 leads to a maximum yield of 48% for **1i** under difluorination conditions and 55% for **1d** under chlorofluorination conditions. It is argued that these data support the involvement of a common intermediate, namely an activated complex of **1a-*cis*** with ***IF2***.

Computational studies

Computational methods

DFT calculations were carried out using Gaussian 16 suite of programmes.[24] Conformational searches were performed using Spartan ’20.[25] Geometry optimisations and frequency calculations were performed using Truhlar’s M06-2X functional,[26] which has been used before in computational modelling of hypervalent iodine-mediated transformations,[27–29] Pople’s 6-31+G(d) basis set for all atoms except for I, for which LANL2DZ was used,[30] and the SMD[31] continuum model to capture solvent effects of dichloromethane. More accurate single point energies were computed using the M06-2X functional again, SMD to again capture solvent effects of CH_2Cl_2, and the larger def2-TZVP which includes f functions on I, which have been shown to be important in correctly modelling the rate-determining steps in hypervalent iodine-mediated transformations.[32] Thermodynamic data were computed at standard pressure (1 atm) and a temperature of 227.15 K as the reaction methodology utilises a temperature of −46 °C. Natural bond orbital analysis was carried out as implemented by Gaussian 16 at the M06-2X/def2-TZVP/SMD(DCM) level of theory.[33] AIM analysis was performed using MultiWFN.[34] Transition states were computed using the quadratic synchronous transit method[35] or the Berny optimisation algorithm and their nature as first order saddle points confirmed by observing a single imaginary frequency along the desired reaction coordinate. Structures were visualised using CYLview.[36]

Activation barrier for first order reaction at −46 °C

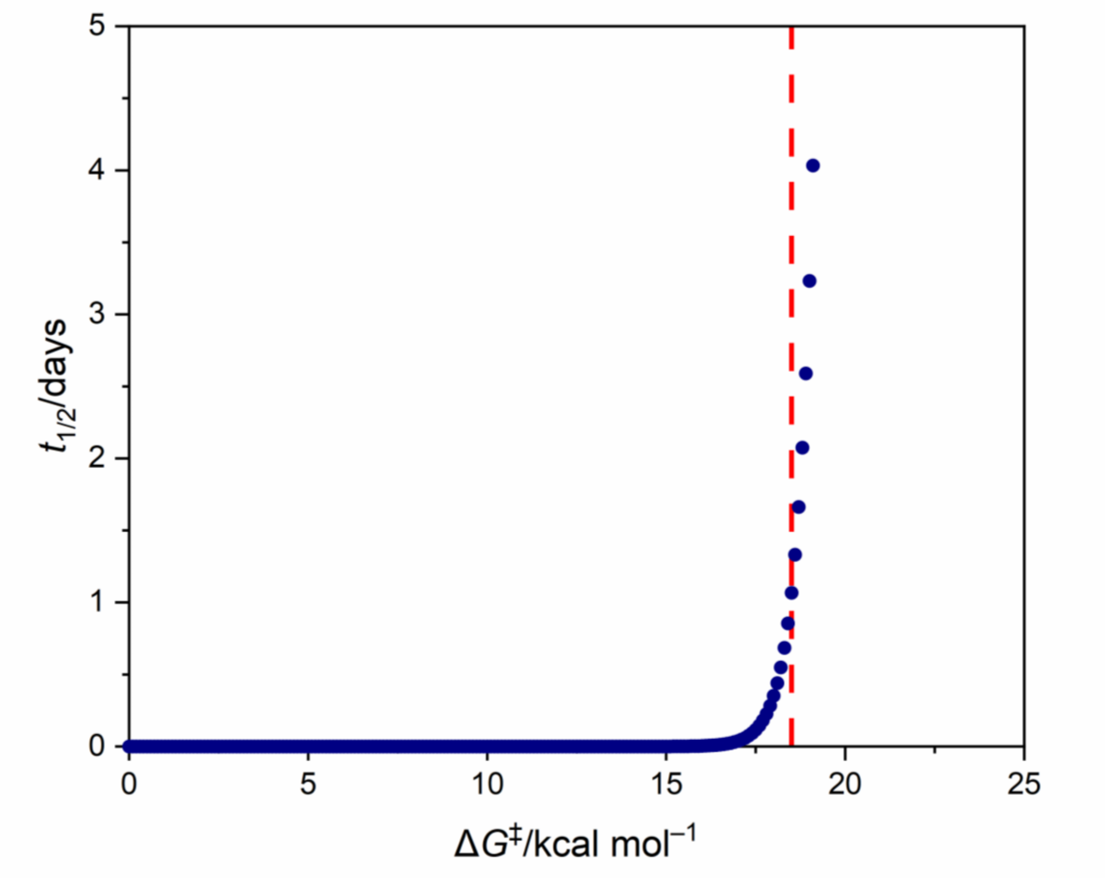

Figure S41 Plot of first-order reaction half-life in days against activation barrier in kcal/mol for a reaction at temperature −46 °C.

- Enthalpy and entropy are temperature-dependent state functions
- **~18.5 kcal/mol (red line) is free energy difference for a reaction half-life of 1 day at –46 °C.**

Iodine(III)iranium cation vs iodine(III)-π complex

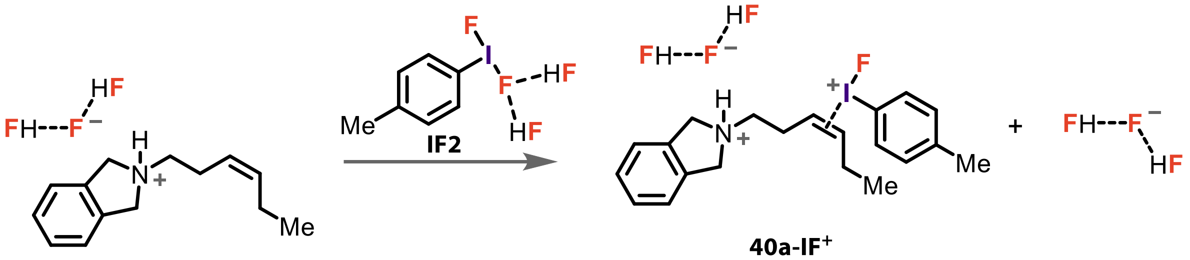

40a-onium

40a-IF⁺
$\Delta_f G$ +17.7 kcal/mol

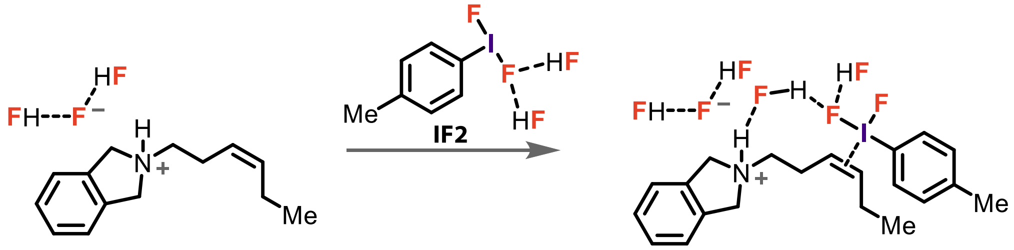

40a-onium

40a-IF2
$\Delta_f G$ +8.3 kcal/mol

Figure S42 Computed free energy changes of formation in kcal/mol for iodine(III)iranium cation formation, which is found to be less feasible than iodine(III)-π complexation which is found to be more feasible at the reaction temperature −46 °C.

We have found a lower free energy change of formation $\Delta_f G$ for iodine(III)-π complexes (which are overall neutral) **40a-IF2** than iodine(III)iranium cations **40a-IF⁺**, whose formation would be slower at −46 °C. Moreover, iodine(III)iranium cations have previously been reported as high energy intermediates and iodine(III)-π complexes were also shown to be lower energy intermediates.[29] Therefore, our subsequent calculations focus on the formation and reaction of neutral iodine(III)-π complexes.

Chloronium-forming transition states via anchimeric assistance

Chloronium-forming transition states via 1,2-chloride shift/NGP

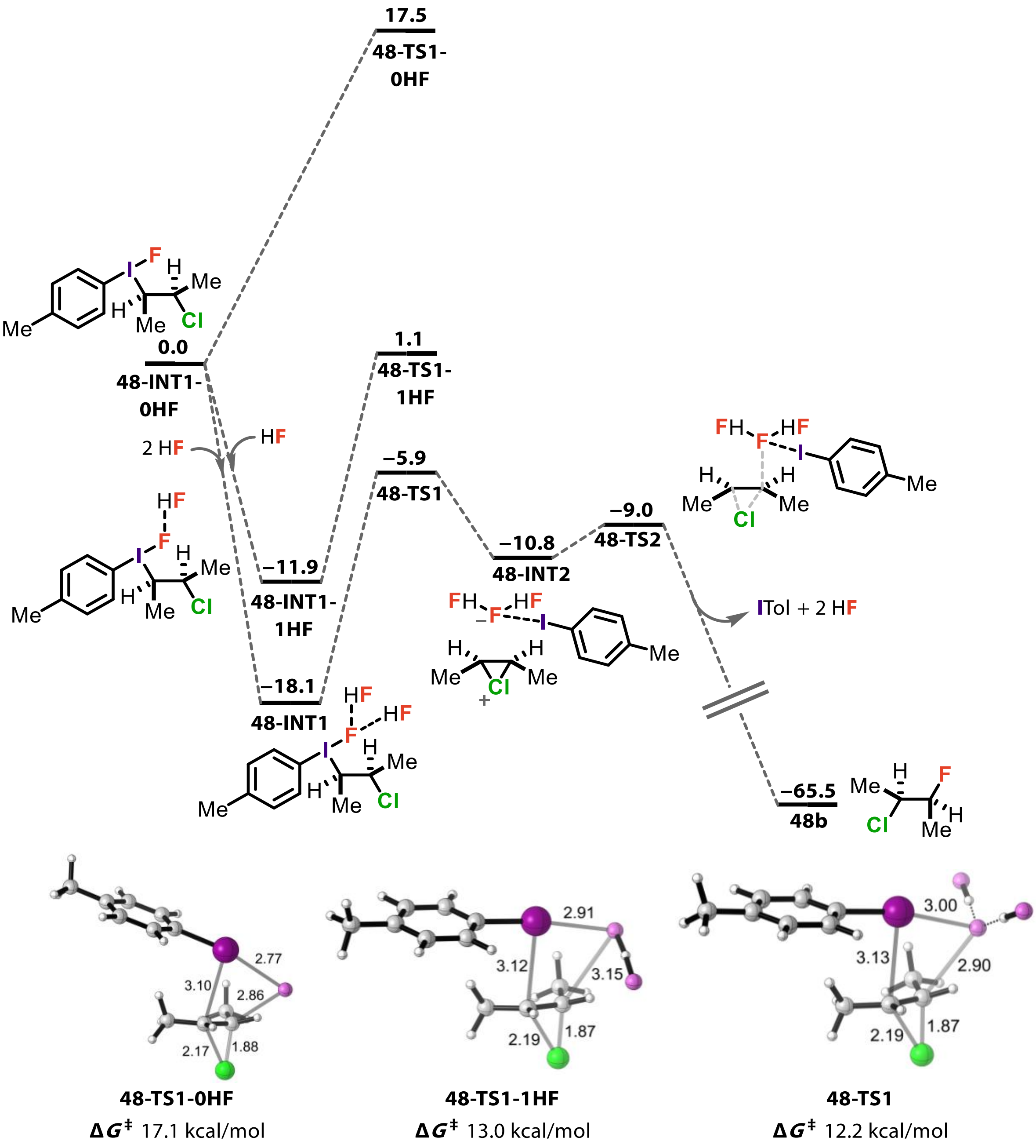

Figure S43 Potential energy surface for the formal 1,2-chloride shift mechanism proposed as the operative mechanism in the anti-chlorofluorination protocol. Free energy changes are reported in kcal/mol.

QTAIM analysis of F---I-Tol halogen bonding

The electron density of intermediate **48-INT2** and transition state **48-TS2** was calculated at the M06-2X/def2-TZVP+SMD(CH_2Cl_2) level of theory. The electron density and its topological properties at the F−I bond critical point for intermediate **48-INT2** are tabulated below.

Table S16 Topological properties of the electron density at the F−I bond critical point in intermediate **48-INT2**.

Species	ρ_b(F−I)/e Å^{-3}	$\nabla^2\rho_b$(F−I)/e Å^{-3}	$H(r_b)$[F−I]/
**48-INT2**	0.014	0.057	0.002

The electron density and its topological properties at the F−I bond critical point for TS **48-TS2** is tabulated below.

Table S17 Topological properties of the electron density at the F−I bond critical point in transition state **48-TS2**.

Species	ρ_b(F−I)/e Å^{-3}	$\nabla^2\rho_b$(F−I)/e Å^{-3}	$H(r_b)$[F−I]/
**48-TS2**	0.013	0.053	0.002

The low values of the BCP electron density (ρ_b< 0.100 e Å^{-3}), the positive values of the BCP energy density $H(r_b)$ and the positive values of the BCP Laplacian of the electron density $\nabla^2\rho_b$ indicate that the F−I interaction is electrostatic in nature, but the high directionality of the bond additionally indicates that there could be a halogen bonding interaction in the intermediate and transition state.

Ligand coupling transition states

Terminal alkene model

C–Cl ligand coupling in terminal alkene model

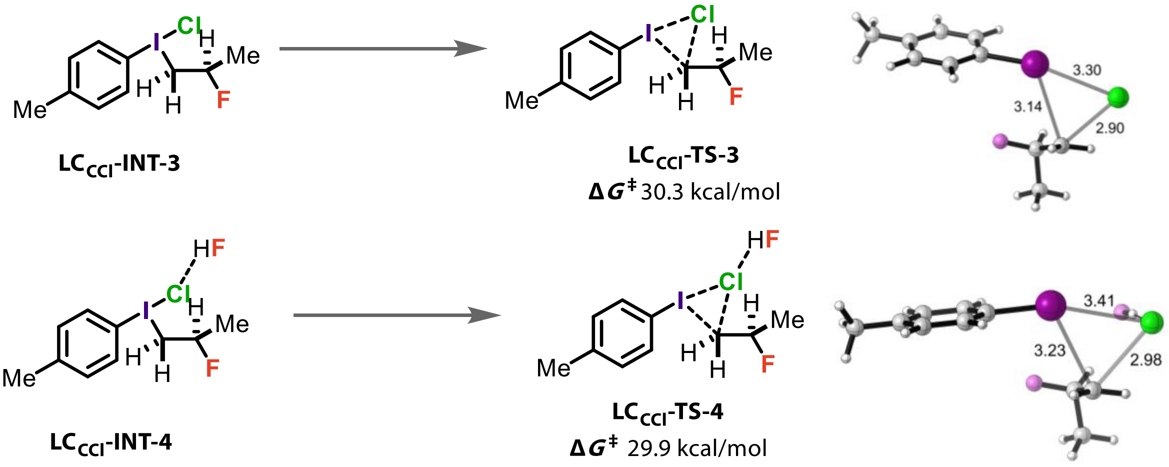

C–F ligand coupling in terminal alkene model

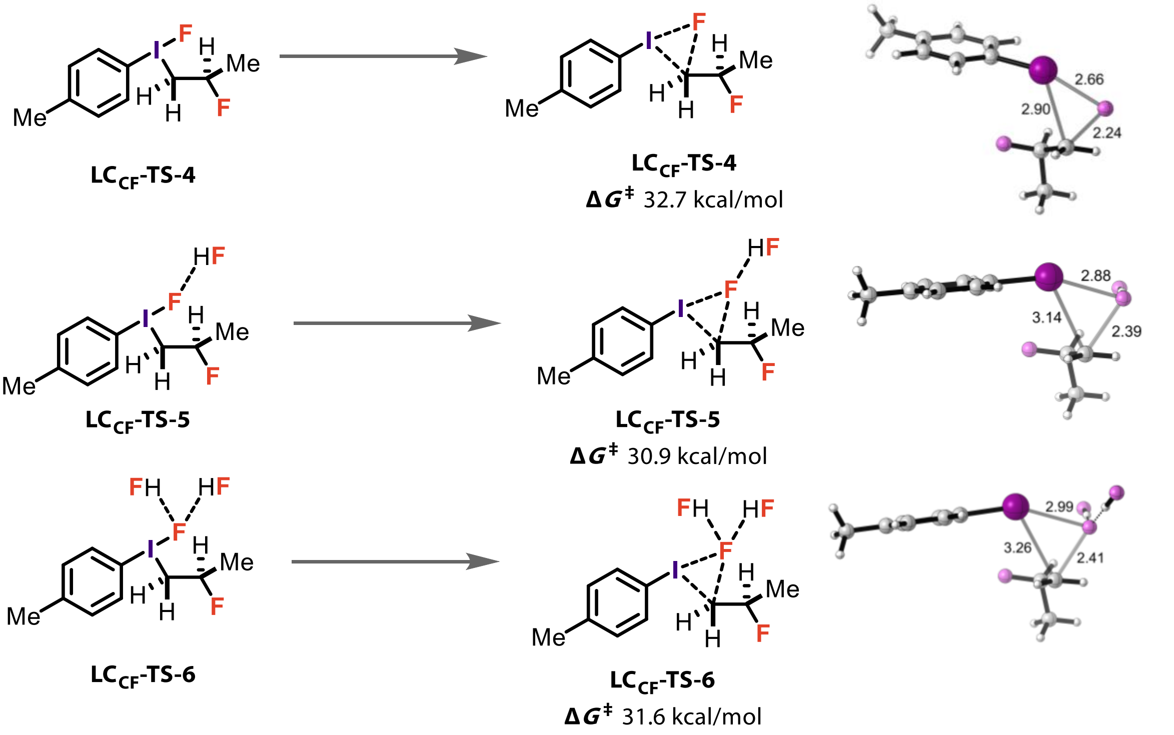

Figure S44 Ligand coupling transition states for terminal alkene model system where halide ligand and alkyl ligand are in a cis configuration at the I(III) centre, which lead to computed activation barriers too high at reaction temperature −46 °C and suggest against their operativity.

Internal alkene model

C–Cl ligand coupling in internal alkene model

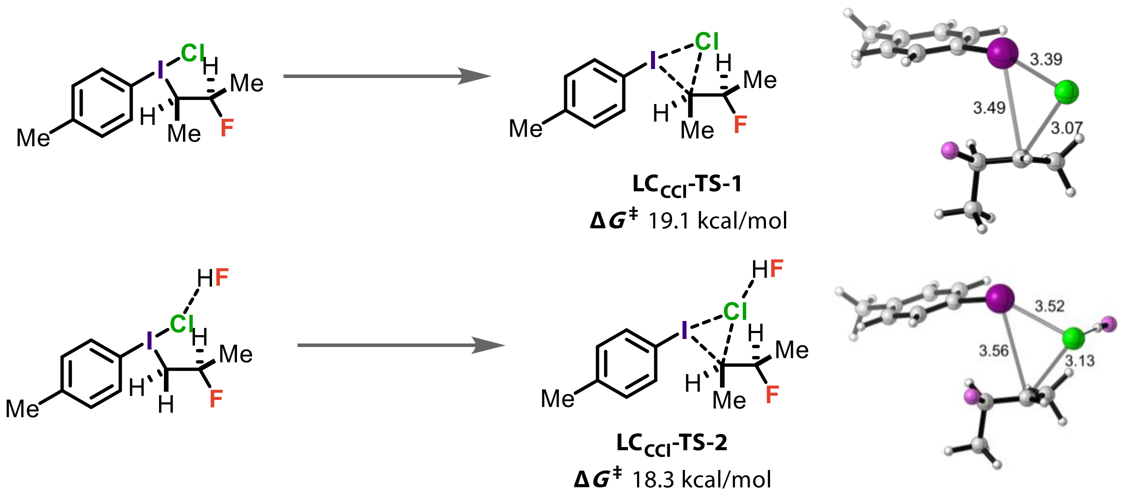

C–F ligand coupling in internal alkene model

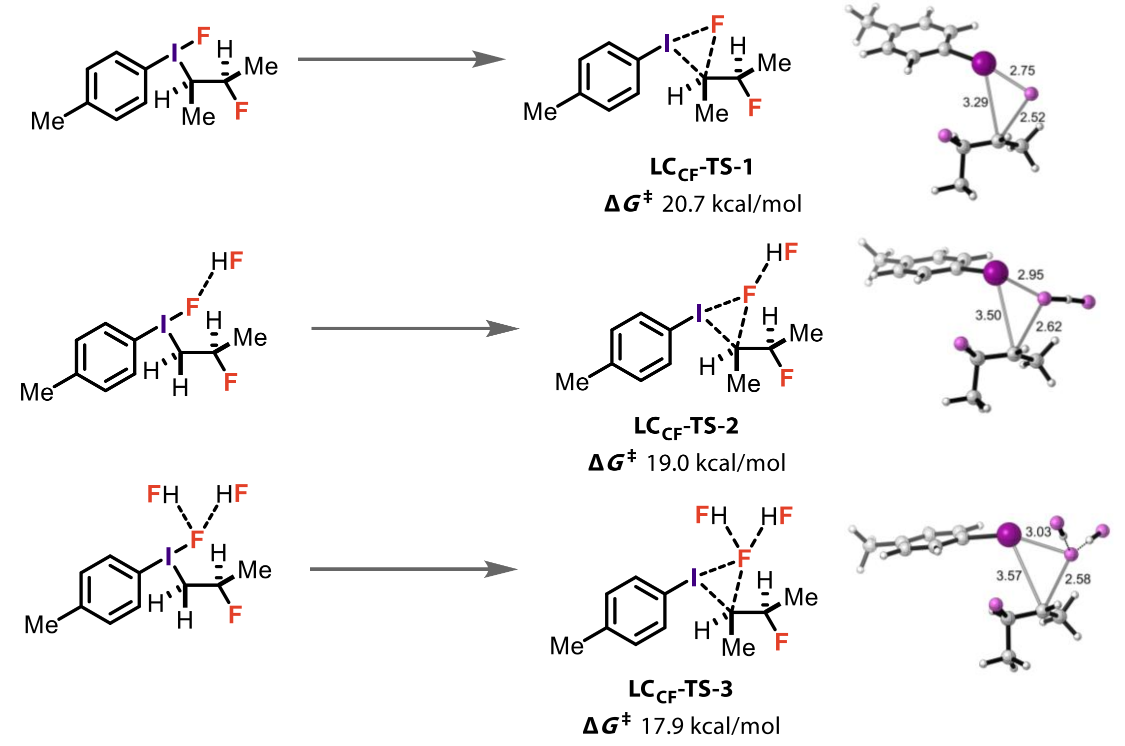

Figure S45 Ligand coupling transition states for internal alkene model system where halide ligand and alkyl ligand are in a cis configuration at the I(III) centre, which lead to computed activation barriers too high at reaction temperature −46 °C and suggest against their operativity.

Direct chloronium-forming transition states by formal "Cl$^+$" transfer from chloride-bearing iodanes

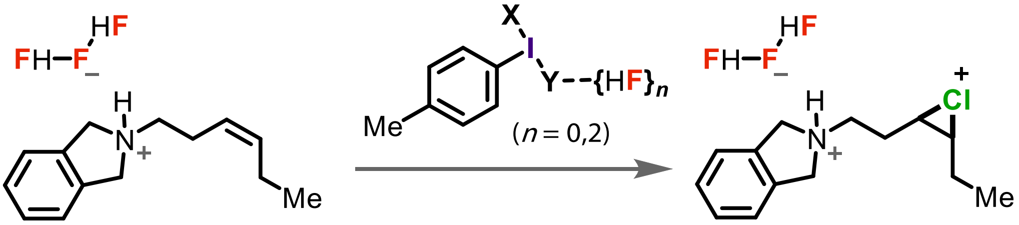

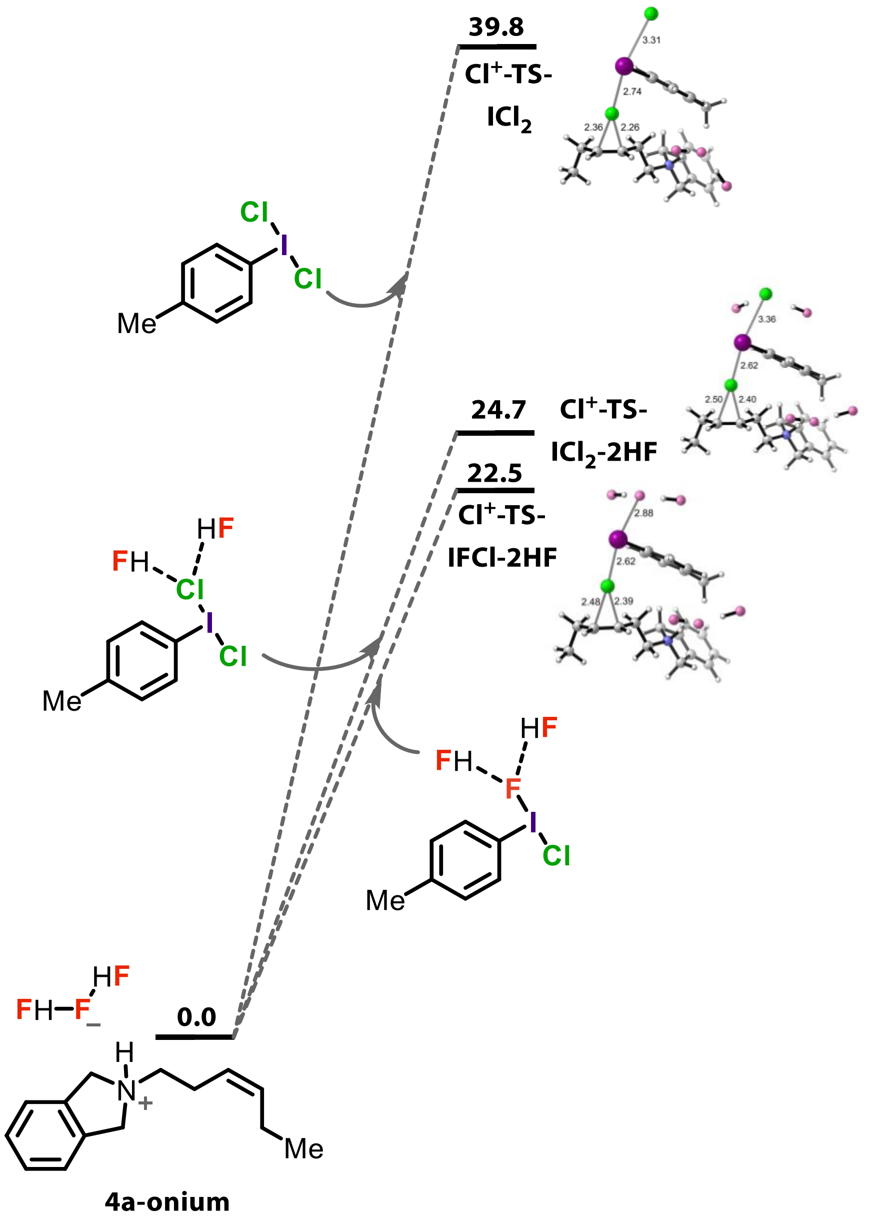

Figure S46 Direction chloronium-forming transition states by formal "Cl+" transfer by overlap of the alkene π MO with the iodane σ* MO, which lead to activation barriers too high for reaction temperature −46 °C and suggest against their operativity.

Syn 1,2-halo-λ^3-iodanation VS halide nucleophilic addition

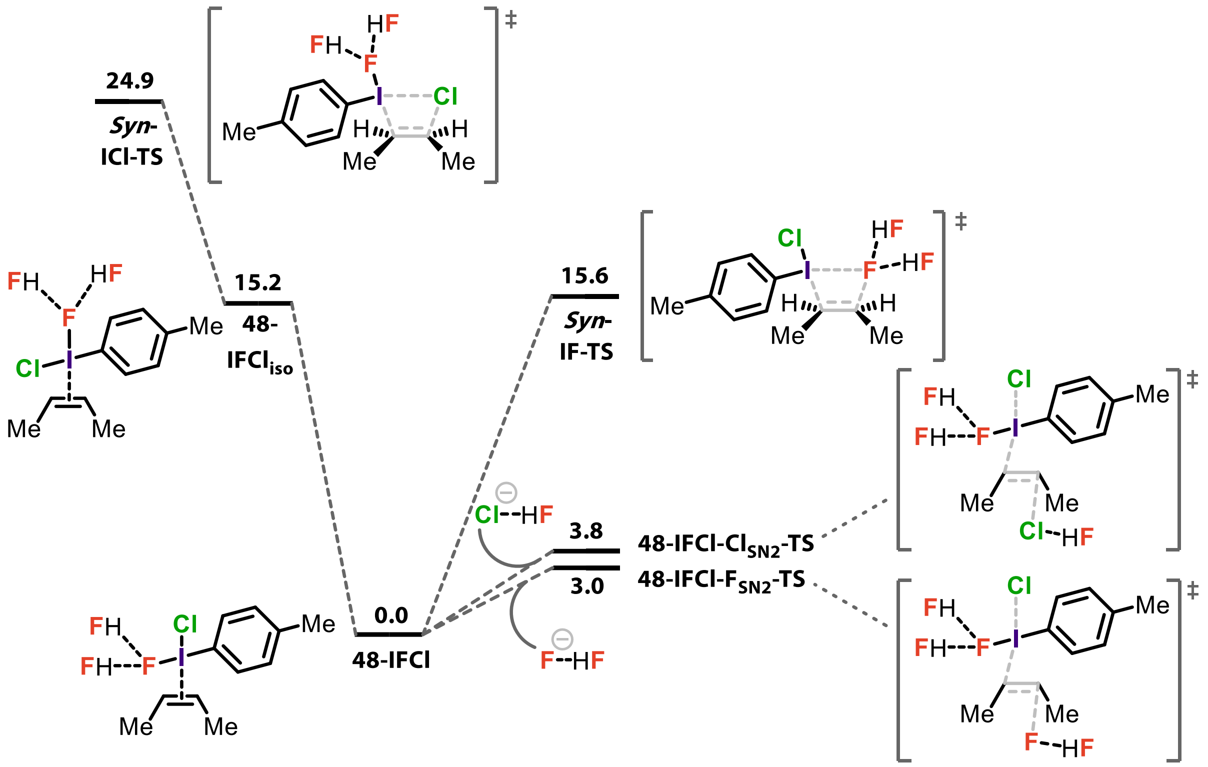

Figure S47 *Syn*-1,2-halo-λ^3-iodanation transition states starting from complex **48-IFCl**, which would have to isomerise to complex **48-IFCl$_{iso}$** which would undergo more facile *syn*-addition. *Syn*-1,2-halo-λ^3-iodanation is outcompeted by halide nucleophilic addition (chloride-1HF shown above).

Whilst these barriers are theoretically accessible at room temperature, these processes are considered to be less feasible at −46 °C. The nucleophilic interception of intermediate **48-IFCl** by halide (chloride or fluoride) in the reaction system is expected to proceed with low activation barriers. Transition state structures **48-IFCl-Cl$_{SN2}$-TS** and **48-IFCl-F$_{SN2}$-TS** were located for the nucleophilic addition of chloride-1HF and fluoride-1HF to **48-IFCl**, respectively. However, an intrinsic reaction coordinate (IRC) calculation failed to connect a reactant structure (**48-IFCl** + Cl$^-$-1HF reaction complex) and a product structure (**48-INT1-1HF**), instead the forward and reverse descents located the structure of **48-INT1**. Despite this, with 48-IFCl-Cl$_{SN2}$-TS as an approximate transition state, we do not expect that the activation barrier will largely change for either transition state if further geometry optimisation attempts were made. Therefore, the *syn*-1,2-halo-λ^3-iodanation is considered a higher energy reaction pathway that is outcompeted by nucleophilic addition pathways involving either chloride first and fluoride second (*anti*-chlorofluorination regime), or fluoride first and chloride second (*syn*-chlorofluorination regime). A previous computational study of the geminal difluorination of β-substituted styrenes found that *syn*-1,2-fluoro-λ^3-iodanation was a higher energy pathway compared to the *anti*-1,2-fluoro-λ^3-iodanation.[27] We note that the halides modelled with one HF molecule are a simplification of the reaction system and do not here account for which nucleophile attacks first or second.

Charge and orbital features of iodanes: IF2, IFCl and ICl2

IF2: Computed structure and LUMO coefficients

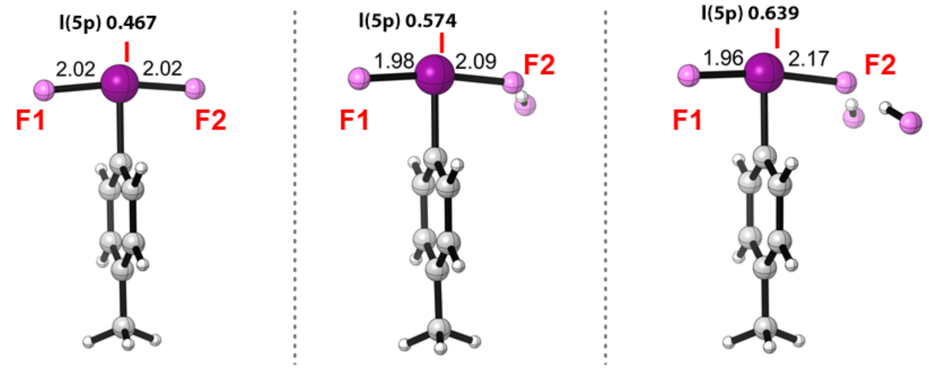

IF2-0HF IF2-1HF IF2

IFCl: Computed structure and LUMO coefficients

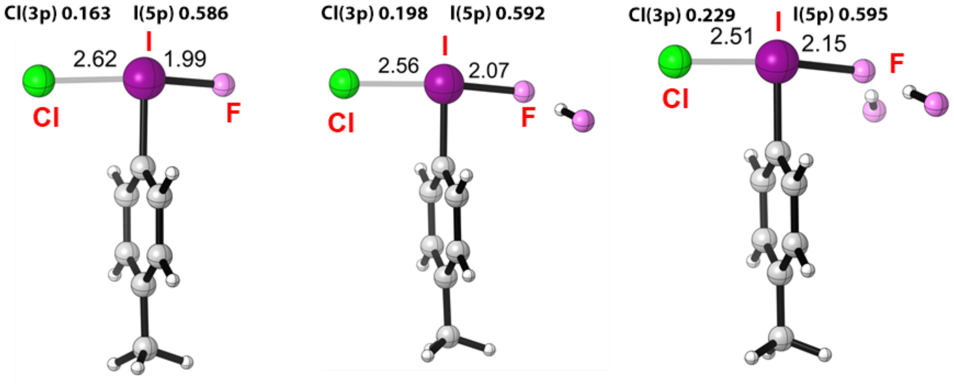

IFCl-0HF IFCl-1HF IFCl

ICl2: Computed structure and LUMO coefficients

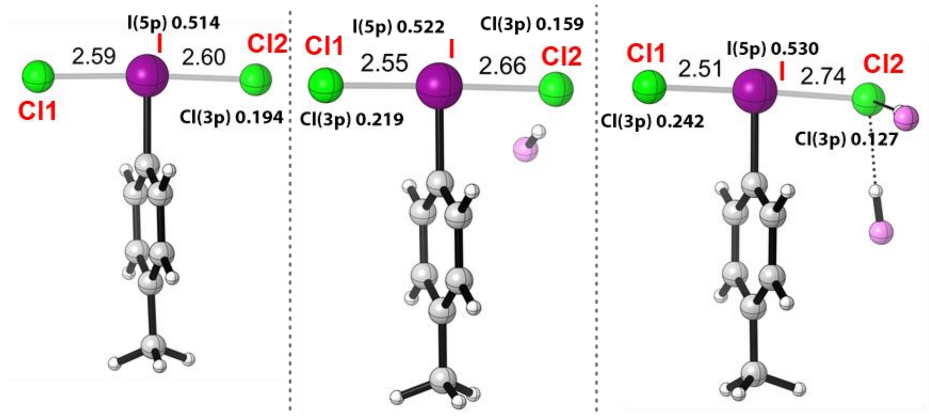

ICl2-0HF ICl2-1HF ICl2

Figure S48 LUMO coefficients on I(III) centres and ligands of *p*-Tol-IF_2, *p*-Tol-IFCl and *p*-Tol-ICl_2 with 0-2 HF molecules on one of the halide ligands.

Table S18 Computed atomic charges (AIM and NBO) of the I(III) centre and ligands in IF2, IFCl and ICl2 with 0-2 HF molecules coordinated to the same ligand.

Iodane species	AIM			NBO		
	**F1**	**I**	**F2**	**F1**	**I**	**F2**
IF2-0HF	−0.753	1.446	−0.753	−0.702	1.485	−0.702
IF2-1HF	−0.736	1.462	−0.770	−0.676	1.497	−0.718
IF2	−0.714	1.461	−0.783	−0.649	1.508	−0.742
	**Cl**	**I**	**F**	**Cl**	**I**	**F**
IFCl-0HF	−0.620	1.255	−0.730	−0.605	1.319	−0.679
IFCl-1HF	−0.555	1.222	−0.750	−0.527	1.291	−0.782
IFCl	−0.493	1.191	−0.771	−0.454	1.263	−0.783
	**Cl1**	**I**	**Cl2**	**Cl1**	**I**	**Cl2**
ICl2-0HF	−0.553	0.981	−0.557	−0.537	1.070	−0.533
ICl2-1HF	−0.502	0.993	−0.584	−0.475	1.092	−0.583
ICl2	−0.503	1.015	−0.584	−0.482	1.105	−0.582

In summary and on the basis of their ground state properties, IFCl is predicted to be the more reactive chloride ligand-bearing iodane with a more partially positive I(III) centre with a higher LUMO coefficient than that in ICl2.

π-complexation of alkene and three possible iodanes

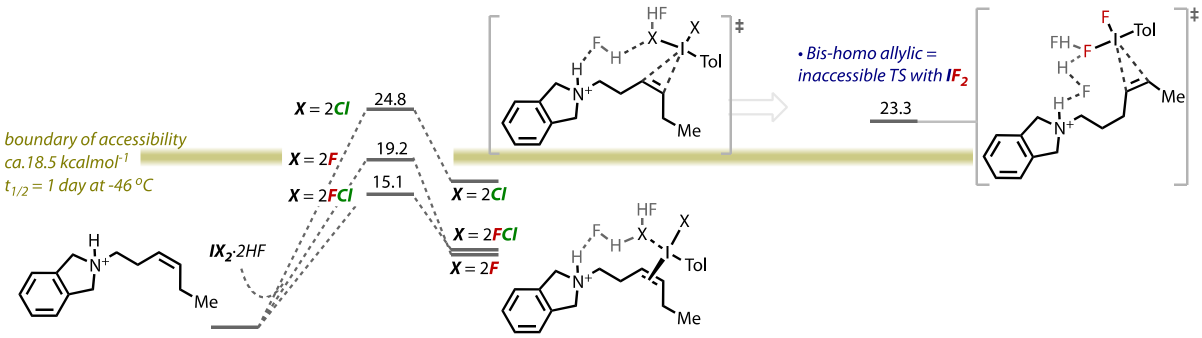

Figure S49 Iodine(III)-π complexation activation barriers with *p*-TolIF$_2$, *p*-TolIFCl and *p*-TolICl$_2$.

π-complexation transition states were computed for the computational model substrate **40a-*cis*** with the three iodanes: *p*-Tol-IF$_2$, *p*-TolICl$_2$ and *p*-TolIFCl. The lowest activation barrier was calculated for *p*-TolIFCl, which alongside the results of the iodane mixing studies strongly suggests that the mixed iodane is the more reactive iodane responsible for alkene activation at the low reaction temperature.

π-complexation of *trans*-alkene

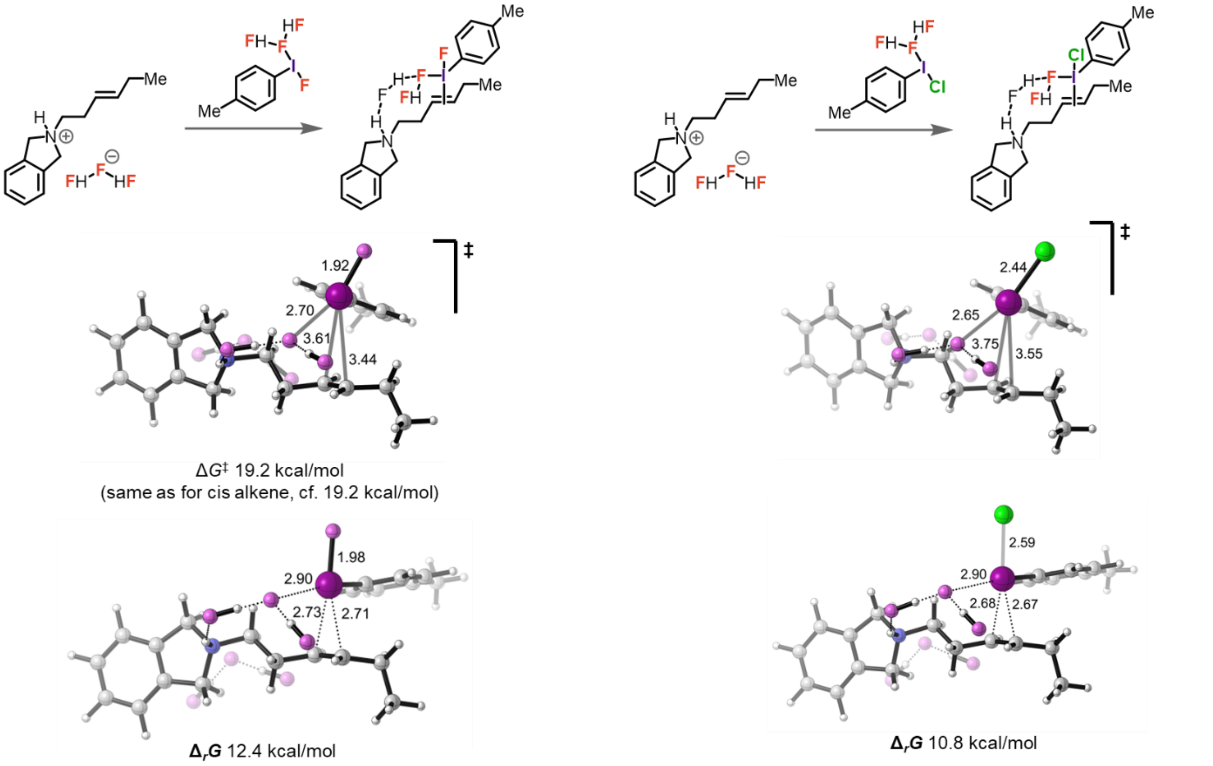

Figure S50 Activation barriers ($\Delta G^{\ddagger}$) and free energy changes of reaction ($\Delta_r G$) for the π-complexation of computational model of *trans*-alkene. Energies are reported in kcal/mol.

Ligand exchange

There are three possible iodane species, ***IF2***, ***IFCl*** and ***ICl_2*** which were modelled with different extents and sites of HF coordination. Associative ligand exchange transition states were computed for the first ligand exchange process ***IF_2 → IFCl*** and the second ligand exchange process ***IFCl → ICl_2***. The impact of HF coordination on the nucleophilicity of the incoming chloride was investigated. Coordination of two HF molecules to the chloride nucleophile slightly more than doubles the activation energy from 8.0 kcal/mol (**TS1-0HF**) to 16.2 kcal/mol (**TS1-2HF**). Coordination of HF to bare **IFCl** leads to species higher in free energy: fluoride-coordinated **IFCl-1HF** and **IFCl-2HF** are computed to be 2.7 and 6.3 kcal/mol more stable than chloride-coordinated **IFCl-1HF(Cl)** and **IFCl-2HF(Cl)**, respectively. Second ligand exchange is modelled from **IFCl-2HF**, where activation energies are much lower for ligand exchange with a second chloride than for the first ligand exchange with chloride. Second exchange with chloride is 2.4 kcal/mol (**TS2-0HF**; compare 7.6 kcal/mol to **TS1-0HF**), with one HF 9.4 kcal/mol (**TS2-1HF**; compare 12.7 kcal/mol to **TS1-1HF**) and with two HF molecules 11.1 kcal/mol (**TS2-2HF**; compare 15.8 kcal/mol to **TS1-2HF**). The reverse process from **ICl_2** is expected to be feasible with sufficient HF coordination to the chloride ligands, which is destabilising with respect to the non-coordinated iodane ground states.

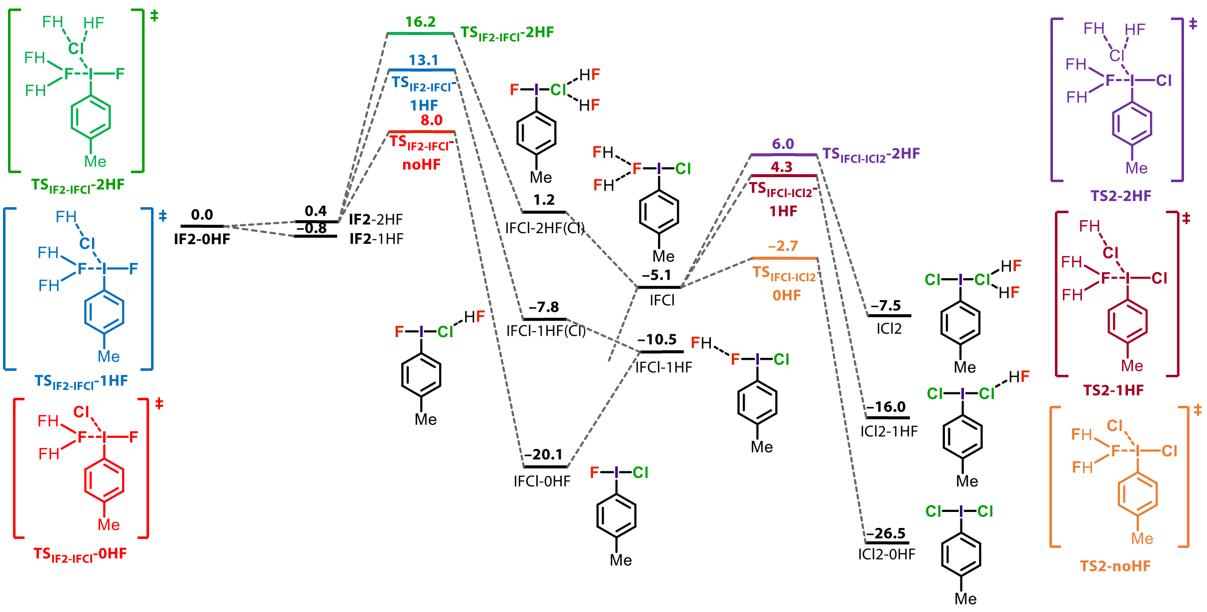

Figure S51 Free energy profile for the ligand exchange from *p*-TolIF$_2$ to *p*-TolIFCl and *p*-TolIFCl to *p*-TolICl$_2$. Free energy changes reported in kcal/mol.

Ligand metathesis mechanism between iodanes

Because of the results of the iodane mixing experiments which showed that p-TolICl2 in the presence of equimolar p-TolIF2 restored the yield of 1b (see Section: Use of pre-formed p-TolIF2 and p-TolICl2 in the reaction), a theoretical study of a ligand metathesis mechanism was carried out to assess the feasibility of a reaction that could produce p-TolIFCl from p-TolIF2 and p-TolICl2. A theoretically feasible process was identified to involve p-TolIF2 (IF2) and p-TolICl2, which come together in a transition state TSmet that corresponds to exchange of fluoride and chloride ligands to form two equivalents of IFCl with a computed barrier of 15.6 kcal/mol.

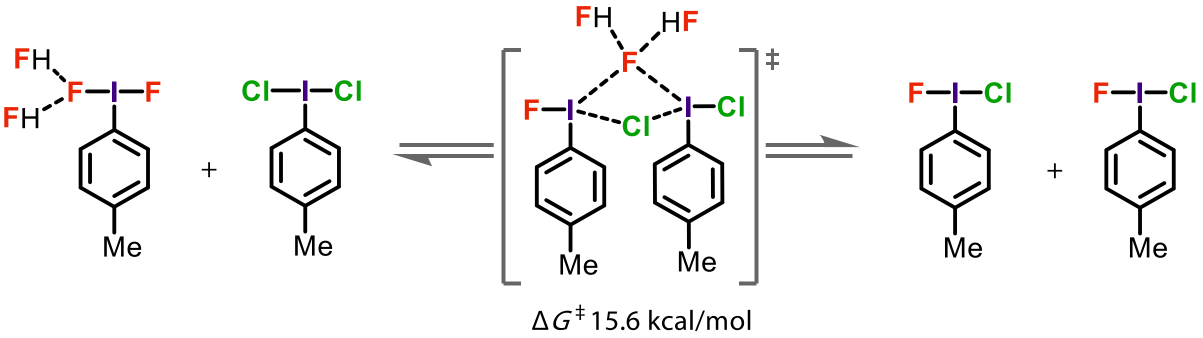

Scheme S5 Metathetical ligand exchange between *p*-TolIF$_2$-2HF and *p*-TolICl$_2$ via an accessible transition state of 15.6 kcal/mol at −46 °C.

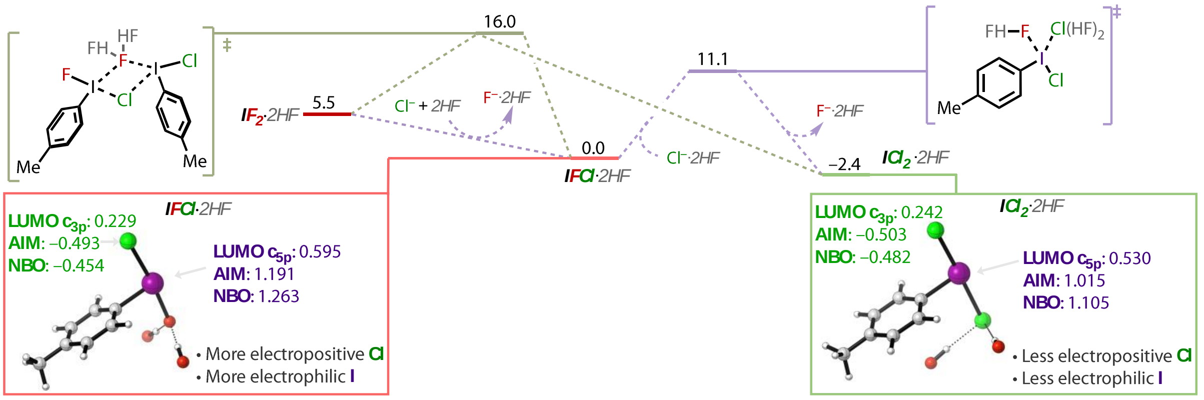

Figure S52 Condensed free energy profile for ligand exchange from *IF2* to *IFCl* to *ICl2* with Brønsted activation by two HF molecules, which includes metathetical ligand exchange.

Calculations of fluoride and chloride nucleophilicities

Structures of the isolated $X(HF)_z^-$ anions (X = F or Cl; z = 0 – 6) were optimised in the ground state using the M06-2X functional[26] and the Dunning aug-cc-pVTZ basis set.[37] Calculations were performed in SPARTAN’20 (v1.0.0).[25] Local ionisation potential maps were then plotted using SPARTAN’20 with an isovalue of 0.002 e/au^3, and the local ionisation potential associated with the anion within the cluster was determined. The most positive value for the ionisation potential was used for when the anion featured a range of ionisation potential values. Nucleophilicity of the anion within the cluster was then determined by taking the negative value of the local ionisation potential associated with the anion. This method was adopted for determining the nucleophilicity of the anion due to halides being electron donors when acting as nucleophiles.

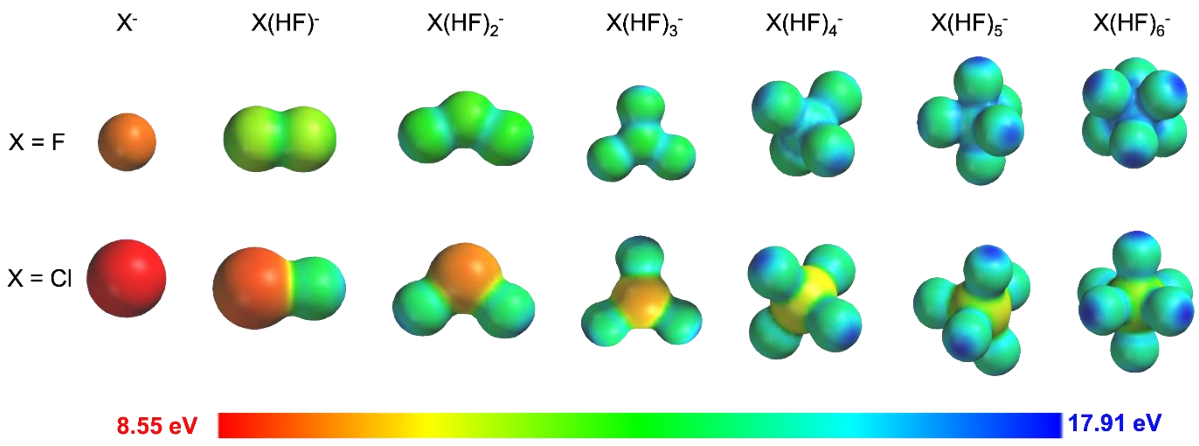

Figure S53 The local ionisation potential maps found for the optimised structures of different $X(HF)_z^-$ anionic clusters (X = F or Cl; z = 0-6).

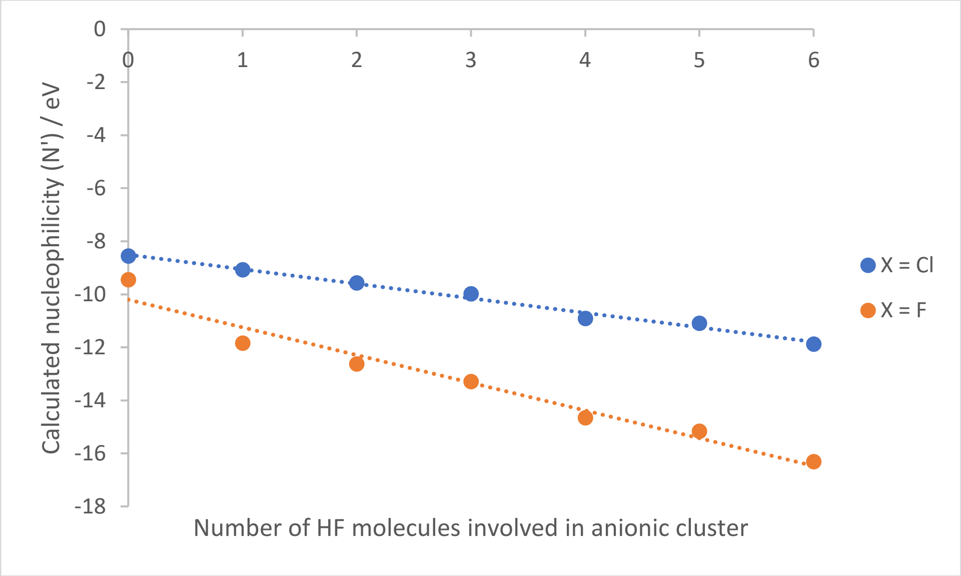

Figure S54 The calculated nucleophilicity values found for the optimised structures of different $X(HF)_z^-$ anionic clusters (X=F or Cl; z = 0-6).

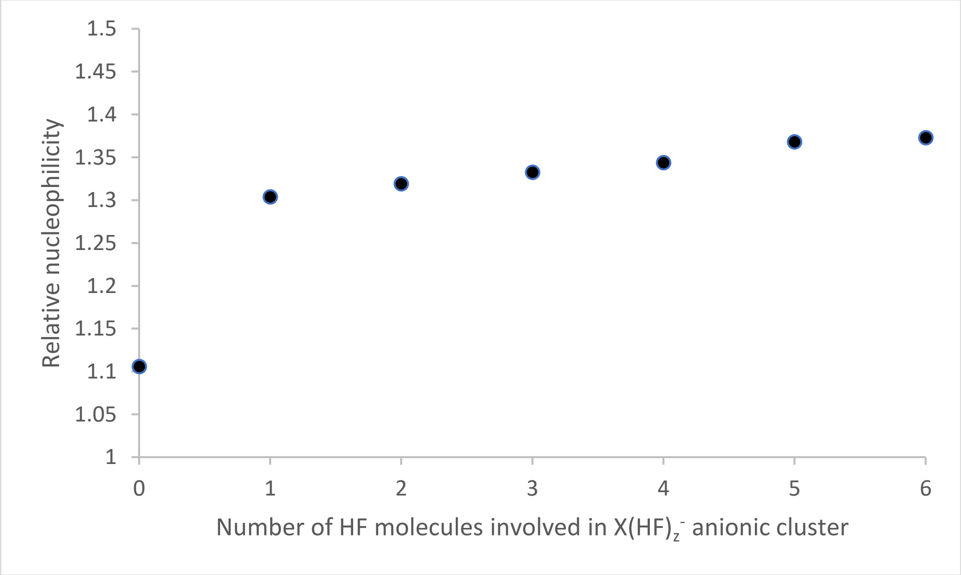

Figure S55 The relative nucleophilicity of $Cl(HF)_z^-$ anionic clusters compared to $F(HF)_z^-$ anionic clusters. The relative nucleophilicities demonstrate that for any sized cluster involving HF molecules, the chloride anionic cluster is more nucleophilic than the equivalent fluoride anionic cluster.

References

1. Foroozandeh, M., Morris, G. A. & Nilsson, M. PSYCHE Pure Shift NMR Spectroscopy. *Chem. Eur. J.* **24**, 13988–14000 (2018).
2. Liu, R., Wei, Z., Wang, J., Liu, Y. & Xue, H. Highly selective hydrosilylation of equilibrating allylic azides. *Chem. Commun.* **56**, 5038–5041 (2020).
3. Banik, S. M., Medley, J. W. & Jacobsen, E. N. Catalytic, Diastereoselective 1,2-Difluorination of Alkenes. *J. Am. Chem. Soc.* **138**, 5000–5003 (2016).
4. Doobary, S., Poole, D. L. & Lennox, A. J. J. Intramolecular Alkene Fluoroarylation of Phenolic Ethers Enabled by Electrochemically Generated Iodane. *J. Org. Chem.* **86**, 16095–16103 (2021).
5. Cresswell, A. J. *et al.* Diastereodivergent Hydroxyfluorination of Cyclic and Acyclic Allylic Amines: Synthesis of 4-Deoxy-4-fluorophytosphingosines. (2012) doi:10.1021/jo301056r.
6. Yan, L. *et al.* Carbohydrate/DBU Cocatalyzed Alkene Diboration: Mechanistic Insight Provides Enhanced Catalytic Efficiency and Substrate Scope. *J. Am. Chem. Soc.* **140**, 3663–3673 (2018).
7. Rodríguez-Fernández, M., Yan, X., Collados, J. F., White, P. B. & Harutyunyan, S. R. Lewis Acid Enabled Copper-Catalyzed Asymmetric Synthesis of Chiral β-Substituted Amides. *J. Am. Chem. Soc.* **139**, 14224–14231 (2017).
8. Liu, G. *et al.* A six-coordinated cationic ruthenium carbyne complex with liable pyridine ligands: synthesis, structure, catalytic investigation, and DFT study on initiation mechanism. *Tetrahedron* **70**, 4718–4725 (2014).
9. Le Darz, A. *et al.* Tandem superelectrophilic activation for the regioselective chlorofluorination of recalcitrant allylic amines. *Tetrahedron* **72**, 674–689 (2016).
10. SAINT, version 8.39.0. (2018).
11. SADABS. (2018).
12. Sheldrick, G. M. SHELXT – Integrated space-group and crystal-structure determination. *urn:issn:2053-2733* **71**, 3–8 (2015).
13. Sheldrick, G. M. & IUCr. A short history of SHELX. *urn:issn:0108-7673* **64**, 112–122 (2007).
14. Sheldrick, G. M. Crystal structure refinement with SHELXL. *urn:issn:2053-2296* **71**, 3–8 (2015).
15. Dolomanov, O. V. *et al.* OLEX2: a complete structure solution, refinement and analysis program. *urn:issn:0021-8898* **42**, 339–341 (2009).
16. Olah, G. A. *et al.* Synthetic Methods and Reactions. 63. Pyridinium Poly(hydrogen fluoride)(30% Pyridine-70% Hydrogen Fluoride): A Convenient Reagent for Organic Fluorination Reactions. *J. Org. Chem.* **44**, 3872–3881 (1979).
17. Crespo, L. T. C., Ribeiro, R. D. S., De Mattos, M. C. S. & Esteves, P. M. Halofluorination of alkenes using trihaloisocyanuric acids and HF-pyridine. *Synthesis* **2010**, 2379–2382 (2010).
18. Glendening, E. D., Reed, A. E., Carpenter, J. E. & Weinhold, F. Deconstructing the Catalytic, Vicinal Difluorination of Alkenes: HF-Free Synthesis and Structural Study of p-TolIF2. (2001).
19. Gao, K. & Yoshikai, N. Cobalt-catalyzed ortho alkylation of aromatic imines with primary and secondary alkyl halides. *J. Am. Chem. Soc.* **135**, 9279–9282 (2013).

20. Li, Z., Hammond, G. B. & Xu, B. Quantification of hydrogen fluoride-based reagents using a bifunctional NMR internal standard. *J. Fluor. Chem.* **184**, 72–74 (2016).

21. Yokoyama, Y. *et al.* Fluoride Ion-Selective Electrode for Organic Solutions. *Anal. Chem.* **93**, 15058–15062 (2021).

22. Hoops, S. *et al.* COPASI - A COmplex PAthway SImulator. *Bioinformatics* **22**, 3067–3074 (2006).

23. Wang, J., Zhao, Y., Zhao, W., Wang, P. & Li, J. Total synthesis of N-butyl-1-deoxynojirimycin. *J. Carbohydr. Chem.* **35**, 445–454 (2016).

24. Frisch, M. J. *et al.* Gaussian 16 Revision C.01. (2016).

25. Spartan ’20 Version 1.0.0. (2021).

26. Zhao, Y. & Truhlar, D. G. The M06 suite of density functionals for main group thermochemistry, thermochemical kinetics, noncovalent interactions, excited states, and transition elements: Two new functionals and systematic testing of four M06 functionals and 12 other functionals (T. *Theor. Chem. Acc.* **119**, 525 (2008).

27. Zhou, B., Haj, M. K., Jacobsen, E. N., Houk, K. N. & Xue, X. S. Mechanism and Origins of Chemo- and Stereoselectivities of Aryl Iodide-Catalyzed Asymmetric Difluorinations of β-Substituted Styrenes. *J. Am. Chem. Soc.* **140**, 15206–15218 (2018).

28. Zheng, H., Sang, Y., Houk, K. N., Xue, X. S. & Cheng, J. P. Mechanism and Origins of Enantioselectivities in Spirobiindane-Based Hypervalent Iodine(III)-Induced Asymmetric Dearomatizing Spirolactonizations. *J. Am. Chem. Soc.* **141**, 16046–16056 (2019).

29. Shu, S., Li, Y., Jiang, J., Ke, Z. & Liu, Y. Mechanism of Hypervalent Iodine Promoted Fluorocyclization of Unsaturated Alcohols: Metathesis via Double Acids Activation. *J. Org. Chem.* **84**, 458–462 (2019).

30. Hay, P. J. & Wadt, W. R. Ab initio effective core potentials for molecular calculations. Potentials for K to Au including the outermost core orbitals. *J. Chem. Phys.* **82**, 299 (1998).

31. Marenich, A. V., Cramer, C. J. & Truhlar, D. G. Universal Solvation Model Based on Solute Electron Density and on a Continuum Model of the Solvent Defined by the Bulk Dielectric Constant and Atomic Surface Tensions. *J. Phys. Chem. B* **113**, 6378–6396 (2009).

32. Sun, T. Y. *et al.* Revisiting the effect of f-functions in predicting the right reaction mechanism for hypervalent iodine reagents. *J. Comput. Chem.* **42**, 470–474 (2021).

33. Glendenning, E. D., Reed, A. E., Carpenter, J. E. & Weinhold, F. NBO Version 3.1. (2001).

34. Tian, L. Multiwfn Version 3.8. (2023).

35. Peng, C. & Schlegel, H. B. Combining Synchronous Transit and Quasi-Newton Methods to Find Transition States. *Isr. J. Chem.* **33**, 449–454 (1993).

36. Legault, C. Y. CYLview 1.0b. (2009).

37. Kendall, R. A., Dunning, T. H. & Harrison, R. J. Electron affinities of the first-row atoms revisited. Systematic basis sets and wave functions. *J. Chem. Phys.* **96**, 6796–6806 (1992).

NMR spectra of optimisation substrates and products

(Z)-N-benzyl-4-fluoro-N-(hex-3-en-1-yl)aniline, 1a-cis

^{1}H NMR (400 MHz, $CDCl_3$):

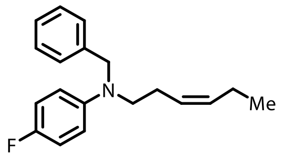

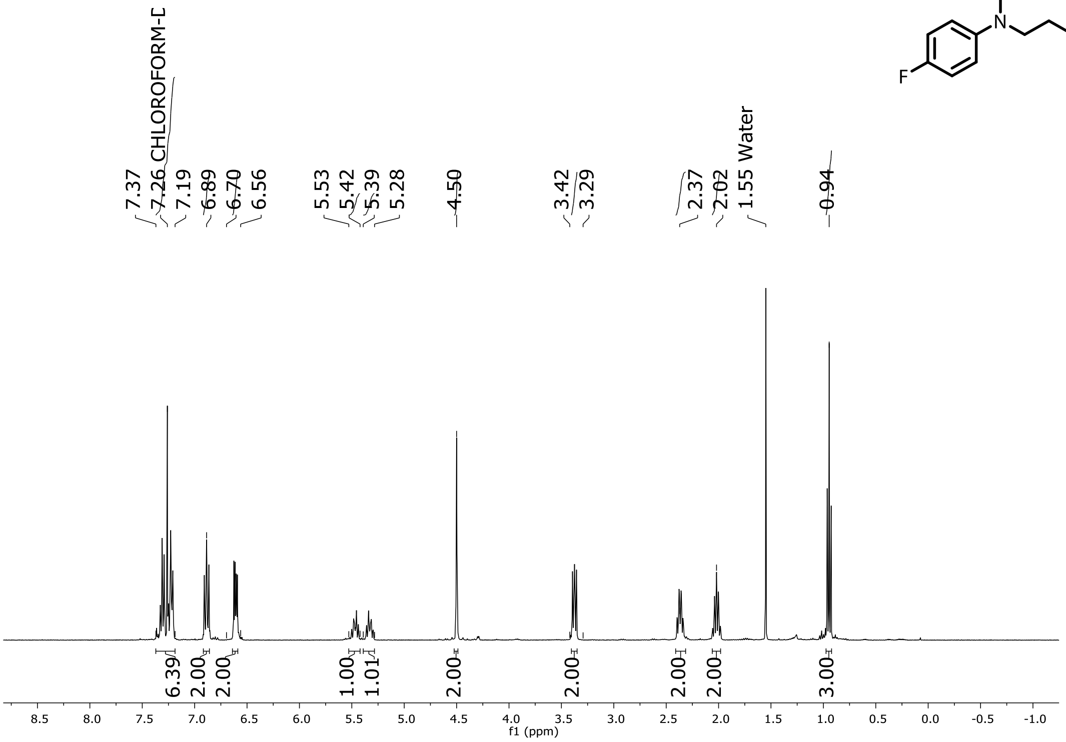

^{19}F NMR (376 MHz, $CDCl_3$):

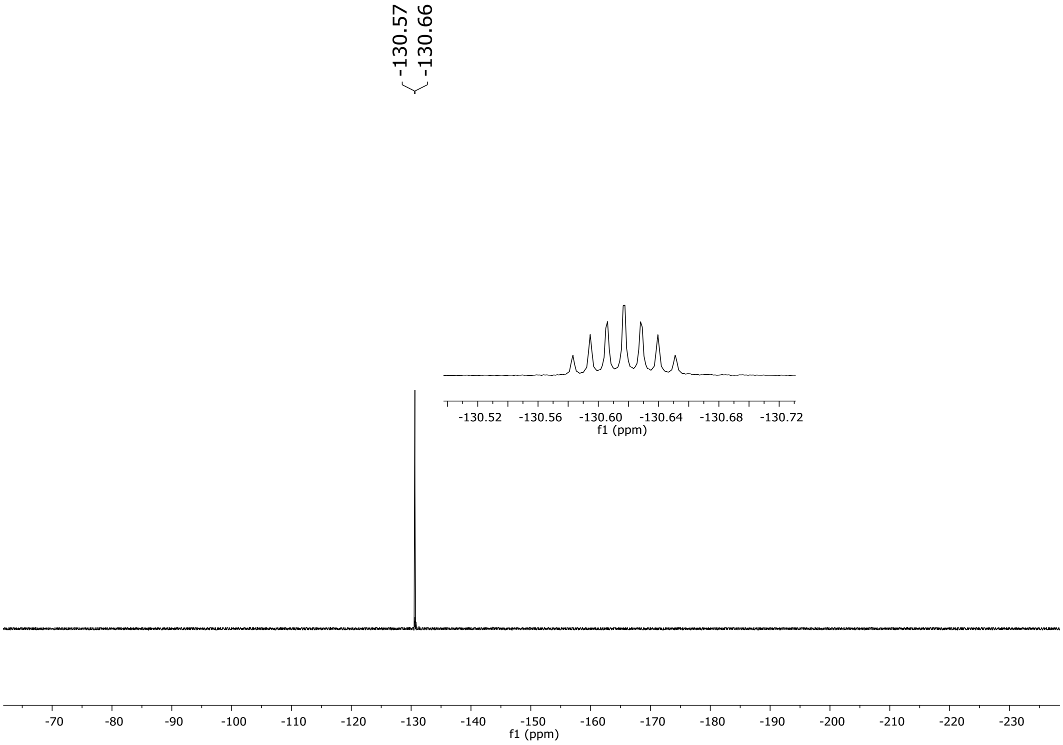

$^{13}C\ \{^{1}H\}$ NMR (100 MHz, $CDCl_3$):

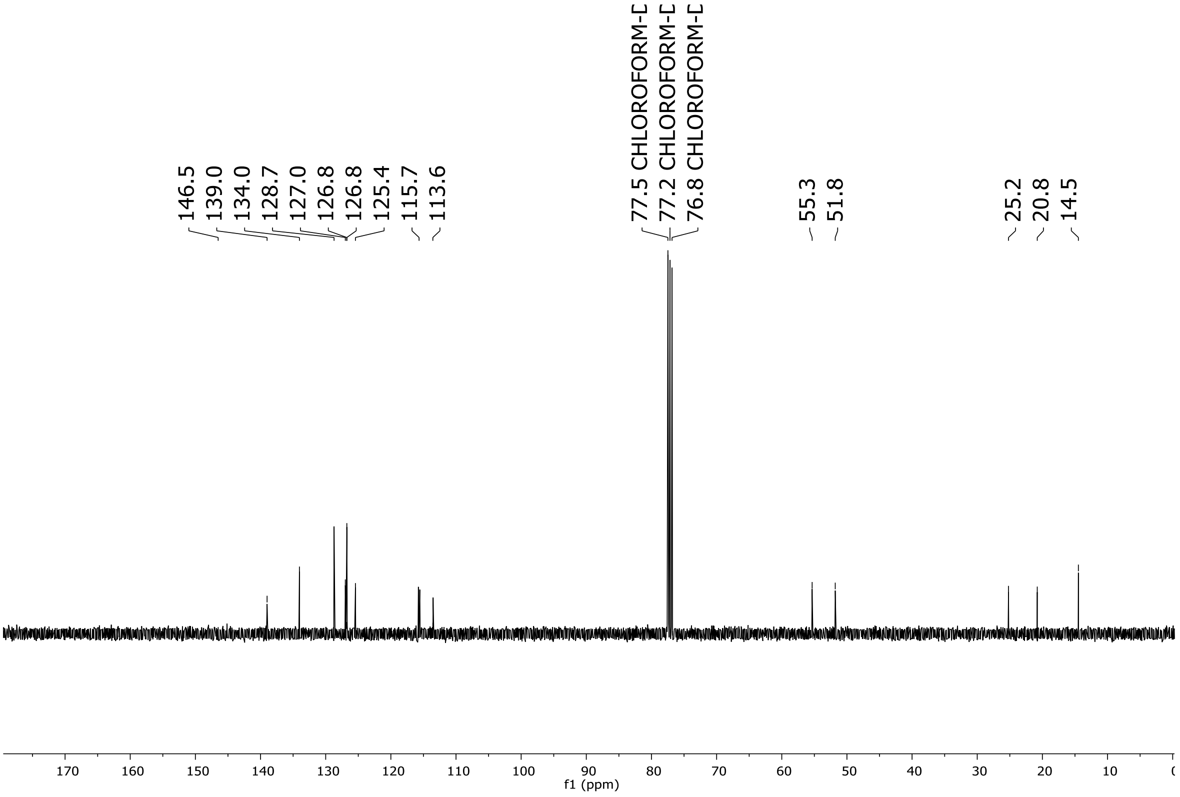

(E)-N-benzyl-4-fluoro-N-(hex-3-en-1-yl)aniline, 1a-trans

^{1}H NMR (400 MHz, $CDCl_3$):

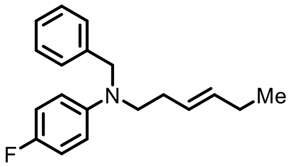

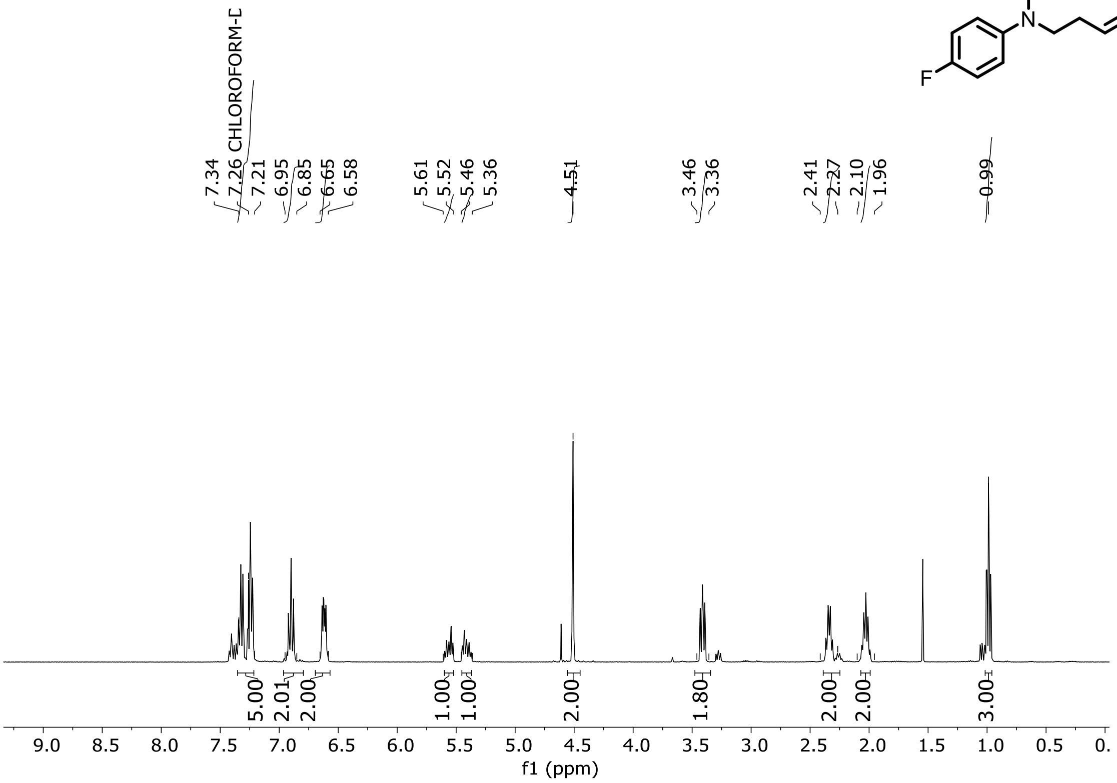

^{19}F NMR (376 MHz, $CDCl_3$):

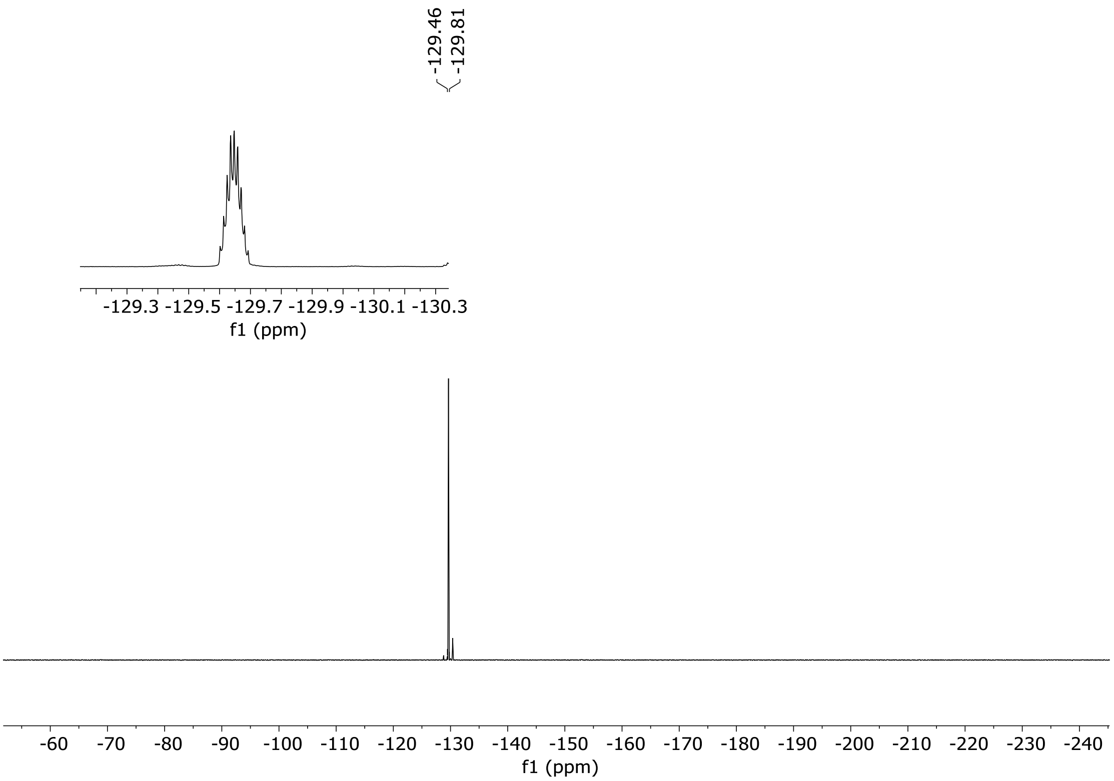

^{13}C {^{1}H} NMR (100 MHz, CDCl3):

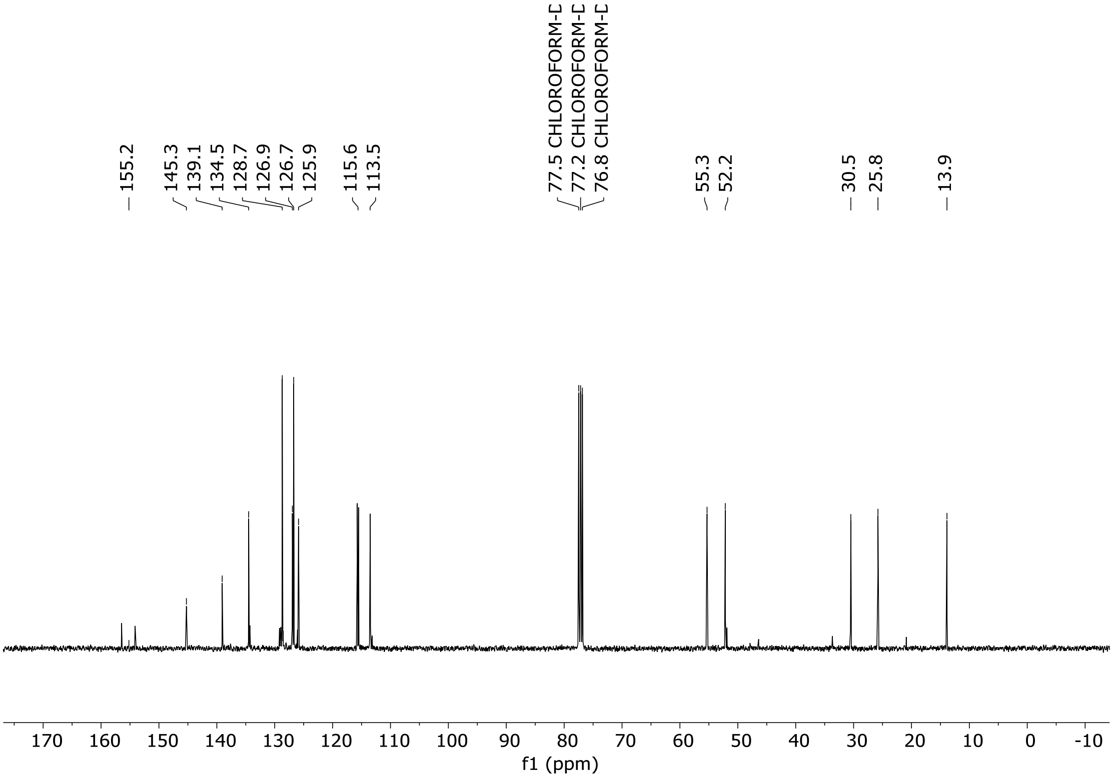

N-benzyl-N-((3S,4S)-3-chloro-4-fluorohexyl)-4-fluoroaniline, 1b

^{1}H NMR (400 MHz, $CDCl_3$):

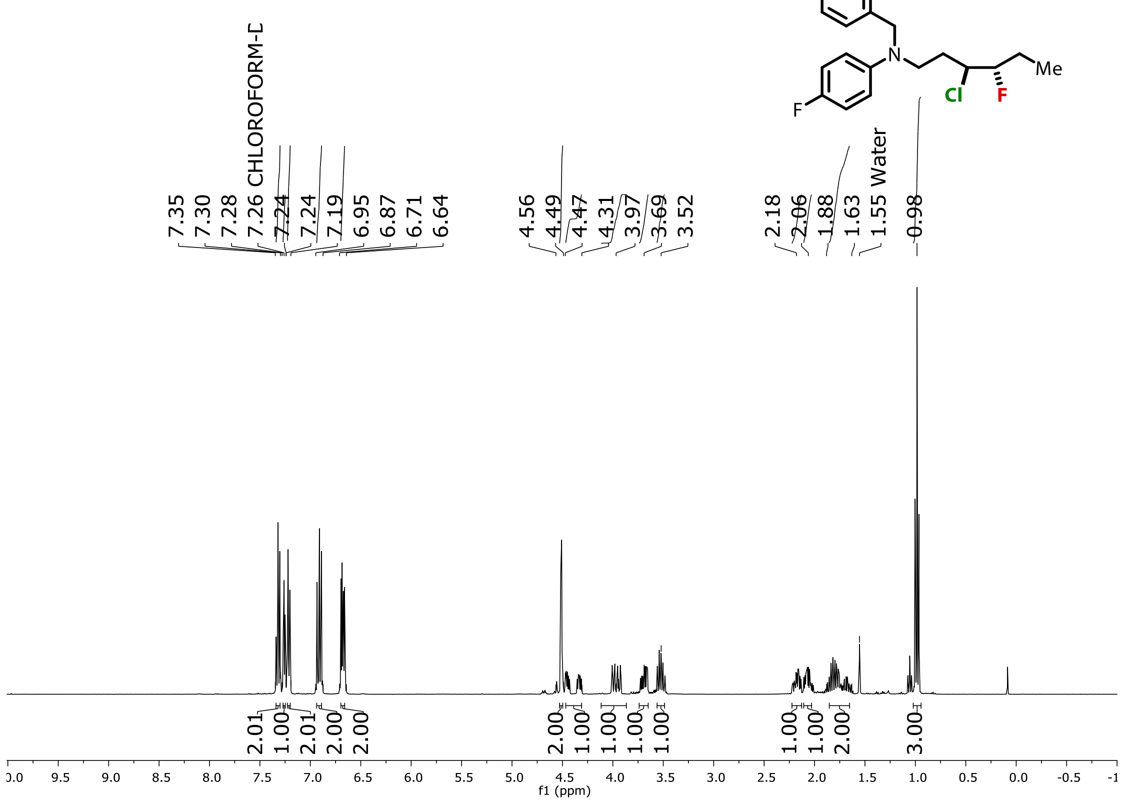

^{19}F NMR (376 MHz, $CDCl_3$):

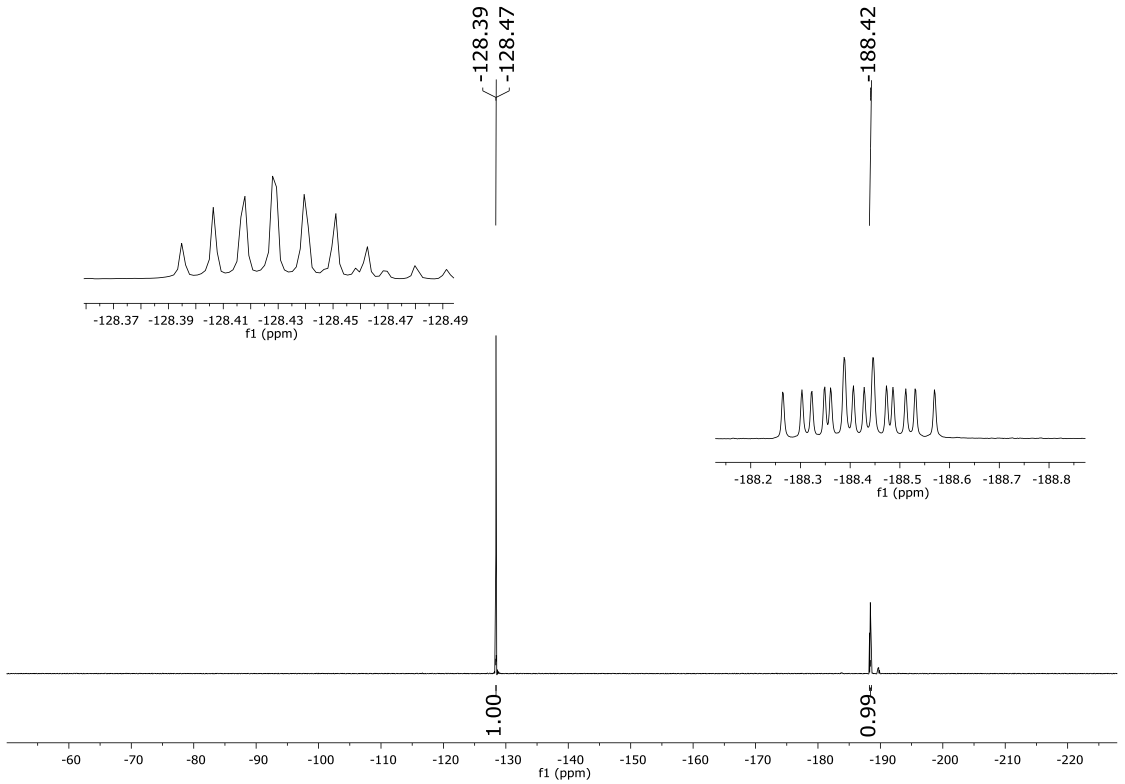

Back to top

$^{13}C\ \{^1H\}$ NMR (100 MHz, $CDCl_3$):

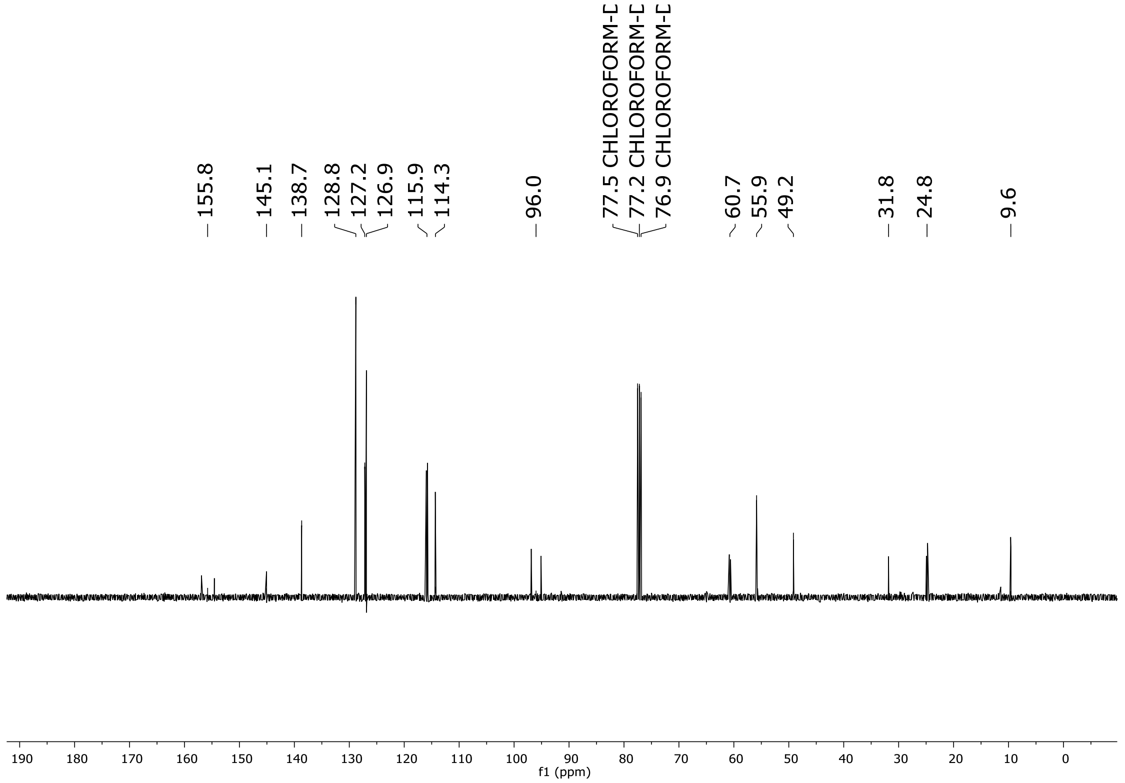

N-benzyl-N-((3S,4R)-3-chloro-4-fluorohexyl)-4-fluoroaniline, 1d

^{1}H NMR (400 MHz, $CDCl_3$):

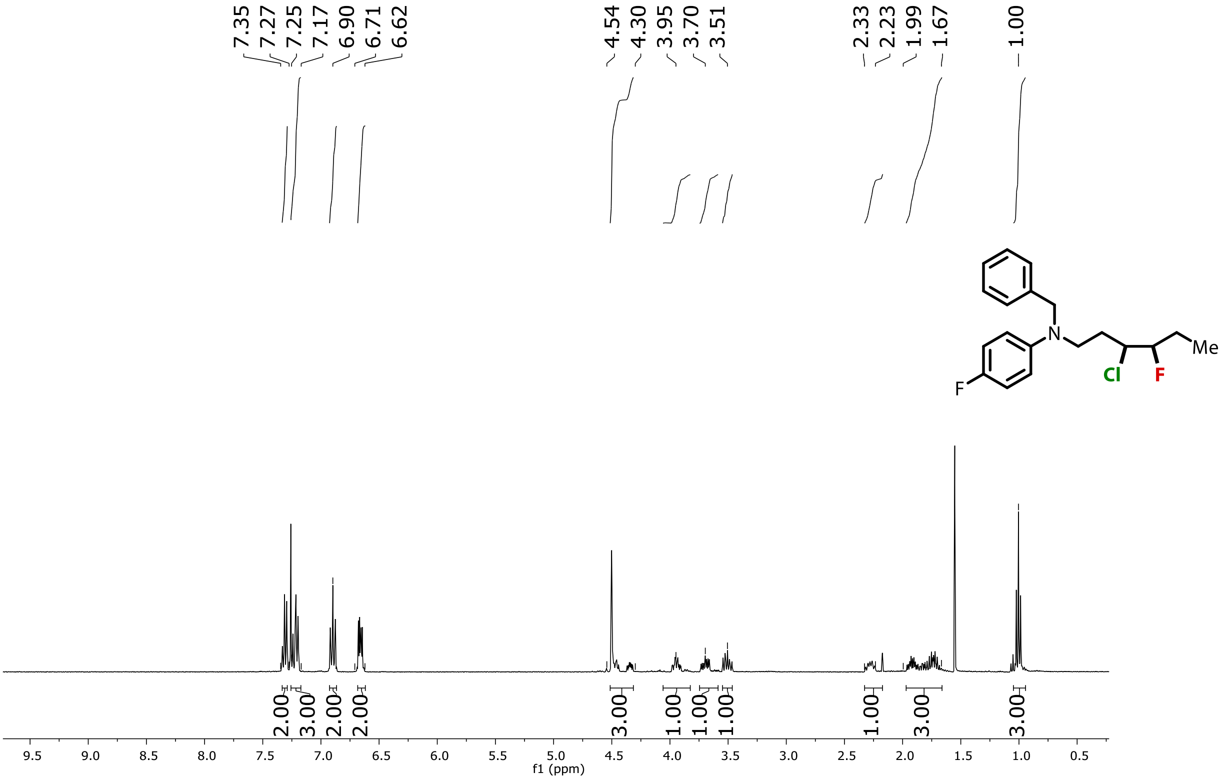

^{19}F NMR (376 MHz, $CDCl_3$):

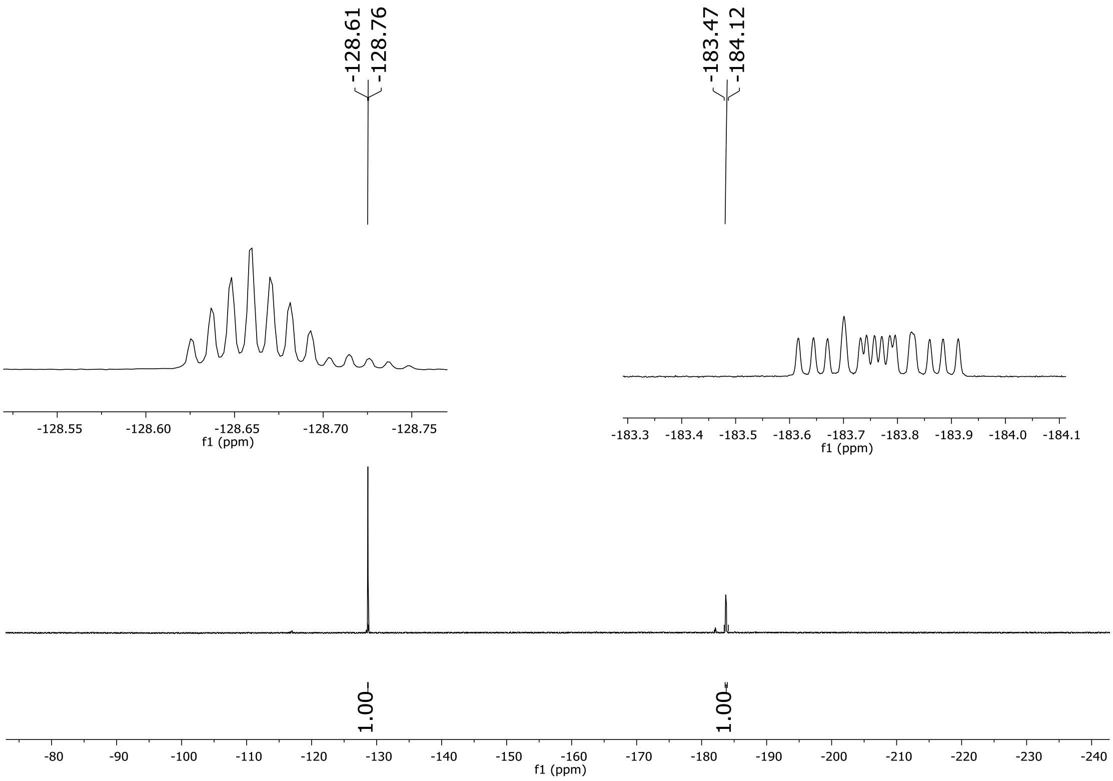

Back to top

^{13}C {^{1}H} NMR (400 MHz, $CDCl_3$):

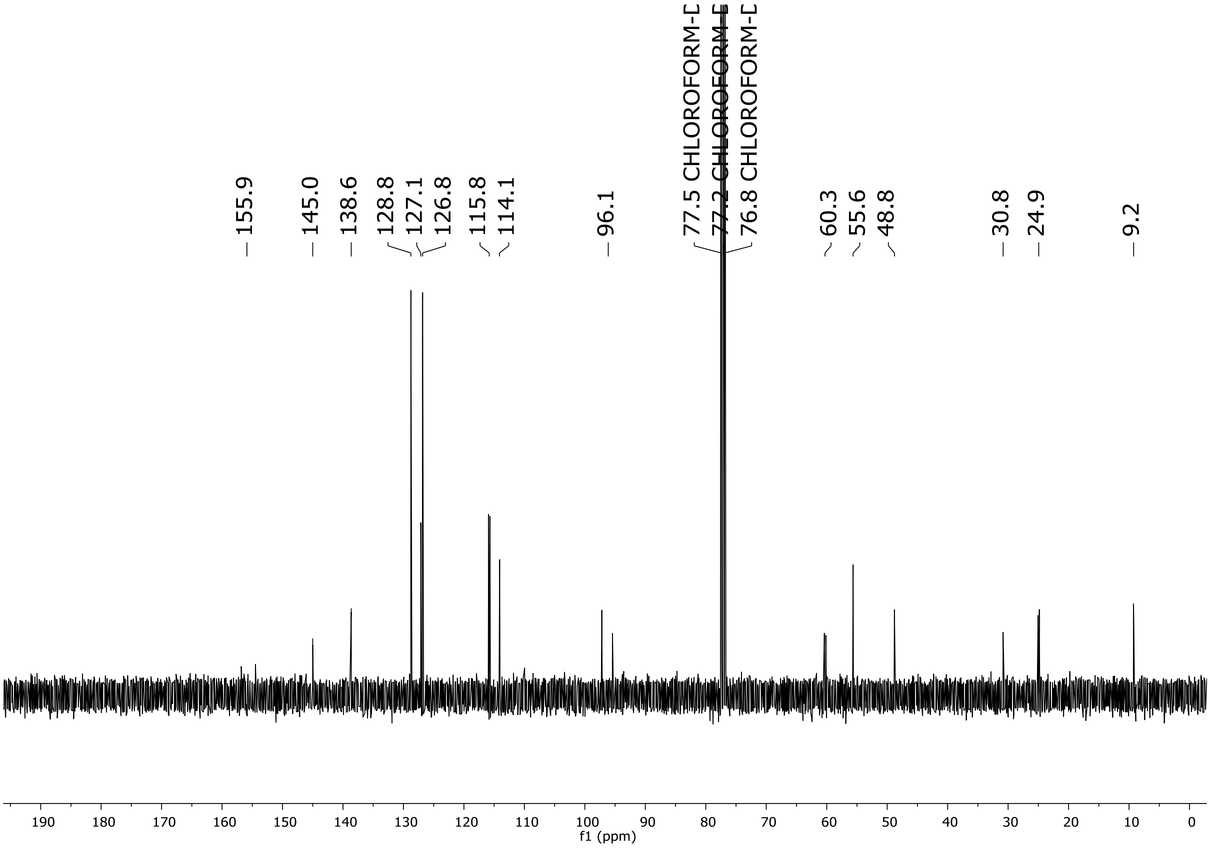

N-benzyl-N-((3S,4S)-3,4-dichlorohexyl)-4-fluoroaniline, 1f

^{1}H NMR (500 MHz, $CDCl_3$):

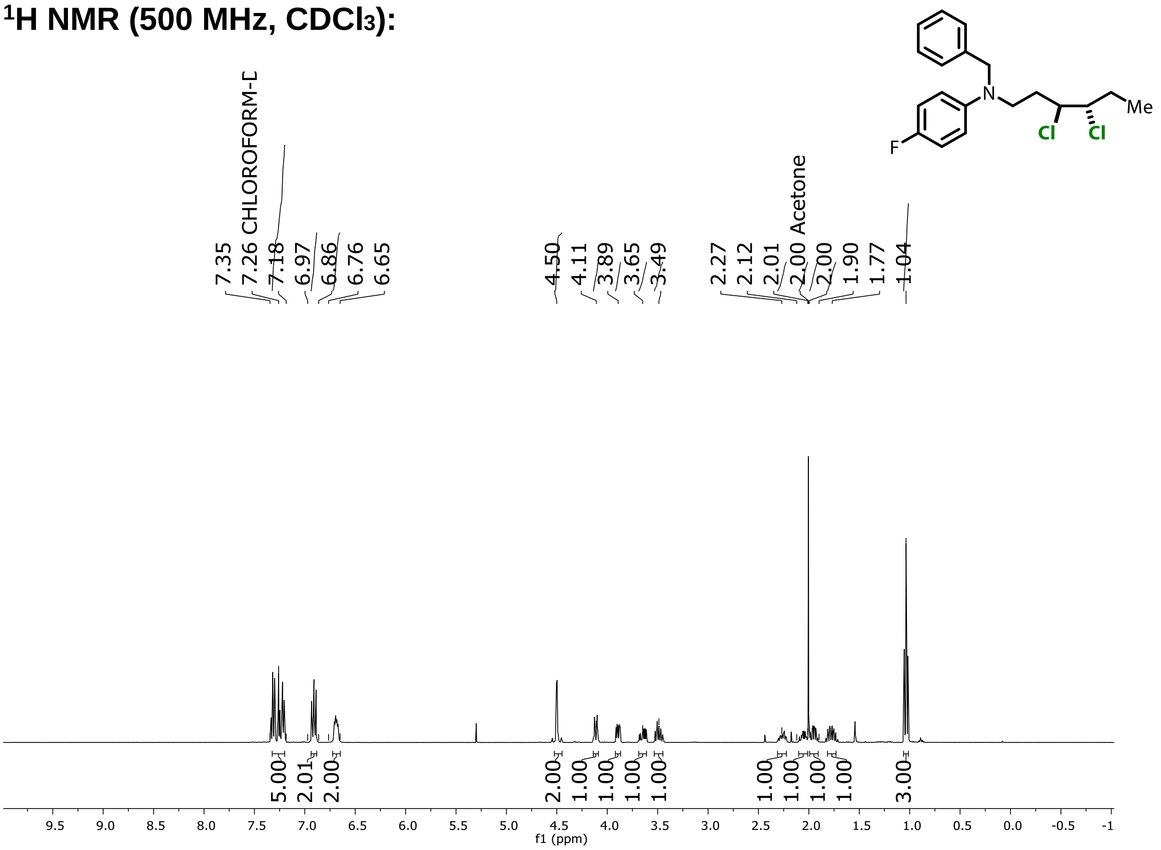

^{19}F NMR (376 MHz, $CDCl_3$):

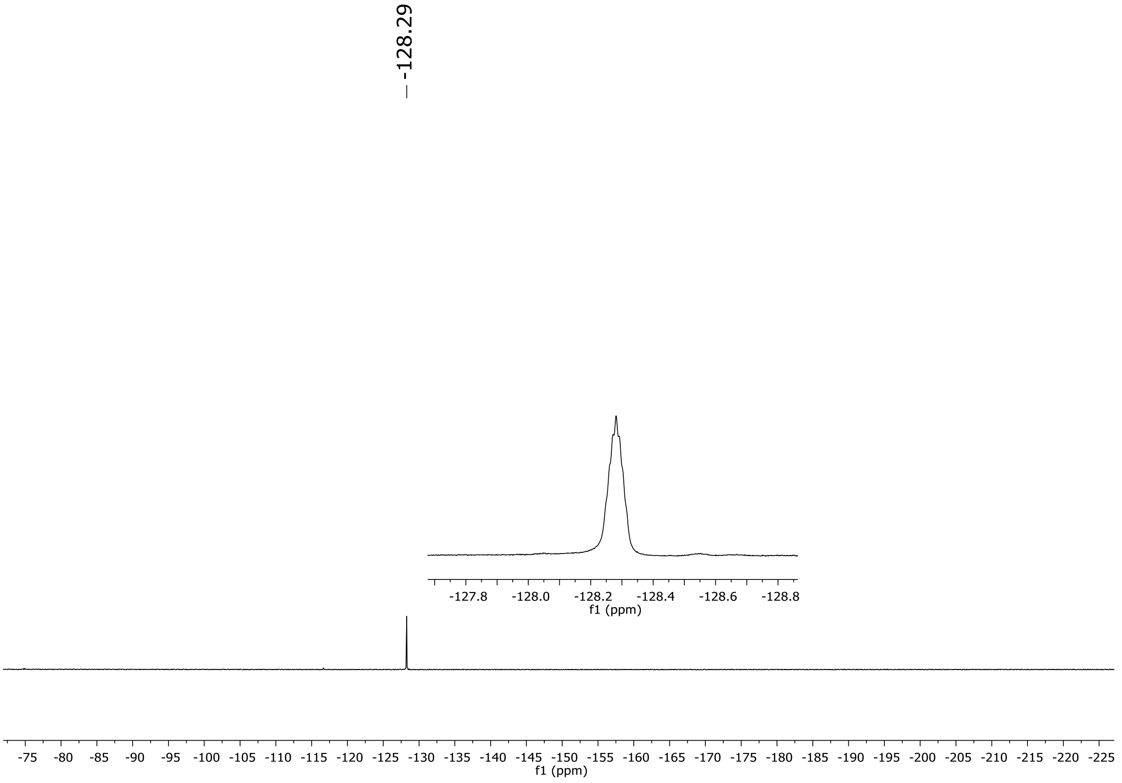

^{13}C {^{1}H} NMR (125 MHz, $CDCl_3$):

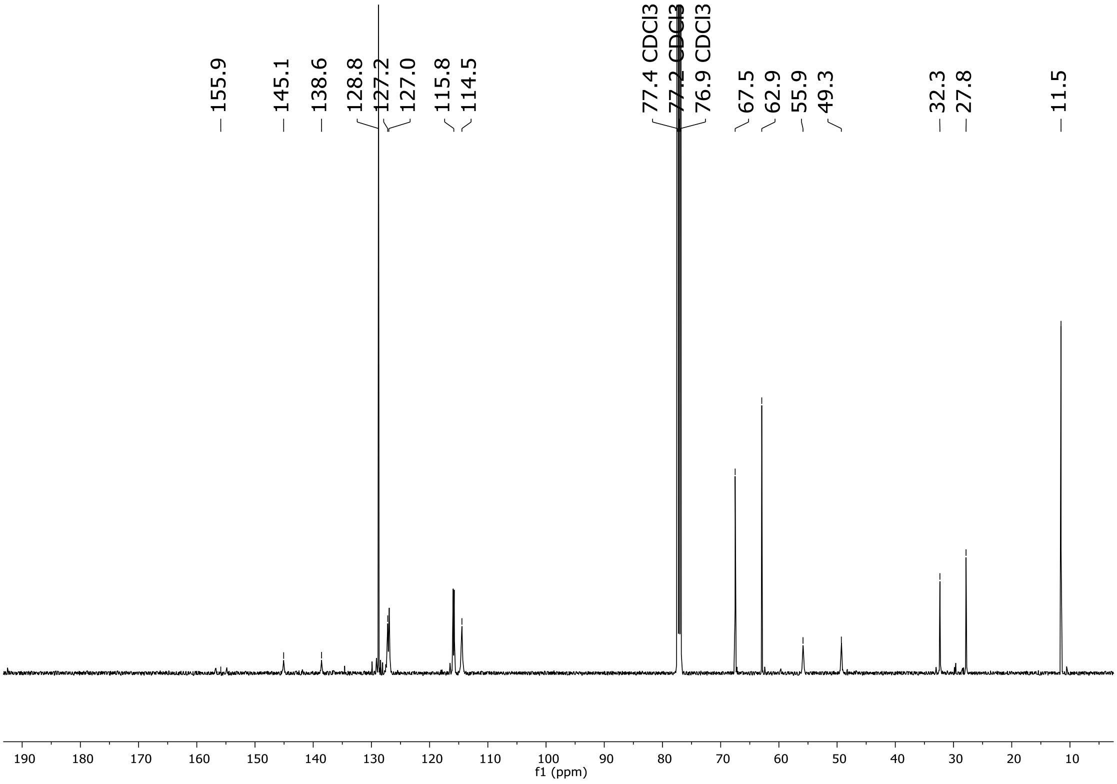

N-benzyl-N-((3S,4R)-3,4-dichlorohexyl)-4-fluoroaniline, 1h

^{1}H NMR (500 MHz, $CDCl_3$):

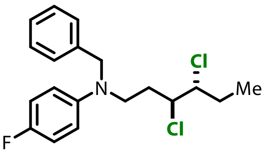

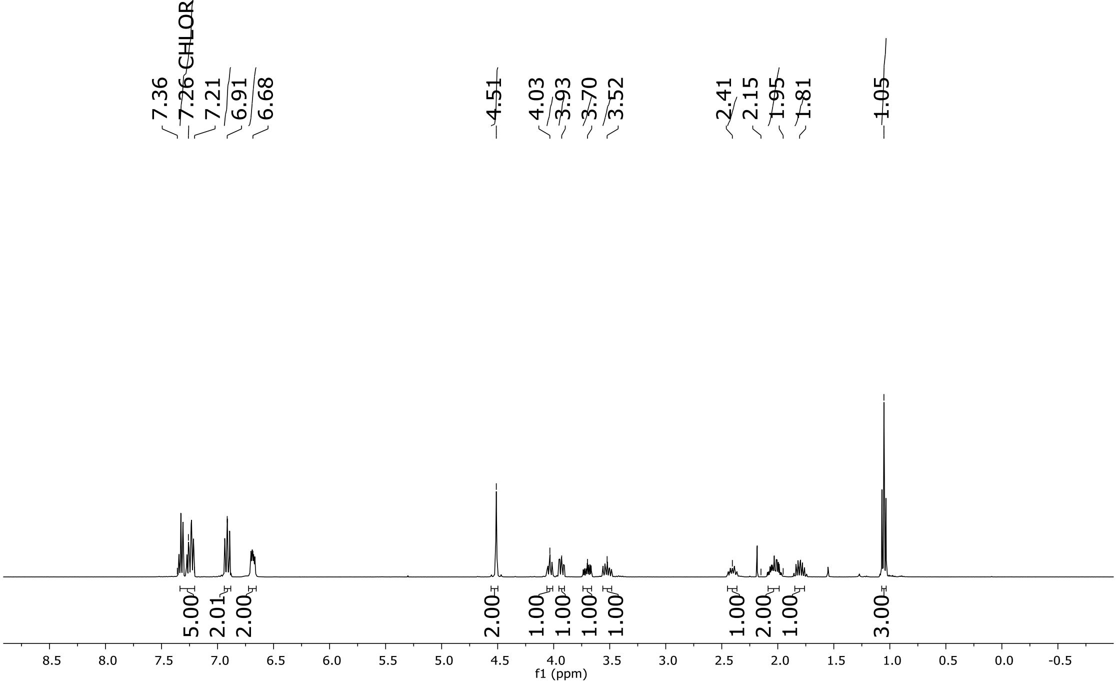

^{19}F NMR (376 MHz, $CDCl_3$):

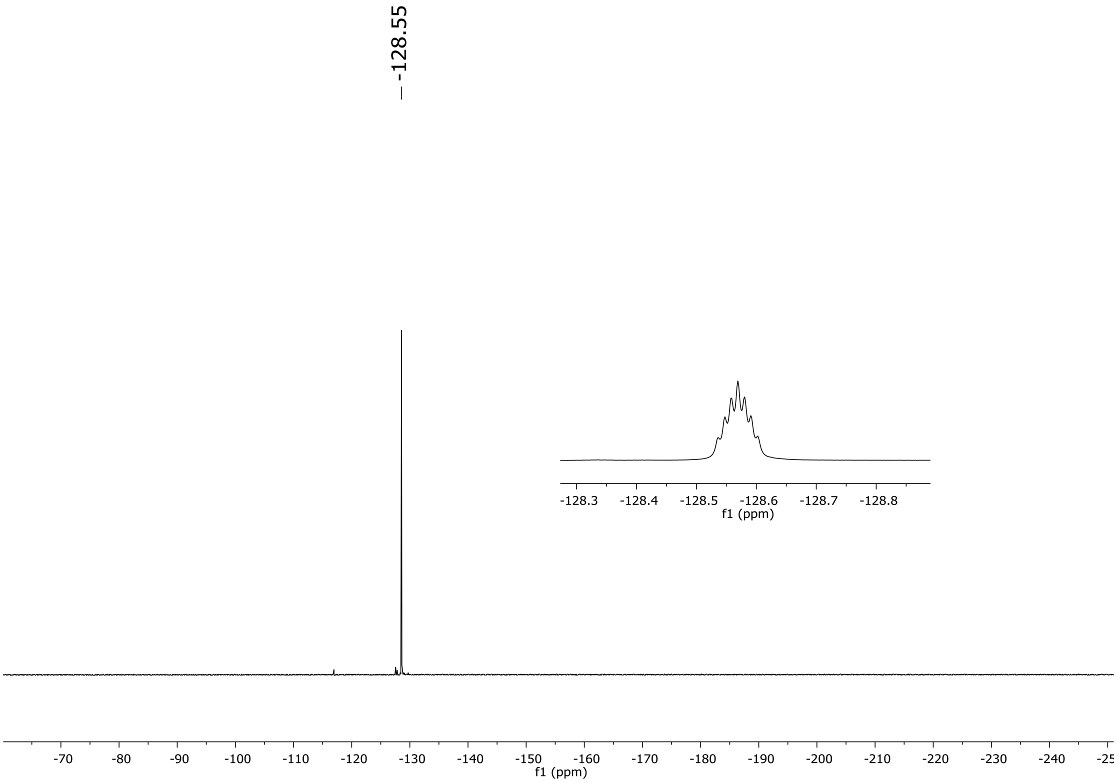

$^{13}C\ \{^1H\}$ NMR (125 MHz, $CDCl_3$):

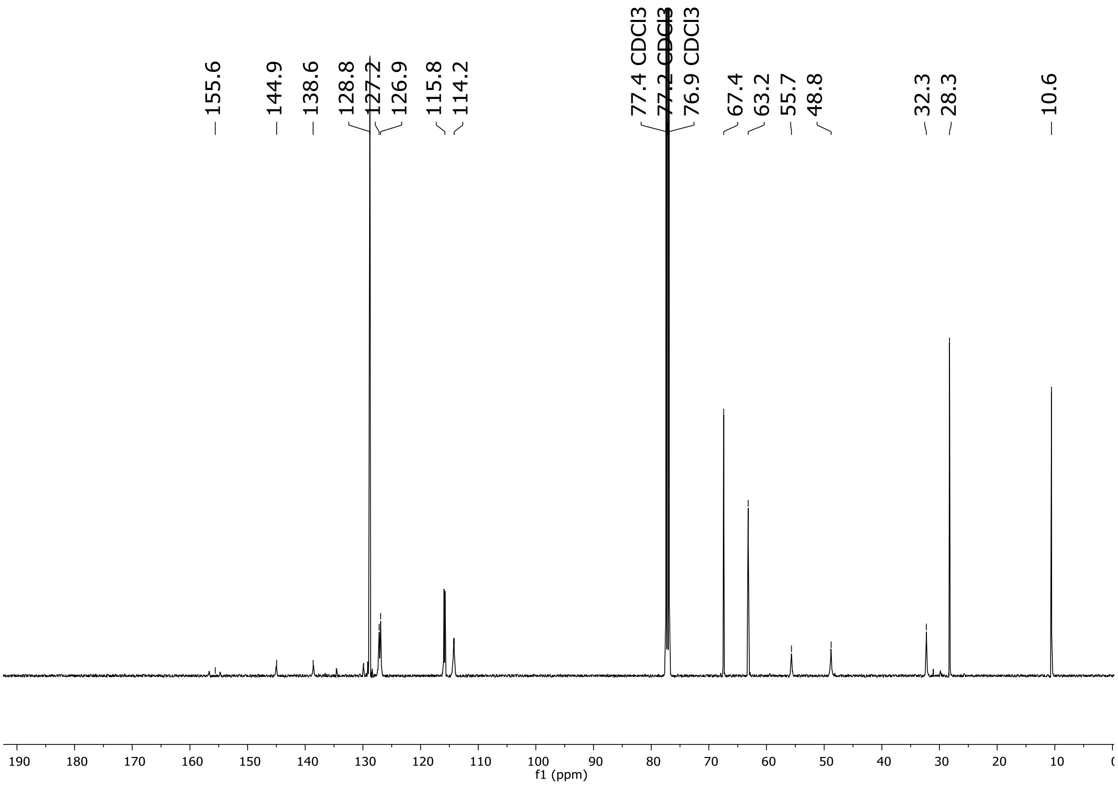

N-benzyl-N-((3R,4S)-3,4-difluorohexyl)-4-fluoroaniline, 1g

^{1}H NMR (400 MHz, $CDCl_3$):

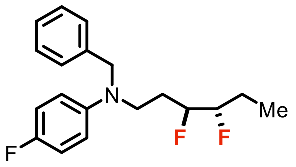

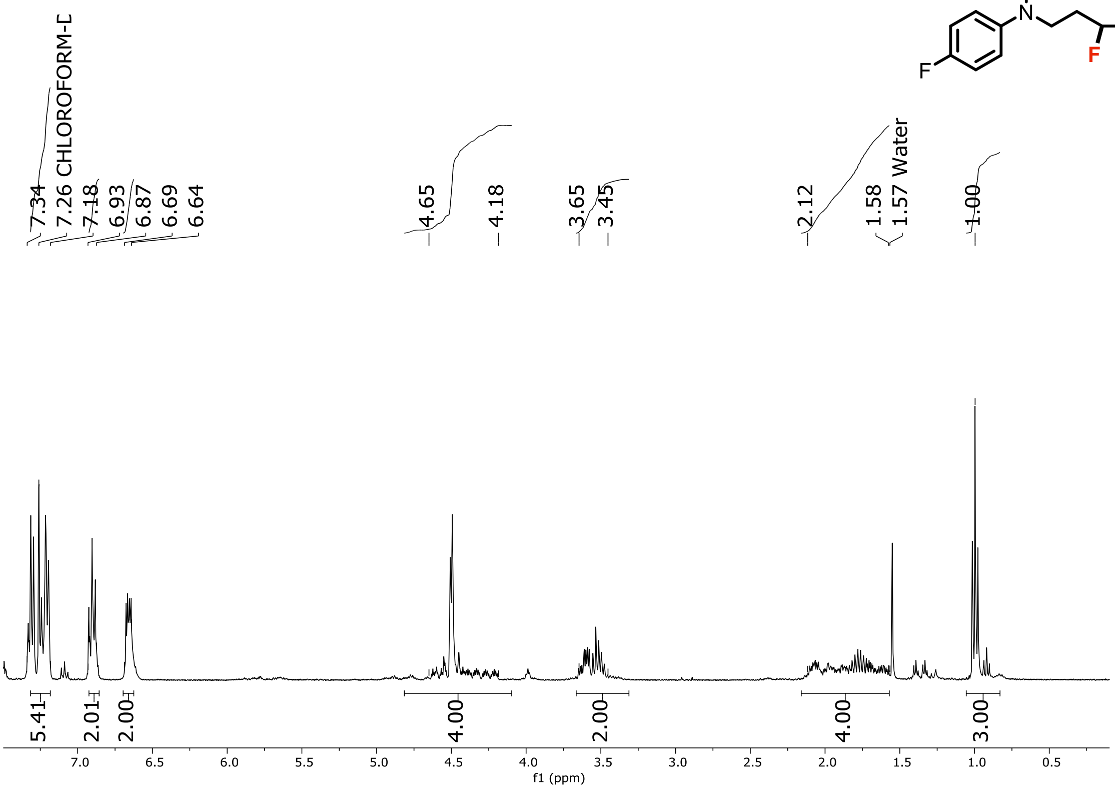

^{19}F NMR (376 MHz, $CDCl_3$):

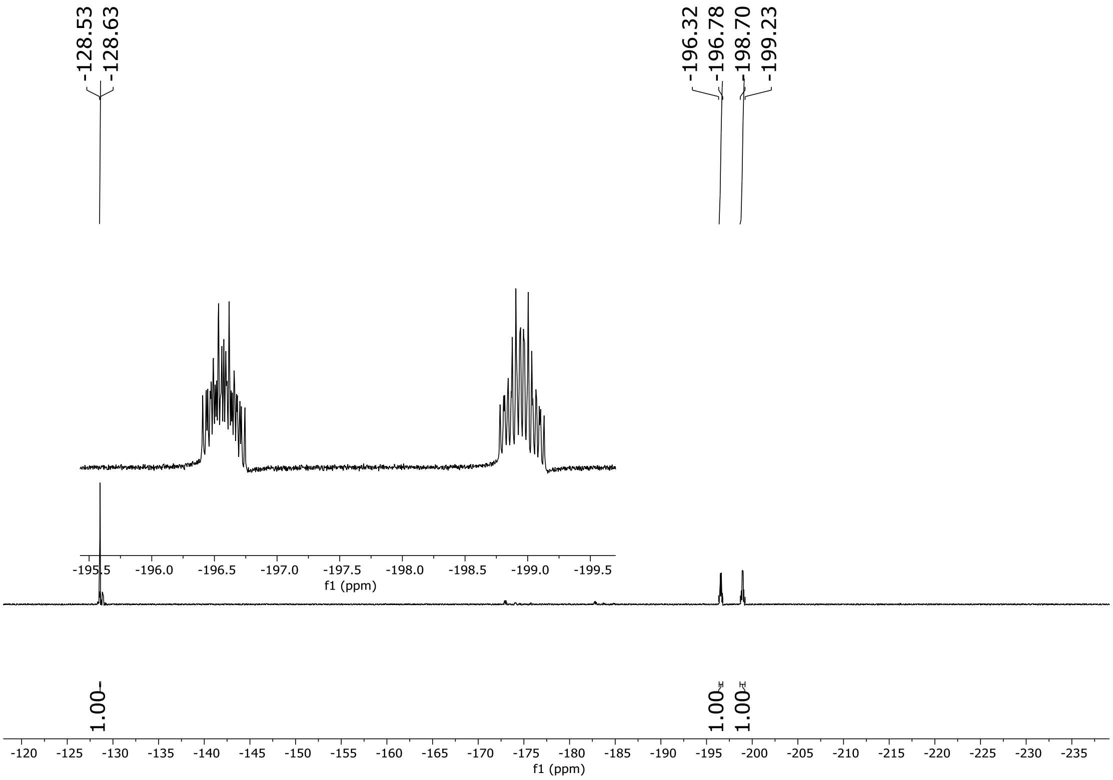

$^{13}C\ \{^{1}H\}$ NMR (100 MHz, $CDCl_3$):

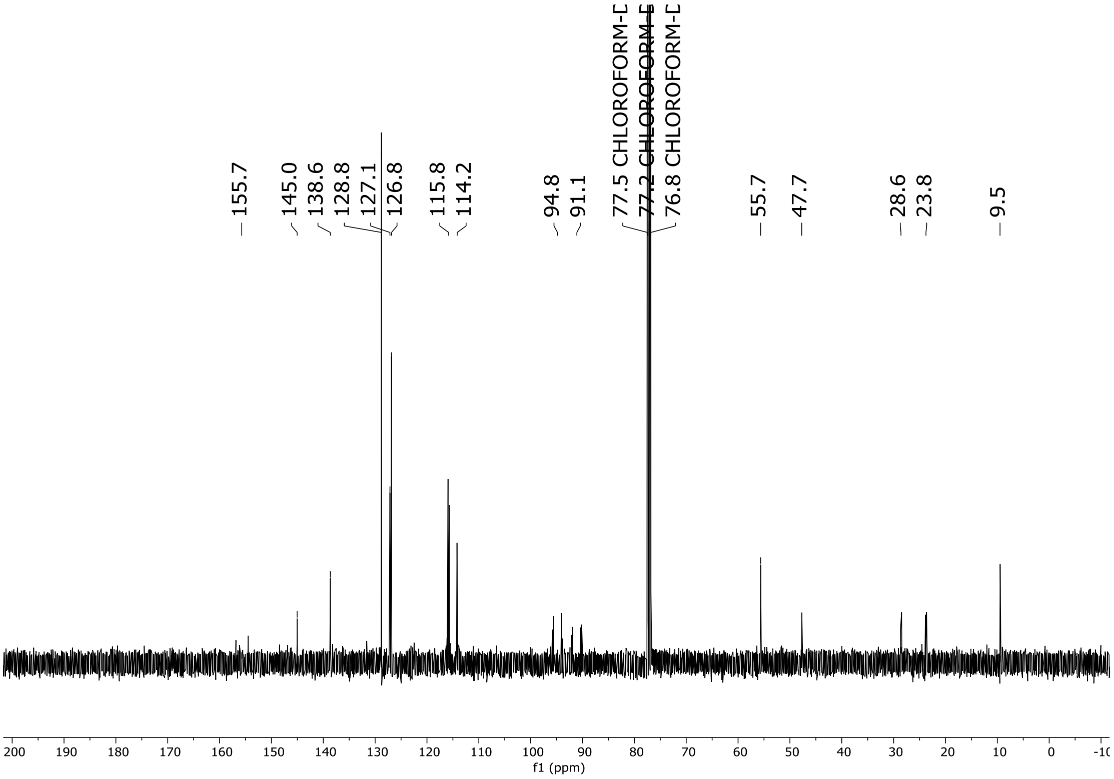

N-benzyl-N-((3S,4S)-3,4-difluorohexyl)-4-fluoroaniline, 1i

^{1}H NMR (400 MHz, CDCl$_3$):

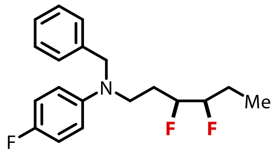

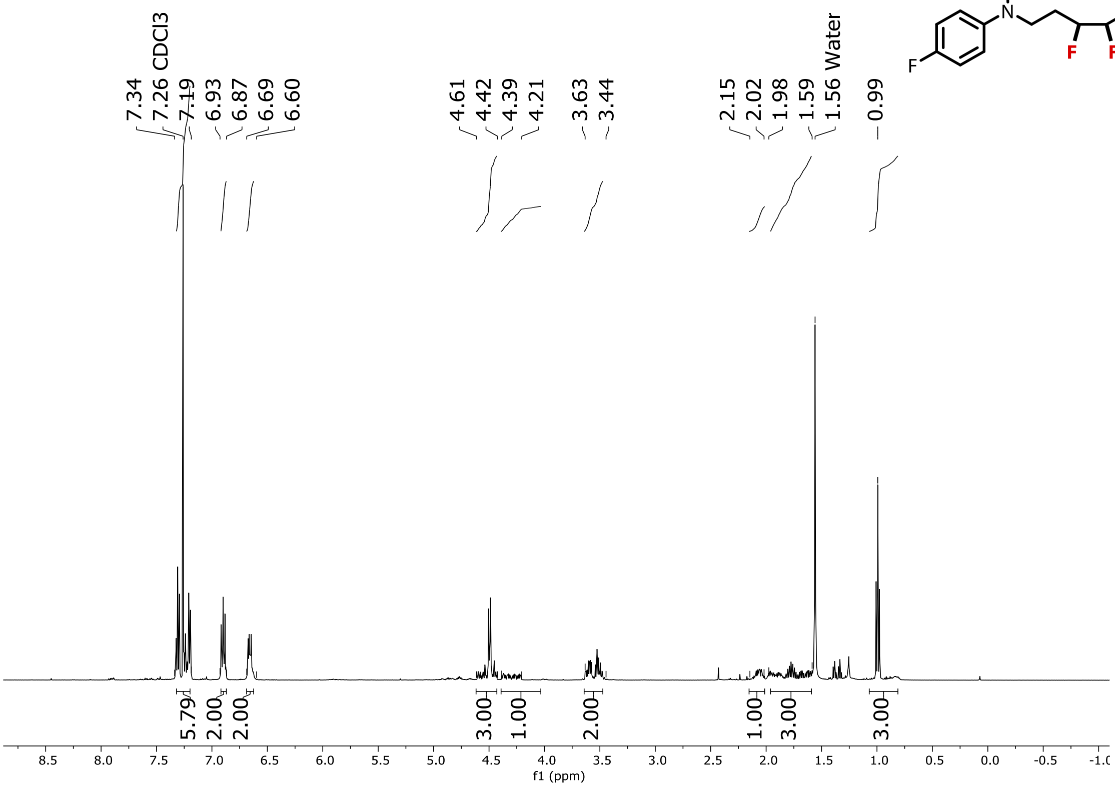

^{19}F NMR (376 MHz, CDCl$_3$):

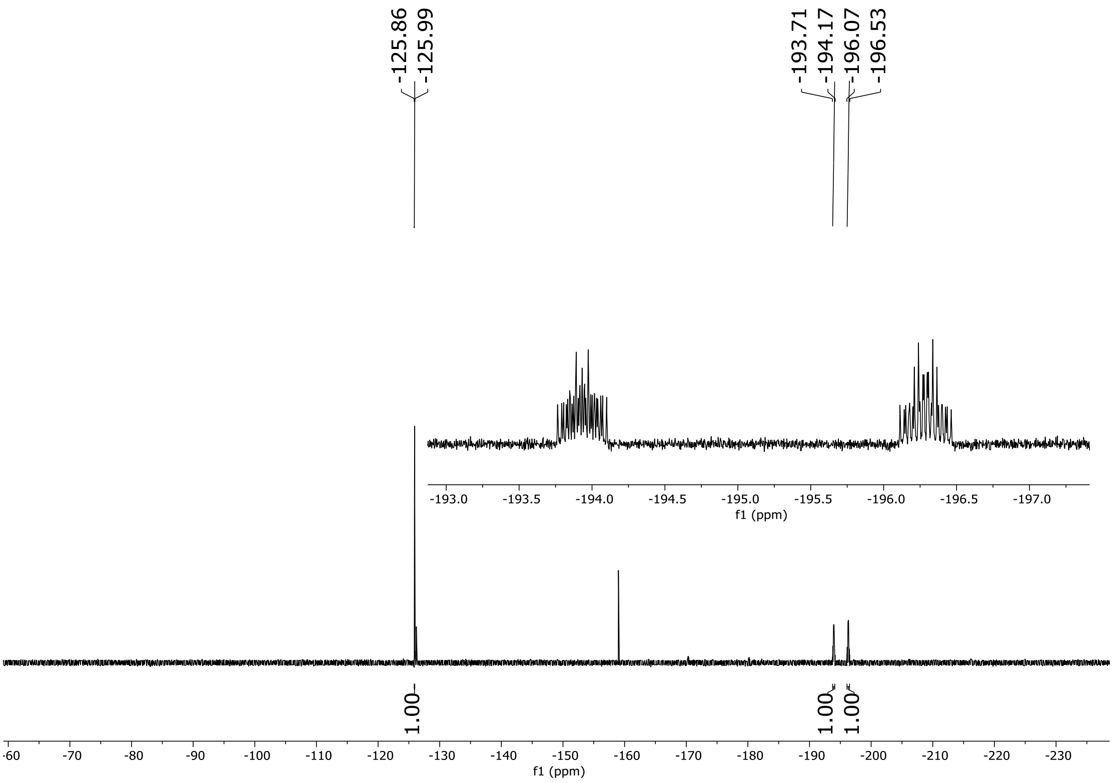

^{13}C {^{1}H} NMR (100 MHz, $CDCl_3$):

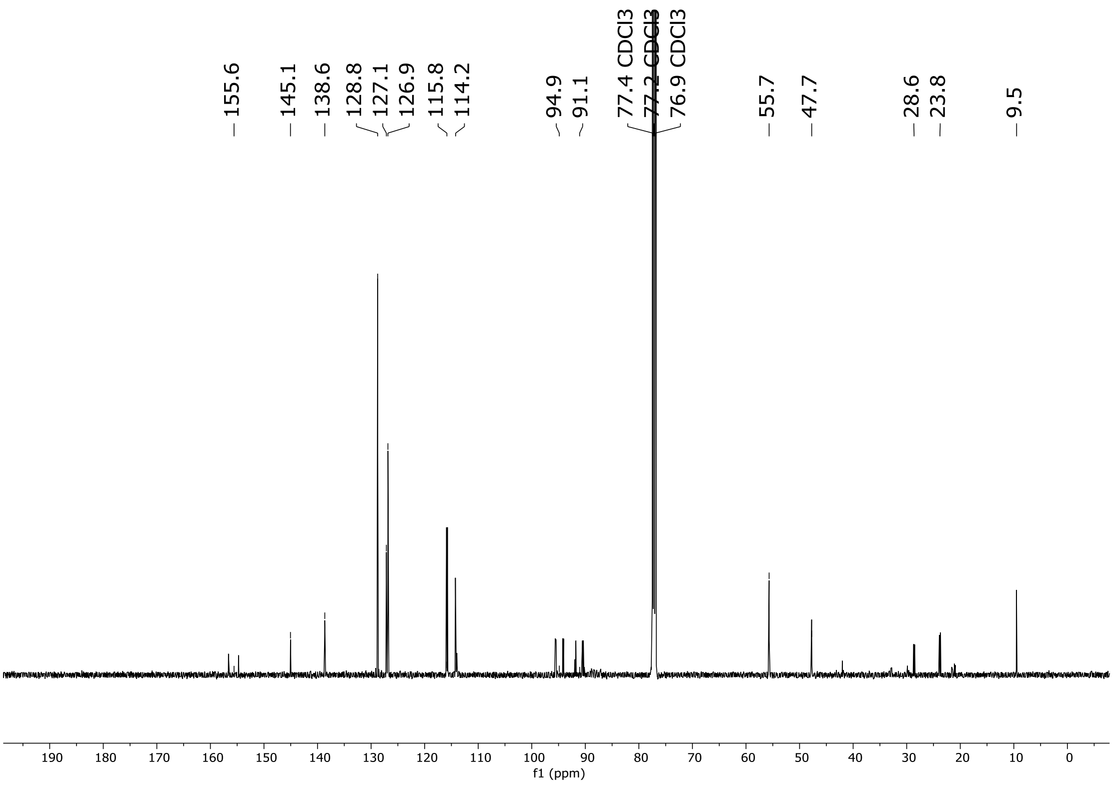

NMR spectra of substrates

N-allyl-N-(4-fluoro-2-methylphenyl)-4-nitrobenzenesulfonamide, 3a

^{1}H NMR (400 MHz, $CDCl_3$):

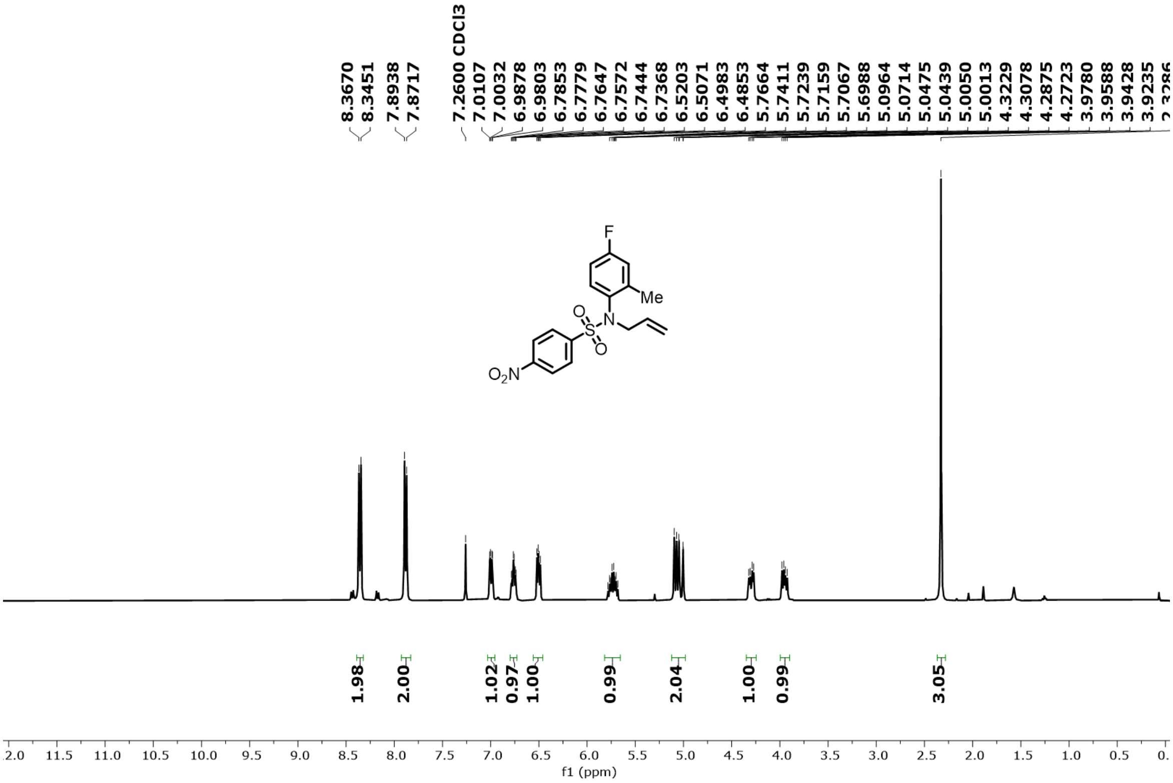

^{13}C NMR (101 MHz, $CDCl_3$):

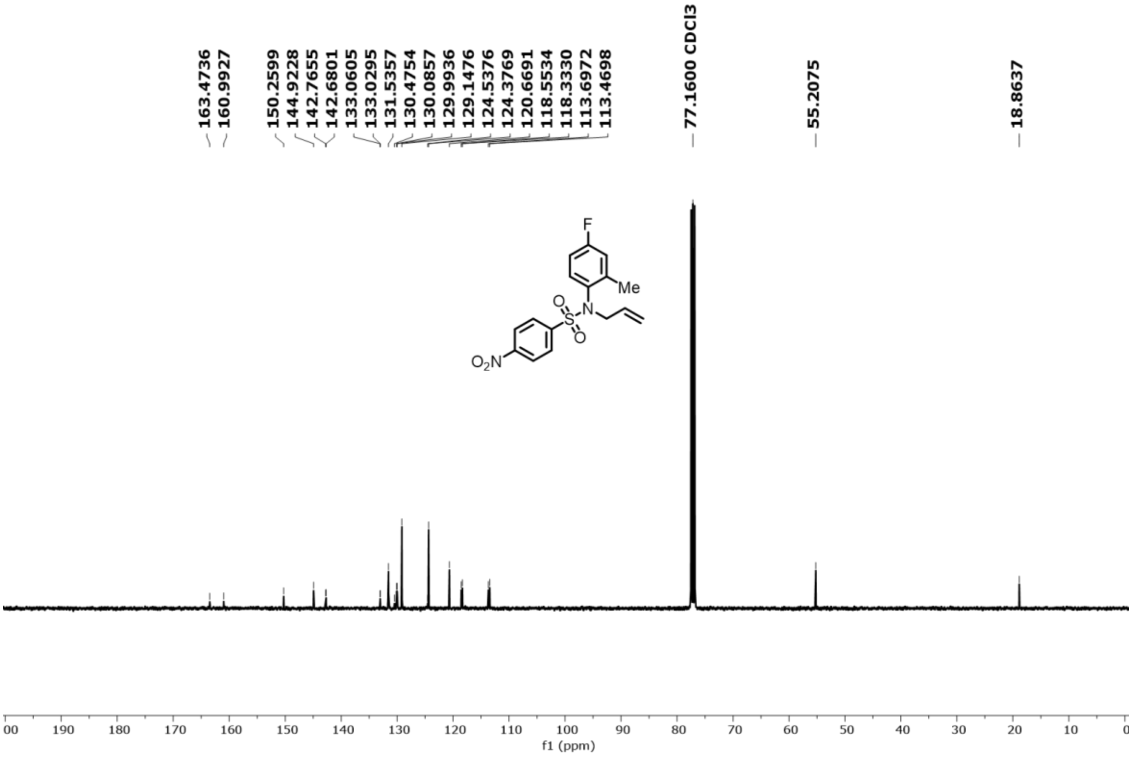

N-allyl-N-(4-methylisoxazol-3-yl)-4-nitrobenzenesulfonamide, 4a

^{1}H NMR (500 MHz, $CDCl_3$):

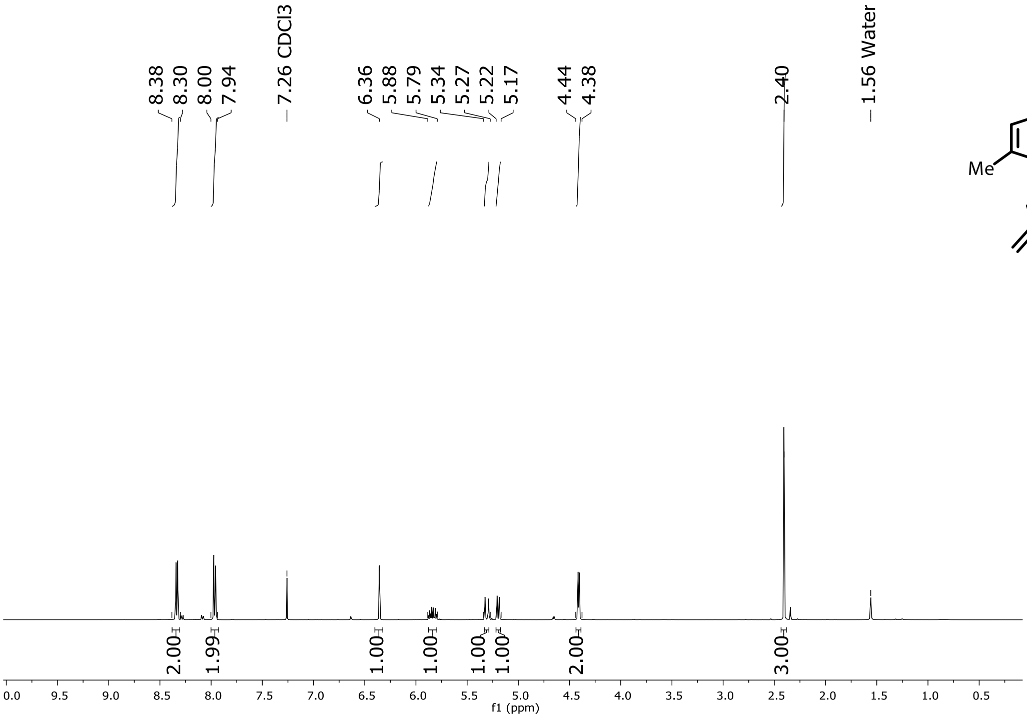

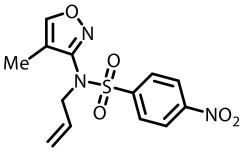

^{13}C {^{1}H} NMR (125 MHz, $CDCl_3$):

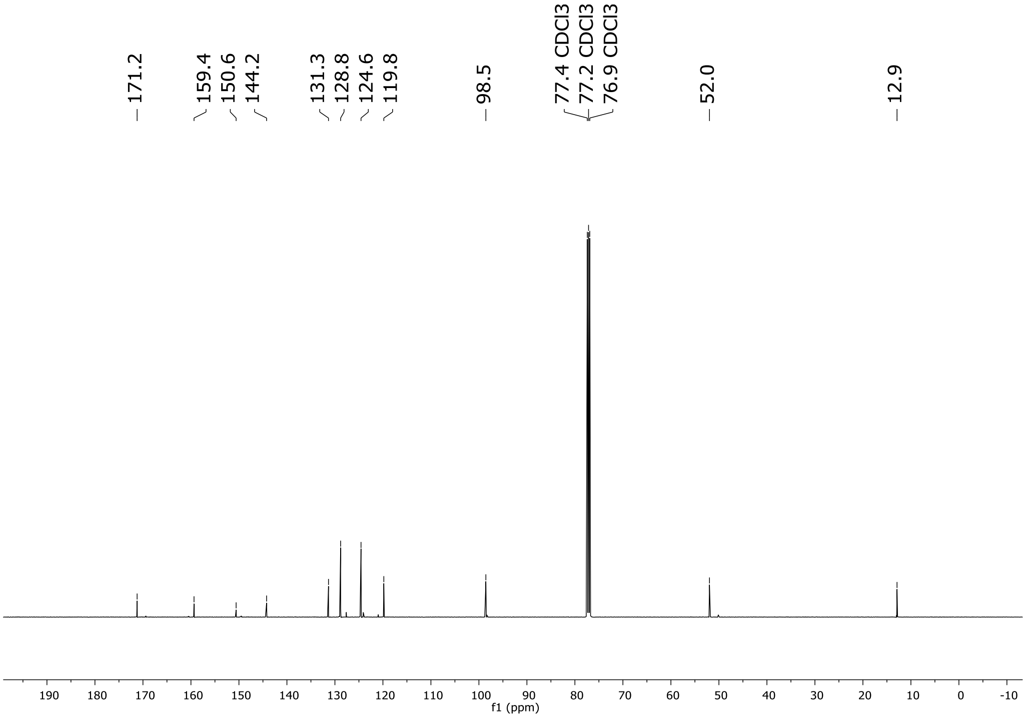

4-allyl-N,N-dimethylaniline, 5a

^{1}H NMR (400 MHz, $CDCl_3$):

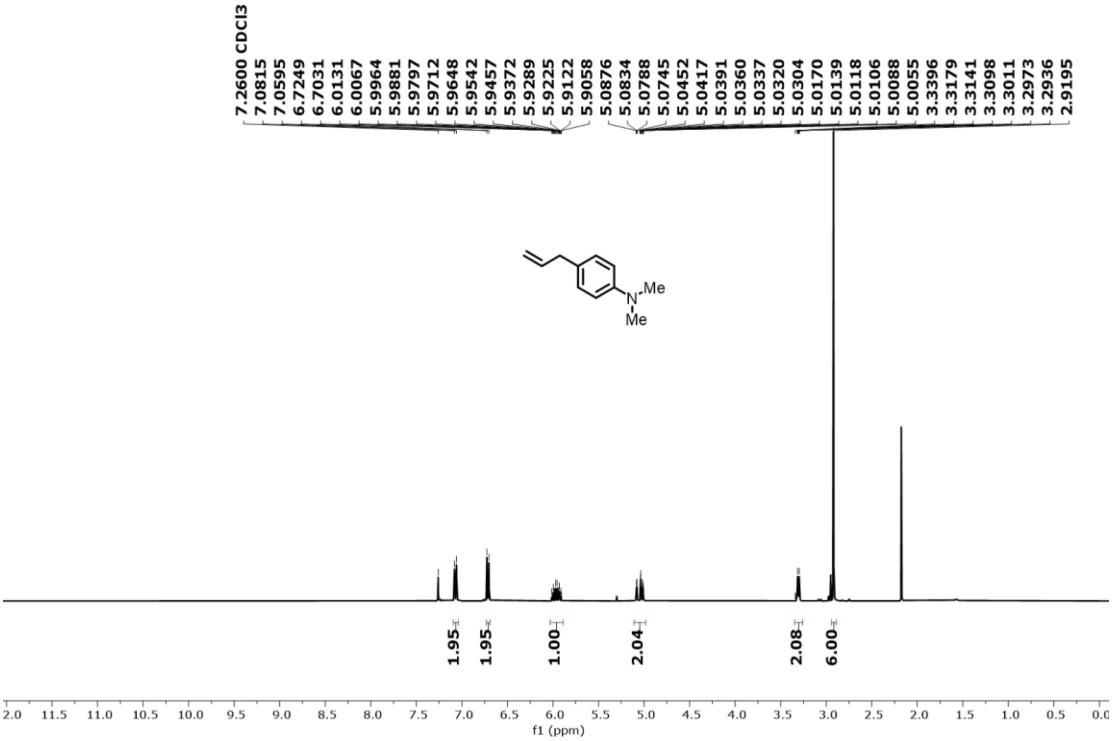

^{13}C NMR (101 MHz, $CDCl_3$):

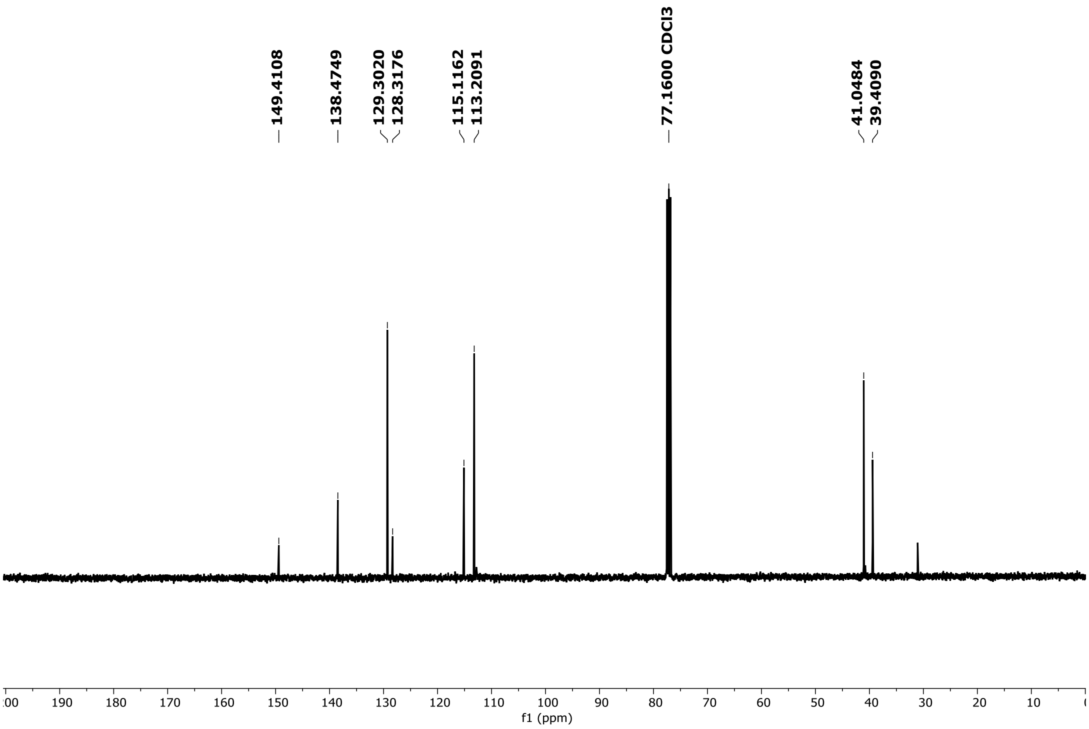

2-(undec-10-en-1-yl)isoindoline-1,3-dione, 9a
^{1}H NMR (400 MHz, $CDCl_3$):

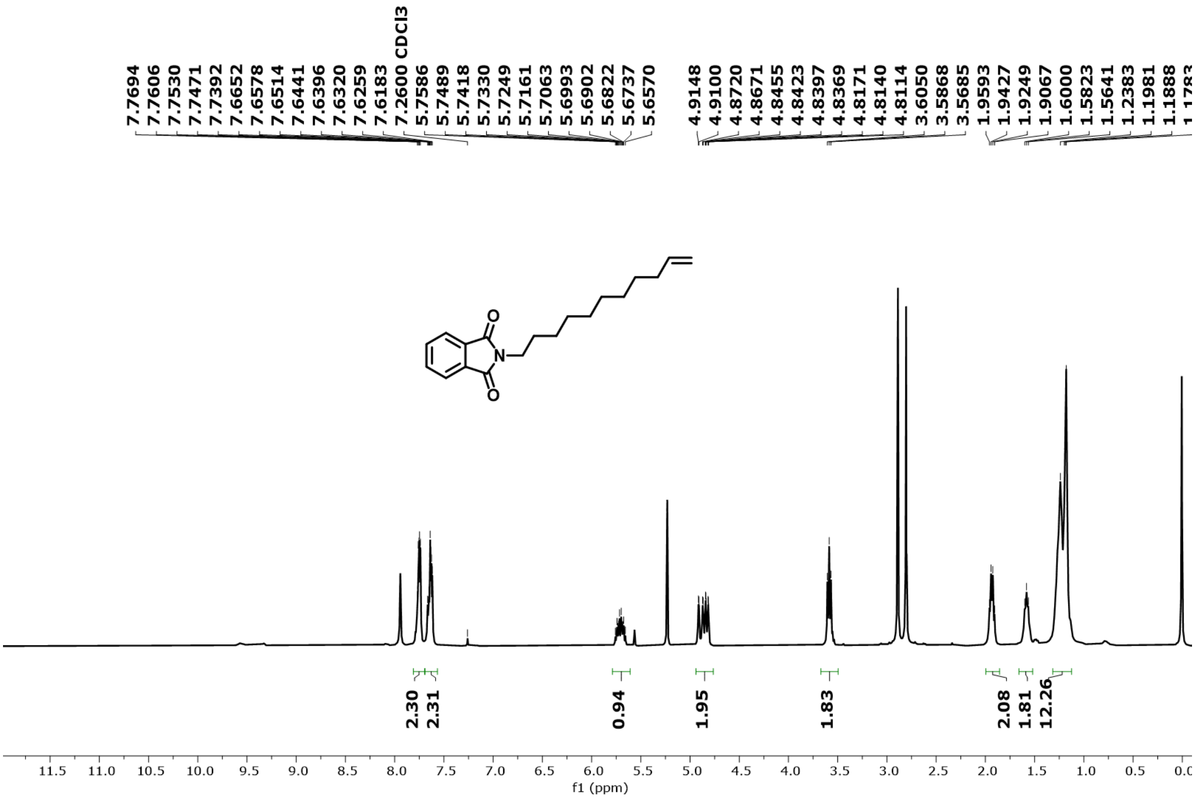

(S)-quinolin-4-yl((1S,2S,4S,5R)-5-vinylquinuclidin-2-yl)methyl acetate, 11a
^{1}H NMR (400 MHz, $CDCl_3$):

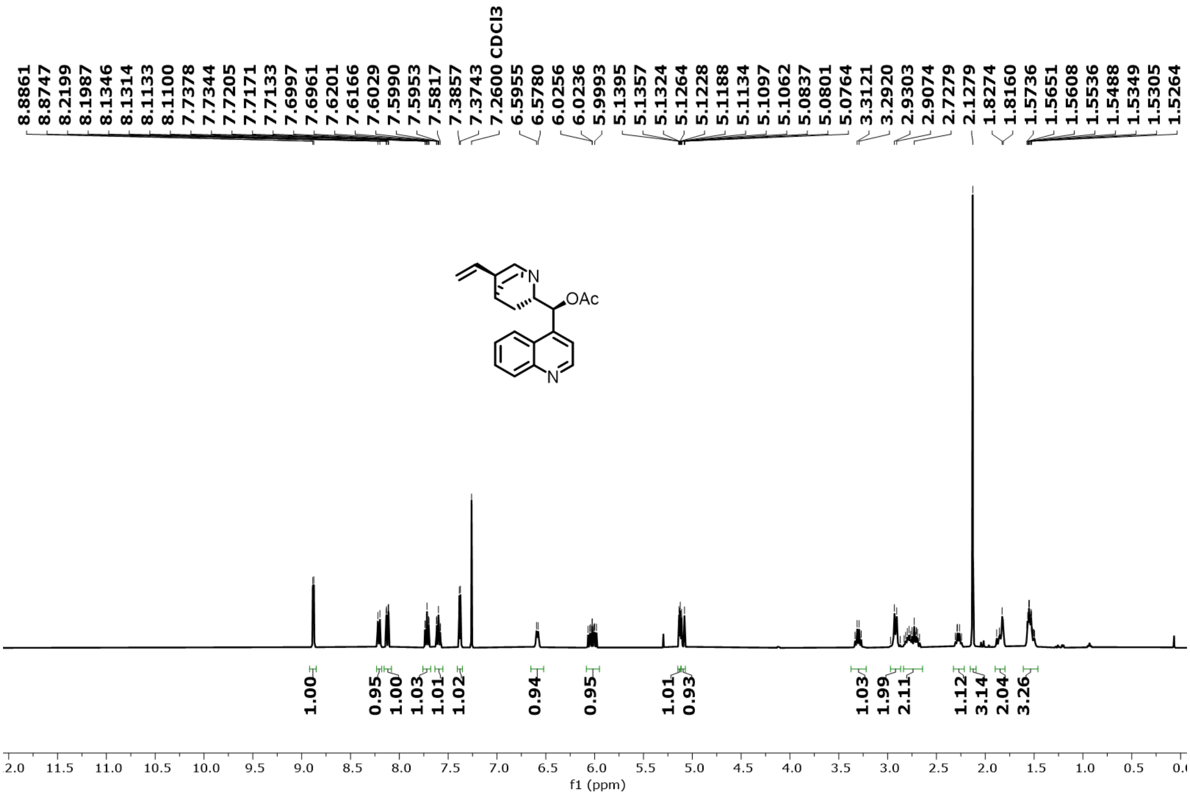

^{13}C {^{1}H} NMR (101 MHz, $CDCl_3$):

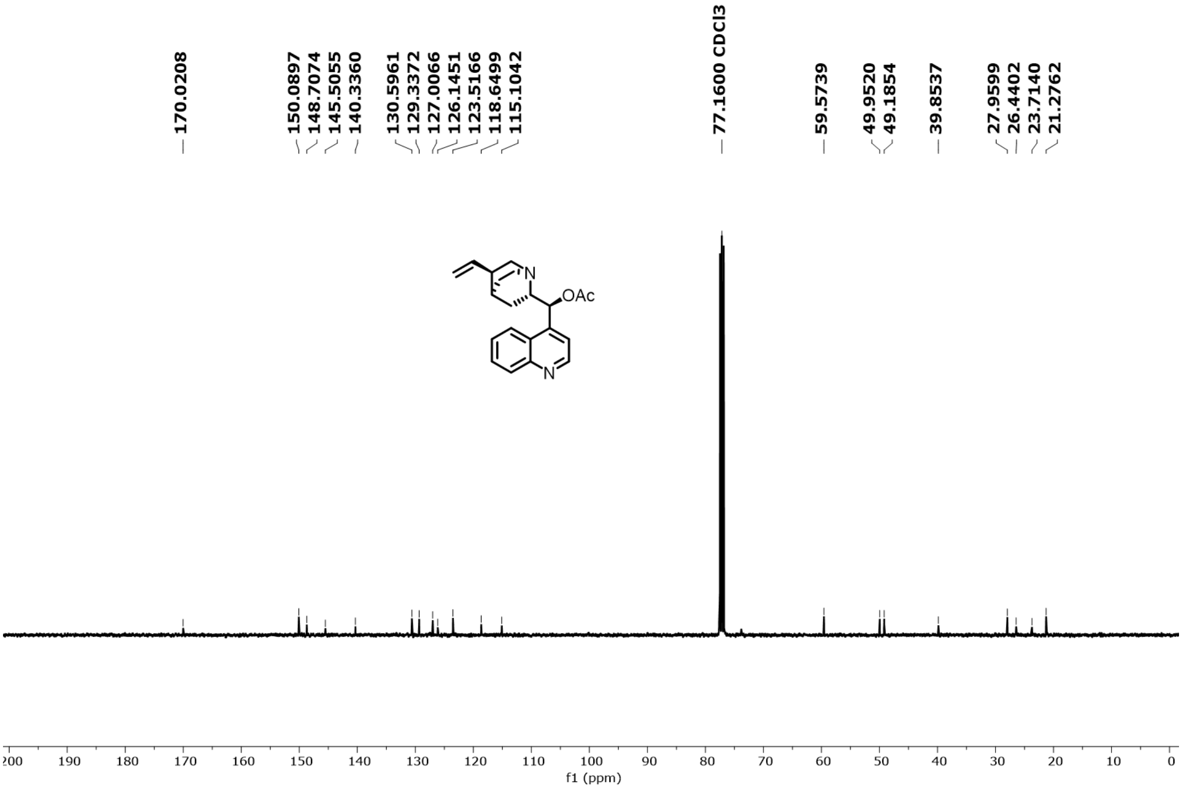

Hex-5-en-1-yl-3,5-dinitrobenzoate, 15a

^{1}H NMR (500 MHz, $CDCl_3$):

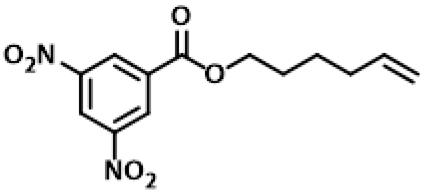

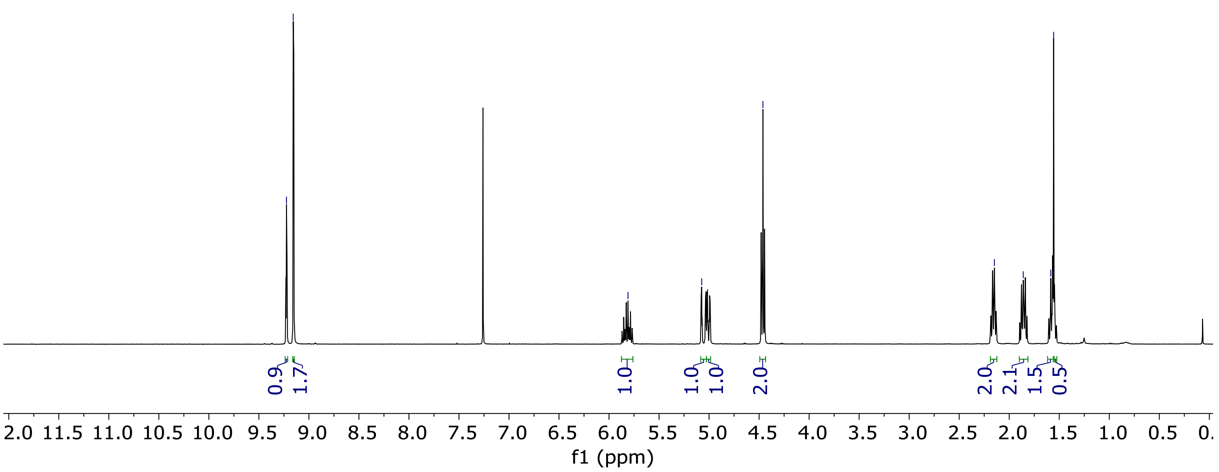

^{13}C {^{1}H} NMR (125 MHz, $CDCl_3$):

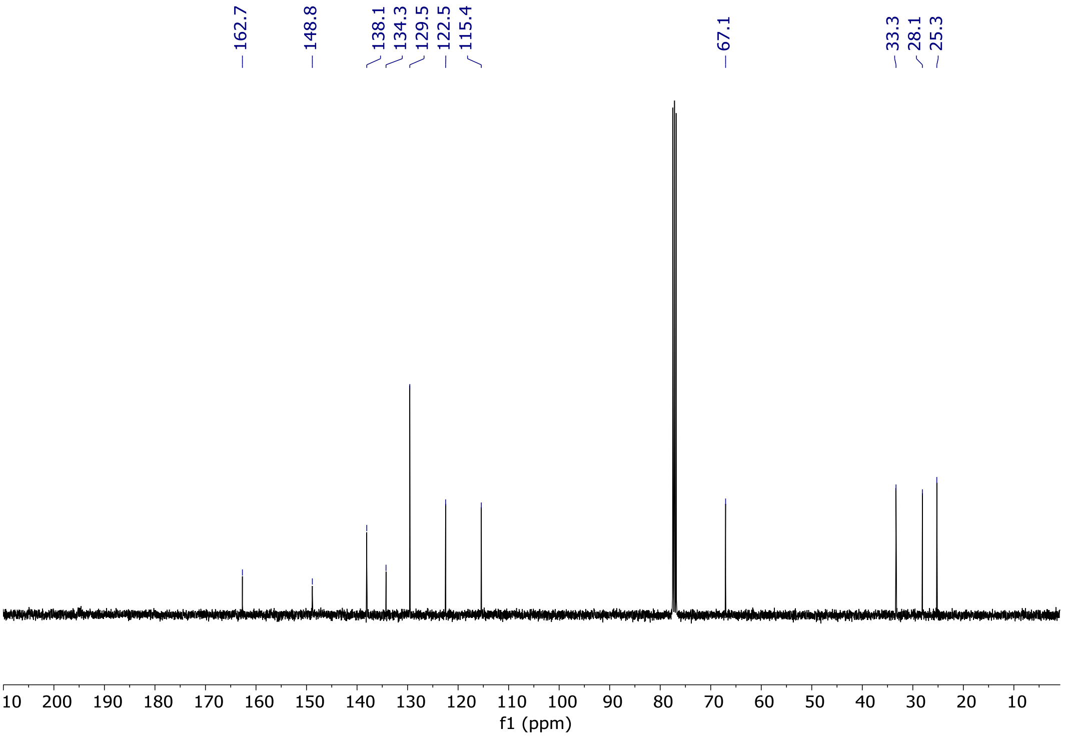

Back to top

(Z)-N-benzyl-N-(hex-3-en-1-yl)-4-(trifluoromethyl)aniline, 16a

^{1}H NMR (500 MHz, $CDCl_3$):

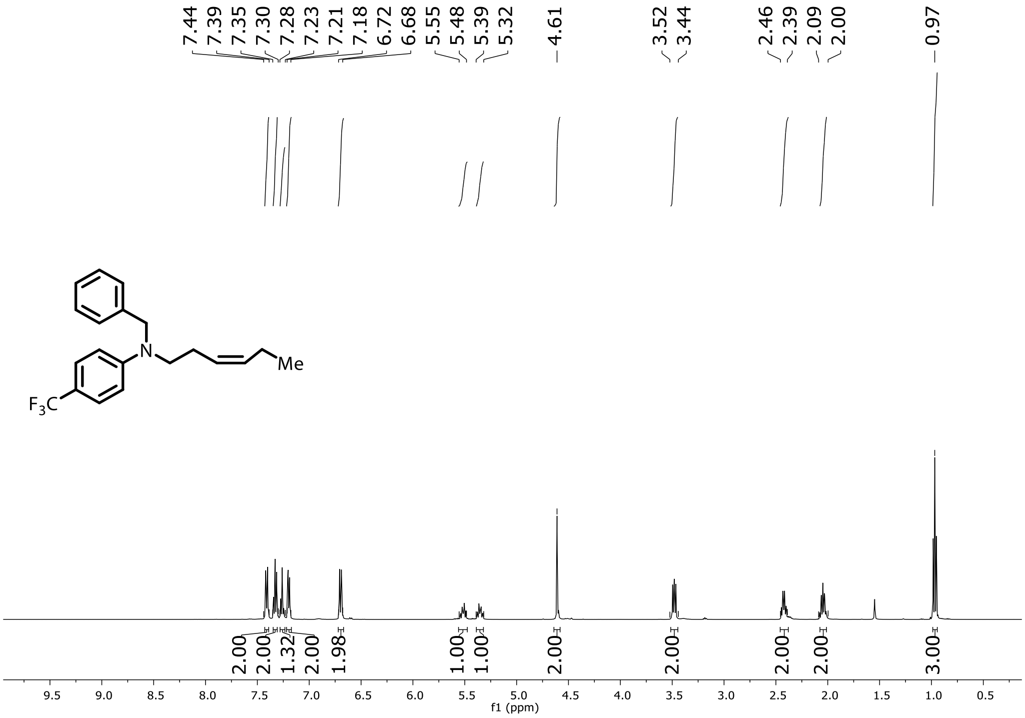

^{19}F NMR (376 MHz, $CDCl_3$):

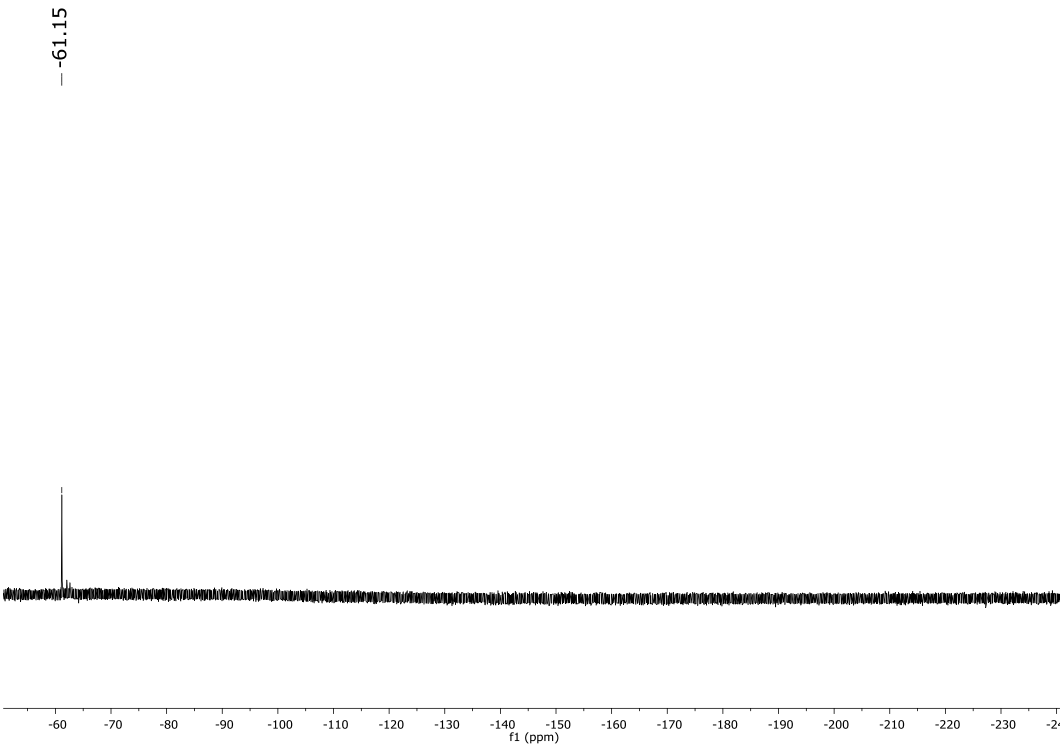

^{13}C {^{1}H} NMR (125 MHz, $CDCl_3$):

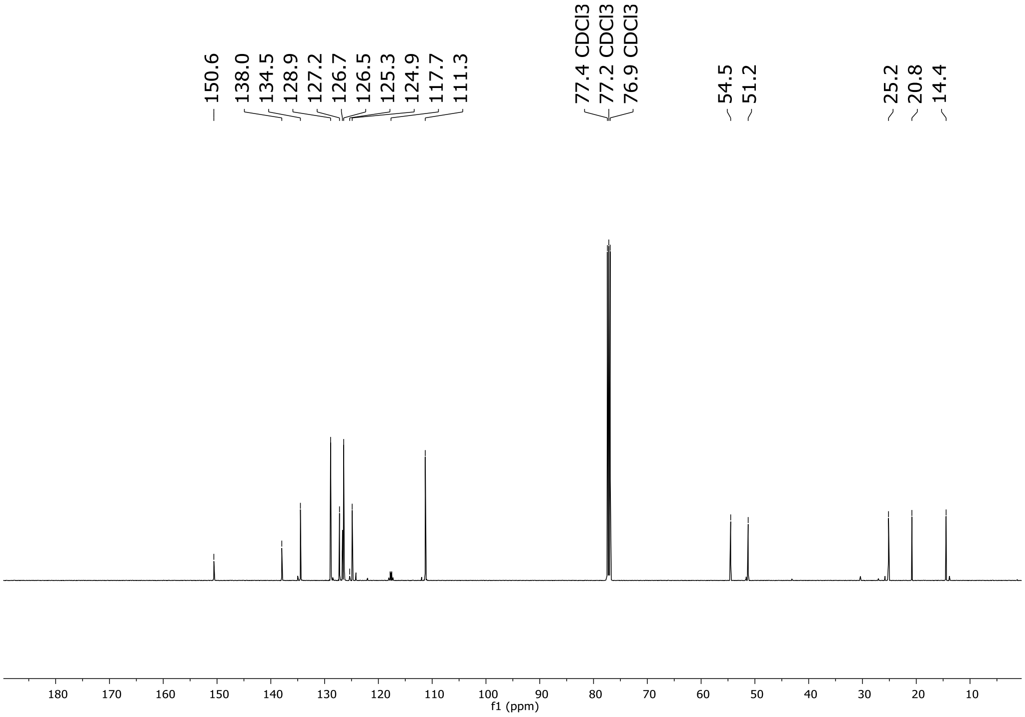

(Z)-N-benzyl-N-(hex-3-en-1-yl)-4-iodoaniline, 17a
^{1}H NMR (500 MHz, CDCl$_3$):

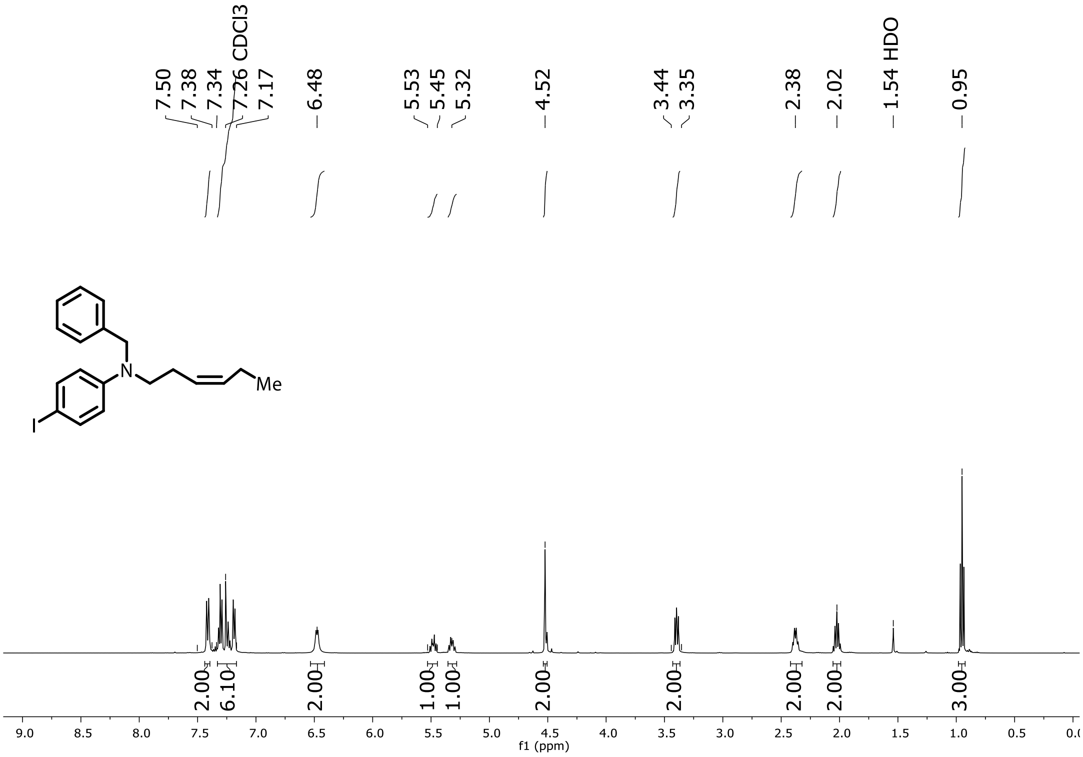

^{13}C {^{1}H} NMR (125 MHz, CDCl$_3$):

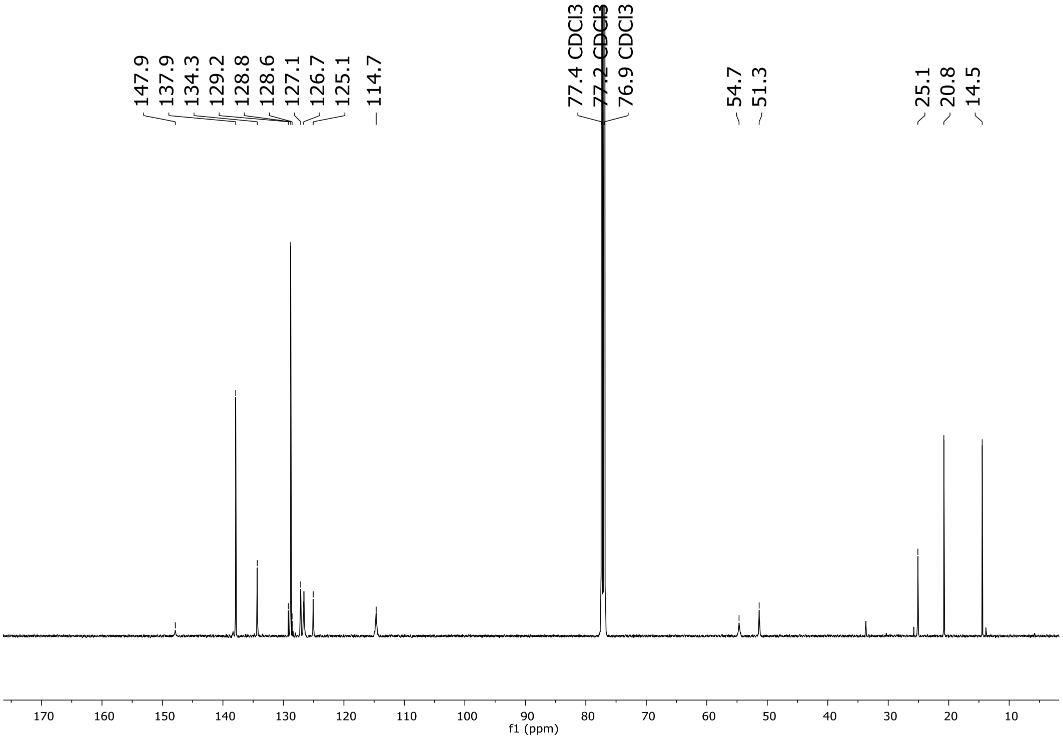

(Z)-N-benzyl-N-(hex-3-en-1-yl)aniline, 18a

^{1}H NMR (500 MHz, $CDCl_3$):

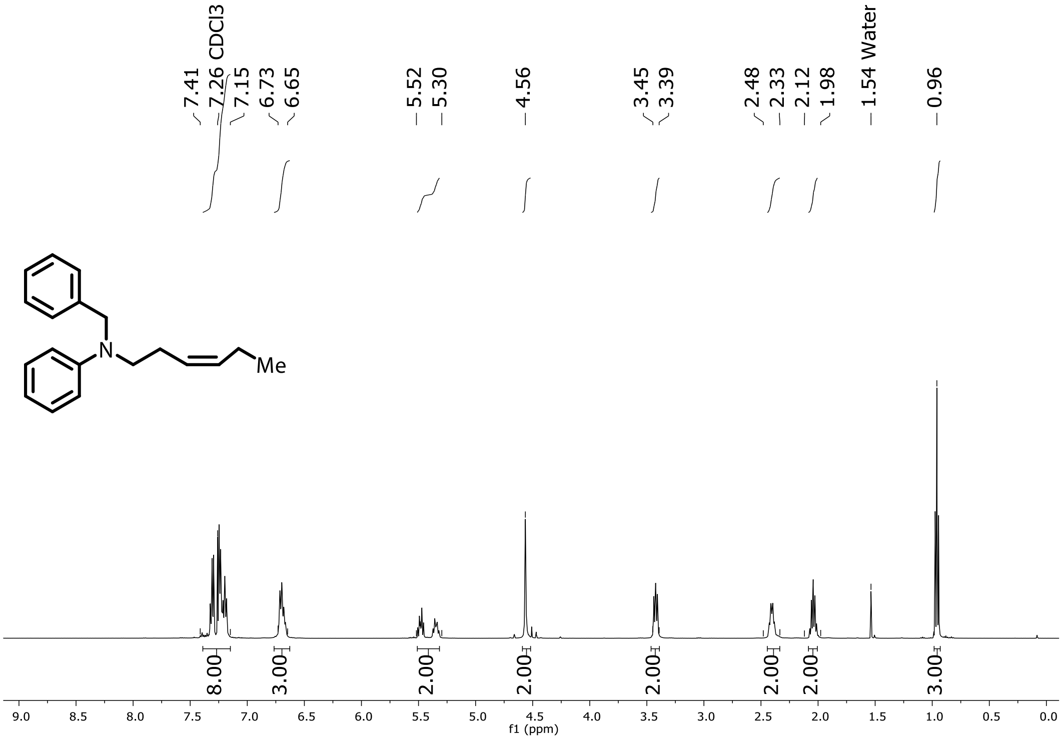

^{13}C {^{1}H} NMR (125 MHz, $CDCl_3$):

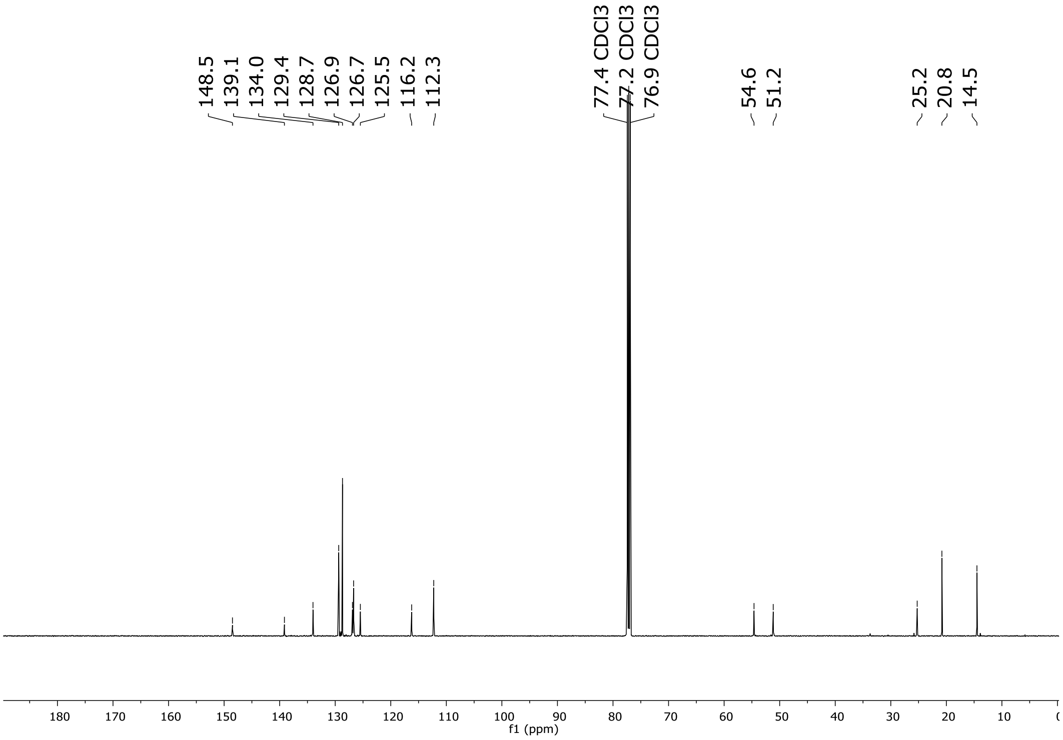

(Z)-N-benzyl-N-(hex-3-en-1-yl)-4-methylaniline, 19a

^{1}H NMR (400 MHz, $CDCl_3$):

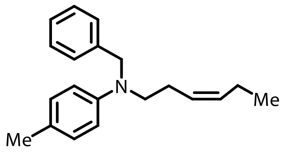

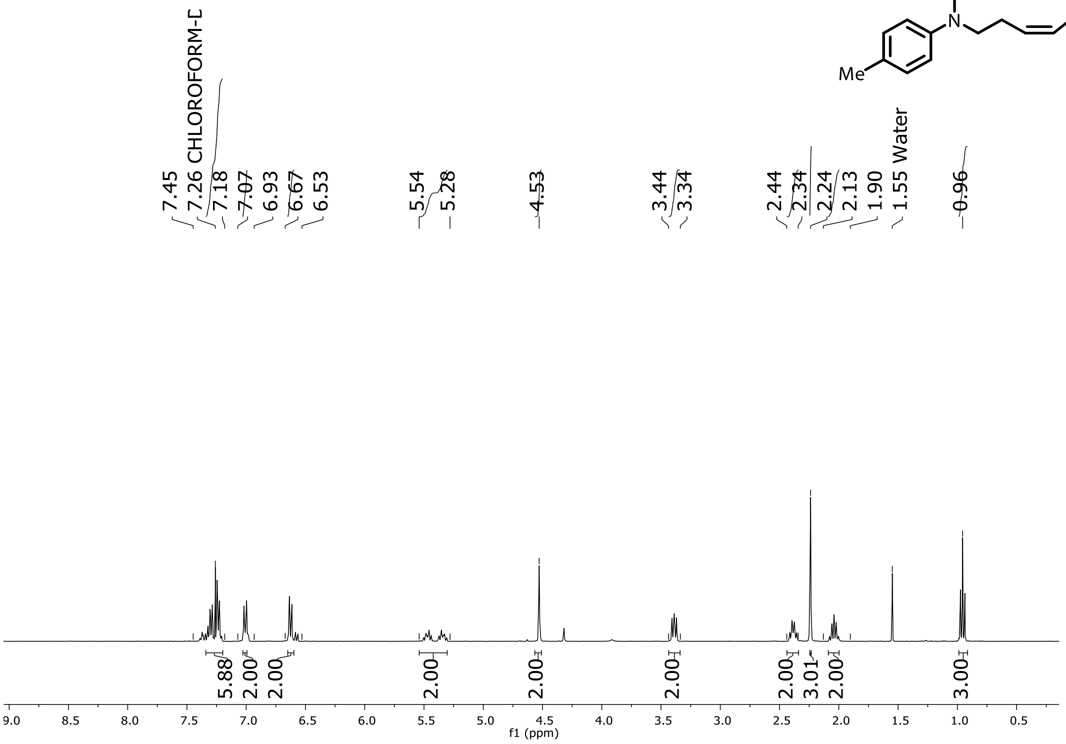

^{13}C {^{1}H} NMR (100 MHz, $CDCl_3$):

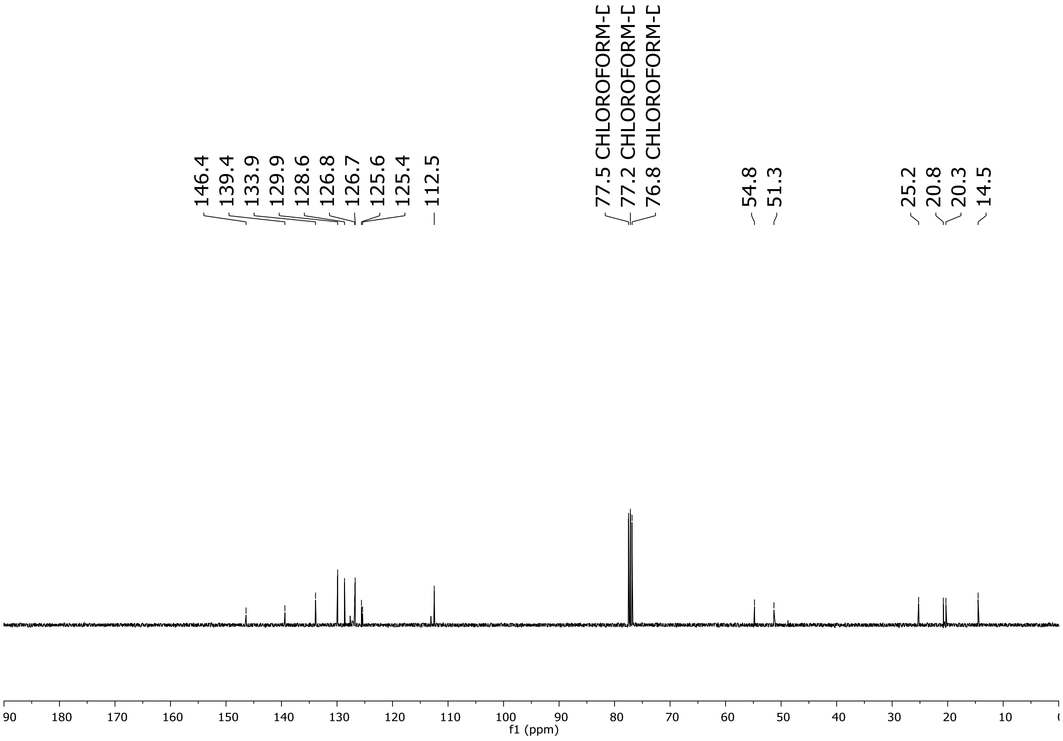

(Z)-4-fluoro-N-(hex-3-en-1-yl)aniline, 20a

^{1}H NMR (400 MHz, CDCl$_3$):

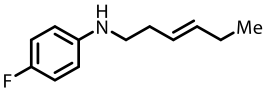

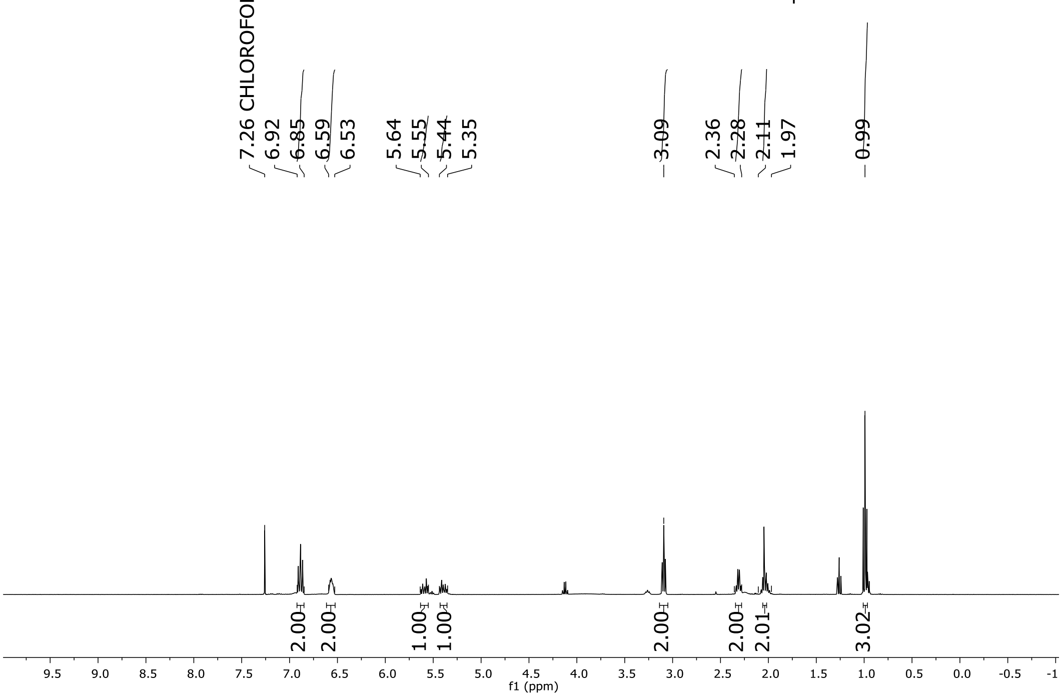

^{19}F NMR (376 MHz, CDCl$_3$):

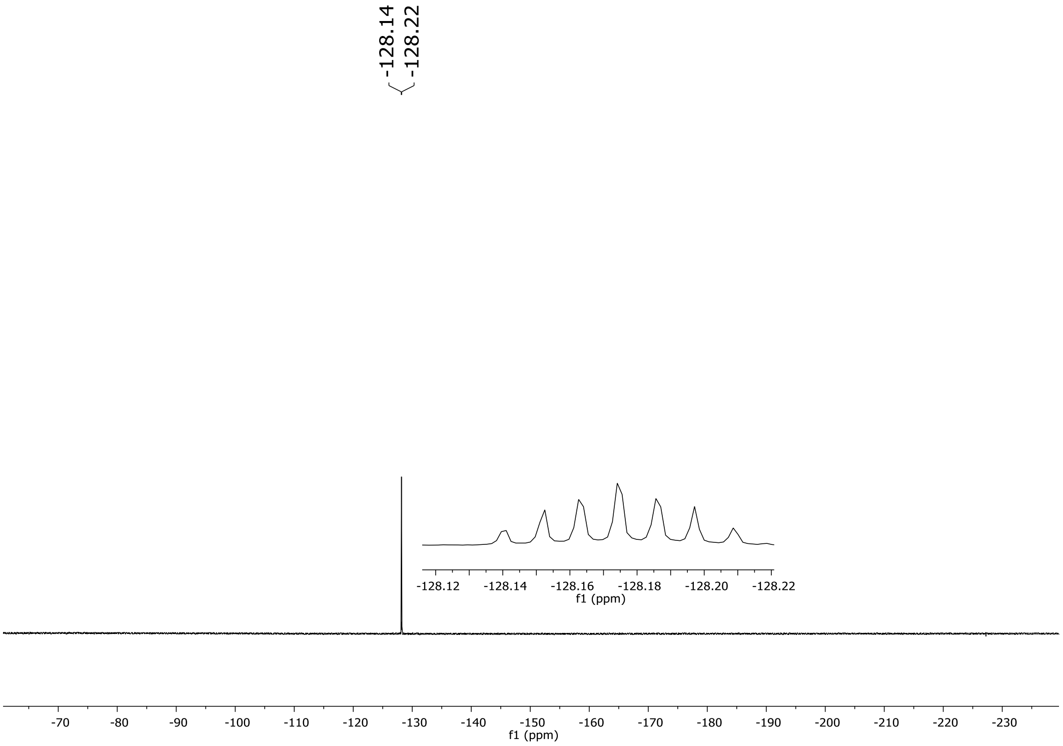

^{13}C {^{1}H} NMR (125 MHz, CDCl$_3$):

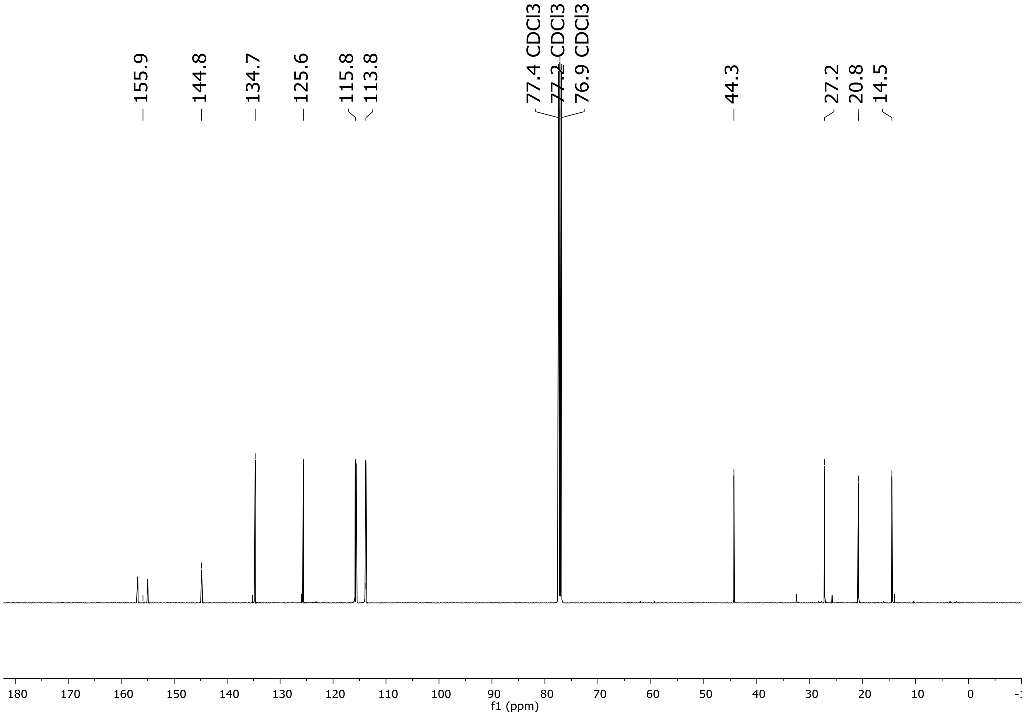

(Z)-N-benzhydryl-N-benzylhex-3-en-1-amine, 22a
^{1}H NMR (400 MHz, $CDCl_3$):

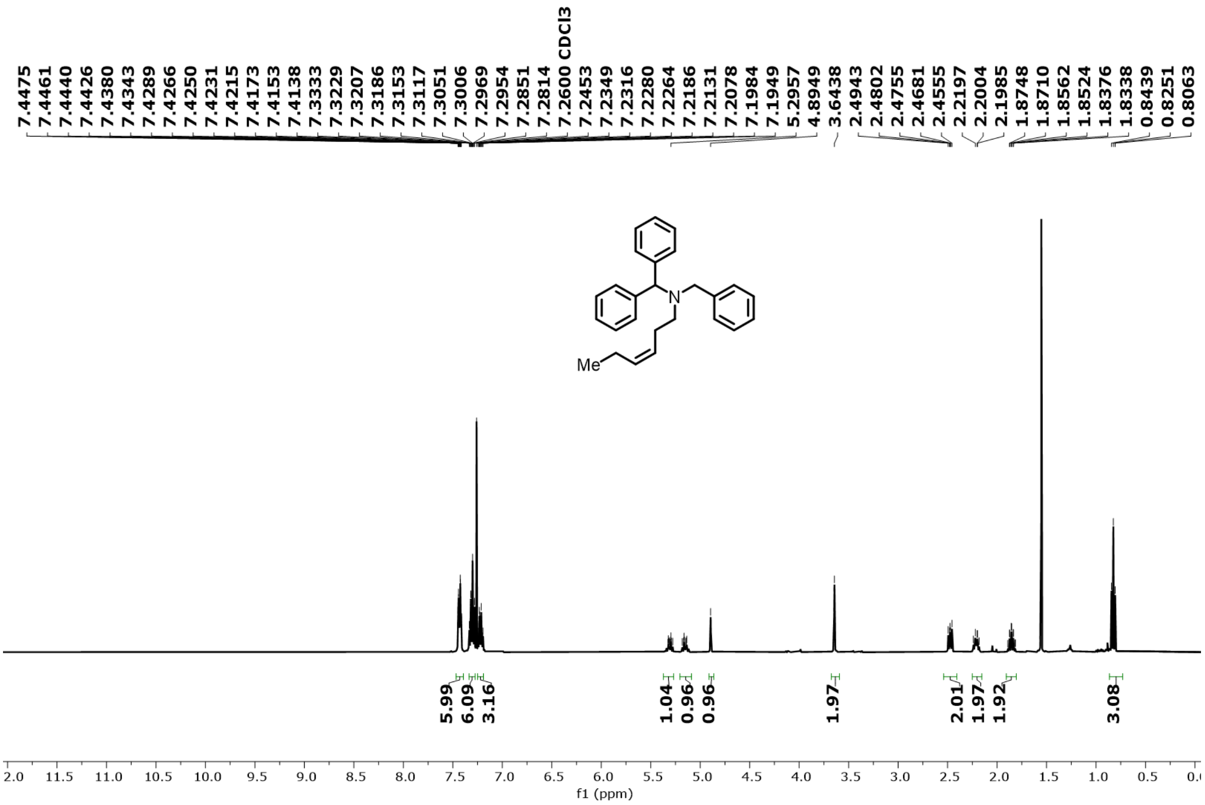

4-(Trifluoromethyl)phenyl (E)-octadec-9-enoate, 23a
^{1}H NMR (400 MHz, $CDCl_3$):

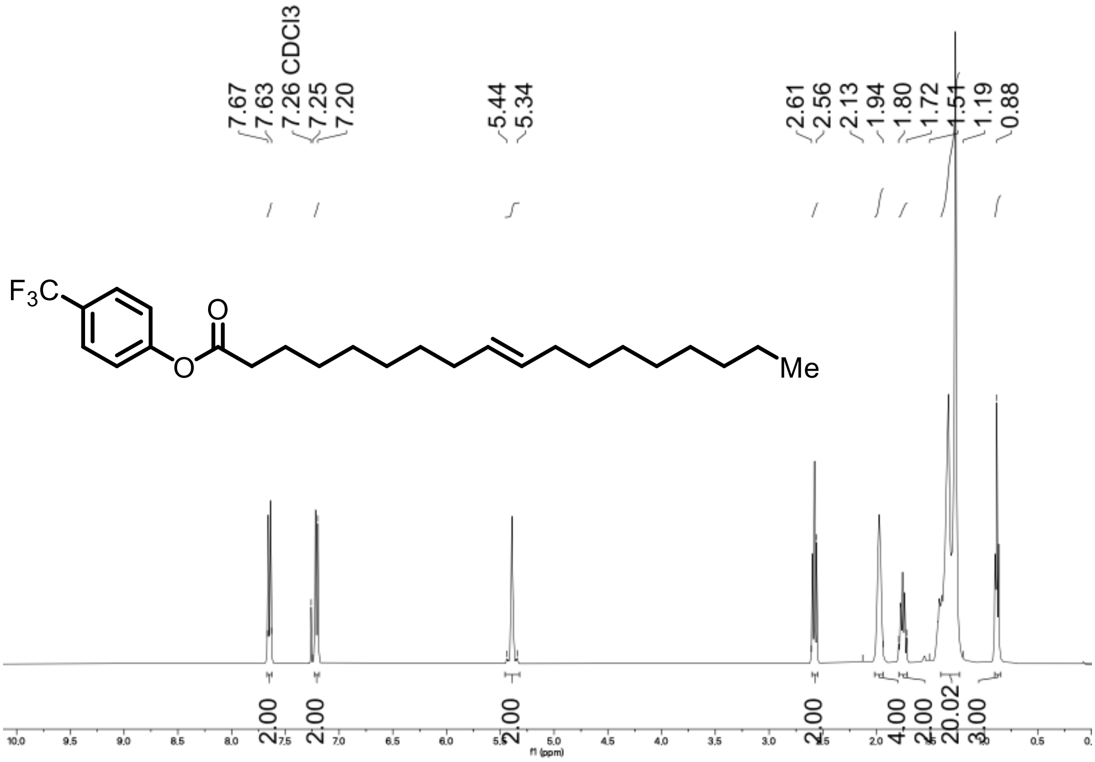

^{13}C {^{1}H} NMR (100 MHz, $CDCl_3$):

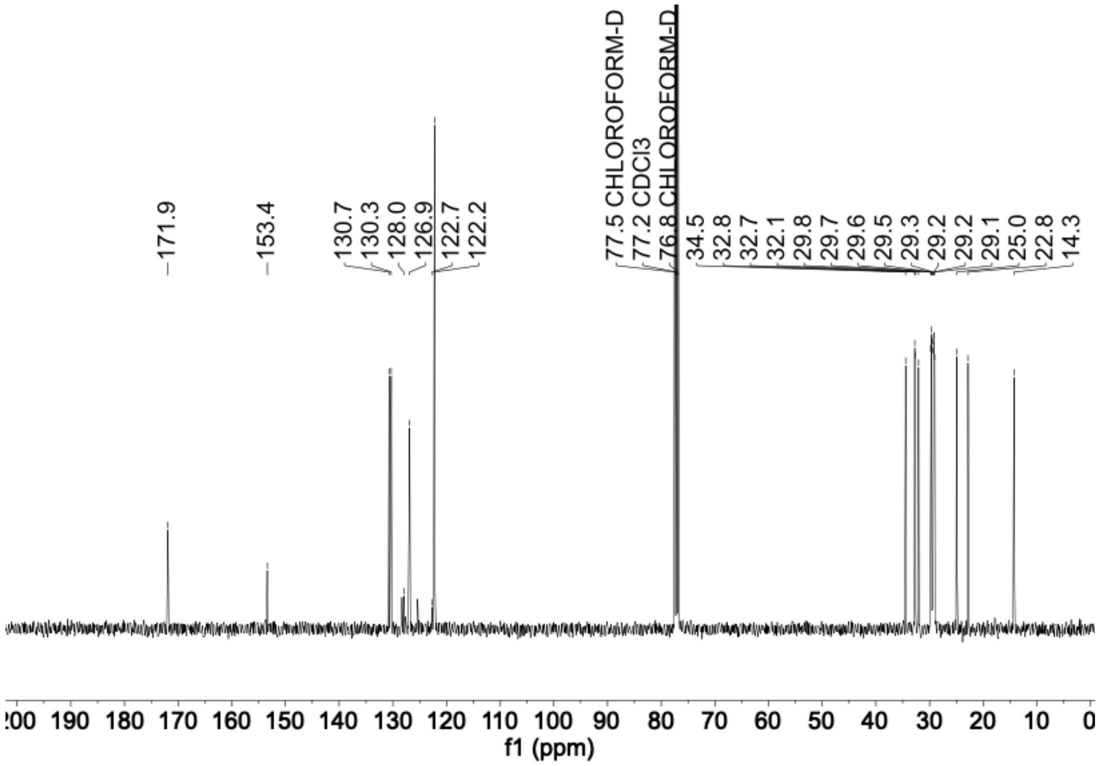

5-methyl-4-(2-methylprop-1-en-1-yl)thiazole, 24a

^{1}H NMR (500 MHz, CDCl$_3$):

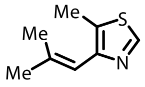

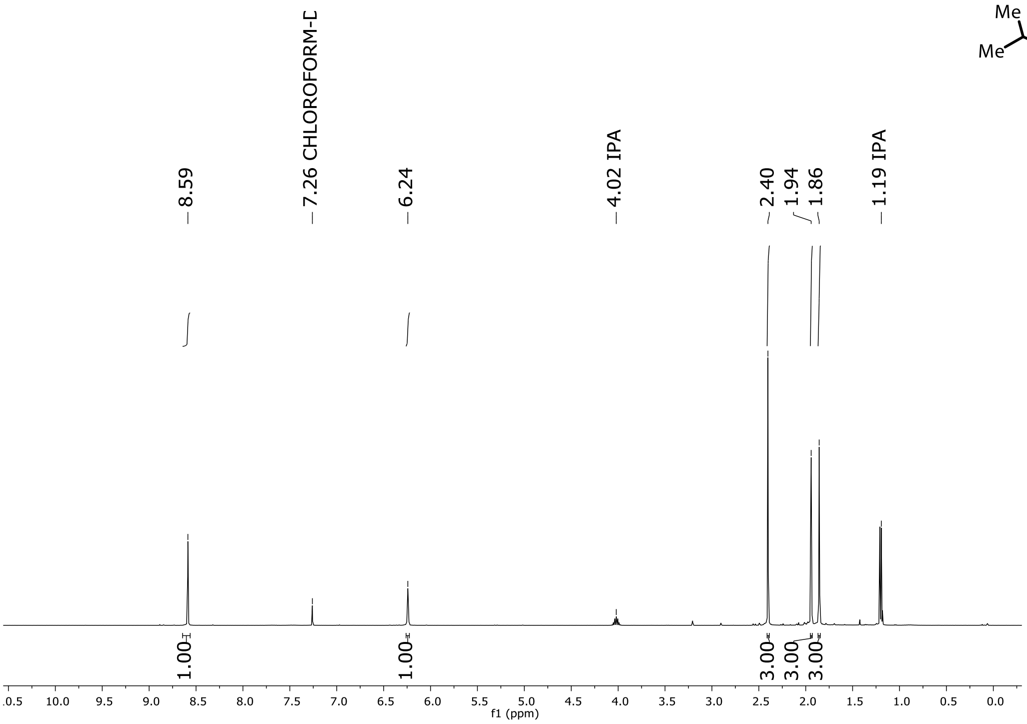

^{13}C {^{1}H} NMR (125 MHz, CDCl$_3$):

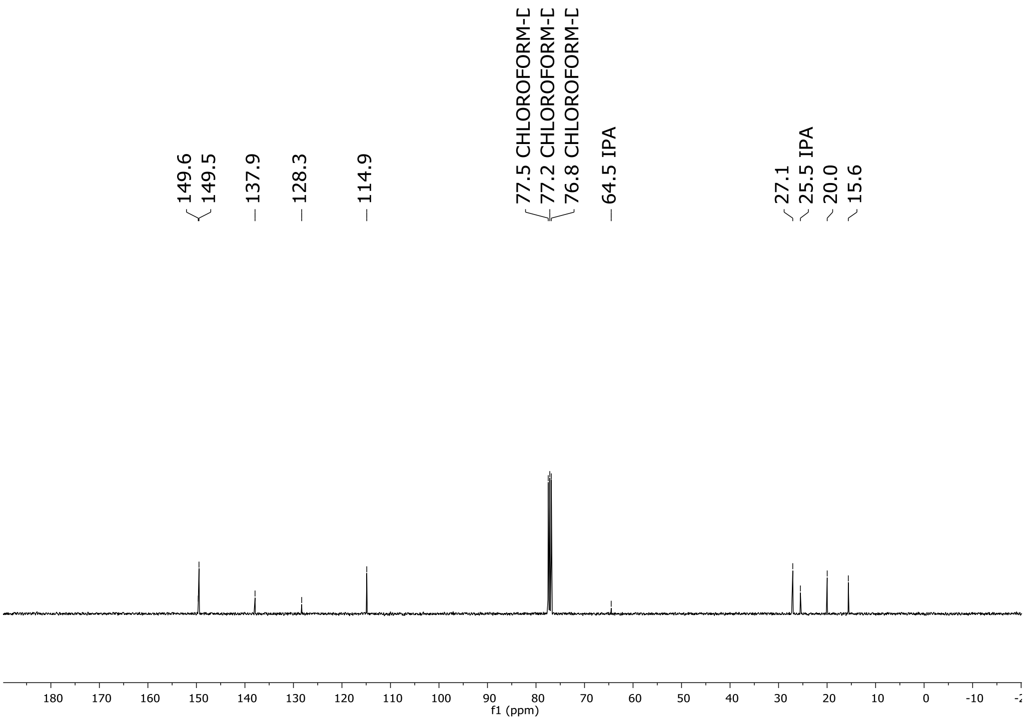

(Z)-N,N-dibenzylhex-3-en-1-amine, 26a-*cis*

^{1}H NMR (400 MHz, $CDCl_3$):

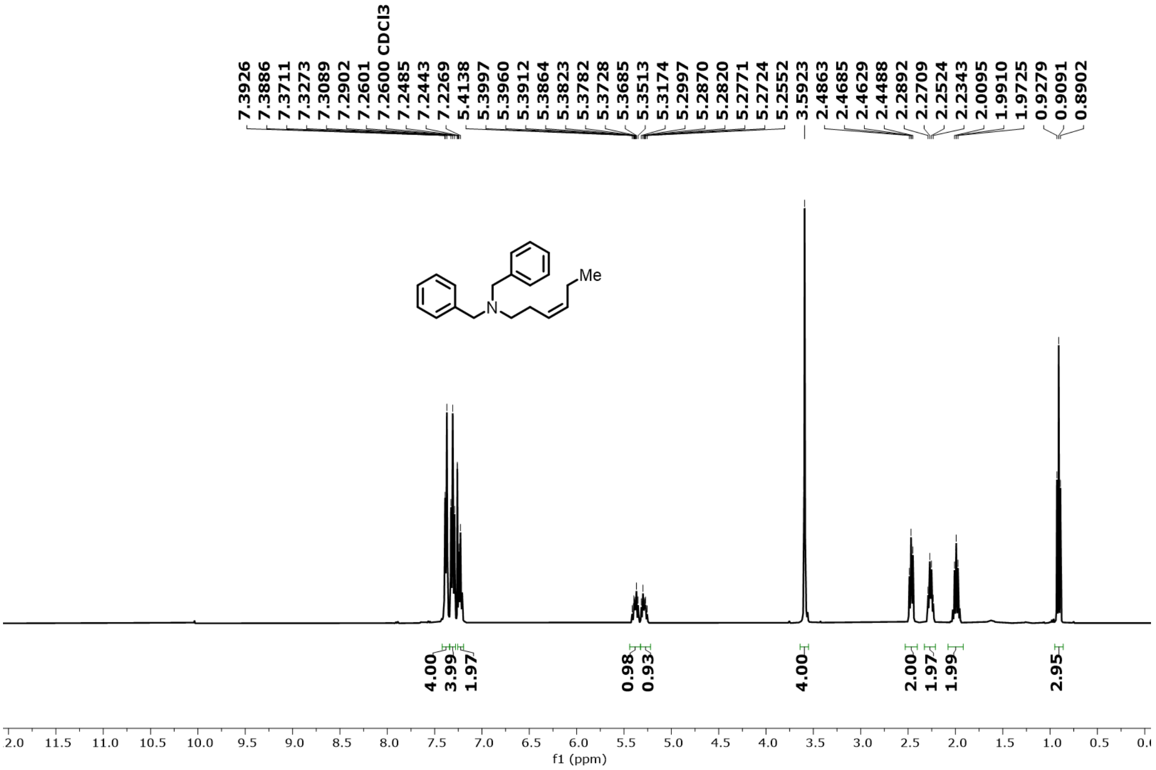

^{13}C {^{1}H} NMR (101 MHz, $CDCl_3$):

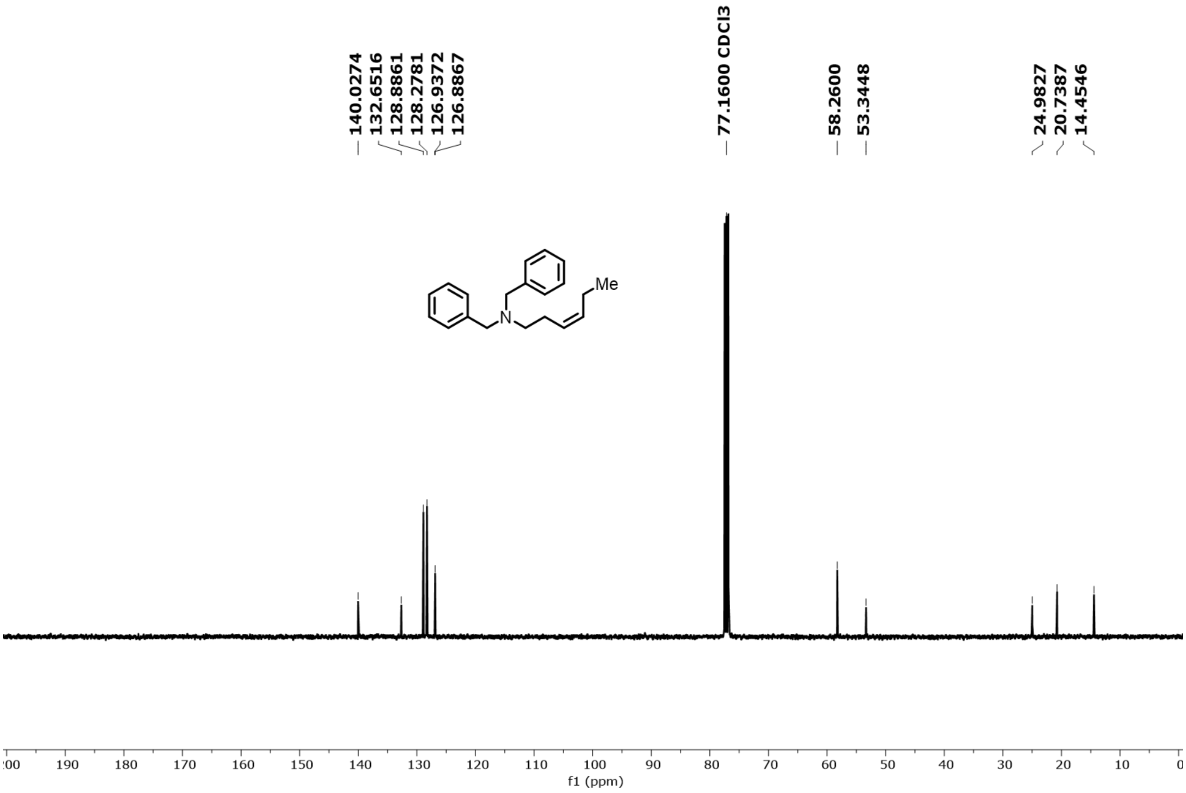

(Z)-N,N-dibenzylhex-4-en-1-amine, 27a
^{1}H NMR (400 MHz, $CDCl_3$):

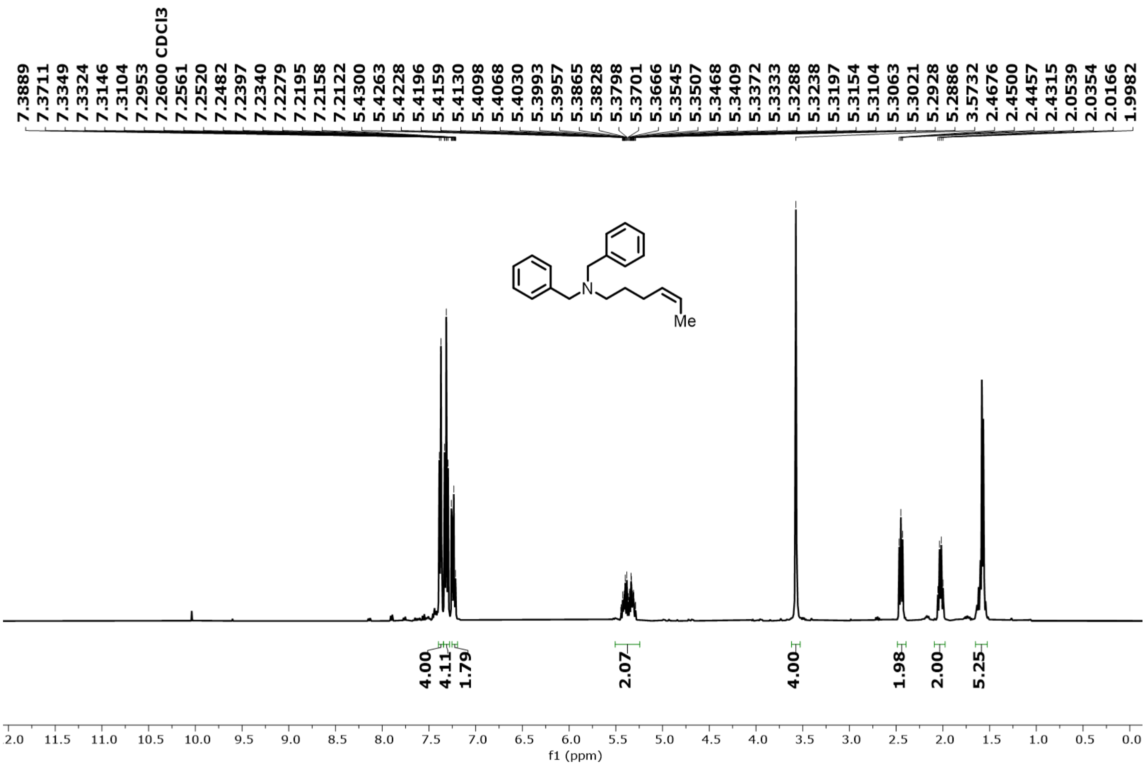

^{13}C {^{1}H} NMR (101 MHz, CDCl3):

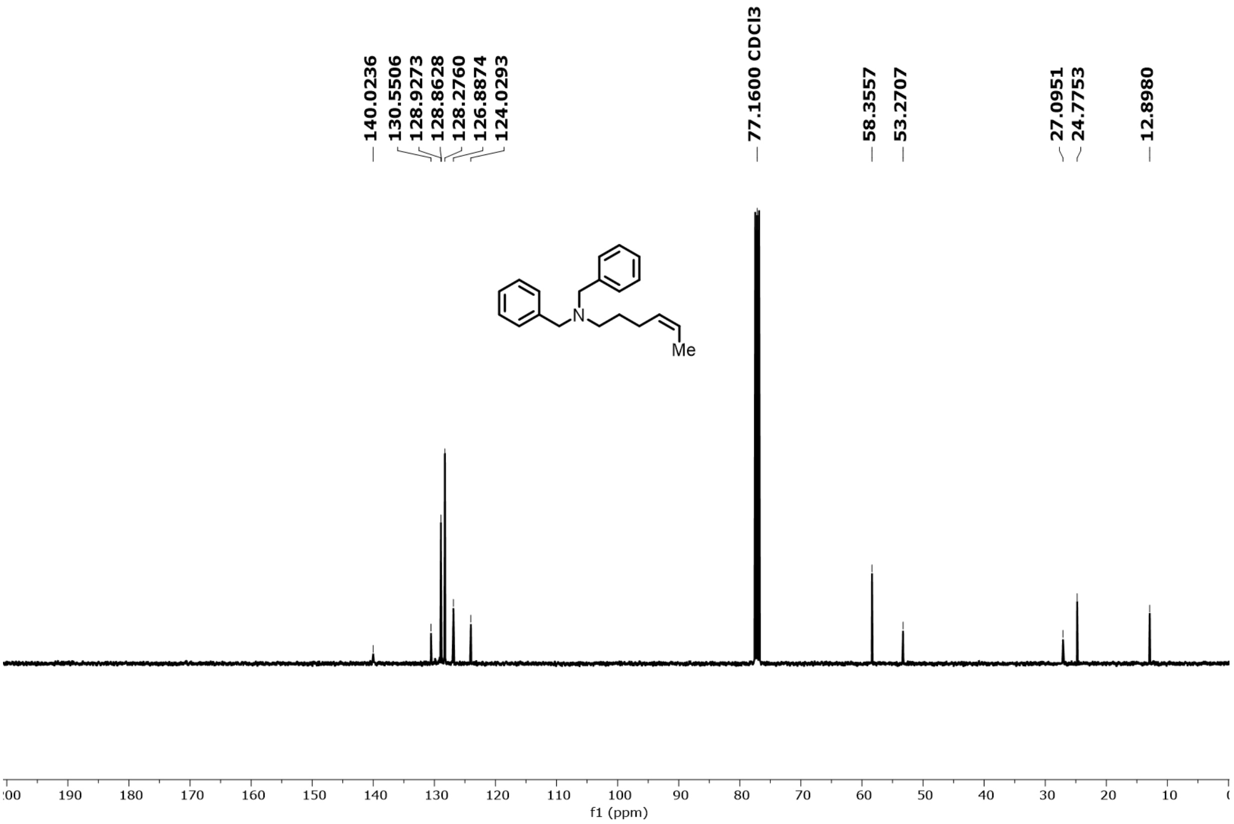

(*Z*)-tert-butyl(hex-3-en-1-yloxy)diphenylsilane, 29a

^{1}H NMR (400 MHz, CDCl$_3$):

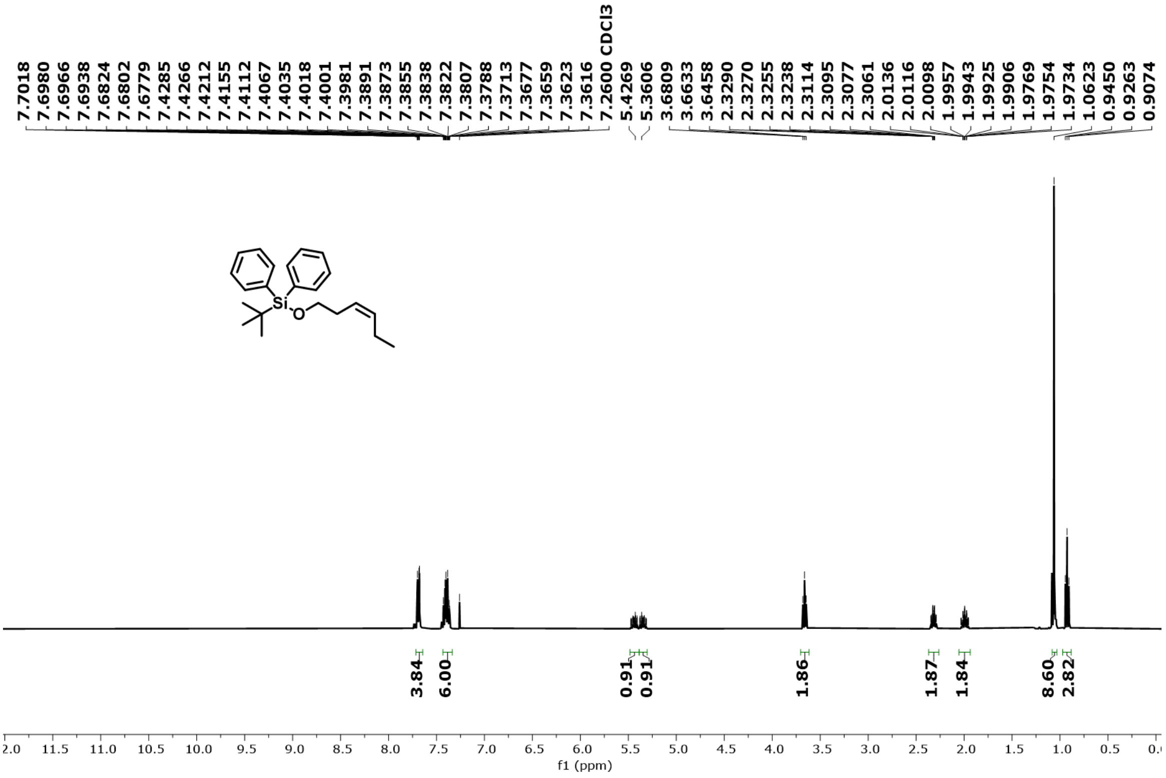

^{13}C {^{1}H} NMR (101 MHz, CDCl3):

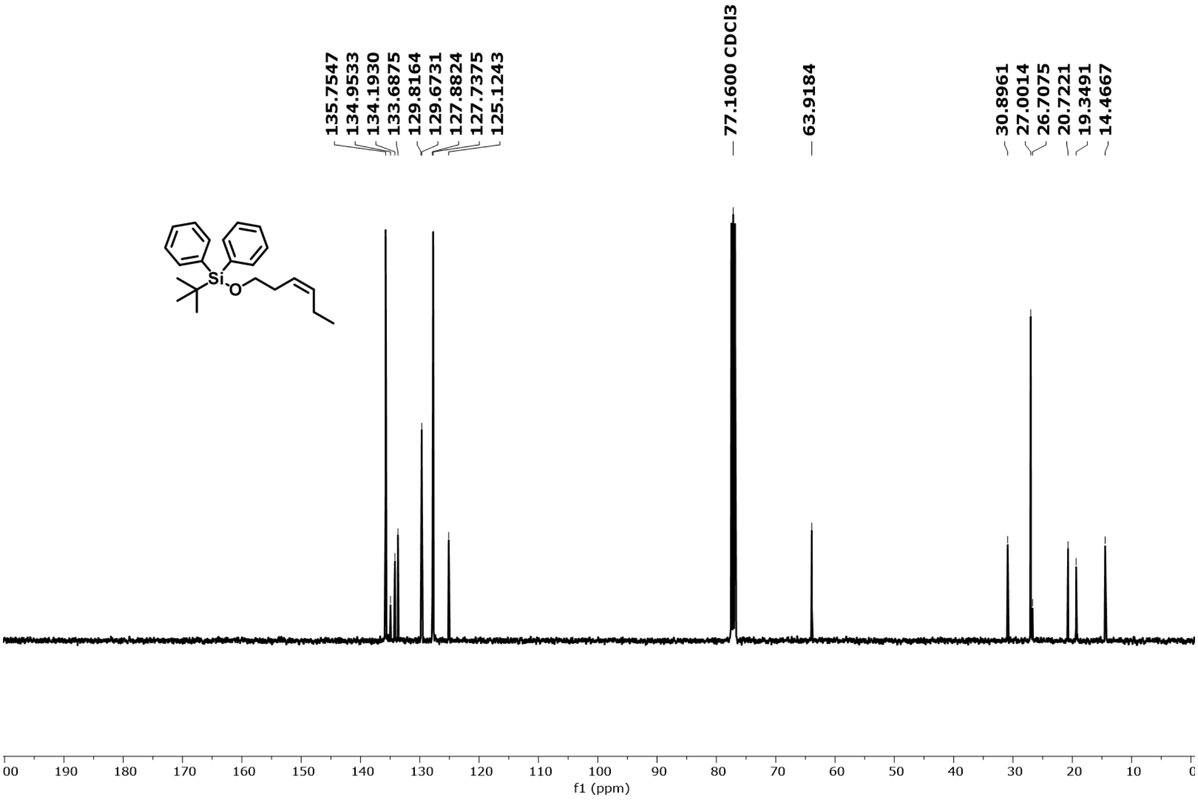

^{29}Si (79.5 Hz, $CDCl_3$):

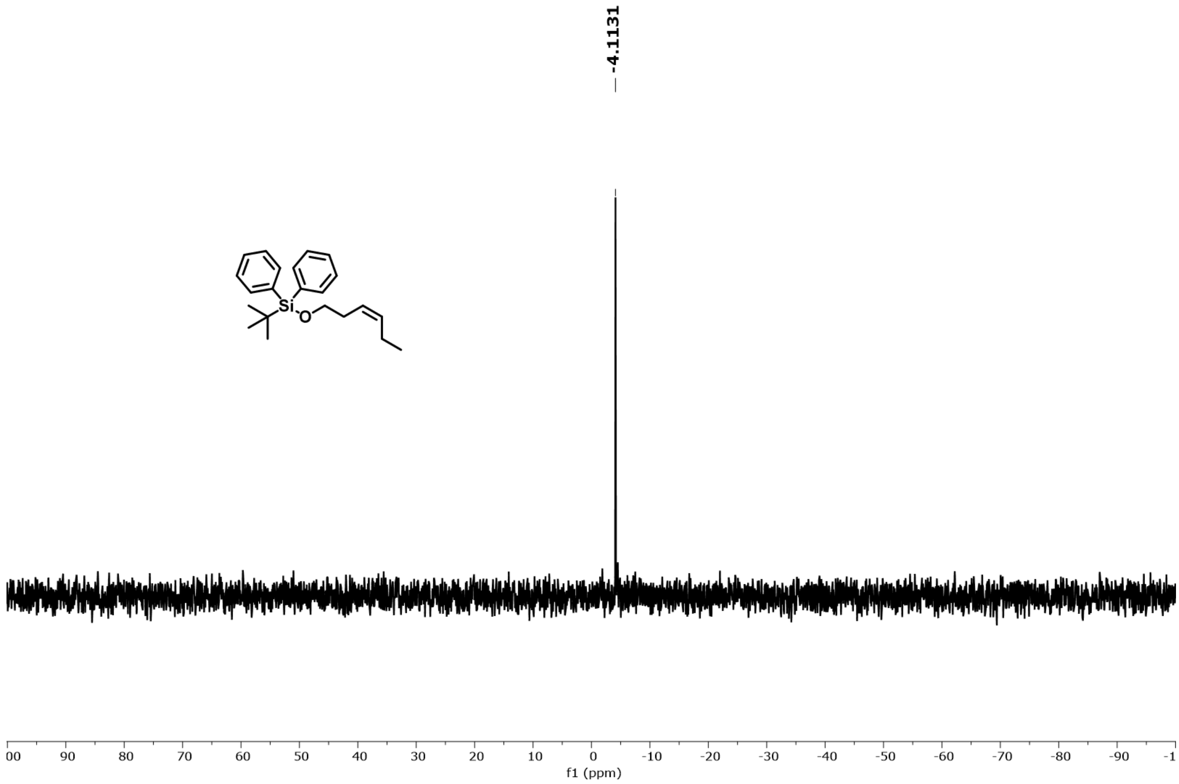

(*E*)-*N*,*N*-dibenzylbut-2-enamide, 31a
^{1}H NMR (400 MHz, $CDCl_3$):

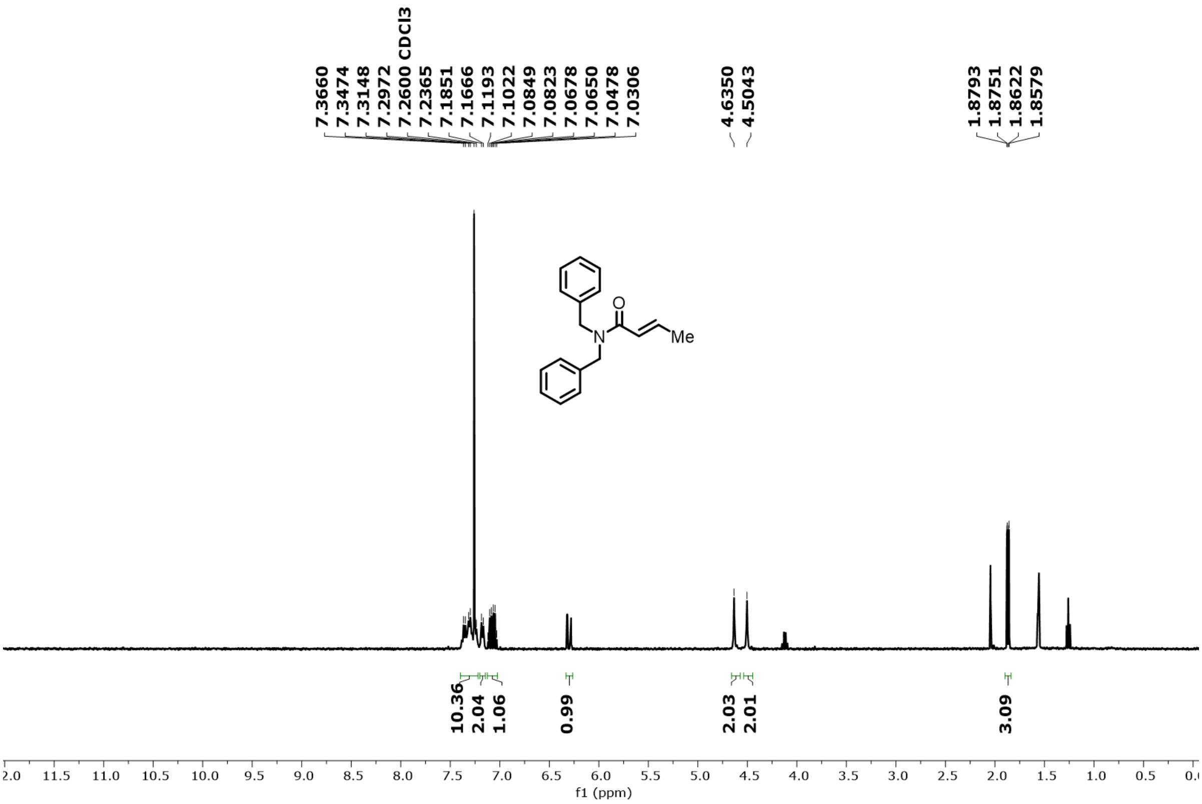

1-tosyl-2,5-dihydro-1H-pyrrole, 32a

^{1}H NMR (400 MHz, $CDCl_3$):

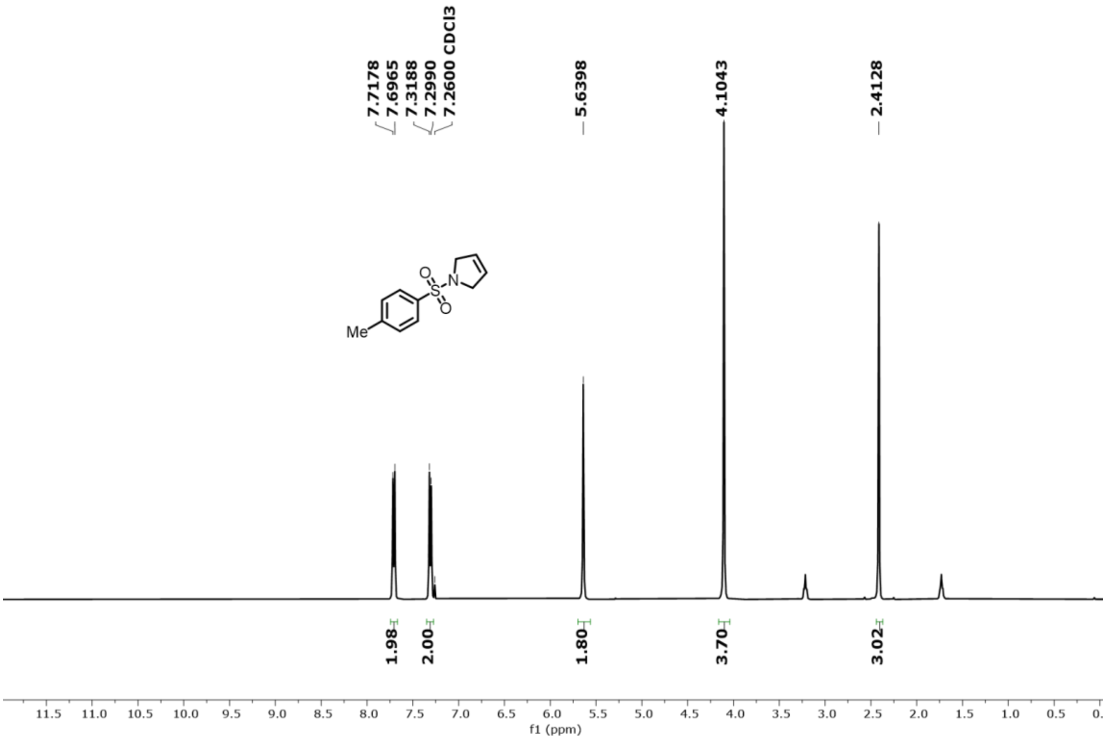

^{13}C {^{1}H} NMR (101 MHz, $CDCl_3$):

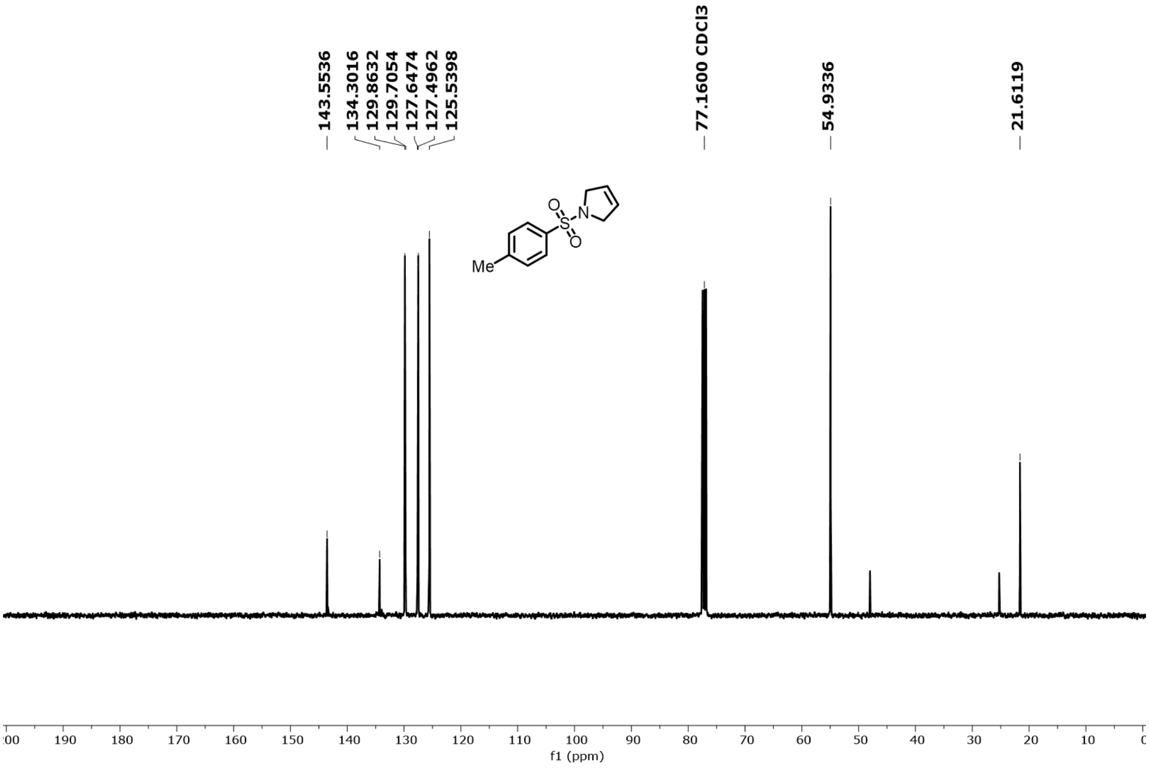

tert-Butyl-(Z)-9-(hex-3-en-1-yl)-3,9-diazaspiro[5.5]undecane-3-carboxylate, 34a

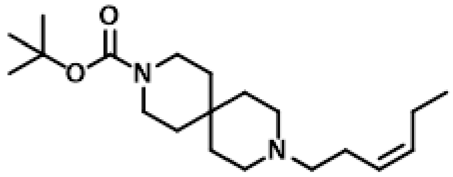

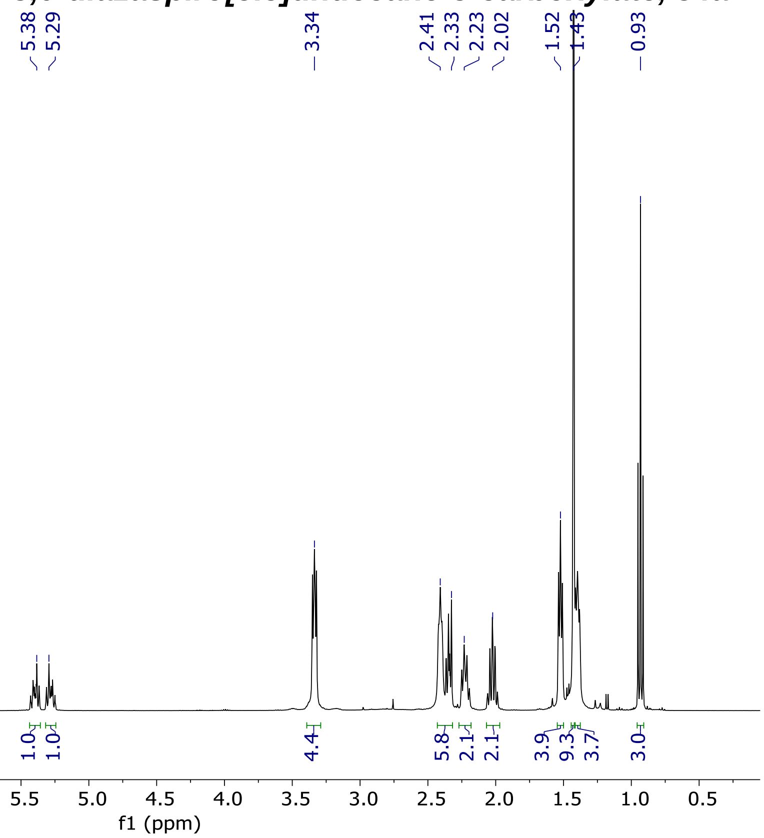

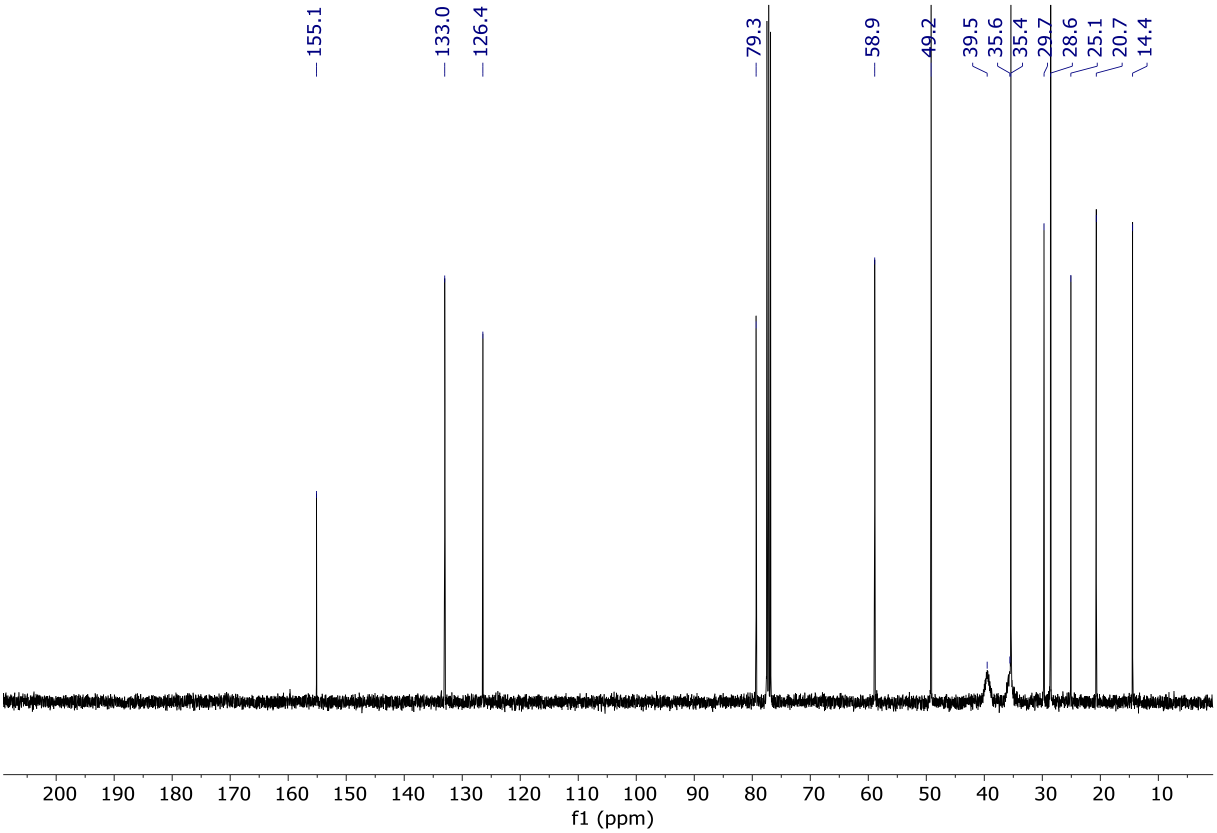

(*E*)-hex-3-en-1-yl 3,5-dinitrobenzoate, 36a
^{1}H NMR (400 MHz, $CDCl_3$):

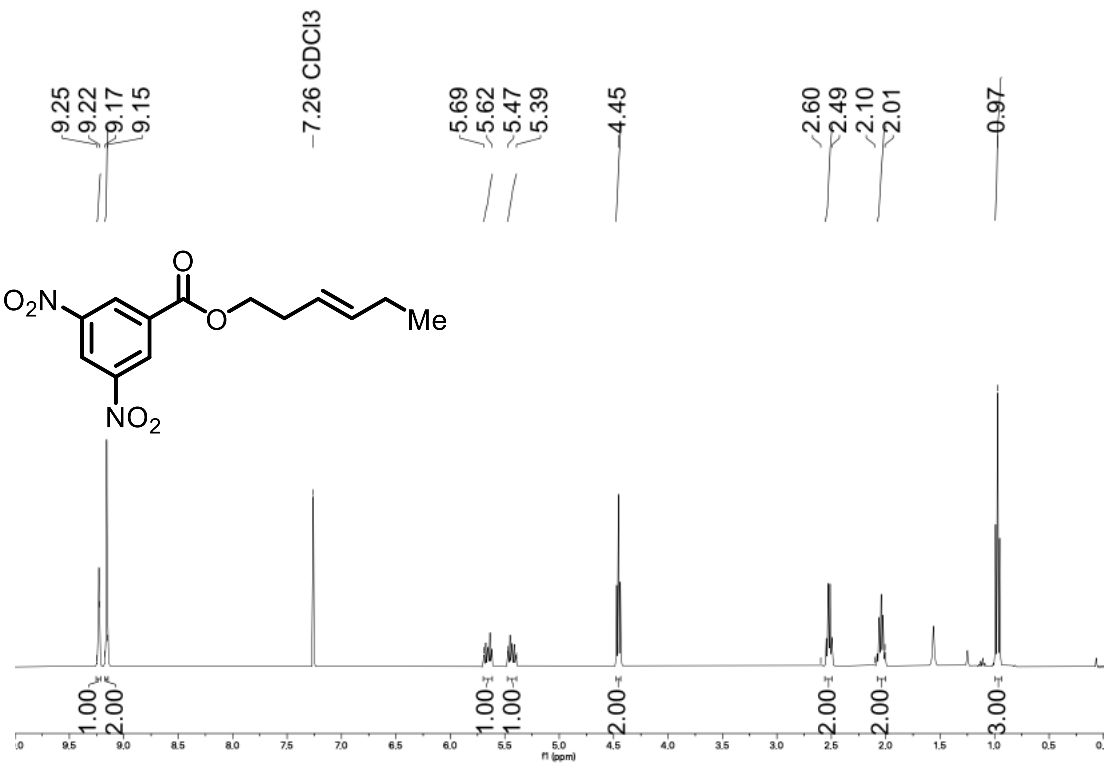

^{13}C {^{1}H} NMR (100 MHz, $CDCl_3$):

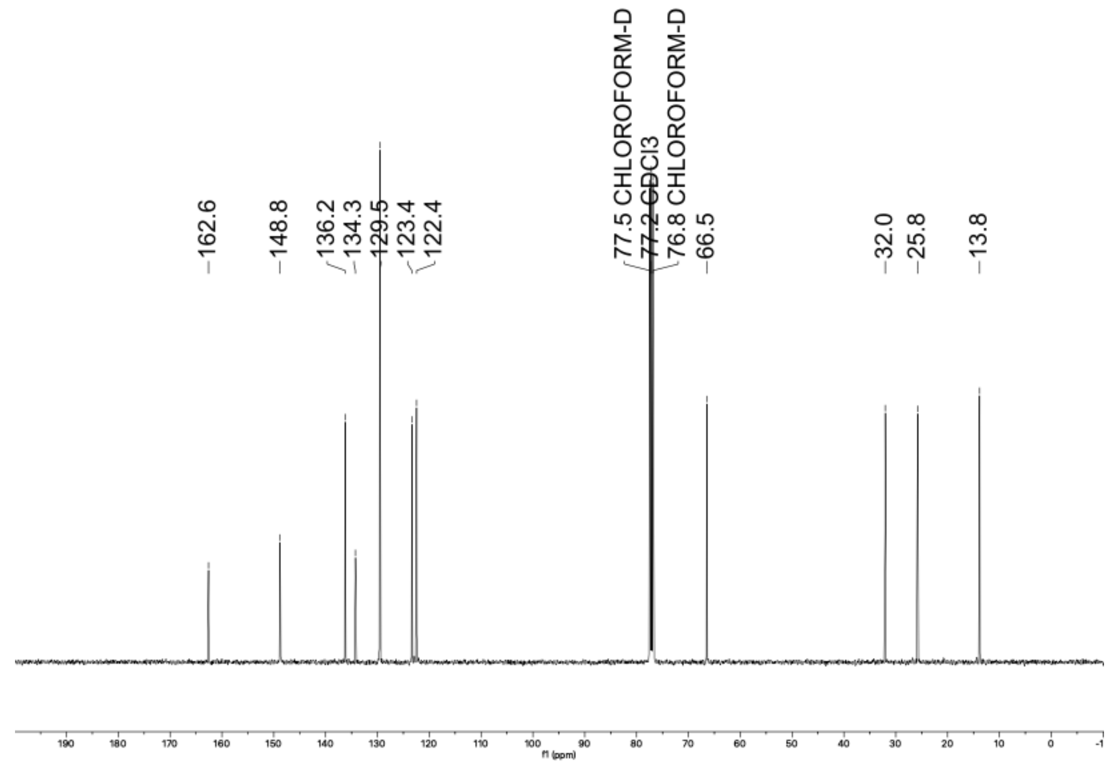

(*Z*)-2-(1-(Hex-3-en-1-yl)piperidin-4-yl)-4,6-dimethoxypyrimidine, 37a

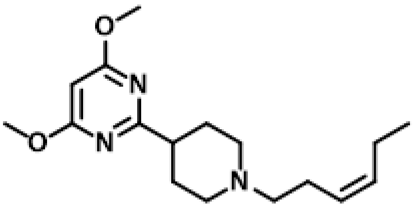

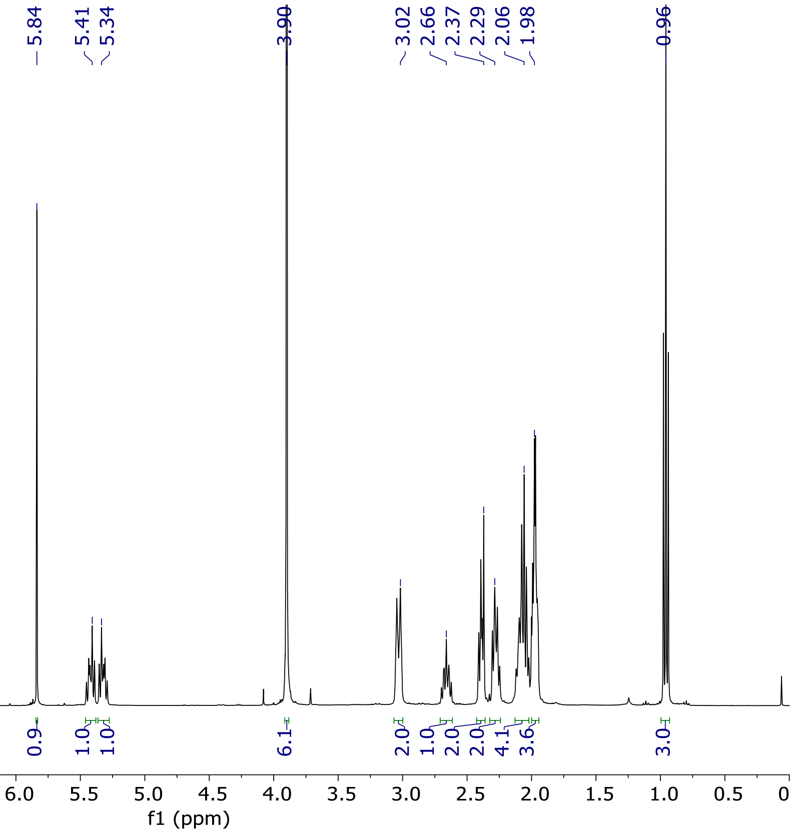

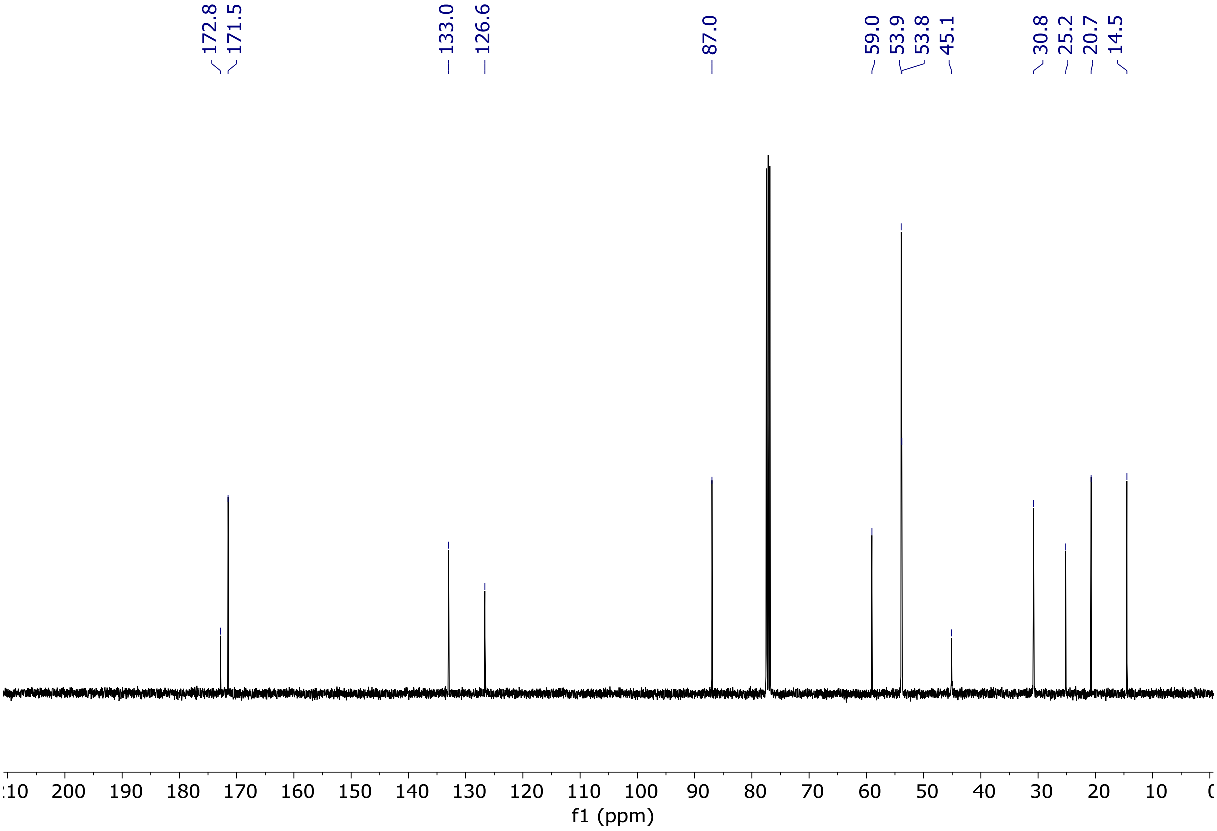

(Z)-2-(hex-3-en-1-yl)isoindoline, 40a-cis

^{1}H NMR (500 MHz, $CDCl_3$):

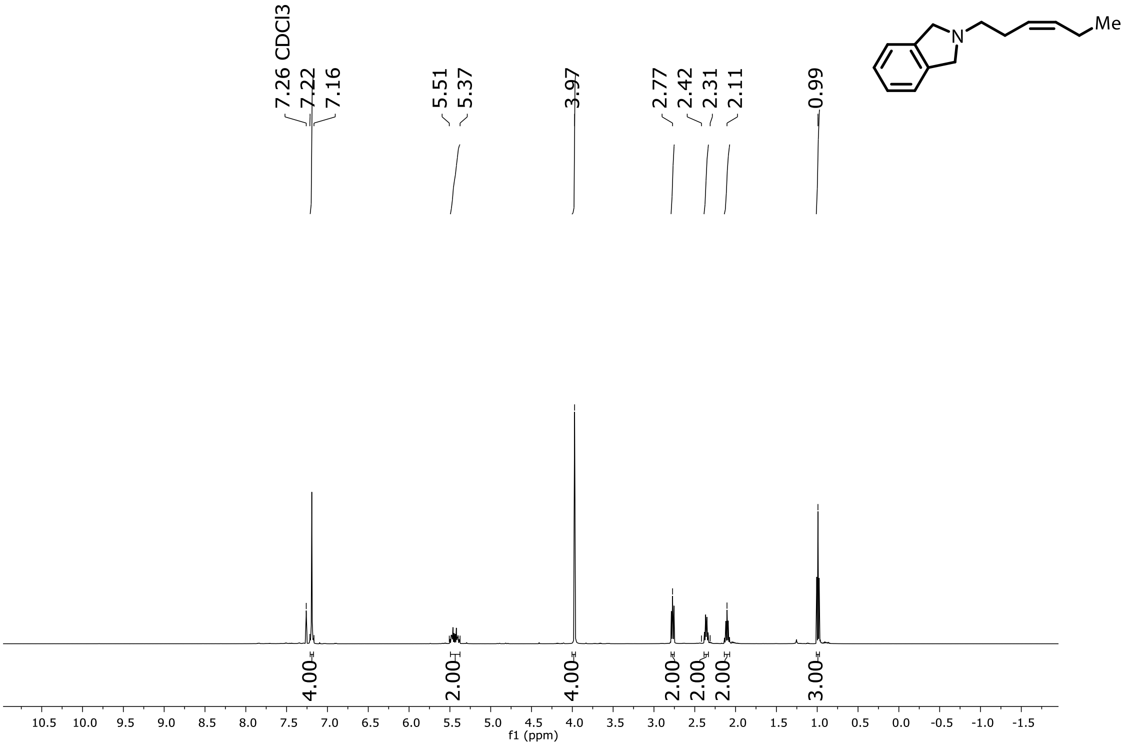

^{13}C {^{1}H} NMR (125 MHz, $CDCl_3$):

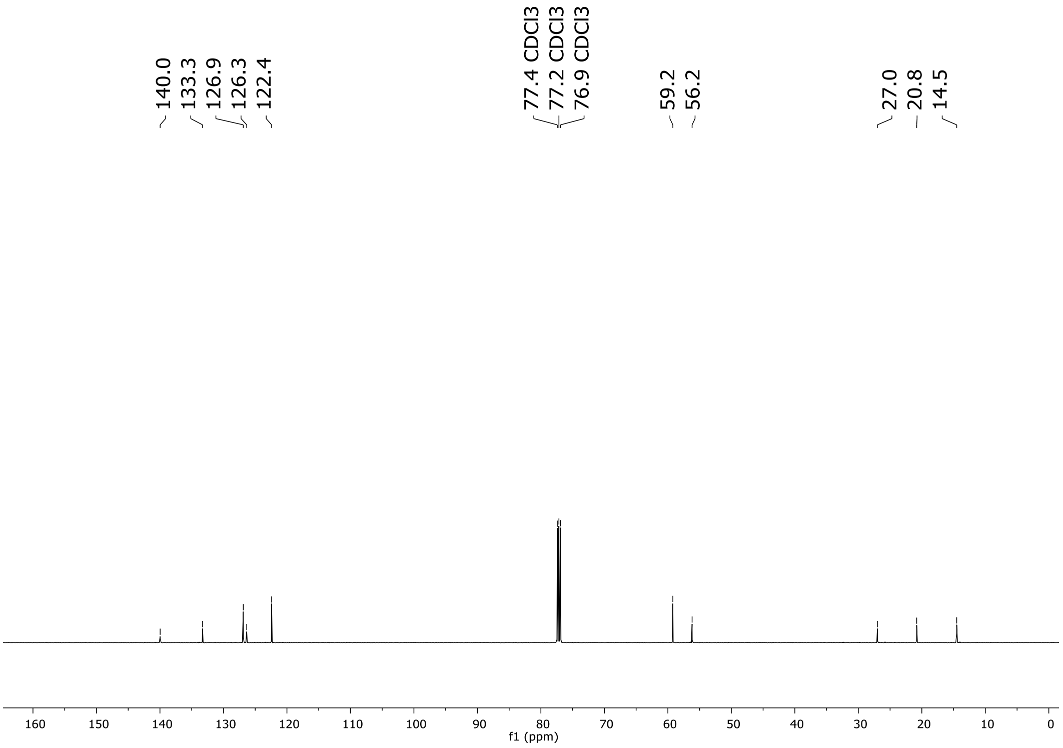

(Z)-6-fluoro-3-(1-(hex-3-en-1-yl)piperidin-4-yl)benzo[d]isoxazole, 41a

^{1}H NMR (500 MHz, CDCl$_3$):

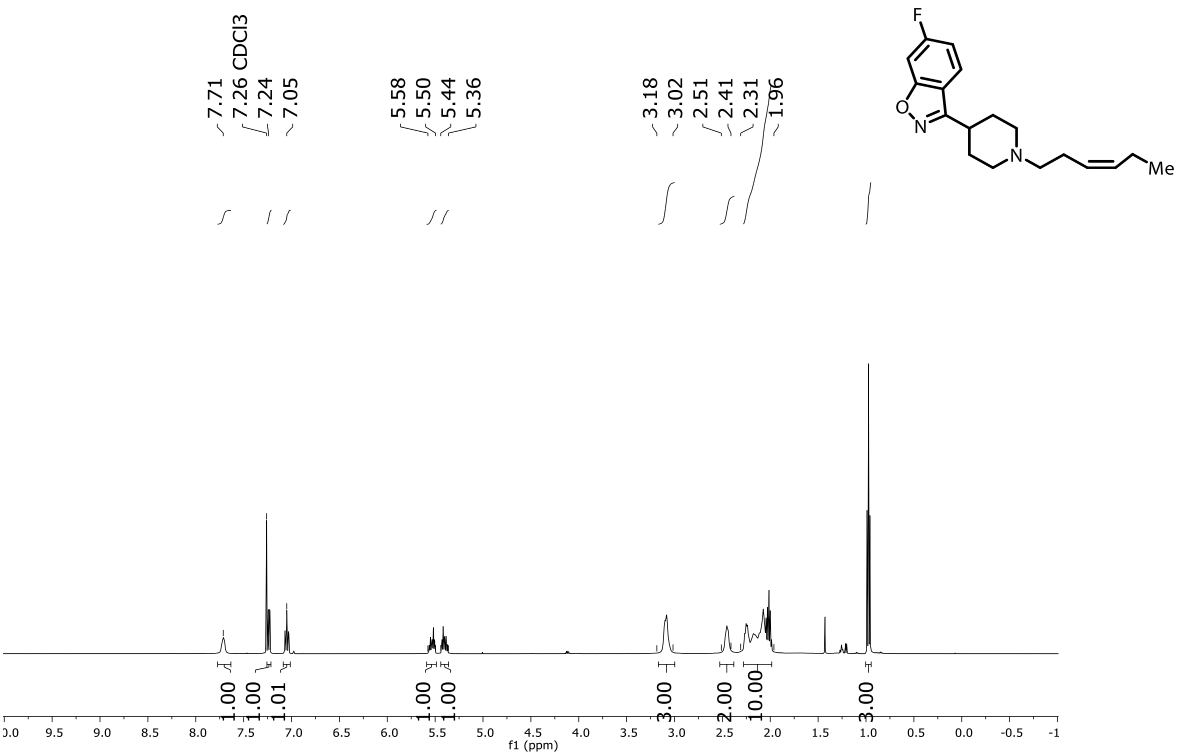

^{19}F NMR (376 MHz, CDCl$_3$):

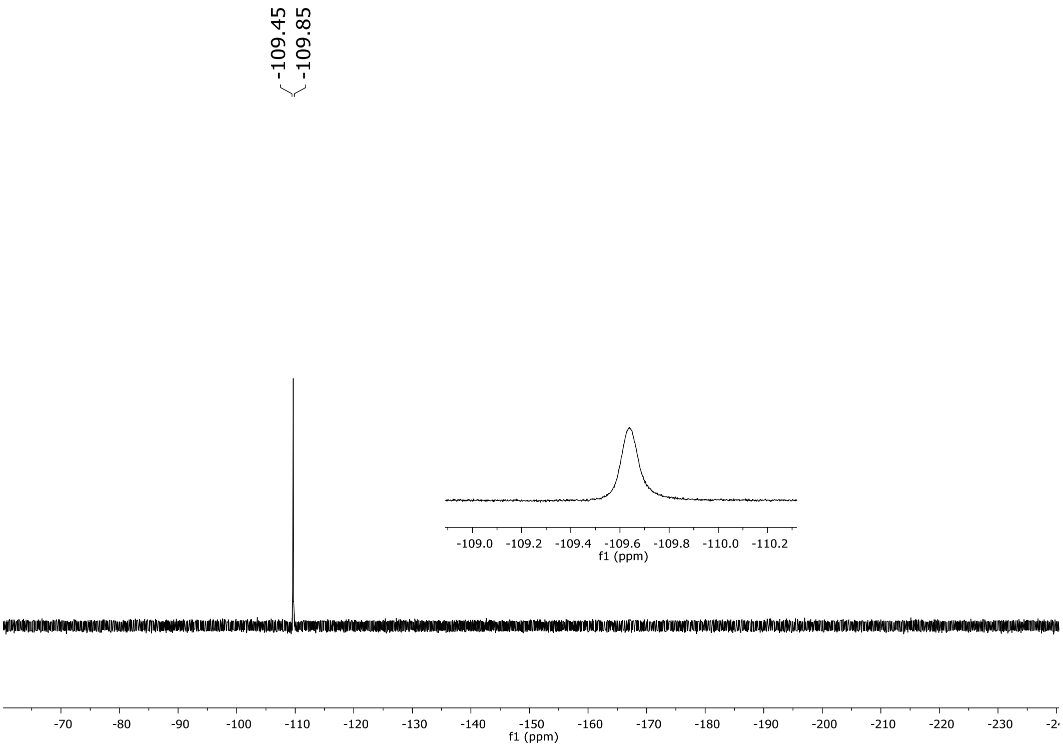

(Z)-4-(hex-3-en-1-yl)morpholine, 42a

^{1}H NMR (500 MHz, CDCl$_3$):

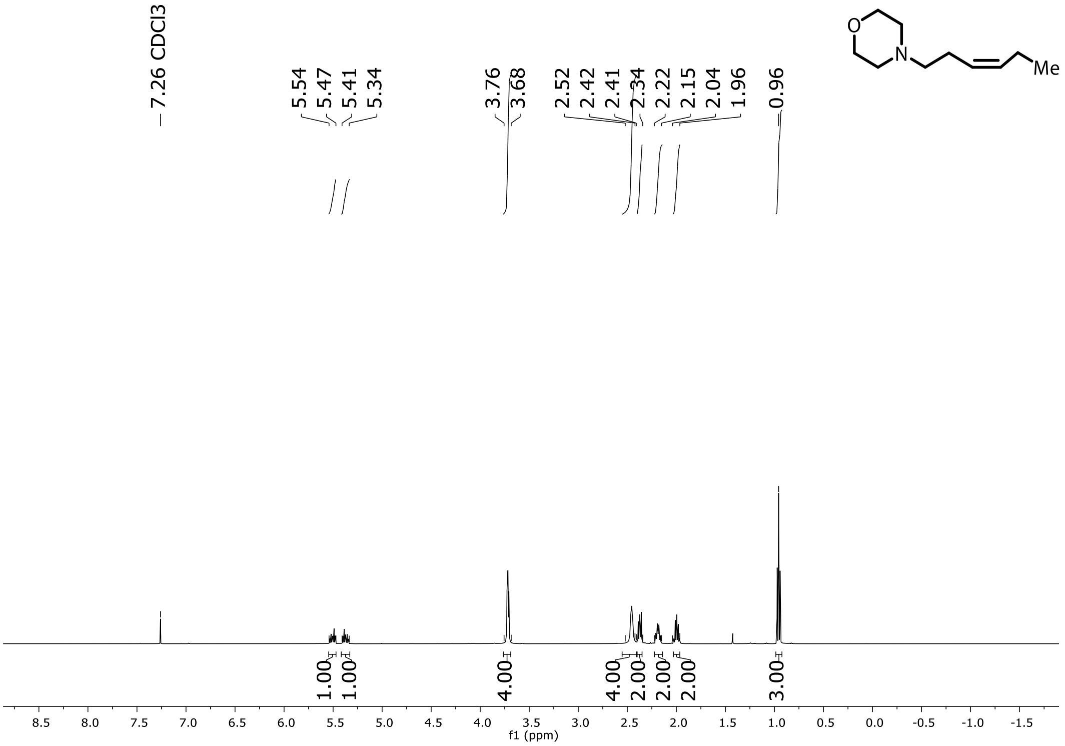

^{13}C {^{1}H} NMR (125 MHz, CDCl$_3$):

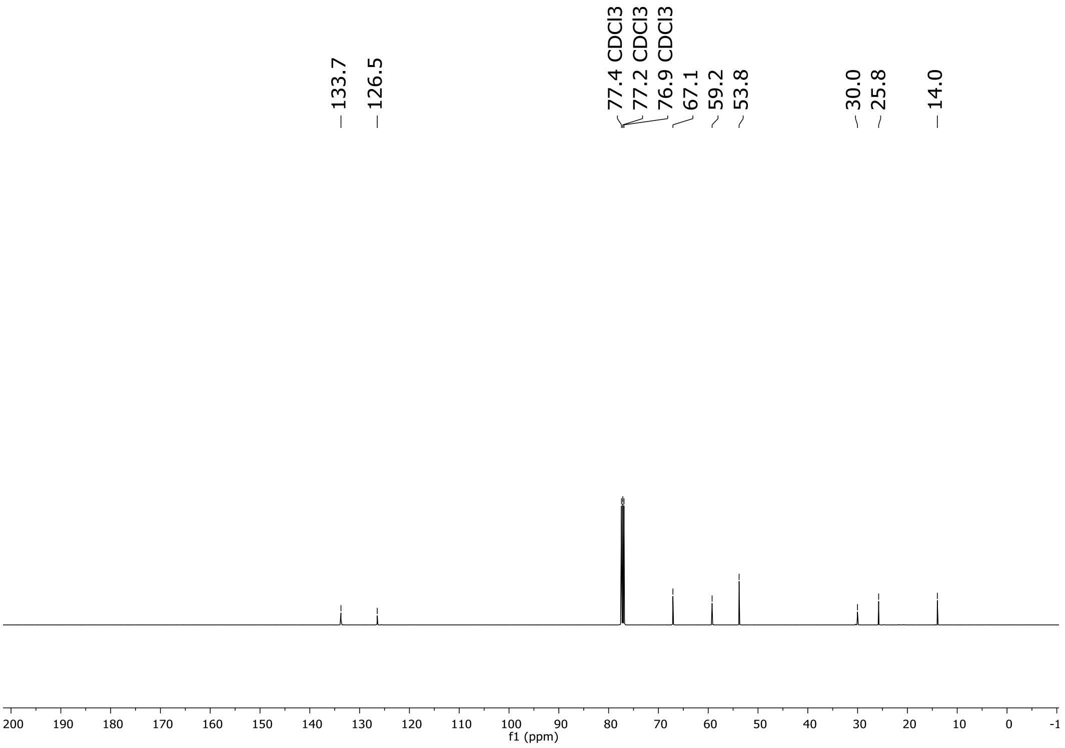

(Z)-1-(hex-3-en-1-yl)-4-(4-nitrophenyl)piperazine, 43a

^{1}H NMR (500 MHz, CDCl$_3$):

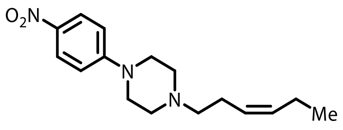

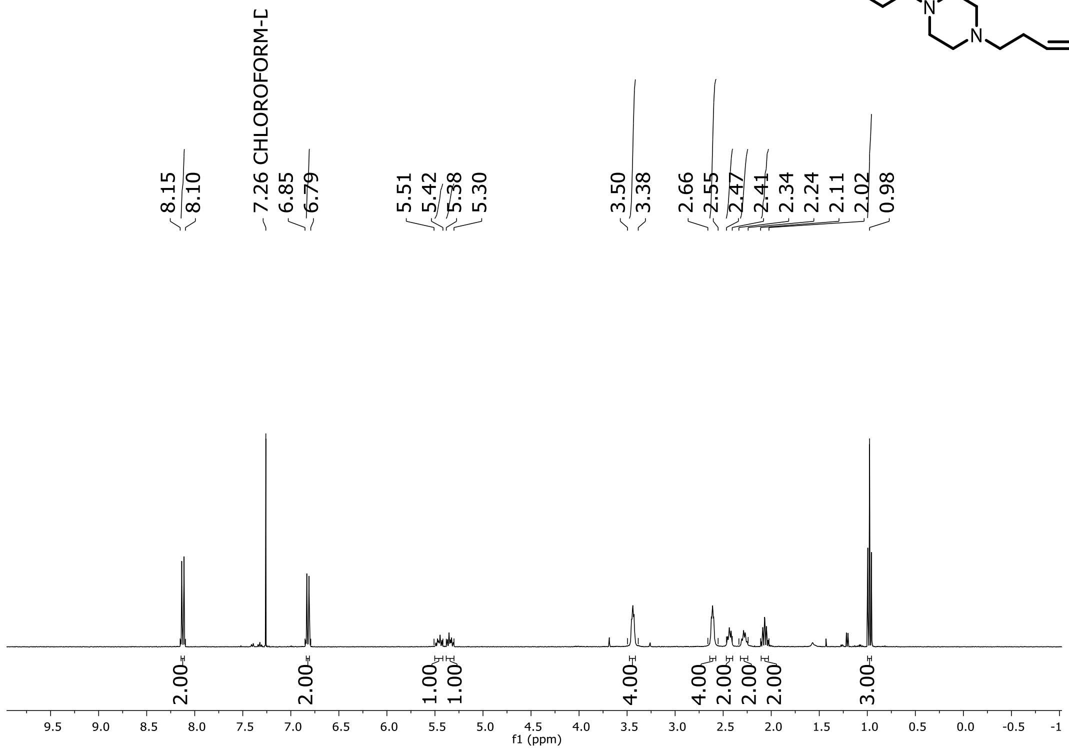

^{13}C {^{1}H} NMR (125 MHz, CDCl$_3$):

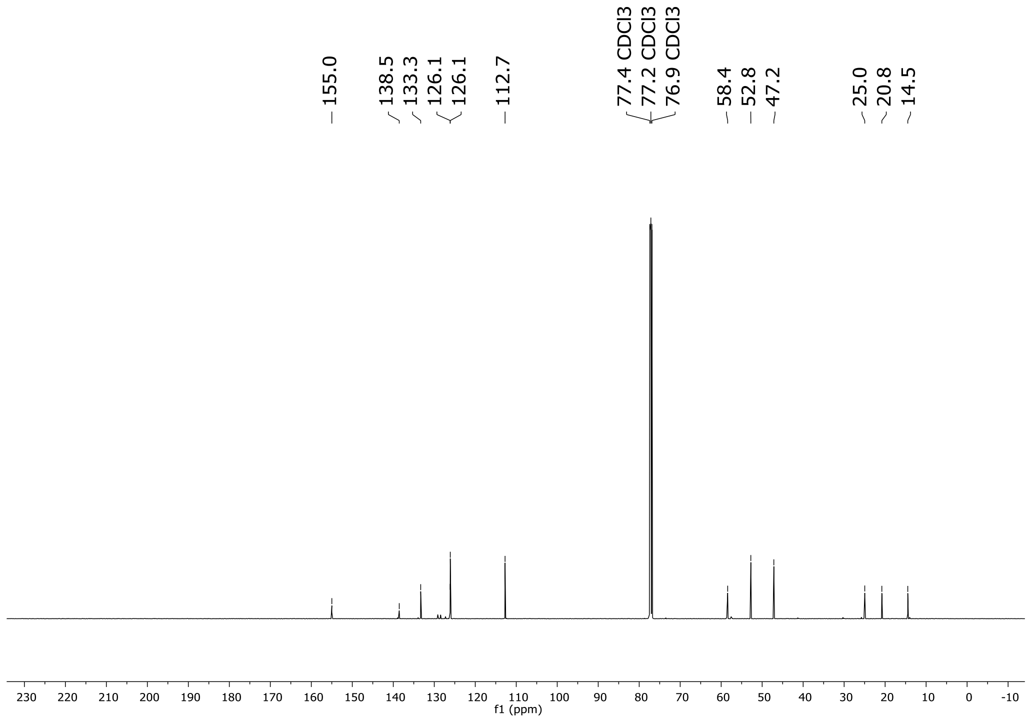

Back to top

(1R,5S)-8-((E)-hex-3-en-1-yl)-8-azabicyclo[3.2.1]octan-3-one, 44a

^{1}H NMR (500 MHz, CDCl$_3$):

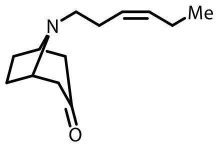

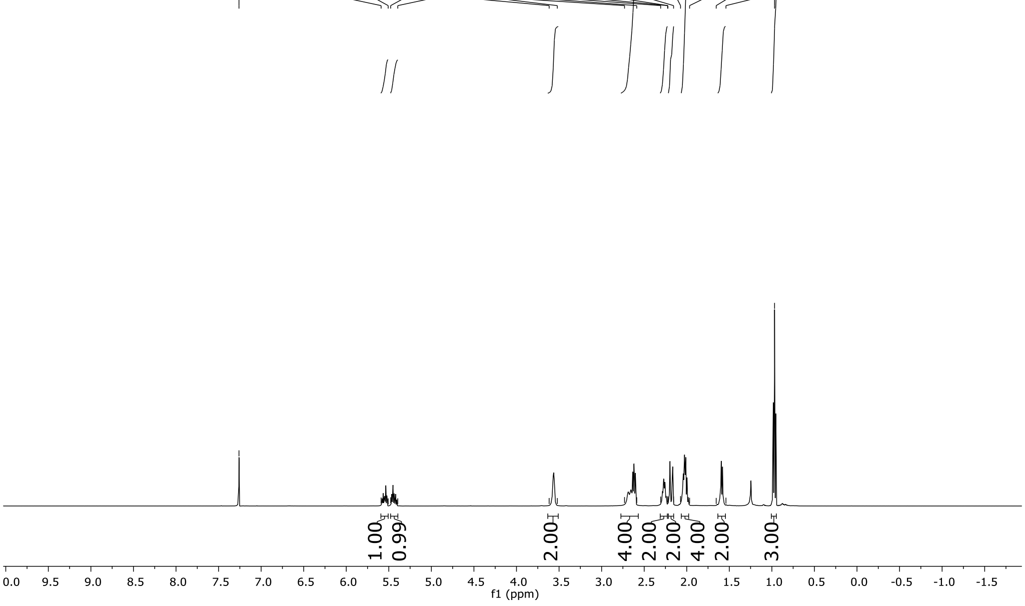

^{13}C {^{1}H} NMR (125 MHz, CDCl$_3$):

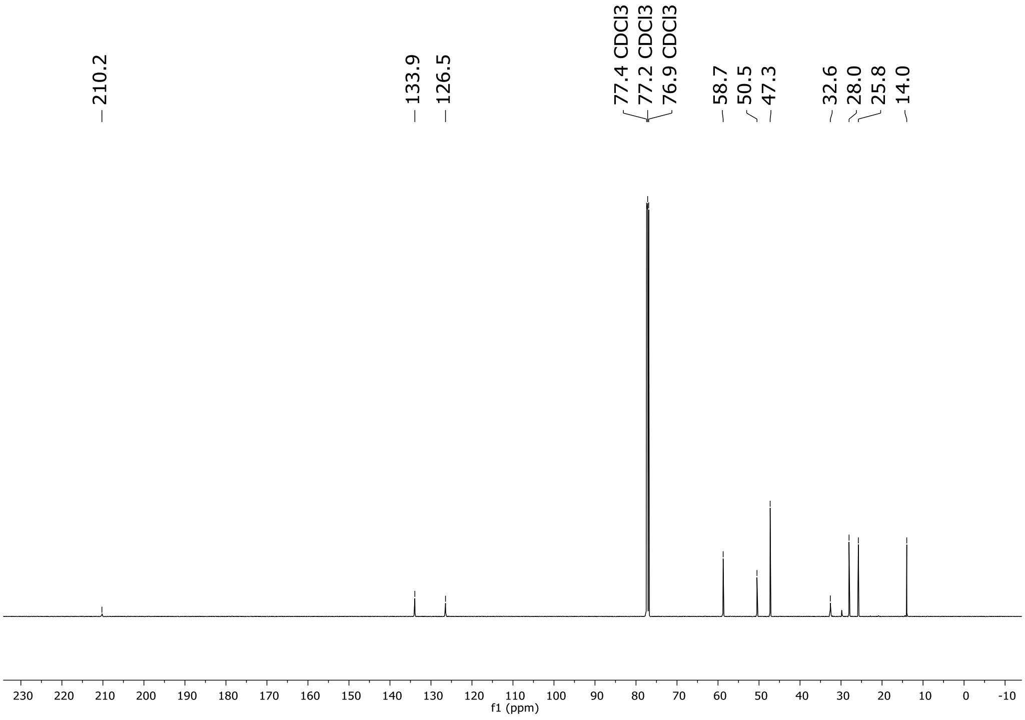

Back to top

(E)-N,N-dibenzyloct-3-en-1-amine, 45a
^{1}H NMR (500 MHz, $CDCl_3$):

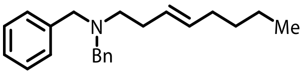

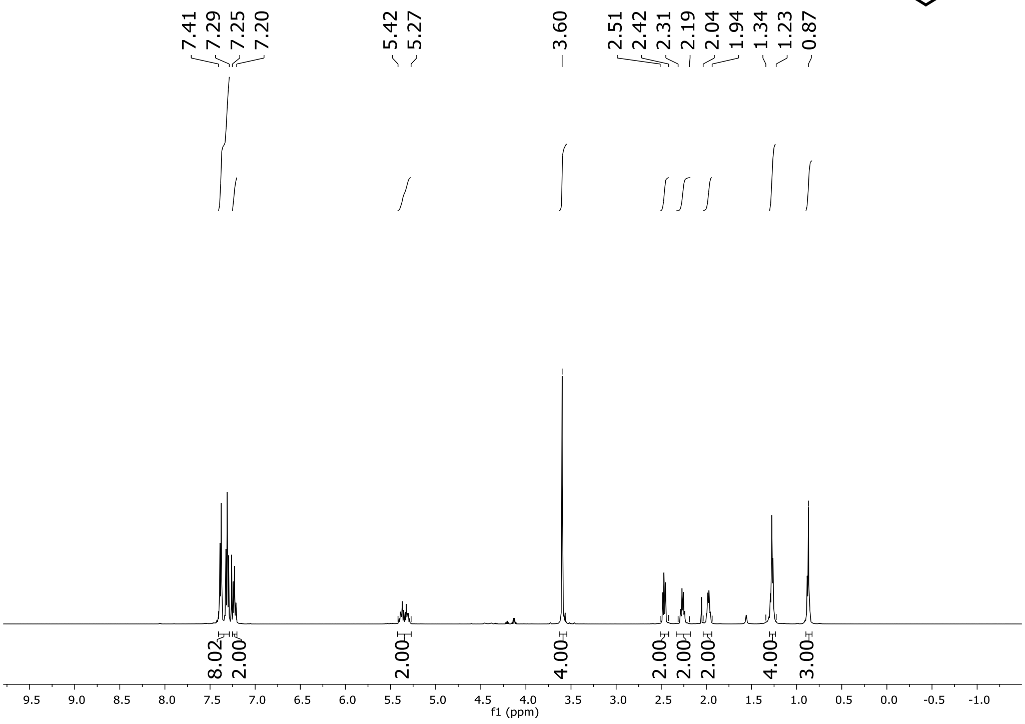

^{13}C {^{1}H} NMR (125 MHz, $CDCl_3$):

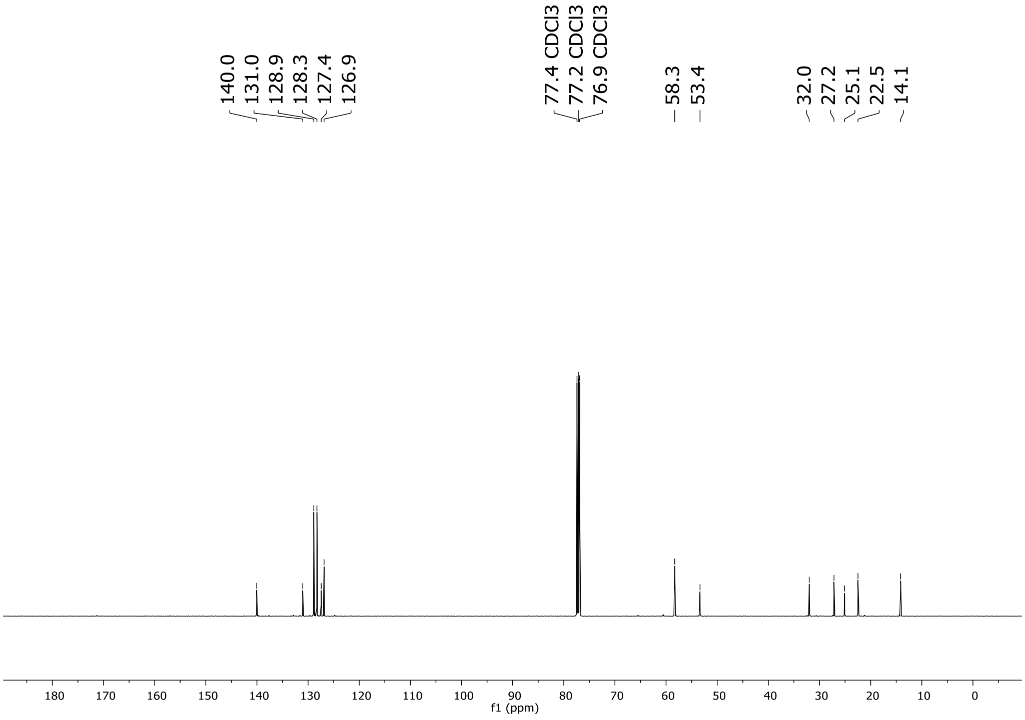

(E)-N,N-dibenzylnon-3-en-1-amine, 46a

^{1}H NMR (500 MHz, CDCl$_3$):

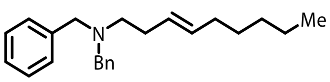

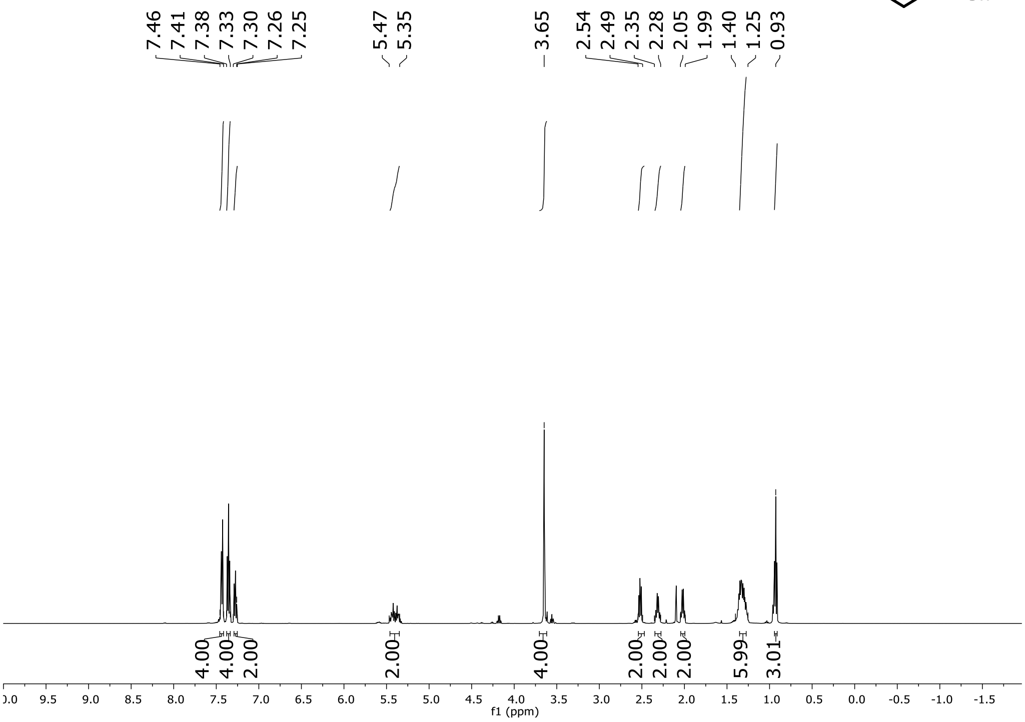

^{13}C {^{1}H} NMR (125 MHz, CDCl$_3$):

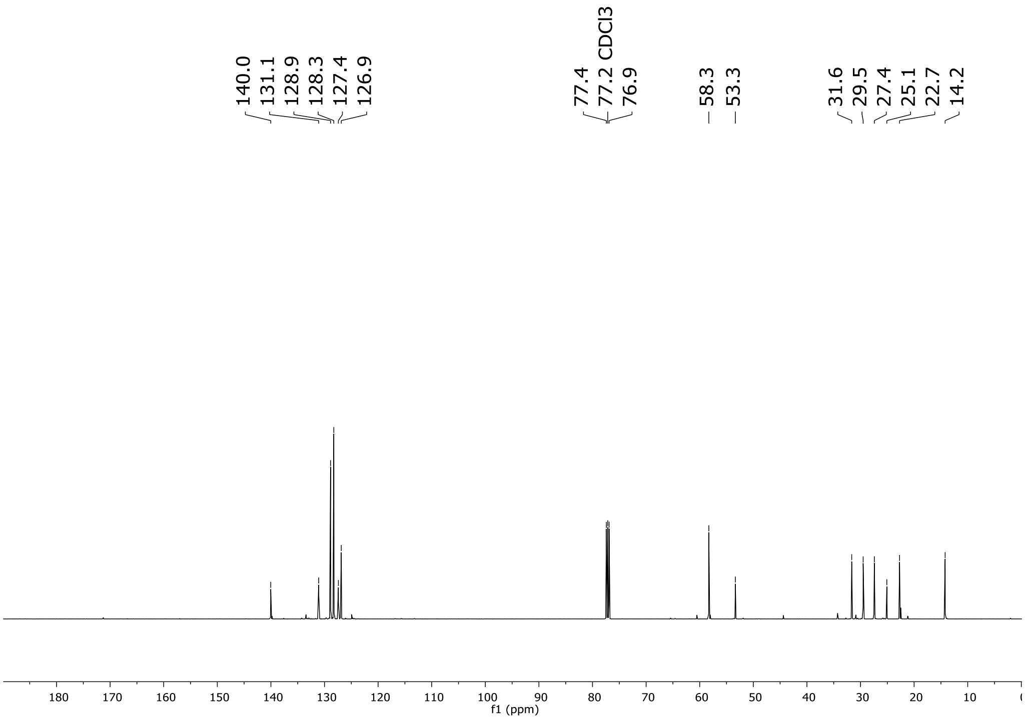

(E)-N-(4-fluorobenzyl)-N-((E)-hex-3-en-1-yl)hex-3-en-1-amine, 47a

^{1}H NMR (500 MHz, $CDCl_3$):

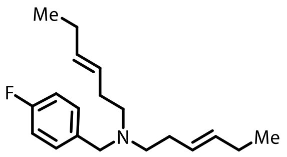

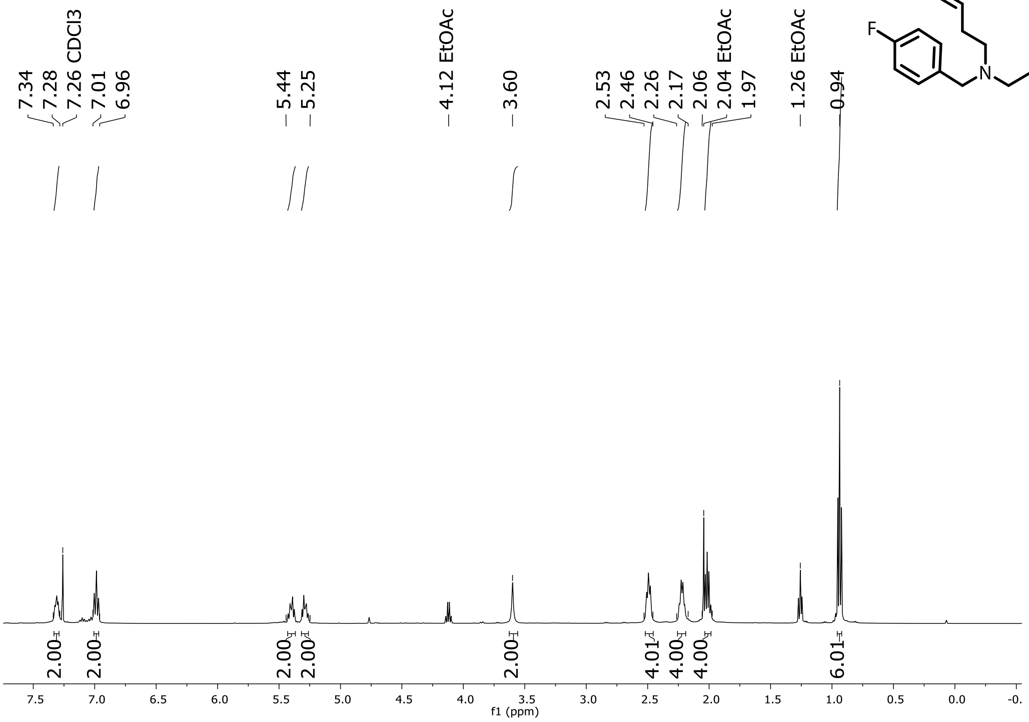

^{19}F NMR (376 MHz, $CDCl_3$):

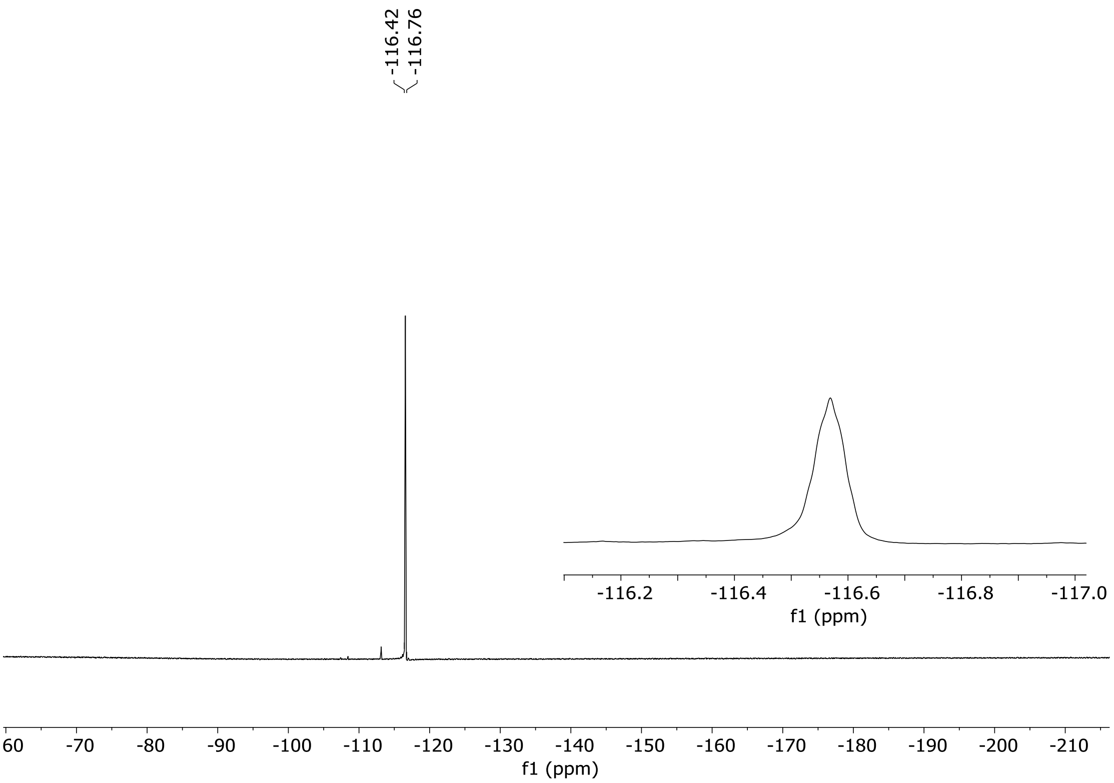

Back to top

^{13}C {^{1}H} NMR (125 MHz, $CDCl_3$):

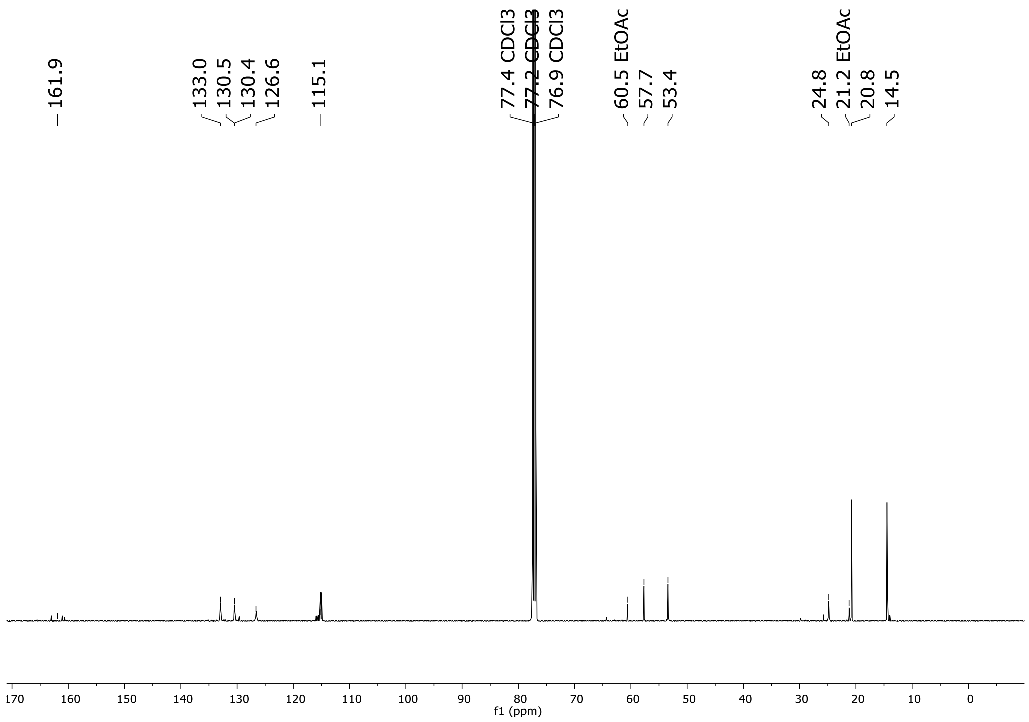

(E)-2-(hex-3-en-1-yl)isoindoline, 40a-trans

^{1}H NMR (500 MHz, $CDCl_3$):

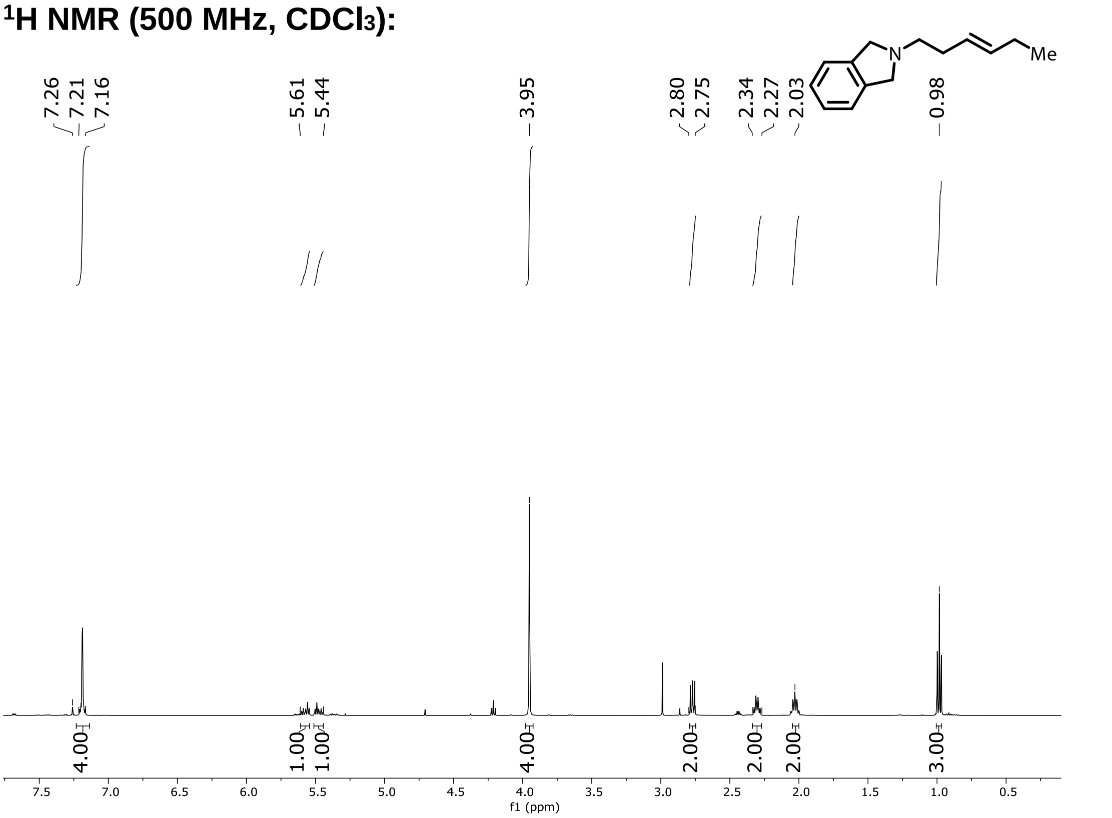

^{13}C {^{1}H} NMR (125 MHz, $CDCl_3$):

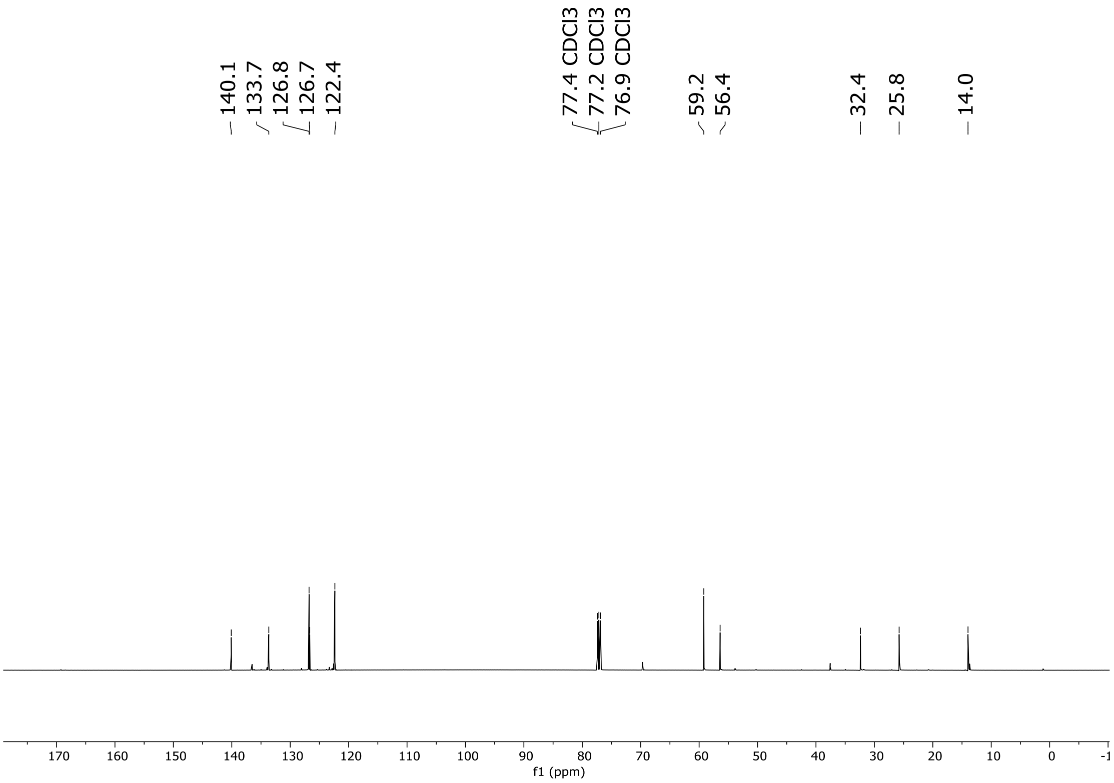

Back to top

NMR spectra of products

NMR spectra of terminal chlorofluorination products

N-(5-chloro-4-fluoropentyl)-4-methylbenzenesulfonamide, 2j

^{1}H NMR (500 MHz, CDCl$_3$):

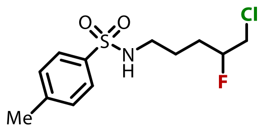

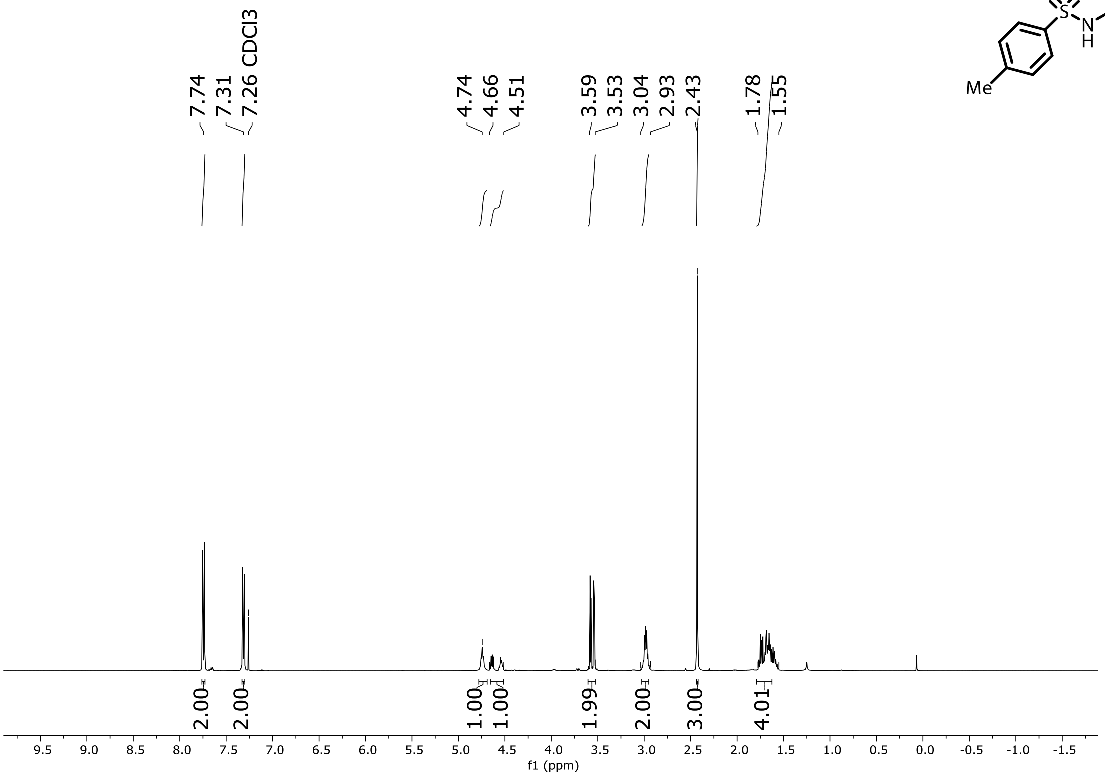

^{19}F NMR (376 MHz, CDCl$_3$):

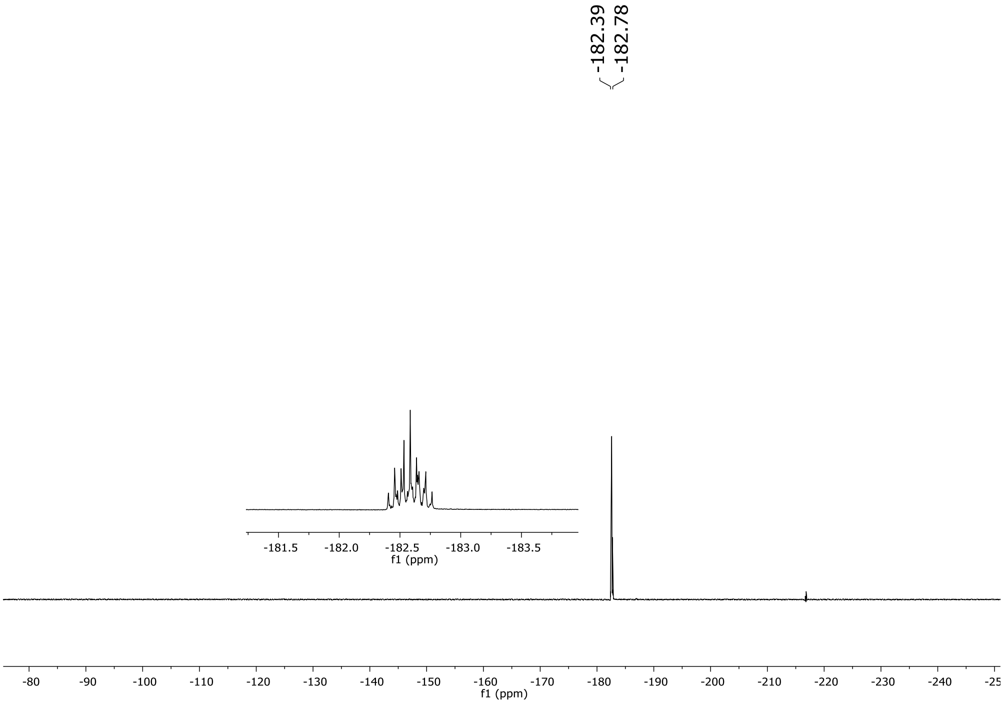

^{13}C {^{1}H} NMR (125 MHz, CDCl$_3$):

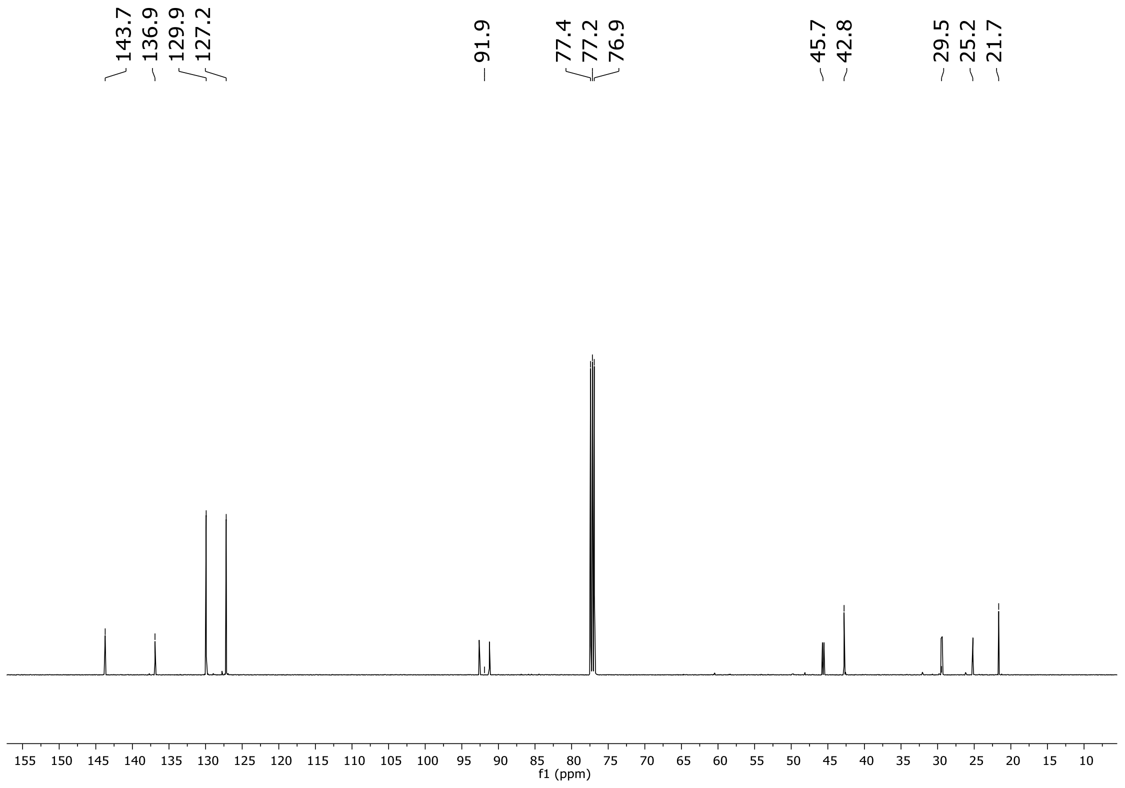
143.7
136.9
129.9
127.2
91.9
77.4
77.2
76.9
45.7
42.8
29.5
25.2
21.7
155 150 145 140 135 130 125 120 115 110 105 100 95 90 85 80 75 70 65 60 55 50 45 40 35 30 25 20 15 10
f1 (ppm)

N-(3-chloro-2-fluoropropyl)-N-(4-fluoro-2-methylphenyl)-4-nitrobenzenesulfonamide, 3j

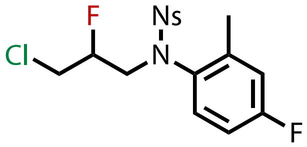

^{1}H NMR (400 MHz, $CDCl_3$):

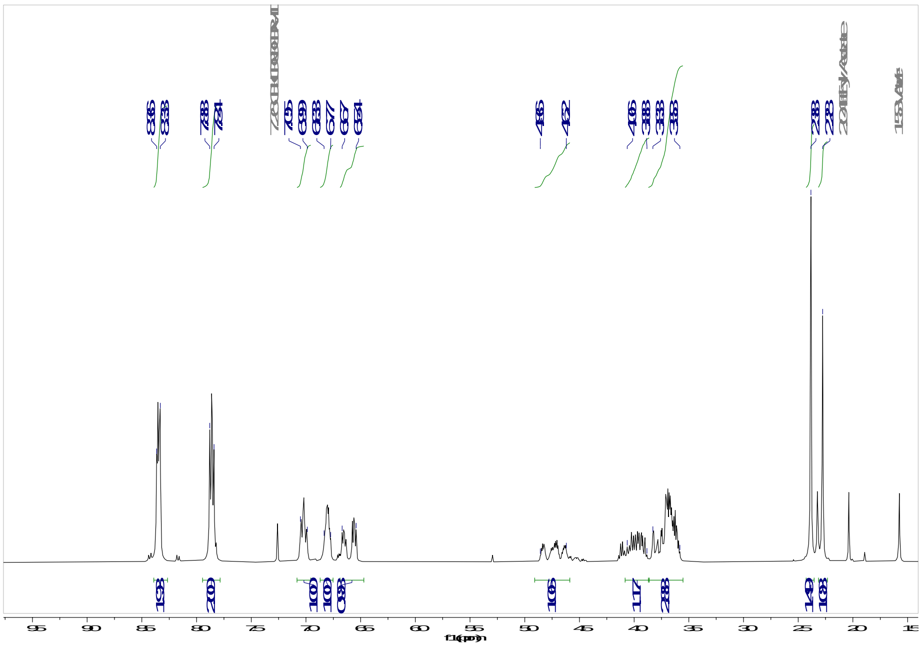

^{13}C NMR (101 MHz, $CDCl_3$):

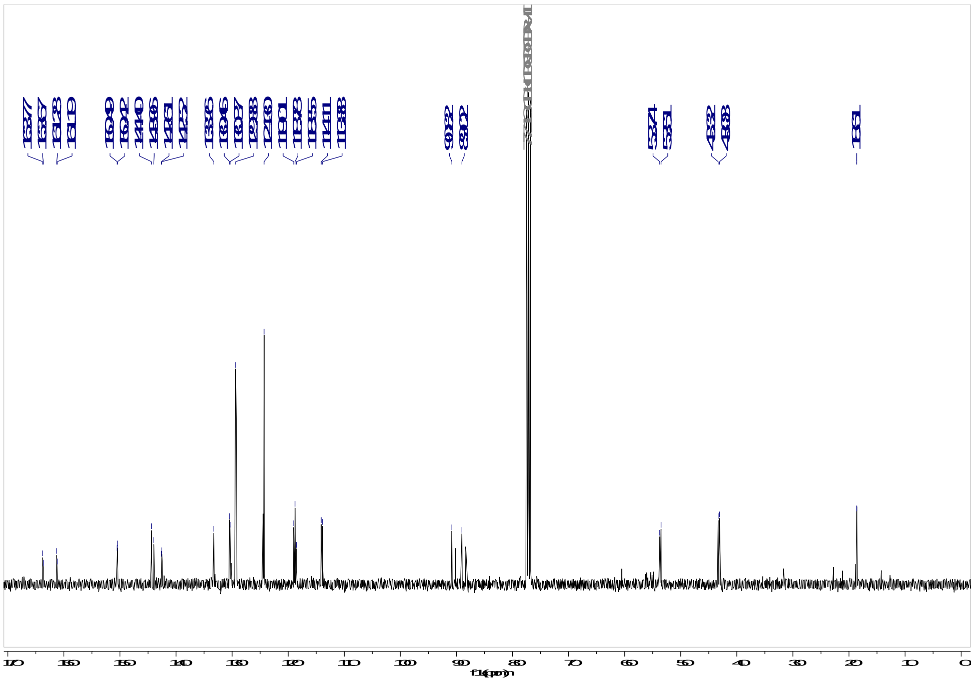

^{19}F NMR (377 MHz, $CDCl_3$):

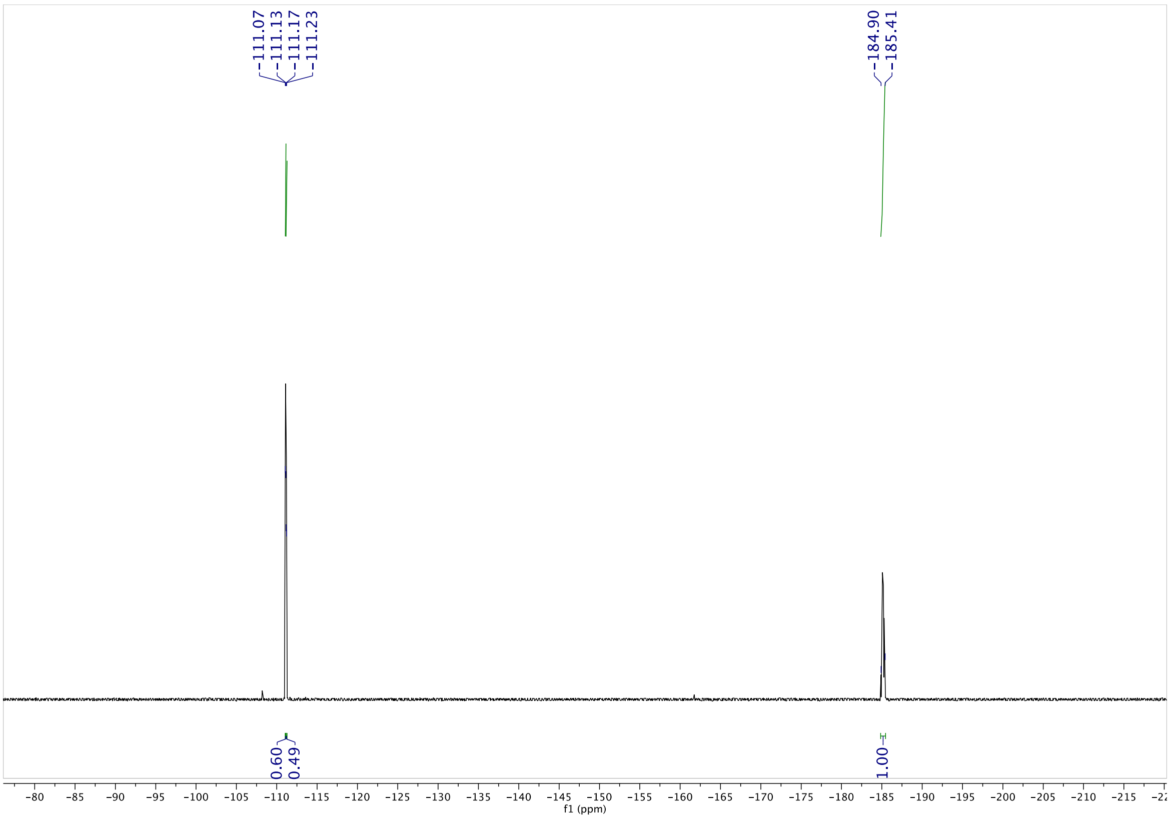
-111.07
-111.13
-111.17
-111.23
-184.90
-185.41
0.60
0.49
1.00
f1 (ppm)

N-(3-chloro-2-fluoropropyl)-N-(4-methylisoxazol-3-yl)-4-nitrobenzenesulfonamide, 4j

^{1}H NMR (500 MHz, $CDCl_3$):

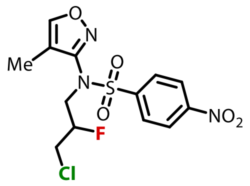

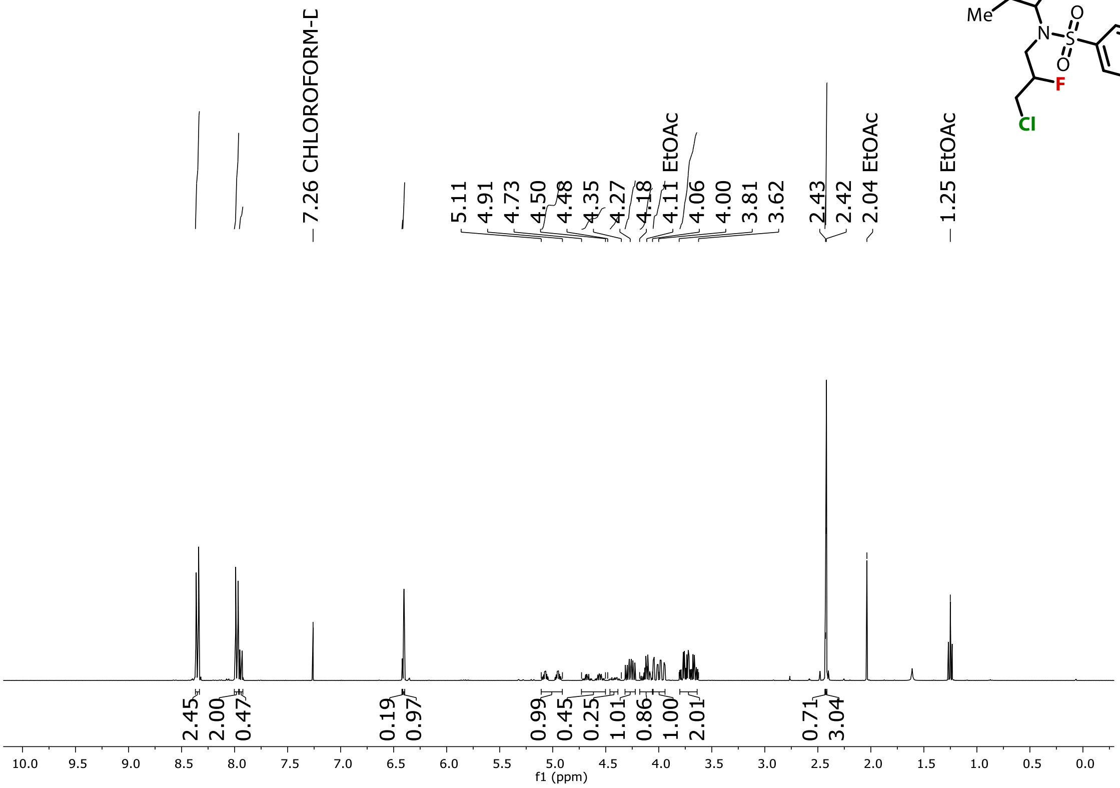

^{19}F NMR (376 MHz, $CDCl_3$):

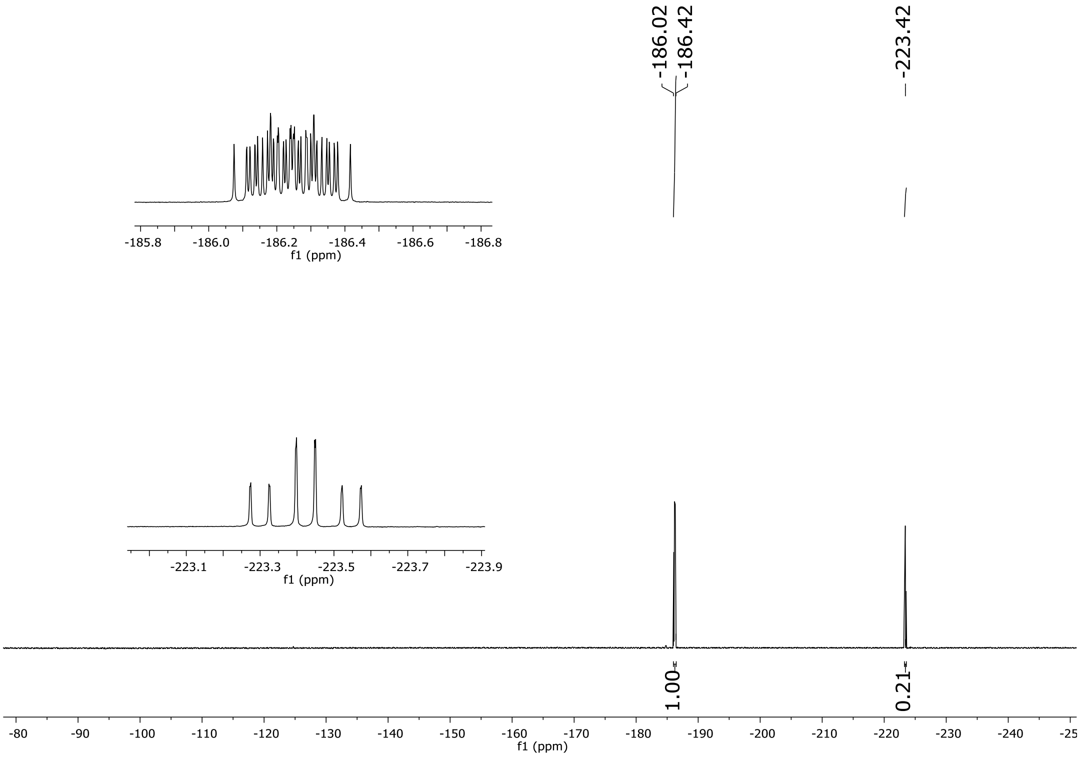

$^{13}C\ \{^1H\}$ NMR (125 MHz, CDCl$_3$):

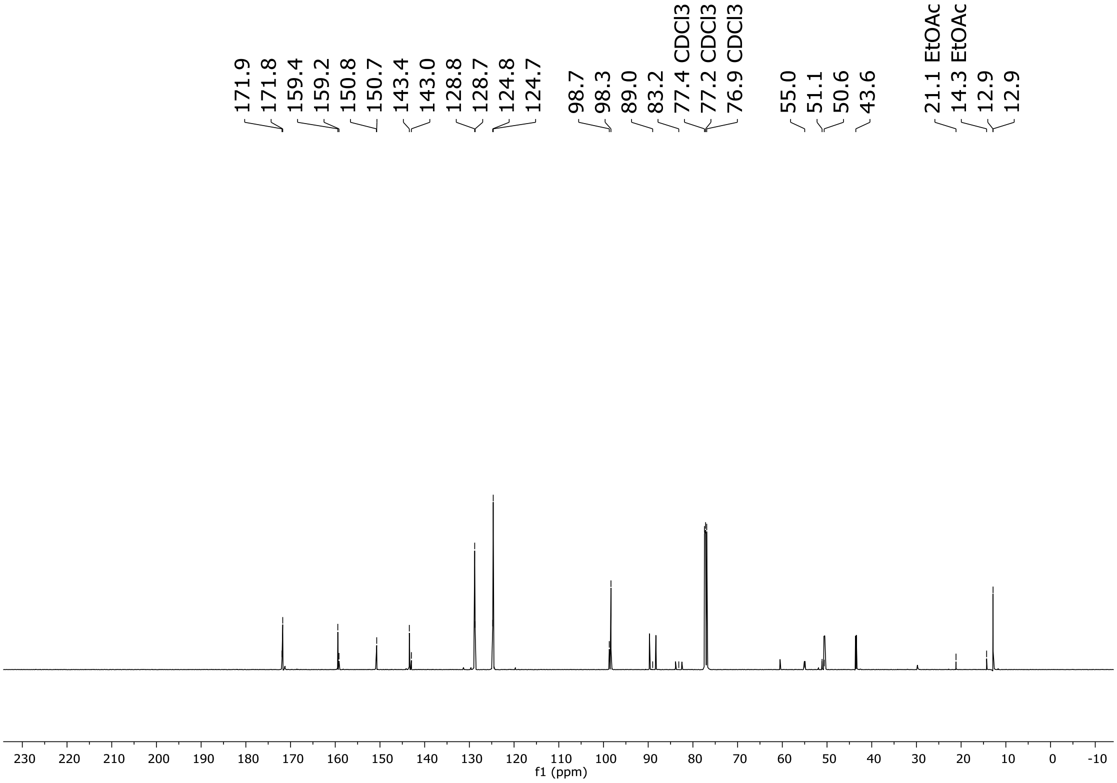

4-(3-chloro-2-fluoropropyl)-N,N-dimethylaniline, 5j

^{1}H NMR (400 MHz, $CDCl_3$):

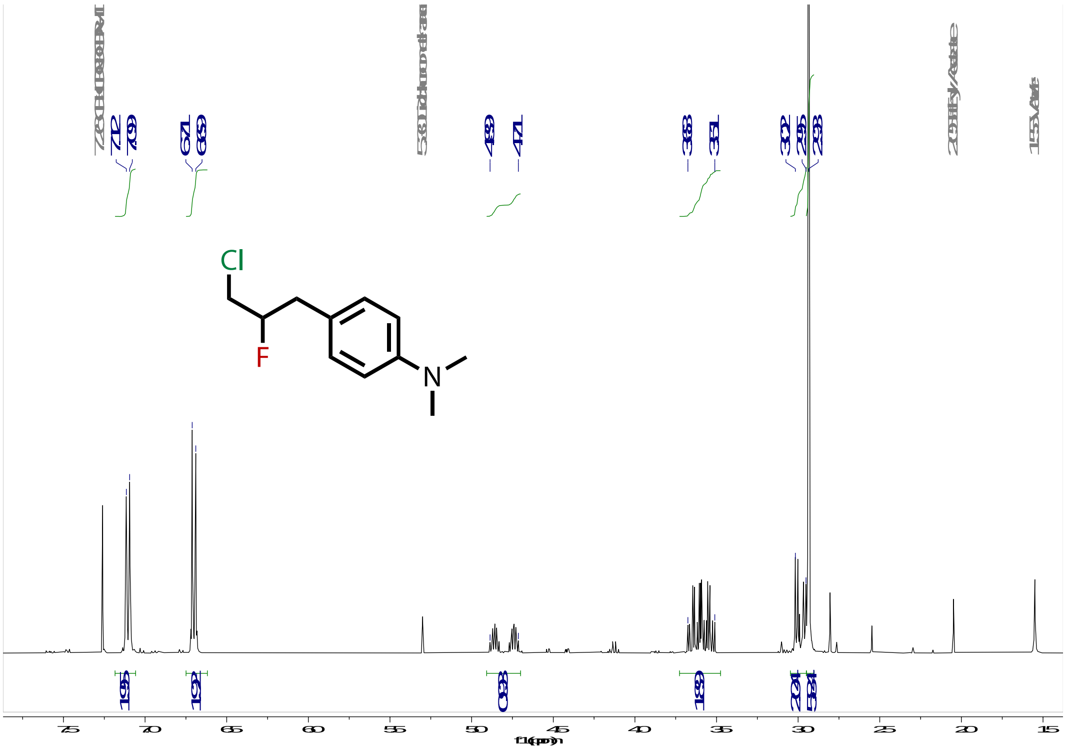

^{13}C NMR (101 MHz, $CDCl_3$):

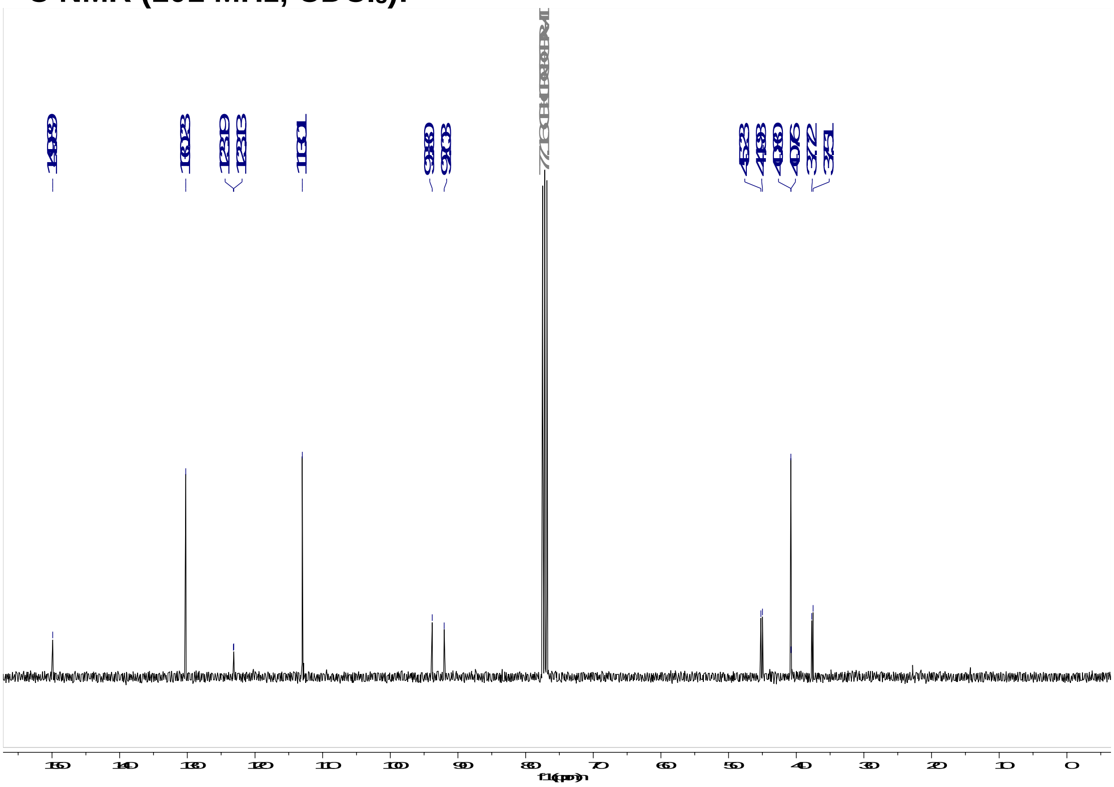

^{19}F NMR (377 MHz, $CDCl_3$):

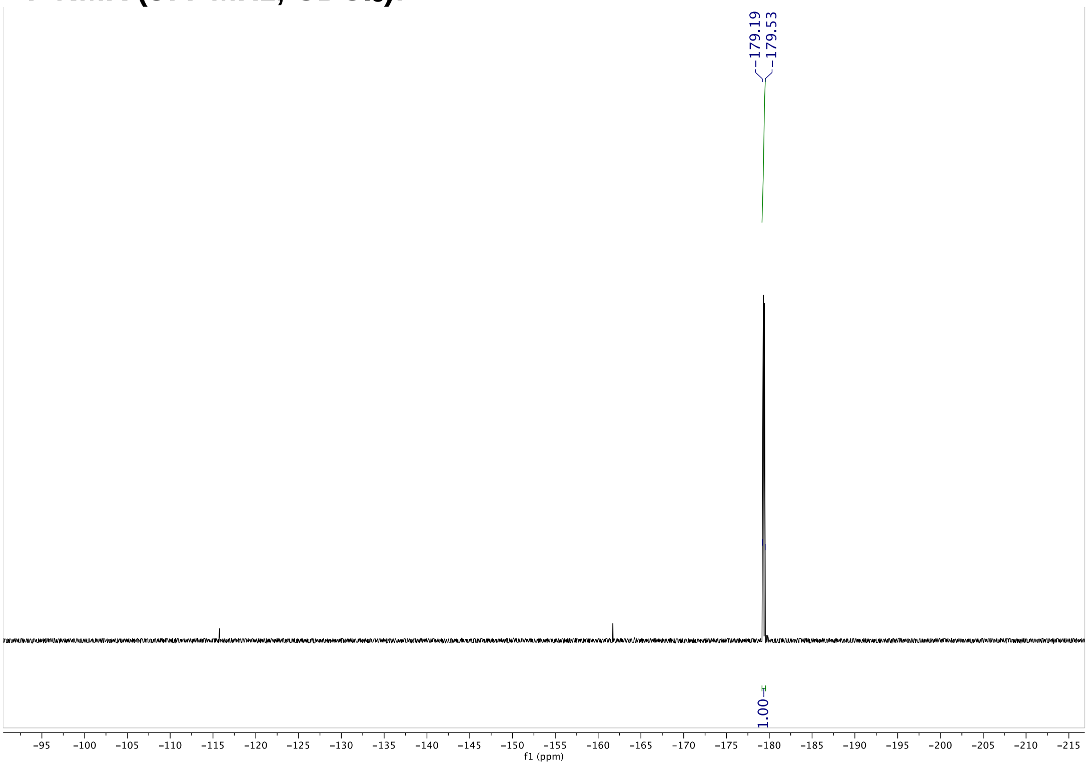

1-(2-chloro-3-fluoropropyl)-4-fluorobenzene, 6j

^{1}H NMR (400 MHz, CDCl3):

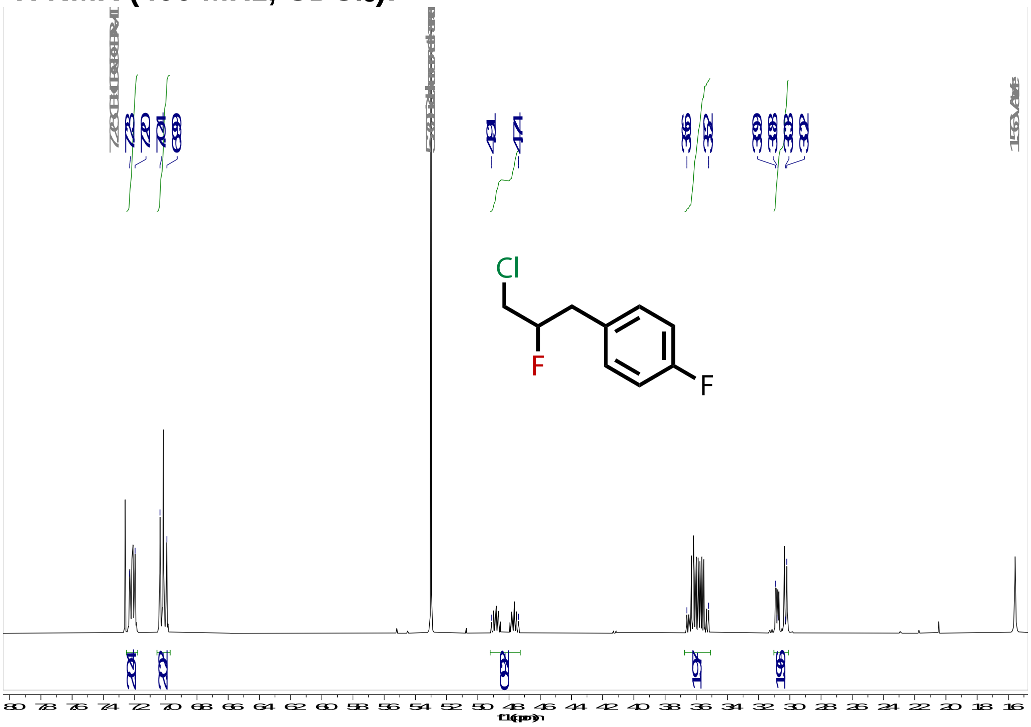

^{13}C NMR (101 MHz, CDCl3):

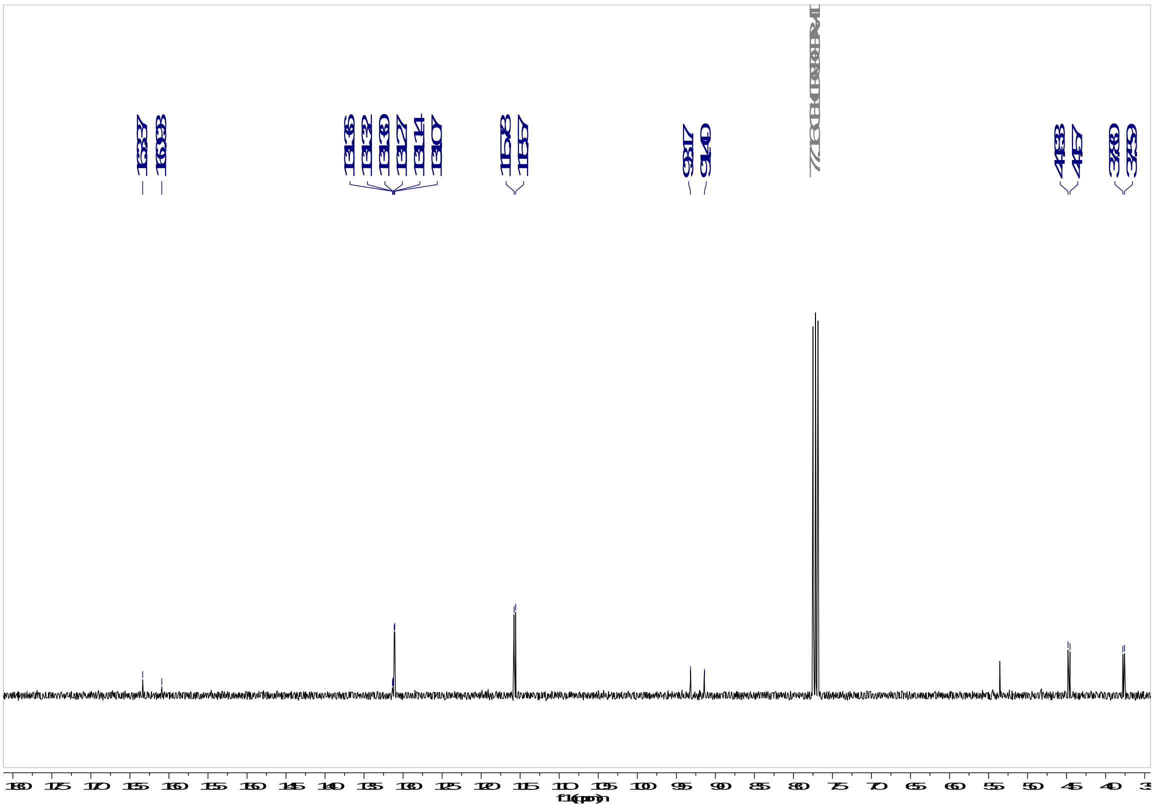

^{19}F NMR (376 MHz, CDCl3):

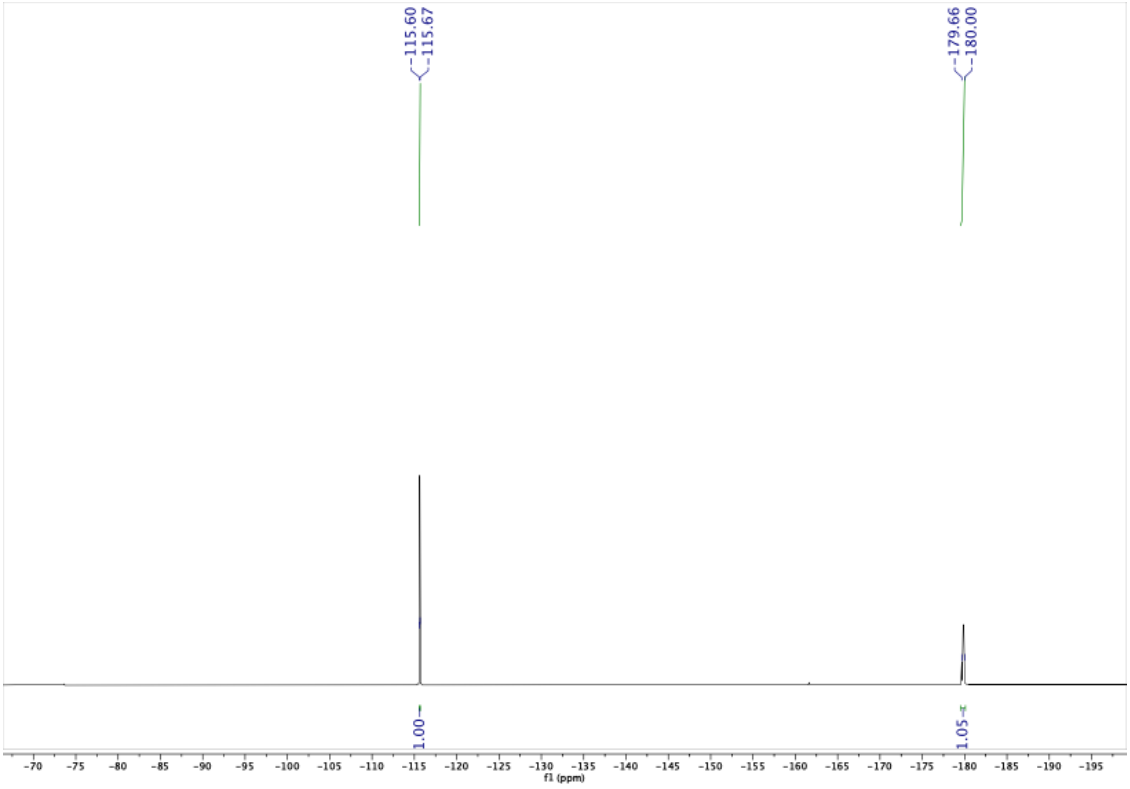
-115.60
-115.67
-179.66
-180.00
1.00
1.05
-70 -75 -80 -85 -90 -95 -100 -105 -110 -115 -120 -125 -130 -135 -140 -145 -150 -155 -160 -165 -170 -175 -180 -185 -190 -195
f1 (ppm)

11-chloro-10-fluoroundecan-1-ol, 8j

^{1}H NMR (400 MHz, $CDCl_3$):

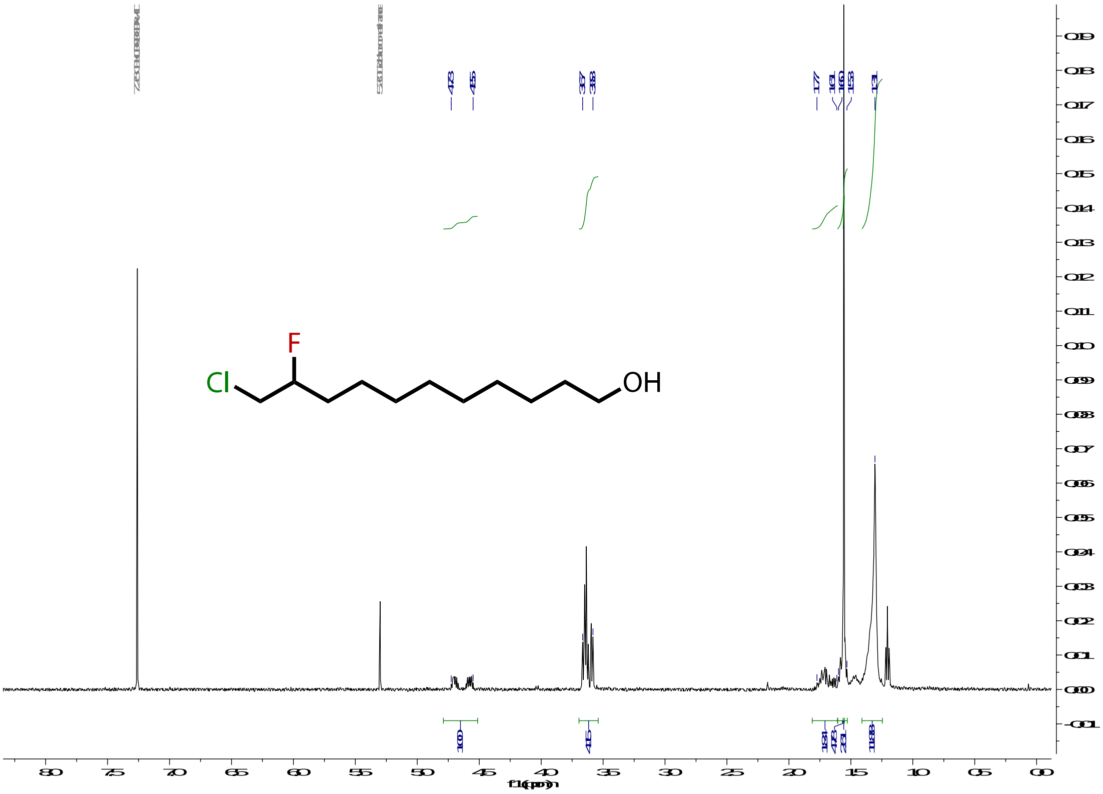

^{19}F NMR (376 MHz, $CDCl_3$):

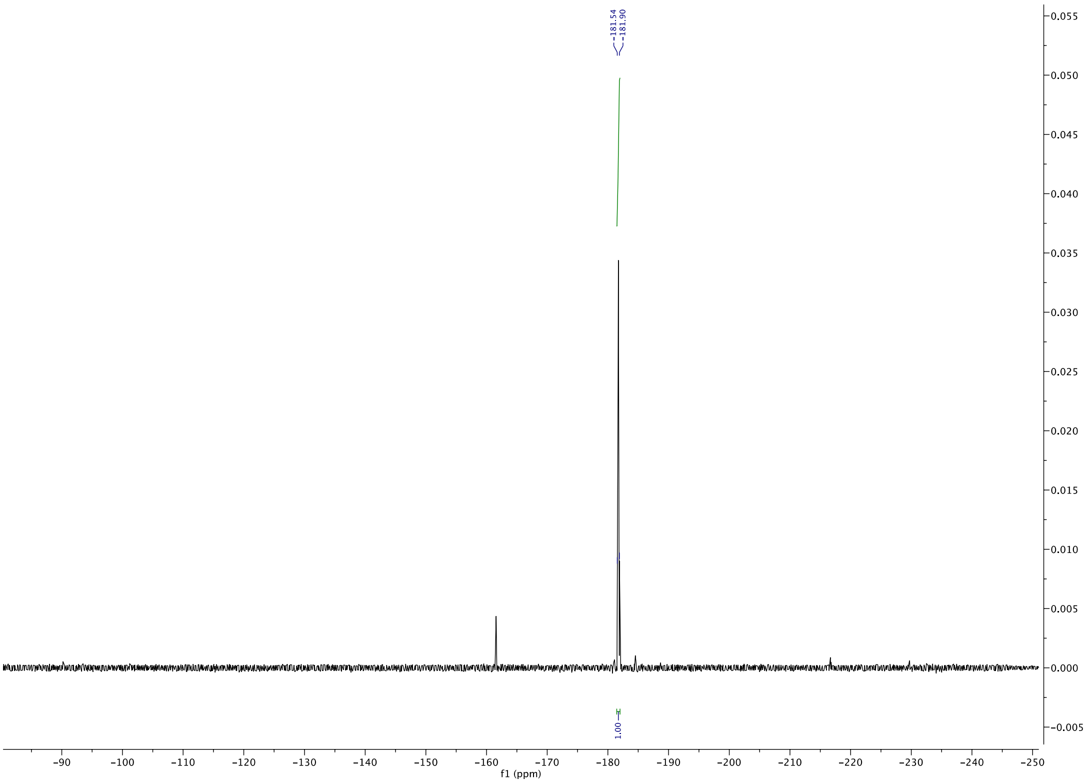

2-(11-chloro-10-fluoroundecyl)isoindoline-1,3-dione, 9j

^{1}H NMR (500 MHz, $CDCl_3$):

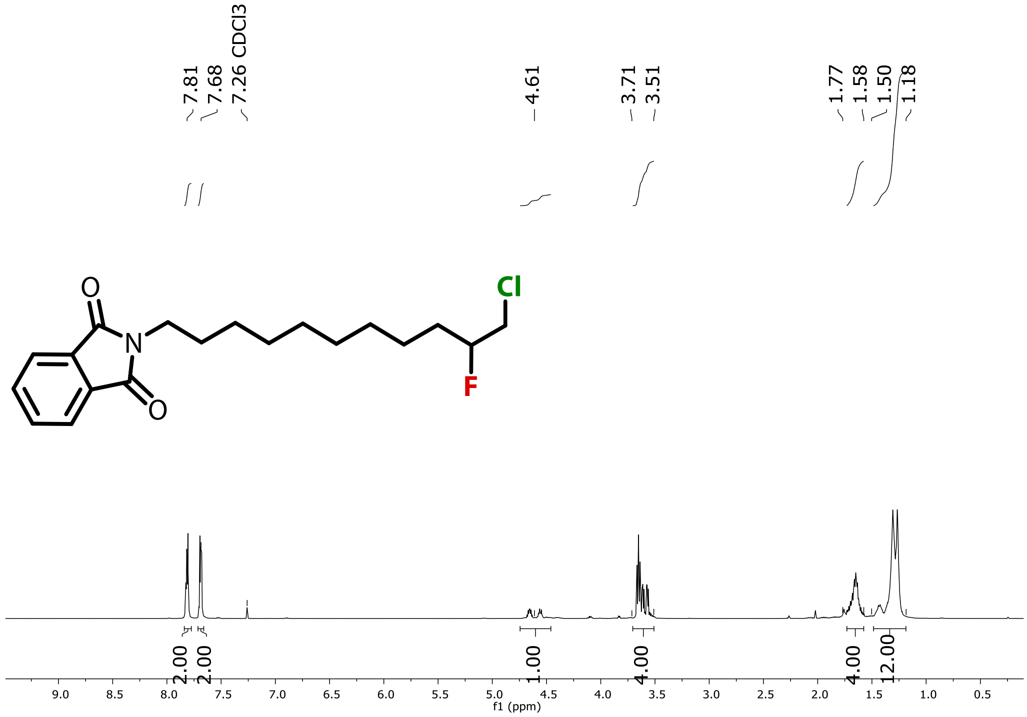

^{19}F NMR (376 MHz, $CDCl_3$):

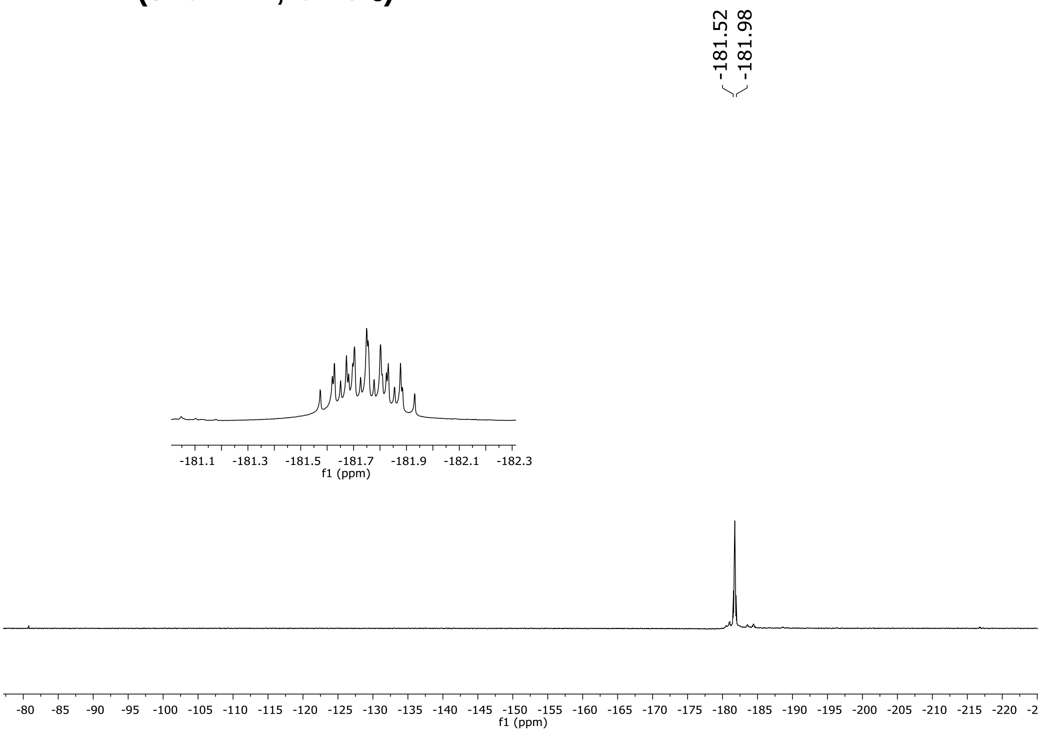

^{13}C NMR (151 MHz, $CDCl_3$):

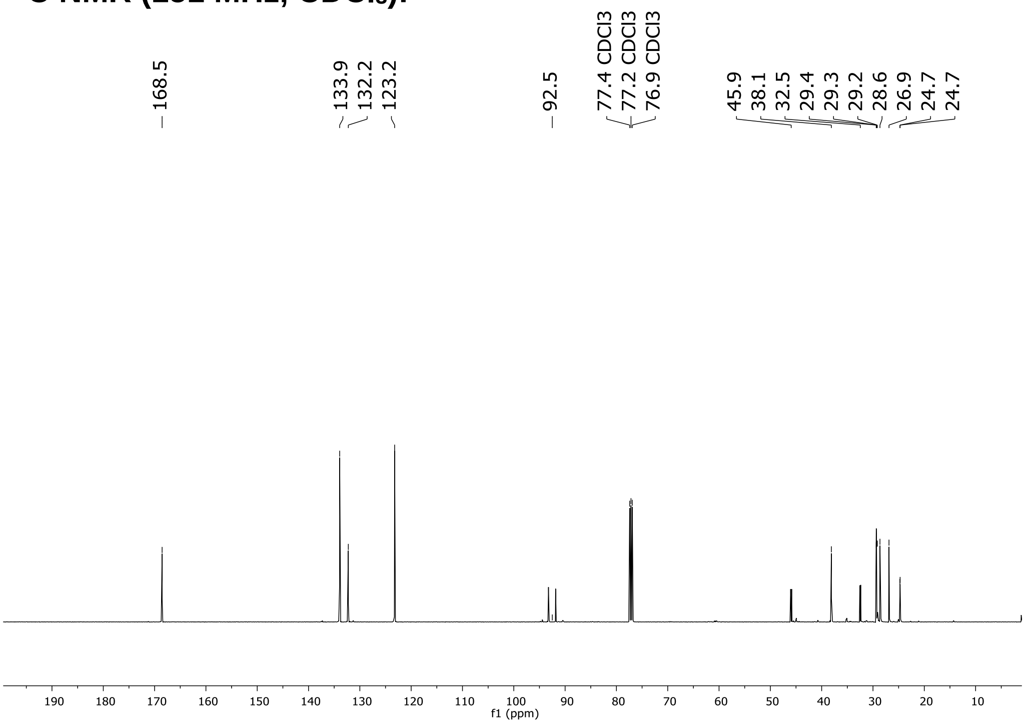

1-(2-chloro-1-fluoroethyl)-3-nitrobenzene, 10j

^{1}H NMR (400 MHz, $CDCl_3$):

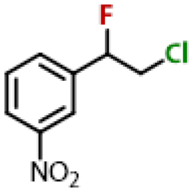

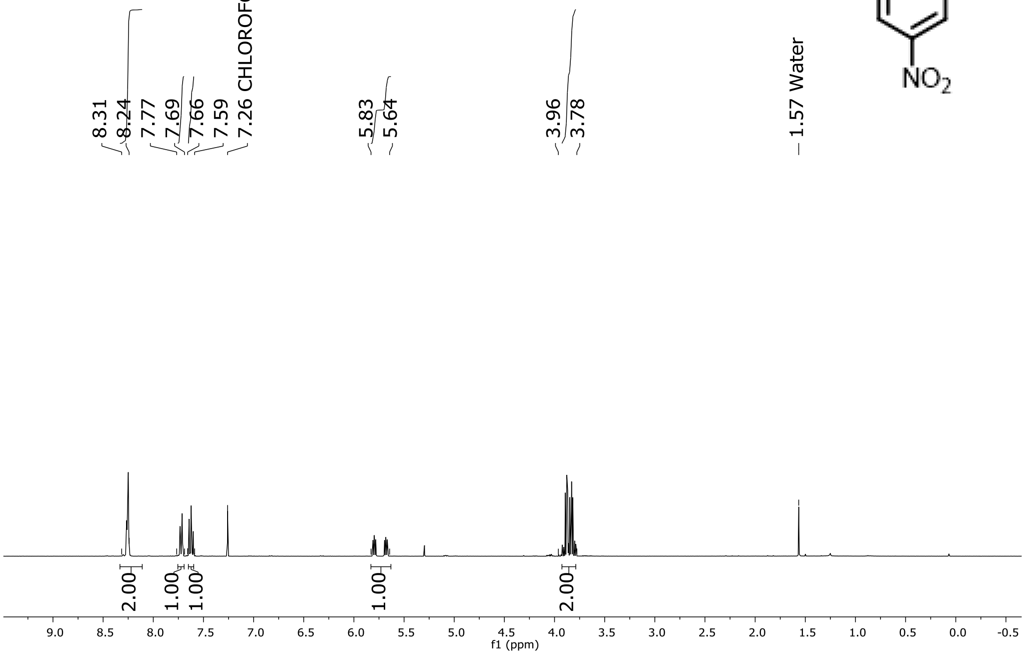

^{19}F NMR (371 MHz, $CDCl_3$):

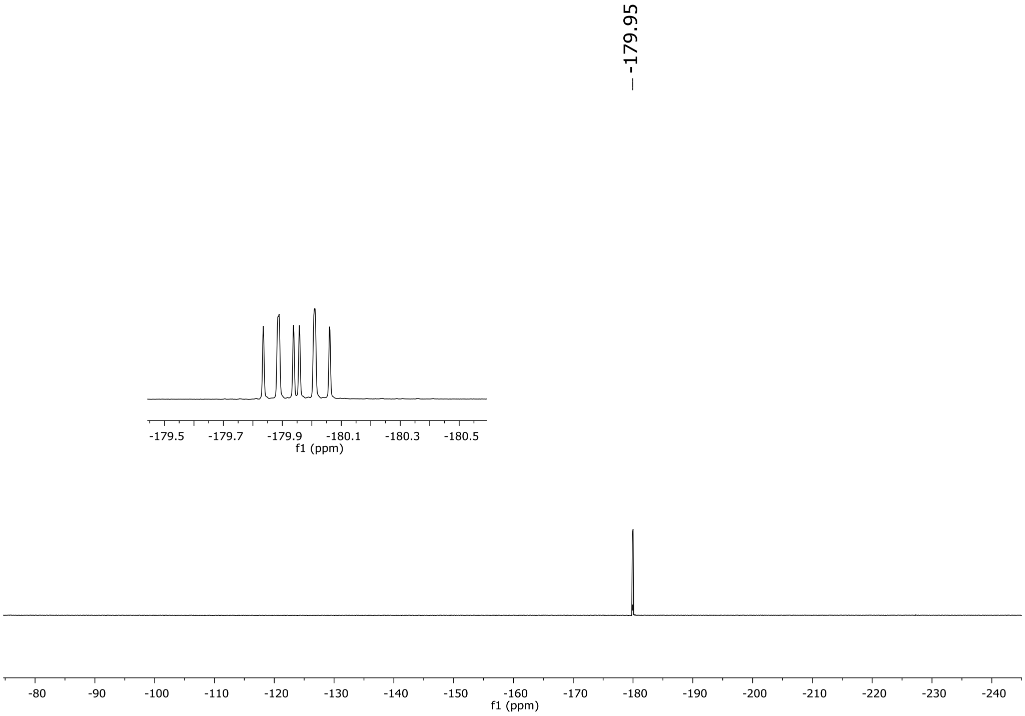

^{13}C NMR (151 MHz, $CDCl_3$):

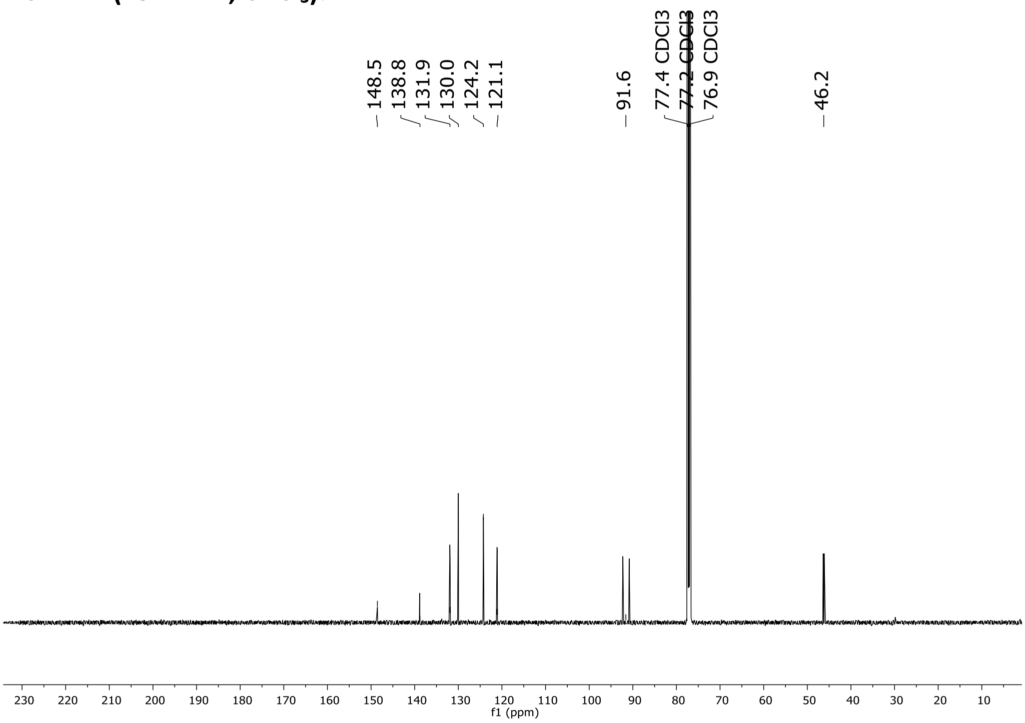

(S)-((1S,2S,4S,5R)-5-((R)-1-chloro-2-fluoroethyl)quinuclidin-2-yl)(quinolin-4-yl)methyl acetate, 11k

^{1}H NMR (500 MHz, $CDCl_3$):

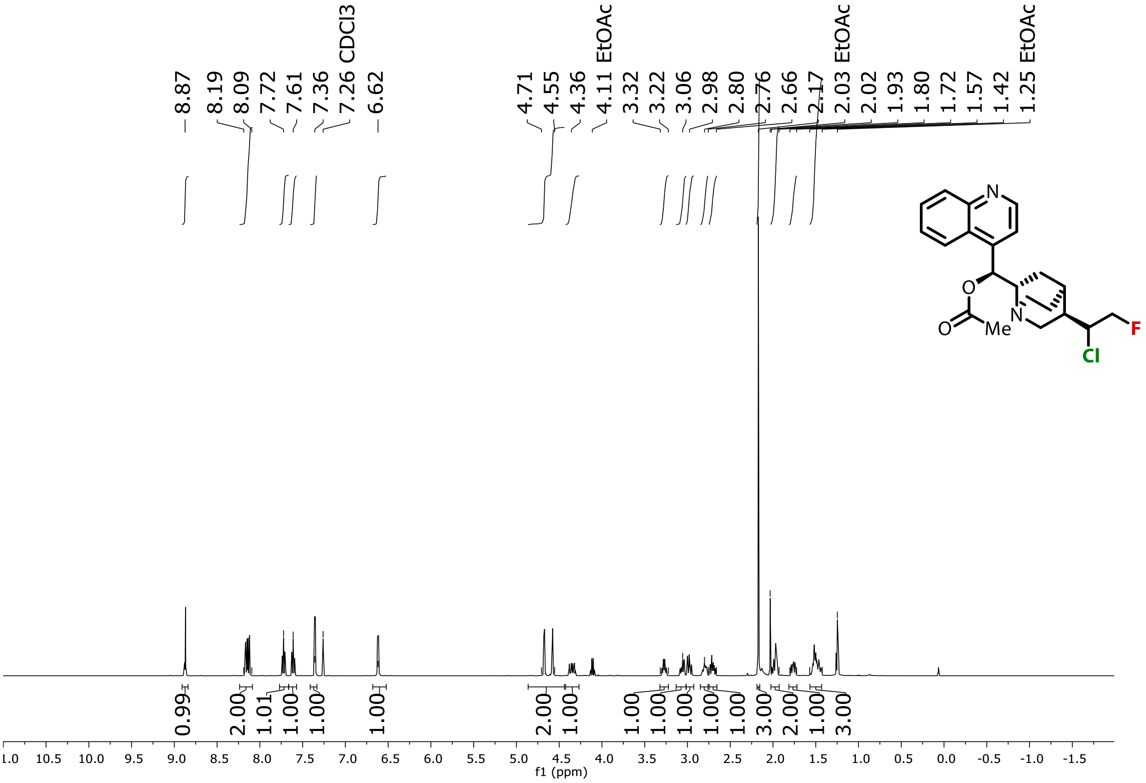

^{19}F NMR (376 MHz, $CDCl_3$):

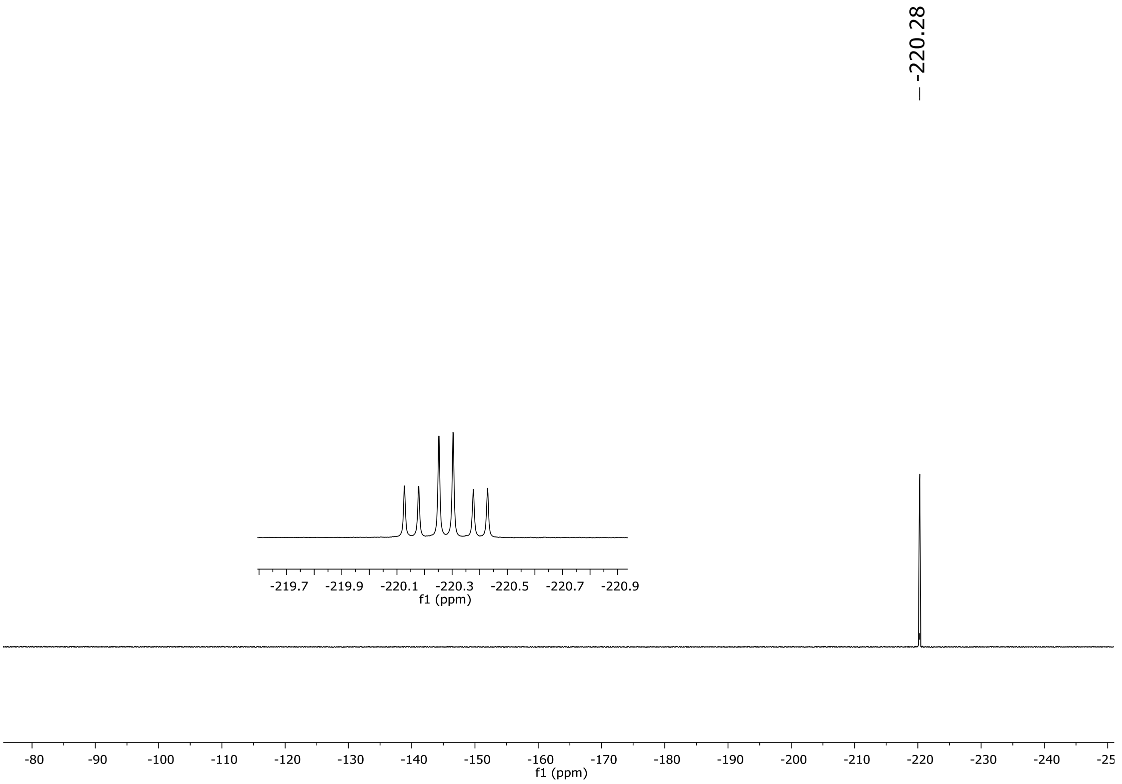

Back to top

$^{13}C\ \{^{1}H\}$ NMR (125 MHz, $CDCl_3$):

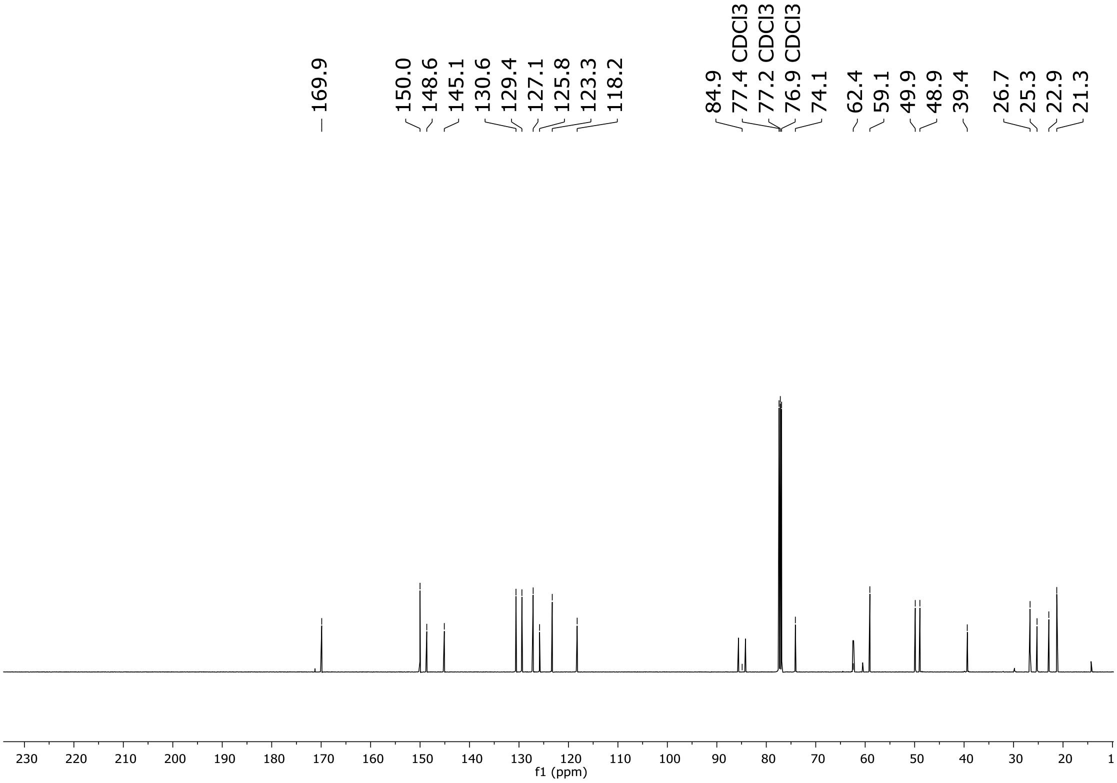

1-(2-(3-chloro-2-fluoropropoxy)-2-(2,4-dichlorophenyl)ethyl)-1H-imidazole, 12j

^{1}H NMR (500 MHz, $CDCl_3$):

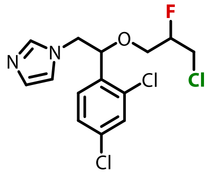

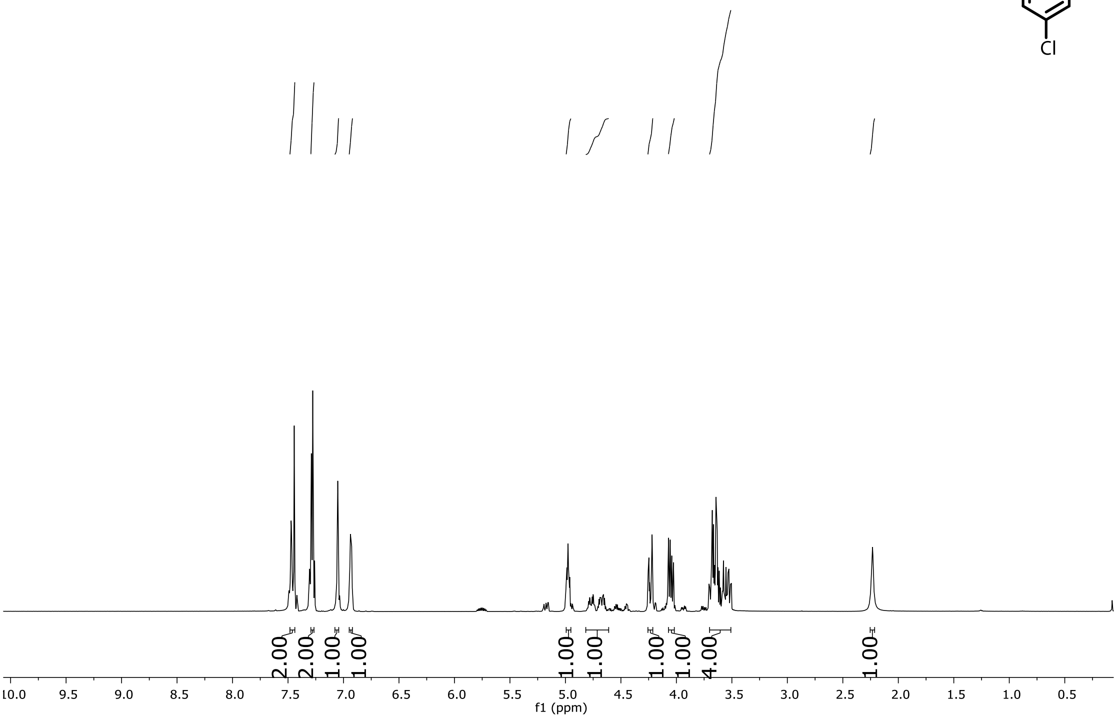

^{19}F NMR (376 MHz, $CDCl_3$):

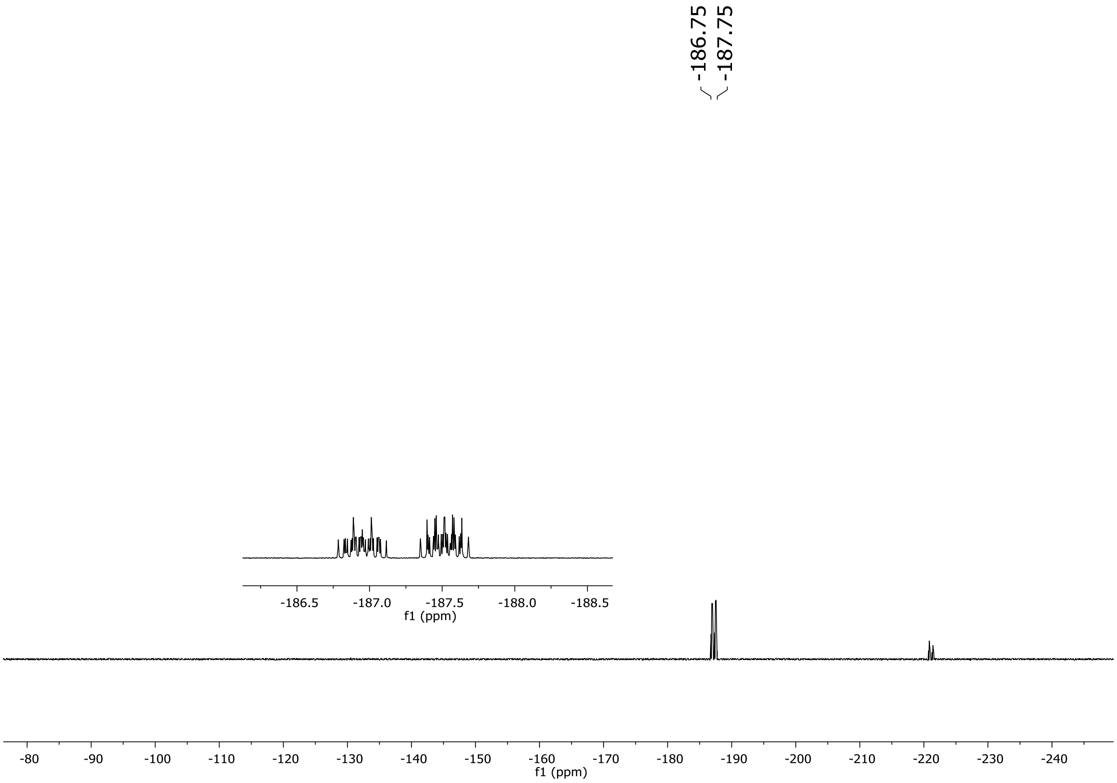

$^{13}C\ \{^{1}H\}$ NMR (125 MHz, CDCl₃):

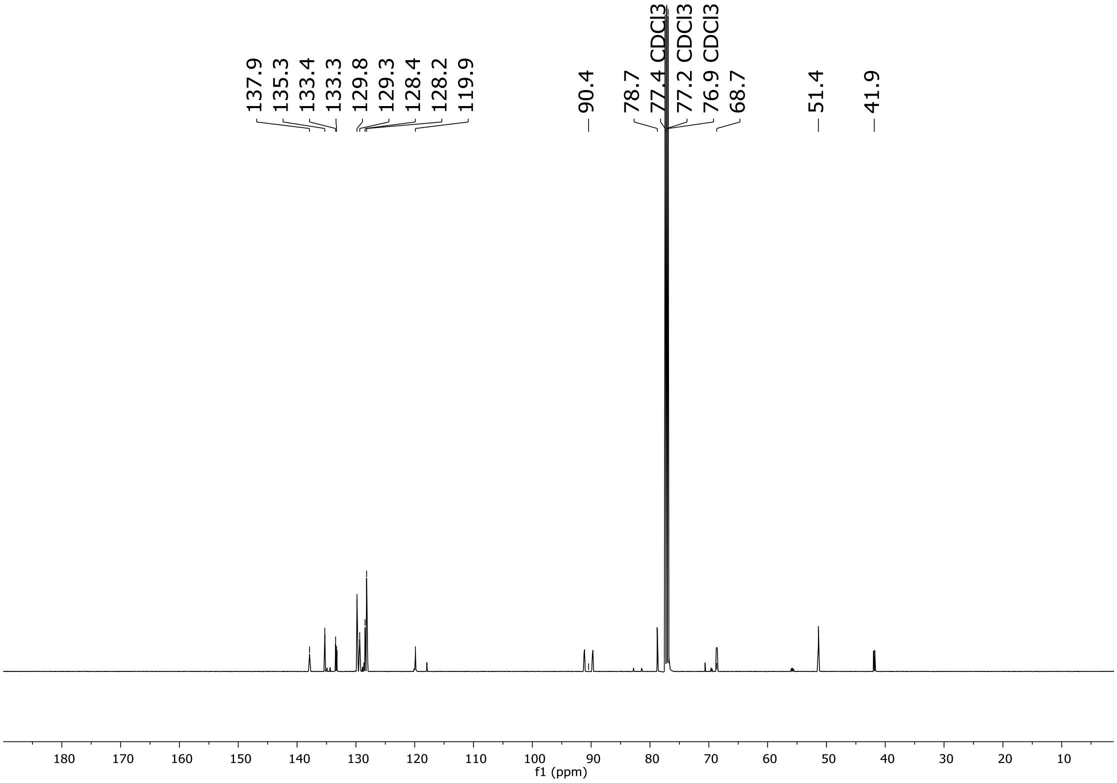

Benzyl 2-(3-chloro-4-(3-chloro-2-fluoropropoxy)phenyl)acetate, 13j

^{1}H NMR (400 MHz, CDCl$_3$):

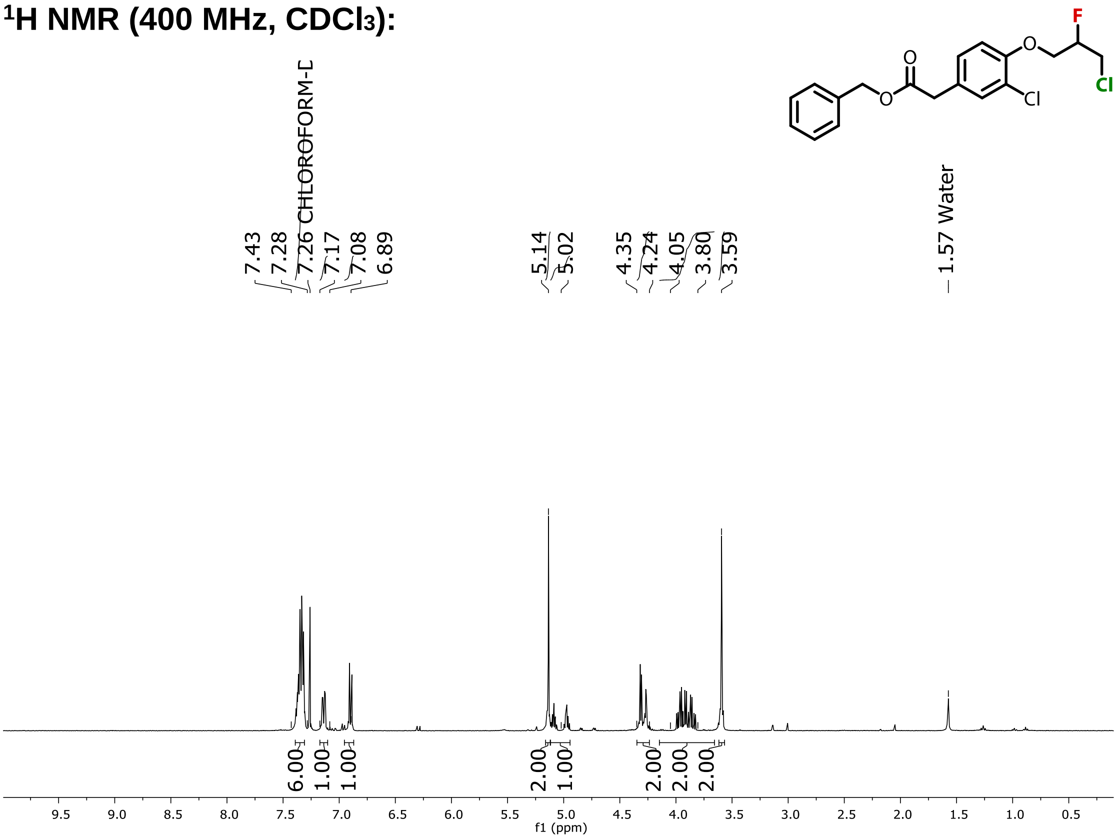

^{19}F NMR (376 MHz, CDCl$_3$):

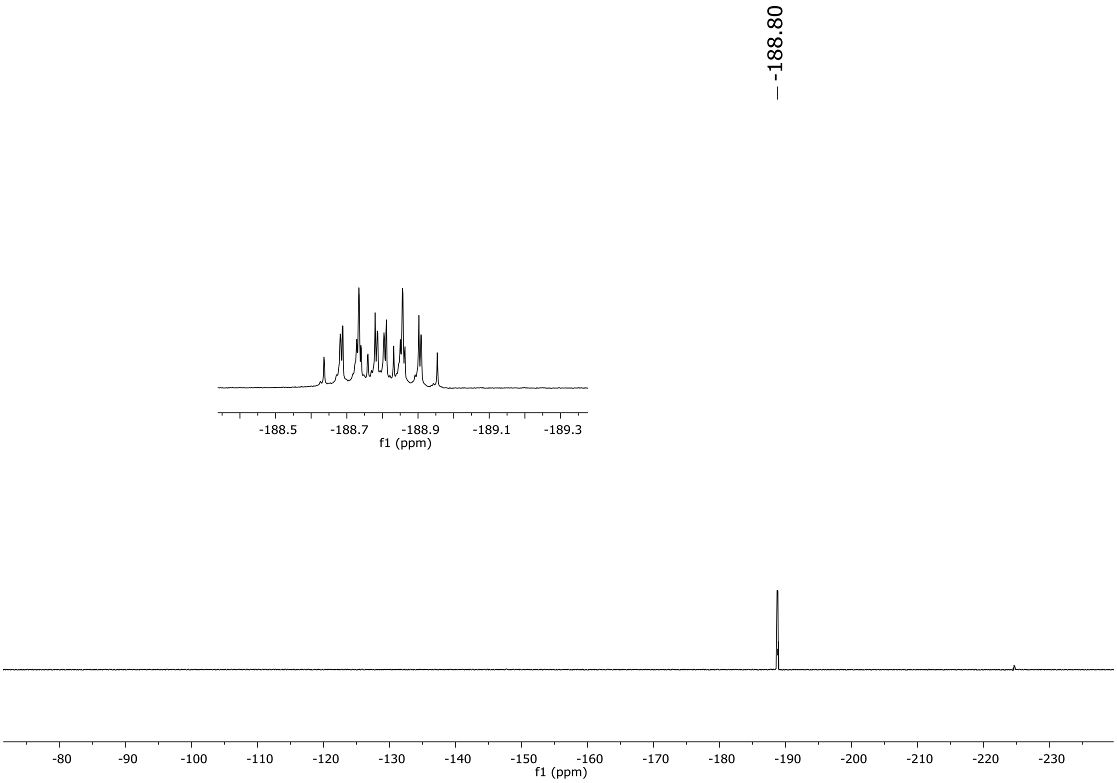

^{13}C {^{1}H} NMR (100 MHz, $CDCl_3$):

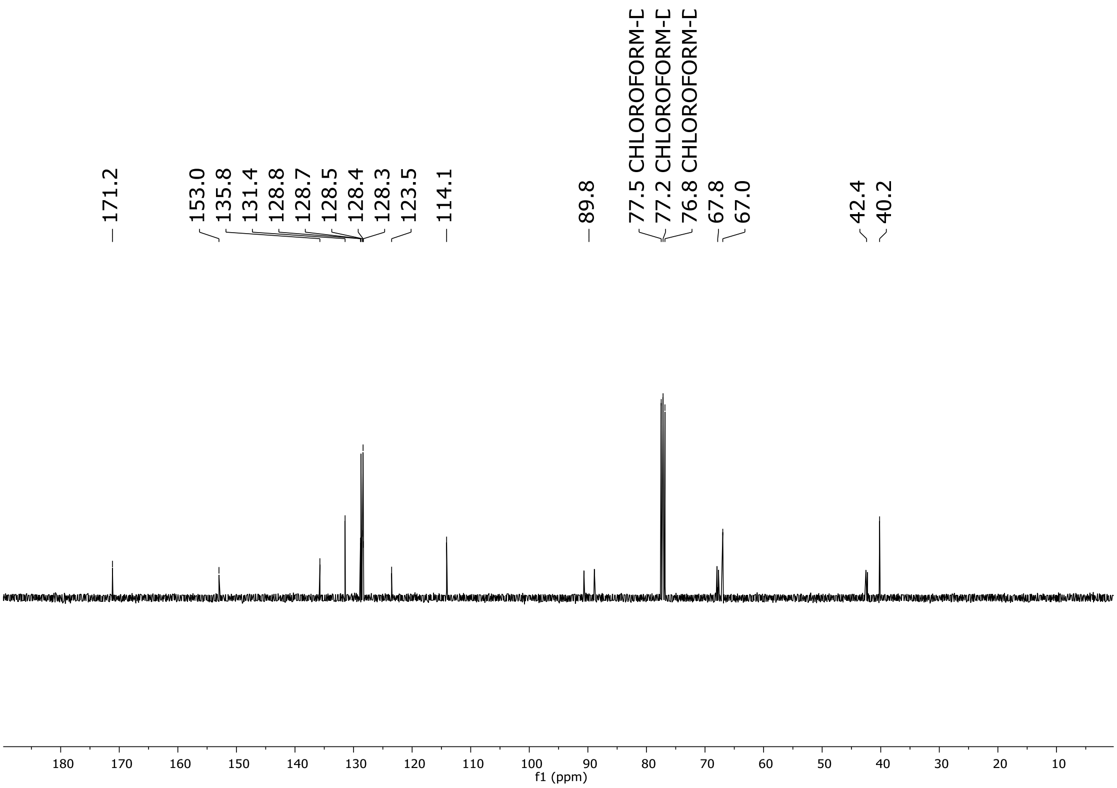

(4S)-4-(1-chloro-2-fluoropropan-2-yl)-2-methylcyclohex-2-en-1-one, 14j

^{1}H NMR (500 MHz, $CDCl_3$):

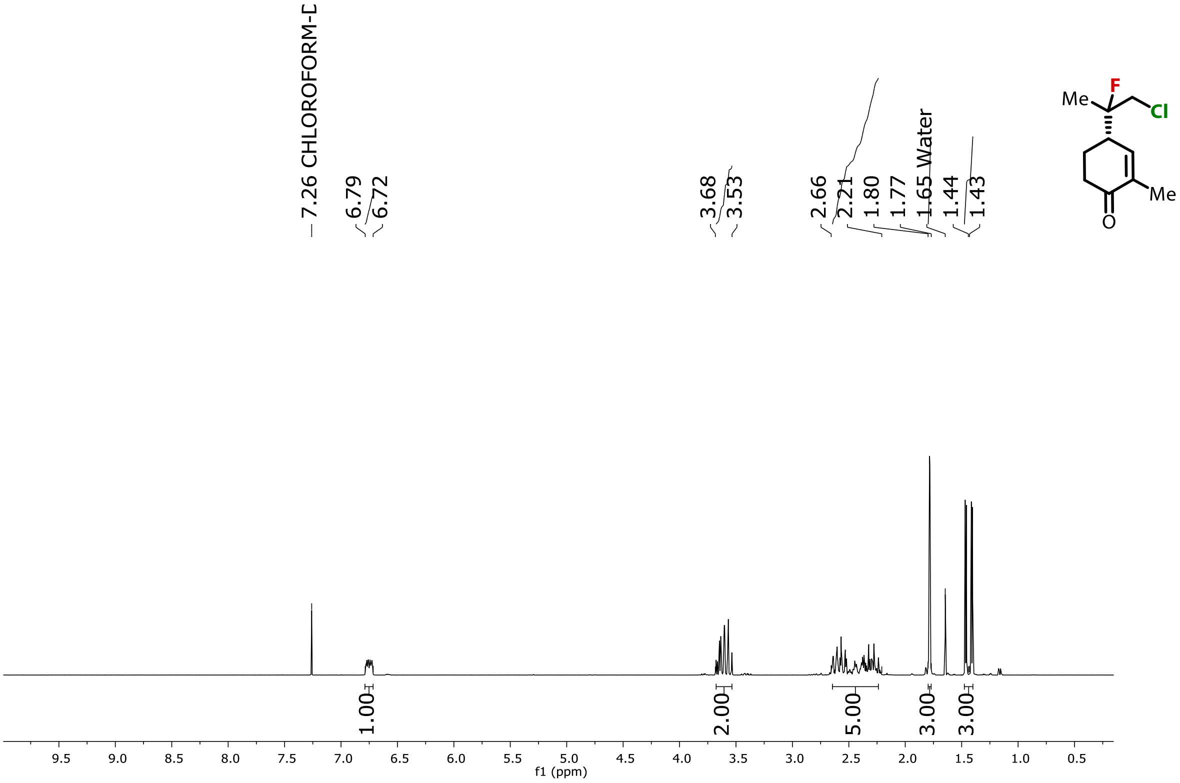

^{19}F NMR (376 MHz, $CDCl_3$):

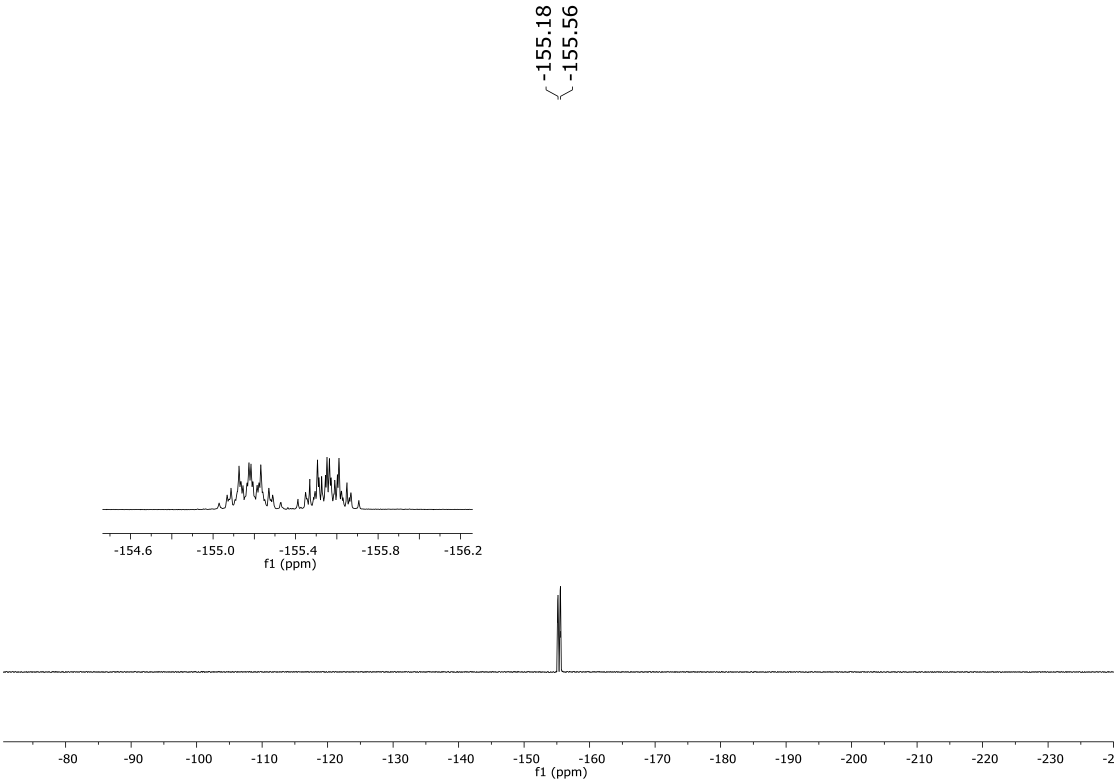

Back to top

$^{13}C\ \{^{1}H\}$ NMR (125 MHz, $CDCl_3$):

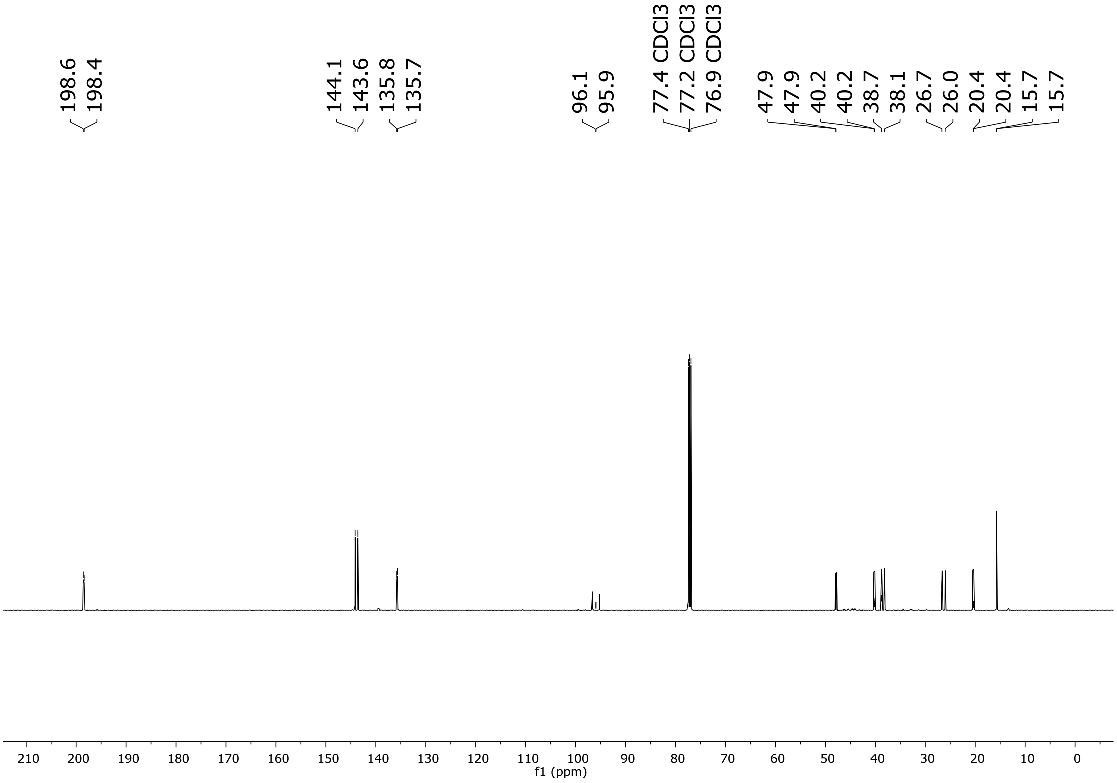

6-chloro-5-fluorohexyl 3,5-dinitrobenzoate, 15j

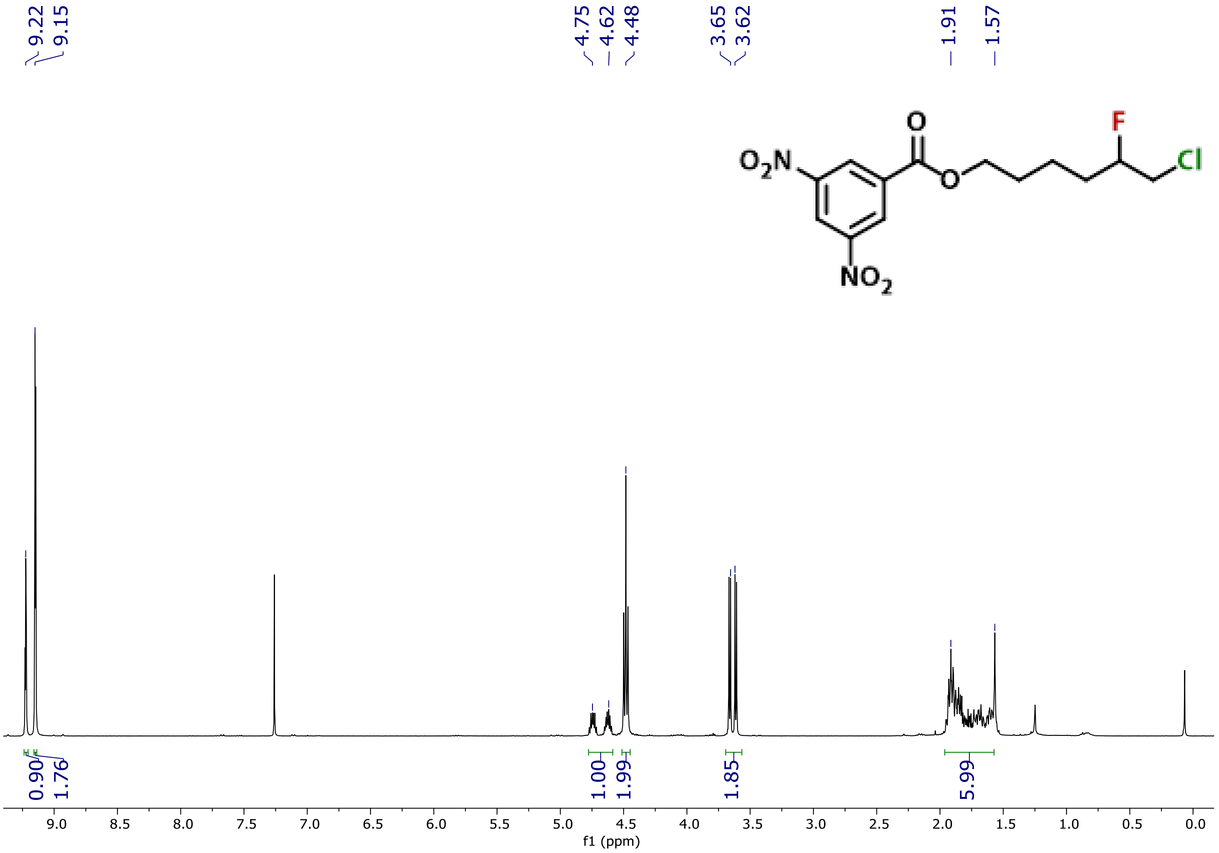

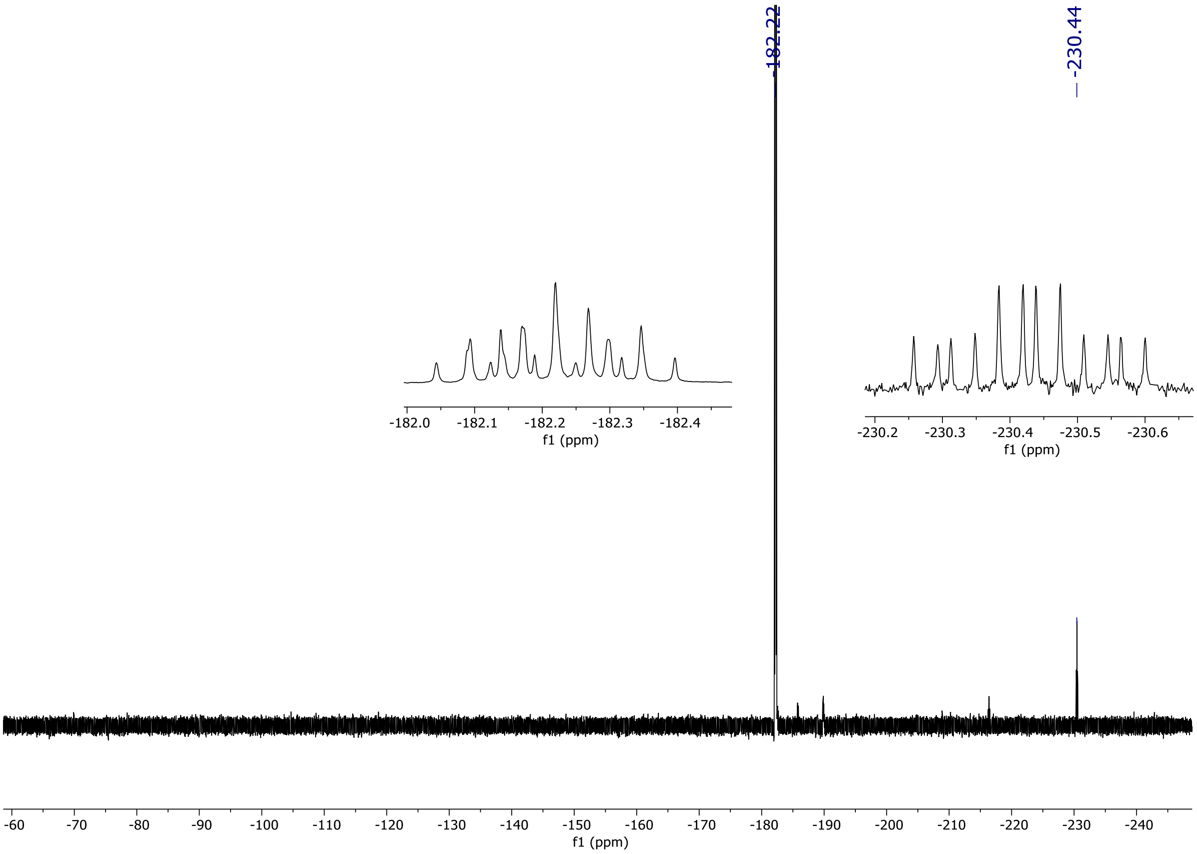

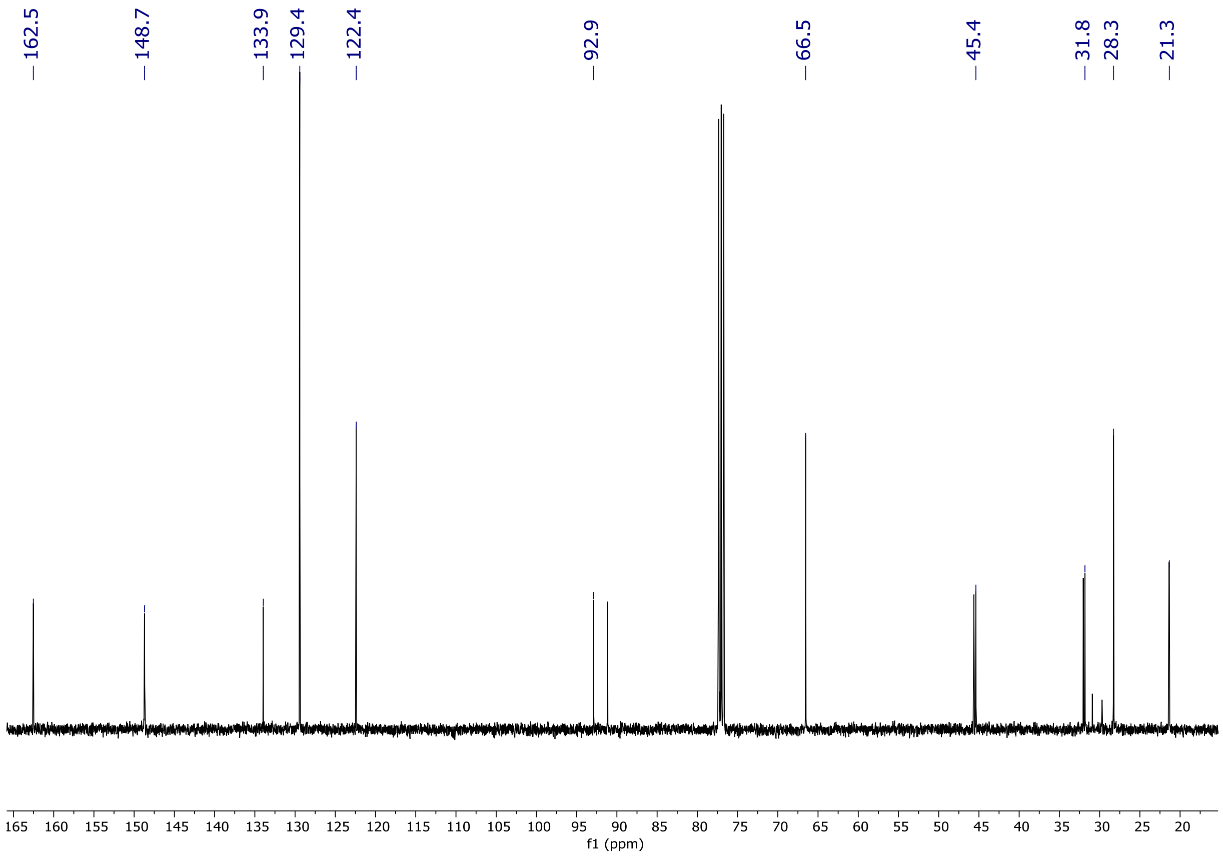
162.5
148.7
133.9
129.4
122.4
92.9
66.5
45.4
31.8
28.3
21.3
165 160 155 150 145 140 135 130 125 120 115 110 105 100 95 90 85 80 75 70 65 60 55 50 45 40 35 30 25 20
f1 (ppm)

NMR spectra of internal alkene *anti*-chlorofluorination products

N-benzyl-N-((3S,4S)-3-chloro-4-fluorohexyl)-4-fluoroaniline, 1b

^{1}H NMR (500 MHz, $CDCl_3$):

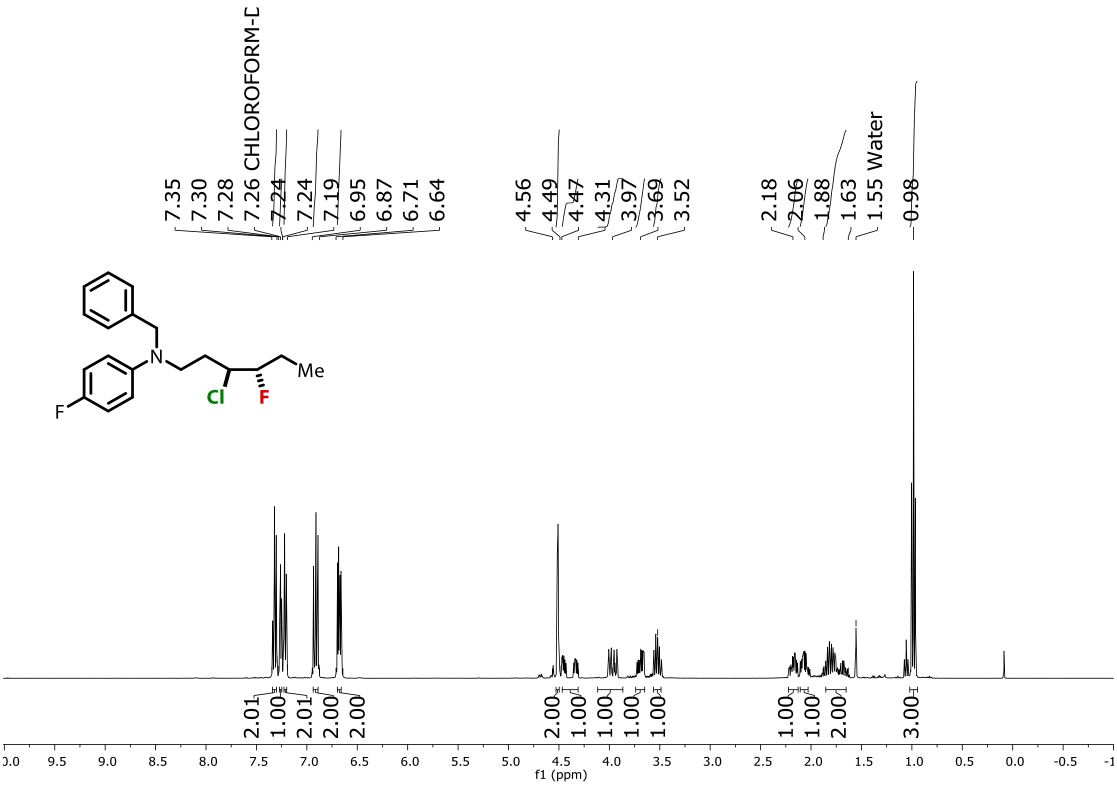

^{19}F NMR (376 MHz, $CDCl_3$):

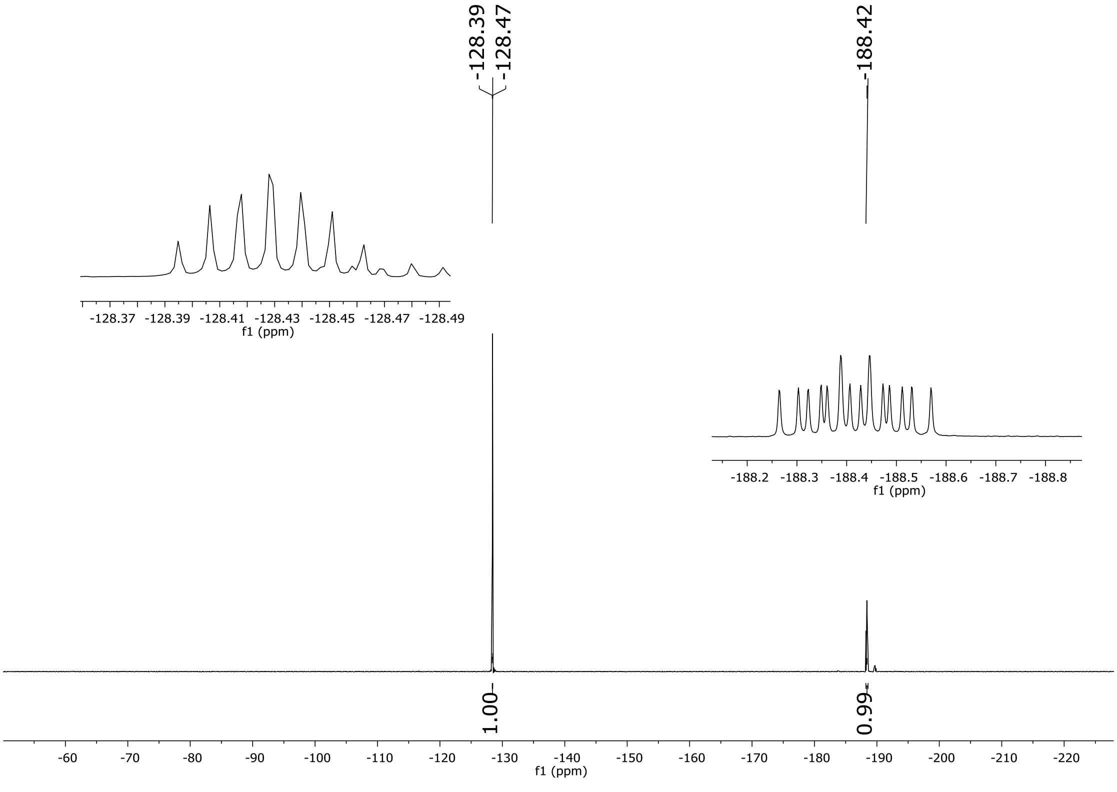

$^{13}C\ \{^{1}H\}$ NMR (125 MHz, $CDCl_3$):

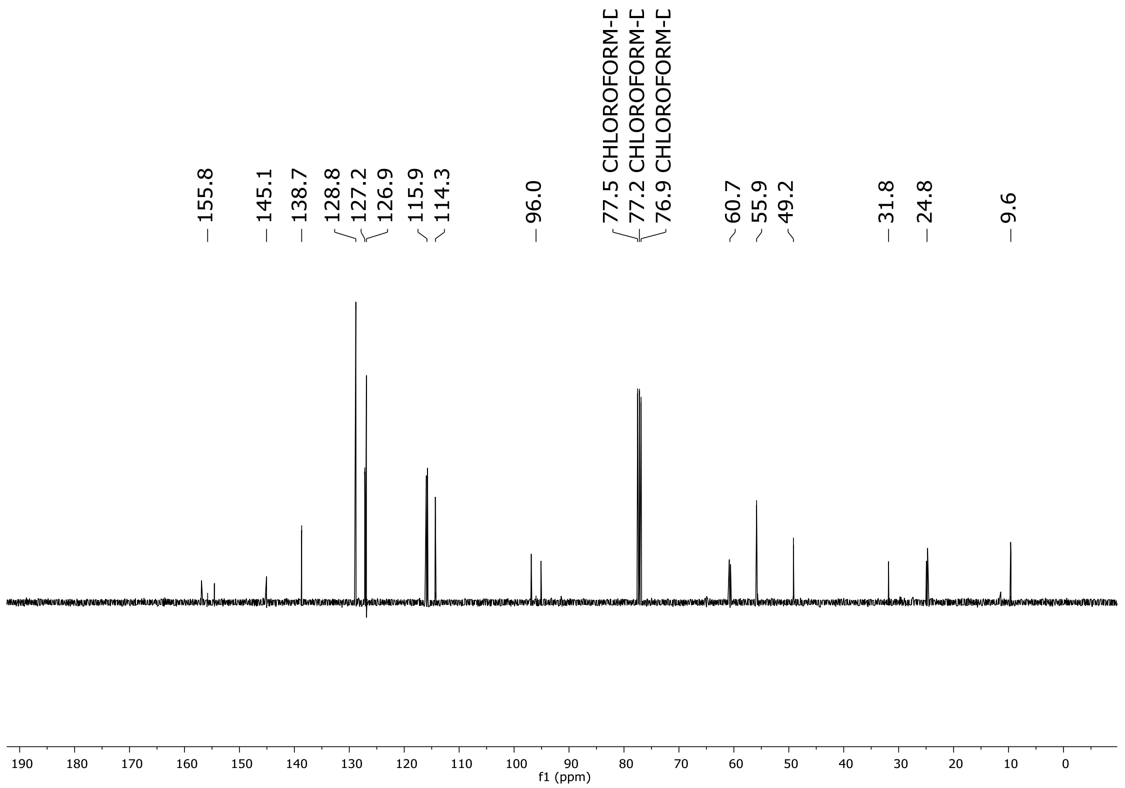

N-benzyl-N-((3S,4S)-3-chloro-4-fluorohexyl)-4-(trifluoromethyl)aniline, 16b

^{1}H NMR (500 MHz, CDCl$_3$):

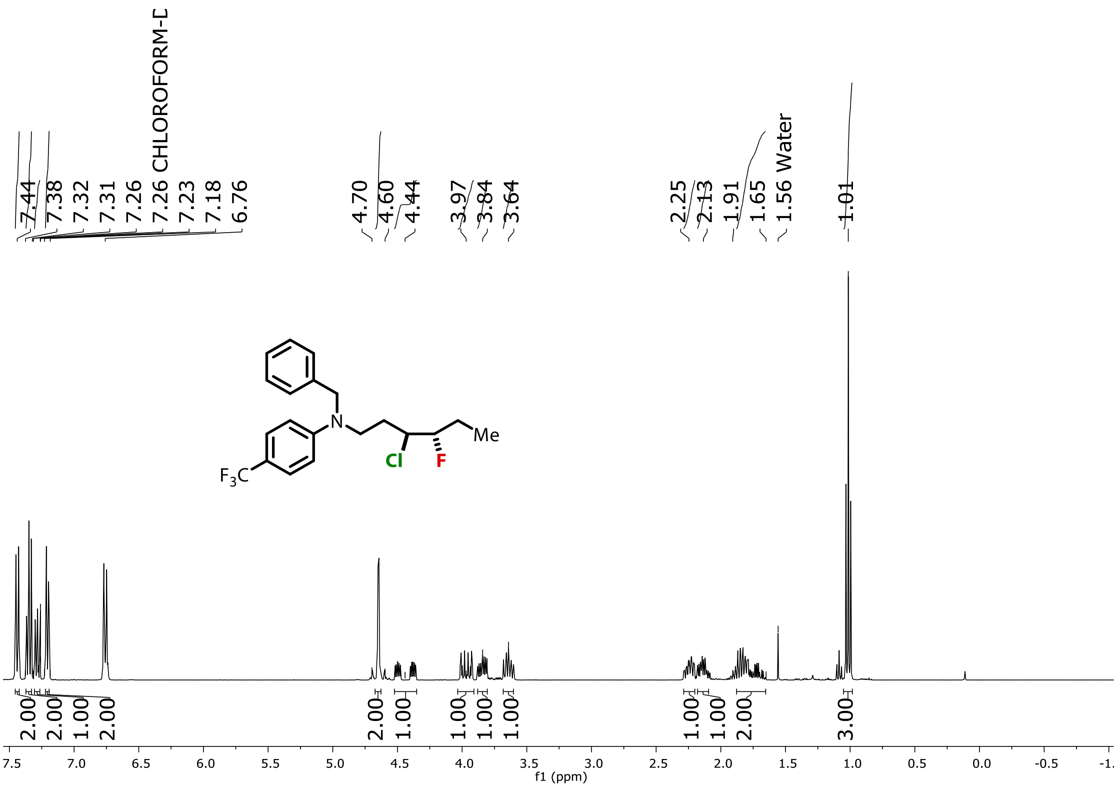

^{19}F NMR (376 MHz, CDCl$_3$):

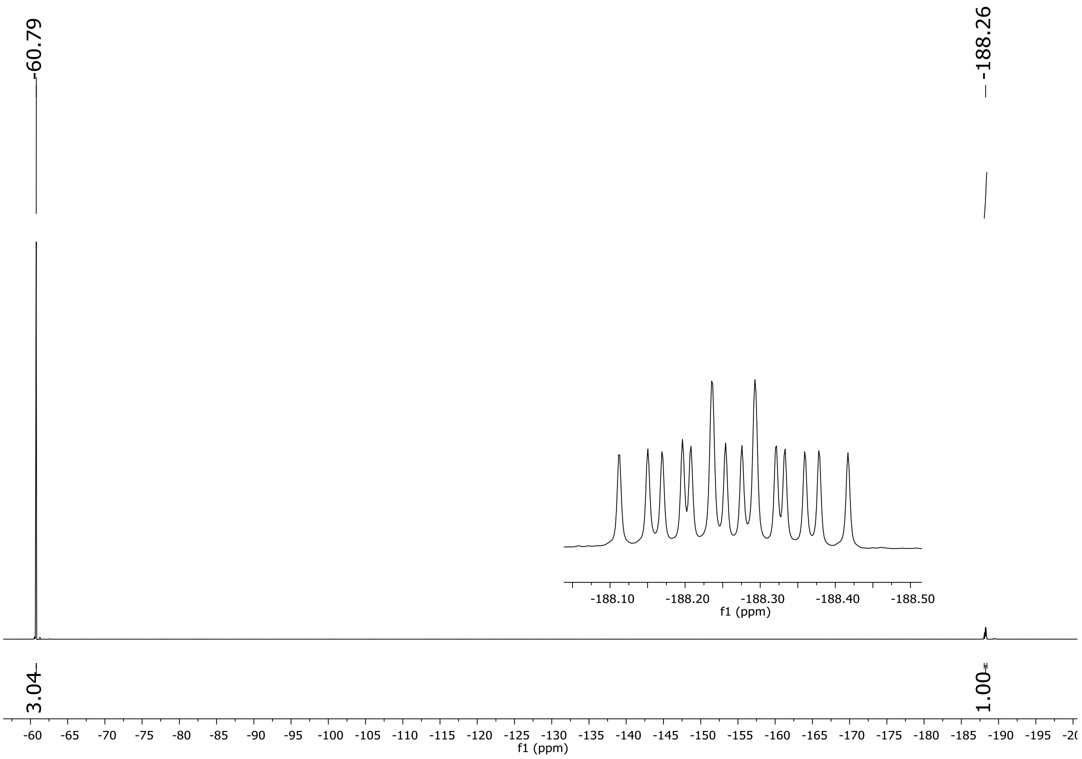

$^{13}C\ \{^{1}H\}$ NMR (125 MHz, CDCl3):

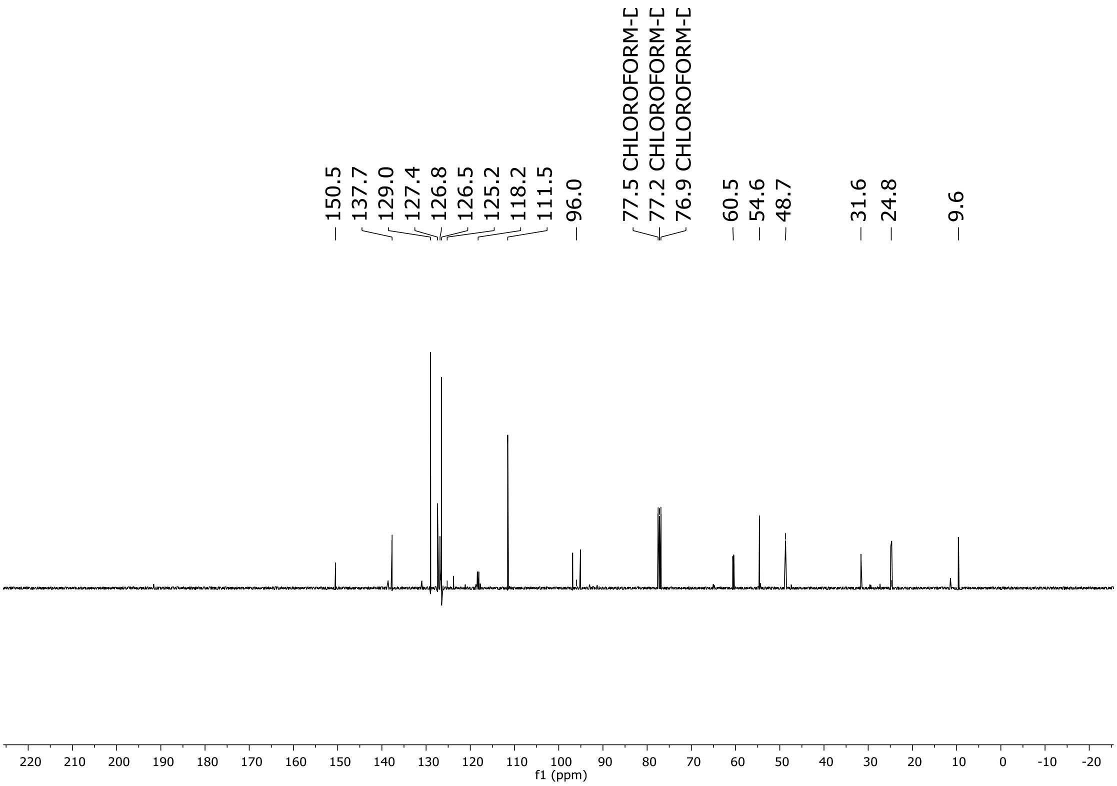

N-benzyl-N-((3S,4S)-3-chloro-4-fluorohexyl)-4-iodoaniline, 17b

^{1}H NMR (400 MHz, CDCl$_3$):

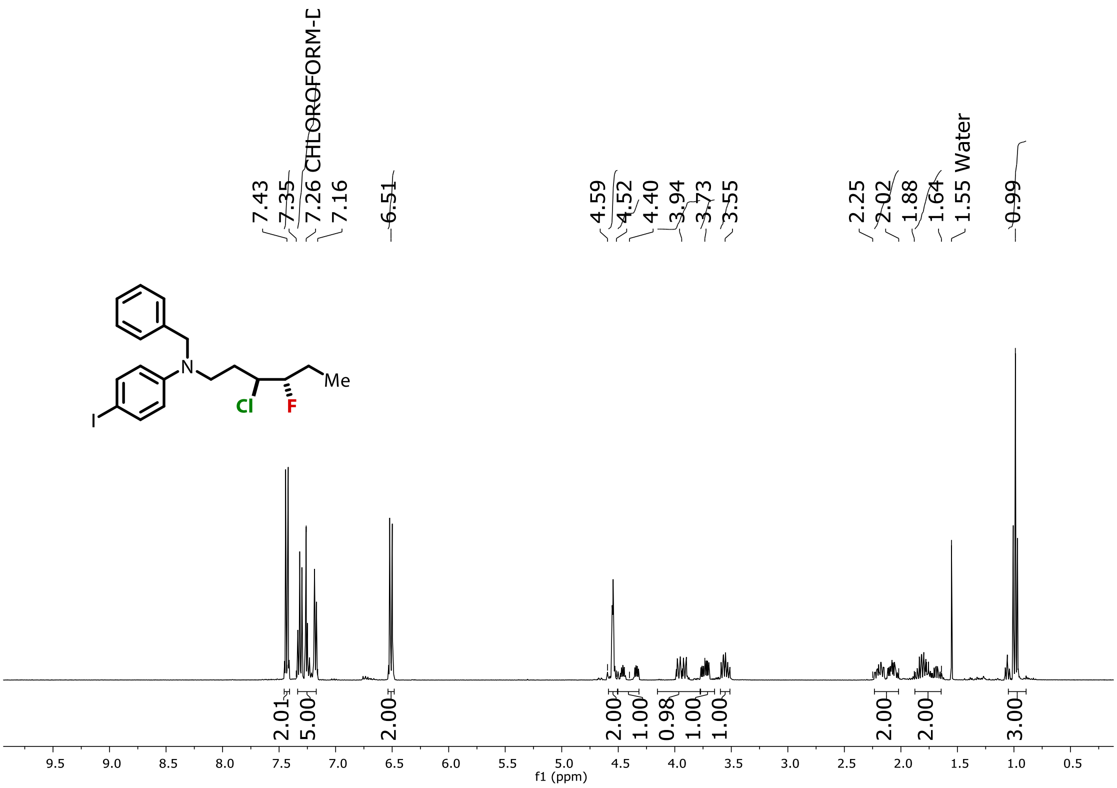

^{19}F NMR (376 MHz, CDCl$_3$):

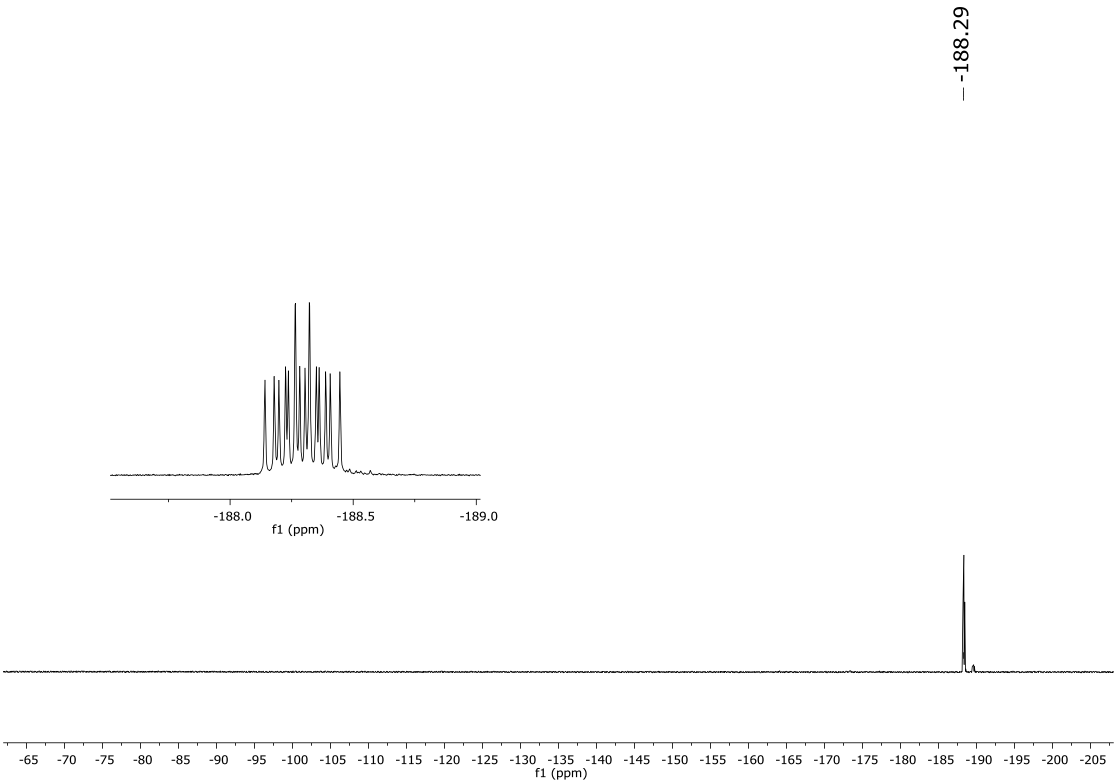

^{13}C {^{1}H} NMR (100 MHz, $CDCl_3$):

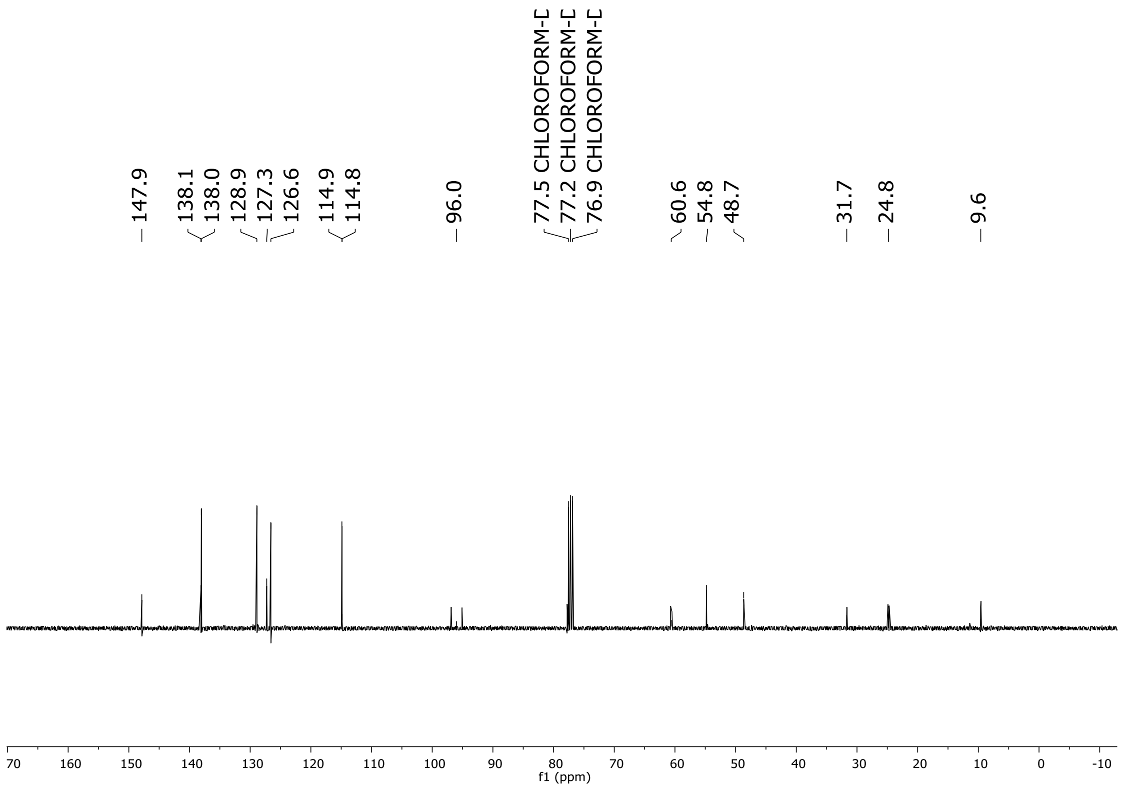

N-benzyl-N-((3S,4S)-3-chloro-4-fluorohexyl)aniline, 18b

^{1}H NMR (400 MHz, $CDCl_3$):

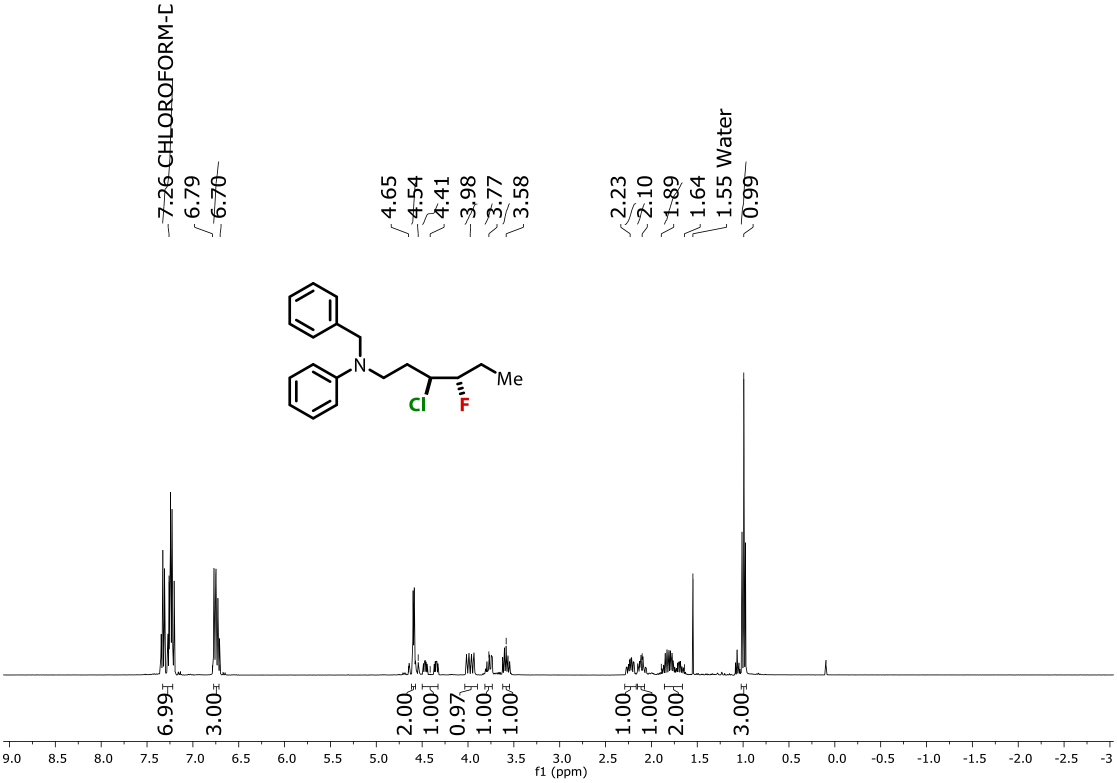

^{19}F NMR (376 MHz, $CDCl_3$):

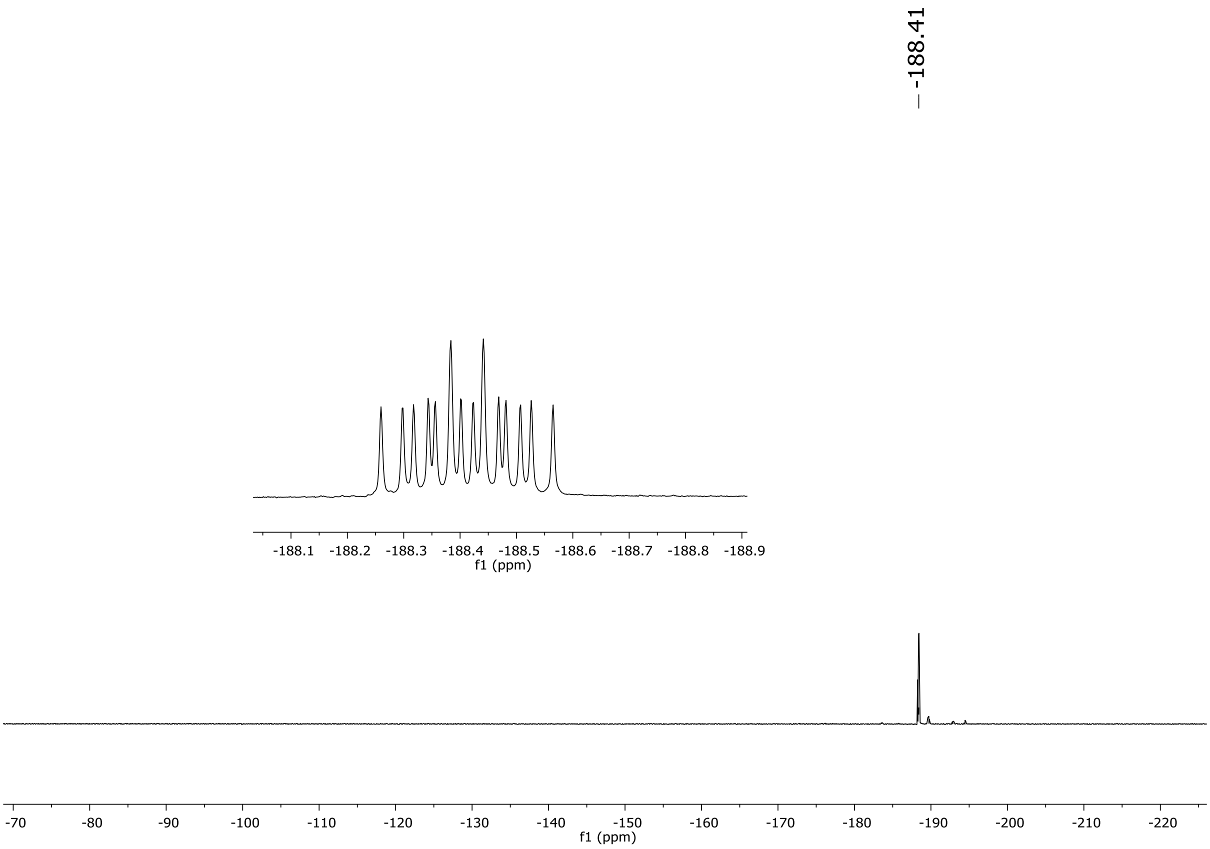

^{13}C {^{1}H} NMR (100 MHz, $CDCl_3$):

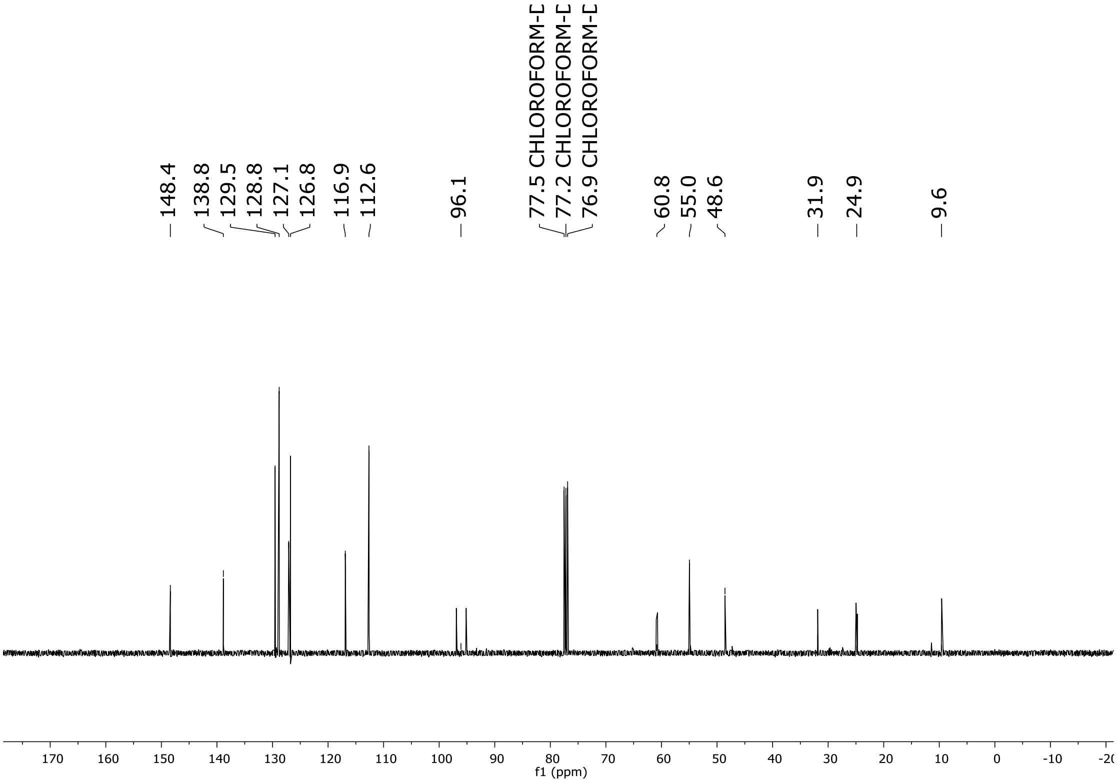

N-benzyl-N-((3S,4S)-3-chloro-4-fluorohexyl)-4-methylaniline, 19b

^{1}H NMR (500 MHz, $CDCl_3$):

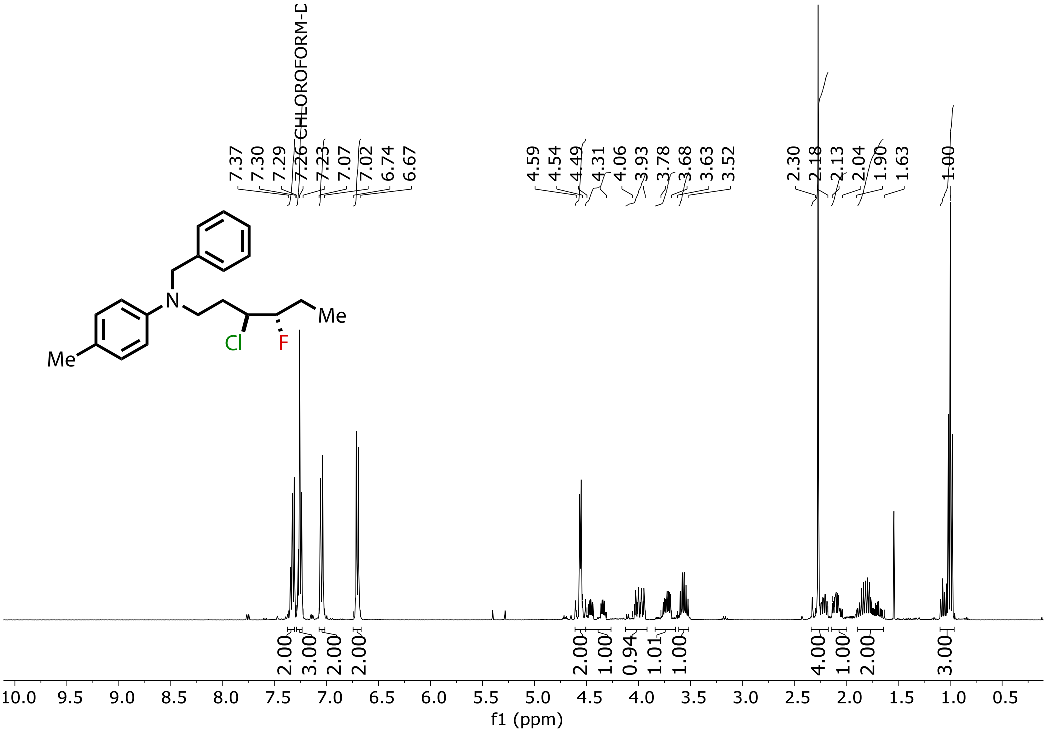

^{19}F NMR (376 MHz, $CDCl_3$):

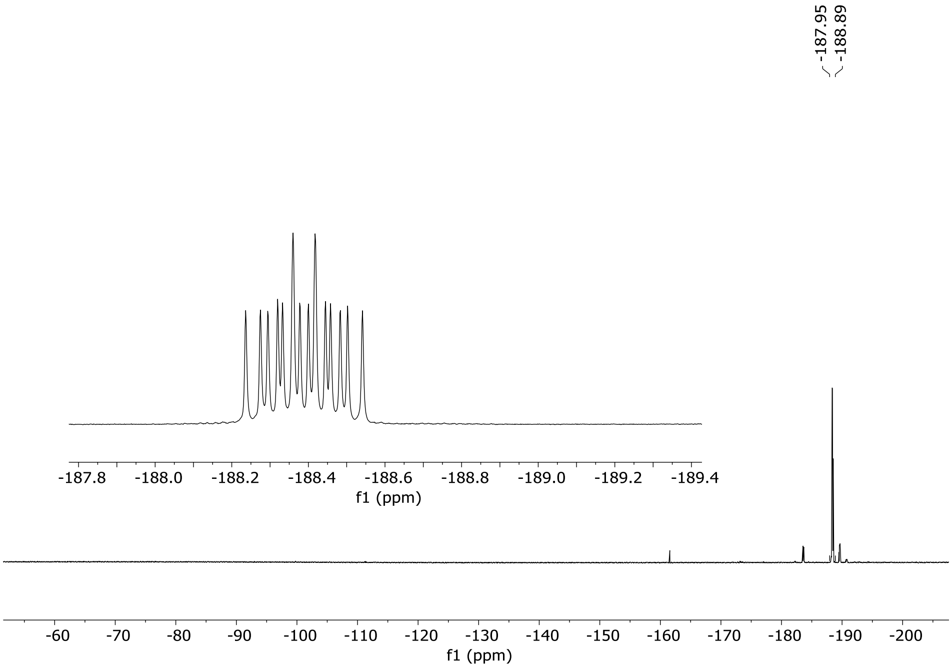

^{13}C {^{1}H} NMR (125 MHz, $CDCl_3$):

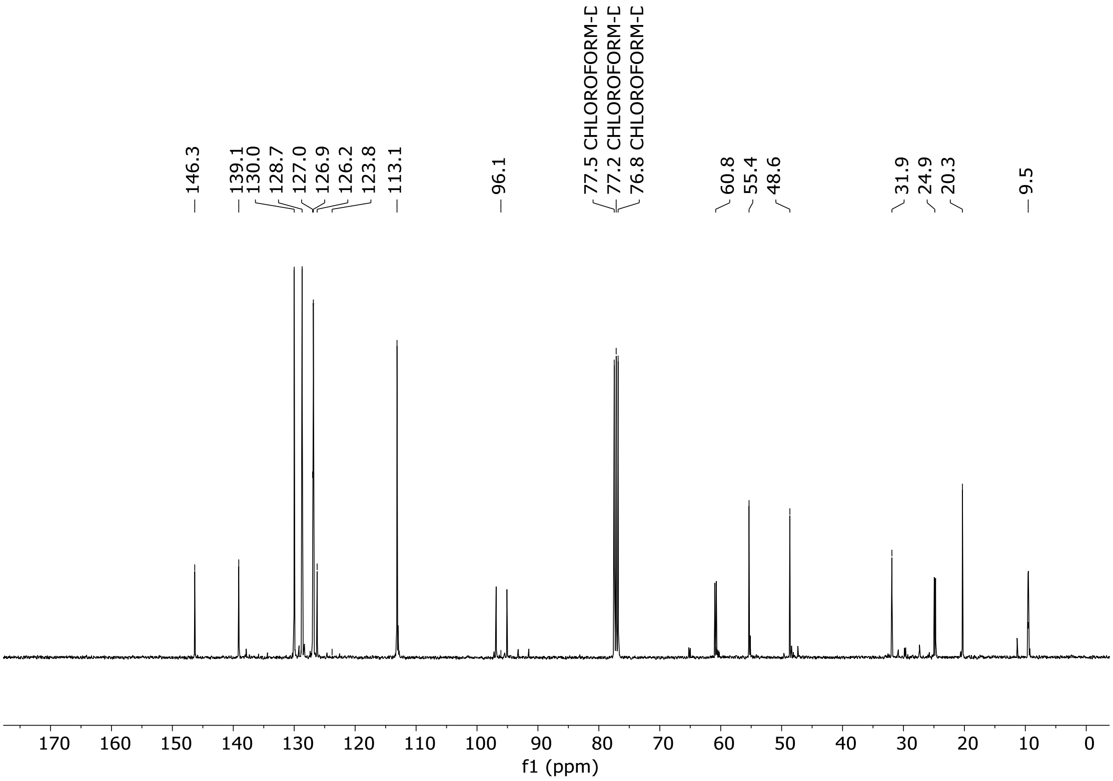
146.3
139.1
130.0
128.7
127.0
126.9
126.2
123.8
113.1
96.1
77.5 CHLOROFORM-D
77.2 CHLOROFORM-D
76.8 CHLOROFORM-D
60.8
55.4
48.6
31.9
24.9
20.3
9.5
170 160 150 140 130 120 110 100 90 80 70 60 50 40 30 20 10 0
f1 (ppm)

N-((3S,4R)-3-chloro-4-fluorohexyl)-4-fluoroaniline, 20d

^{1}H NMR (500 MHz, CDCl$_3$):

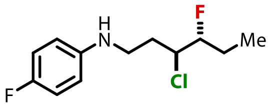

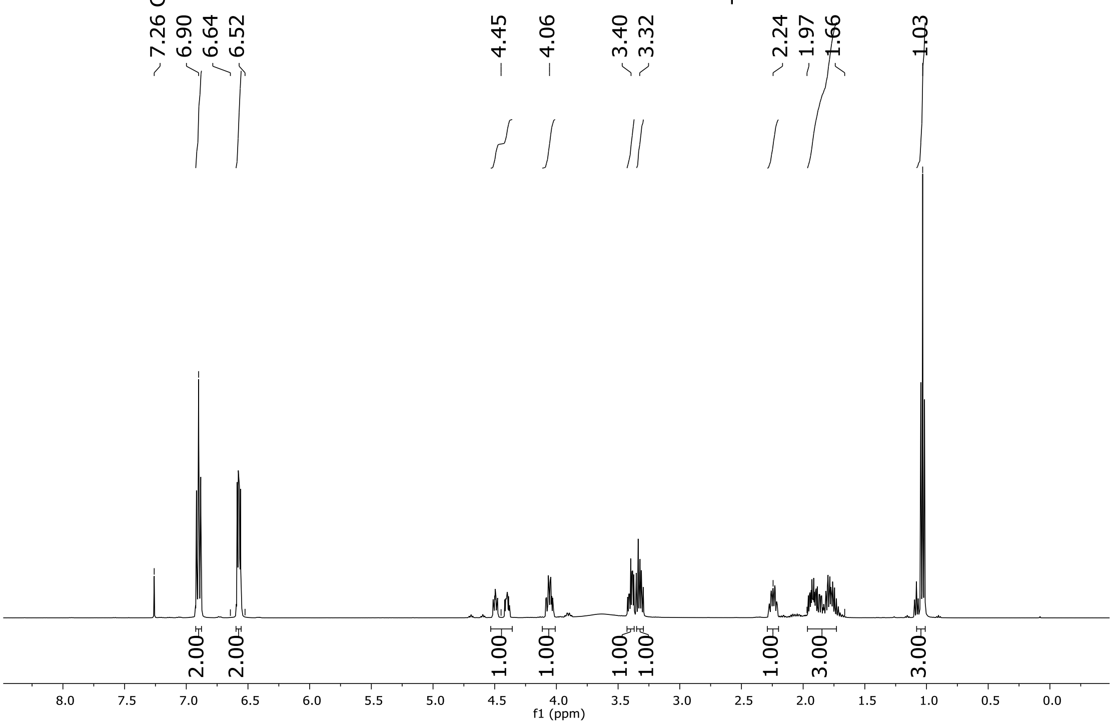

^{19}F NMR (376 MHz, CDCl$_3$):

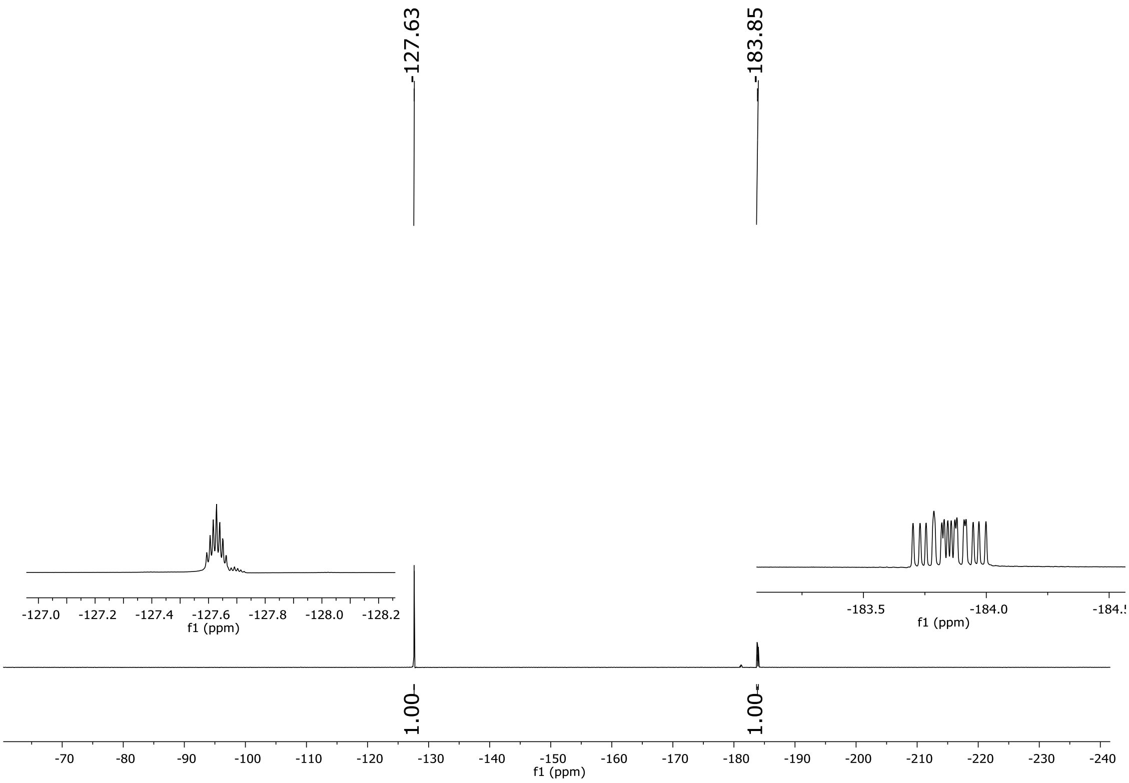

$^{13}C\ \{^{1}H\}$ NMR (125 MHz, $CDCl_3$):

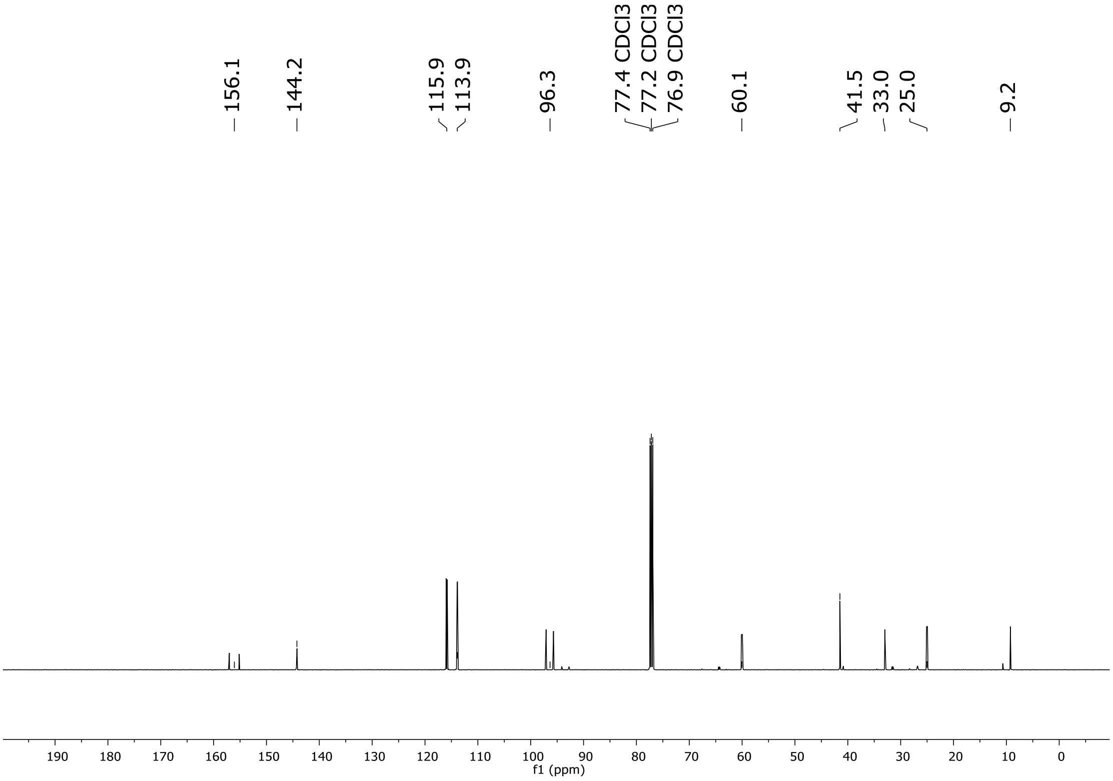

N-benzyl-N-((3S,4R)-3-chloro-4-fluorohexyl)-4-fluoroaniline, 21d

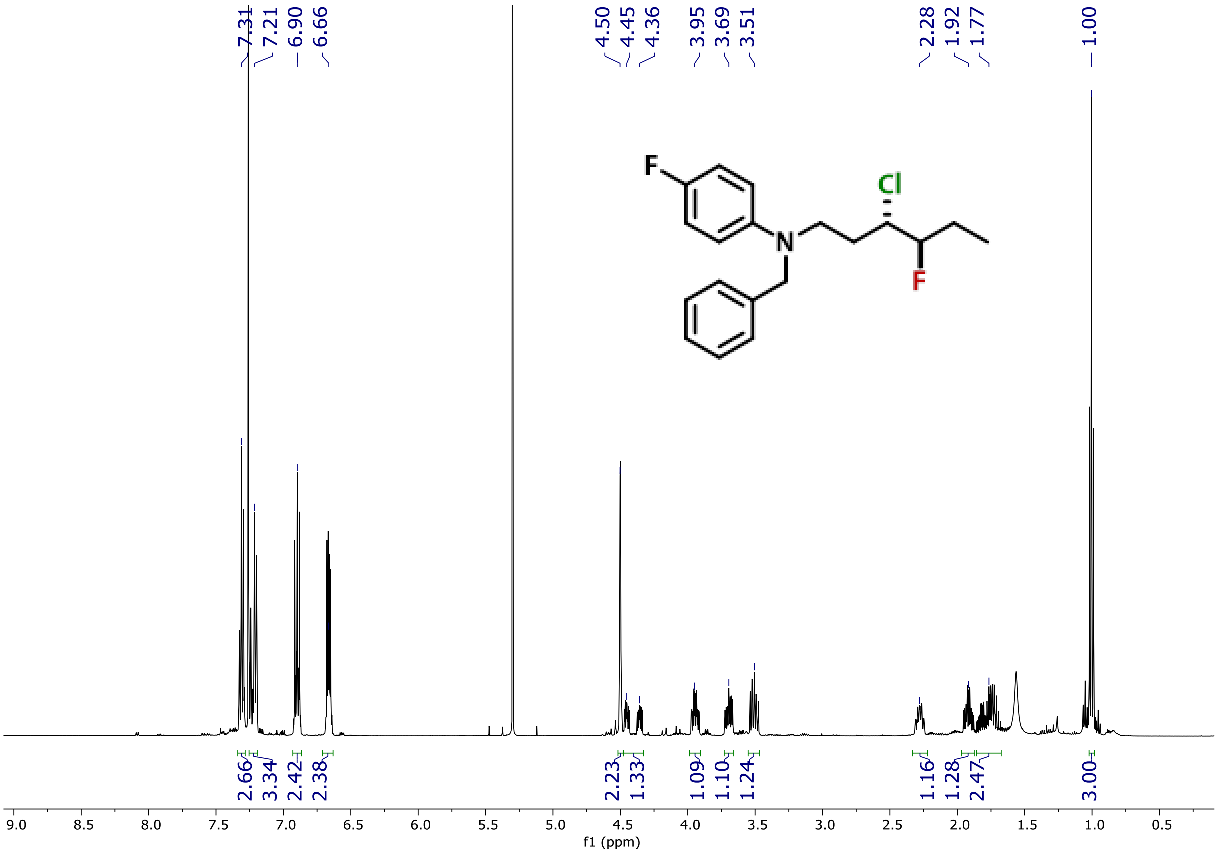

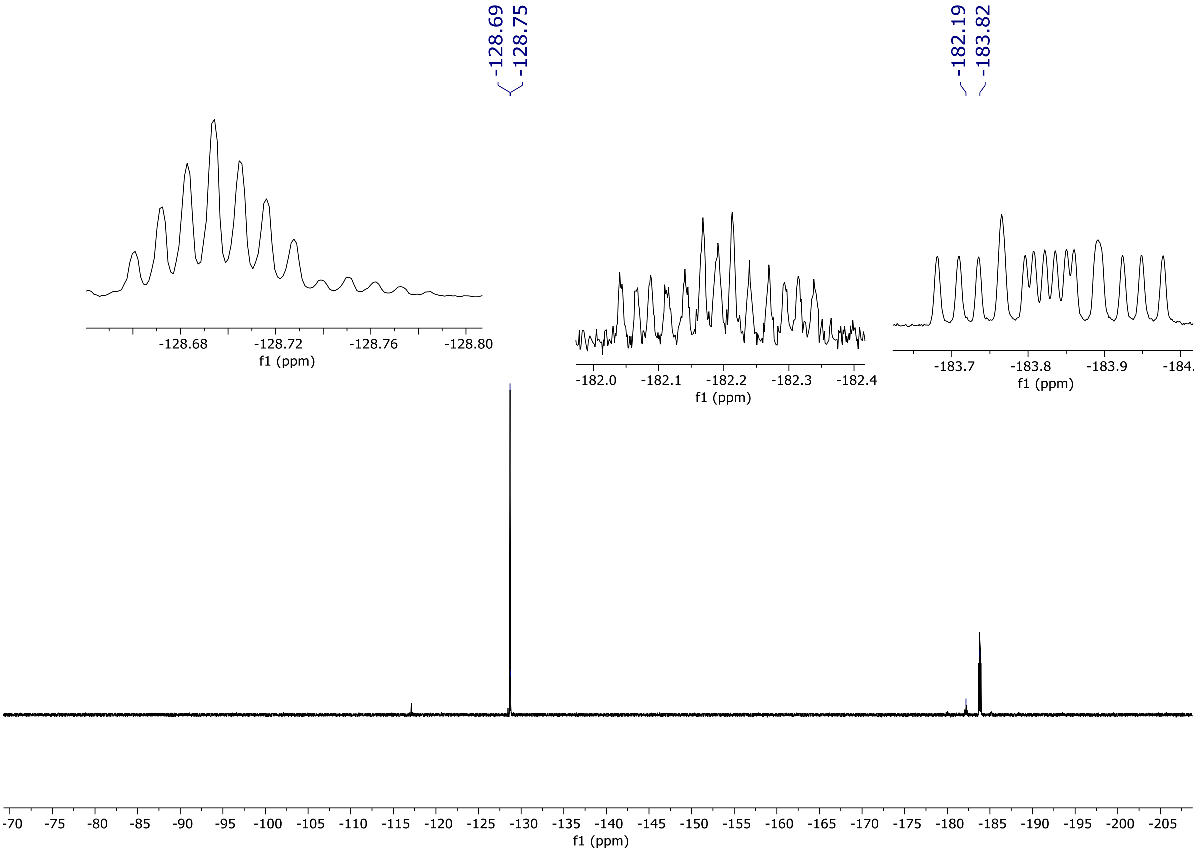

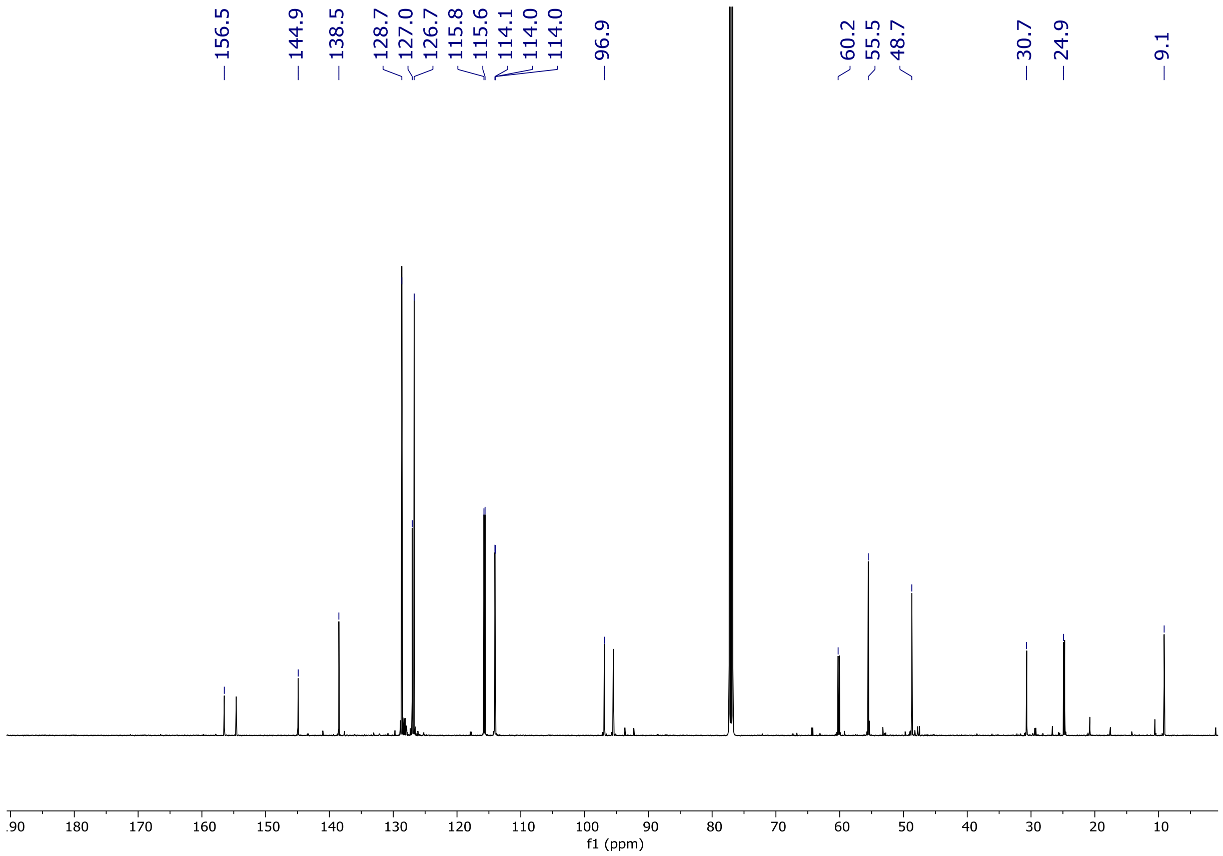
156.5
144.9
138.5
128.7
127.0
126.7
115.8
115.6
114.1
114.0
114.0
96.9
60.2
55.5
48.7
30.7
24.9
9.1
190
180
170
160
150
140
130
120
110
100
90
80
70
60
50
40
30
20
10
f1 (ppm)

(3R,4R)-N-benzhydryl-N-benzyl-3-chloro-4-fluorohexan-1-amine, 22b

^{1}H NMR (400 MHz, $CDCl_3$):

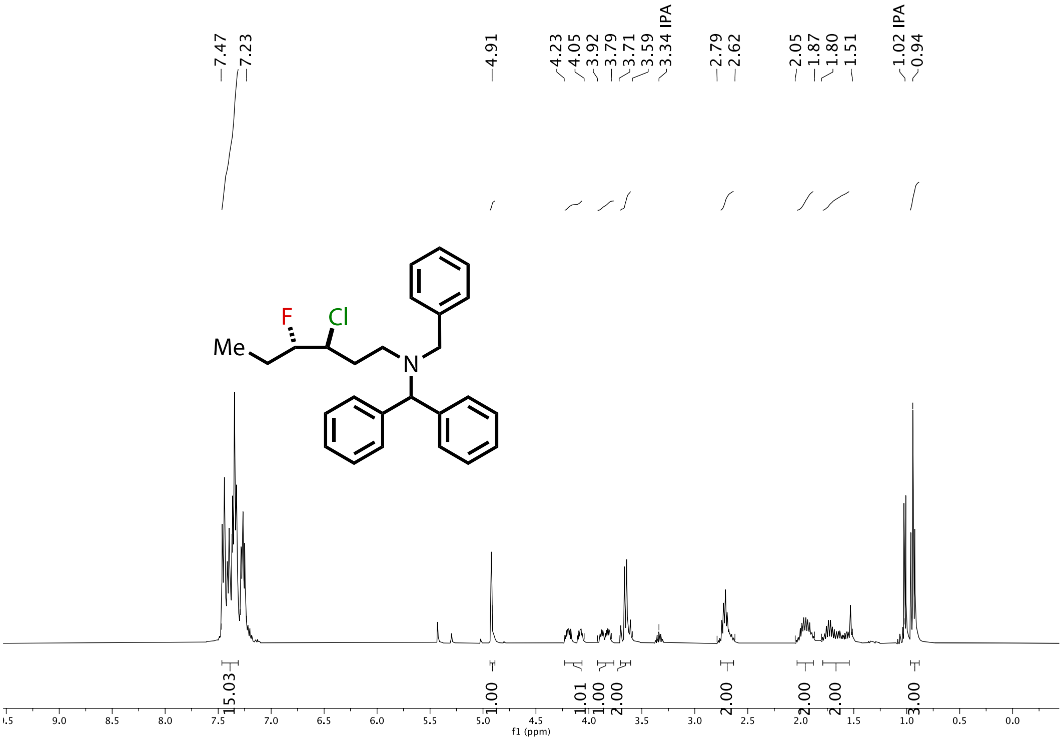

^{13}C NMR (100 MHz, $CDCl_3$):

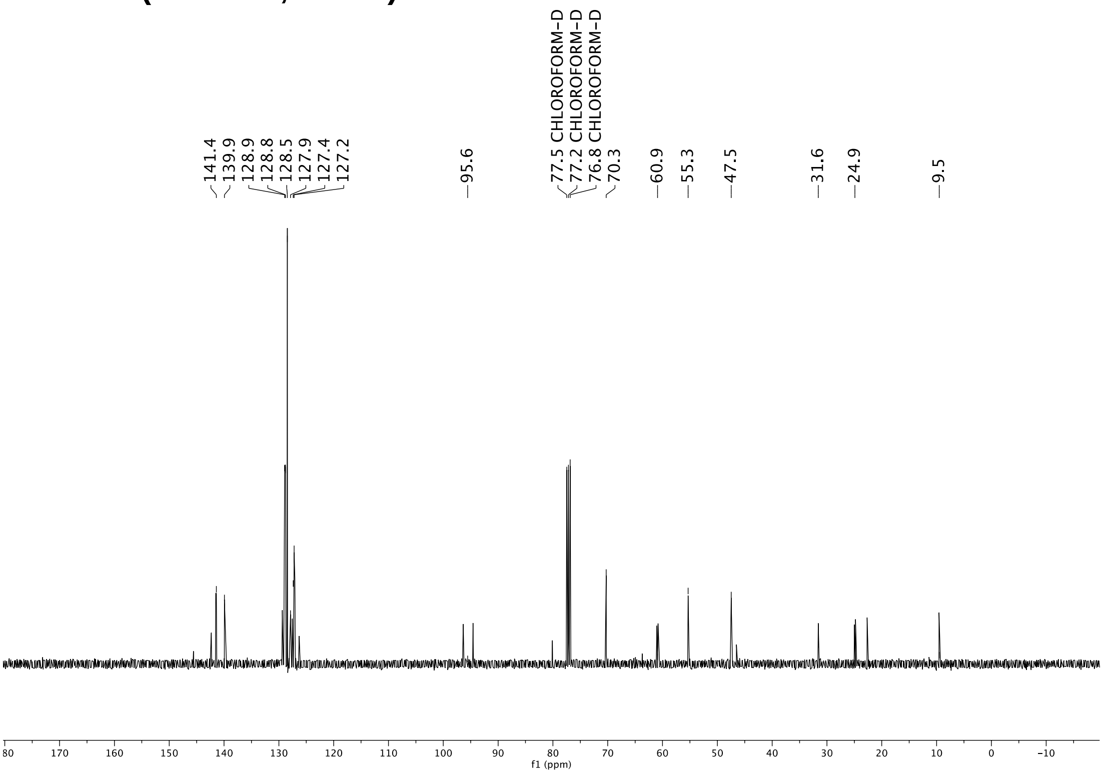

^{19}F NMR (376 MHz, $CDCl_3$):

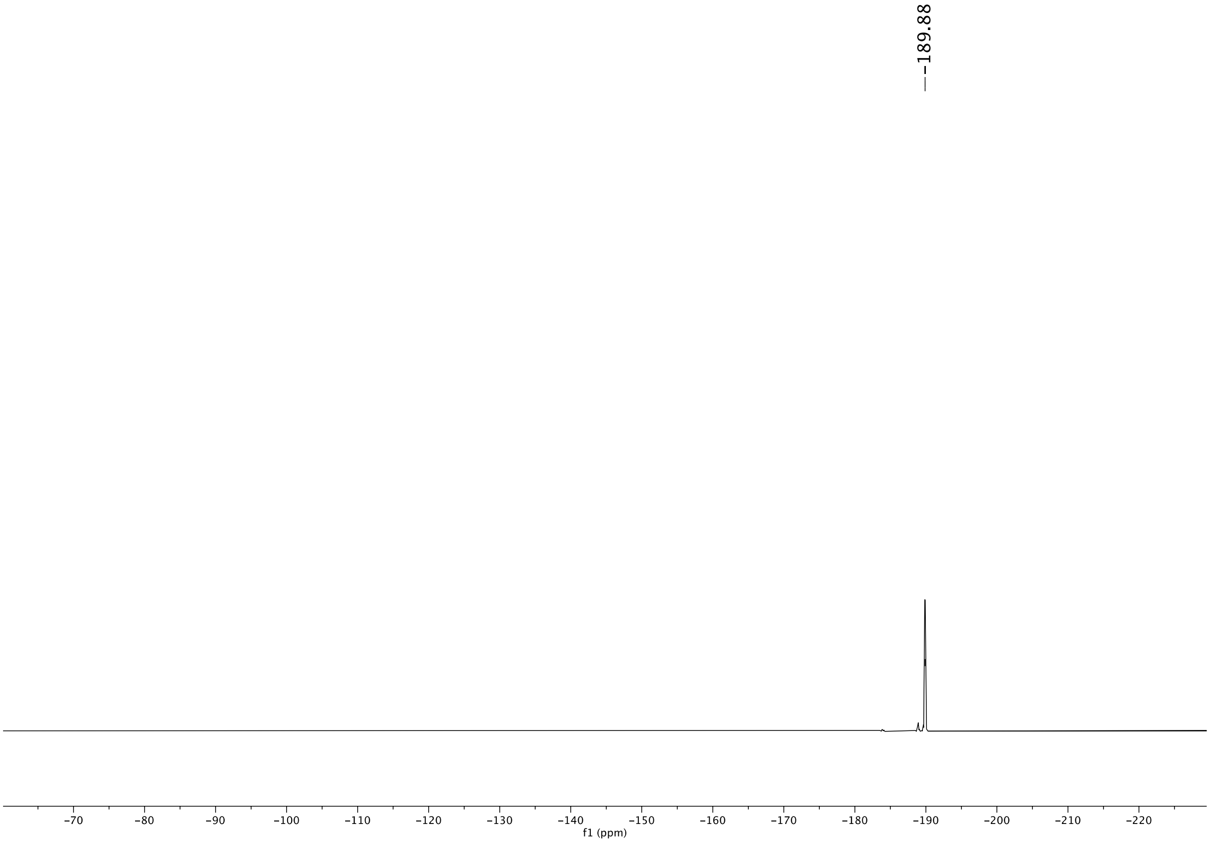

4-(trifluoromethyl)phenyl (9R,10S)-9-chloro-10-fluorooctadecanoate, 23d

^{1}H NMR (400 MHz, $CDCl_3$):

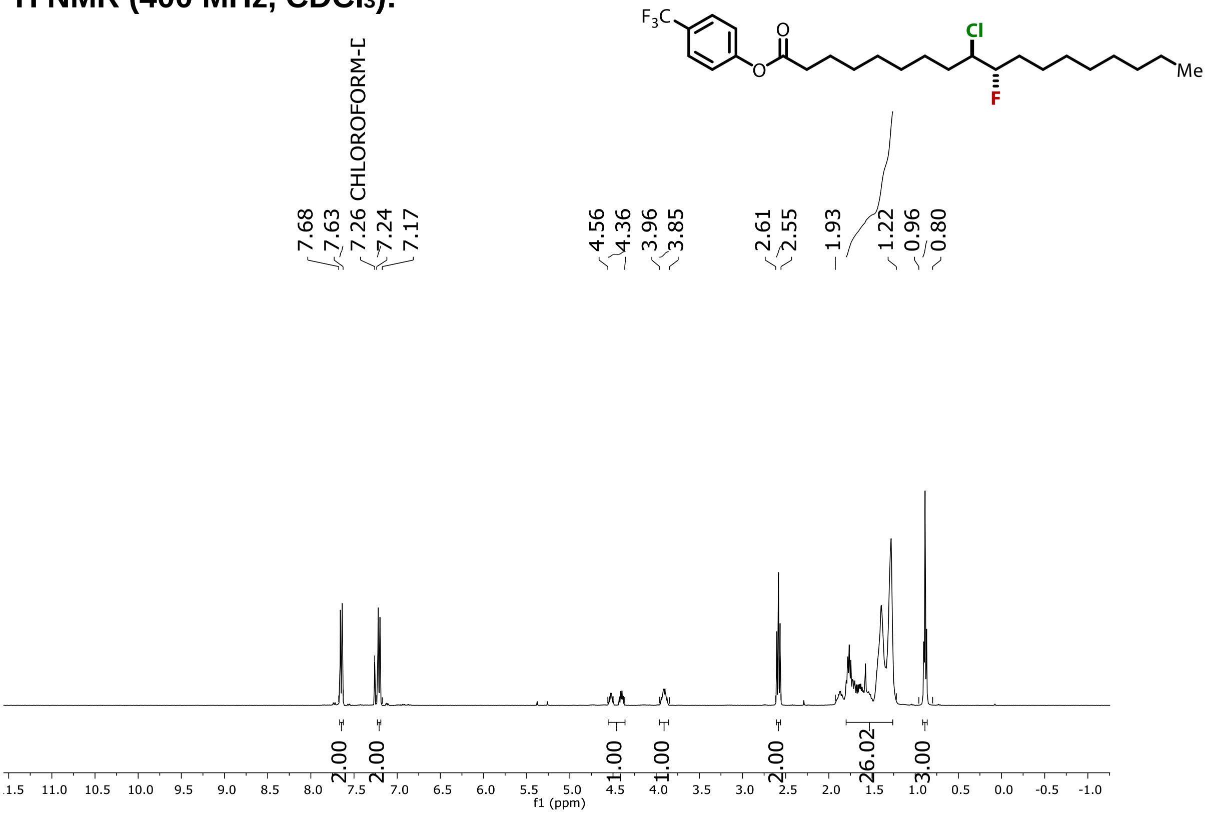

^{19}F NMR (376 MHz, $CDCl_3$):

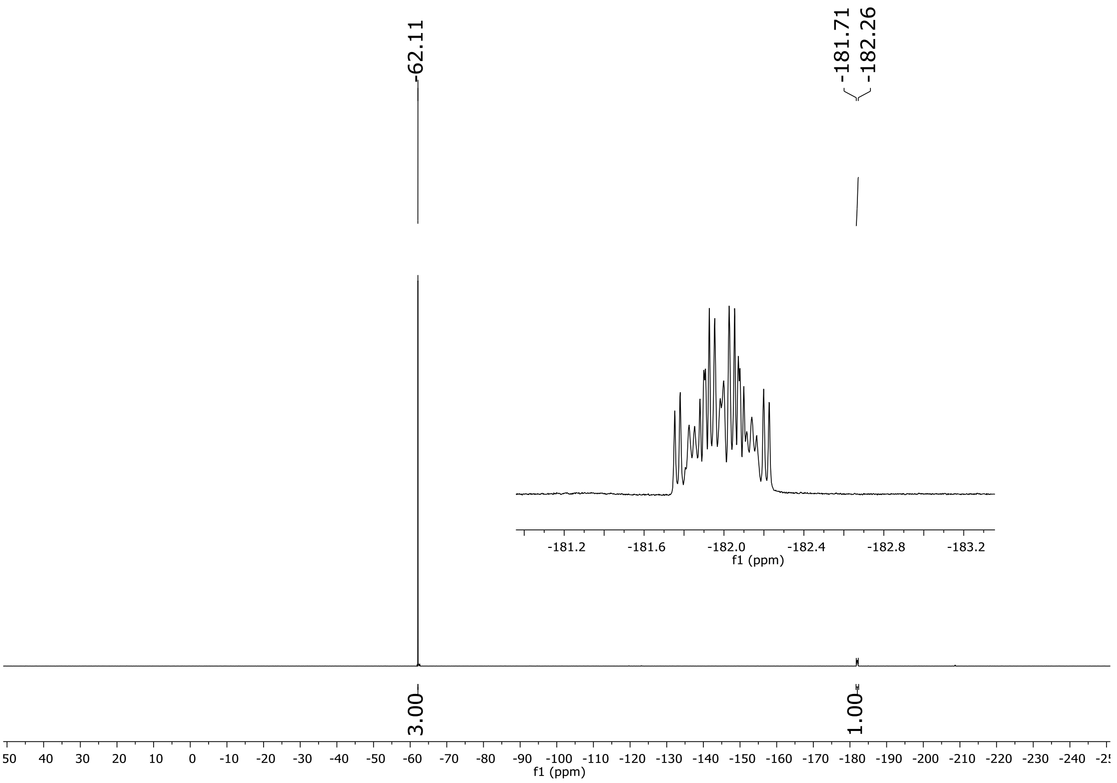

Back to top

$^{13}C\ \{^{1}H\}$ NMR (100 MHz, $CDCl_3$):

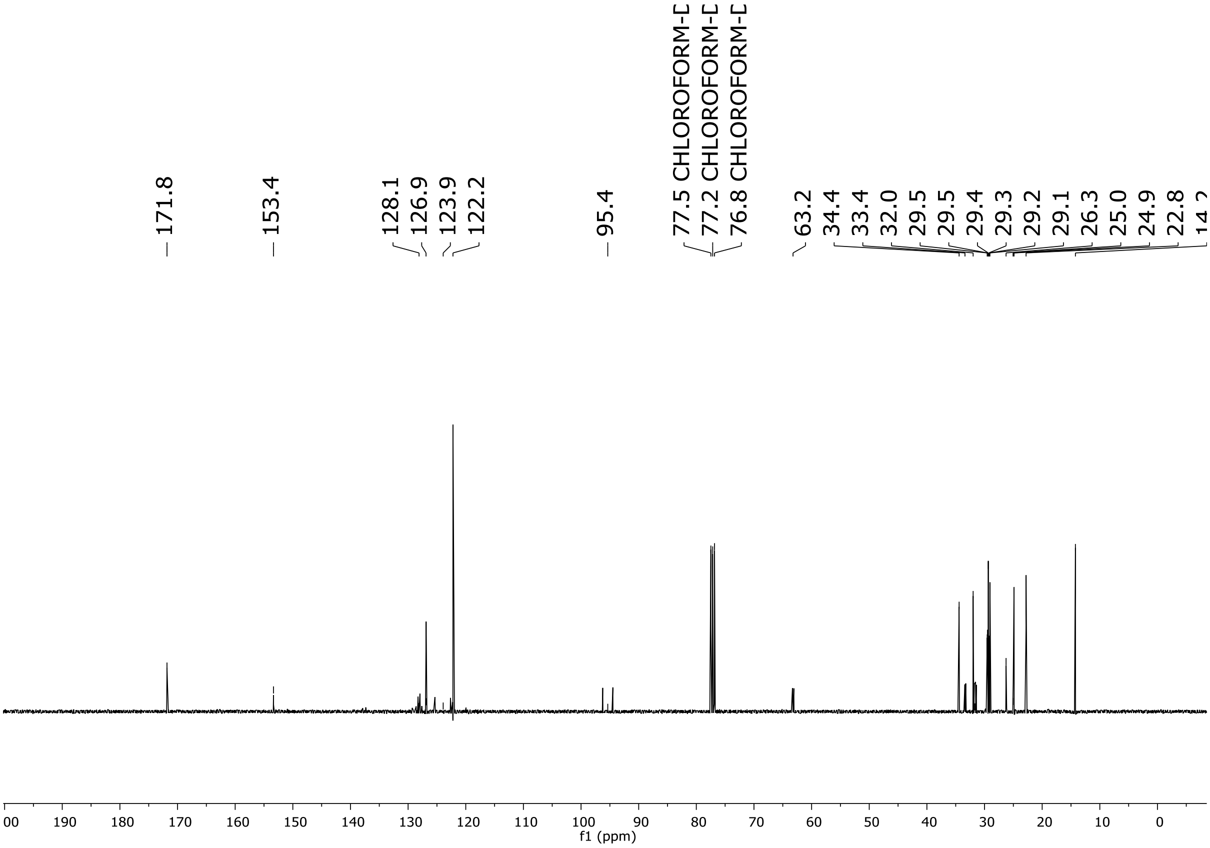

(R)-4-(2-chloro-1-fluoro-2-methylpropyl)-5-methylthiazole, 24b

^{1}H NMR (500 MHz, $CDCl_3$):

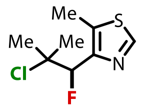

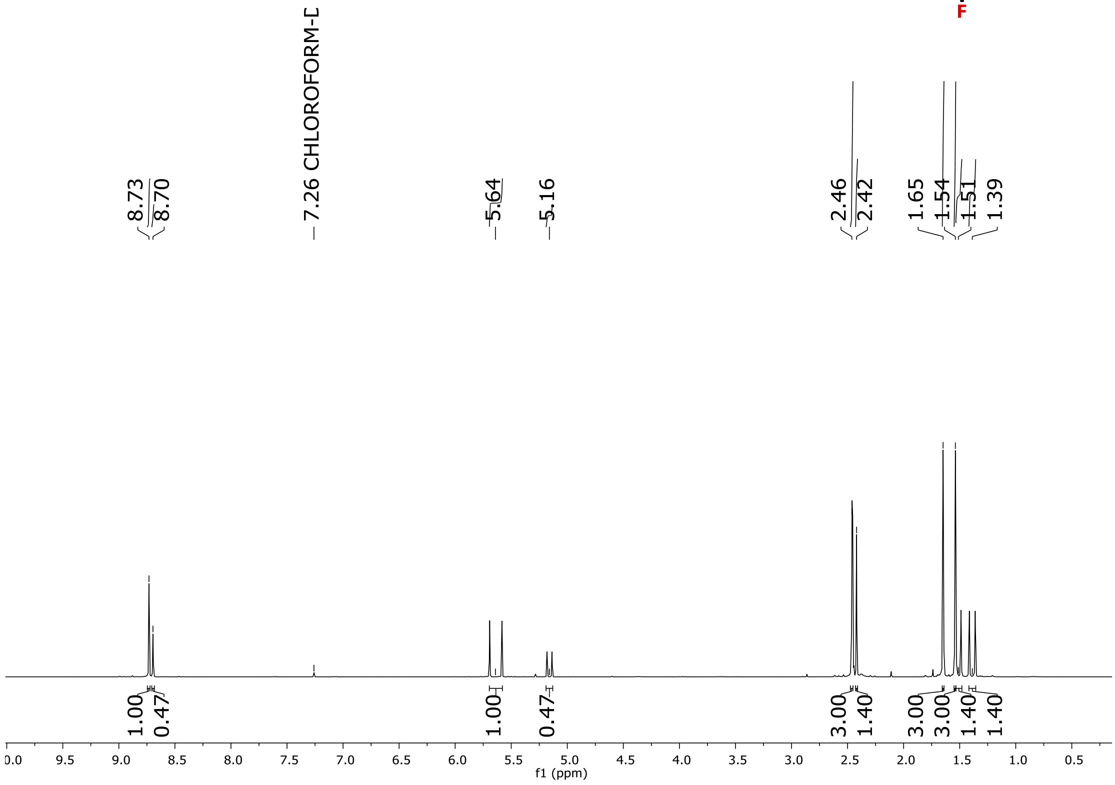

^{19}F NMR (376 MHz, $CDCl_3$):

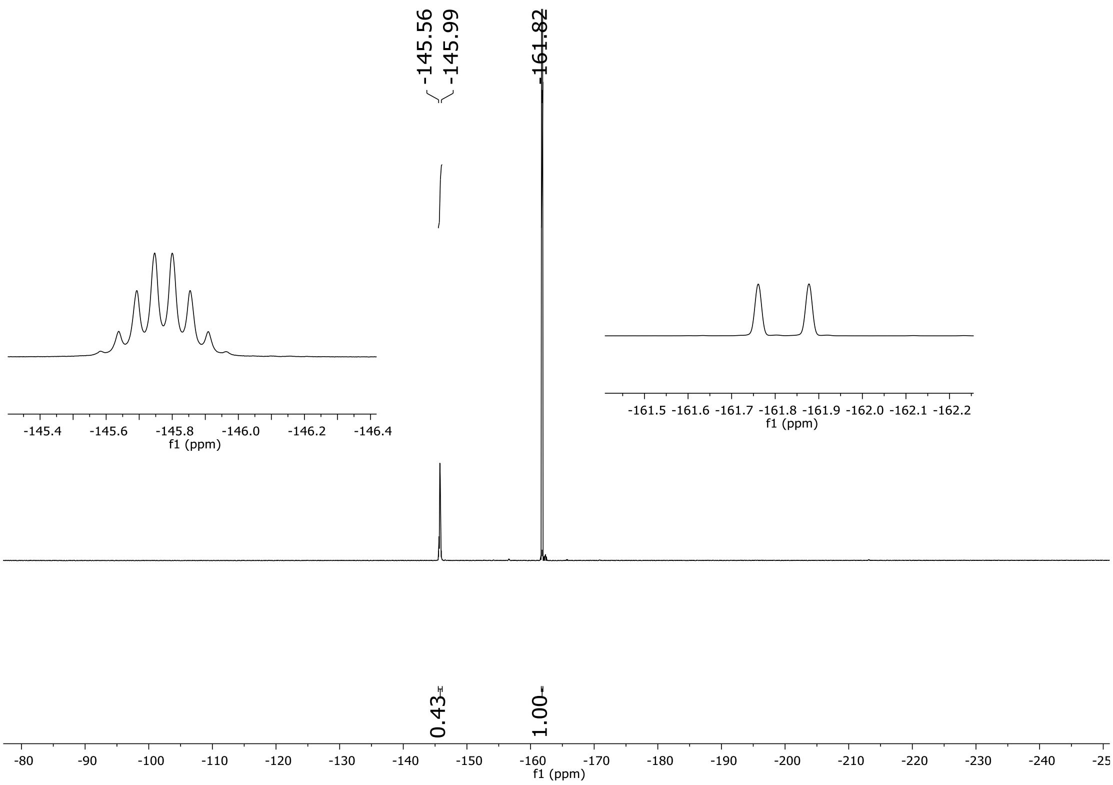

^{13}C {^{1}H} NMR (125 MHz, $CDCl_3$):

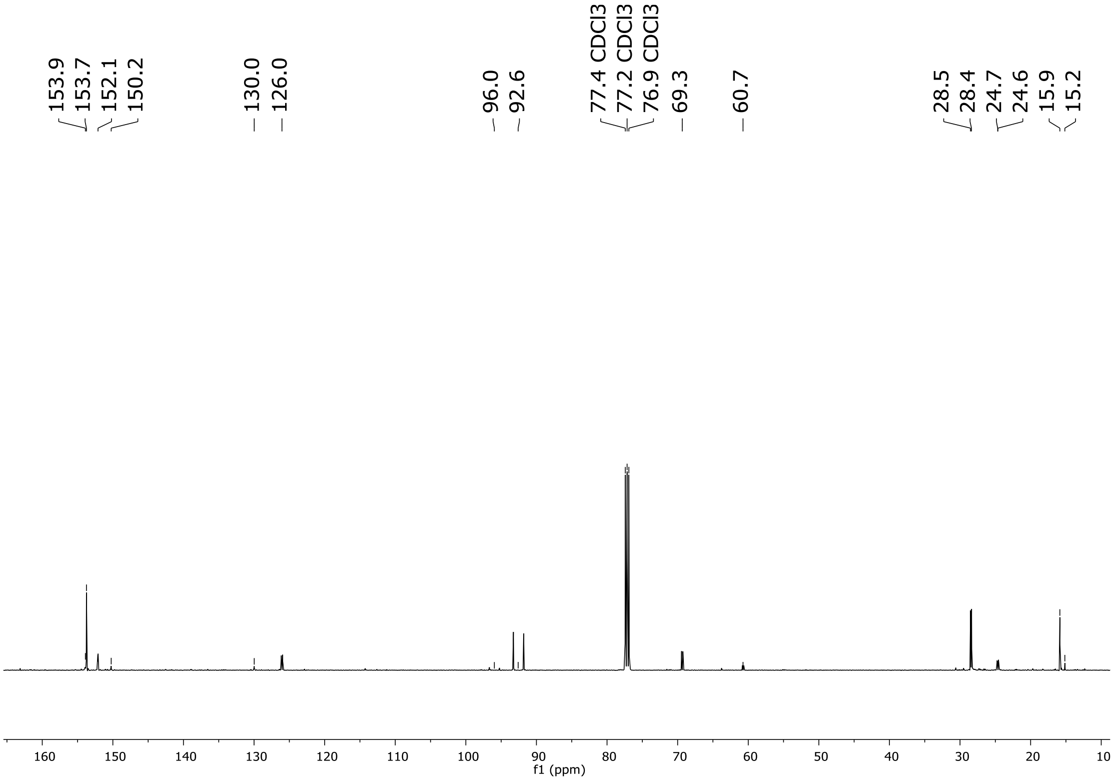

(2S,3S)-N,N-dibenzyl-2-chloro-3-fluorohexan-1-amine, 25b

^{1}H NMR (400 MHz, $CDCl_3$):

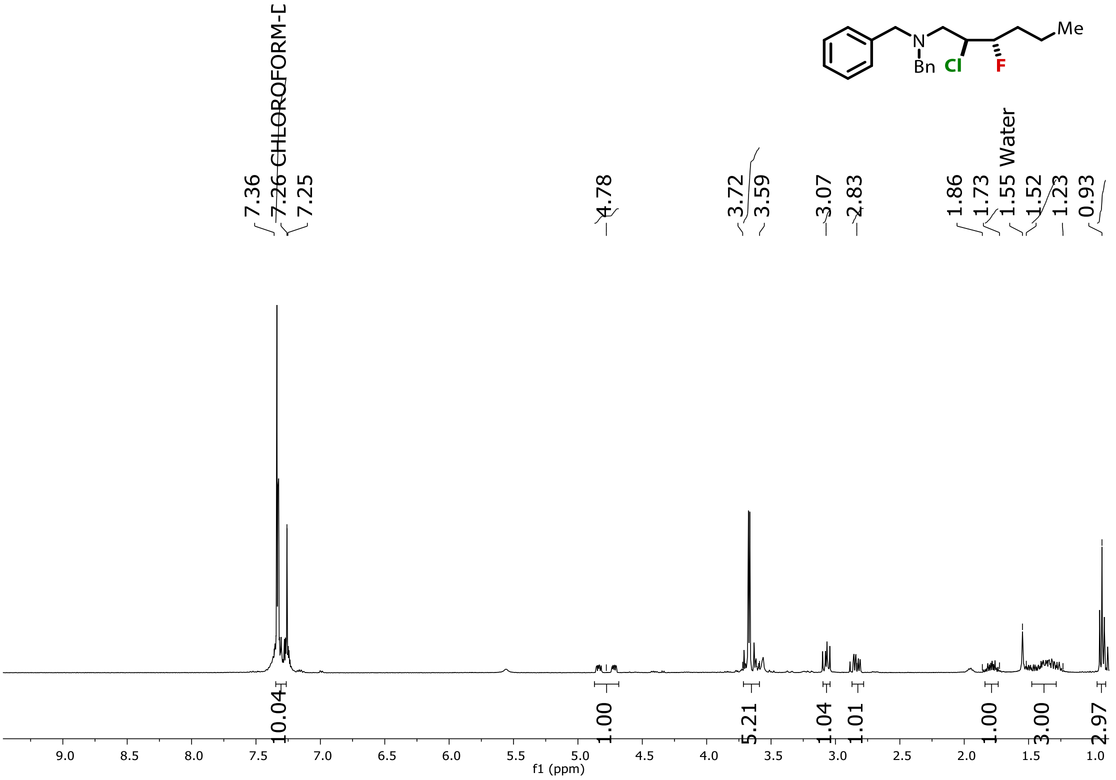

^{19}F NMR (376 MHz, $CDCl_3$):

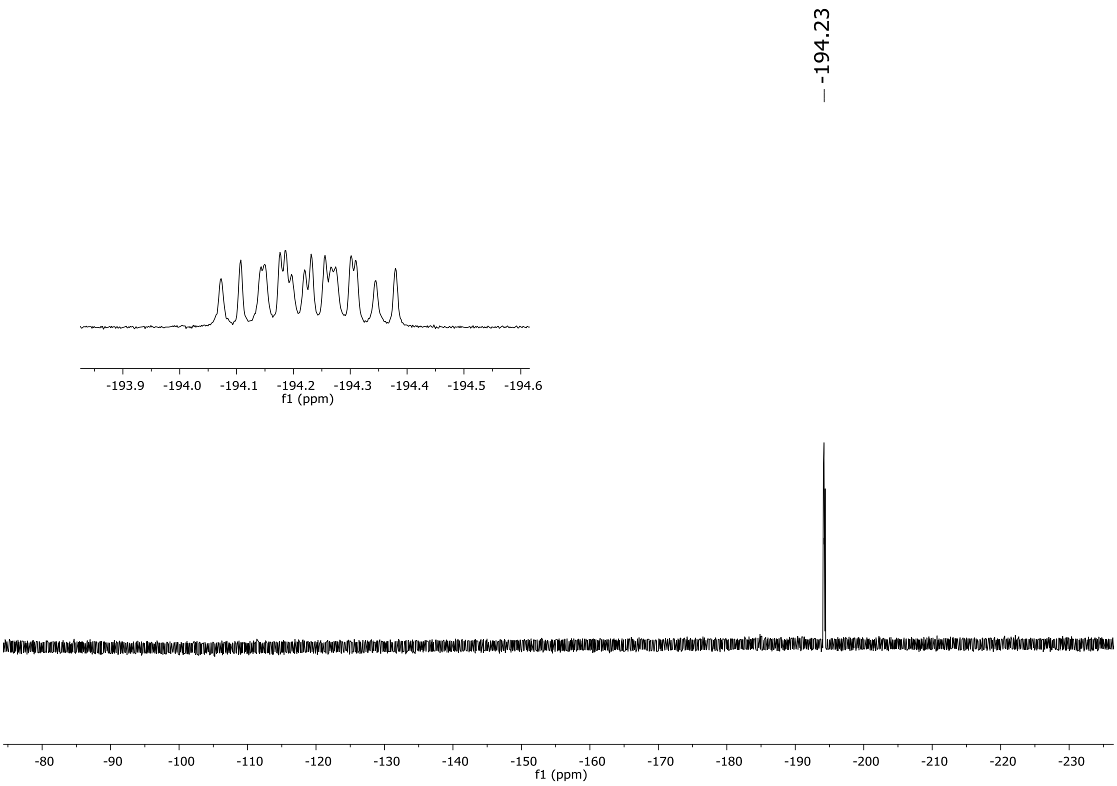

$^{13}C\{^{1}H\}$ NMR (100 MHz, $CDCl_3$):

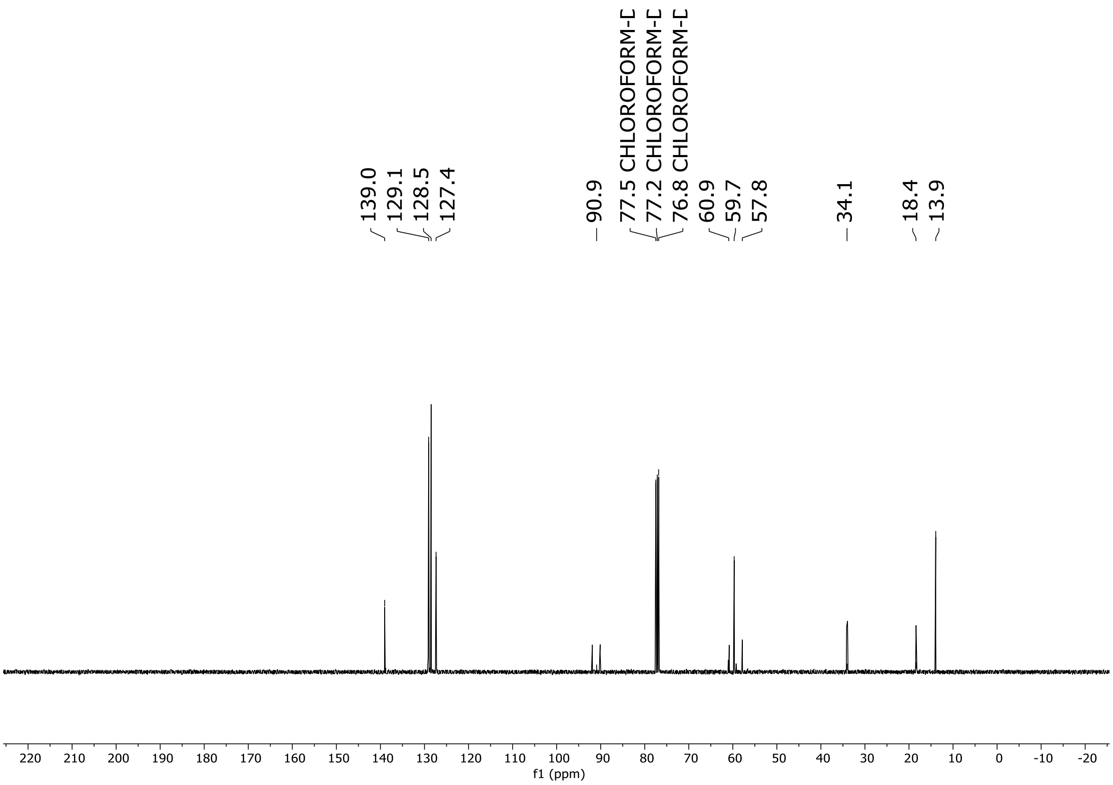

N,N-dibenzyl-3-chloro-4-fluorohexan-1-amine, 26b

^{1}H NMR (400 MHz, $CDCl_3$):

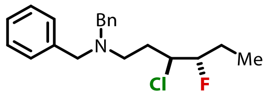

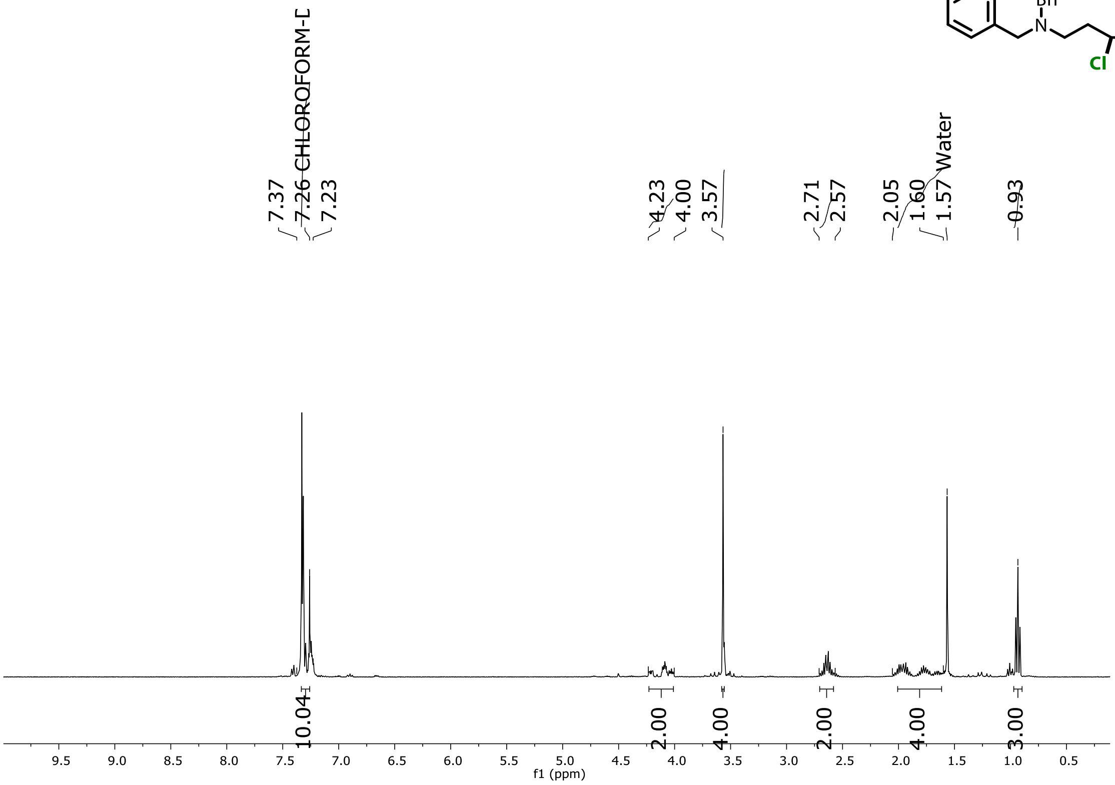

^{19}F NMR (376 MHz, $CDCl_3$):

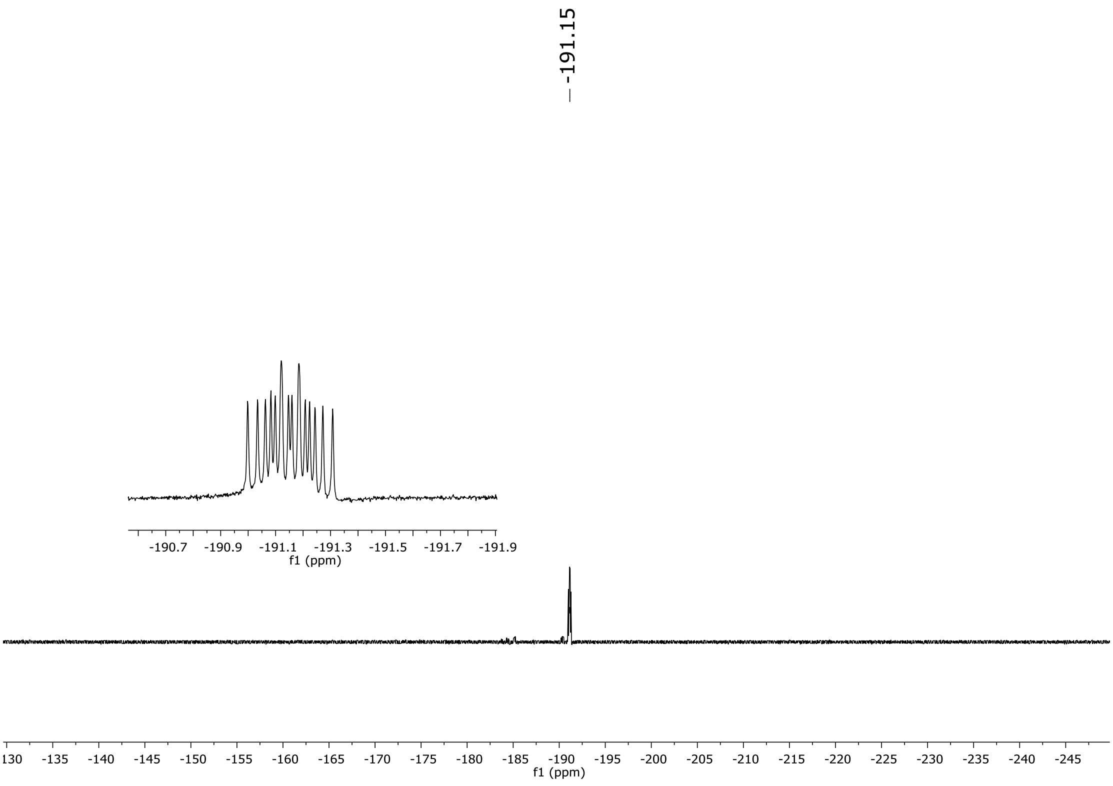

Back to top

$^{13}C\ \{^{1}H\}$ NMR (100 MHz, $CDCl_3$):

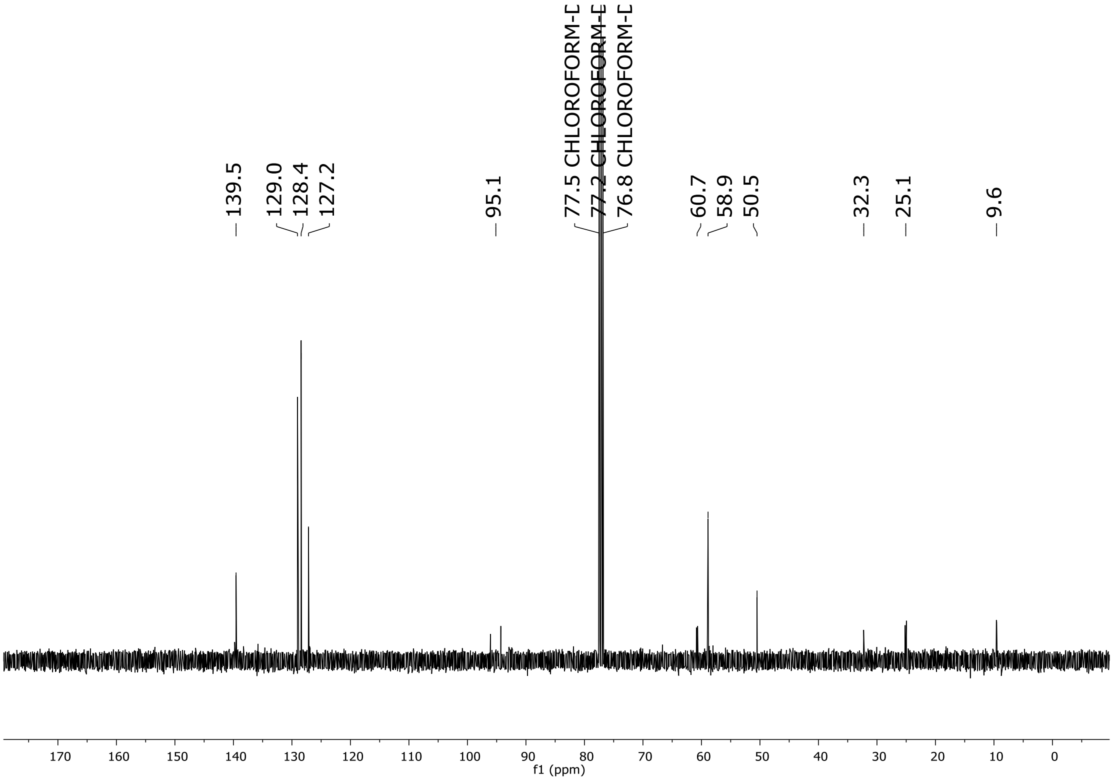

(4S,5S)-N,N-dibenzyl-4-chloro-5-fluorohexan-1-amine, 27b

^{1}H NMR (500 MHz, CDCl$_3$):

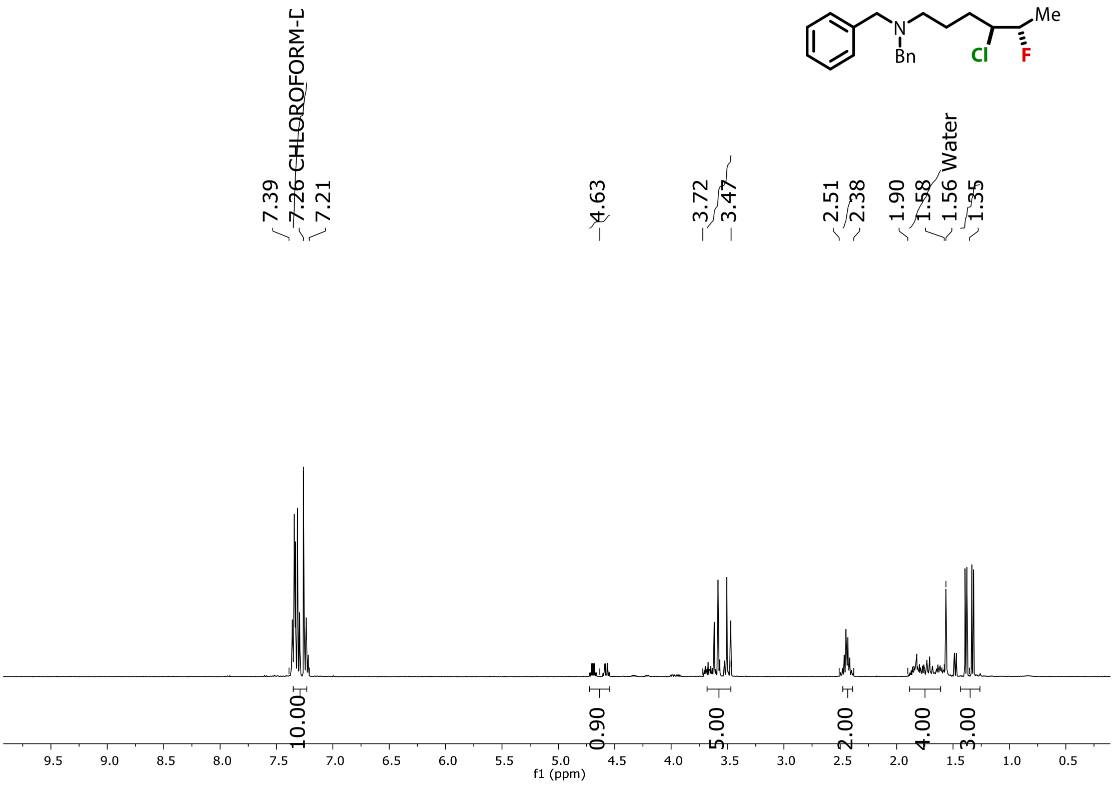

^{19}F NMR (376 MHz, CDCl$_3$):

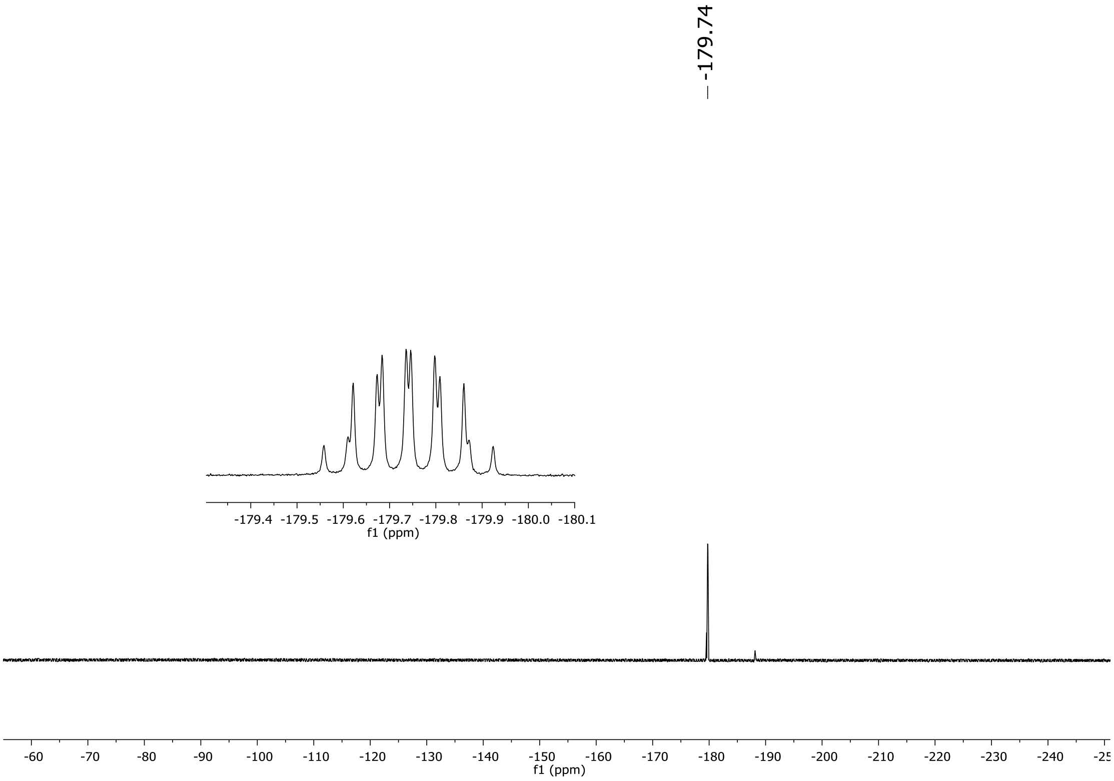

^{13}C {^{1}H} NMR (125 MHz, $CDCl_3$):

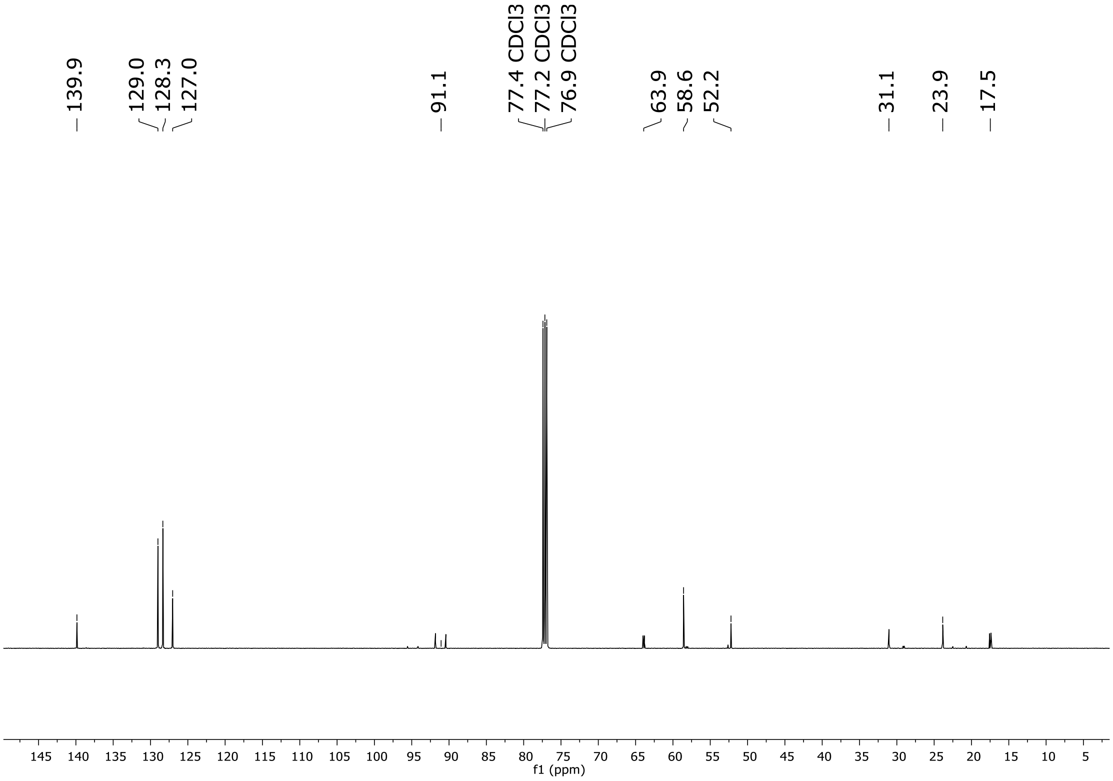

(3S,5S,6S,8S,9S,10R,13R,14S)-5-chloro-6-fluoro-10,13-dimethyl-17-((R)-6-methylheptan-2-yl)hexadecahydro-1H-cyclopenta[a]phenanthren-3-ol, 28b

^{1}H NMR (500 MHz, $CDCl_3$):

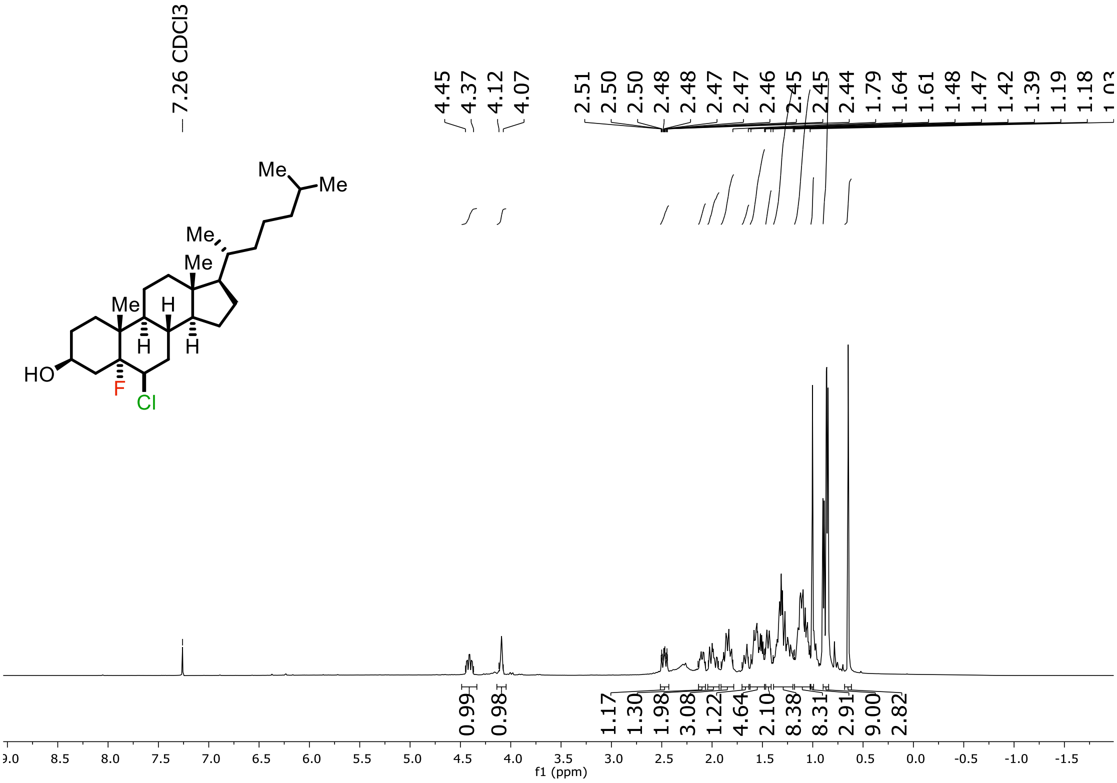

^{19}F NMR (376 MHz, $CDCl_3$):

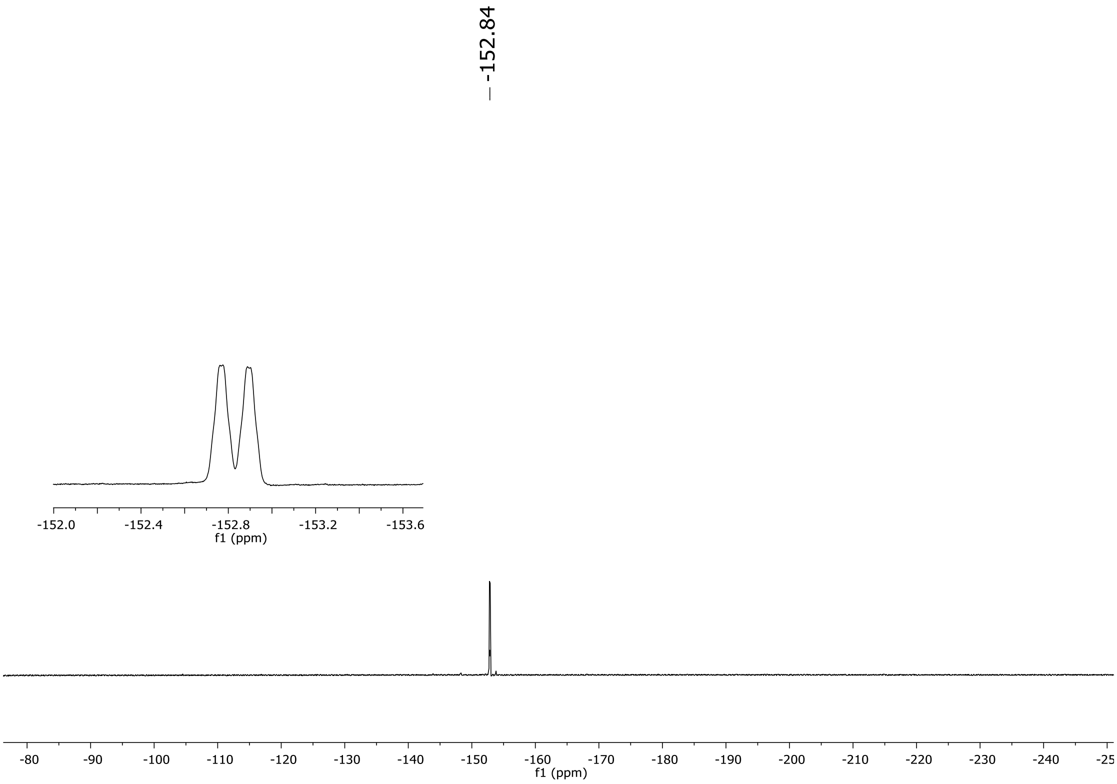

$^{13}C\ \{^{1}H\}$ NMR (125MHz, $CDCl_3$):

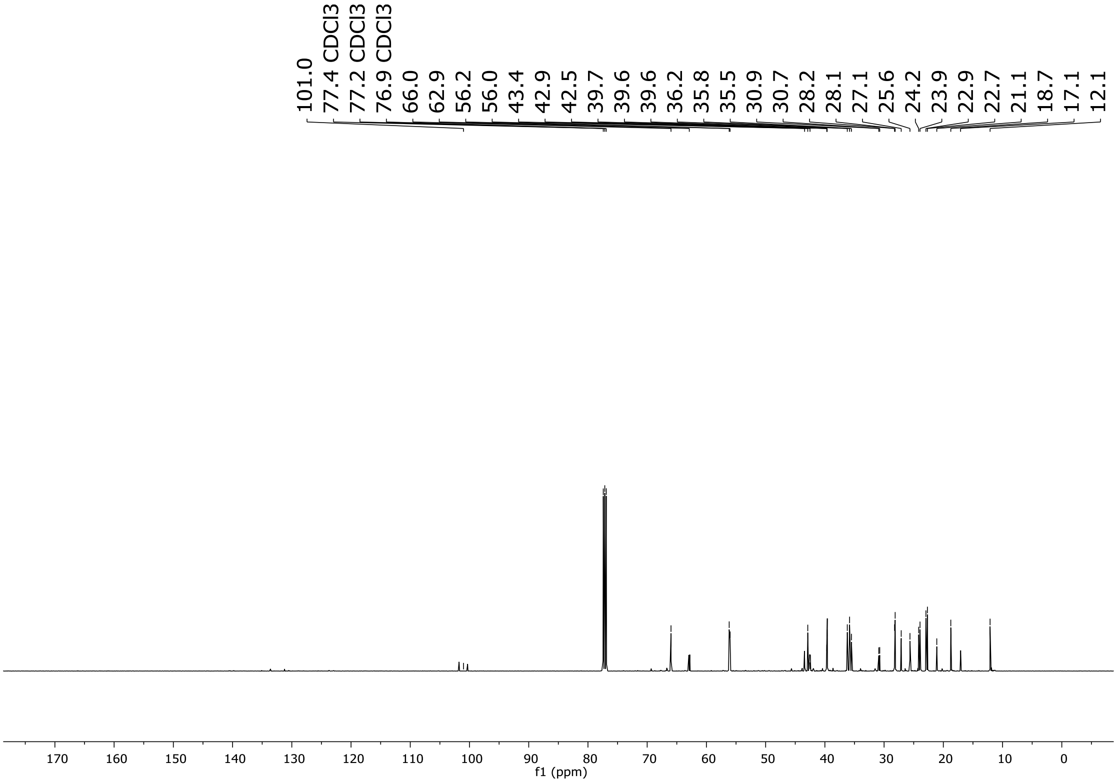

Tert-butyl(((3S,4S)-3-chloro-4-fluorohexyl)oxy)diphenylsilane, 29b

^{1}H NMR (500 MHz, $CDCl_3$):

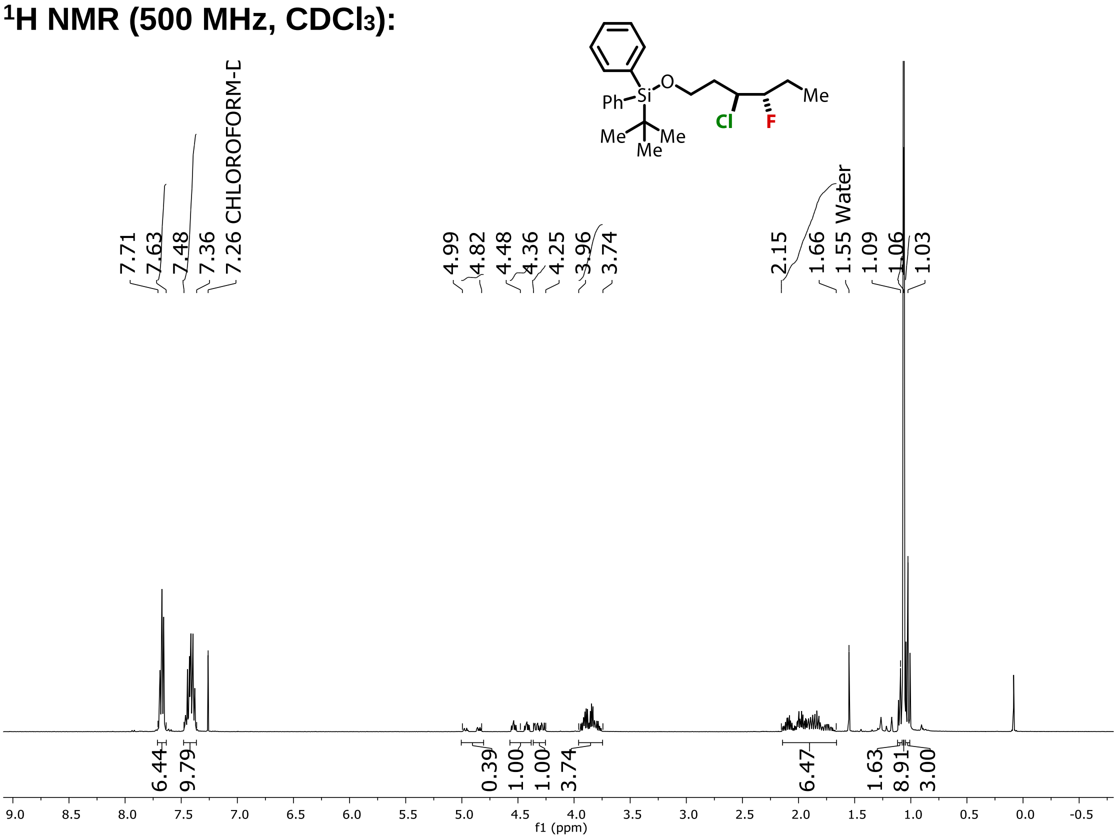

^{19}F NMR (376 MHz, $CDCl_3$):

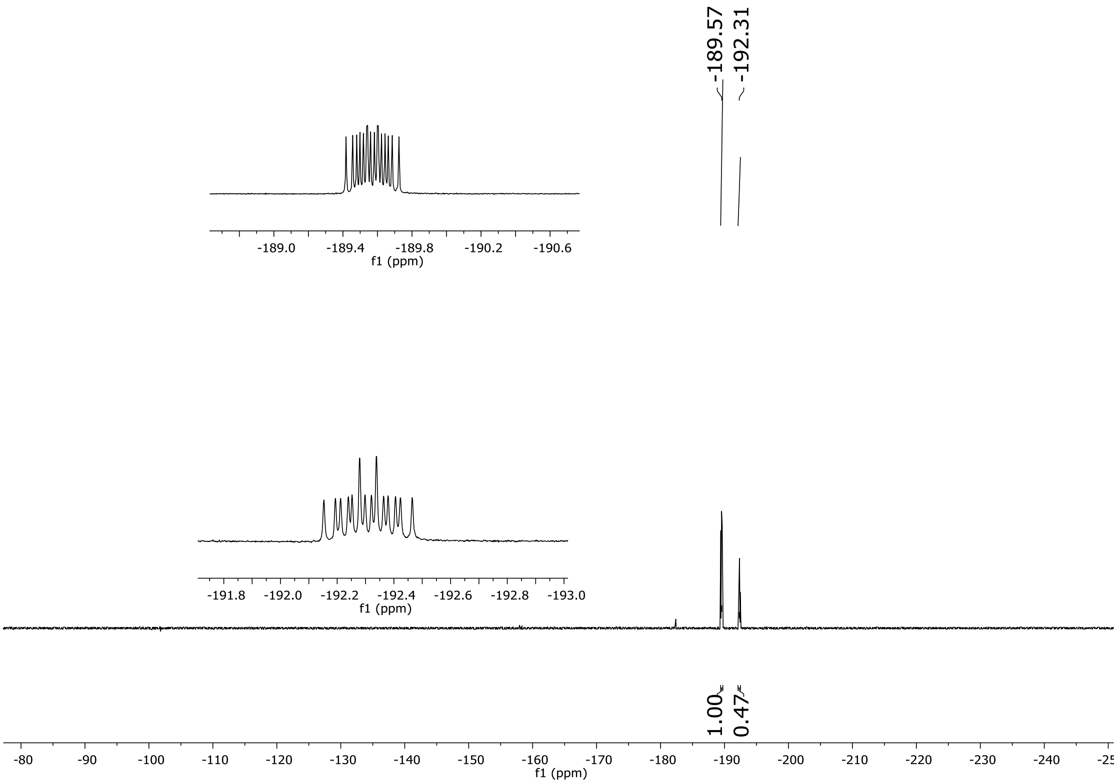

13C {1H} NMR (125 MHz, $CDCl_3$):

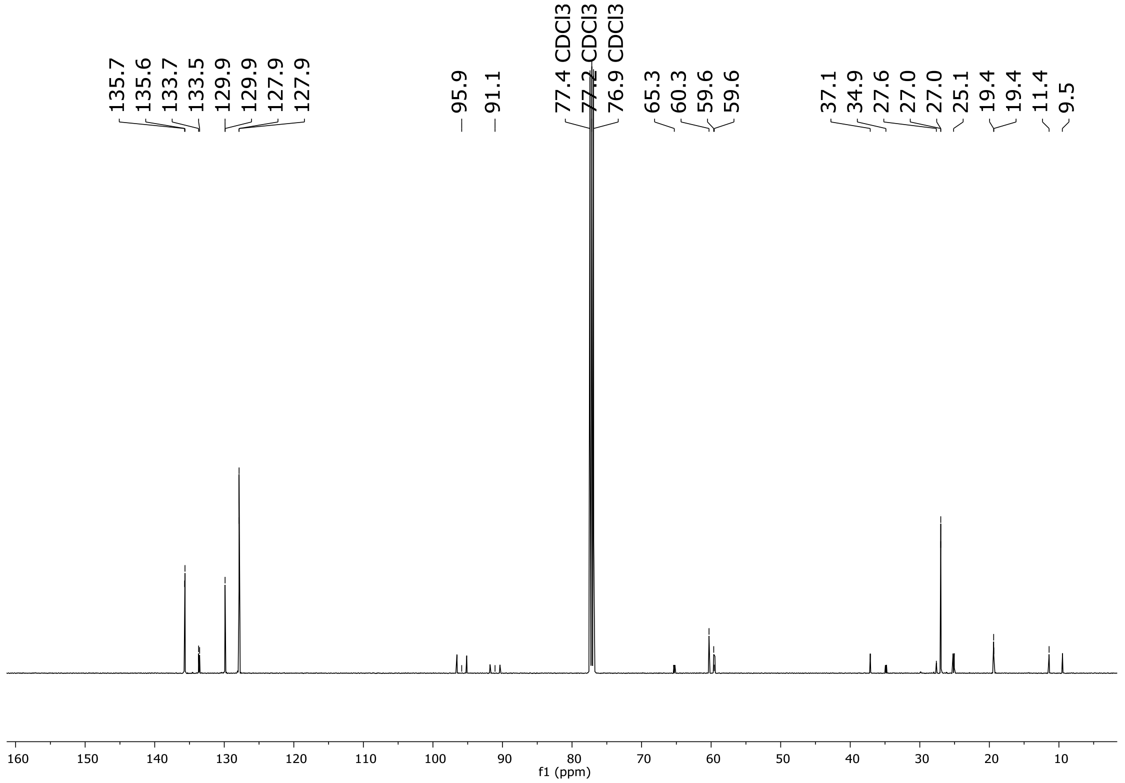

1-chloro-2-fluoropropyl 4-fluorobenzoate, 30d

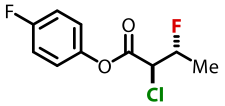

^{1}H NMR (400 MHz, $CDCl_3$):

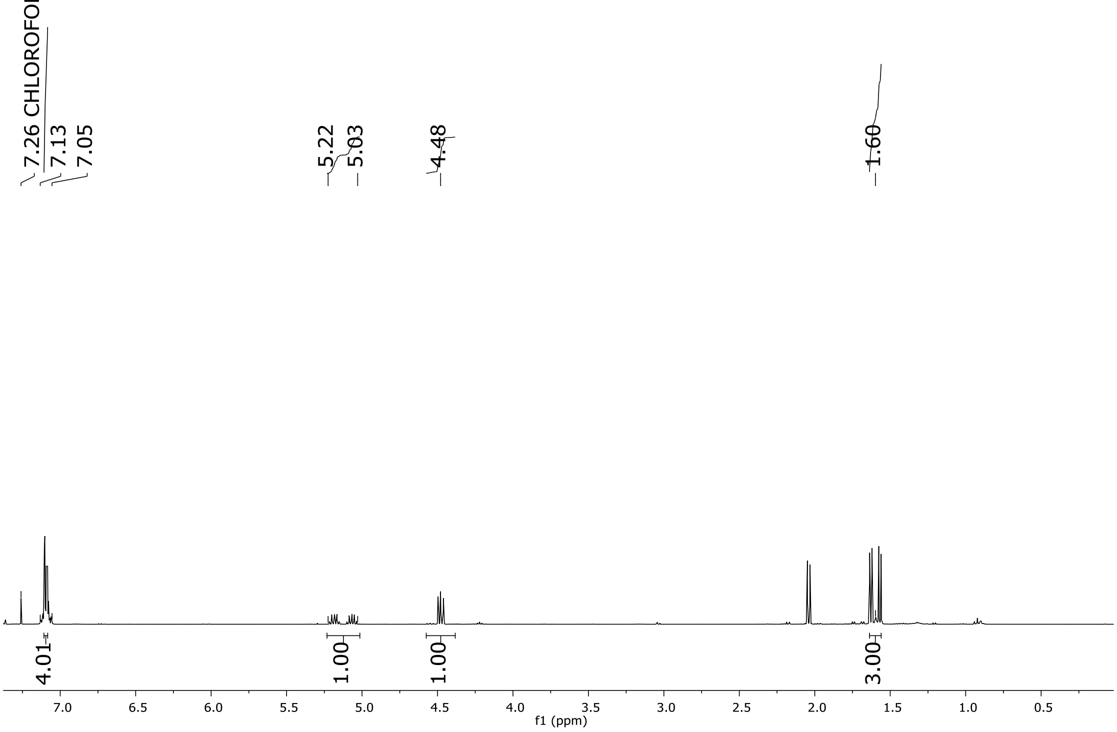

^{19}F NMR (376 MHz, $CDCl_3$):

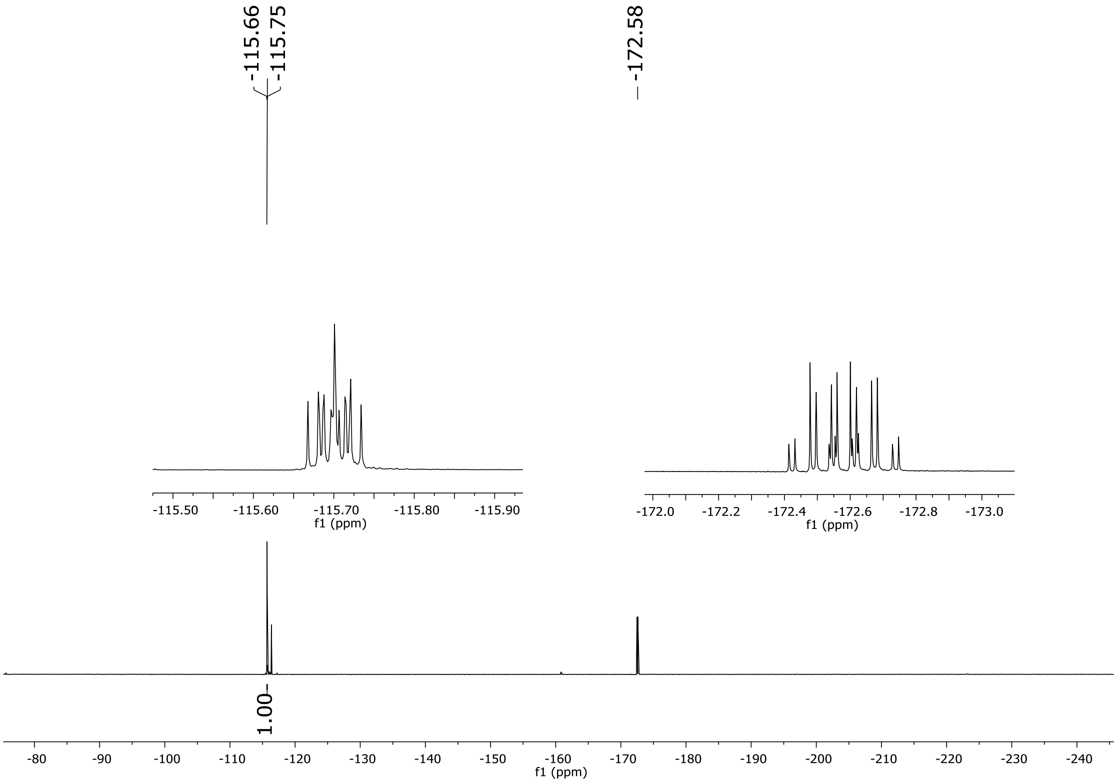

$^{13}C\ \{^1H\}$ NMR (100 MHz, $CDCl_3$):

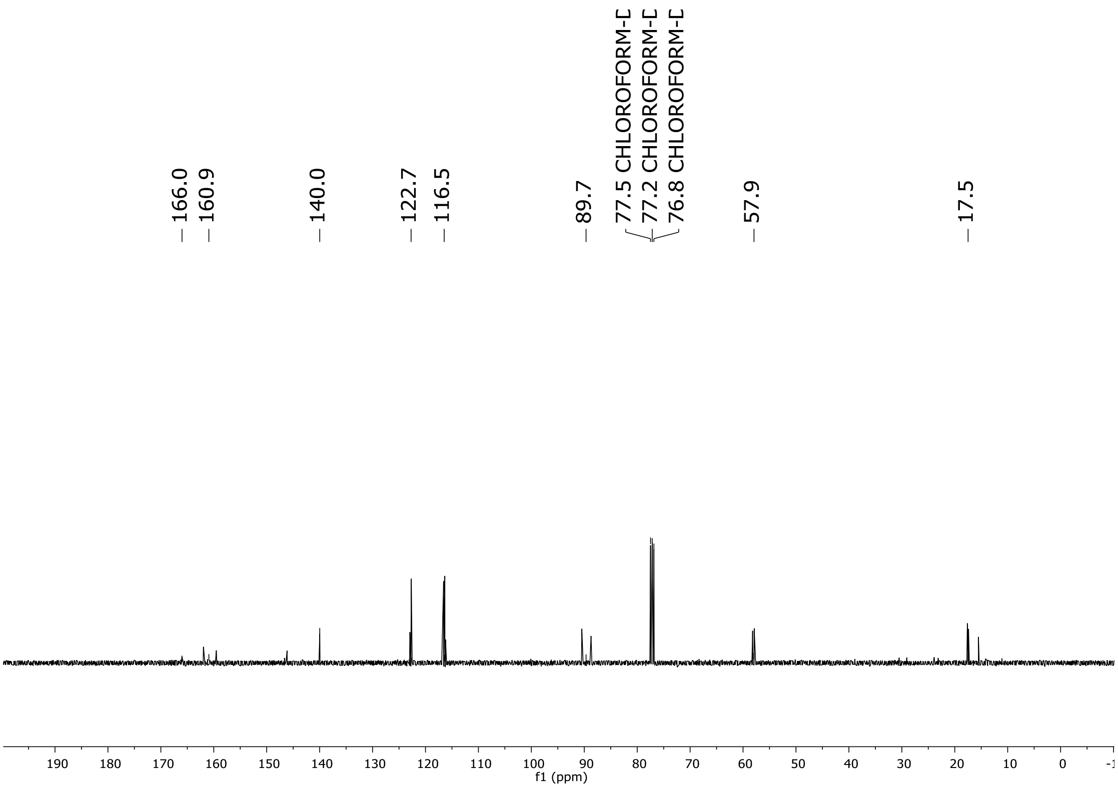

(2S,3R)-N,N-dibenzyl-2-chloro-3-fluorobutanamide, 31d

^{1}H NMR (500 MHz, $CDCl_3$):

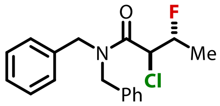

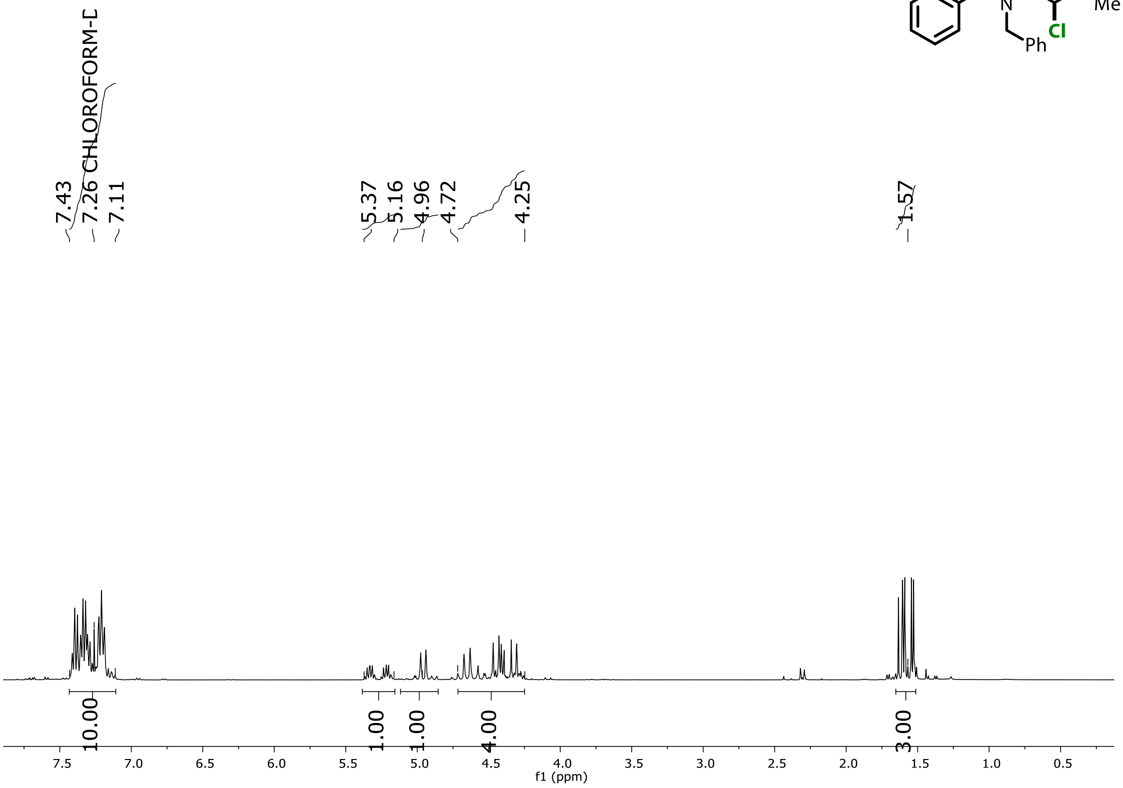

^{19}F NMR (376 MHz, $CDCl_3$):

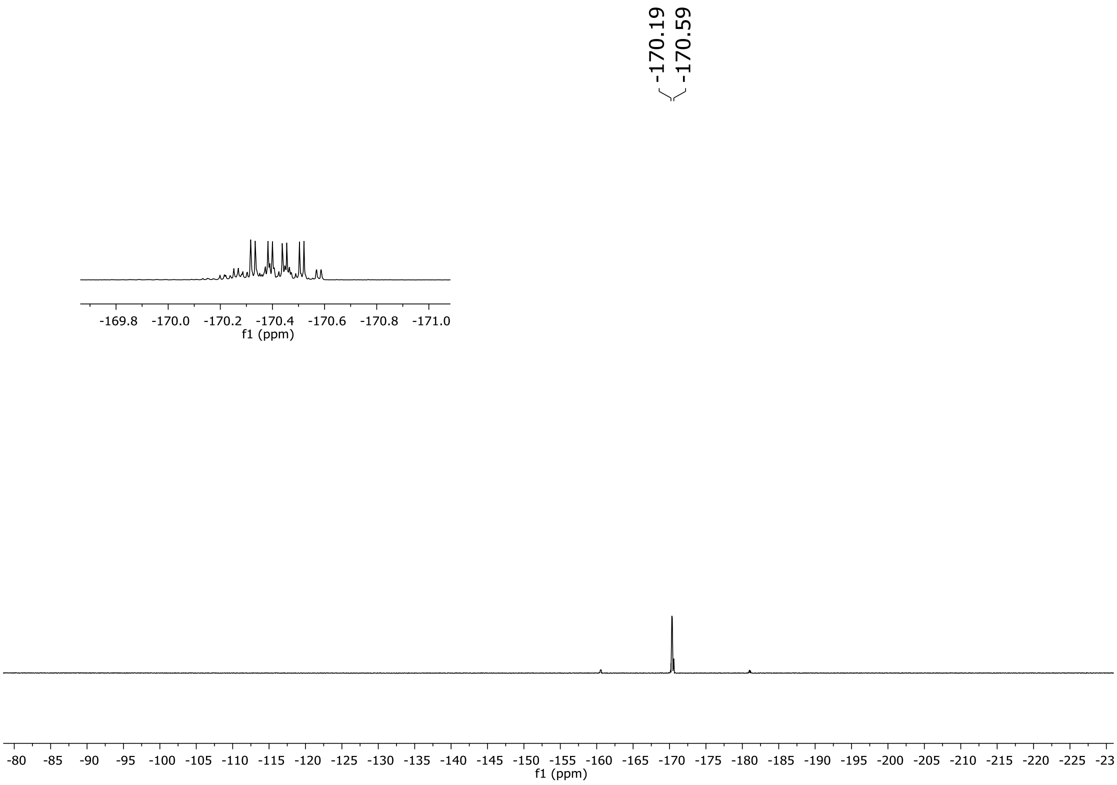

Back to top

^{13}C {^{1}H} NMR (125 MHz, $CDCl_3$):

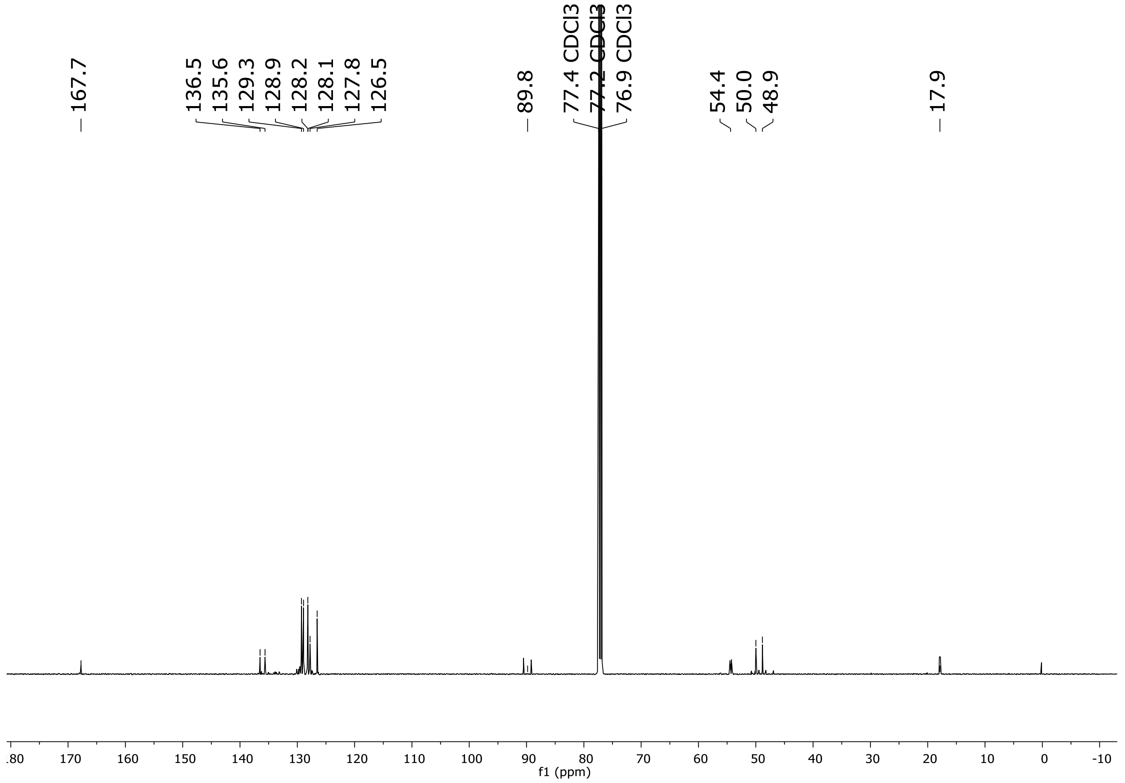

(3S,4S)-3-chloro-4-fluoro-1-tosylpyrrolidine, 32b

^{1}H NMR (500 MHz, $CDCl_3$):

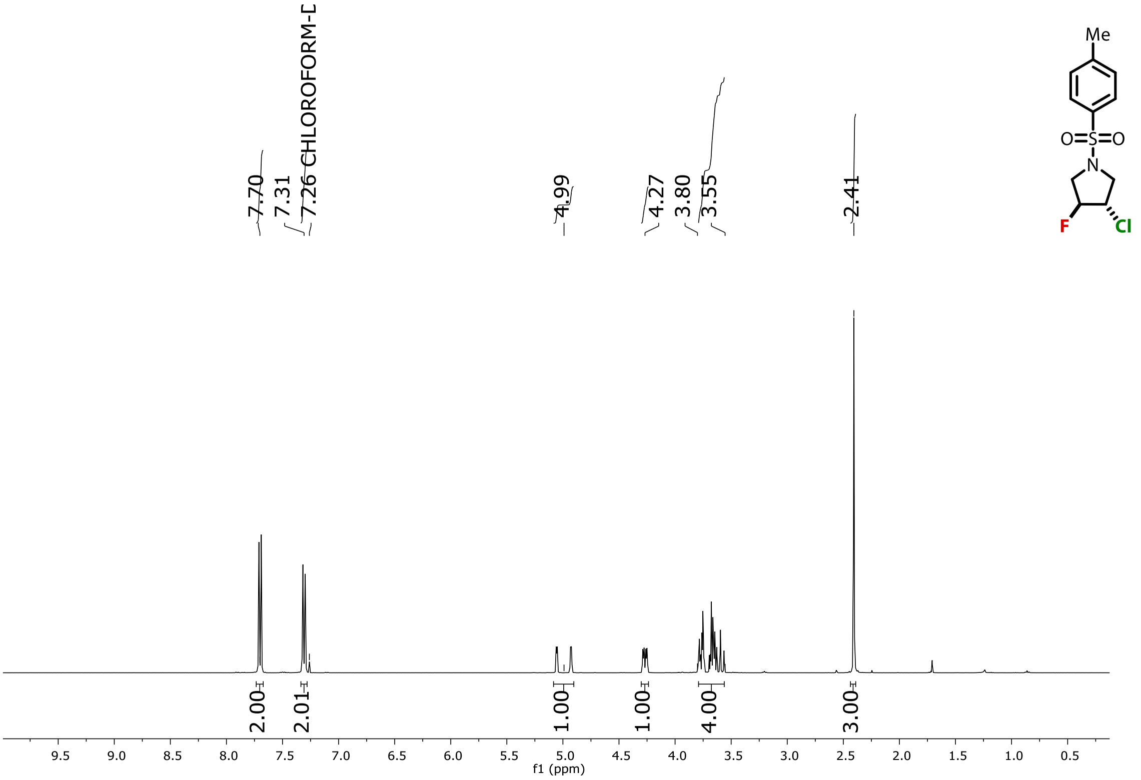

^{19}F NMR (376 MHz, $CDCl_3$):

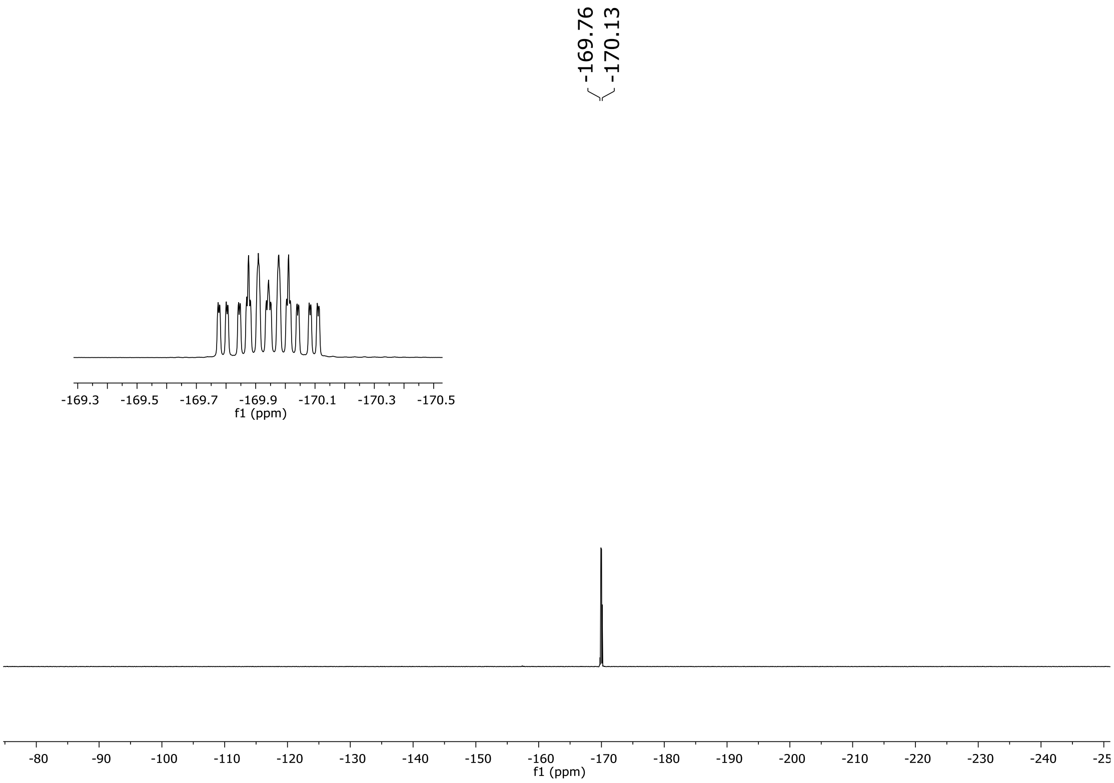

^{13}C {^{1}H} NMR (125 MHz, $CDCl_3$):

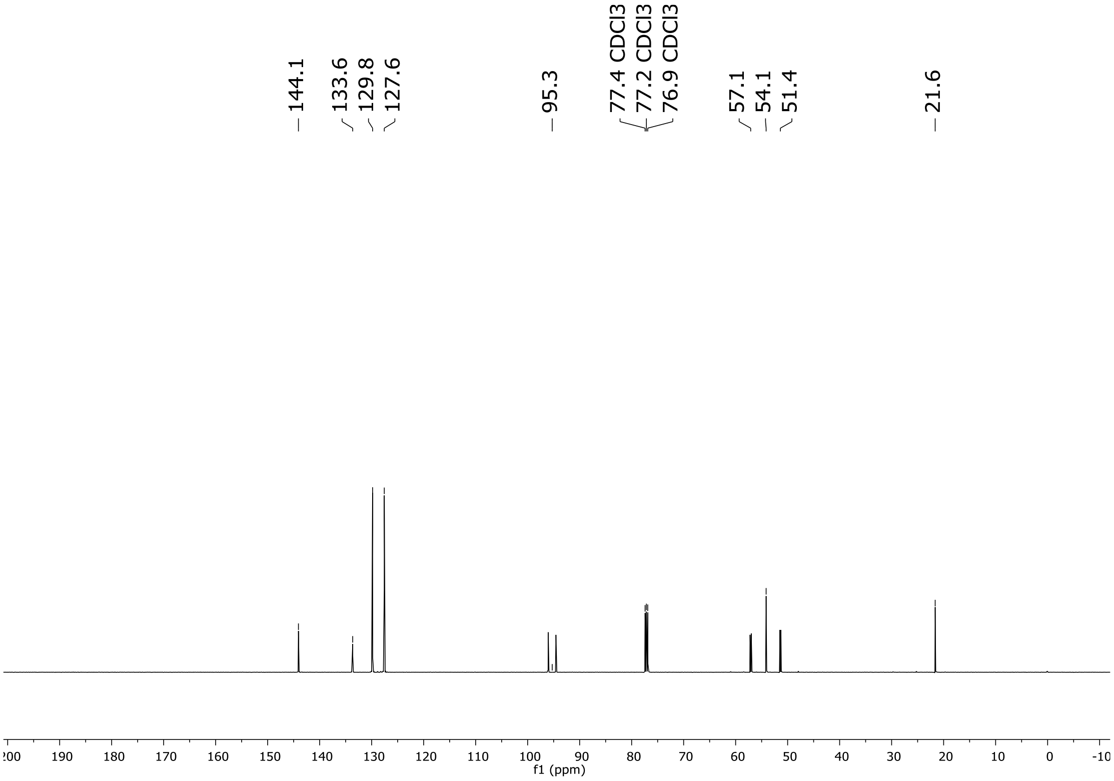

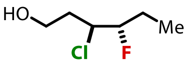

(3S,4S)-3-chloro-4-fluorohexan-1-ol, 33b

^{1}H NMR (500 MHz, $CDCl_3$):

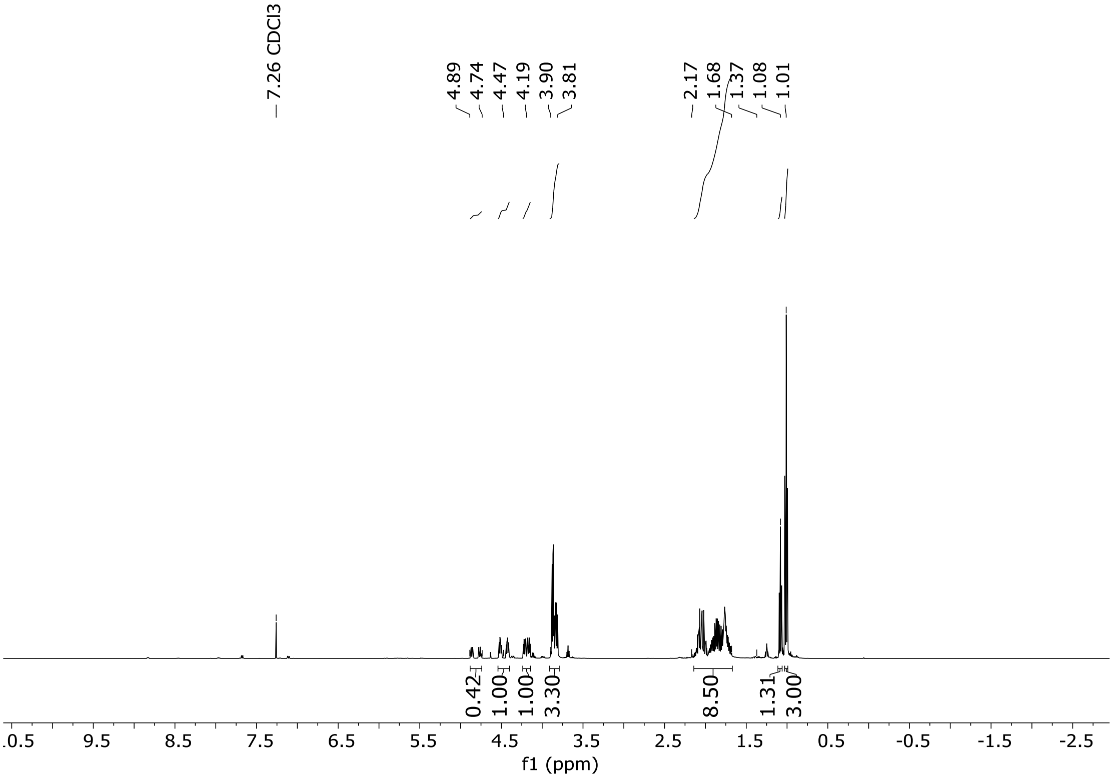

^{19}F NMR (376 MHz, $CDCl_3$):

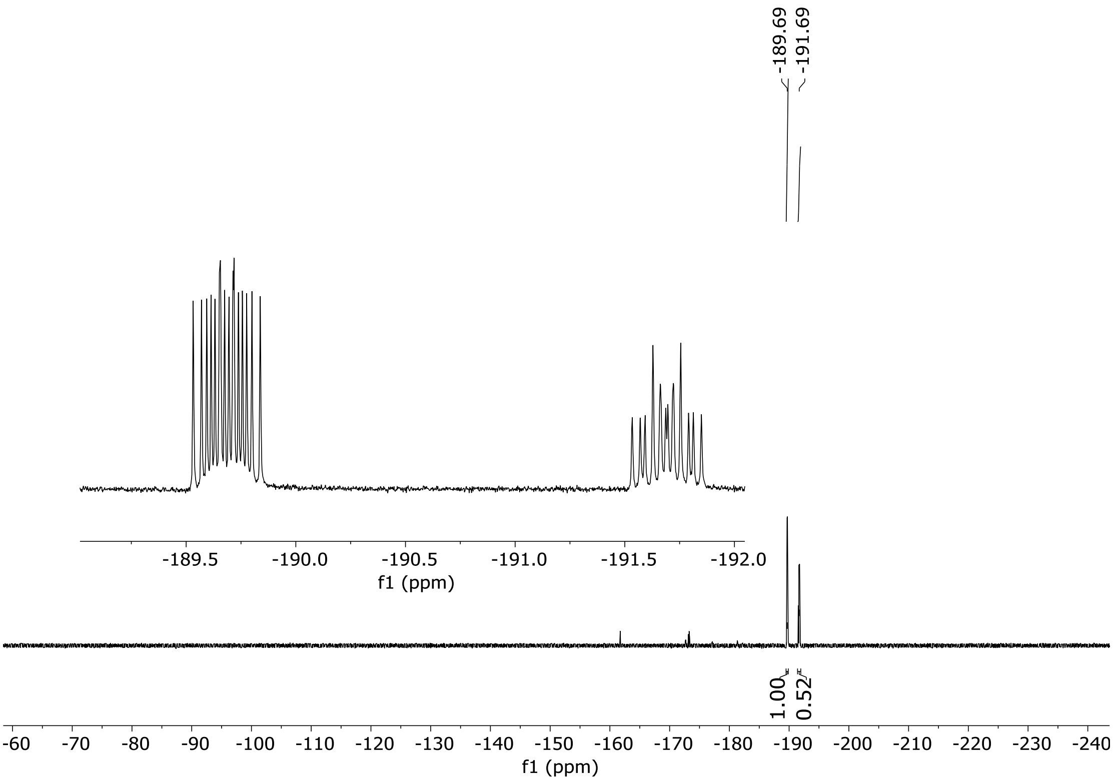

Back to top

^{13}C {^{1}H} NMR (125 MHz, $CDCl_3$):

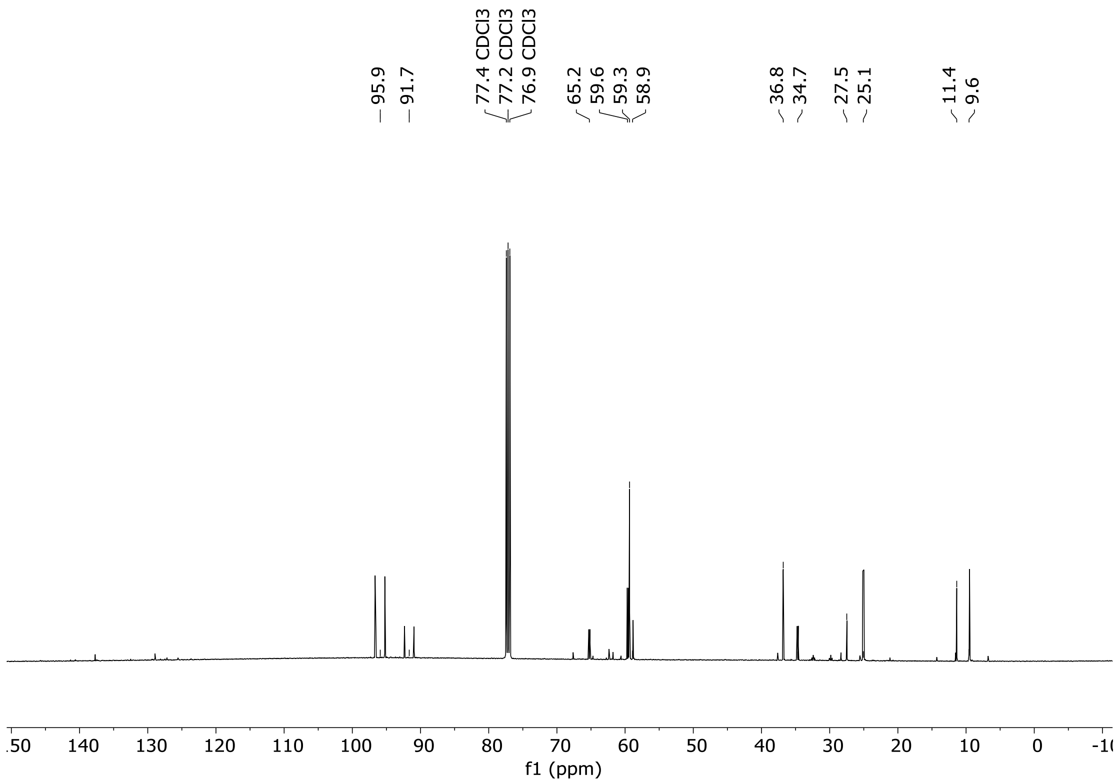

tert-butyl 9-((3*R*,4*R*)-3-chloro-4-fluorohexyl)-3,9-diazaspiro[5.5]undecane-3-carboxylate, 34b

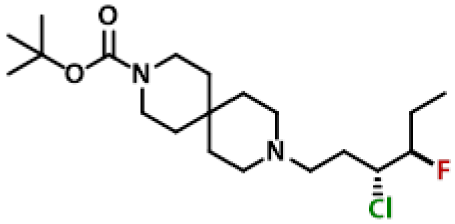

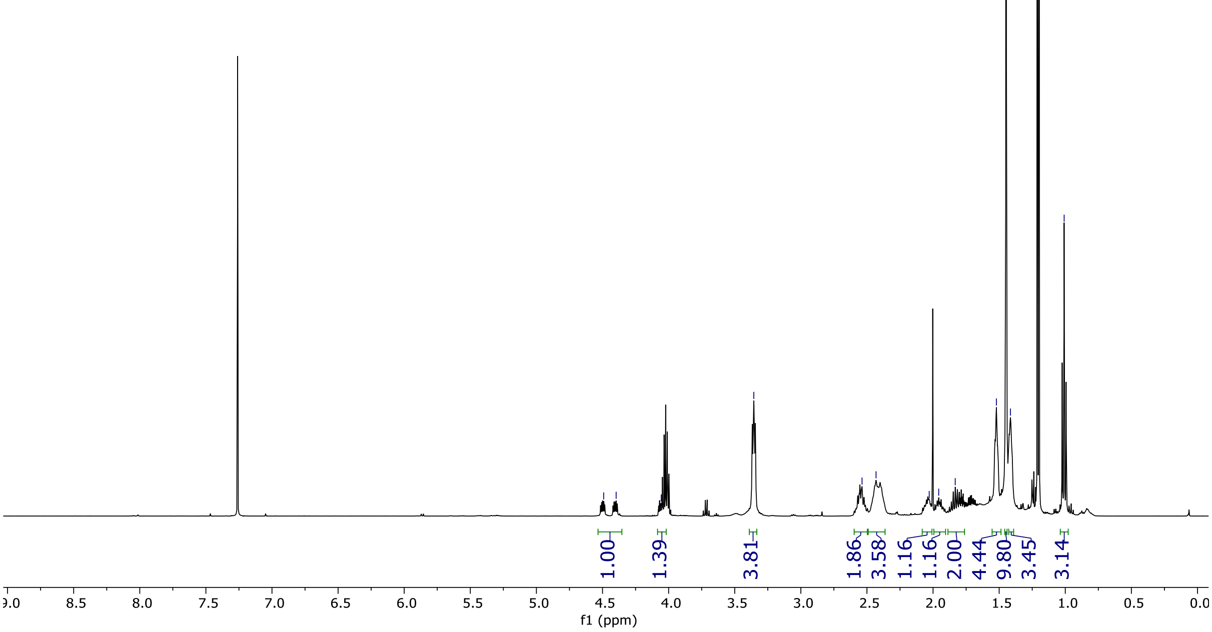

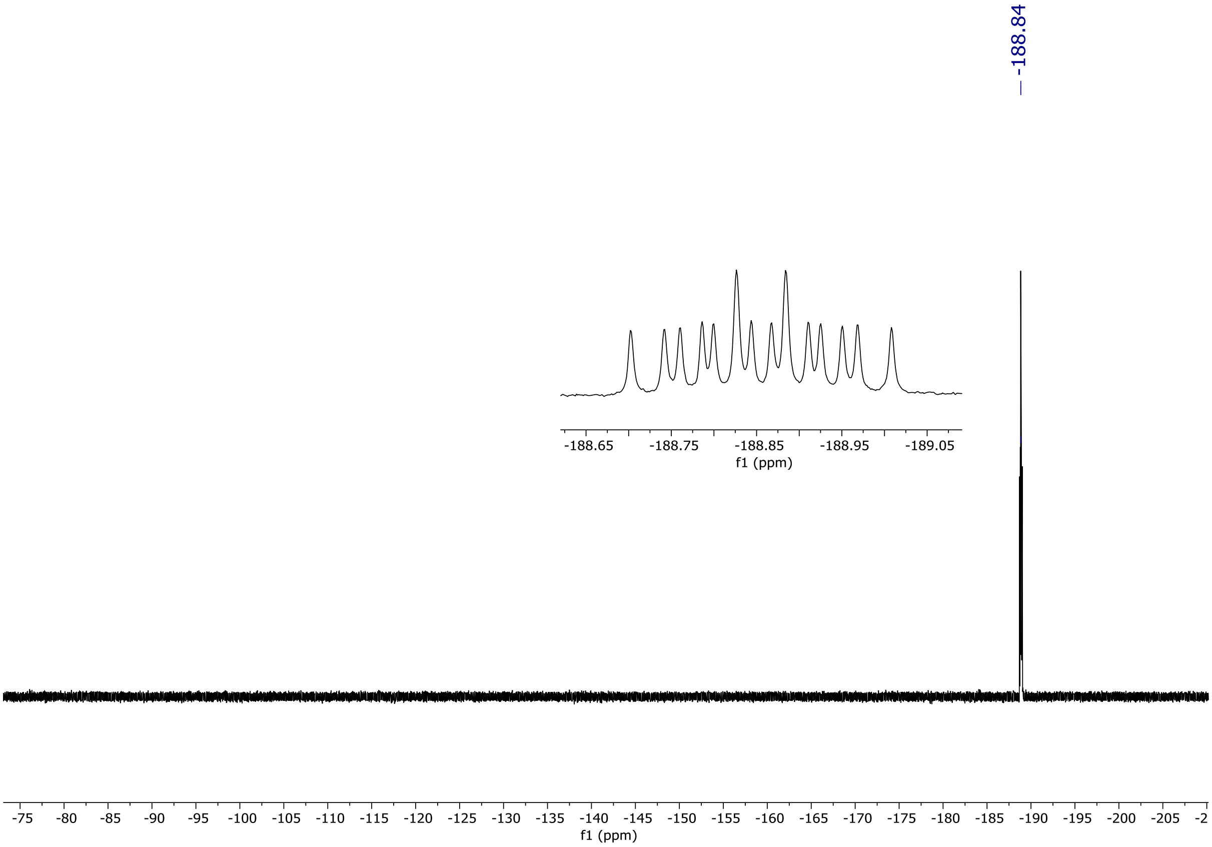

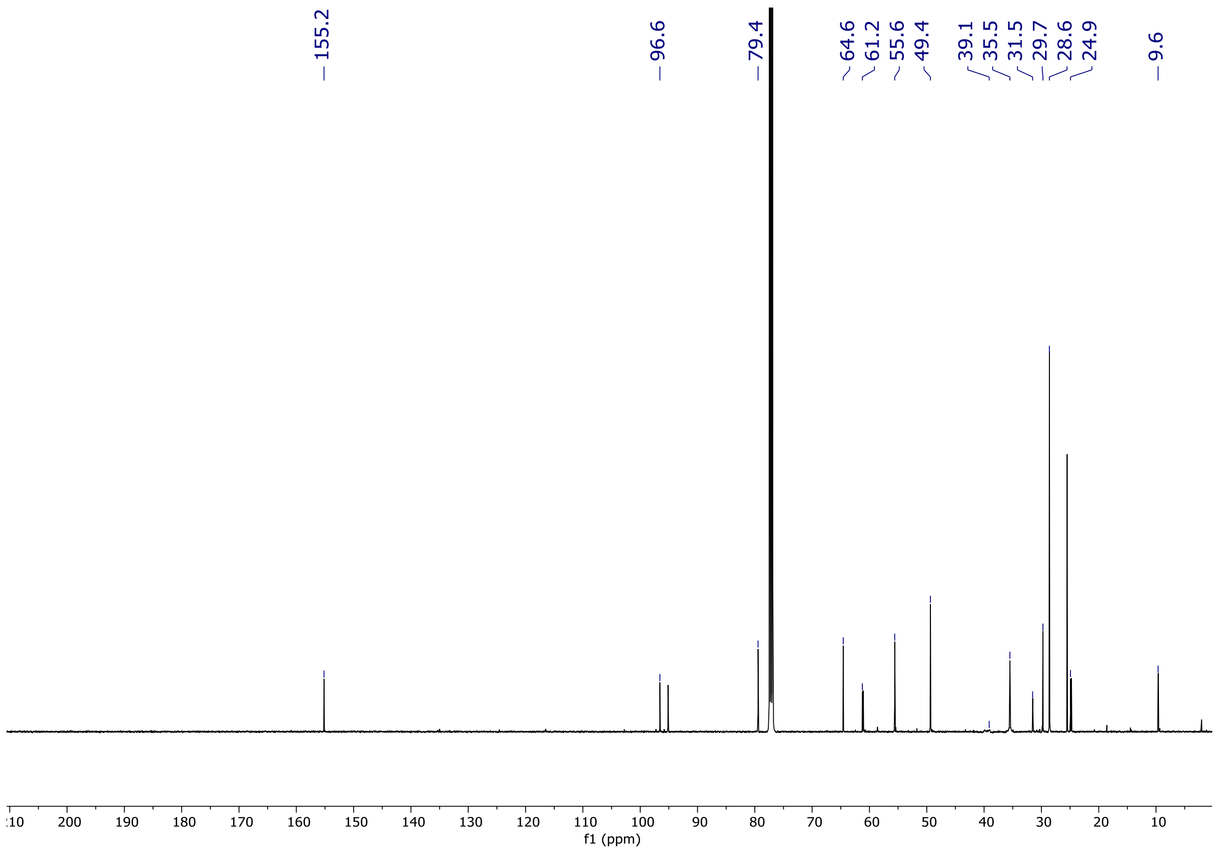
155.2
96.6
79.4
64.6
61.2
55.6
49.4
39.1
35.5
31.5
29.7
28.6
24.9
9.6
210
200
190
180
170
160
150
140
130
120
110
100
90
80
70
60
50
40
30
20
10
f1 (ppm)

3,4,6-tri-O-acetyl-2-deoxy-2-chloro-α-D-mannopyranosyl fluoride, α-anti-35b

^{1}H NMR (400 MHz, $CDCl_3$):

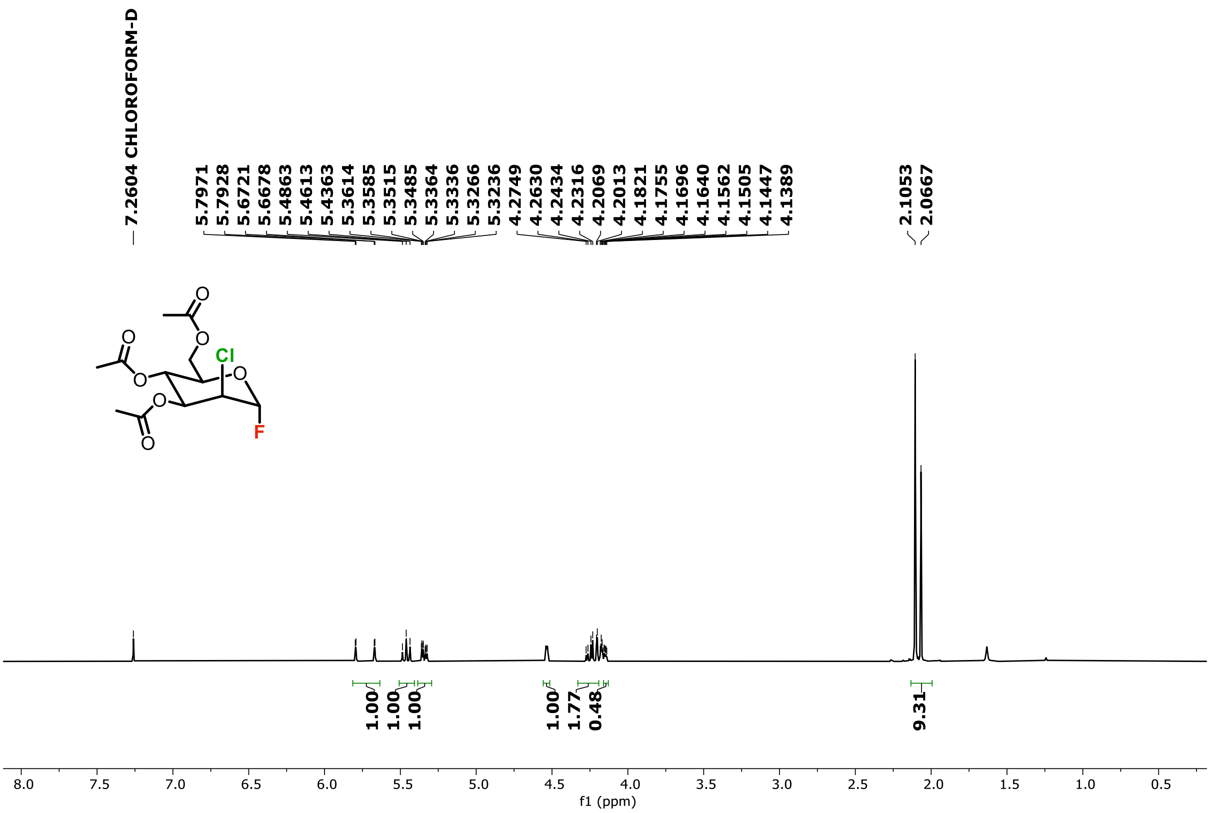

^{19}F NMR (376 MHz, $CDCl_3$):

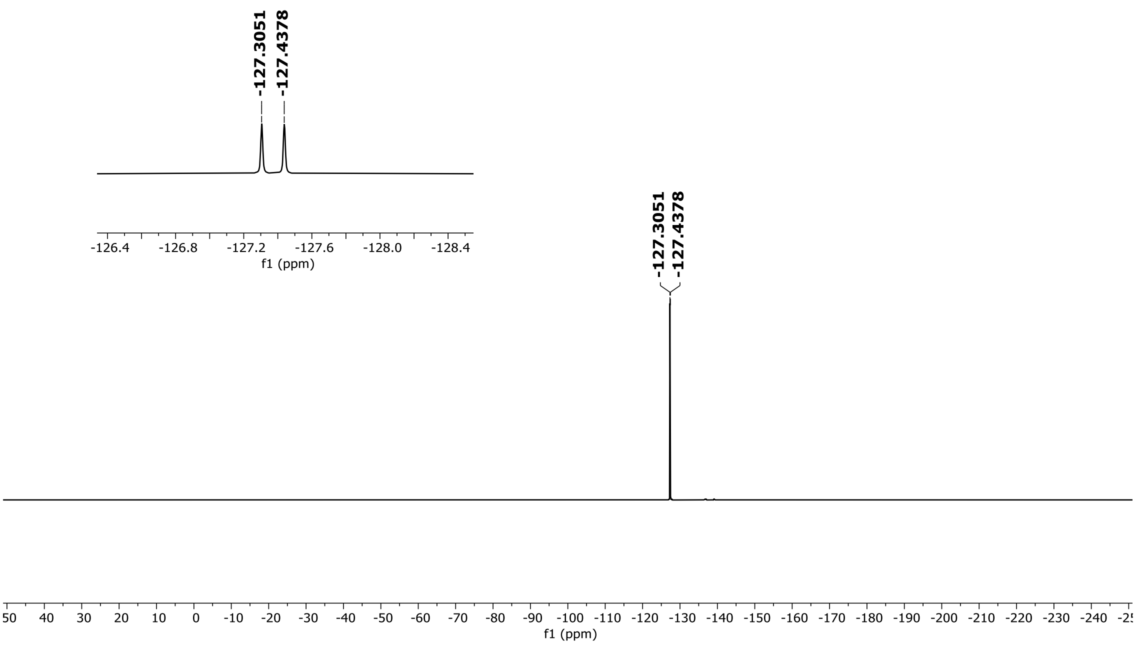

^{13}C {^{1}H} NMR (100 MHz, $CDCl_3$):

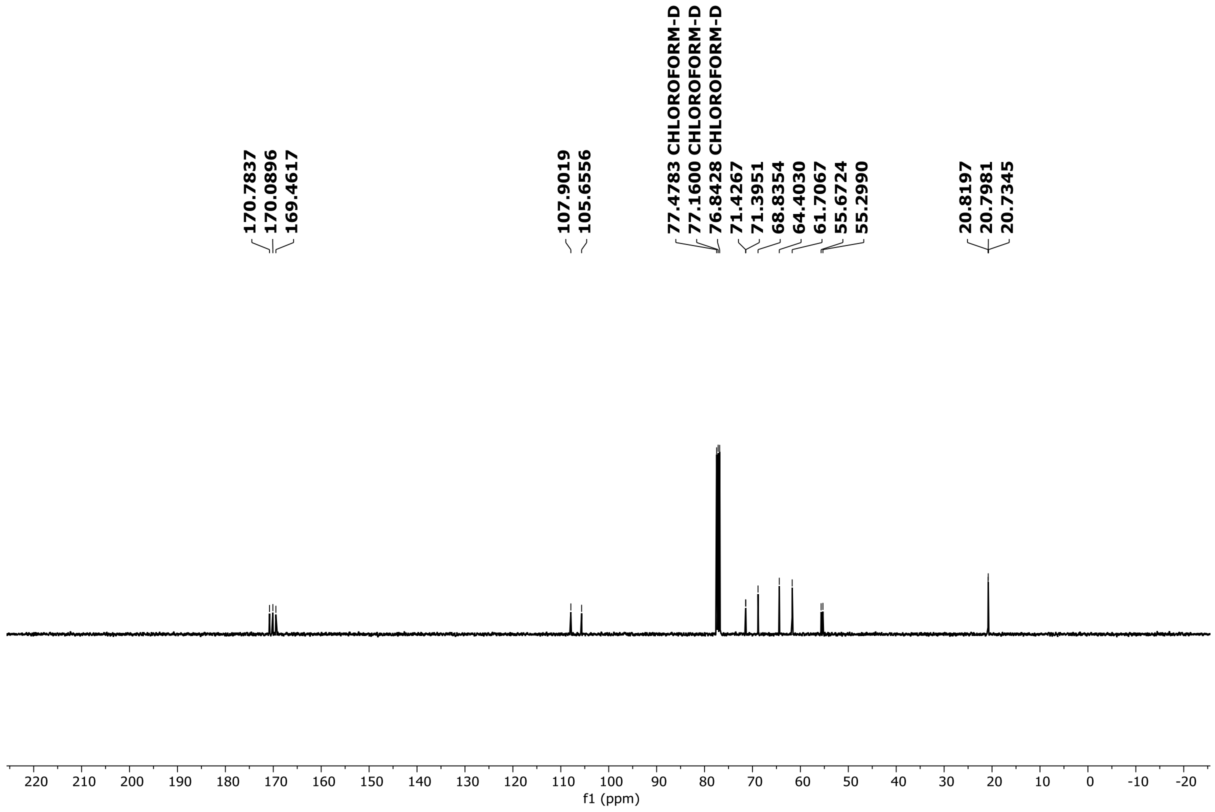

***3,4,6-tri-O-acetyl-2-deoxy-2-chloro-α-D-glucopyranosyl fluoride,* α-*syn*-35b**

^{1}H NMR (400 MHz, $CDCl_3$):

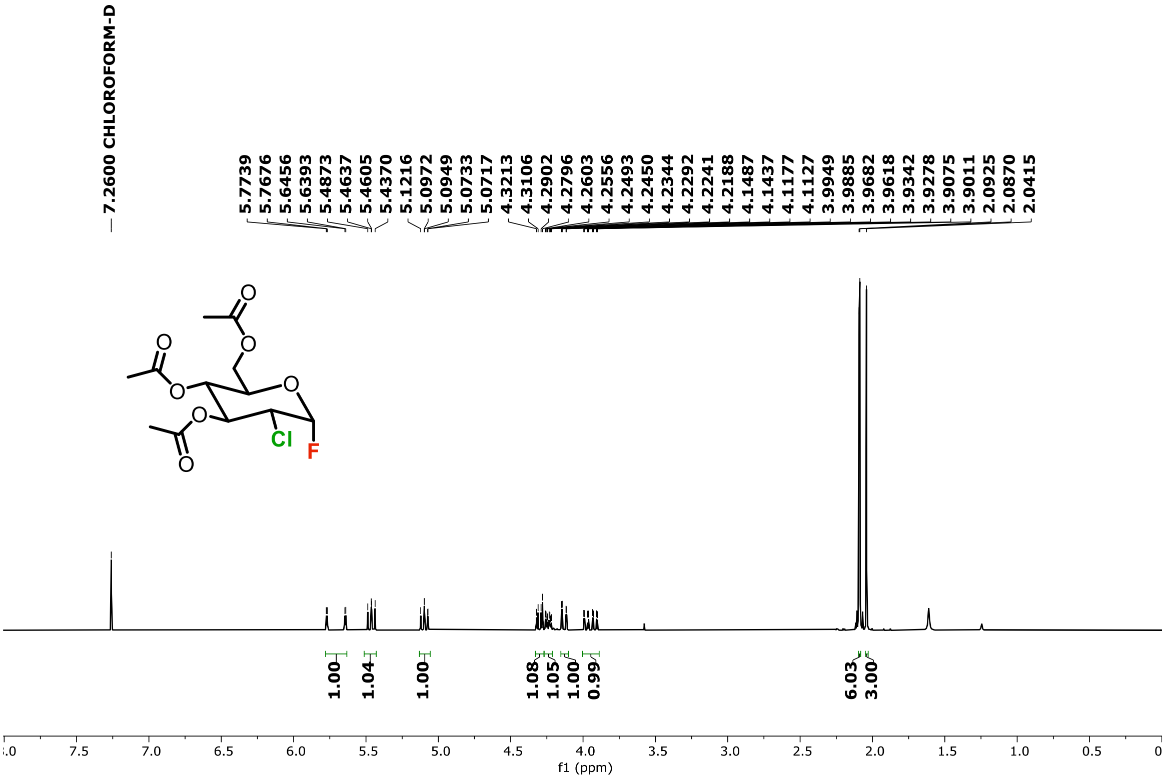

^{19}F NMR (376 MHz, $CDCl_3$):

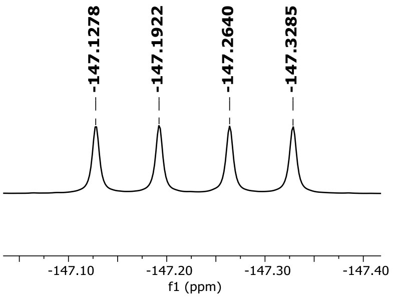

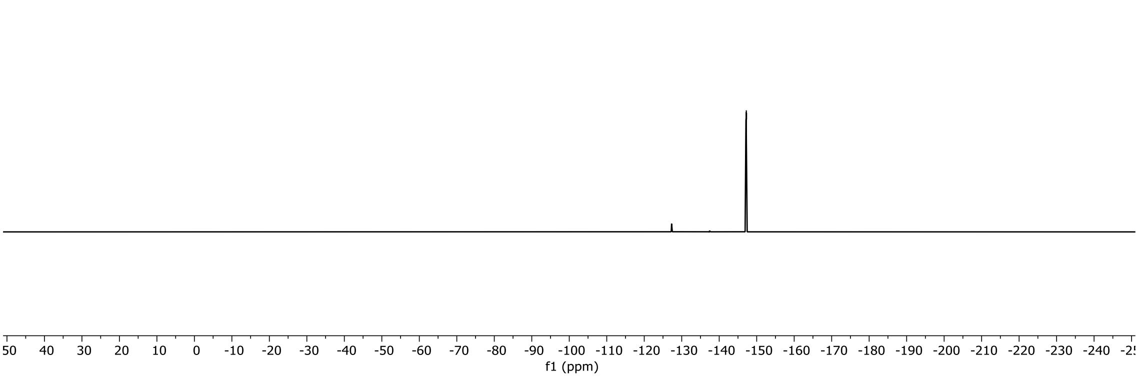

^{13}C {^{1}H} NMR (100 MHz, $CDCl_3$):

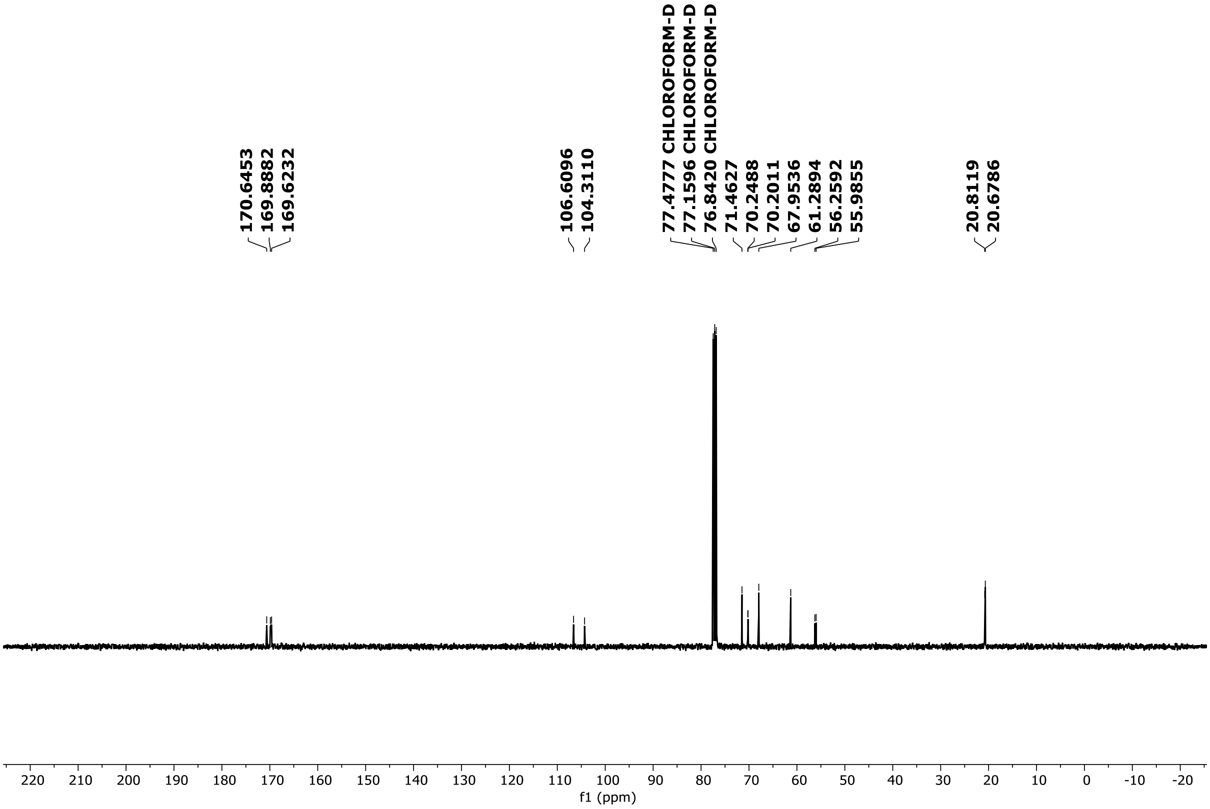

3,4,6-tri-O-acetyl-2-deoxy-2-chloro-β-D-glucopyranosyl fluoride, β-*anti*-35b

^{1}H NMR (400 MHz, $CDCl_3$):

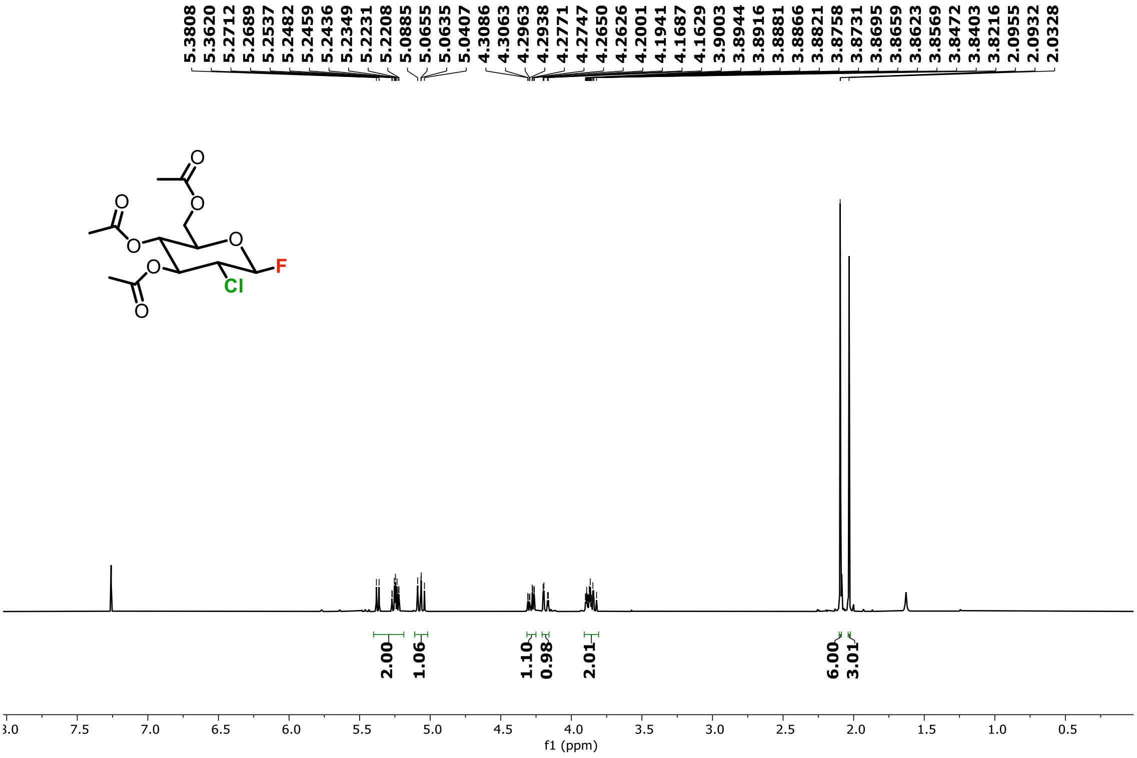

^{19}F NMR (376 MHz, $CDCl_3$):

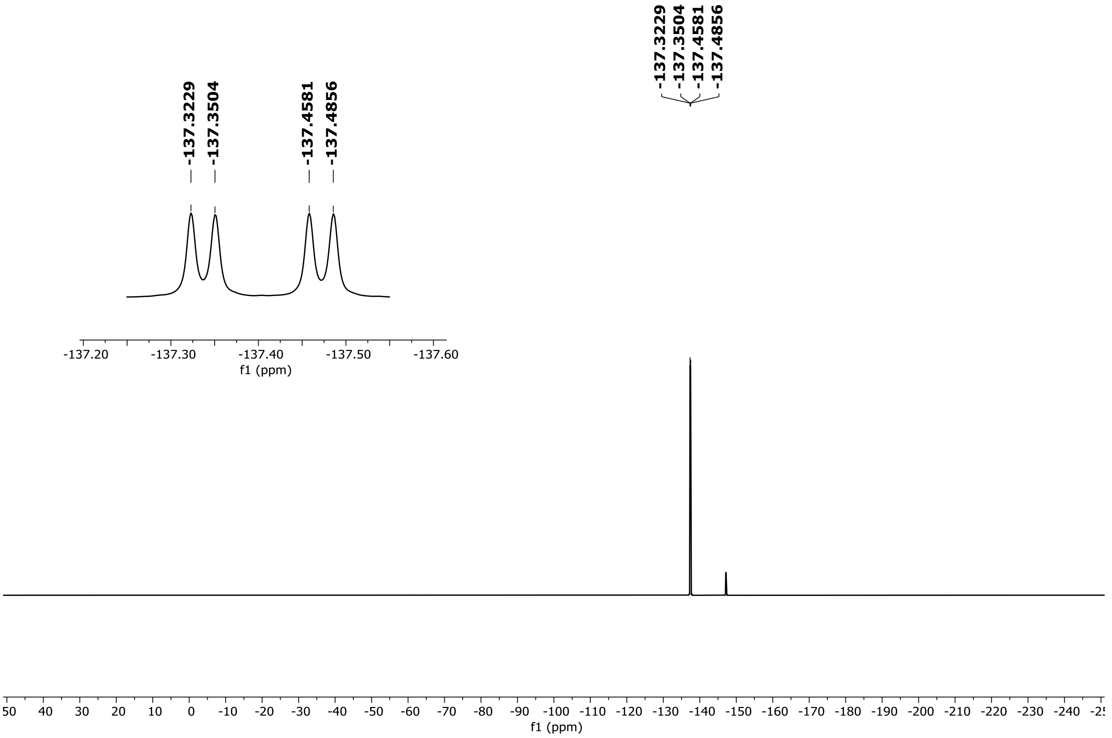

^{13}C {^{1}H} NMR (100 MHz, $CDCl_3$):

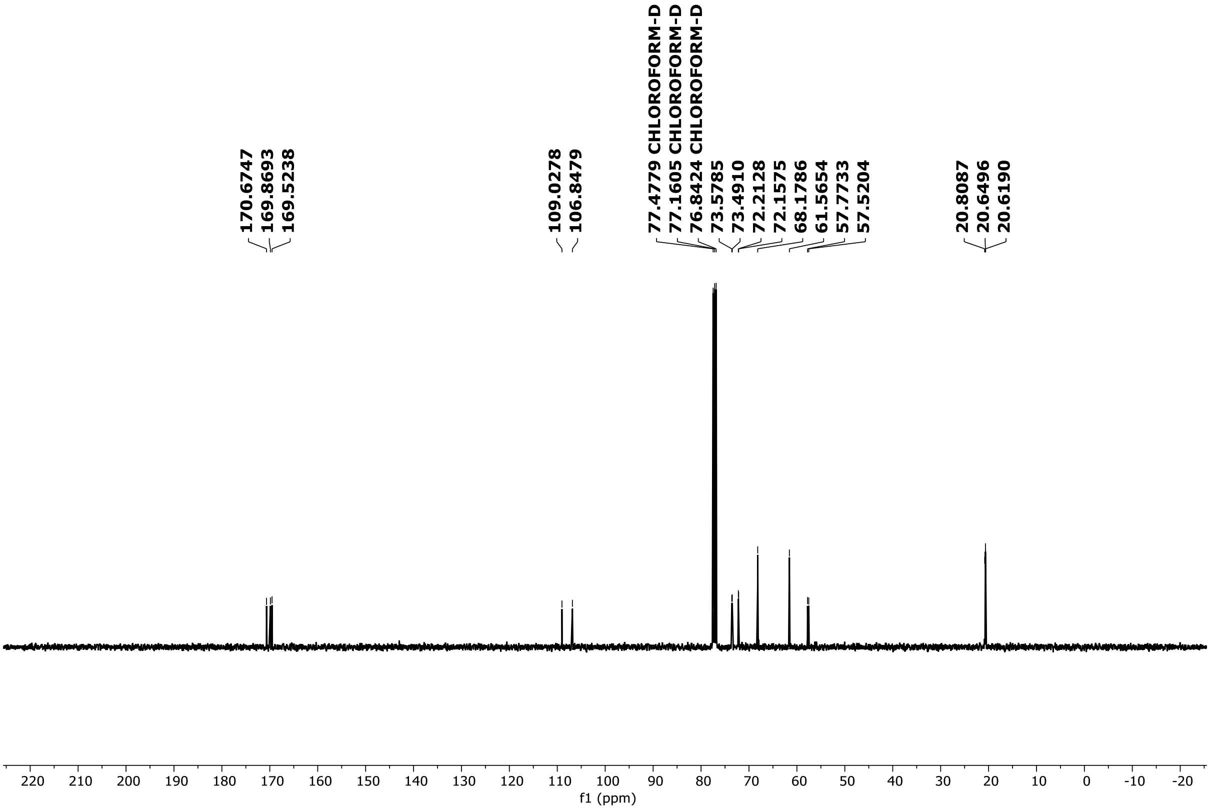

***(3R,4R)*-3-chloro-4-fluorohexyl 3,5-dinitrobenzoate, 36d**

^{1}H NMR (400 MHz, $CDCl_3$):

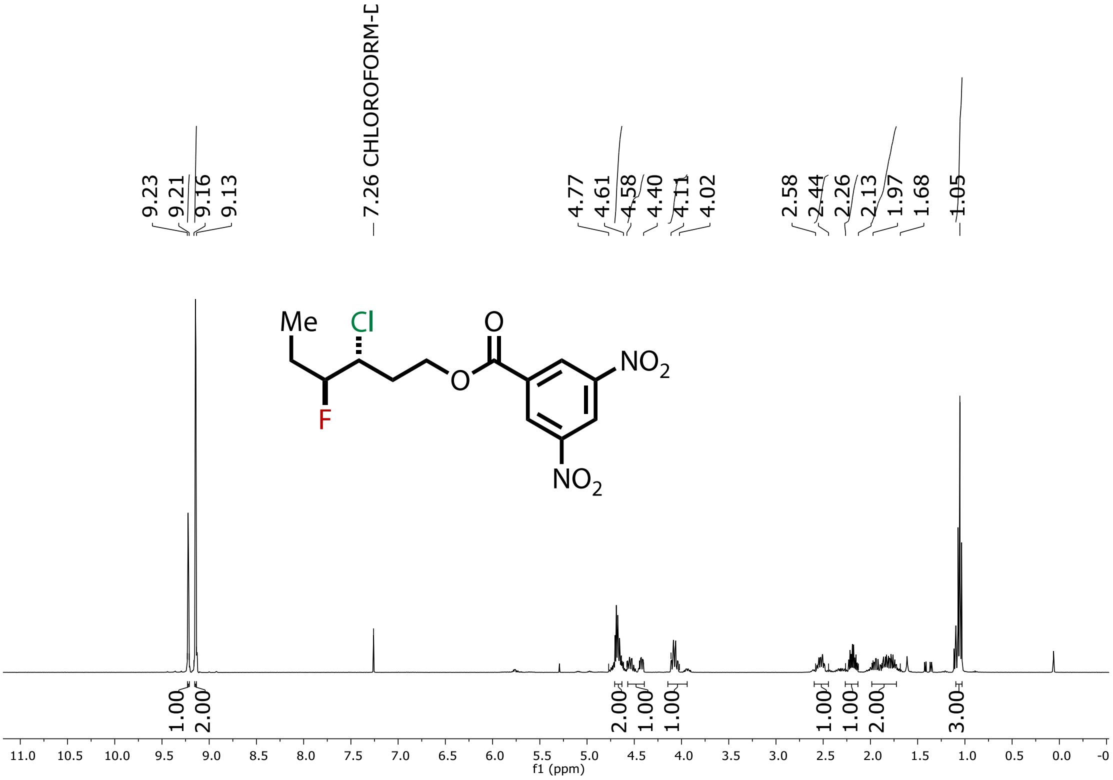

^{19}F NMR (376 MHz, $CDCl_3$):

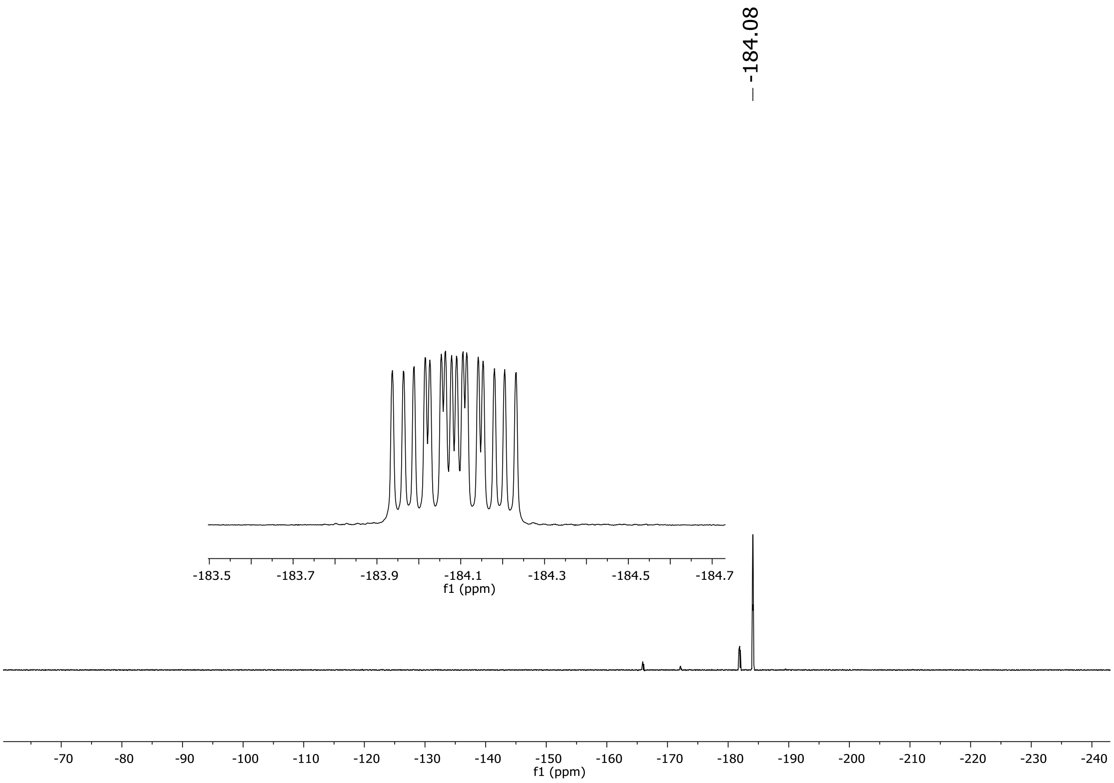

Back to top

$^{13}C\ \{^1H\}$ NMR (100 MHz, $CDCl_3$):

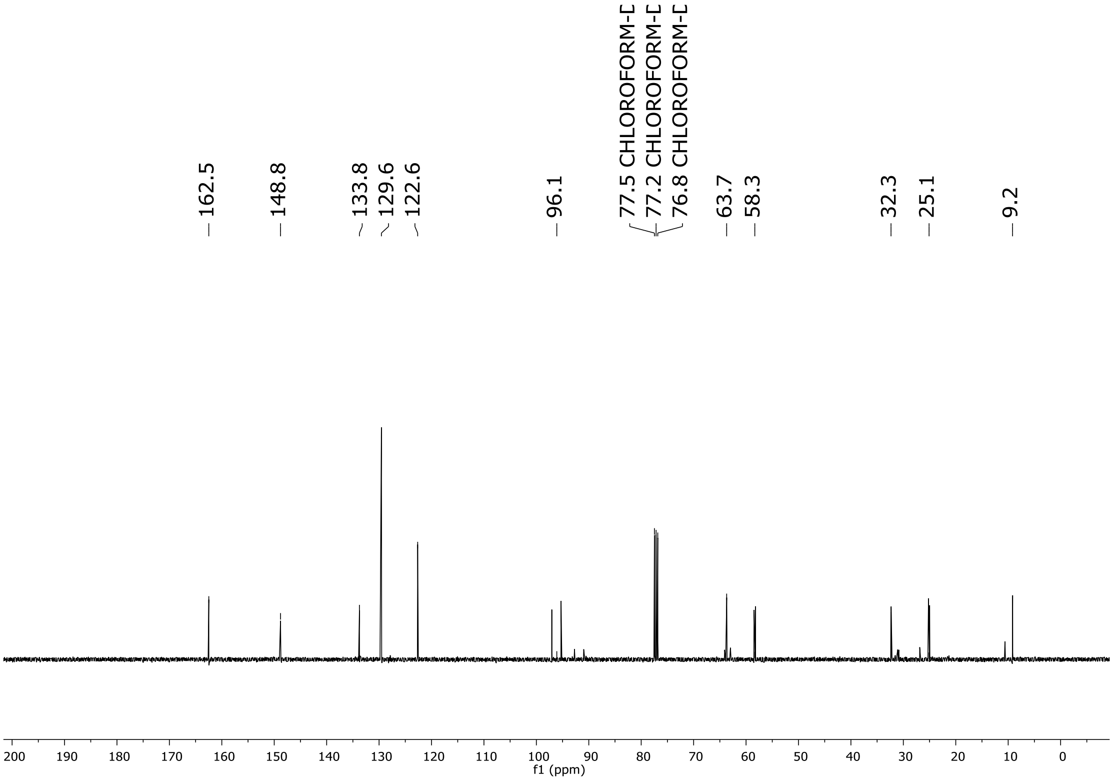

2-(1-((3R,4R)-3-chloro-4-fluorohexyl)piperidin-4-yl)-4,6-dimethoxypyrimidine, 37b

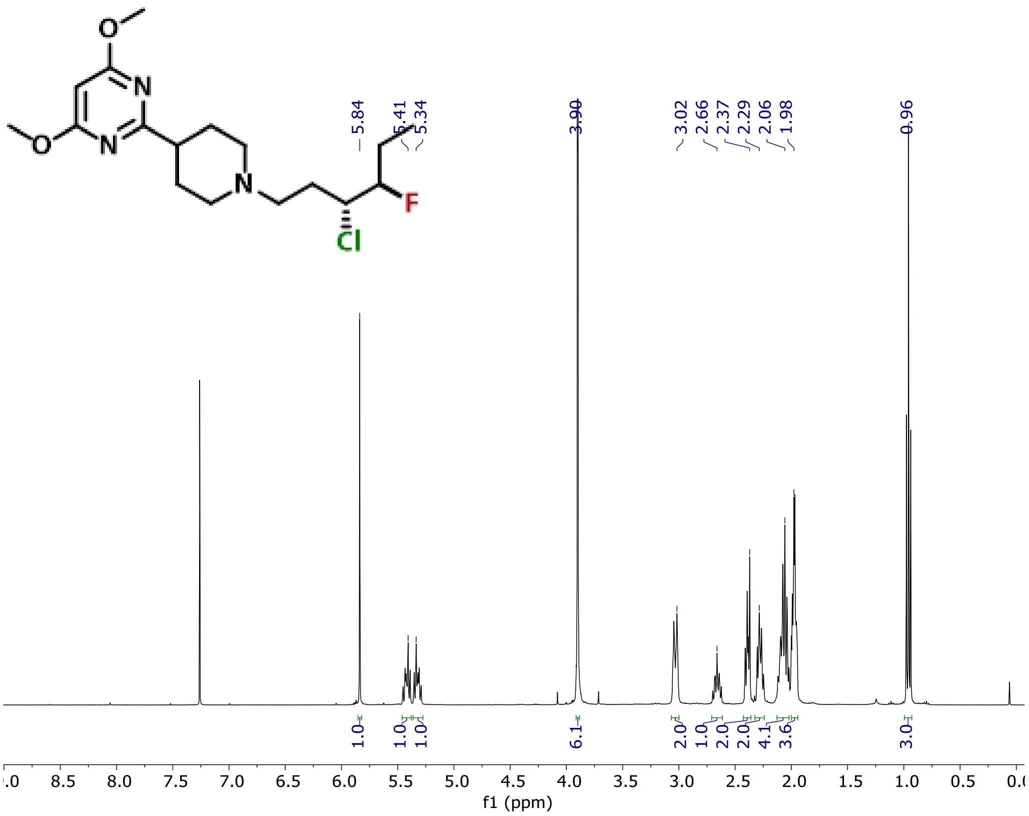

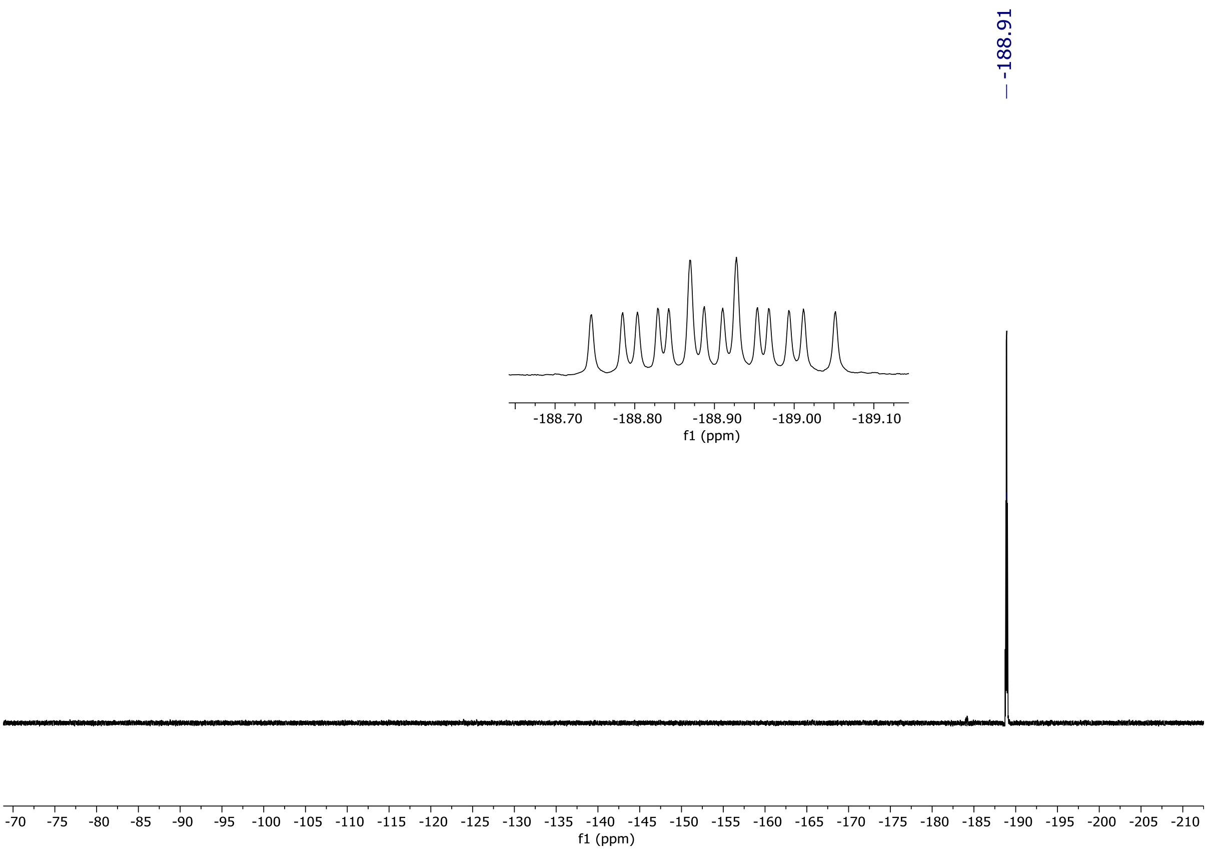

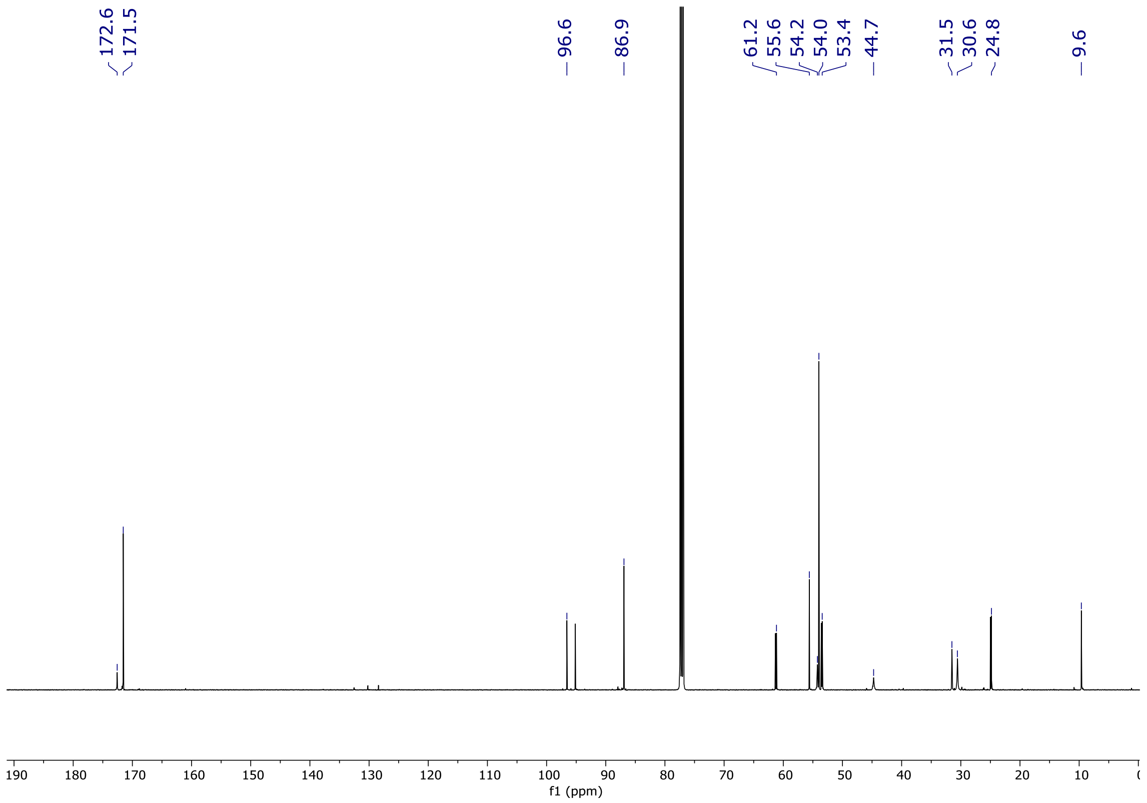
172.6
171.5
96.6
86.9
61.2
55.6
54.2
54.0
53.4
44.7
31.5
30.6
24.8
9.6
190
180
170
160
150
140
130
120
110
100
90
80
70
60
50
40
30
20
10
f1 (ppm)

6-chloro-7-fluoro-8-oxabicyclo[3.2.1]octan-3-one, 39b

^{1}H NMR (400 MHz, $CDCl_3$):

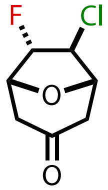

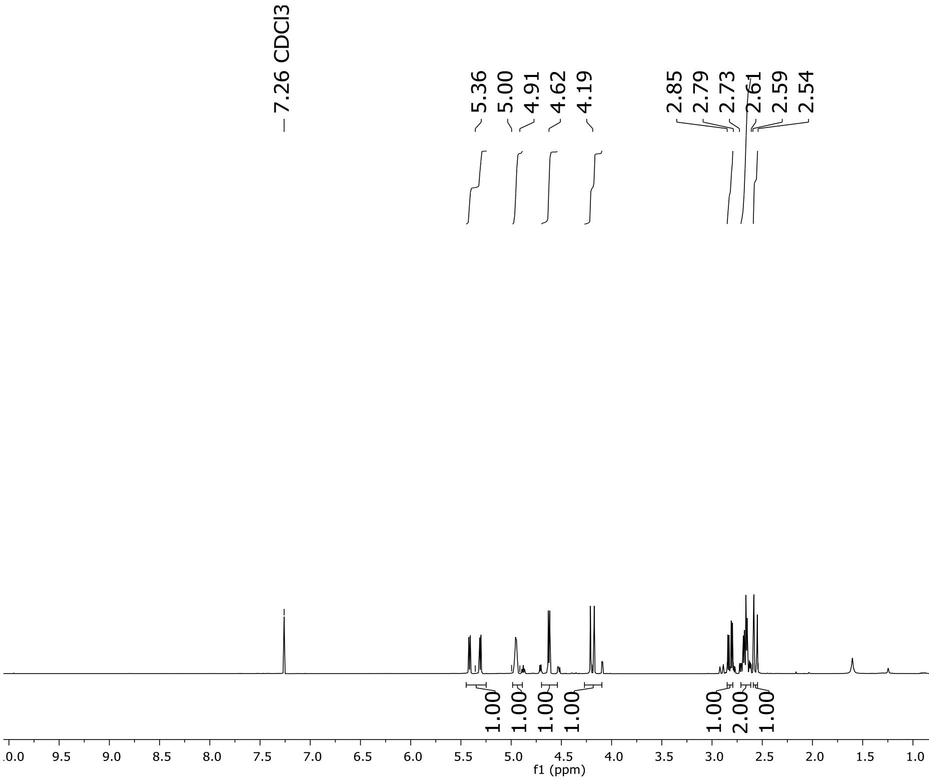

^{19}F NMR (376 MHz, $CDCl_3$):

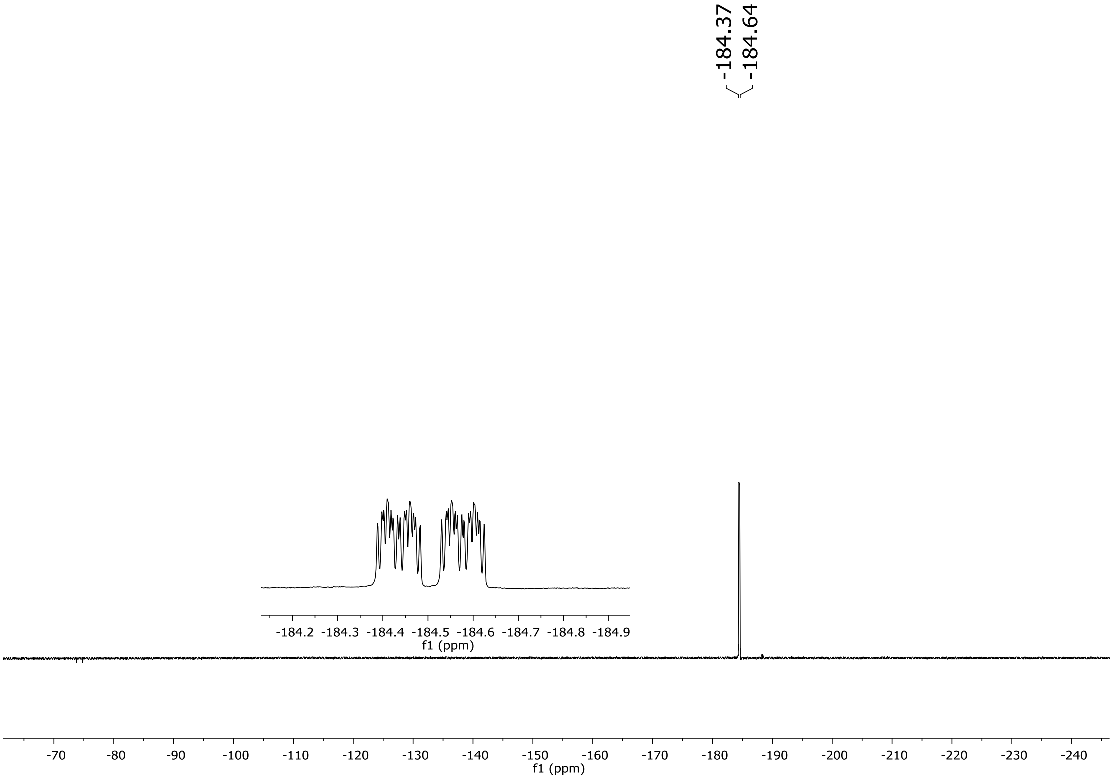

^{13}C {^{1}H} NMR (100 MHz, $CDCl_3$):

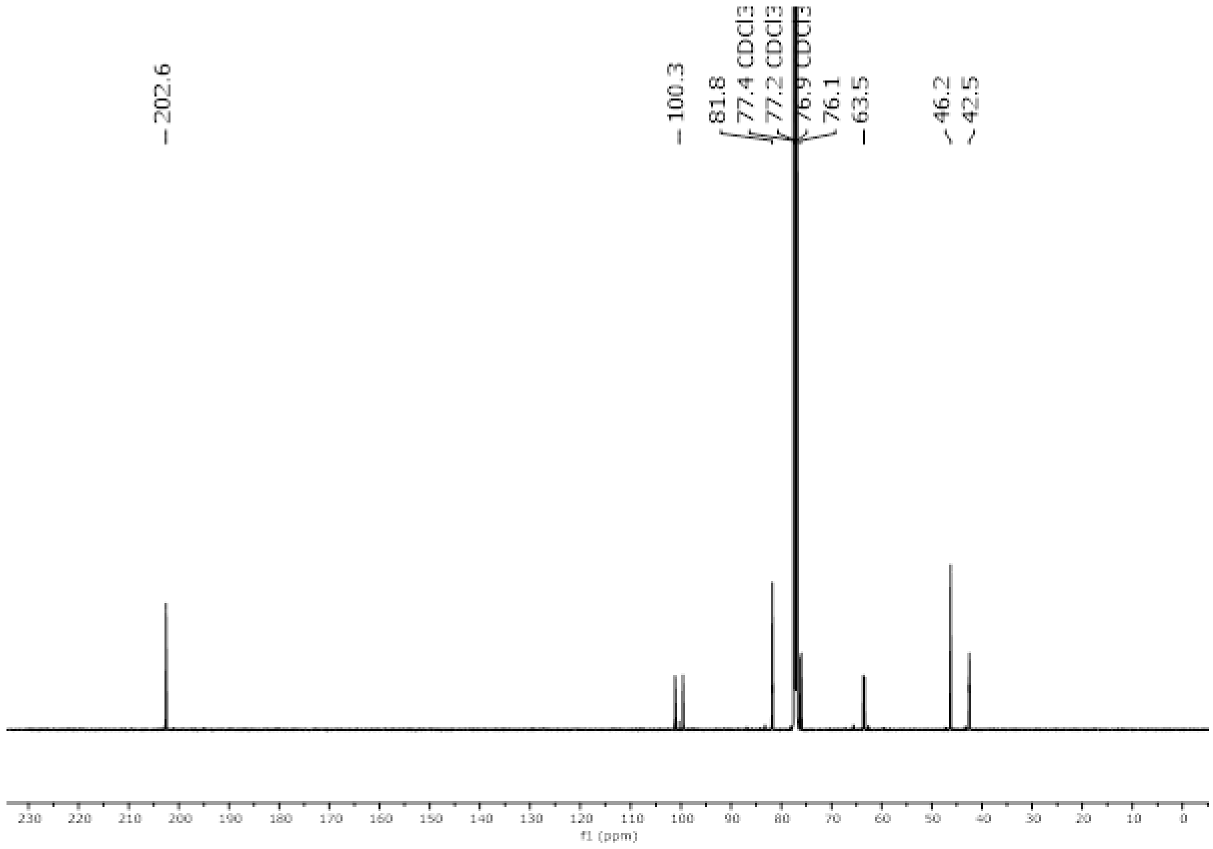
202.6
100.3
81.8
77.4 CDCl3
77.2 CDCl3
76.9 CDCl3
76.1
63.5
46.2
42.5
230
220
210
200
190
180
170
160
150
140
130
120
110
100
90
80
70
60
50
40
30
20
10
0
f1 (ppm)

NMR spectra of internal *syn*-chlorofluorination products

N-benzyl-N-((3S,4R)-3-chloro-4-fluorohexyl)-4-(trifluoromethyl)aniline, 16d

^{1}H NMR (500 MHz, $CDCl_3$):

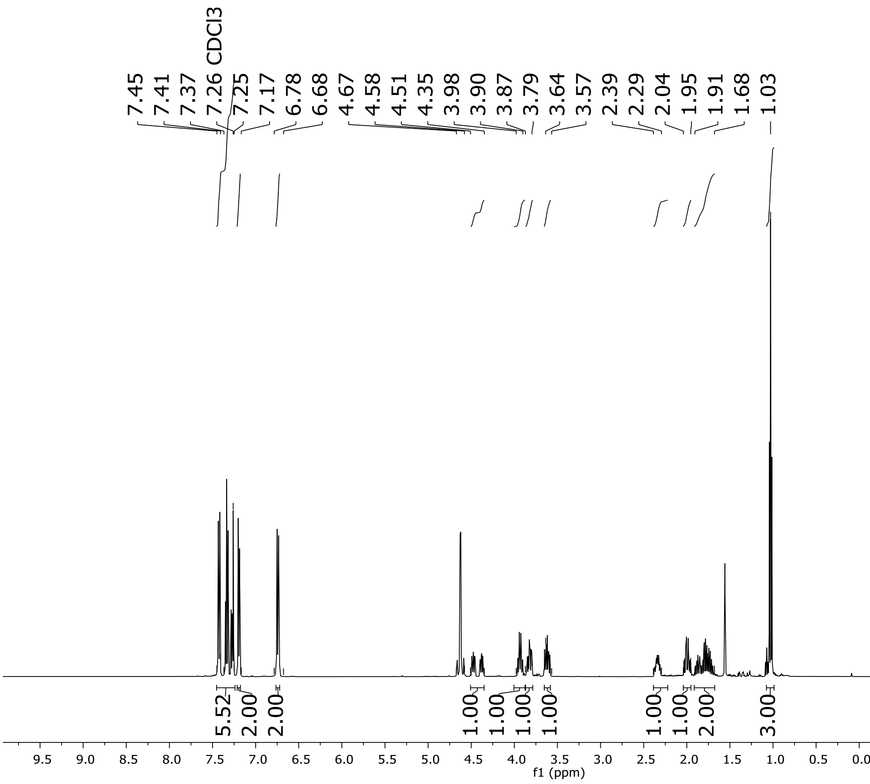

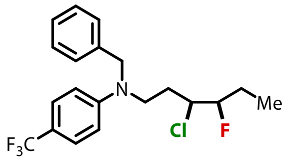

^{19}F NMR (376 MHz, $CDCl_3$):

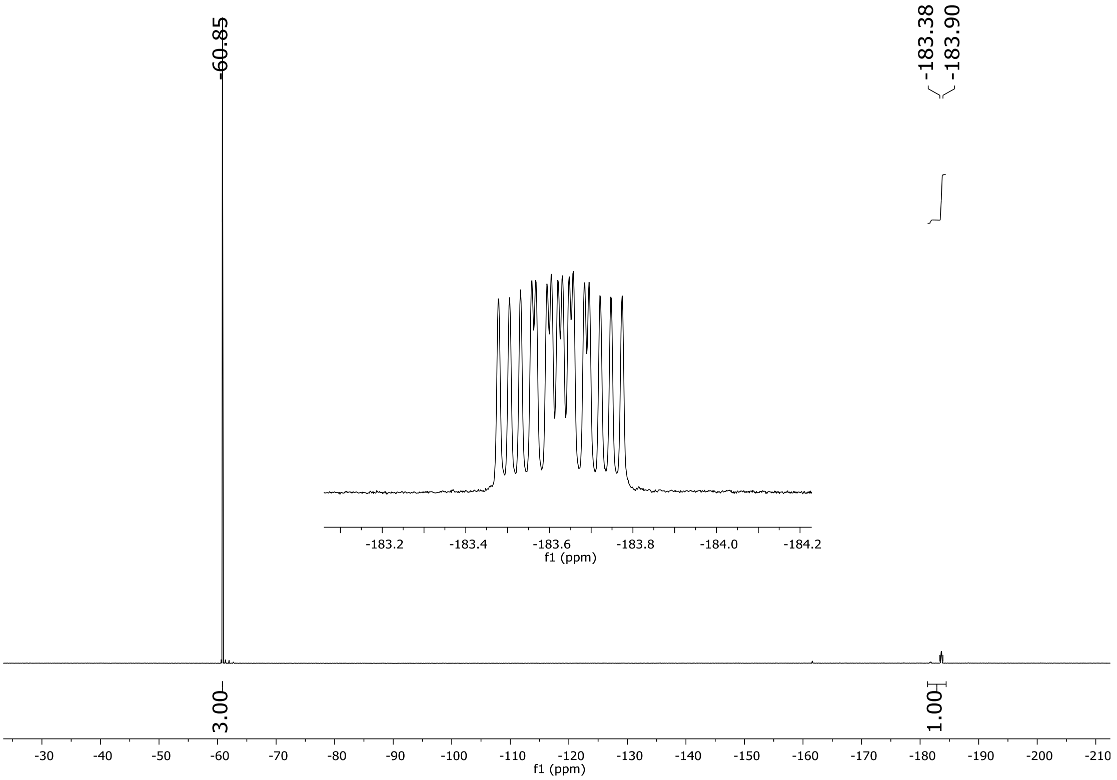

^{13}C {^{1}H} NMR (125 MHz, CDCl$_3$):

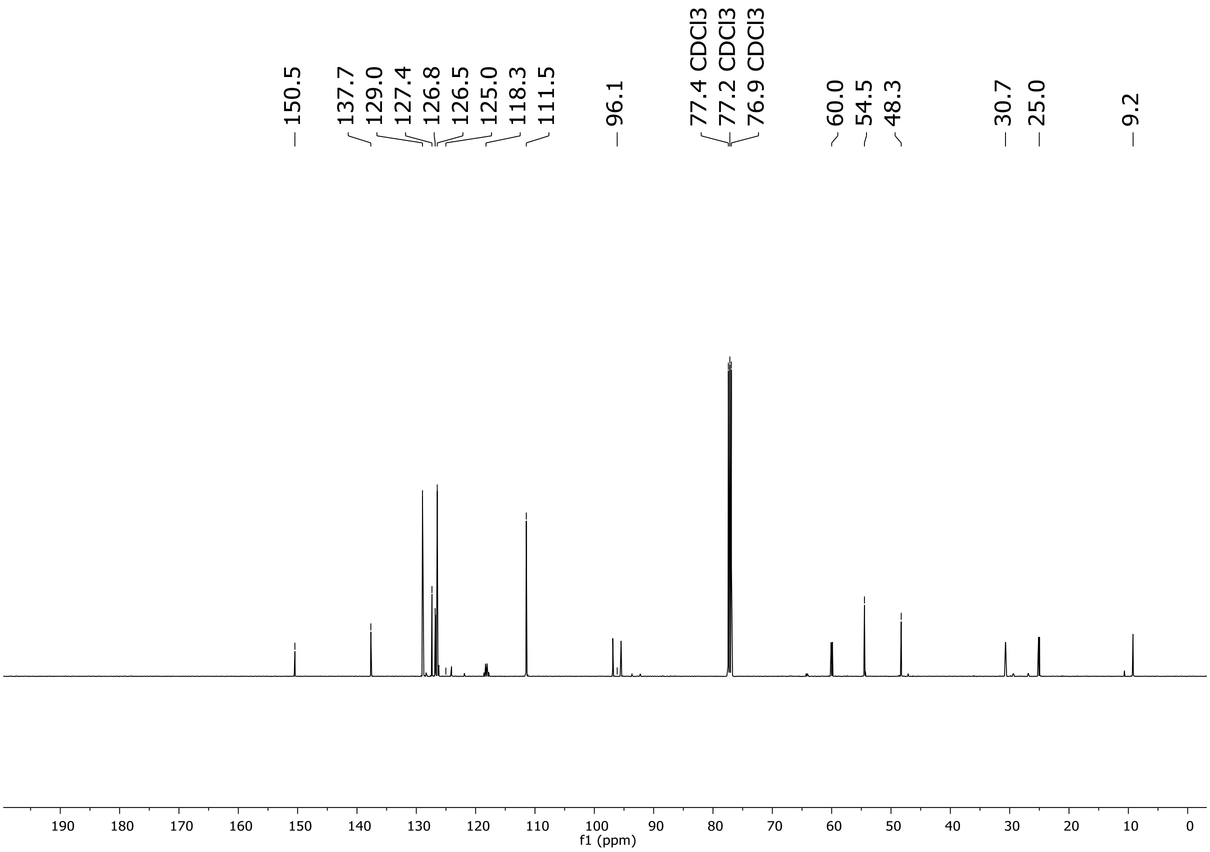

N-benzyl-N-((3S,4R)-3-chloro-4-fluorohexyl)-4-iodoaniline, 17d

^{1}H NMR (500 MHz, CDCl$_3$):

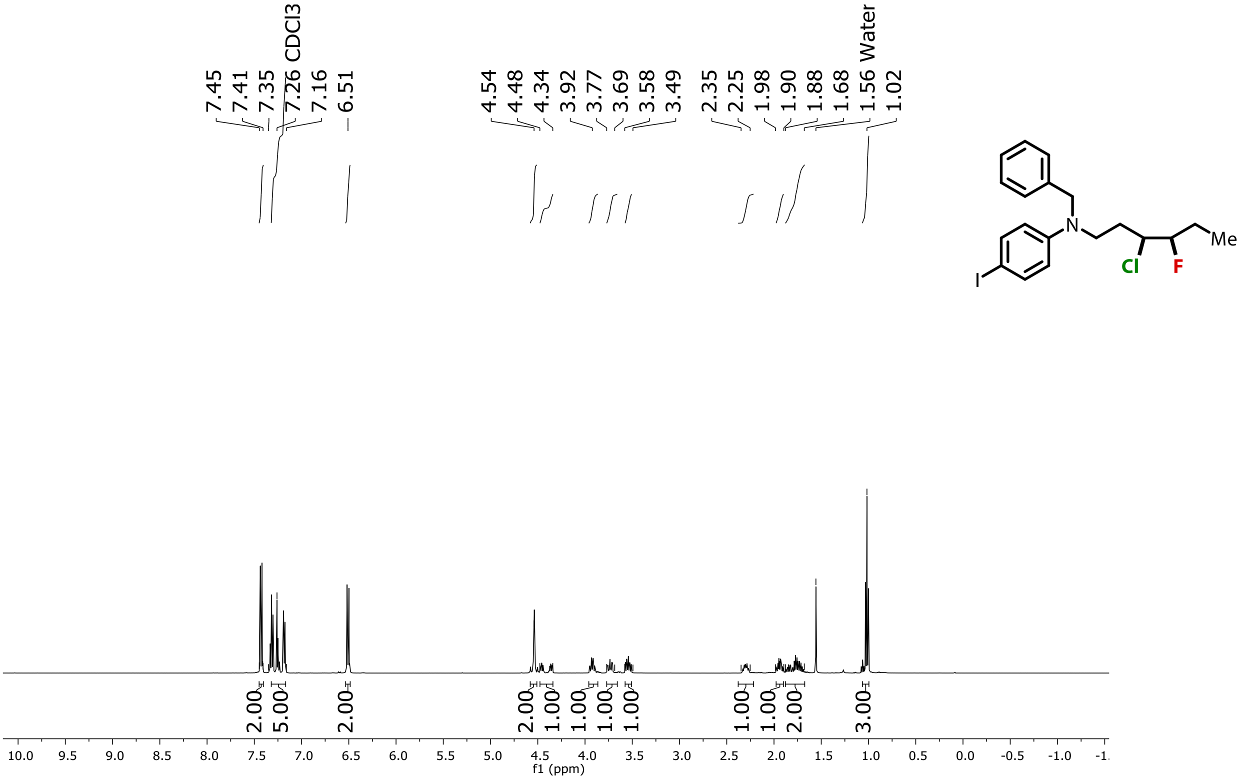

^{19}F NMR (376 MHz, CDCl$_3$):

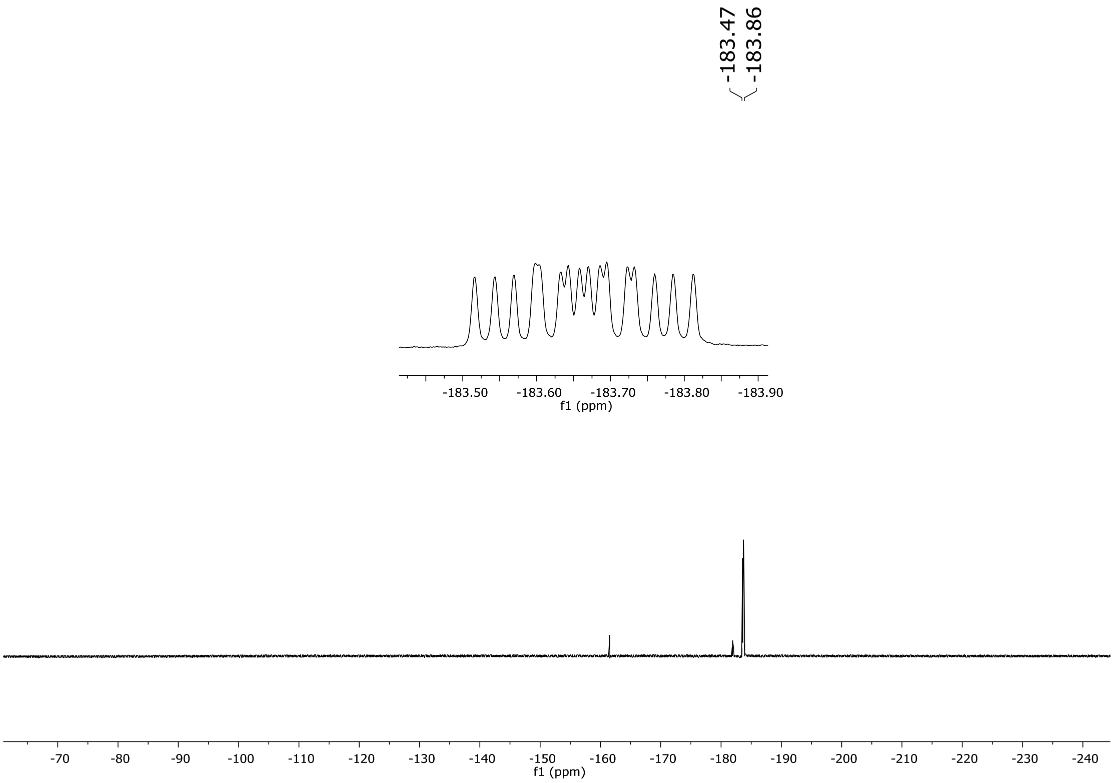

^{13}C {^{1}H} NMR (125 MHz, CDCl$_3$):

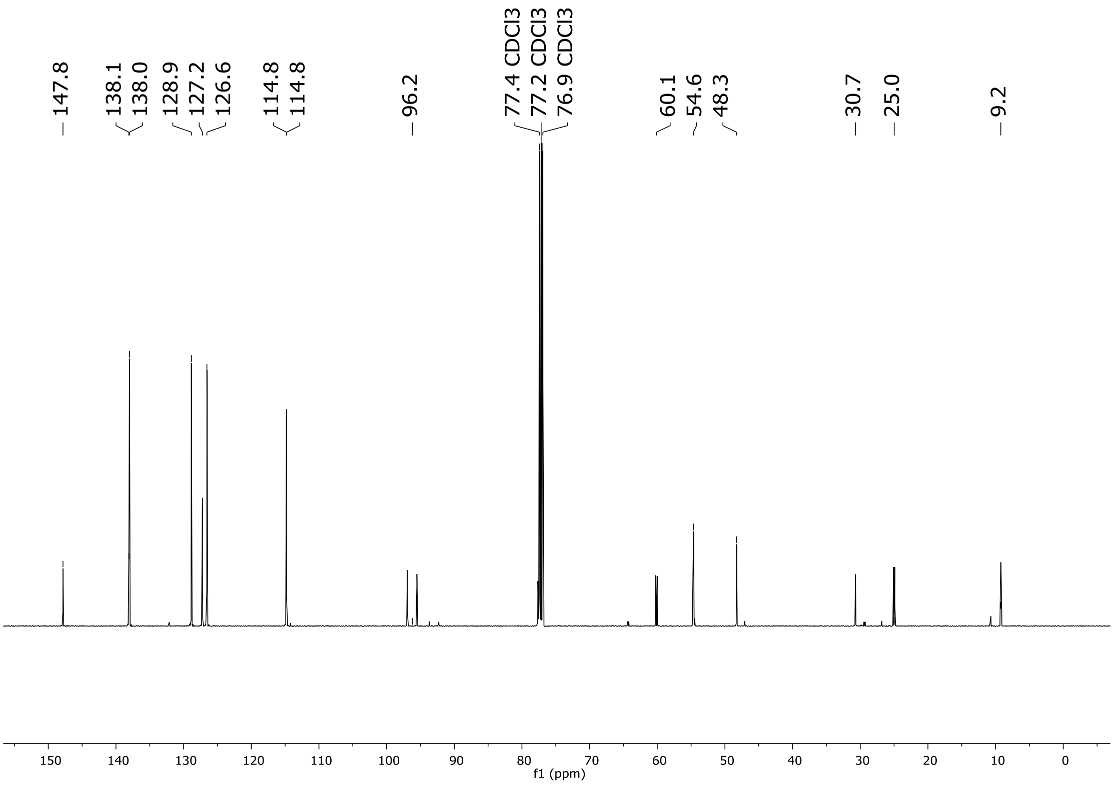

N-benzyl-N-((3S,4R)-3-chloro-4-fluorohexyl)-4-fluoroaniline, 1d

^{1}H NMR (500 MHz, CDCl$_3$):

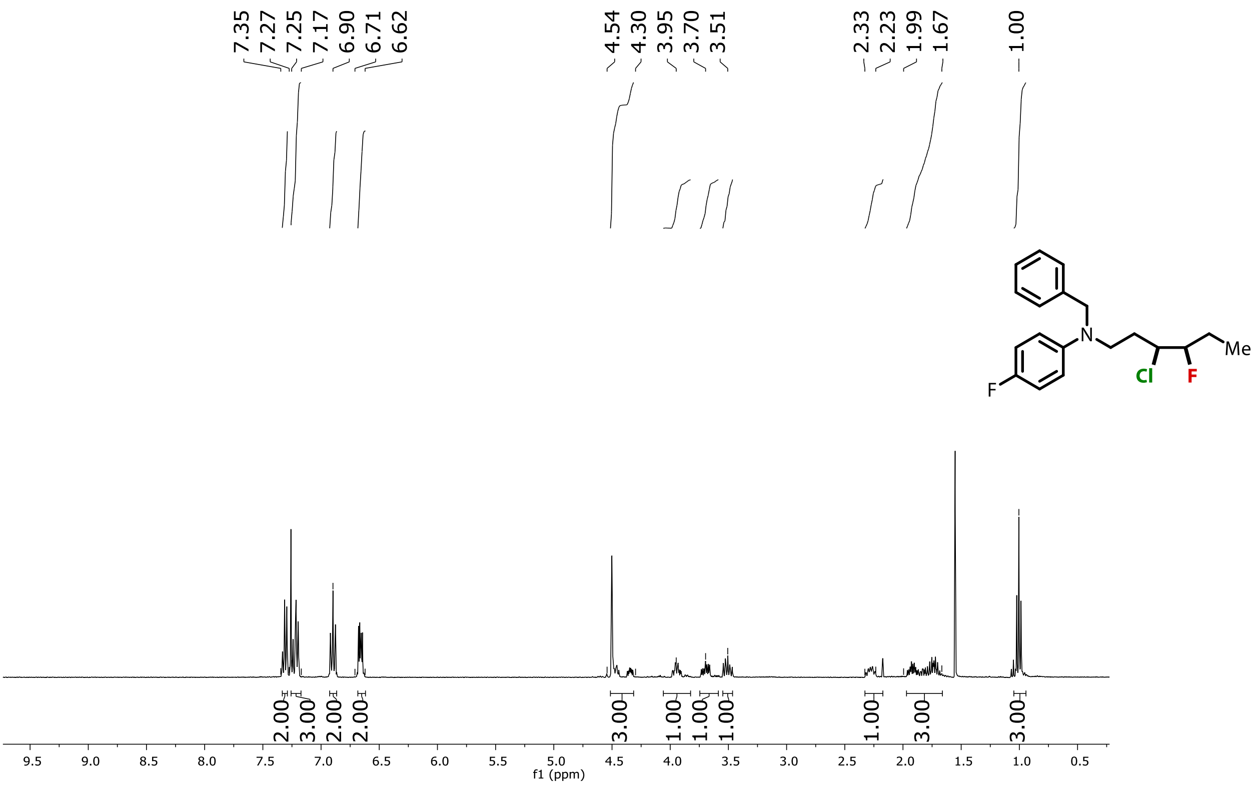

^{19}F NMR (376 MHz, CDCl$_3$):

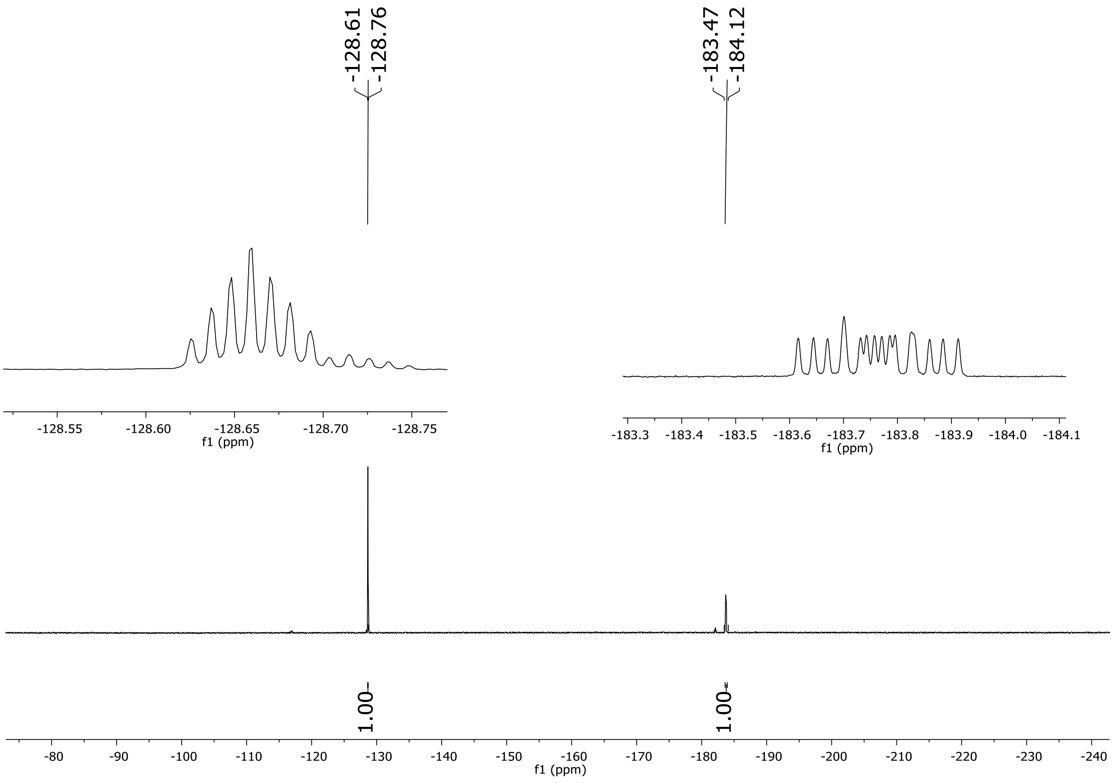

^{13}C NMR (100 MHz, $CDCl_3$):

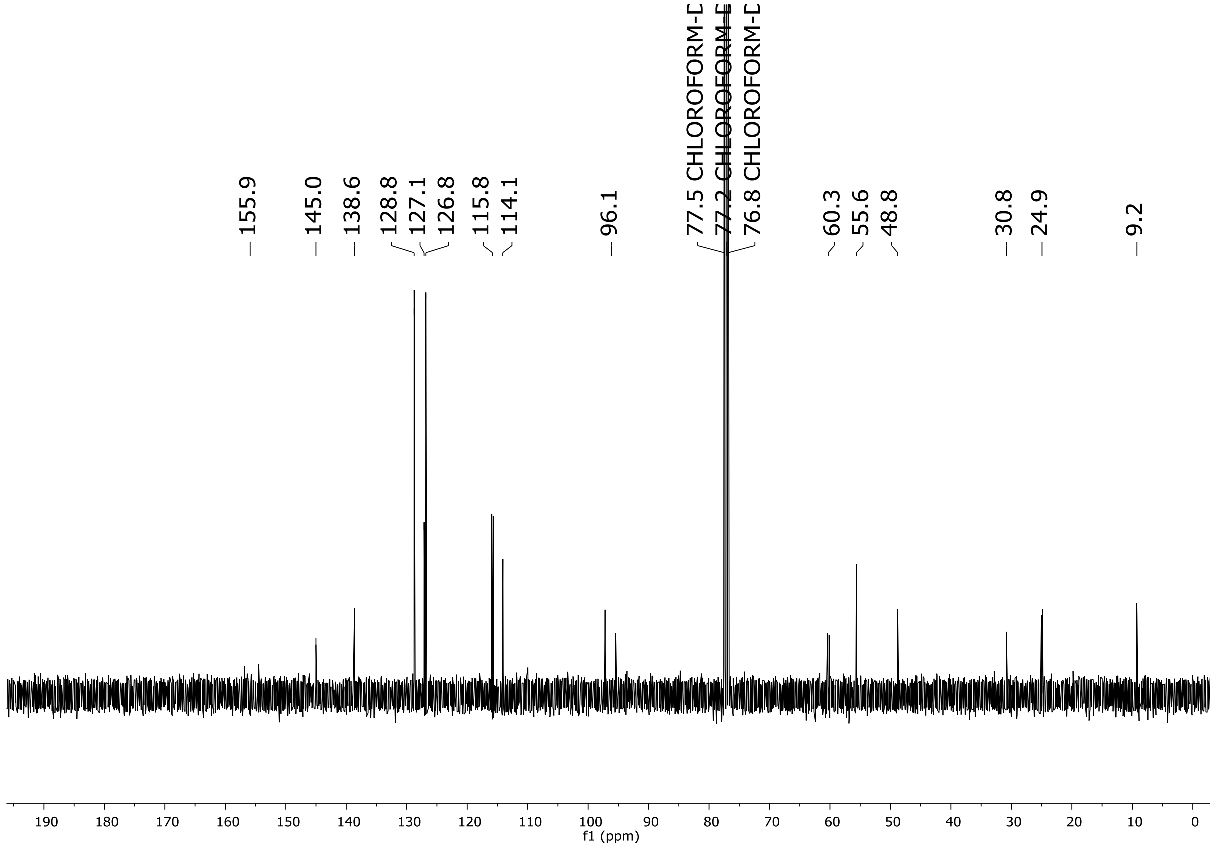

N-benzyl-N-((3S,4R)-3-chloro-4-fluorohexyl)aniline, 18d

^{1}H NMR (400 MHz, $CDCl_3$):

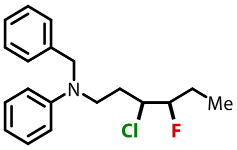

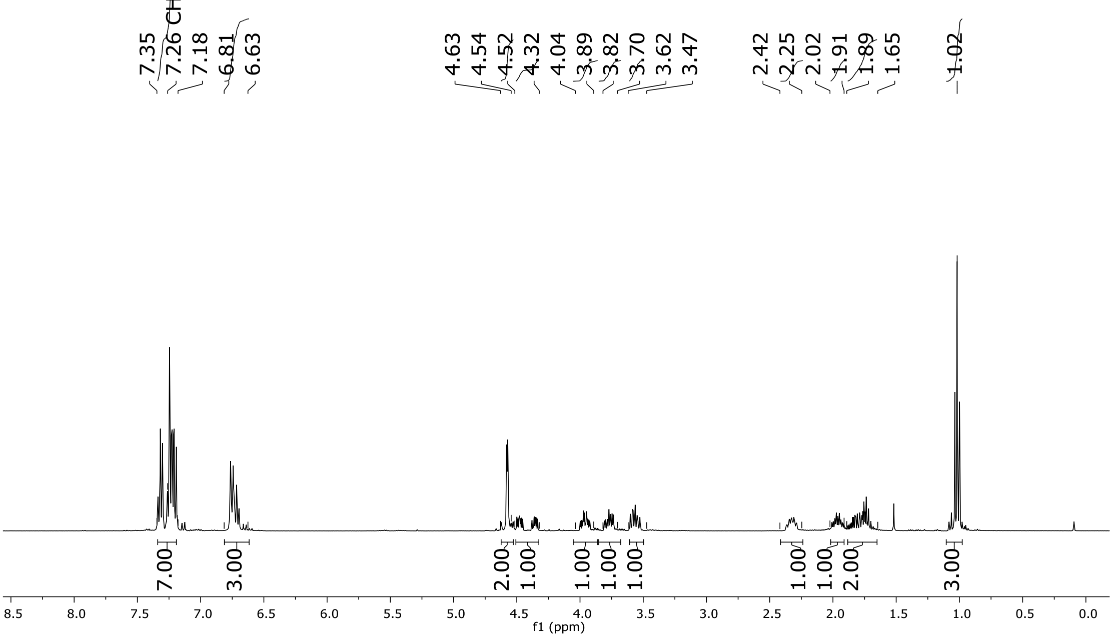

^{19}F NMR (376 MHz, $CDCl_3$):

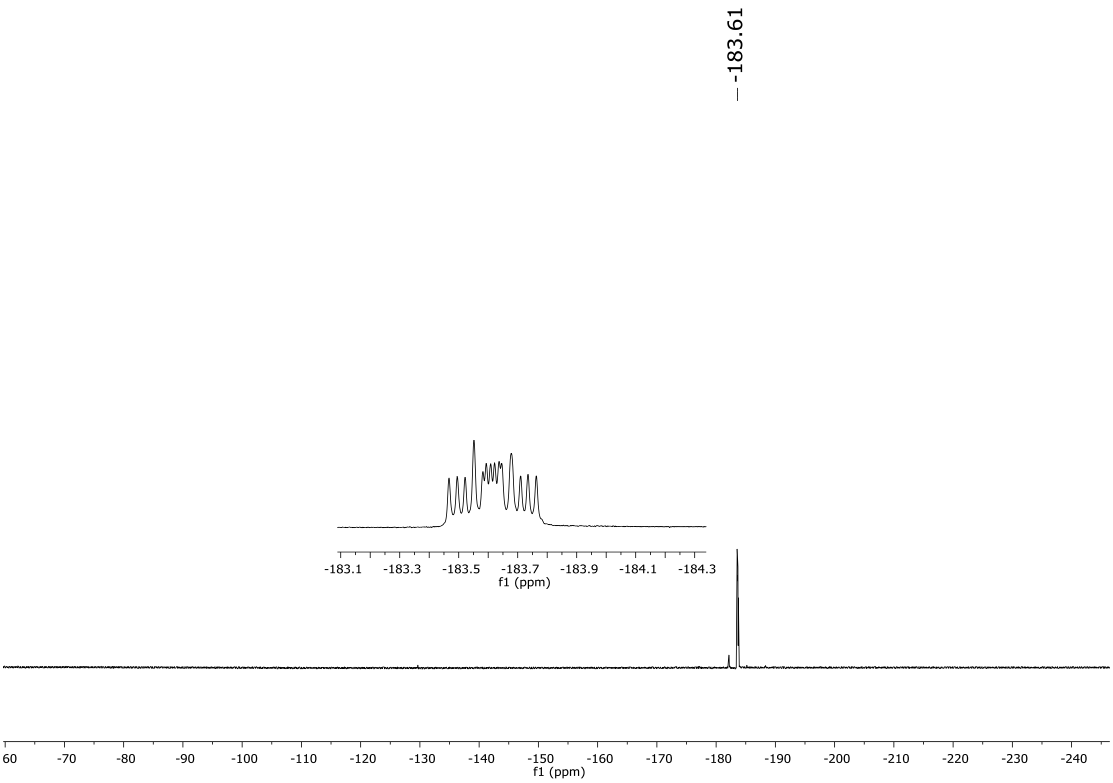

^{13}C {^{1}H} NMR (100 MHz, $CDCl_3$):

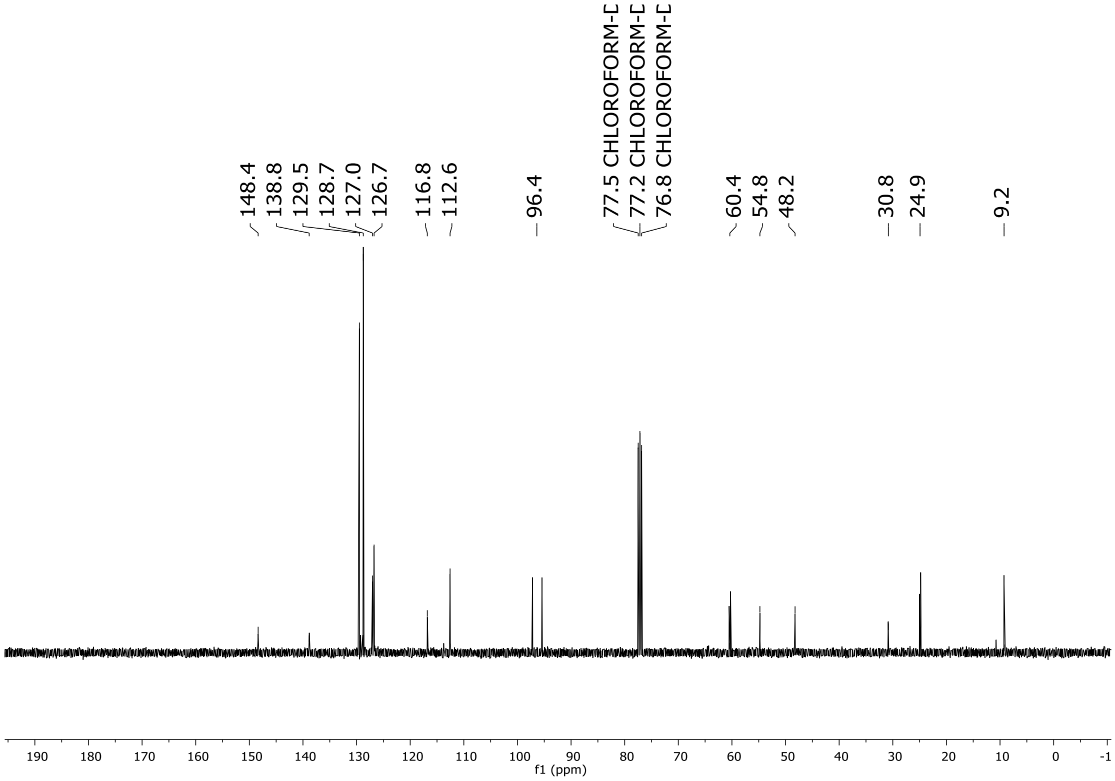

N-benzyl-N-((3S,4R)-3-chloro-4-fluorohexyl)-4-methylaniline, 19d

^{1}H NMR (500 MHz, $CDCl_3$):

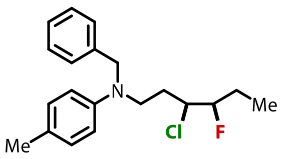

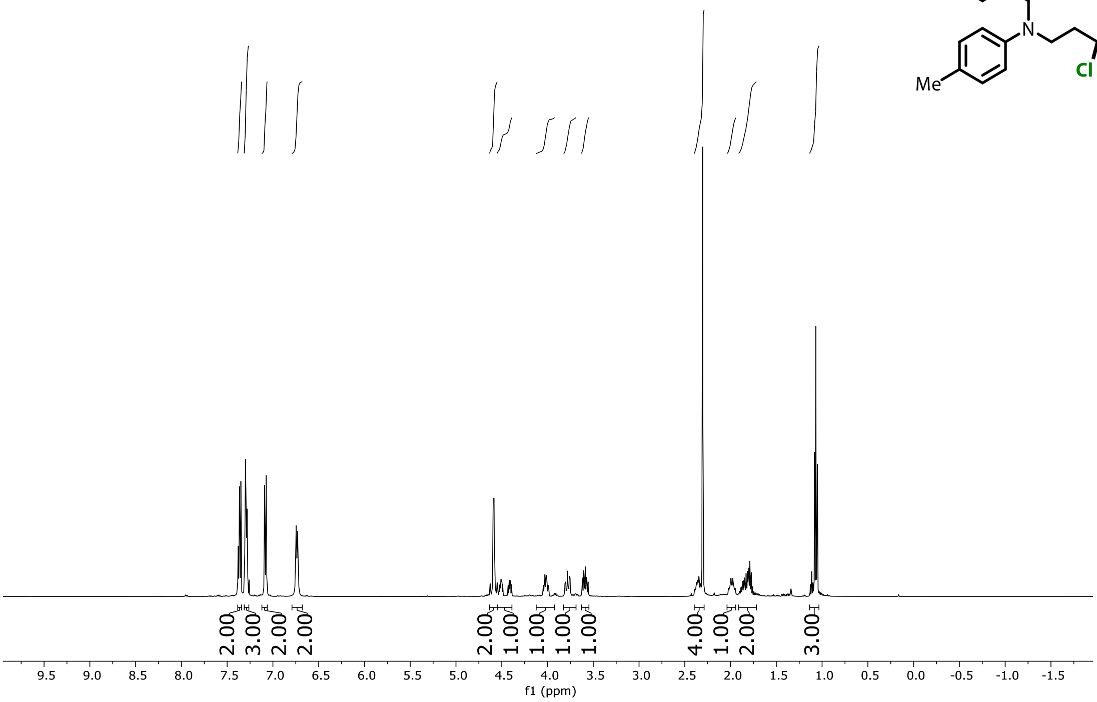

^{19}F NMR (376 MHz, $CDCl_3$):

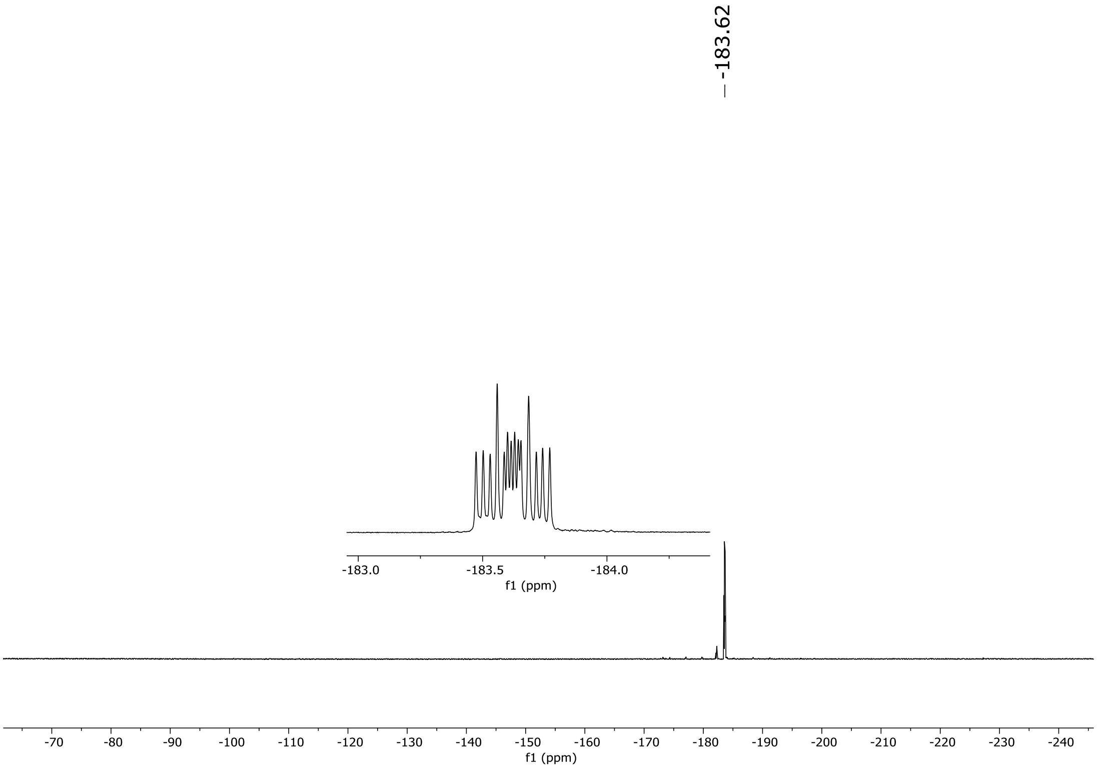

Back to top

^{13}C {^{1}H} NMR (125 MHz, CDCl$_3$):

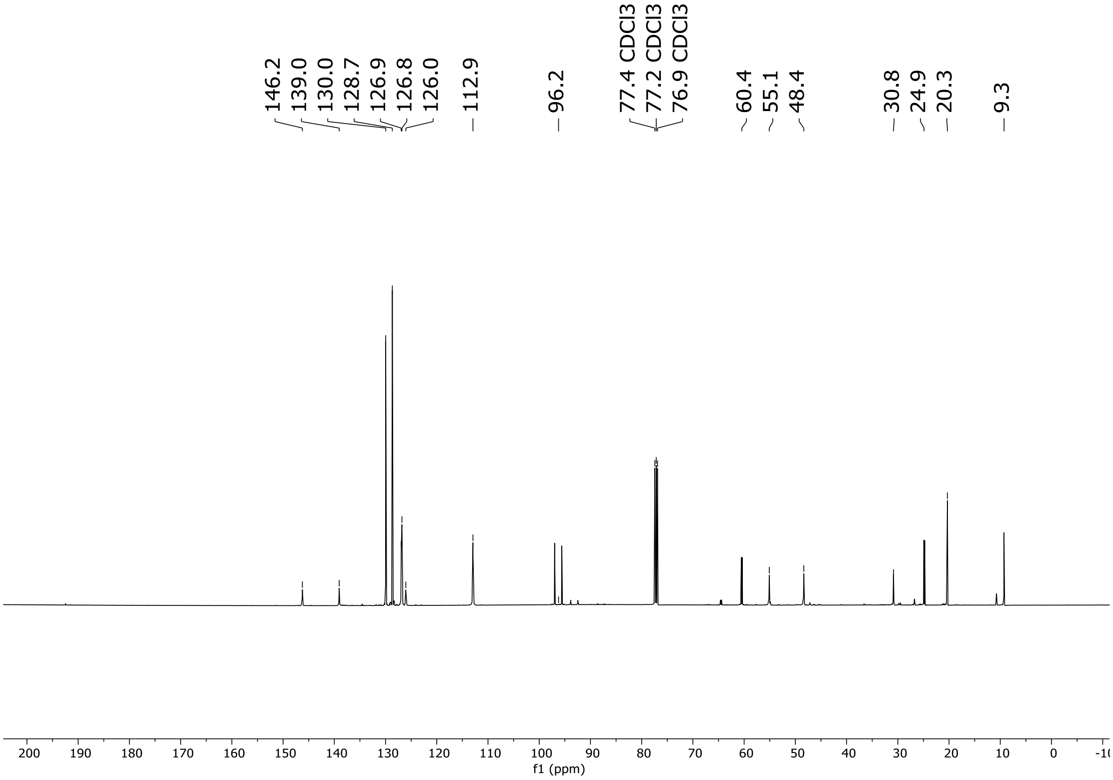

2-((3S,4R)-3-Chloro-4-fluorohexyl)isoindoline, 39d

^{1}H NMR (400 MHz, $CDCl_3$):

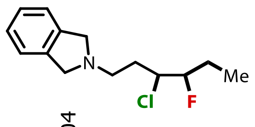

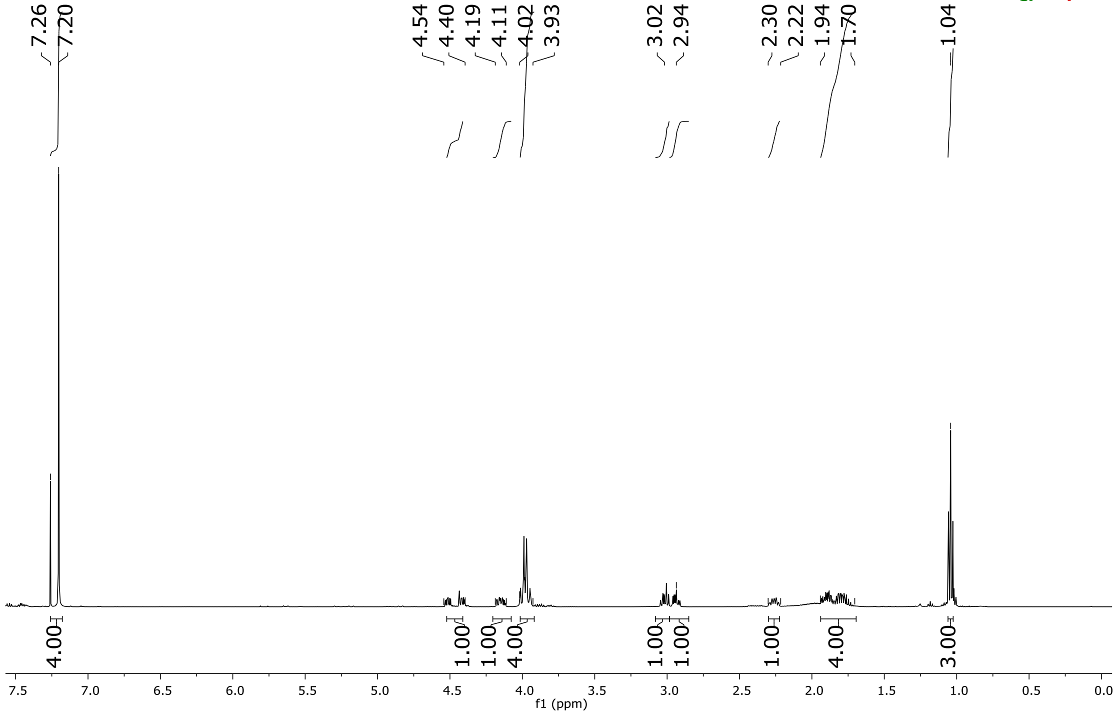

^{19}F NMR (376 MHz, $CDCl_3$):

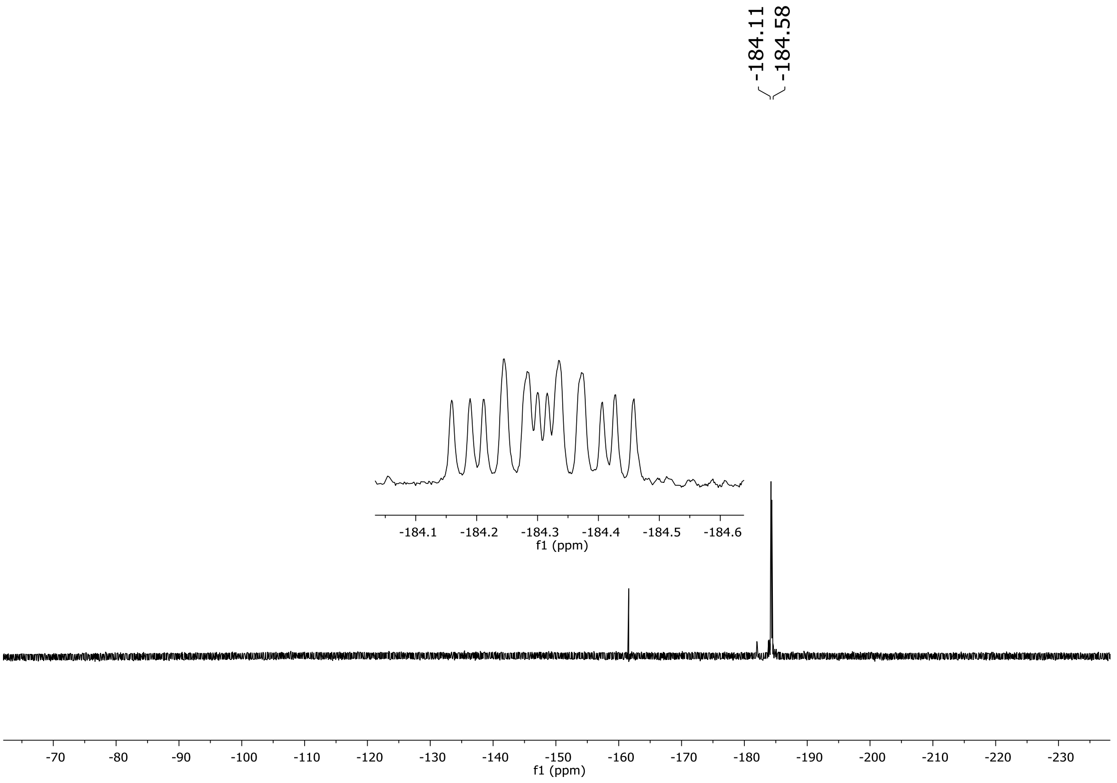

Back to top

^{13}C {^{1}H} NMR (100 MHz, CDCl$_3$):

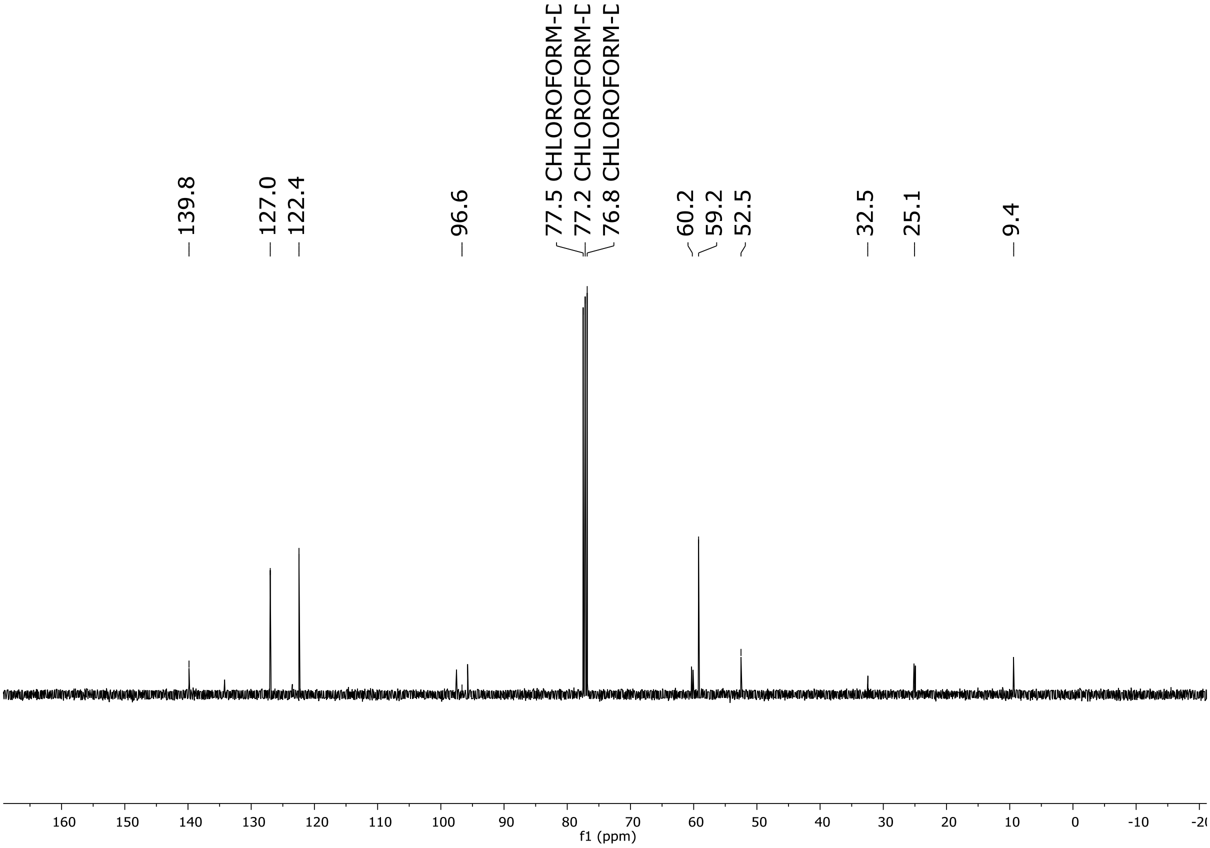

3-(1-((3S,4R)-3-chloro-4-fluorohexyl)piperidin-4-yl)-6-fluorobenzo[d]isoxazole, 41d

^{1}H NMR (400 MHz, $CDCl_3$):

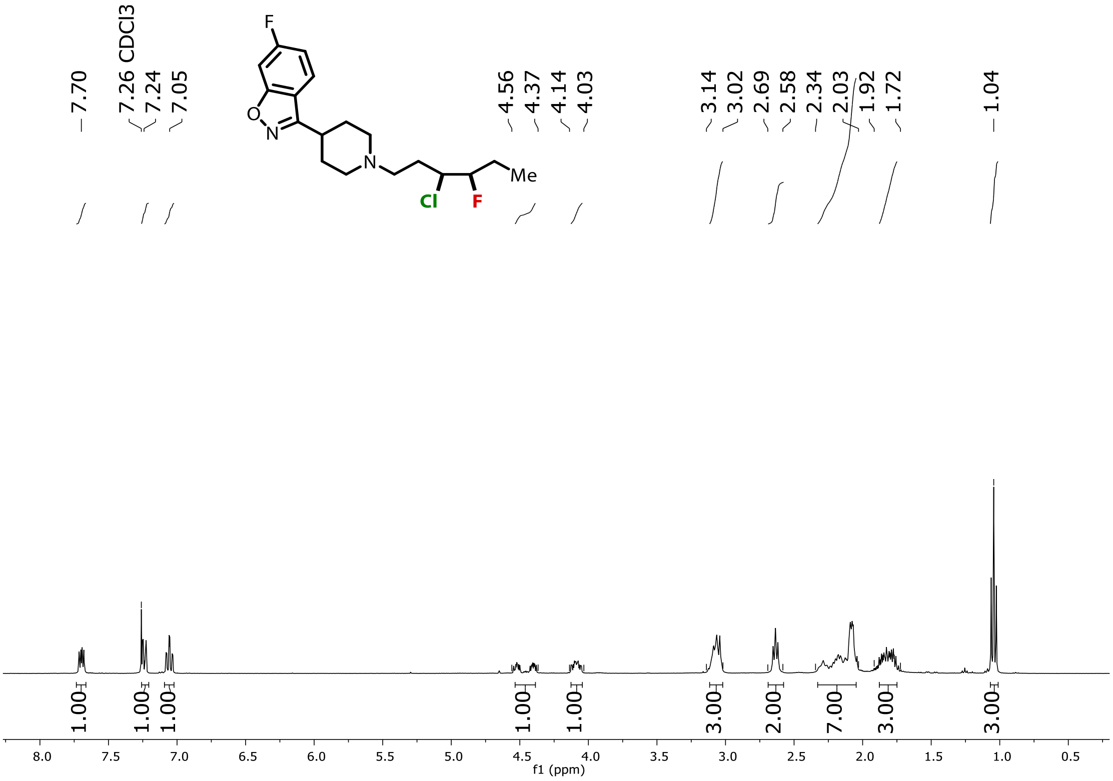

^{19}F NMR (376 MHz, $CDCl_3$):

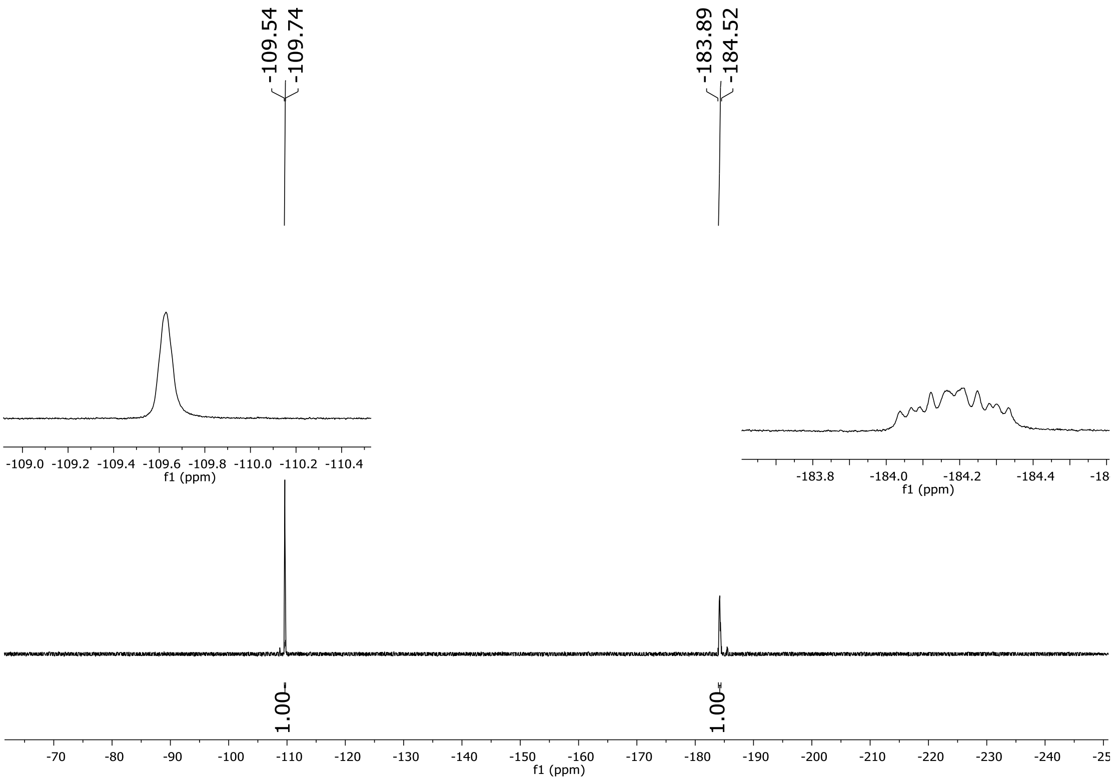

Back to top

$^{13}C\ \{^1H\}$ NMR (100 MHz, $CDCl_3$):

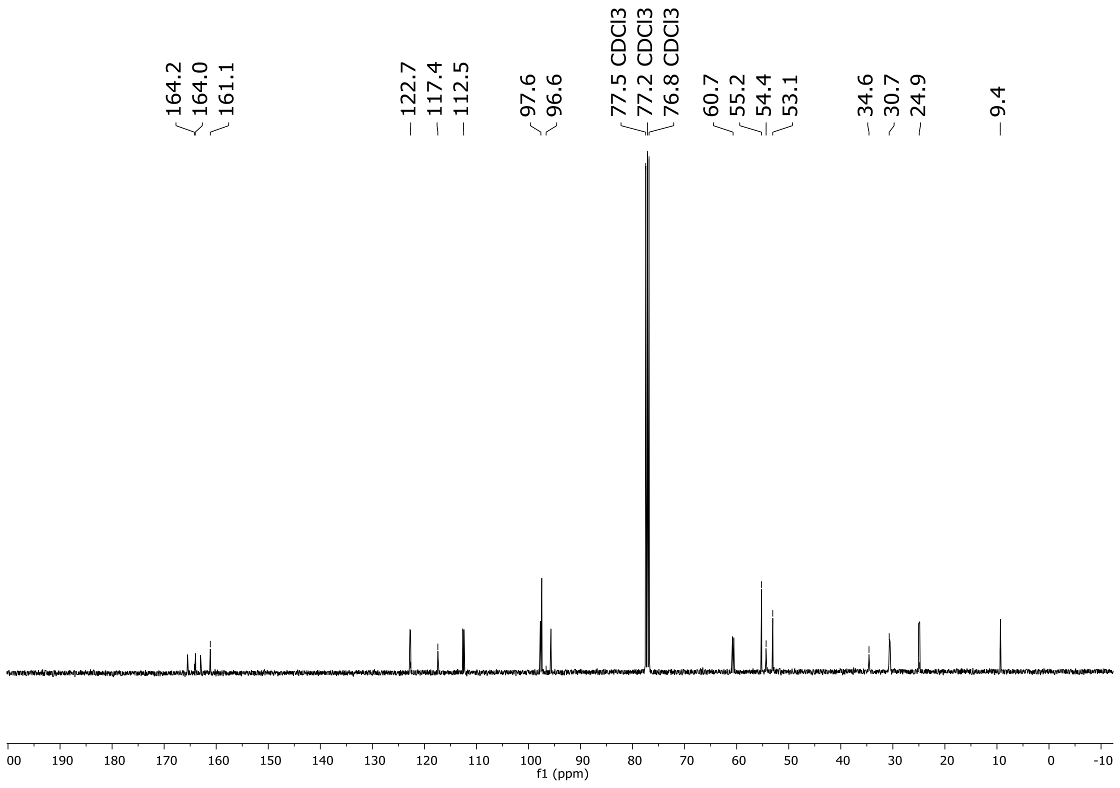

2-(1-((3R,4S)-3-chloro-4-fluorohexyl)piperidin-4-yl)-4,6-dimethoxypyrimidine, 37d

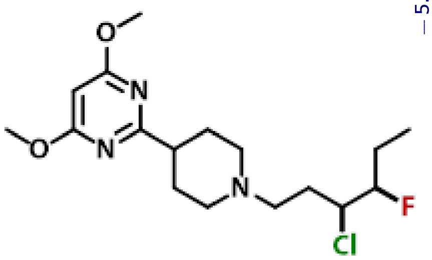

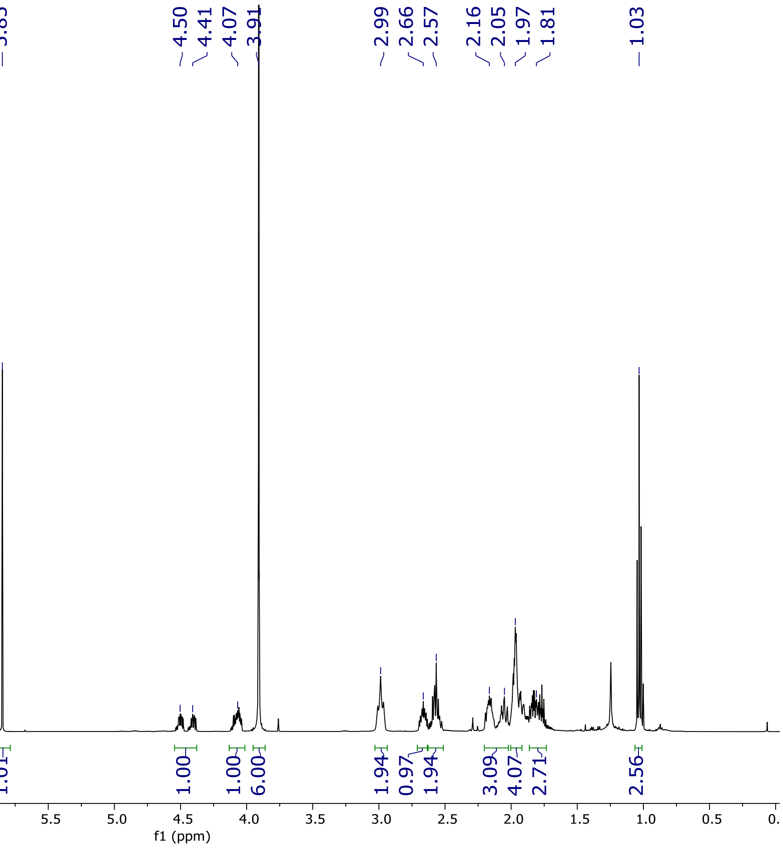

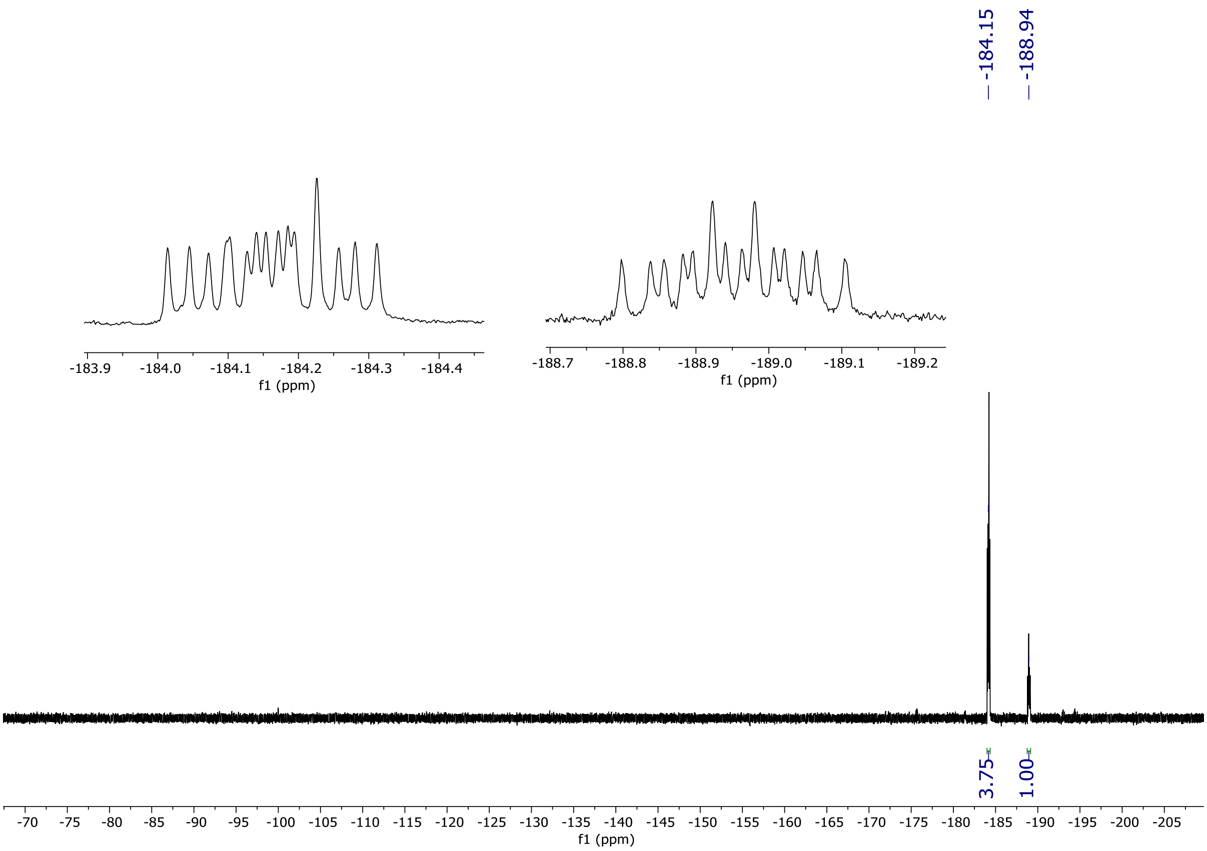

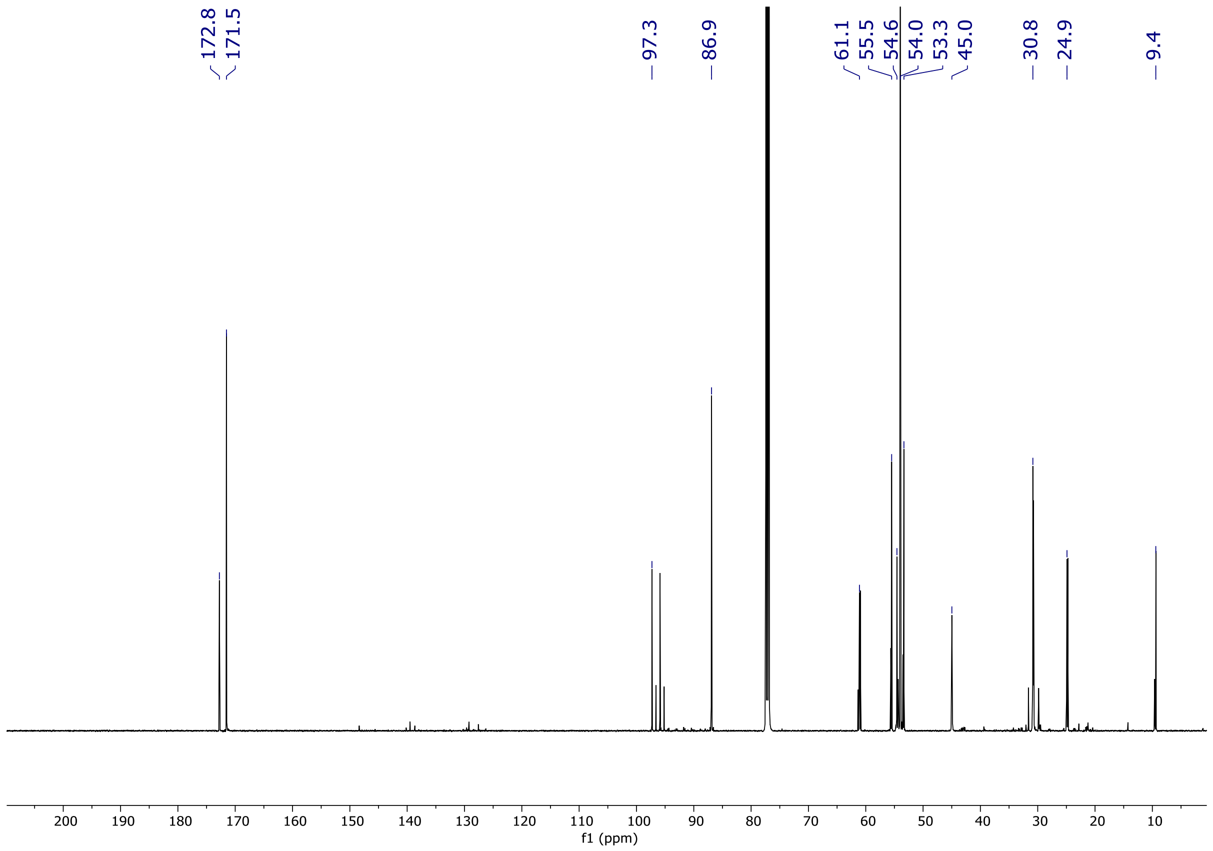
172.8
171.5
97.3
86.9
61.1
55.5
54.6
54.0
53.3
45.0
30.8
24.9
9.4
200
190
180
170
160
150
140
130
120
110
100
90
80
70
60
50
40
30
20
10
f1 (ppm)

4-((3S,4R)-3-Chloro-4-fluorohexyl)morpholine, 42d

^{1}H NMR (400 MHz, $CDCl_3$):

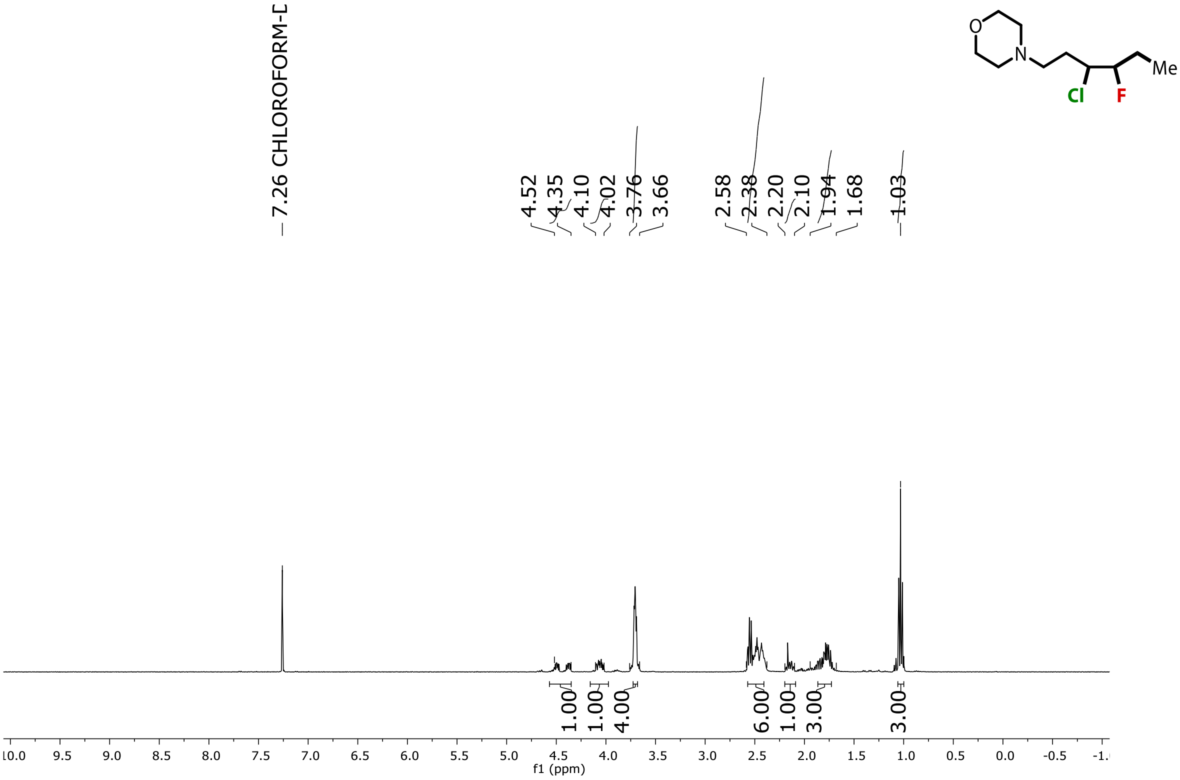

^{19}F NMR (376 MHz, $CDCl_3$):

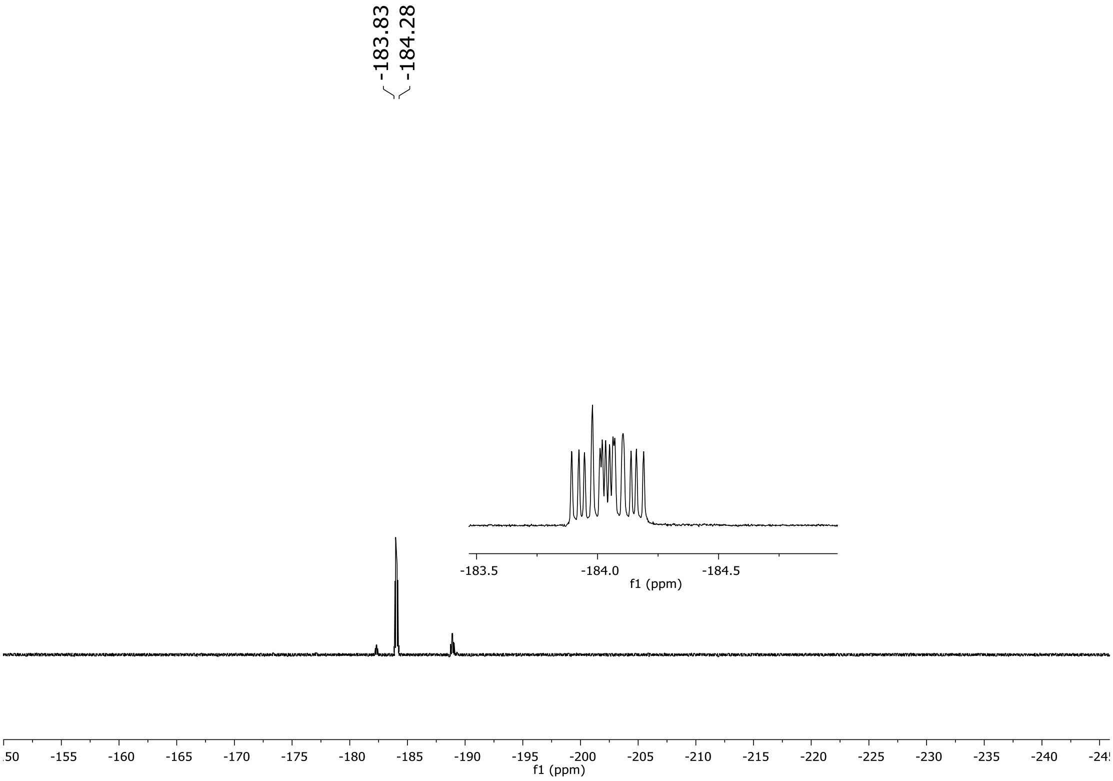

$^{13}C\ \{^{1}H\}$ NMR (100 MHz, $CDCl_3$):

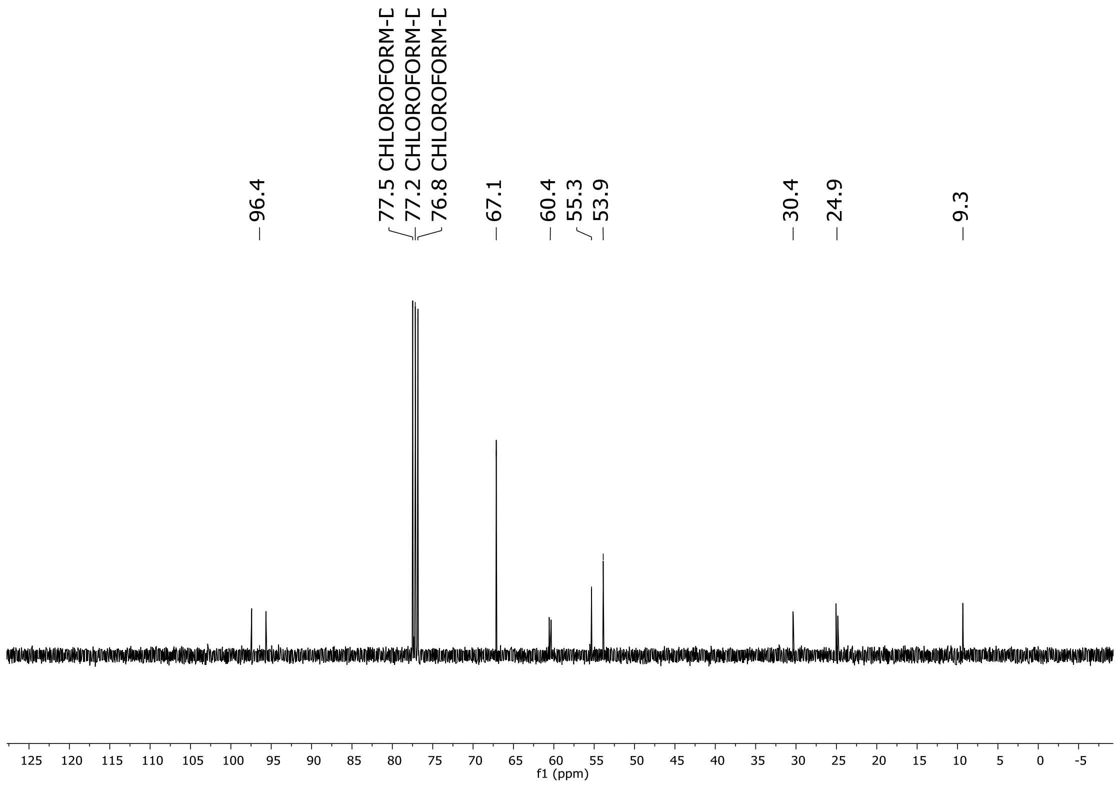

1-((3S,4R)-3-Chloro-4-fluorohexyl)-4-(4-nitrophenyl)piperazine, 43d

^{1}H NMR (500 MHz, $CDCl_3$):

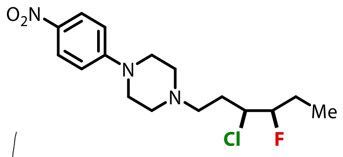

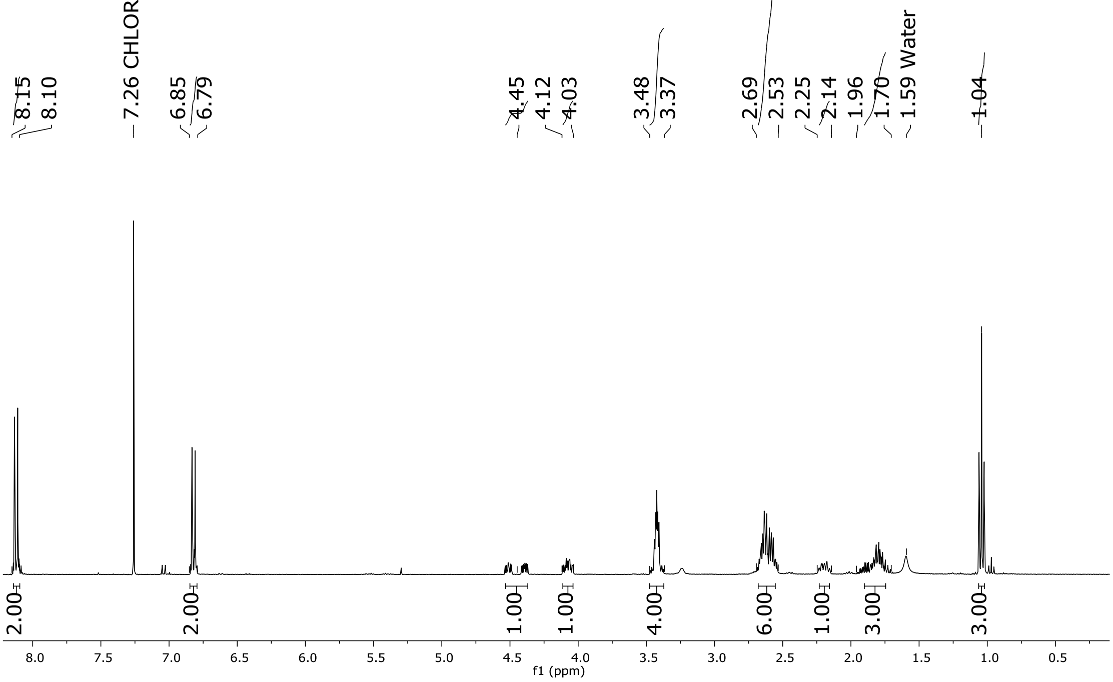

^{19}F NMR (376 MHz, $CDCl_3$):

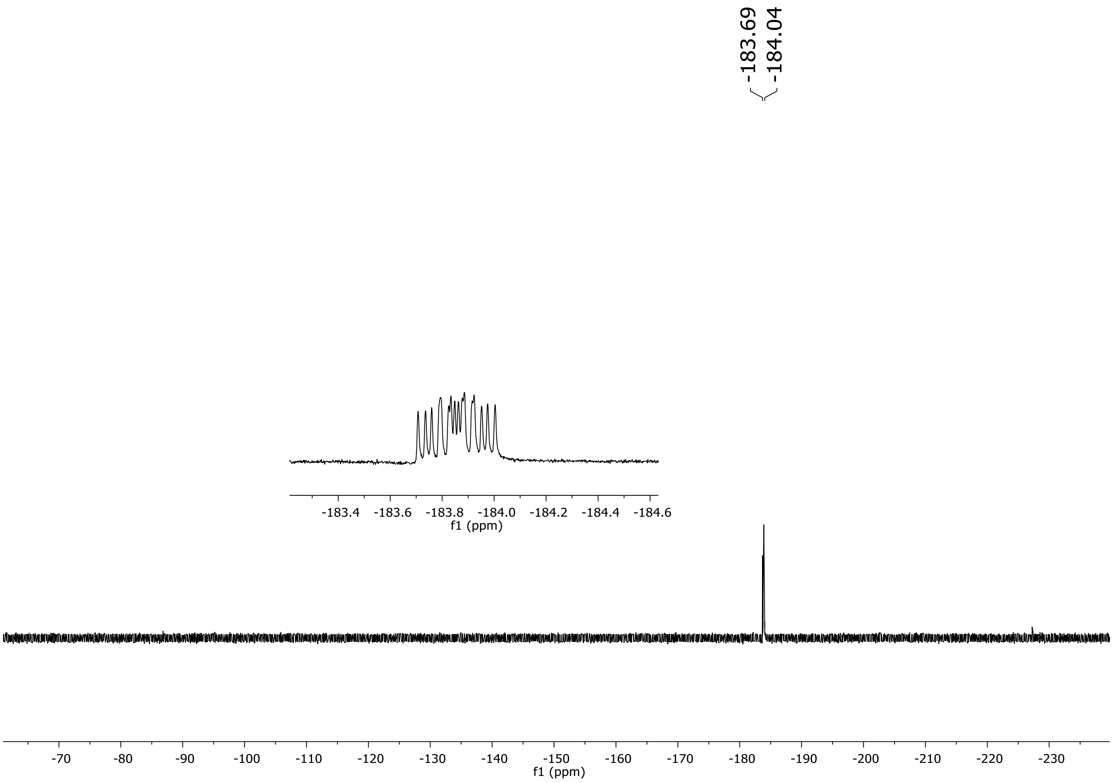

^{13}C NMR (100 MHz, CDCl$_3$):

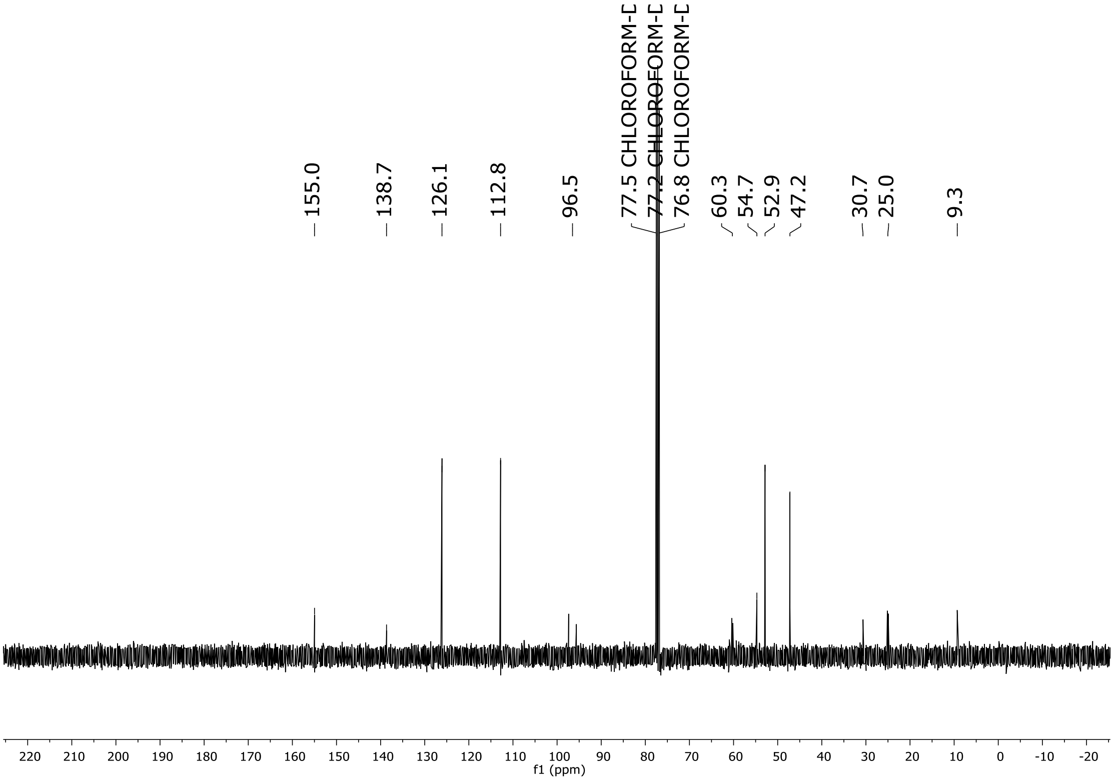

(1R,5S)-8-((3R,4R)-3-chloro-4-fluorohexyl)-8-azabicyclo[3.2.1]octan-3-one, 44d

^{1}H NMR (500 MHz, $CDCl_3$):

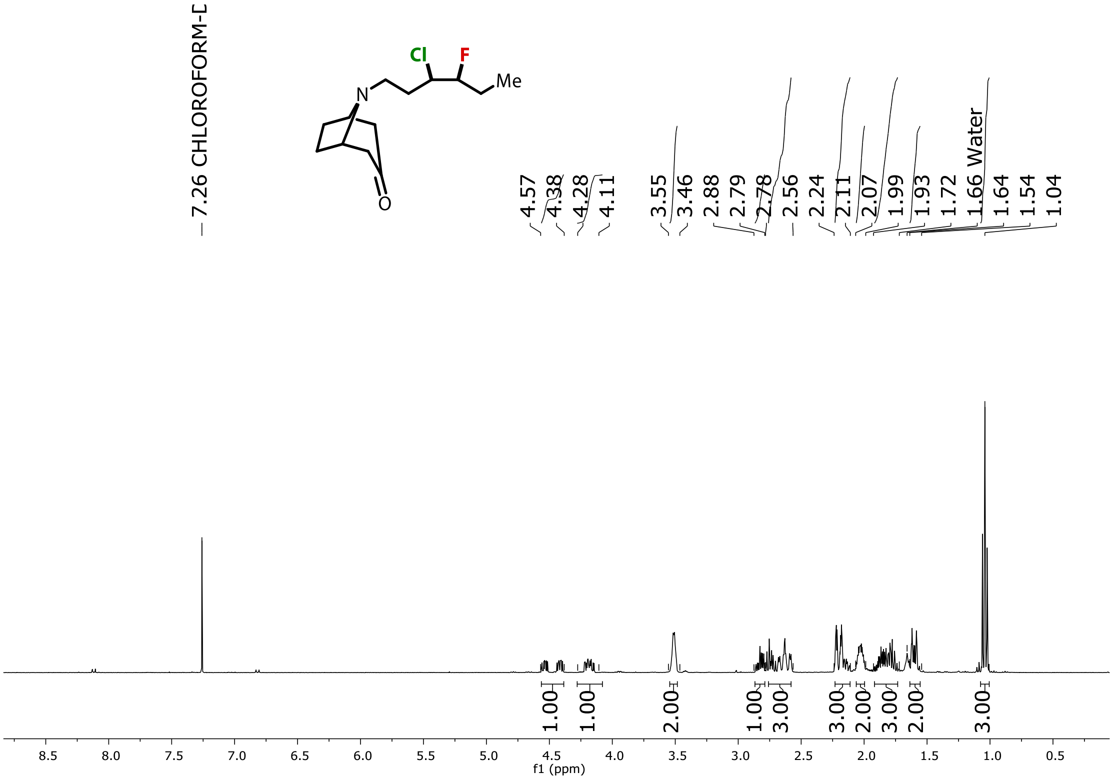

^{19}F NMR (376 MHz, $CDCl_3$):

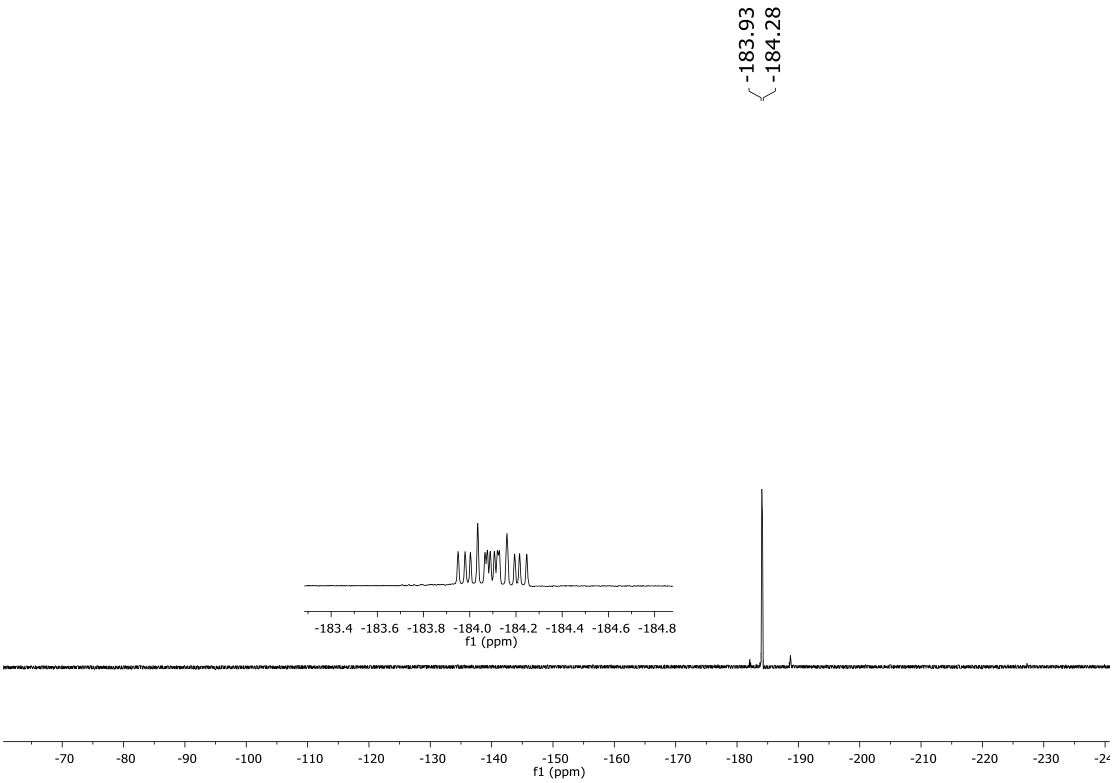

^{13}C {^{1}H} NMR (125 MHz, $CDCl_3$):

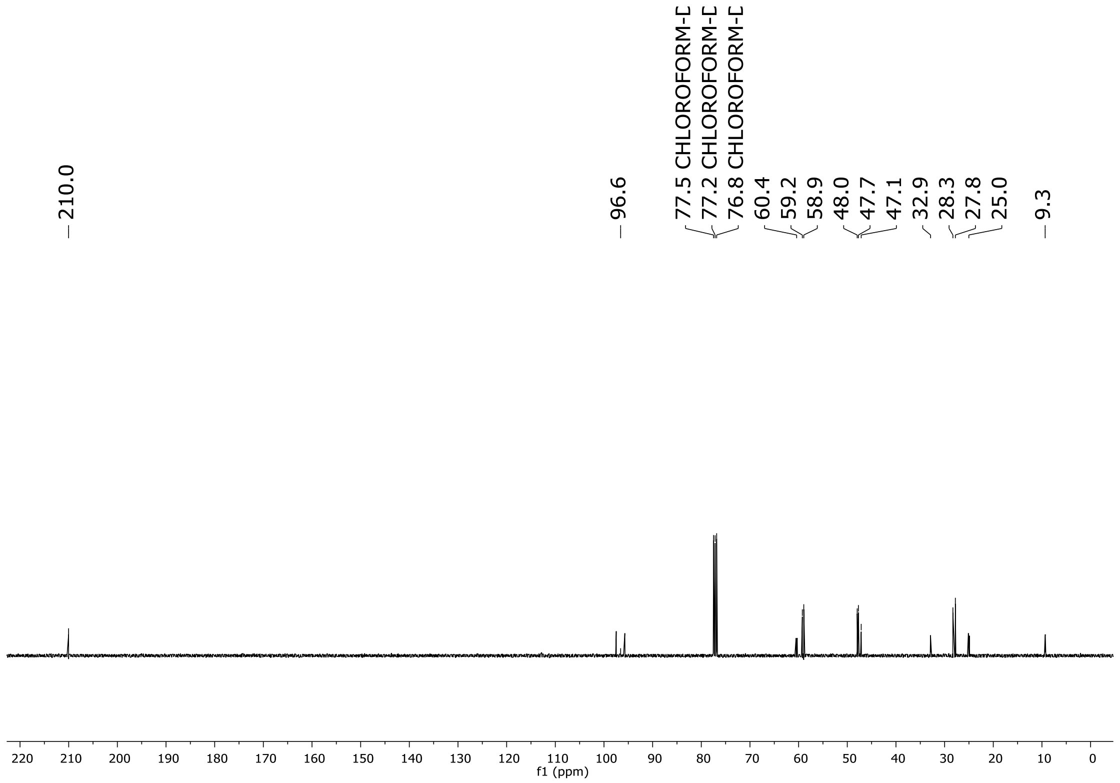
210.0
96.6
77.5 CHLOROFORM-D
77.2 CHLOROFORM-D
76.8 CHLOROFORM-D
60.4
59.2
58.9
48.0
47.7
47.1
32.9
28.3
27.8
25.0
9.3
220 210 200 190 180 170 160 150 140 130 120 110 100 90 80 70 60 50 40 30 20 10 0
f1 (ppm)

(3R,4R)-N,N-dibenzyl-3-chloro-4-fluorooctan-1-amine, 45b

^{1}H NMR (400 MHz, $CDCl_3$):

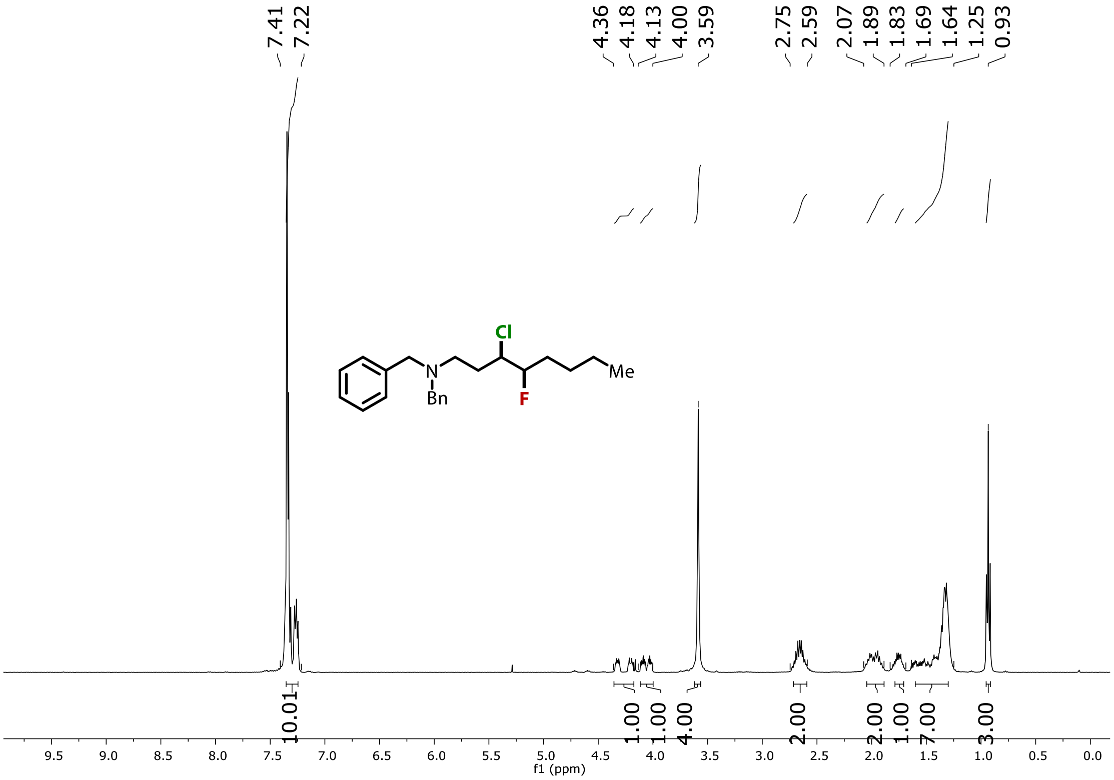

^{19}F NMR (376 MHz, $CDCl_3$):

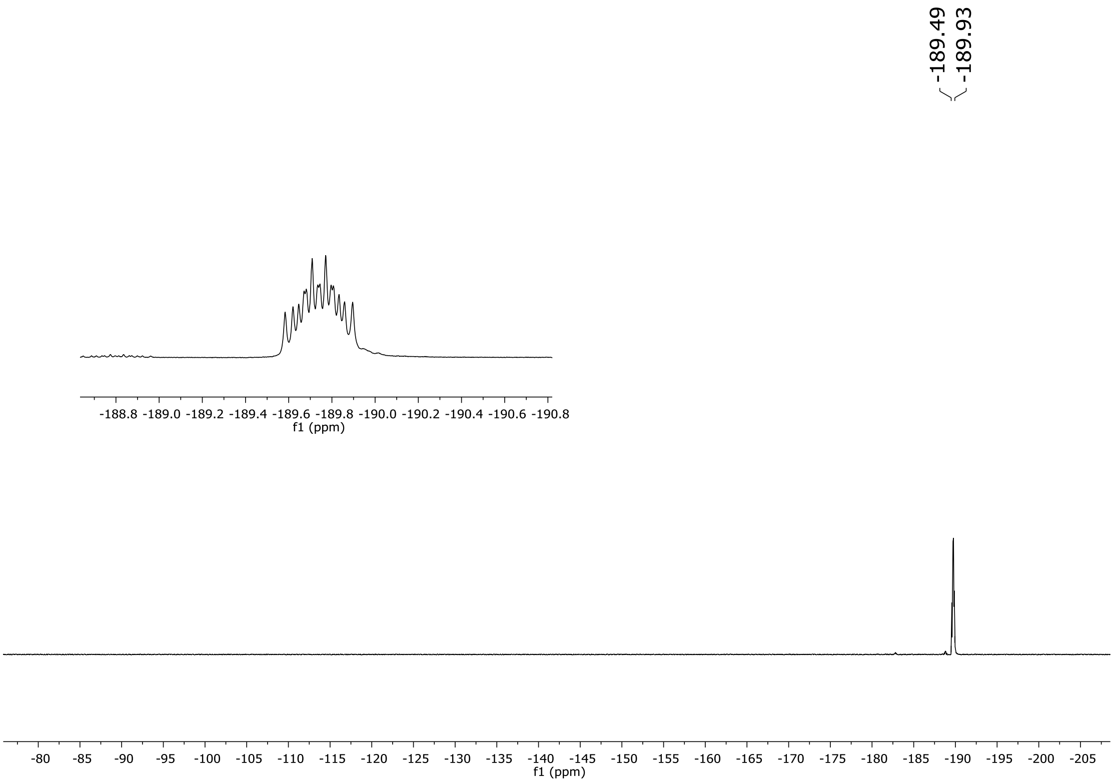

$^{13}C\ \{^1H\}$ NMR (100 MHz, $CDCl_3$):

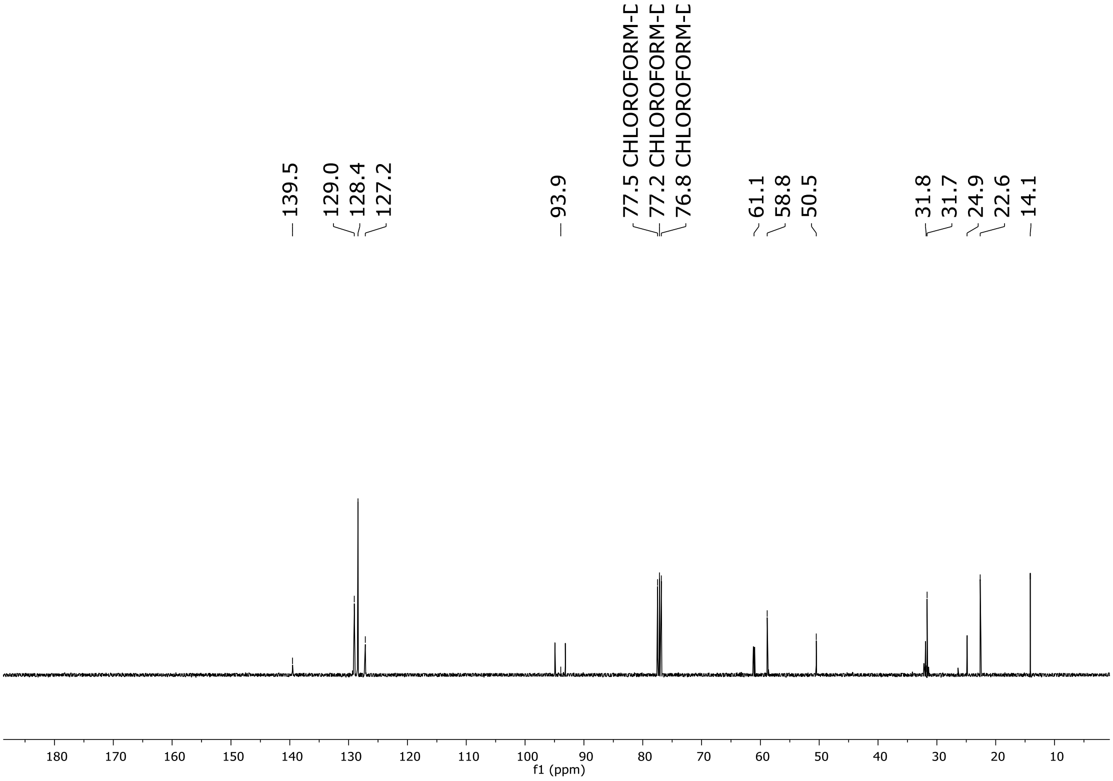

(3R,4R)-N,N-dibenzyl-3-chloro-4-fluorononan-1-amine, 46b

^{1}H NMR (400 MHz, $CDCl_3$):

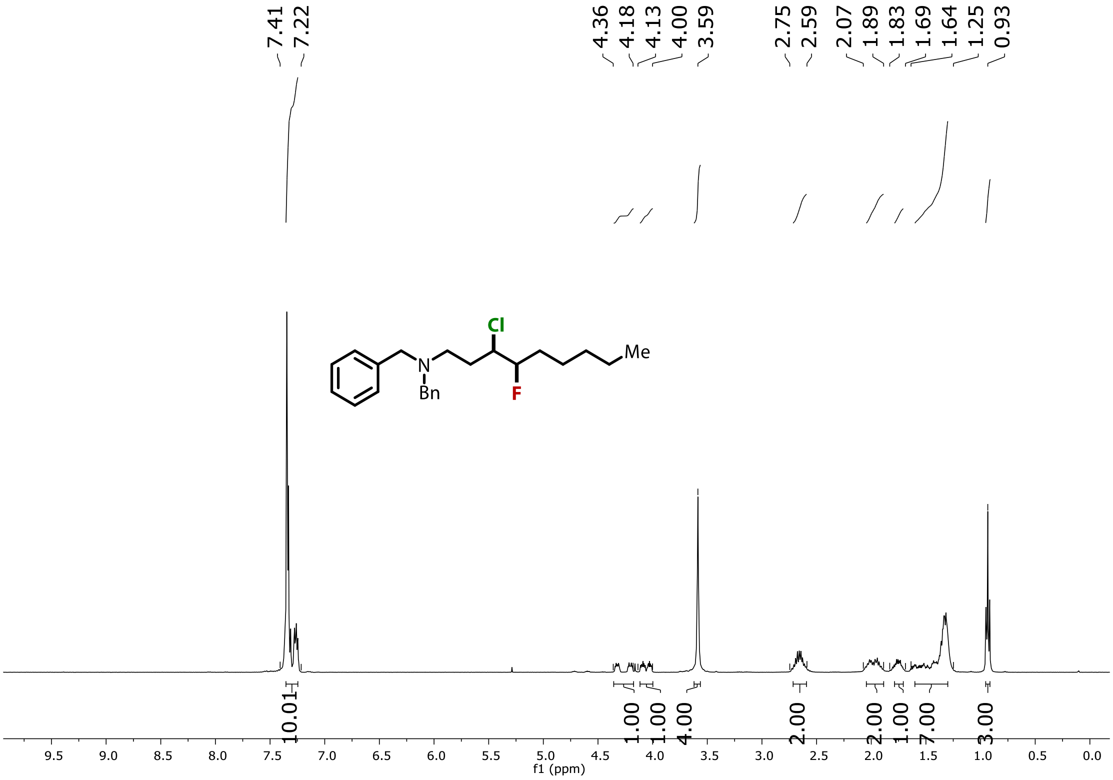

^{19}F NMR (376 MHz, $CDCl_3$):

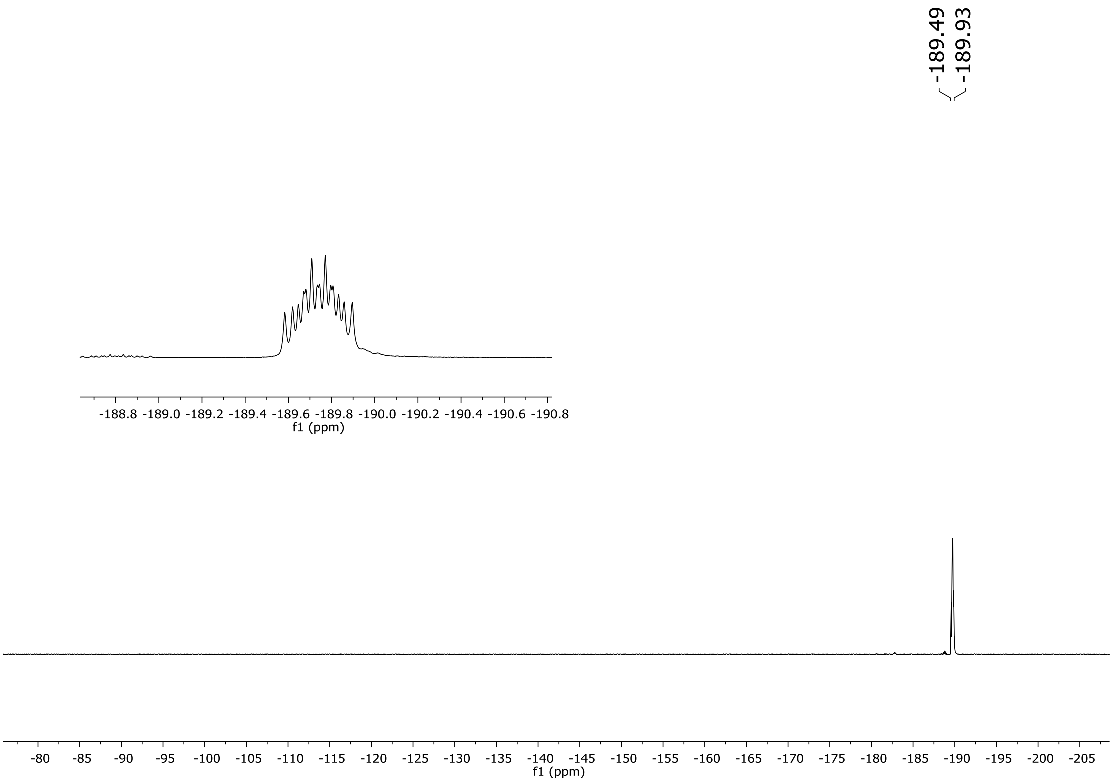

$^{13}C\ \{^{1}H\}$ NMR (100 MHz, $CDCl_3$):

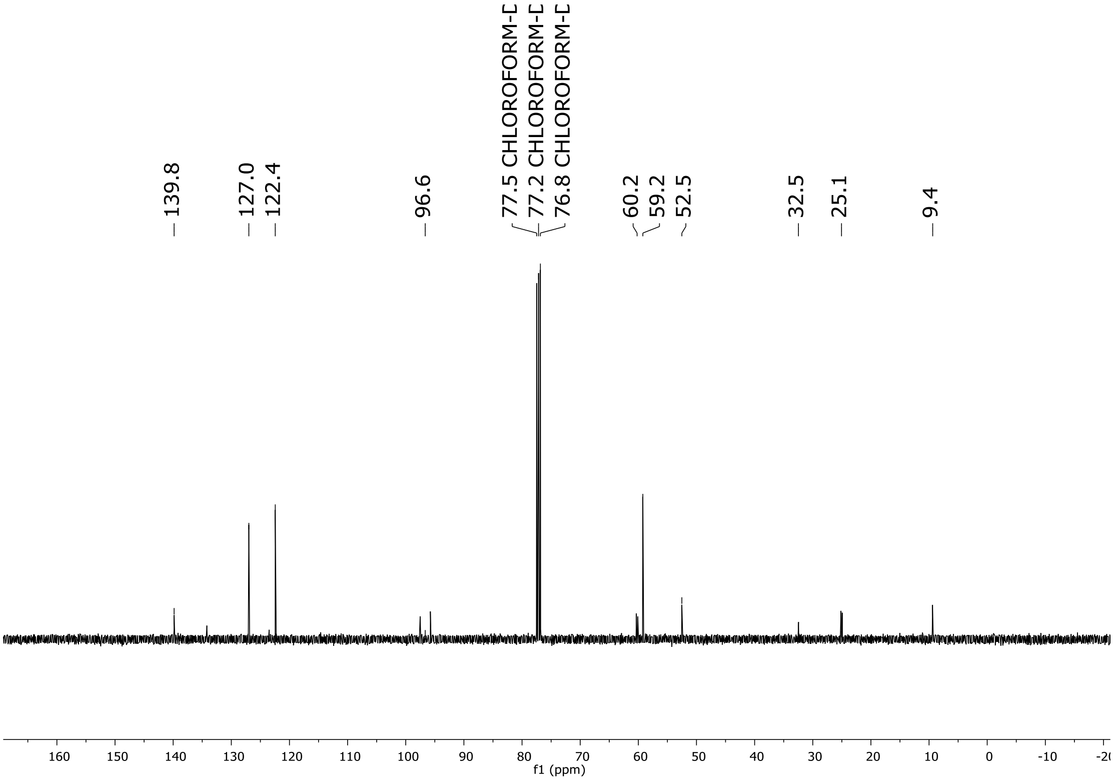

(E)-N-((3S,4S)-3-chloro-4-fluorohexyl)-N-(4-fluorobenzyl)hex-3-en-1-amine, 47b

^{1}H NMR (500 MHz, $CDCl_3$):

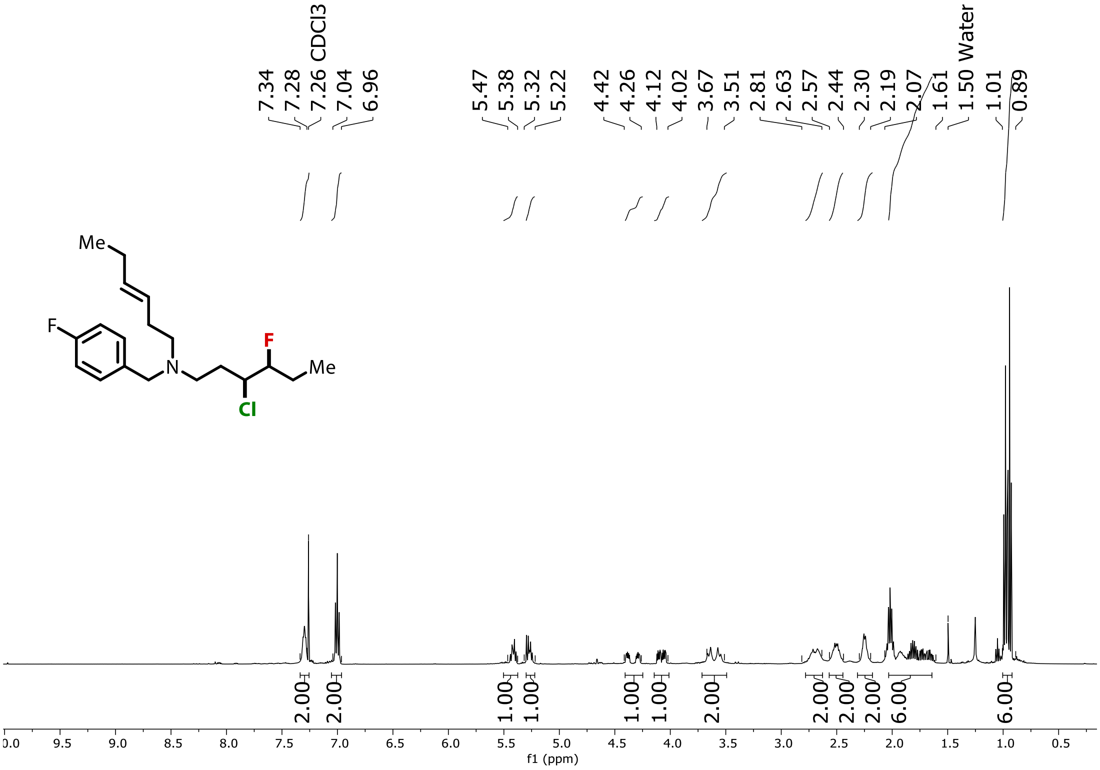

^{19}F NMR (376 MHz, $CDCl_3$):

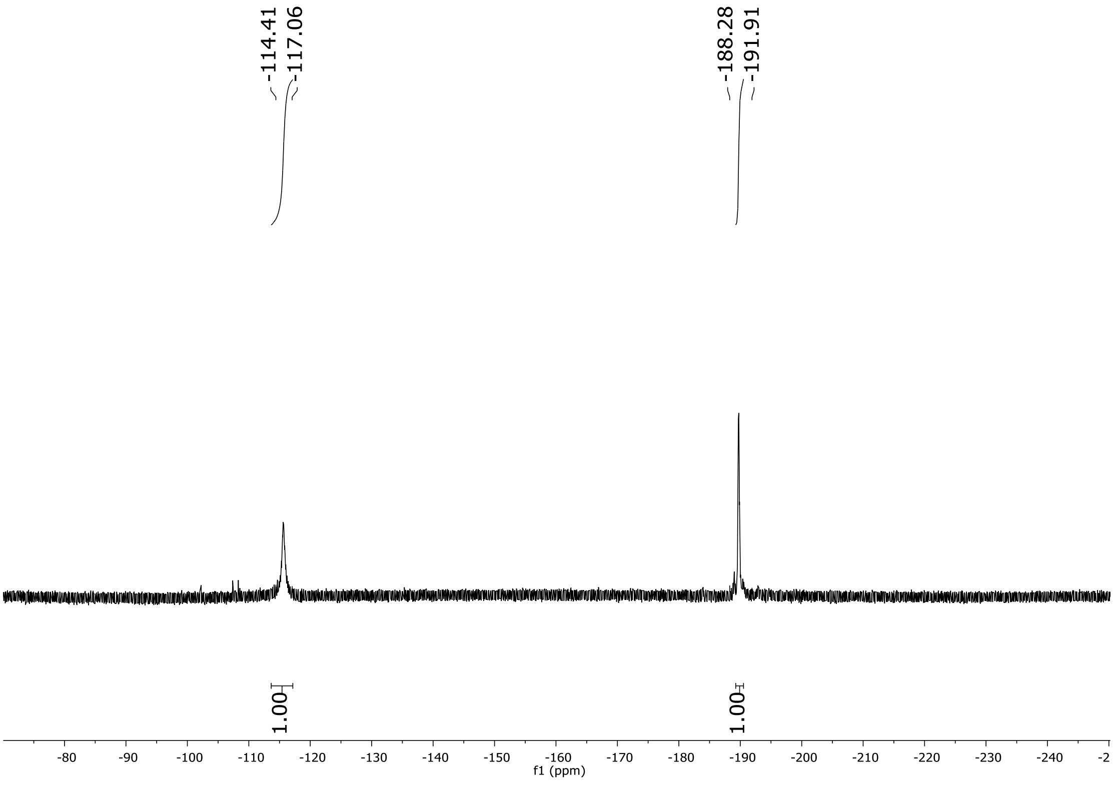

$^{13}C\ \{^{1}H\}$ NMR (125 MHz, CDCl$_3$):

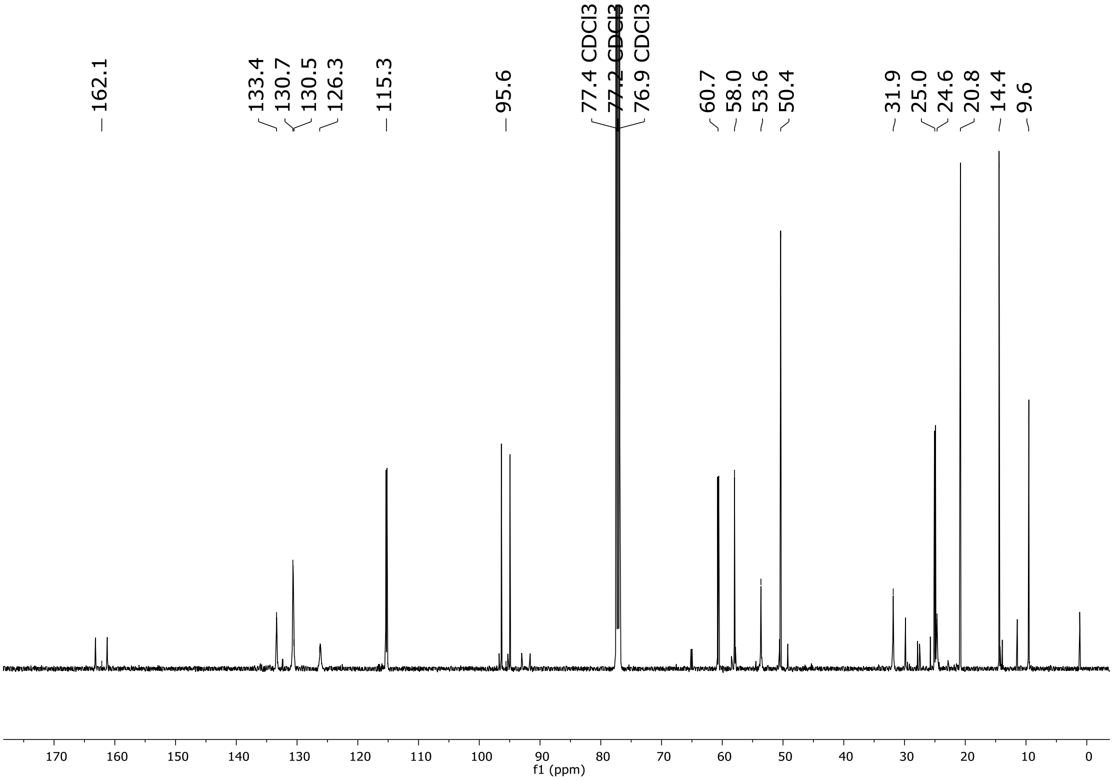

2-((3S,4S)-3-Chloro-4-fluorohexyl)isoindoline, 40b

^{1}H NMR (500 MHz, CDCl$_3$):

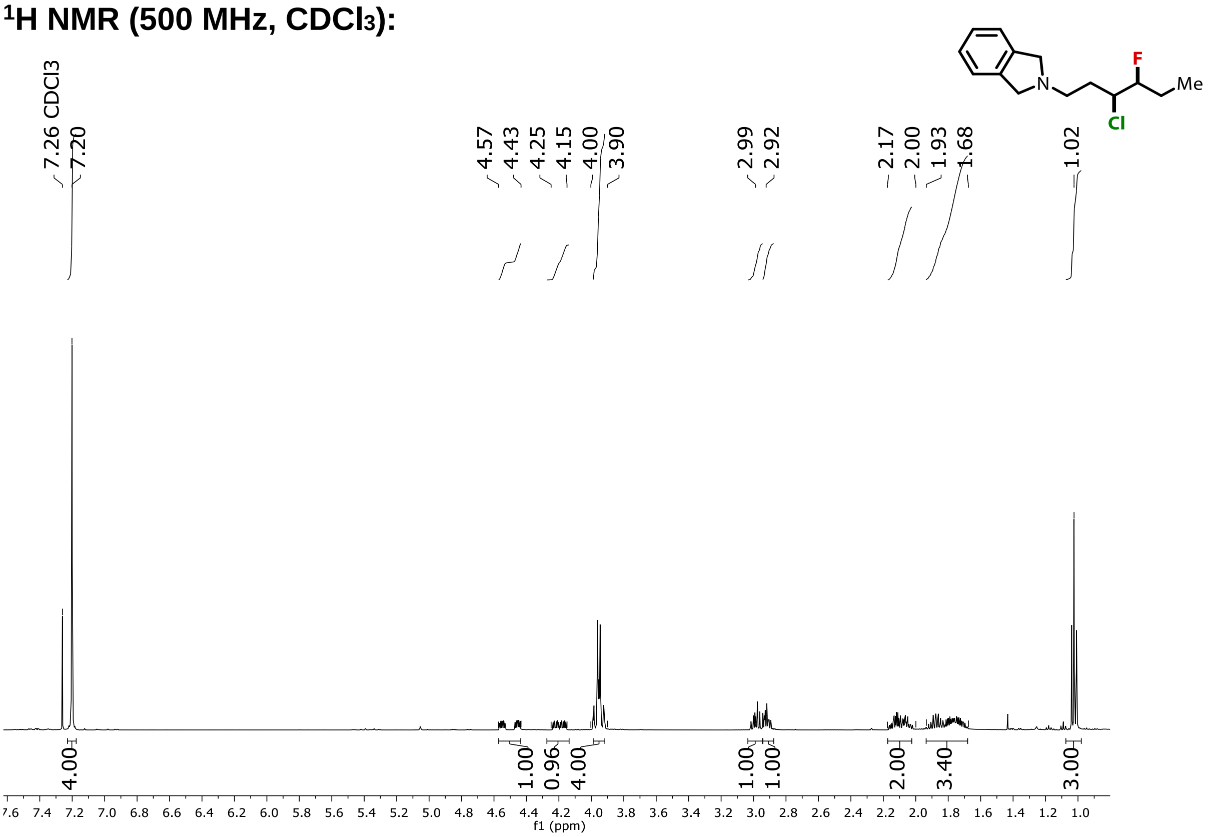

^{19}F NMR (376 MHz, CDCl$_3$):

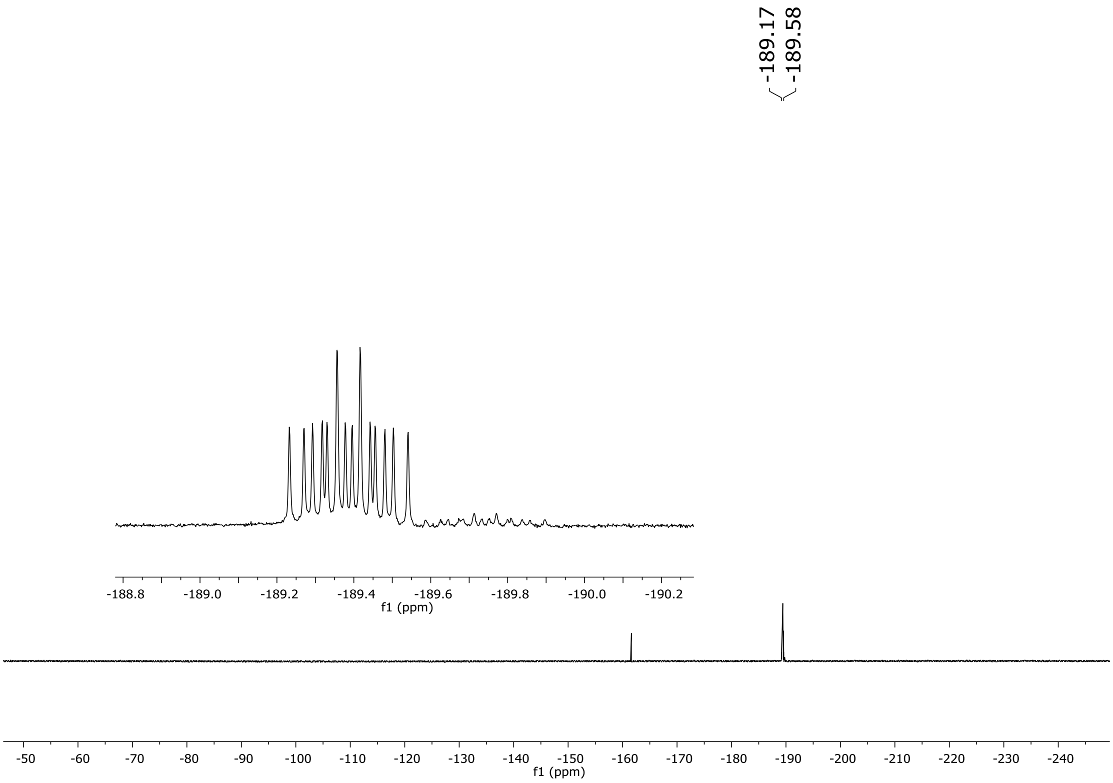

^{13}C {^{1}H} NMR (125 MHz, CDCl$_3$):

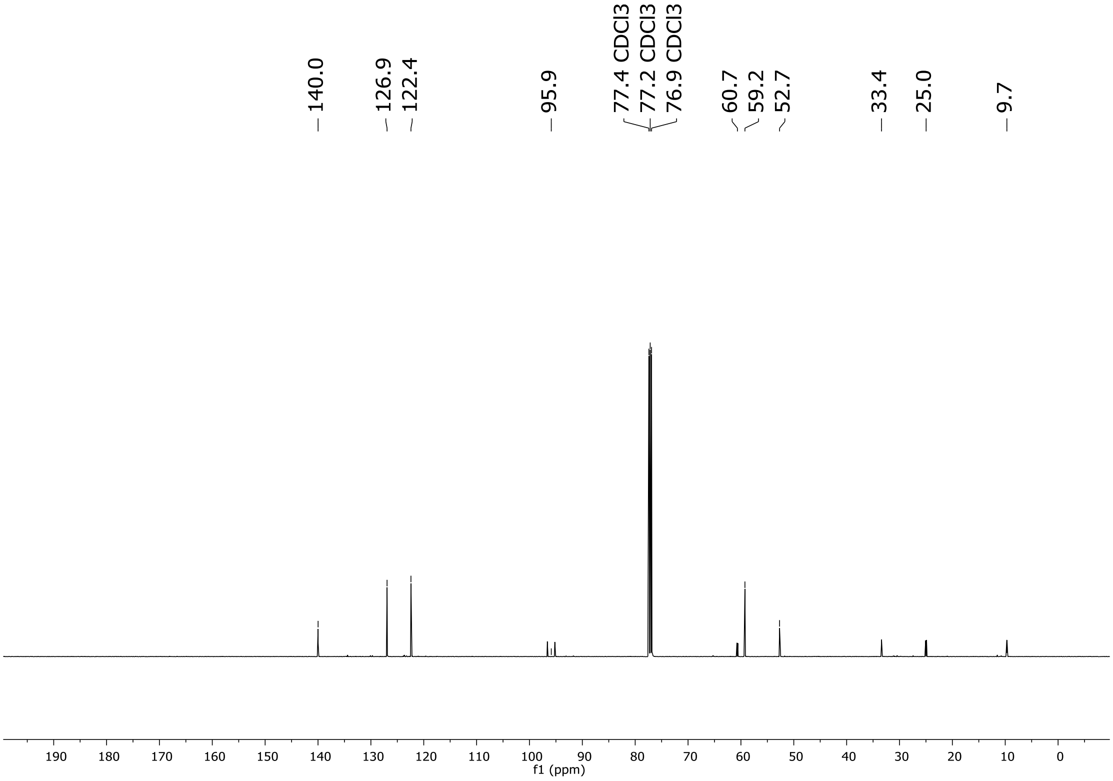

Supplement: Supplementary file 1 — Supplementary discussion, Figs. 1–55, Tables 1–18 and Schemes 1–5. [file 41557_2024_1561_MOESM1_ESM.pdf]
