# Supplementary material for: Diastereodivergent nucleophile–nucleophile alkene chlorofluorination
Source: Nat Chem. 2024 Jul 1;16(10):1647–55. doi: 10.1038/s41557-024-01561-6 (PMC11446824; doi:10.1038/s41557-024-01561-6)
Supplement: Supplementary file 3 — Eight files of xyz coordinates: 1,2_chloride_shift.docx Cartesian coordinates of model alkene forming anti-chlorofluoride through 1,2-chloride shift via chloronium cation. alkene_activation.docx Cartesian coordinates of I(III)–alkene complexes and complexation transition states. direct_chloronium_formation_transition_states.docx Cartesian coordinates of direct Cl+ delivery to alkene transition states. iodane_ligand_exchange.docx Cartesian coordinates of iodanes IF2, IFCl and ICl2 and ligand exchange transition states between them with different sites and extents of HF coordination. iodine(III)iranium_vs_iodine(III)-π_complex.docx Cartesian coordinates of iodine(III)iranium and iodine(III)–π complex with model homoallylic amine showing latter is favoured thermodynamically. isolated_fluoride_chloride_hf_clusters.docx Cartesian coordinates of fluoride and chloride with 0–6 HF coordinated to anions. ligand_coupling_transition_states.docx Cartesian coordinates of ligand coupling of fluoride or chloride from C–I(III) intermediates. syn-1,2-halo-λ3-iodanation.docx Cartesian coordinates of alkene syn-difunctionalisation to form C–I(III) and C–X (X = F or Cl). [file 41557_2024_1561_MOESM3_ESM.zip › Calculations archive/1,2 chloride shift.docx]

### 1,2-chloride shift

#### 48-INT1-0HF

H -0.77379200 1.29259200 -1.82932000

C -1.58758700 1.87363500 -1.38004500

H -1.15892000 2.74145600 -0.87109400

H -2.23914700 2.22538100 -2.18364400

C -2.36667900 0.99293200 -0.42432100

H -2.90140600 0.19515600 -0.94011100

C -1.56049800 0.38715000 0.71932500

H -2.21784200 -0.16959900 1.38575300

Cl -3.68563800 1.98614500 0.35962200

I -0.45683200 -1.34505700 -0.13033400

H 3.59628100 1.33354000 -2.01659400

C 3.22193500 0.80328700 -1.14421200

C 2.24782500 -0.56218900 1.07965300

C 4.01874100 0.71035200 0.00150900

C 1.95235000 0.22581200 -1.18681600

C 1.46513900 -0.44772200 -0.06917800

C 3.51412600 0.01768600 1.10948100

H 1.35275700 0.30674800 -2.08932100

H 4.12003900 -0.06841200 2.00858800

H 1.87466800 -1.09431800 1.95043300

C 5.39909300 1.31110800 0.03939600

H 5.59867000 1.77919800 1.00822600

H 5.52598100 2.06397600 -0.74343400

H 6.15939400 0.53609700 -0.11371100

C -0.62325100 1.31228400 1.45387000

H -0.14351300 0.80151400 2.29246800

H -1.22123500 2.13789800 1.85933900

H 0.15036200 1.73683400 0.80852900

F -2.77128100 -2.00449900 -0.10265900

SCF Done: E(RM062X) = -1285.80752881 A.U. after 22 cycles

Zero-point correction= 0.235299 (Hartree/Particle)

Thermal correction to Energy= 0.245993

Thermal correction to Enthalpy= 0.246712

Thermal correction to Gibbs Free Energy= 0.203258

#### 48-INT1-1HF

C 2.50745800 1.14134700 -0.03486500

H 2.94935600 0.29299400 -0.55443200

C 0.98987500 1.13122500 -0.18673000

H 0.70510100 1.17547200 -1.23901800

Cl 3.07303500 2.60637300 -0.96890800

H -2.10304700 -0.25588400 2.18527700

C -2.52522300 -0.16132600 1.18942000

C -3.60798300 0.09962200 -1.37072100

C -1.74753900 -0.39977300 0.05755600

C -3.85620300 0.21088800 1.02084100

C -4.41535400 0.34940400 -0.25674900

C -2.27324200 -0.28003200 -1.22429300

H -4.47026100 0.40058500 1.89816500

H -1.66104500 -0.47713600 -2.09946300

H -4.02463200 0.19809700 -2.36981000

I 0.27749100 -0.94945100 0.29011100

C -5.85758100 0.75164900 -0.41372800

H -6.51891300 0.00915900 0.04609400

H -6.12983300 0.84640500 -1.46807100

H -6.05001700 1.71035100 0.07972500

C 3.01468900 1.22548800 1.39010000

H 2.61794600 0.38186600 1.96441000

H 4.10510600 1.15805700 1.39987200

H 2.71347400 2.16060800 1.87019000

F 2.87841100 -1.70132500 0.46757800

F 3.02373700 -3.11129500 -1.33566300

H 2.97571800 -2.45789300 -0.48112100

C 0.22242400 2.10763400 0.67135500

H 0.61383300 3.11159500 0.46340500

H -0.84051500 2.10949800 0.42070100

H 0.34183200 1.90559700 1.73929400

SCF Done: E(RM062X) = -1386.29426108 A.U. after 21 cycles

Zero-point correction= 0.245471 (Hartree/Particle)

Thermal correction to Energy= 0.257915

Thermal correction to Enthalpy= 0.258635

Thermal correction to Gibbs Free Energy= 0.209004

#### 48-INT1

C 2.23468 1.50150 -0.10688

H 2.66170 0.80566 -0.82739

C 0.71232 1.41195 -0.11398

H 0.31452 1.64357 -1.10295

Cl 2.62120 3.15464 -0.77962

H -2.07982 -0.61840 2.11569

C -2.58663 -0.41115 1.17829

C -3.88637 0.13083 -1.23442

C -1.88626 -0.39324 -0.02595

C -3.95403 -0.14999 1.15580

C -4.62155 0.12675 -0.04501

C -2.51613 -0.13150 -1.23776

H -4.51170 -0.16062 2.08922

H -1.95950 -0.13149 -2.17000

H -4.38843 0.33685 -2.17613

I 0.18882 -0.77254 -0.01812

C -6.09886 0.41568 -0.04258

H -6.65118 -0.38258 0.46394

H -6.48540 0.51169 -1.06047

H -6.30865 1.34867 0.49242

C 2.88691 1.34428 1.25147

H 2.58432 0.39042 1.69671

H 3.97403 1.33669 1.14253

H 2.60407 2.15600 1.92722

F 2.96233 -1.35990 -0.20208

F 2.70876 -2.82582 -2.03683

H 2.84379 -2.21572 -1.24829

F 3.07603 -2.32993 1.94959

H 3.05710 -1.93277 1.02525

C -0.01346 2.13115 0.99633

H 0.28361 3.18704 0.95759

H -1.09605 2.08802 0.85749

H 0.24181 1.73616 1.98325

SCF Done: E(RM062X) = -1486.77625804 A.U. after 21 cycles

Zero-point correction= 0.257749 (Hartree/Particle)

Thermal correction to Energy= 0.271701

Thermal correction to Enthalpy= 0.272420

Thermal correction to Gibbs Free Energy= 0.219182

#### 48-TS1-0HF

C 2.81830800 0.66500700 0.41919700

H 3.20412900 -0.35139700 0.25546200

C 1.90082300 1.08949600 -0.64023000

H 1.97291000 0.54151500 -1.57667800

Cl 3.93071600 1.85277300 -0.52523500

H -1.66499500 -0.13039100 2.13110000

C -2.21159600 0.00911100 1.20245200

C -3.62885100 0.37814300 -1.16362500

C -1.69046900 -0.46761700 -0.00191600

C -3.44172800 0.66421000 1.21220700

C -4.17069300 0.85833100 0.03222900

C -2.39771400 -0.28049400 -1.18822700

H -3.84174500 1.03105700 2.15515900

H -1.99879900 -0.64568300 -2.13042800

H -4.17424700 0.51864700 -2.09407800

I 0.19965500 -1.42286200 -0.02745600

C -5.51135200 1.54524200 0.06314000

H -6.27668000 0.88187600 0.48281300

H -5.83199400 1.83280100 -0.94207100

H -5.48103200 2.44445400 0.68682100

C 2.62273500 1.07940500 1.84564000

H 1.82974600 0.44442700 2.25836400

H 3.53871300 0.88922100 2.40962700

H 2.33844700 2.12748700 1.95737100

F 2.87568100 -2.14349400 -0.11101900

C 0.91273400 2.18021000 -0.54467600

H 1.01491800 2.78345700 0.35728100

H 0.95403000 2.80557200 -1.44125600

H -0.08161900 1.70667900 -0.54329600

SCF Done: E(RM062X) = -1285.77231087 A.U. after 20 cycles

Zero-point correction= 0.231384 (Hartree/Particle)

Thermal correction to Energy= 0.243015

Thermal correction to Enthalpy= 0.243735

Thermal correction to Gibbs Free Energy= 0.195991

#### 48-TS1-1HF

C 2.63442600 0.99064700 0.24001900

H 3.07640200 0.21976400 -0.39148100

C 1.34775900 1.48232100 -0.26352000

H 1.13131900 1.26024300 -1.30705800

Cl 3.20829700 2.61594700 -0.49707900

H -2.14119000 -0.44942100 2.14803100

C -2.55881900 -0.22591800 1.17068200

C -3.64499000 0.35677900 -1.32615000

C -1.83974600 -0.50839200 0.01190800

C -3.82728900 0.34955000 1.06814300

C -4.38989100 0.64805800 -0.17583100

C -2.37797600 -0.21670200 -1.24216700

H -4.38514600 0.56741700 1.97584500

H -1.81894300 -0.43699200 -2.14701800

H -4.06264800 0.58046400 -2.30550200

I 0.09635400 -1.34338700 0.15395600

C -5.76574500 1.25127400 -0.28900700

H -6.46876200 0.53364200 -0.72715700

H -5.75655200 2.13497300 -0.93563300

H -6.15090800 1.54586200 0.69105400

C 2.92978500 0.88228100 1.70436200

H 2.40132000 -0.00177600 2.07777600

H 4.00113300 0.72435500 1.84677300

H 2.61223600 1.76131100 2.26840900

F 2.88799700 -2.14815400 0.32599800

F 3.46871100 -1.31146900 -1.71334500

H 3.19830300 -1.77216000 -0.71389900

C 0.36830900 2.28338300 0.49174200

H 0.17282900 3.21965100 -0.04340600

H -0.58576900 1.73677200 0.47696400

H 0.66322500 2.48125000 1.52202300

SCF Done: E(RM062X) = -1386.27047303 A.U. after 20 cycles

Zero-point correction= 0.242514 (Hartree/Particle)

Thermal correction to Energy= 0.255295

Thermal correction to Enthalpy= 0.256014

Thermal correction to Gibbs Free Energy= 0.205949

#### 48-TS1

C 2.32878 1.54033 0.04179

H 2.84644 0.80707 -0.57262

C 0.99756 1.89865 -0.45794

H 0.80125 1.66885 -1.50316

Cl 2.73615 3.21839 -0.68189

H -2.01699 -0.38887 2.12528

C -2.57185 -0.27885 1.19822

C -4.00966 0.00625 -1.17074

C -1.95601 -0.51688 -0.03034

C -3.91325 0.09996 1.22685

C -4.65348 0.24544 0.04738

C -2.66744 -0.37337 -1.21902

H -4.39202 0.28290 2.18634

H -2.18857 -0.55842 -2.17598

H -4.56262 0.11535 -2.10073

I 0.08170 -1.06960 -0.08613

C -6.11103 0.62242 0.09761

H -6.72321 -0.24258 0.37874

H -6.46183 0.97831 -0.87511

H -6.29135 1.40635 0.83977

C 2.62624 1.45088 1.50730

H 2.17222 0.52403 1.87499

H 3.70633 1.38465 1.65639

H 2.22942 2.29615 2.07251

F 3.07468 -1.26033 -0.06386

F 3.18484 -2.70414 -1.92299

H 3.15039 -2.10370 -1.11316

F 3.15874 -2.07094 2.14744

H 3.13529 -1.74317 1.19291

C -0.05813 2.57948 0.31026

H -0.34939 3.50026 -0.20818

H -0.94934 1.93515 0.28109

H 0.21480 2.78682 1.34453

SCF Done: E(RM062X) = -1486.75165902 A.U. after 21 cycles

Zero-point correction= 0.254444 (Hartree/Particle)

Thermal correction to Energy= 0.269013

Thermal correction to Enthalpy= 0.269732

Thermal correction to Gibbs Free Energy= 0.213976

#### 48-INT2

H -2.21114600 2.22877500 0.91415700

C -2.71555500 1.42614300 1.45371200

H -1.97750800 0.65761900 1.72030800

H -3.15712900 1.80726600 2.37684600

C -3.74195000 0.72325800 0.64313400

H -4.37572800 0.00495600 1.15843500

C -3.70255300 0.59302000 -0.80573800

H -4.31788900 -0.20263300 -1.21954800

C -2.63783800 1.15151500 -1.67529700

H -1.89482100 0.34866400 -1.77555100

H -2.15006900 2.03159900 -1.25470000

H -3.03493400 1.37240600 -2.66832300

Cl -5.00803600 1.94224200 -0.22060600

F -2.30572200 -1.32904900 0.10794200

F -2.26819100 -2.60625900 -1.88511800

H -2.27180700 -2.09752500 -1.02184800

F -2.40188200 -2.24713700 2.28718900

H -2.34844000 -1.89141000 1.35154400

C 3.34984100 -0.14279900 -1.17054400

C 4.69651600 0.21383600 -1.20157700

C 5.34489800 0.71026700 -0.06377000

C 4.60205100 0.84019000 1.11236800

C 3.25115700 0.48766400 1.16077800

C 2.63001800 -0.00268300 0.01621900

H 2.86912300 -0.52844000 -2.06461300

H 5.25351600 0.10160000 -2.12955300

H 5.08220600 1.22139700 2.01065900

H 2.69637800 0.59489600 2.08808600

C 6.80316100 1.08674400 -0.11760300

H 6.97893000 1.86956300 -0.86370900

H 7.15232100 1.45488800 0.85114900

H 7.41986200 0.22545100 -0.39727000

I 0.58353600 -0.54902100 0.07498100

SCF Done: E(RM062X) = -1486.75764715 A.U. after 18 cycles

Zero-point correction= 0.254973 (Hartree/Particle)

Thermal correction to Energy= 0.270139

Thermal correction to Enthalpy= 0.270858

Thermal correction to Gibbs Free Energy= 0.212155

#### 48-TS2

H -0.96873100 2.08711400 -0.38366000

C -0.40735600 1.97422800 0.54508300

H 0.63517700 2.26518200 0.37073700

H -0.82532600 2.63111300 1.31132400

C -0.38021100 0.56722100 1.04596400

H 0.08421300 0.42200900 2.01903700

C -0.27950300 -0.59070200 0.16307300

H 0.04140400 -1.51786000 0.63114900

C -0.50295800 -0.59543400 -1.29373500

H 0.49549000 -0.71416800 -1.73793300

H -0.95583900 0.31896200 -1.67628800

H -1.08801600 -1.47311400 -1.58102200

Cl -2.09162800 -0.21494400 1.18668700

F 1.99293500 -0.08445400 0.08528200

F 3.07888900 -1.94817300 -0.91763500

H 2.65726800 -1.15505400 -0.48578600

F 3.02222300 1.26746300 1.74875000

H 2.62103400 0.68858000 1.04260300

C 2.97657200 2.62037900 -5.15321900

C 2.74424600 3.12500600 -3.87383400

C 2.68532900 4.49878700 -3.65963500

C 2.85792000 5.37010700 -4.73770900

C 3.08947500 4.89052500 -6.02937100

C 3.14653100 3.50386000 -6.21742100

H 3.02650700 1.54905300 -5.32301600

H 2.50879900 4.89617700 -2.66460800

H 2.81123300 6.44265800 -4.56383100

H 3.32773000 3.10541400 -7.21362000

I 2.47636500 1.79082800 -2.24941400

C 3.27490200 5.82889500 -7.19390300

H 4.24739700 5.67166400 -7.67317900

H 2.50537400 5.66445300 -7.95635200

H 3.21801600 6.87276600 -6.87306200

SCF Done: E(RM062X) = -1486.75704600 A.U. after 18 cycles

Zero-point correction= 0.255181 (Hartree/Particle)

Thermal correction to Energy= 0.269659

Thermal correction to Enthalpy= 0.270378

Thermal correction to Gibbs Free Energy= 0.214483

#### 4-iodotoluene

C 0.85334 1.21001 -0.00525

C 2.24854 1.19971 -0.01077

C 2.96783 -0.00000 -0.01088

C 2.24854 -1.19972 -0.01077

C 0.85334 -1.21002 -0.00525

C 0.16436 -0.00000 -0.00169

H 0.31601 2.15322 -0.00701

H 2.78423 2.14634 -0.01681

H 2.78423 -2.14634 -0.01681

H 0.31601 -2.15322 -0.00701

C 4.47447 0.00000 0.01686

H 4.84251 0.00022 1.04983

H 4.87975 -0.88768 -0.47768

H 4.87975 0.88749 -0.47805

I -1.95594 0.00000 0.00227

SCF Done: E(RM062X) = -568.576727772 A.U. after 18 cycles

Zero-point correction= 0.118397 (Hartree/Particle)

Thermal correction to Energy= 0.123364

Thermal correction to Enthalpy= 0.124083

Thermal correction to Gibbs Free Energy= 0.093407

#### 2,3-chlorofluorobutane

C -0.88801 -0.44221 -0.35798

H -0.73974 -1.21310 -1.11965

C -1.25490 -1.04689 0.97677

H -2.10241 -1.72612 0.85046

H -0.41392 -1.61758 1.38211

H -1.53350 -0.26825 1.69268

F -1.97907 0.33619 -0.79807

C 0.31044 0.50936 -0.35970

H 0.38637 0.95164 -1.35529

C 0.26907 1.58564 0.70617

H 1.09643 2.28686 0.57015

H -0.67138 2.14098 0.61879

H 0.33404 1.15939 1.71115

Cl 1.81389 -0.49265 -0.17408

SCF Done: E(RM062X) = -717.291552064 A.U. after 11 cycles

Zero-point correction= 0.117086 (Hartree/Particle)

Thermal correction to Energy= 0.121640

Thermal correction to Enthalpy= 0.122359

Thermal correction to Gibbs Free Energy= 0.094745

#### Hydrogen fluoride

F 0.00000000 0.00000000 0.09403900

H 0.00000000 0.00000000 -0.84635200

SCF Done: E(RM062X) = -717.291552064 A.U. after 11 cycles

Zero-point correction= 0.008964 (Hartree/Particle)

Thermal correction to Energy= 0.010763

Thermal correction to Enthalpy= 0.011482

Thermal correction to Gibbs Free Energy= -0.002879
