# Supplementary material for: Diastereodivergent nucleophile–nucleophile alkene chlorofluorination
Source: Nat Chem. 2024 Jul 1;16(10):1647–55. doi: 10.1038/s41557-024-01561-6 (PMC11446824; doi:10.1038/s41557-024-01561-6)
Supplement: Supplementary file 3 — Eight files of xyz coordinates: 1,2_chloride_shift.docx Cartesian coordinates of model alkene forming anti-chlorofluoride through 1,2-chloride shift via chloronium cation. alkene_activation.docx Cartesian coordinates of I(III)–alkene complexes and complexation transition states. direct_chloronium_formation_transition_states.docx Cartesian coordinates of direct Cl+ delivery to alkene transition states. iodane_ligand_exchange.docx Cartesian coordinates of iodanes IF2, IFCl and ICl2 and ligand exchange transition states between them with different sites and extents of HF coordination. iodine(III)iranium_vs_iodine(III)-π_complex.docx Cartesian coordinates of iodine(III)iranium and iodine(III)–π complex with model homoallylic amine showing latter is favoured thermodynamically. isolated_fluoride_chloride_hf_clusters.docx Cartesian coordinates of fluoride and chloride with 0–6 HF coordinated to anions. ligand_coupling_transition_states.docx Cartesian coordinates of ligand coupling of fluoride or chloride from C–I(III) intermediates. syn-1,2-halo-λ3-iodanation.docx Cartesian coordinates of alkene syn-difunctionalisation to form C–I(III) and C–X (X = F or Cl). [file 41557_2024_1561_MOESM3_ESM.zip › Calculations archive/Syn-1,2-halo-╬╗3-iodanation.docx]

### *Syn*-1,2-halo-λ^3^-iodanation

#### 48-IFCl

H -1.93295600 -3.45238700 -1.84803300

C -1.87283900 -2.38869800 -1.57756600

H -1.74495900 -1.82510100 -2.50582400

H -2.80804100 -2.09537800 -1.09960700

C -0.68922000 -2.22012000 -0.68900700

H 0.29037000 -2.25250000 -1.16659400

C -0.70508600 -2.22394400 0.69449800

H 0.26310400 -2.25384100 1.19502300

C -1.90649300 -2.39942100 1.55620400

H -1.92968100 -3.45166300 1.87310600

H -2.83897200 -2.16651500 1.04185500

H -1.82788600 -1.79277800 2.46297300

I -0.48689500 0.32909500 0.00068100

F -3.22446900 -0.09047200 -0.00097700

Cl -0.07188000 2.89707700 0.00229200

H 1.72729300 0.04812900 2.16267500

C 2.26810800 -0.01208900 1.22295100

C 3.65722600 -0.14862200 -1.19856100

C 3.65401400 -0.16634700 1.21143400

C 1.60394300 0.06519500 0.00498600

C 2.27475000 0.00450500 -1.21383400

C 4.36465500 -0.23643400 0.00877600

H 4.18750600 -0.22874500 2.15605800

H 1.73648200 0.07728800 -2.15415900

H 4.19610500 -0.19733300 -2.14158800

C 5.86038700 -0.39925000 -0.00256300

H 6.26054500 -0.45867000 1.01265500

H 6.33615600 0.44565700 -0.51202500

H 6.14703700 -1.30887600 -0.54133500

H -3.50972300 0.45237000 -1.22112900

F -3.65643200 0.82218700 -2.14133900

F -3.68116800 0.83085600 2.13042300

H -3.52304400 0.45620300 1.21400900

SCF Done: E(RM062X) = -1486.73629613 A.U. after 21 cycles

Zero-point correction= 0.254261 (Hartree/Particle)

Thermal correction to Energy= 0.269390

Thermal correction to Enthalpy= 0.270106

Thermal correction to Gibbs Free Energy= 0.214670

#### 48-IFCl_iso_

H -1.95343600 -3.46515500 -1.70346800

C -1.94177100 -2.37725200 -1.53190200

H -1.87180600 -1.88964300 -2.50662300

H -2.86698800 -2.08900700 -1.03184200

C -0.72588400 -2.12296200 -0.72248300

H 0.23314500 -2.18202500 -1.23854900

C -0.68049900 -2.14198500 0.69445800

H 0.31113900 -2.22137200 1.14110900

C -1.84496000 -2.41892000 1.57158500

H -1.91026100 -3.51555000 1.64342700

H -2.78642700 -2.03845900 1.17119600

H -1.67746700 -2.02565600 2.57615000

I -0.48418000 0.19655300 0.02799500

F 0.21978800 2.48451800 0.06459700

Cl -3.56726700 0.26712300 0.05815500

H 1.71953700 -0.23973700 2.19081200

C 2.27108500 -0.20479100 1.25572300

C 3.69582200 -0.07127500 -1.14468600

C 3.66171000 -0.30964800 1.25310900

C 1.61829700 -0.04404800 0.03917700

C 2.30907000 0.03355800 -1.16757100

C 4.38988200 -0.24545900 0.06059600

H 4.18490400 -0.43446200 2.19726600

H 1.78362900 0.17547000 -2.10713000

H 4.24811300 -0.01194100 -2.07932300

C 5.89070200 -0.35207300 0.05757200

H 6.27877700 -0.52498600 1.06441200

H 6.34017600 0.56810100 -0.33135700

H 6.22004500 -1.17400700 -0.58712300

H 0.79941700 2.86668500 -1.19872800

F 1.15910700 3.07824100 -2.08729400

F 1.55402400 2.98916100 2.02846000

H 1.03277400 2.81125000 1.21716600

SCF Done: E(RM062X) = -1486.71378093 A.U. after 22 cycles

Zero-point correction= 0.255027 (Hartree/Particle)

Thermal correction to Energy= 0.270054

Thermal correction to Enthalpy= 0.270774

Thermal correction to Gibbs Free Energy= 0.216404

#### *Syn*-ICl-TS

C -3.19274700 -2.42001700 0.86514000

H -2.50695300 -2.50585300 1.71009600

H -4.04546100 -1.77976700 1.10186600

C -2.54494000 -1.98558700 -0.37718900

H -3.18065500 -1.56976800 -1.15676900

C -1.17632100 -2.11839200 -0.65828400

H -0.90990100 -2.00918800 -1.71057200

C -0.26600500 -2.95232100 0.19756200

H -0.31805300 -2.68998900 1.25802200

H -0.57299900 -4.00071900 0.09099400

Cl -3.96224600 0.86321600 -0.13827700

I -0.85532500 0.26260200 -0.11840100

C 1.19106400 -0.21116600 -0.08955100

C 1.81149100 -0.44249400 1.13517000

C 1.88496800 -0.27768100 -1.29591900

C 3.17125500 -0.74384500 1.14282100

H 1.25353000 -0.39166900 2.06513100

C 3.24115300 -0.58729500 -1.26341700

H 1.38410000 -0.09171200 -2.24137800

C 3.90163500 -0.82167700 -0.04929300

H 3.66997900 -0.92356000 2.09159300

H 3.79635100 -0.64364700 -2.19621600

F -0.09322800 2.33751100 0.28121300

C 5.37462200 -1.12481900 -0.02980100

H 5.66840600 -1.71223800 -0.90445600

H 5.65442600 -1.67281800 0.87391200

H 5.95192900 -0.19273600 -0.04818000

F 1.06890100 2.66935500 2.43778700

H 0.62805100 2.56624400 1.58120800

F 1.03118600 3.26985400 -1.71398700

H 0.60266100 2.94115200 -0.90976700

H 0.76891300 -2.86695700 -0.13916600

H -3.59272300 -3.42807100 0.65106200

SCF Done: E(RM062X) = -1486.70240223 A.U. after 22 cycles

Zero-point correction= 0.254922 (Hartree/Particle)

Thermal correction to Energy= 0.267902

Thermal correction to Enthalpy= 0.268621

Thermal correction to Gibbs Free Energy= 0.217974

#### *Syn*-IF-TS

C -1.74880300 1.71043900 0.62424700

C -1.16112100 0.81265000 1.56300800

H -0.16126400 1.18170200 1.82382600

C -1.98822400 0.30833500 2.72077300

H -1.09741100 2.11104400 -0.15239100

C 2.92164400 1.41133500 -1.18054100

C 1.70660800 0.73829900 -1.03705900

C 1.50591500 -0.00288400 0.11952300

C 2.46570800 -0.10721600 1.12204300

C 3.66767100 0.57240400 0.95106900

H 3.09577200 1.99852400 -2.07835700

H 0.94446300 0.79413100 -1.80942400

H 2.28673000 -0.70341300 2.01155400

H 4.43059400 0.50174100 1.72269800

I -0.33541500 -1.00332800 0.36504200

C 3.91256500 1.33888600 -0.19741500

F -2.74425000 0.34853200 -1.00561600

H -4.10123700 0.51992000 -1.18418500

H -1.92143800 0.71321700 -2.02862600

F -5.08630800 0.65006600 -1.27834300

F -1.25704900 1.02644200 -2.71384500

C -3.06960200 2.31313100 0.78984800

H -2.90452500 3.12676500 1.52296000

H -3.79561600 1.62899900 1.23487100

H -3.44178400 2.76237500 -0.13035300

H -2.86235700 -0.25065600 2.37153400

H -2.33906700 1.15189100 3.32669600

H -1.39045900 -0.34524900 3.36017100

C 5.22716900 2.05242700 -0.36439400

H 5.20330000 2.73104100 -1.22088100

H 5.47545200 2.63049900 0.53157100

H 6.03688000 1.33129200 -0.52349700

Cl 0.91001500 -3.16586100 -0.81245800

SCF Done: E(RM062X) = -1486.71305081 A.U. after 22 cycles

Zero-point correction= 0.254582 (Hartree/Particle)

Thermal correction to Energy= 0.268186

Thermal correction to Enthalpy= 0.268905

Thermal correction to Gibbs Free Energy= 0.216844

#### 48-IFCl-Cl_SN2_-TS

H -1.32914700 3.09032900 1.90029800

C -1.53794900 2.04444200 1.63528700

H -1.56330900 1.46055400 2.55823800

H -2.51342400 1.99566700 1.14803000

C -0.42952800 1.58769200 0.74072200

H 0.53615400 1.39479900 1.20717500

C -0.38620400 1.91361900 -0.62674000

H 0.53239900 1.68872300 -1.16459000

C -1.54002800 2.47271100 -1.37106900

H -1.87584800 3.39399400 -0.88200700

H -2.38574300 1.77207300 -1.33586400

H -1.27770200 2.68205100 -2.40789100

I -0.78843600 -0.72993700 0.03090800

F -3.41819400 0.21812800 -0.03837400

Cl -0.85952600 -3.45589200 -0.23303700

H 1.43458900 -0.83050200 -2.13737500

C 1.97631200 -0.93722500 -1.20232300

C 3.36425300 -1.22725800 1.20467600

C 3.36360600 -1.06646900 -1.19840300

C 1.30930300 -0.94626600 0.01725100

C 1.97808300 -1.09924700 1.22704700

C 4.07482100 -1.20699400 -0.00174500

H 3.89889000 -1.05612300 -2.14433900

H 1.43732200 -1.11334800 2.16874300

H 3.90071500 -1.34328100 2.14303600

C 5.57670200 -1.30104500 -0.00661400

H 5.94473500 -1.71515200 -0.94941100

H 5.93699400 -1.92563700 0.81584000

H 6.01739300 -0.30429500 0.11526500

H -3.84569900 -0.17106200 1.19821900

F -4.10849600 -0.44183000 2.12781600

F 2.79936700 2.17675300 0.27947400

H 2.23083800 2.96011800 0.10009900

Cl 1.03058700 4.34998700 -0.26814600

F -4.24764800 -0.78880900 -2.01329800

H -3.92158100 -0.37378800 -1.16059500

SCF Done: E(RM062X) = -2047.58068600 A.U. after 21 cycles

Zero-point correction= 0.266187 (Hartree/Particle)

Thermal correction to Energy= 0.283779

Thermal correction to Enthalpy= 0.284498

Thermal correction to Gibbs Free Energy= 0.222638
