# Supplementary material for: Diastereodivergent nucleophile–nucleophile alkene chlorofluorination
Source: Nat Chem. 2024 Jul 1;16(10):1647–55. doi: 10.1038/s41557-024-01561-6 (PMC11446824; doi:10.1038/s41557-024-01561-6)
Supplement: Supplementary file 3 — Eight files of xyz coordinates: 1,2_chloride_shift.docx Cartesian coordinates of model alkene forming anti-chlorofluoride through 1,2-chloride shift via chloronium cation. alkene_activation.docx Cartesian coordinates of I(III)–alkene complexes and complexation transition states. direct_chloronium_formation_transition_states.docx Cartesian coordinates of direct Cl+ delivery to alkene transition states. iodane_ligand_exchange.docx Cartesian coordinates of iodanes IF2, IFCl and ICl2 and ligand exchange transition states between them with different sites and extents of HF coordination. iodine(III)iranium_vs_iodine(III)-π_complex.docx Cartesian coordinates of iodine(III)iranium and iodine(III)–π complex with model homoallylic amine showing latter is favoured thermodynamically. isolated_fluoride_chloride_hf_clusters.docx Cartesian coordinates of fluoride and chloride with 0–6 HF coordinated to anions. ligand_coupling_transition_states.docx Cartesian coordinates of ligand coupling of fluoride or chloride from C–I(III) intermediates. syn-1,2-halo-λ3-iodanation.docx Cartesian coordinates of alkene syn-difunctionalisation to form C–I(III) and C–X (X = F or Cl). [file 41557_2024_1561_MOESM3_ESM.zip › Calculations archive/Isolated Fluoride and Chloride HF clusters.docx]

### Isolated Fluoride Anionic Clusters

**Fluoride anion (F_clust-0HF)**

F 0.00000000 0.00000000 0.00000000

**F_clust-1HF**

H -0.00004302 0.00000000 -0.00012350

F -0.00015369 0.00000000 -1.13003489

F 0.00019671 0.00000000 1.13015839

**F_clust-2HF**

H 0.40999555 -0.73287786 -0.83219241

F 0.54747766 -0.99999499 -1.79047548

F 0.23957895 -0.39699566 0.42559608

H -0.36816665 0.66164475 0.90656320

F -0.82888551 1.46822375 1.29050861

**F_clust-3HF**

H 0.00011764 0.00000000 1.42822557

F -0.00018826 0.00000000 2.39884669

F 0.00001383 0.00000000 0.01127736

H 1.21972702 0.00000000 -0.71080357

F 2.05288629 0.00000000 -1.20872914

H -1.21967055 0.00000000 -0.71048999

F -2.05288600 0.00000000 -1.20832692

**F_clust-4HF**

H 0.27014350 0.29913897 1.46309600

F 0.43778231 0.47828971 2.38424942

F 0.00113426 0.00589489 0.00754772

H 1.09358613 0.58876405 -0.85673946

F 1.78412965 0.94510691 -1.40895509

H -1.32458715 0.60769435 -0.39138516

F -2.15722320 0.99543199 -0.64683867

H -0.03752972 -1.48834276 -0.20905365

F -0.06743578 -2.43197812 -0.34192111

**F_clust-5HF**

H 0.00132928 -0.00018594 1.58031170

F 0.00306641 0.00277324 2.52379246

F -0.00027204 -0.00189634 -0.00761844

H -1.62792086 0.00221296 -0.00981456

F -2.56769510 -0.00010008 -0.00976857

H -0.00099946 -1.38175036 -0.78398866

F 0.00152822 -2.20355455 -1.24776795

H 1.62695310 -0.00095501 -0.00933741

F 2.56669161 -0.00052797 -0.00722711

H -0.00020495 1.38017361 -0.78384339

F -0.00247618 2.20381044 -1.24473807

**F_clust-6HF**

H -0.00041781 -0.00036939 1.67015143

F -0.00114758 -0.00055645 2.60637836

F 0.00001682 0.00040471 0.00001958

H -0.00041834 1.67032783 -0.00017690

F -0.00032717 2.60655035 -0.00008783

H -1.67041921 0.00024183 0.00037130

F -2.60665173 -0.00009990 0.00124361

H 0.00002940 -1.66997318 0.00061115

F 0.00001360 -2.60620737 0.00042683

H 1.67098381 -0.00024859 -0.00001474

F 2.60722492 -0.00041265 -0.00096241

H 0.00021119 0.00032178 -1.67086025

F 0.00090209 0.00002100 -2.60710012

### Isolated Chloride Anionic Clusters

**Chloride anion (Cl_clust-0HF)**

Cl 0.00000000 0.00000000 0.00000000

**Cl_clust-1HF**

H 0.00000000 0.00000000 -0.32000751

Cl 0.00000000 0.00000000 1.60033240

F 0.00000000 0.00000000 -1.28032489

**Cl_clust-2HF**

H -1.52803575 0.00000000 0.01131392

F -2.27630128 0.00000000 0.59938795

Cl -0.00063317 0.00000000 -1.22155341

H 1.52831673 0.00000000 0.01139539

F 2.27666036 0.00000000 0.59944219

**Cl_clust-3HF**

H -0.01112078 0.00000000 2.09136133

F 0.00656541 0.00000000 3.03501582

Cl -0.00207942 0.00000000 0.06595344

H -1.67597770 0.00000000 -1.02888412

F -2.45657969 0.00000000 -1.56291092

H 1.67611130 0.00000000 -1.03850242

F 2.46308088 0.00000000 -1.56203313

**Cl_clust-4HF**

H 1.42501642 -0.31813304 1.38763393

F 2.08477841 -0.48351468 2.03857102

Cl -0.00430792 0.04676700 -0.02520012

H -0.32335965 2.06461628 -0.21468512

F -0.46818379 2.99101056 -0.29408732

H 0.61000311 -0.83237172 -1.77165856

F 0.89532301 -1.23819208 -2.57138555

H -1.71555541 -0.89890109 0.58601914

F -2.50371417 -1.33128123 0.86479259

**Cl_clust-5HF**

H -1.84659991 -0.09462289 -0.72966774

F -2.72162303 -0.15264082 -1.06553751

Cl 0.07952112 0.04747723 0.02091883

H 0.47426873 2.10219037 0.23999777

F 0.64786862 3.01925453 0.32107672

H 1.20829586 0.07021887 -1.76736818

F 1.73412545 0.06505647 -2.54201795

H 0.73064222 -1.94803242 0.22803519

F 1.00930626 -2.83734865 0.31913638

H -0.51394958 -0.10615676 2.04349524

F -0.80185574 -0.16539595 2.93193124

**Cl_clust-6HF**

H -0.00000003 0.00000000 2.14658637

F 0.00000002 0.00000000 3.08057850

Cl 0.00000006 -0.00000001 0.00000000

H -0.00000003 2.14658624 0.00000000

F 0.00000002 3.08057838 0.00000000

H -2.14658647 0.00000000 0.00000000

F -3.08057860 0.00000000 0.00000000

H -0.00000003 -2.14658621 0.00000000

F 0.00000002 -3.08057835 0.00000000

H 2.14658644 0.00000000 0.00000000

F 3.08057857 0.00000000 0.00000000

H -0.00000003 0.00000000 -2.14658640

F 0.00000002 0.00000000 -3.08057854
