# Supplementary material for: Diastereodivergent nucleophile–nucleophile alkene chlorofluorination
Source: Nat Chem. 2024 Jul 1;16(10):1647–55. doi: 10.1038/s41557-024-01561-6 (PMC11446824; doi:10.1038/s41557-024-01561-6)
Supplement: Supplementary file 3 — Eight files of xyz coordinates: 1,2_chloride_shift.docx Cartesian coordinates of model alkene forming anti-chlorofluoride through 1,2-chloride shift via chloronium cation. alkene_activation.docx Cartesian coordinates of I(III)–alkene complexes and complexation transition states. direct_chloronium_formation_transition_states.docx Cartesian coordinates of direct Cl+ delivery to alkene transition states. iodane_ligand_exchange.docx Cartesian coordinates of iodanes IF2, IFCl and ICl2 and ligand exchange transition states between them with different sites and extents of HF coordination. iodine(III)iranium_vs_iodine(III)-π_complex.docx Cartesian coordinates of iodine(III)iranium and iodine(III)–π complex with model homoallylic amine showing latter is favoured thermodynamically. isolated_fluoride_chloride_hf_clusters.docx Cartesian coordinates of fluoride and chloride with 0–6 HF coordinated to anions. ligand_coupling_transition_states.docx Cartesian coordinates of ligand coupling of fluoride or chloride from C–I(III) intermediates. syn-1,2-halo-λ3-iodanation.docx Cartesian coordinates of alkene syn-difunctionalisation to form C–I(III) and C–X (X = F or Cl). [file 41557_2024_1561_MOESM3_ESM.zip › Calculations archive/Direct chloronium formation transition state.docx]

### Direct chloronium formation transition state

#### IFCl-2HF

C -0.58776700 -0.29264100 0.02930000

C -3.35136200 -0.44990000 0.07957000

C -1.26743600 -0.44273000 -1.17672100

C -1.23430900 -0.22010400 1.25701500

C -2.62622400 -0.29762700 1.26586200

C -2.65496800 -0.52094200 -1.13548300

H -0.73544400 -0.49739700 -2.12104800

H -0.68086800 -0.10485600 2.18335600

H -3.15198200 -0.23971400 2.21491500

H -3.20605700 -0.63823700 -2.06517800

C -4.85277500 -0.54127400 0.09381600

H -5.18569500 -1.48570200 -0.34998100

H -5.29423900 0.27016700 -0.49477400

H -5.24387400 -0.48109100 1.11239800

I 1.50669300 -0.15262700 -0.00902900

F 1.12377600 1.96686200 -0.06905300

Cl 1.70368000 -2.65054000 0.03911400

H 0.58348900 2.49519200 -1.42899500

F 0.27566700 2.74623400 -2.30409800

H 0.49079800 2.66253500 1.16600300

F 0.12517700 3.05558200 1.96325100

SCF Done: E(RM062X) = -1329.53309122 A.U. after 20 cycles

Zero-point correction= 0.144548 (Hartree/Particle)

Thermal correction to Energy= 0.155171

Thermal correction to Enthalpy= 0.155890

Thermal correction to Gibbs Free Energy= 0.111437

#### ICl2

C 0.64030700 0.04543700 -0.00980600

C 3.41037900 0.06286100 -0.00760800

C 1.30373700 0.22058700 1.19978500

C 1.30581800 -0.11171800 -1.21807300

C 2.70015500 -0.10151900 -1.20130900

C 2.69458600 0.23098700 1.18550300

H 0.75771700 0.34341600 2.12967100

H 0.76409200 -0.24528200 -2.14899700

H 3.23969700 -0.22795600 -2.13606400

H 3.23168800 0.36515300 2.12117300

C 4.91478000 0.04546800 0.01129800

H 5.27947000 -0.83459600 0.55303800

H 5.30911800 0.93023100 0.52149000

H 5.32407300 0.01651400 -1.00159600

I -1.46823700 -0.03107200 0.00246800

Cl -1.53148200 2.55839700 -0.08077900

Cl -1.28662600 -2.62035300 0.08499800

SCF Done: E(RM062X) = -1488.95483171 A.U. after 19 cycles

Zero-point correction= 0.120266 (Hartree/Particle)

Thermal correction to Energy= 0.127390

Thermal correction to Enthalpy= 0.128110

Thermal correction to Gibbs Free Energy= 0.092314

#### ICl2-2HF

C -1.20036200 -0.10822700 -0.45552800

C 0.76728900 -0.77592500 1.37007800

C -0.71535500 -1.41066500 -0.45067500

C -0.74731200 0.87505800 0.41878000

C 0.24352200 0.52361700 1.32964200

C 0.27353200 -1.73225200 0.47534100

H -1.09140800 -2.15565500 -1.14464500

H -1.14978500 1.88269500 0.39571000

H 0.61456600 1.27506900 2.02169500

H 0.66708900 -2.74483000 0.49625200

I -2.70370300 0.39400800 -1.84238300

C 1.85882300 -1.12355500 2.34405200

H 2.82274000 -0.75118800 1.97751000

H 1.94344700 -2.20532500 2.47582400

H 1.67691500 -0.66275200 3.31951200

F -0.60924200 3.78933000 -1.96354600

F 1.79712000 0.31861700 -1.89545800

H -0.53812400 3.03009900 -2.53437100

H 1.01492800 0.54586300 -2.38722700

Cl -0.67401600 1.16416600 -3.51434300

Cl -4.51105200 -0.29341700 -0.24165400

SCF Done: E(RM062X) = -1689.88612965 A.U. after 22 cycles

Zero-point correction= 0.142073 (Hartree/Particle)

Thermal correction to Energy= 0.153668

Thermal correction to Enthalpy= 0.154388

Thermal correction to Gibbs Free Energy= 0.107620

#### Cl^+^-TS-IFCl-2HF

H -3.81902900 -1.63709600 -3.05654300

C -4.53395800 -1.33789100 -2.29476000

C -6.38351000 -0.57583000 -0.29925100

C -4.20591100 -0.38053400 -1.34227500

C -5.80547300 -1.91439000 -2.24289200

C -6.72031000 -1.53726200 -1.25546200

C -5.11789900 -0.00436600 -0.35683900

H -6.08243900 -2.67005400 -2.97189300

H -7.70094700 -2.00270800 -1.22574700

H -7.09146200 -0.28875900 0.47303800

C -2.92644700 0.39312100 -1.18267200

H -2.01430300 -0.20168100 -1.27021100

H -2.87080000 1.25219400 -1.86104600

C -4.51325800 1.05382400 0.52436500

H -4.66473200 0.91220900 1.59600700

H -4.82584600 2.06344900 0.23758400

N -3.04288800 0.91828800 0.22277800

C -2.26543400 2.15834600 0.50026700

H -2.69305000 2.95697600 -0.11206900

H -2.42356900 2.39174200 1.55739500

C -0.78109600 1.94365000 0.19865700

H -0.61540800 1.86037500 -0.87885200

H -0.45350000 1.00348000 0.66725700

C 0.02352400 3.05892000 0.79534300

H 0.05056600 3.08524200 1.88343700

C 0.63084000 4.08888500 0.11280000

H 1.11754100 4.85351800 0.71787300

C 0.59319400 4.33975300 -1.35836700

H 1.59509100 4.63614100 -1.68818700

H 0.31332400 3.43969600 -1.91252600

C -0.39306400 5.47883700 -1.66534200

H -1.41337000 5.20352100 -1.37998700

H -0.12099700 6.39104300 -1.12476000

H -0.38268800 5.70076500 -2.73648100

H -2.68397800 0.19533400 0.88648800

F -2.22594000 -0.38256100 2.39000900

H -2.53163000 -1.59886300 2.99810800

F -2.76636800 -2.46312600 3.42671400

H -1.20058700 0.38365700 2.89400000

F -0.47778500 0.99614600 3.21746800

I 3.82725800 0.14927100 0.11992200

F 4.63657100 -2.52523900 -0.59635800

Cl 2.21508800 2.20006200 0.39694500

C 2.03619000 -0.95044100 0.07658500

C 1.43261600 -1.20981900 -1.14834600

C 1.45083900 -1.31351300 1.28684500

C 0.19242700 -1.84927100 -1.14878500

H 1.91185300 -0.92903000 -2.08084800

C 0.21296000 -1.94919400 1.25820000

H 1.94047300 -1.10259700 2.23237400

C -0.43749600 -2.21577900 0.04605000

H -0.28870000 -2.06661600 -2.09961500

H -0.25754400 -2.23611200 2.19485100

C -1.80046100 -2.85385500 0.04194400

H -2.55826300 -2.14352900 0.39936300

H -2.08553400 -3.17841800 -0.96297700

H -1.83203800 -3.71917100 0.71132800

F 3.07980900 -4.19907700 0.01825400

H 3.73449500 -3.48241600 -0.24645300

F 4.64001000 -1.99324700 -2.88678400

H 4.63981500 -2.24793800 -1.90438600

SCF Done: E(RM062X) = -2230.58171265 A.U. after 23 cycles

Zero-point correction= 0.487163 (Hartree/Particle)

Thermal correction to Energy= 0.510930

Thermal correction to Enthalpy= 0.511646

Thermal correction to Gibbs Free Energy= 0.435658

#### Cl^+^-TS-ICl2-2HF

H -3.81828700 -1.80756200 -3.03246300

C -4.54525500 -1.53323300 -2.27266700

C -6.42485400 -0.83584200 -0.28136400

C -4.27291800 -0.52930000 -1.35122500

C -5.77569800 -2.19006700 -2.19135900

C -6.70509900 -1.84537900 -1.20569500

C -5.20023300 -0.18450600 -0.36844600

H -6.00786300 -2.98374700 -2.89518500

H -7.65204400 -2.37414000 -1.15189000

H -7.14354300 -0.57353500 0.48982100

C -3.04347000 0.32744400 -1.22153900

H -2.09675400 -0.21100200 -1.30419200

H -3.04822500 1.17305100 -1.91917000

C -4.65784900 0.93601500 0.47583000

H -4.79942900 0.82174500 1.55196900

H -5.02972900 1.91518400 0.15536400

N -3.18293800 0.87524500 0.17319600

C -2.46990500 2.15797400 0.42789500

H -2.92635300 2.91987800 -0.20972100

H -2.65322600 2.40920100 1.47674100

C -0.97273200 2.00673600 0.15304300

H -0.78585400 1.90163200 -0.91911500

H -0.60873900 1.09682600 0.65341900

C -0.23202500 3.17513700 0.73116100

H -0.22592300 3.23130300 1.81840600

C 0.34039900 4.21221500 0.03163900

H 0.78208900 5.01337400 0.62391200

C 0.32249600 4.42154500 -1.44634700

H 1.31874000 4.74743800 -1.76556900

H 0.08805200 3.49675900 -1.98079000

C -0.69998900 5.51326700 -1.80331100

H -1.71452000 5.20582200 -1.53021600

H -0.47423500 6.44893600 -1.28178000

H -0.67669600 5.70793700 -2.87954500

H -2.78731600 0.18618100 0.85097400

F -2.33506800 -0.30439200 2.39258400

H -2.57181000 -1.51404600 3.04261500

F -2.75687500 -2.37417600 3.50325200

H -1.35106000 0.51784800 2.88533700

F -0.65810200 1.16814400 3.20172600

I 3.70254200 0.39227100 0.22020900

Cl 2.01356400 2.39117300 0.40390800

C 1.95530200 -0.77916300 0.17379600

C 1.36028800 -1.04737300 -1.05352500

C 1.38665800 -1.17381800 1.38172200

C 0.14648900 -1.73491000 -1.05842600

H 1.82818500 -0.74099800 -1.98370600

C 0.17444800 -1.85732000 1.34748800

H 1.86834200 -0.95128600 2.32860500

C -0.46679100 -2.13741600 0.13349700

H -0.32634300 -1.96308000 -2.01092000

H -0.28302200 -2.17171800 2.28175300

C -1.80109200 -2.83306400 0.12297800

H -2.05553200 -3.19103200 -0.87875400

H -1.80663000 -3.68397000 0.81108400

H -2.59445700 -2.14832600 0.45146800

Cl 4.92744700 -2.63727800 -0.54455200

F 4.09317600 -1.44407400 -3.06847800

H 4.37182300 -1.86908700 -2.23994600

F 2.48349700 -4.16916000 -0.03805900

H 3.28032300 -3.64361400 -0.20109900

SCF Done: E(RM062X) = -2590.93451927 A.U. after 21 cycles

Zero-point correction= 0.486769 (Hartree/Particle)

Thermal correction to Energy= 0.524639

Thermal correction to Enthalpy= 0.525583

Thermal correction to Gibbs Free Energy= 0.409046

#### Cl^+^-TS-ICl2

H -3.85715800 -1.87175900 -3.01196600

C -4.53388500 -1.53205500 -2.23253200

C -6.28472500 -0.66507500 -0.19002800

C -4.14791300 -0.55136200 -1.32685100

C -5.81396300 -2.07861600 -2.10967900

C -6.67932700 -1.65031900 -1.09889000

C -5.01208000 -0.12236800 -0.31960500

H -6.13618500 -2.85151800 -2.80104100

H -7.66653100 -2.09440300 -1.01276800

H -6.95312500 -0.33836600 0.60151100

C -2.84702500 0.19929000 -1.24089700

H -1.95127300 -0.41511700 -1.35458600

H -2.80418600 1.04365900 -1.93847400

C -4.35076300 0.95304100 0.49686800

H -4.47612800 0.86278600 1.57756300

H -4.63879200 1.95854700 0.17187700

N -2.89543500 0.75545100 0.15648300

C -2.06542900 1.96960200 0.38892300

H -2.47726000 2.77240100 -0.22826600

H -2.18807700 2.22807200 1.44499700

C -0.59814500 1.68950200 0.05539400

H -0.45712700 1.59960800 -1.02479300

H -0.29740900 0.74011300 0.52170700

C 0.25935500 2.77288000 0.63458600

H 0.30491200 2.80443100 1.72174400

C 0.84660000 3.81979400 -0.06726700

H 1.34643300 4.58201300 0.52940900

C 0.73089600 4.09222700 -1.52777300

H 1.70763100 4.41654400 -1.90194500

H 0.43644500 3.19800100 -2.08322700

C -0.29309500 5.21937700 -1.75046600

H -1.28957600 4.91892900 -1.41191200

H -0.00485100 6.12601500 -1.20952700

H -0.34796600 5.45924100 -2.81616000

H -2.54493200 0.02715000 0.81949400

F -2.09679000 -0.62515600 2.30941700

H -3.23839600 -0.55905000 3.08794100

F -4.08973000 -0.47344000 3.60133200

H -1.07022700 0.17559600 2.76123700

F -0.34318100 0.80290500 3.04352300

I 4.08251100 -0.09329000 -0.06344900

Cl 2.34535300 2.01465600 0.19506300

C 2.26382700 -1.15571300 -0.07711000

C 1.65278300 -1.44232100 -1.29192000

C 1.66618300 -1.47454800 1.13957000

C 0.40053600 -2.06009400 -1.27783700

H 2.13757500 -1.19867100 -2.23217900

C 0.41604500 -2.08847700 1.12838700

H 2.15629700 -1.24281700 2.08010200

C -0.23834100 -2.38188200 -0.07542800

H -0.08351800 -2.29442400 -2.22330800

H -0.06342300 -2.33267000 2.07263000

C -1.60894700 -3.00557000 -0.06109800

H -2.34725400 -2.30374500 0.34823500

H -1.92696200 -3.29358700 -1.06734500

H -1.62862700 -3.89569500 0.57632800

Cl 5.32796600 -3.12922700 -0.51837900

SCF Done: E(RM062X) = -2389.97713630 A.U. after 22 cycles

Zero-point correction= 0.464570 (Hartree/Particle)

Thermal correction to Energy= 0.485468

Thermal correction to Enthalpy= 0.486185

Thermal correction to Gibbs Free Energy= 0.417746
