# Supplementary material for: Diastereodivergent nucleophile–nucleophile alkene chlorofluorination
Source: Nat Chem. 2024 Jul 1;16(10):1647–55. doi: 10.1038/s41557-024-01561-6 (PMC11446824; doi:10.1038/s41557-024-01561-6)
Supplement: Supplementary file 3 — Eight files of xyz coordinates: 1,2_chloride_shift.docx Cartesian coordinates of model alkene forming anti-chlorofluoride through 1,2-chloride shift via chloronium cation. alkene_activation.docx Cartesian coordinates of I(III)–alkene complexes and complexation transition states. direct_chloronium_formation_transition_states.docx Cartesian coordinates of direct Cl+ delivery to alkene transition states. iodane_ligand_exchange.docx Cartesian coordinates of iodanes IF2, IFCl and ICl2 and ligand exchange transition states between them with different sites and extents of HF coordination. iodine(III)iranium_vs_iodine(III)-π_complex.docx Cartesian coordinates of iodine(III)iranium and iodine(III)–π complex with model homoallylic amine showing latter is favoured thermodynamically. isolated_fluoride_chloride_hf_clusters.docx Cartesian coordinates of fluoride and chloride with 0–6 HF coordinated to anions. ligand_coupling_transition_states.docx Cartesian coordinates of ligand coupling of fluoride or chloride from C–I(III) intermediates. syn-1,2-halo-λ3-iodanation.docx Cartesian coordinates of alkene syn-difunctionalisation to form C–I(III) and C–X (X = F or Cl). [file 41557_2024_1561_MOESM3_ESM.zip › Calculations archive/Alkene activation.docx]

### Alkene activation

#### 40a-IF2-TS

C -1.83567500 4.02824500 0.30196600

C -3.03029600 3.38661000 0.65771200

C -3.31897600 2.10300400 0.20646700

C -2.39450900 1.47707100 -0.62936400

C -1.20141900 2.08931000 -1.01870000

C -0.93364400 3.36688000 -0.54474700

H -3.73883000 3.89249800 1.30769000

H -4.23539600 1.60266900 0.50312600

H -0.49456300 1.58218700 -1.66869400

H -0.00484800 3.85585800 -0.82716600

I -2.75962200 -0.46682400 -1.26019900

C -1.50529200 5.39661900 0.82621400

H -0.65302400 5.34018600 1.51315400

H -1.22047600 6.06651100 0.00852900

H -2.35168800 5.83416900 1.36096300

C -2.30828300 -0.77651700 2.14744800

C -1.13876800 -0.16431000 1.90034600

C -2.55421200 -2.22873400 2.43729000

C 0.21780700 -0.79834700 1.78788800

H 0.16362700 -1.88811300 1.86456400

H 0.85046500 -0.42959500 2.60663200

C 0.83780600 -0.39088700 0.45695500

N 2.17142400 -1.02298700 0.23858300

F -3.84737600 0.10527100 -2.72950700

H -1.65583600 -2.82485000 2.25708300

C -3.03700700 -2.42215200 3.87989200

H -3.32369800 -2.60336900 1.74995400

H -3.94566500 -1.84063600 4.07292000

H -2.27174600 -2.09685400 4.59305000

H -3.26194400 -3.47550400 4.07580200

C 3.22771800 -0.65769600 1.25312400

C 2.73356300 -0.74179000 -1.13493600

H 0.98766700 0.68916300 0.39464500

H 3.15560700 -1.34031800 2.10138600

H 0.21015600 -0.71524700 -0.37791200

H 2.01441600 -2.04298100 0.28697300

H -1.14503700 0.91983800 1.77346200

H -3.19403000 -0.13970500 2.21254200

H 3.04040600 0.36822200 1.58260700

H 7.37243300 -0.98091500 -2.10571500

C 6.55801800 -0.92147000 -1.38969500

C 4.49823200 -0.77316200 0.45754000

C 5.23824400 -0.89646200 -1.84550200

C 6.84100600 -0.87455900 -0.02167600

C 5.80978800 -0.80089500 0.91715800

C 4.21605900 -0.82134100 -0.90632700

H 5.01750700 -0.93313900 -2.90872500

H 7.87310900 -0.89734800 0.31530100

H 6.02909500 -0.76175900 1.98058500

F -1.34914900 -2.58827500 -0.32209100

H -0.14689200 -3.07480700 -0.20874100

F 0.82334400 -3.41021200 -0.12162000

H 2.33004100 -1.48081900 -1.83005600

H 2.41964800 0.26507300 -1.42217000

F -3.15304100 -4.15439800 -0.51210600

H -2.38198500 -3.54232300 -0.40777000

F 5.00759900 2.58910400 -0.22001600

H 4.02341600 2.38575000 -0.16207900

F 1.60050200 2.69269100 1.89480800

H 2.09264300 2.47637900 1.02962600

F 2.69417500 2.12313900 -0.09896000

SCF Done: E(RM062X) = -1870.22579254 A.U. after 21 cycles

Zero-point correction= 0.487822 (Hartree/Particle)

Thermal correction to Energy= 0.511615

Thermal correction to Enthalpy= 0.512334

Thermal correction to Gibbs Free Energy= 0.436452

#### 40a-IFCl

I 1.58277900 1.19913700 -1.15876900

C 1.43186700 0.59157000 1.43292500

C 0.41195200 -0.13547900 0.84993200

C 1.26449700 1.84354000 2.23231900

C -1.04393300 0.18689800 0.94474600

H -1.21937500 1.20073300 1.31226600

H -1.42582400 -0.51874400 1.69812700

C -1.76463200 -0.05581200 -0.37715400

N -3.24256400 0.07168100 -0.22477400

H 0.42277800 2.43484700 1.86214100

C 1.04064700 1.46788200 3.70721100

H 2.16914300 2.45237300 2.14040000

H 1.87378000 0.87338200 4.09519100

H 0.11926800 0.88917900 3.82799500

H 0.95866400 2.37754600 4.30890800

C -3.87178200 -0.79882700 0.83709600

C -3.97862100 -0.18725500 -1.51915000

H -1.56780100 -1.05798000 -0.76006500

H -3.82355700 -0.28003400 1.79576100

H -1.47623400 0.68394400 -1.12841600

H -3.41713600 1.06050000 0.02520700

H 0.65345500 -1.11811500 0.44288000

H 2.41609800 0.12123100 1.45424600

H -3.30868100 -1.73510500 0.88799500

H -8.56145900 -1.41421100 -1.64723700

C -7.62811300 -1.29814000 -1.10427700

C -5.26151000 -1.00744900 0.30451300

C -6.50390000 -0.79597700 -1.76342200

C -7.56569900 -1.64879600 0.24755400

C -6.37742300 -1.50510600 0.96690200

C -5.32350800 -0.65711200 -1.04243800

H -6.55181600 -0.52297400 -2.81389900

H -8.45047800 -2.03591700 0.74418700

H -6.32662500 -1.78056300 2.01665000

F -0.53030100 2.90741000 -0.34160600

H -1.79919700 2.89003100 -0.15585200

F -2.82222800 2.79483300 0.00251800

H -3.98588500 0.73427200 -2.10413600

H -3.43925800 -0.97261700 -2.05564600

F 0.79352300 4.81914100 0.22929000

H 0.21330800 4.05194700 -0.00657900

F -4.55407700 -4.31230800 -0.44124900

H -3.75142800 -3.76127300 -0.68990300

F -0.83807700 -3.02356700 0.43259300

H -1.64457800 -3.07451100 -0.20011400

F -2.66197200 -3.01733600 -1.02479900

Cl 2.39191500 2.04594900 -3.46678900

C 3.27298500 -2.69206500 -2.05961800

C 4.61876600 -2.58836000 -1.68119100

C 5.07466700 -1.38322400 -1.13585800

C 4.21762800 -0.29683400 -0.96808400

C 2.89134700 -0.44269000 -1.35637100

C 2.39717900 -1.62223800 -1.90645000

H 2.90304400 -3.62219200 -2.48352300

H 6.11521300 -1.28656700 -0.83785900

H 4.58162400 0.63575900 -0.54750600

H 1.35681000 -1.71165300 -2.20538300

C 5.54733000 -3.75714600 -1.86704700

H 5.15650700 -4.64681500 -1.36217400

H 5.64763900 -4.00233300 -2.93012300

H 6.54188600 -3.54109500 -1.46899400

SCF Done: E(RM062X) = -2230.60235925 A.U. after 22 cycles

Zero-point correction= 0.487790 (Hartree/Particle)

Thermal correction to Energy= 0.512311

Thermal correction to Enthalpy= 0.513030

Thermal correction to Gibbs Free Energy= 0.434907

#### 40a-IFCl-TS

C -1.62737200 3.89771100 0.68420700

C -2.81464600 3.24330300 1.04366000

C -3.14527200 2.00510700 0.50410600

C -2.26827100 1.44170400 -0.42072300

C -1.08716000 2.06696100 -0.82168500

C -0.77749500 3.29781500 -0.25713900

H -3.48413300 3.70229000 1.76581500

H -4.05528000 1.49403800 0.80166700

H -0.42216900 1.60647900 -1.54599100

H 0.14445500 3.79695300 -0.54396600

I -2.66484300 -0.47552500 -1.14056800

C -1.25404200 5.21863100 1.29355300

H -0.29838500 5.13488400 1.82268100

H -1.12928900 5.97923900 0.51518300

H -2.01419600 5.56229900 1.99883600

C -2.05087200 -0.75252500 2.44375600

C -0.90457400 -0.11414900 2.16775300

C -2.26379400 -2.21884400 2.68980300

C 0.45370400 -0.72894700 1.97762900

H 0.42382300 -1.81654900 2.09436100

H 1.13935600 -0.32514600 2.73452200

C 0.97373000 -0.36602200 0.59149200

N 2.29408300 -0.99972200 0.30459800

H -1.36266300 -2.79448500 2.46048500

C -2.69938100 -2.47766300 4.13686500

H -3.04660800 -2.58075200 2.01057700

H -3.60902100 -1.91694100 4.38061100

H -1.91683800 -2.16987300 4.83906300

H -2.90471400 -3.54100400 4.29770100

C 3.41894500 -0.58075100 1.21937900

C 2.75295100 -0.77912700 -1.11718300

H 1.11102300 0.71148300 0.48026100

H 3.41107600 -1.21614400 2.10646600

H 0.29039400 -0.72441900 -0.18347300

H 2.15213800 -2.01721000 0.40984500

H -0.93368700 0.97169200 2.05500000

H -2.94627900 -0.13608700 2.55387800

H 3.25068400 0.46089300 1.50867300

H 7.30845500 -1.08429400 -2.41164400

C 6.54848500 -0.98633400 -1.64197000

C 4.62917400 -0.73880700 0.34090800

C 5.19910300 -0.97731400 -2.00141200

C 6.93041400 -0.87549100 -0.30192400

C 5.97058600 -0.75220200 0.70517400

C 4.24844600 -0.85173200 -0.99496500

H 4.90127600 -1.06444800 -3.04268100

H 7.98439400 -0.88811800 -0.04050800

H 6.26676800 -0.66474200 1.74683700

F -1.22721100 -2.54496900 -0.21265700

H -0.03122500 -3.05257800 -0.02604100

F 0.92412400 -3.40131000 0.10712400

H 2.29948200 -1.54640100 -1.74789300

H 2.41729300 0.21524800 -1.42321700

F -3.04413900 -4.10963600 -0.28081700

H -2.27155000 -3.49482000 -0.22430300

F 1.94260500 2.67452700 1.87663500

H 2.28871900 2.48768600 0.93607400

F 2.70111700 2.16707100 -0.28120400

Cl -4.21213600 0.26611200 -2.86818800

F 4.97208200 2.58713800 -0.78041300

H 4.00797100 2.40445500 -0.55743100

SCF Done: E(RM062X) = -2230.59398681 A.U. after 24 cycles

Zero-point correction= 0.487152 (Hartree/Particle)

Thermal correction to Energy= 0.511225

Thermal correction to Enthalpy= 0.511945

Thermal correction to Gibbs Free Energy= 0.435335

#### 40a-ICl_2_

C 4.75198200 -3.16845900 0.45398600

C 5.19203600 -1.85806400 0.69096900

C 4.38592500 -0.76251800 0.40068700

C 3.12352700 -1.00370800 -0.13431000

C 2.65347800 -2.28263800 -0.40161600

C 3.48094300 -3.36268400 -0.09609200

H 6.17930800 -1.69129600 1.11466600

H 4.73143300 0.24871200 0.59330900

H 1.66621300 -2.44794400 -0.82242900

H 3.12293600 -4.37058700 -0.28690400

I 1.85781000 0.63813000 -0.54087300

C 5.64052200 -4.33523000 0.78896800

H 5.98083900 -4.27683900 1.82809200

H 5.11854000 -5.28485600 0.64795000

H 6.53214700 -4.33475000 0.15217600

C 1.36368800 0.72581900 2.08044700

C 0.40561400 -0.14549300 1.60606500

C 1.12986400 2.15889300 2.43176900

C -1.04058300 0.16742300 1.40521300

H -1.24400400 1.24254800 1.42741300

H -1.53888400 -0.28258900 2.27726500

C -1.58686100 -0.46604200 0.12973300

N -3.07654100 -0.40136000 0.07643500

H 0.35257100 2.59259600 1.79397700

C 0.71372800 2.26806700 3.90730800

H 2.05021300 2.72712000 2.26503500

H 1.47953000 1.84714000 4.56631500

H -0.22654300 1.73762000 4.08845000

H 0.57341800 3.32025000 4.17111400

C -3.80380000 -1.03350900 1.24092700

C -3.63270900 -1.00701300 -1.19302400

H -1.31467200 -1.51979300 0.05587500

H -3.89851400 -0.29669400 2.04043200

H -1.22842000 0.06643800 -0.75693600

H -3.33890300 0.59825900 0.06207600

H 0.67333000 -1.19990900 1.52334400

H 2.32354300 0.29307400 2.36499600

H -3.21659700 -1.88962000 1.58528500

H -8.11741800 -2.51770100 -1.54122500

C -7.26448800 -2.21798900 -0.93948400

C -5.10590700 -1.45256500 0.61809900

C -6.08214100 -1.82075500 -1.56759500

C -7.36329300 -2.22818900 0.45492700

C -6.28097800 -1.84396400 1.24902900

C -5.00820500 -1.43911400 -0.77163400

H -6.00497800 -1.81065100 -2.65120100

H -8.29201300 -2.53612400 0.92607900

H -6.35592200 -1.85421800 2.33281100

H -2.38314000 2.65633000 -0.50048300

F -3.29420200 2.41229100 -0.21757800

H -3.60225200 -0.24923900 -1.97862900

H -3.00058700 -1.85785800 -1.46031800

F 0.42636900 5.17255300 0.63793000

H 0.06555200 4.47224900 0.08055500

Cl -0.61800100 2.98483700 -1.03051100

Cl 2.91429000 0.88966200 -2.87056900

F -2.35103600 -3.53590500 0.09890300

F -0.67709300 -3.16150300 1.68028000

H -1.41851700 -3.37733800 1.00258300

F -4.23540900 -4.77908000 0.81773600

H -3.43946200 -4.25065300 0.51026800

SCF Done: E(RM062X) = -2590.94940000 A.U. after 21 cycles

Zero-point correction= 0.490199 (Hartree/Particle)

Thermal correction to Energy= 0.514535

Thermal correction to Enthalpy= 0.515255

Thermal correction to Gibbs Free Energy= 0.437375

#### 40a-ICl_2_-TS

C -1.20436200 3.90649500 1.12168800

C -2.45719400 3.33581500 1.40448100

C -2.91768700 2.23141600 0.70238700

C -2.10381100 1.71459900 -0.30769000

C -0.86502000 2.26217600 -0.63569700

C -0.42570300 3.36374700 0.09171200

H -3.07216900 3.75677100 2.19562100

H -3.87735900 1.78175500 0.93611600

H -0.25434000 1.84357000 -1.42992500

H 0.54237100 3.79895200 -0.13850900

I -2.71271600 -0.01279500 -1.29877400

C -0.70084900 5.06281100 1.93608000

H -0.37258600 4.70912600 2.92078700

H 0.14982900 5.54871500 1.45251700

H -1.49079000 5.80267800 2.09789500

C -2.11435100 -0.78925300 2.36978200

C -0.92043000 -0.24765900 2.09275400

C -2.46138900 -2.23917000 2.54443000

C 0.37469100 -0.96307200 1.82364500

H 0.24262900 -2.04974600 1.80126000

H 1.08788200 -0.72304700 2.62380800

C 0.93394300 -0.47110700 0.49450400

N 2.26230600 -1.07510100 0.17546600

H -1.61303200 -2.88182400 2.29282600

C -2.93198000 -2.53024800 3.97400000

H -3.26840200 -2.49688500 1.84563100

H -3.79295100 -1.90640800 4.24007900

H -2.13333300 -2.32748500 4.69591900

H -3.22893900 -3.57911900 4.07717500

C 3.36621500 -0.73231900 1.14835700

C 2.74386300 -0.72144700 -1.21261800

H 1.08432800 0.61063800 0.49751000

H 3.35847300 -1.45855200 1.96270700

H 0.26611400 -0.73961600 -0.33155700

H 2.14366300 -2.09961500 0.18721500

H -0.84862100 0.84116400 2.05115900

H -2.94198600 -0.09683400 2.54221600

H 3.17148000 0.26880300 1.54250500

H 7.32600500 -0.80666300 -2.44029600

C 6.55082800 -0.79699900 -1.67969400

C 4.59248500 -0.77676700 0.28005000

C 5.20797300 -0.78617900 -2.06261900

C 6.90700100 -0.79848100 -0.32800300

C 5.92747500 -0.78953000 0.66719300

C 4.23760800 -0.77531500 -1.06744100

H 4.93032500 -0.78442600 -3.11300100

H 7.95634500 -0.80876100 -0.04856500

H 6.20343800 -0.78922700 1.71800000

H 0.29343200 -3.43376000 -0.48377300

F 1.23289000 -3.65666500 -0.33538100

H 2.31796000 -1.43798800 -1.91797700

H 2.39406700 0.28929100 -1.43724600

F 4.97561500 2.63099500 -0.45399100

H 4.01393200 2.39830300 -0.27185200

F 1.97236600 2.43008900 2.16010200

H 2.30129400 2.32363500 1.20199100

F 2.70901800 2.10022100 -0.04209400

Cl -1.51914400 -2.90526500 -0.77705600

Cl -4.10261300 1.15959400 -2.93984600

F -2.82386300 -5.06947600 0.79036000

H -2.41142900 -4.34673100 0.30908000

SCF Done: E(RM062X) = -2590.94059766 A.U. after 21 cycles

Zero-point correction= 0.486144 (Hartree/Particle)

Thermal correction to Energy= 0.510890

Thermal correction to Enthalpy= 0.511609

Thermal correction to Gibbs Free Energy= 0.433270

#### *trans*-40a

H 4.07051000 -0.64981400 -2.23550000

C 3.87452900 -0.75079100 -1.17153700

C 3.36258800 -0.98245100 1.59660400

C 2.57345900 -0.81496200 -0.68768900

C 4.92621400 -0.80722700 -0.25376400

C 4.67296400 -0.92216800 1.11594300

C 2.32051800 -0.92911800 0.67863300

H 5.95127200 -0.75046500 -0.60824700

H 5.50328500 -0.95410000 1.81541700

H 3.16460000 -1.06101400 2.66210800

C 1.27696100 -0.79421200 -1.44922100

H 1.02652400 -1.77367100 -1.87095500

H 1.20919800 -0.03165100 -2.22780600

C 0.83777900 -0.99251800 0.92624600

H 0.48228900 -2.01918900 1.06975100

H 0.48601800 -0.36438200 1.74627700

N 0.27348200 -0.47720900 -0.37199700

C -1.10943500 -0.93460500 -0.69132800

H -1.07439600 -2.02448500 -0.78738400

H -1.36186000 -0.49926400 -1.66255800

C -2.10566500 -0.49267100 0.37619100

H -2.06951700 0.59800700 0.48541600

H -1.82444800 -0.93547000 1.33998500

C -3.49712300 -0.93033600 0.00929500

H -3.66076100 -2.00733300 -0.07392200

C -4.51554900 -0.09630300 -0.21522100

H -4.34517500 0.97964200 -0.12357100

C -5.91531500 -0.51887500 -0.55840400

H -6.19584200 -0.08648500 -1.52808900

H -5.95423300 -1.60928000 -0.66761800

C -6.92444700 -0.05995900 0.49911800

H -7.94383800 -0.34451100 0.21828900

H -6.89868600 1.02920900 0.61763500

H -6.69945700 -0.50960800 1.47237600

H 0.25156200 0.56768200 -0.29480200

F 0.28818600 2.19405600 -0.21633400

H 1.30041200 2.67658800 -1.04300900

F 2.01336000 2.98423700 -1.66378800

H 0.32579600 2.50870700 1.13528400

F 0.32928800 2.68376500 2.11520100

SCF Done: E(RM062X) = -901.064826649 A.U. after 10 cycles

Zero-point correction= 0.340801 (Hartree/Particle)

Thermal correction to Energy= 0.354450

Thermal correction to Enthalpy= 0.355169

Thermal correction to Gibbs Free Energy= 0.301814

#### *trans*-40a-IF2

C -4.90894000 2.81487800 -0.66203700

C -5.47650500 1.55394600 -0.45048300

C -4.69388900 0.40033300 -0.45662200

C -3.32866500 0.53082400 -0.68879900

C -2.72838600 1.76745700 -0.91887100

C -3.52933500 2.90383100 -0.89692200

H -6.54468900 1.46794400 -0.27038500

H -5.14398600 -0.57211300 -0.28183900

H -1.66127400 1.84932200 -1.10408400

H -3.07416400 3.87621700 -1.06789300

I -2.15229300 -1.20438100 -0.72693000

C -5.75263900 4.06019400 -0.64457300

H -5.38159500 4.76805700 0.10423200

H -5.71527300 4.56404100 -1.61657700

H -6.79598500 3.82910800 -0.41611300

C -1.76956700 -0.85181700 1.93558100

C -0.76091000 -0.12176400 1.36366100

C 0.64584500 -0.62162300 1.22082900

H 0.68416300 -1.71102600 1.32921800

H 1.18557500 -0.18181100 2.07149500

C 1.29795700 -0.16650700 -0.07850300

N 2.74816600 -0.51281200 -0.12473500

F -2.93612200 -1.61716900 -2.49836900

C 3.58659700 -0.00921900 1.02547500

C 3.40778500 -0.04817500 -1.40225600

H 1.22437200 0.91501100 -0.19954700

H 3.53393000 -0.72767300 1.84513600

H 0.85094400 -0.65793300 -0.94685500

H 2.79853600 -1.54716300 -0.13047900

H -0.92325200 0.94226300 1.17824900

H 3.18172200 0.95556500 1.34464400

H 8.10622600 0.51287700 -1.77703700

C 7.21147300 0.40610800 -1.17084500

C 4.94616000 0.12742600 0.39858400

C 5.97174000 0.24174900 -1.79266000

C 7.31320700 0.42947700 0.22322400

C 6.17717400 0.28944800 1.02316100

C 4.84457400 0.10407700 -0.99083200

H 5.89193400 0.22223600 -2.87596100

H 8.28577000 0.55592600 0.68958500

H 6.25473600 0.31053400 2.10662200

F -0.14881700 -3.27600700 -0.43106300

H 1.12219100 -3.23248700 -0.53734500

F 2.15989200 -3.15398500 -0.61419300

H 3.22117100 -0.79245800 -2.17876100

H 2.95872400 0.90979700 -1.67804800

F -1.00384600 -4.07578500 1.63719900

H -0.62400800 -3.77772000 0.76312600

F 4.61604200 3.56800400 0.82045700

H 3.76690600 3.21432800 0.41323800

F 0.79098000 2.68500300 1.34235000

H 1.60140900 2.75157500 0.71746300

F 2.61278500 2.73361300 -0.11901800

C -3.03627200 -0.25784800 2.47189800

H -3.17157400 0.75845700 2.08675000

H -3.89711200 -0.86097300 2.15952300

C -2.96555000 -0.23673700 4.00621700

H -3.89303400 0.17424200 4.41559200

H -2.83152500 -1.24643300 4.40741100

H -2.13182400 0.38384800 4.34915500

H -1.57400200 -1.90106200 2.17228600

SCF Done: E(RM062X) = -1870.23903272 A.U. after 21 cycles

Zero-point correction= 0.487947 (Hartree/Particle)

Thermal correction to Energy= 0.511904

Thermal correction to Enthalpy= 0.512623

Thermal correction to Gibbs Free Energy= 0.436876

#### *trans*-40a-IF2-TS

C -1.53080000 3.89539800 -0.27624100

C -2.75250300 3.37633000 0.17325800

C -3.12053800 2.06350300 -0.10201200

C -2.24957800 1.27954400 -0.85777700

C -1.03685600 1.77024200 -1.34715400

C -0.68633100 3.07929700 -1.04409100

H -3.42052400 4.00323500 0.75735300

H -4.06138200 1.66345400 0.26206500

H -0.37794800 1.14510200 -1.94181800

H 0.26105400 3.47344300 -1.40262500

I -2.75078800 -0.69832500 -1.24771900

C -1.11313200 5.29858300 0.06011300

H -0.23223000 5.28264400 0.71190300

H -0.83808200 5.84849700 -0.84590900

H -1.91156600 5.84124800 0.57162900

C -2.25926100 -0.90008100 2.14946500

C -1.13092800 -0.20926000 1.93651500

C 0.22271800 -0.83557900 1.75973700

H 0.14244200 -1.92697700 1.81536600

H 0.86935000 -0.49961200 2.58108000

C 0.85016200 -0.41600700 0.43686300

N 2.18845100 -1.04856200 0.24038200

F -3.96064600 -0.23674100 -2.65936400

C 3.20949000 -0.73273000 1.30677300

C 2.80650800 -0.72269200 -1.09862300

H 0.99931100 0.66486100 0.38436400

H 3.09507400 -1.44134100 2.12856000

H 0.23737200 -0.73552300 -0.41206500

H 2.02460500 -2.06885700 0.24190200

H -1.16181000 0.88349400 1.93095900

H 3.02584500 0.28499900 1.66266600

H 7.47665900 -1.02237700 -1.89577700

C 6.63551200 -0.97070300 -1.21072800

C 4.50719600 -0.84431800 0.55731300

C 5.33566400 -0.90261900 -1.71730300

C 6.86457400 -0.97688800 0.16813600

C 5.79814600 -0.91443800 1.06746800

C 4.27815200 -0.83830200 -0.81727600

H 5.15733000 -0.89850900 -2.78904500

H 7.88178400 -1.03256900 0.54439400

H 5.97508300 -0.91661700 2.13947900

F -1.30865600 -2.72871000 -0.20087100

H -0.07794100 -3.15029400 -0.29467000

F 0.91235000 -3.41758900 -0.37348300

H 2.41873900 -1.42625200 -1.83784900

H 2.52136700 0.30038700 -1.35585400

F -3.09666300 -4.06568200 0.66956700

H -2.32754600 -3.54821900 0.32435400

F 5.13426900 2.51814200 0.25217100

H 4.14894400 2.32850500 0.16543700

F 1.50767300 2.61090700 1.91078000

H 2.09386900 2.41415900 1.10062800

F 2.82095600 2.08854500 0.04124800

H -2.21068700 -1.99086300 2.15804600

C -3.59955300 -0.29583400 2.45597400

H -4.31664800 -0.57107300 1.66880300

H -3.52061000 0.79740200 2.46053500

C -4.13837600 -0.79008100 3.80269000

H -3.46802800 -0.50188400 4.61942000

H -5.12754300 -0.36734600 4.00619200

H -4.22879000 -1.88199500 3.80786100

SCF Done: E(RM062X) = -1870.22789734 A.U. after 21 cycles

Zero-point correction= 0.487750 (Hartree/Particle)

Thermal correction to Energy= 0.511577

Thermal correction to Enthalpy= 0.512296

Thermal correction to Gibbs Free Energy= 0.436582

#### *trans*-40a-IFCl

I -2.08494400 -1.16319900 -0.41078900

C -1.64350600 -0.54367400 2.14857300

C -0.61649300 0.07226100 1.46304300

C 0.76489800 -0.49069300 1.35177500

H 0.77658700 -1.56078700 1.58457700

H 1.33075600 0.02921200 2.13897800

C 1.39650500 -0.19860900 -0.00408900

N 2.84524700 -0.54760500 -0.03093500

C 3.70283400 0.10899500 1.02407900

C 3.47841300 -0.25806400 -1.37188000

H 1.32100800 0.86041900 -0.25601600

H 3.66785500 -0.49049500 1.93496600

H 0.93287100 -0.79286500 -0.79641700

H 2.89837900 -1.57312500 0.10136900

H -0.74512500 1.11172600 1.15318300

H 3.29949700 1.10802200 1.21547700

H 8.16785800 0.24000600 -1.90893700

C 7.28495500 0.21839900 -1.27687300

C 5.05019200 0.15874000 0.35889000

C 6.03408500 -0.02939000 -1.84666300

C 7.41262600 0.43390500 0.09843800

C 6.29237500 0.40480600 0.93190100

C 4.92262300 -0.05427300 -1.01219600

H 5.93367400 -0.19814500 -2.91520400

H 8.39355600 0.62327200 0.52426800

H 6.39039300 0.57494100 2.00046300

F -0.10524900 -3.24675200 -0.05979600

H 1.17079700 -3.25576800 -0.13286300

F 2.21112300 -3.21544400 -0.18283300

H 3.27631000 -1.09828600 -2.03893100

H 3.02173200 0.65462000 -1.76507500

F -1.03051500 -3.74032300 2.07292700

H -0.62065100 -3.57993100 1.17559300

F 4.71661100 3.61860900 0.25637800

H 3.83430700 3.23456400 -0.03356000

F 0.94417900 2.76122700 1.19287000

H 1.70040200 2.78584700 0.49754800

F 2.63585000 2.71168100 -0.41297300

Cl -3.12023500 -1.99753500 -2.63224100

C -3.38954000 2.92211000 -1.05973100

C -4.75730200 2.90657200 -0.75262400

C -5.34404200 1.70103700 -0.35224600

C -4.59298100 0.53173800 -0.24857700

C -3.24004600 0.59190300 -0.56000100

C -2.61887900 1.76736600 -0.97344400

H -2.91803800 3.84975700 -1.37417700

H -6.40338000 1.67117000 -0.11202800

H -5.05795100 -0.39719400 0.06730100

H -1.56067600 1.79101700 -1.21673200

C -5.57377800 4.16422000 -0.87251300

H -5.08385300 4.99776200 -0.35936500

H -5.68467100 4.44764400 -1.92539300

H -6.57225100 4.03093400 -0.44855100

C -2.85550500 0.17493900 2.64612900

H -2.96860800 1.13694800 2.13613100

H -3.75275100 -0.42597100 2.45756900

C -2.70294000 0.39561200 4.16061700

H -1.82836100 1.01622000 4.37806400

H -3.59142700 0.90234600 4.54818100

H -2.59213000 -0.55646800 4.68873600

H -1.48179100 -1.56488200 2.50248600

SCF Done: E(RM062X) = -2230.60338221 A.U. after 22 cycles

Zero-point correction= 0.487177 (Hartree/Particle)

Thermal correction to Energy= 0.511519

Thermal correction to Enthalpy= 0.512238

Thermal correction to Gibbs Free Energy= 0.435851

#### *trans*-40a-IFCl-TS

C -1.15826400 3.82109900 -0.08143600

C -2.38133300 3.35542700 0.42232700

C -2.83961600 2.07632500 0.12758800

C -2.05520900 1.27613600 -0.70002500

C -0.84689100 1.70858700 -1.24539400

C -0.40631400 2.98600100 -0.92110100

H -2.97969200 3.99860900 1.06184300

H -3.78208600 1.71560200 0.52754800

H -0.26184100 1.06838200 -1.89826900

H 0.53951000 3.33932700 -1.32306700

I -2.68783900 -0.67520500 -1.09473900

C -0.64243500 5.18418900 0.28089400

H 0.17743000 5.09219700 1.00295900

H -0.24597200 5.69907300 -0.59951100

H -1.42545700 5.80031100 0.72953900

C -2.04848700 -0.81374100 2.39347100

C -0.90913500 -0.15086000 2.16164100

C 0.41822100 -0.81626300 1.92753100

H 0.31026300 -1.90450500 1.99966800

H 1.11916500 -0.49121900 2.70782400

C 0.98114000 -0.43149100 0.56546000

N 2.29331900 -1.09281300 0.29902100

C 3.39245100 -0.75890600 1.27892400

C 2.81631600 -0.81693000 -1.09043400

H 1.14916000 0.64480800 0.48552300

H 3.33122800 -1.44343800 2.12645500

H 0.31293900 -0.75037300 -0.24094600

H 2.11672100 -2.10949100 0.34713400

H -0.90848700 0.94224300 2.16360000

H 3.24301800 0.26986300 1.61840500

H 7.41266700 -1.17299000 -2.21760800

C 6.62428800 -1.09603600 -1.47459200

C 4.63179500 -0.90397500 0.44086400

C 5.29122400 -1.03369500 -1.88616300

C 6.95376600 -1.06480500 -0.11657500

C 5.95661700 -0.96917800 0.85651700

C 4.30313700 -0.93731200 -0.91298500

H 5.03483500 -1.05857200 -2.94169000

H 7.99558400 -1.11696000 0.18555700

H 6.21170500 -0.94164300 1.91229400

F -1.26676000 -2.65619400 -0.04427100

H -0.04856200 -3.14638600 -0.05999400

F 0.92349000 -3.46949900 -0.08140100

H 2.37003500 -1.53925000 -1.77697900

H 2.52238300 0.20093400 -1.35905500

F -3.15509100 -3.77562300 0.93742700

H -2.34958400 -3.35442100 0.55246300

F 1.81913700 2.61750200 1.92410100

H 2.32821900 2.38928600 1.07132100

F 2.95296200 2.01942200 -0.03744300

Cl -4.36208300 -0.00283700 -2.73272700

F 5.27400200 2.45322500 -0.11159100

H 4.28604100 2.26176000 -0.07388100

C -3.37169700 -0.18595700 2.72366300

H -3.26663200 0.90466100 2.75854600

C -3.92023000 -0.70626100 4.05625800

H -4.09758800 -0.42062900 1.93117500

H -2.02659100 -1.90547700 2.38952200

H -3.24026600 -0.46113800 4.87919900

H -4.03854700 -1.79528100 4.02799100

H -4.89767000 -0.26510500 4.27681600

SCF Done: E(RM062X) = -2230.59632398 A.U. after 23 cycles

Zero-point correction= 0.486985 (Hartree/Particle)

Thermal correction to Energy= 0.511058

Thermal correction to Enthalpy= 0.511777

Thermal correction to Gibbs Free Energy= 0.435250
