# Supplementary material for: Diastereodivergent nucleophile–nucleophile alkene chlorofluorination
Source: Nat Chem. 2024 Jul 1;16(10):1647–55. doi: 10.1038/s41557-024-01561-6 (PMC11446824; doi:10.1038/s41557-024-01561-6)
Supplement: Supplementary file 3 — Eight files of xyz coordinates: 1,2_chloride_shift.docx Cartesian coordinates of model alkene forming anti-chlorofluoride through 1,2-chloride shift via chloronium cation. alkene_activation.docx Cartesian coordinates of I(III)–alkene complexes and complexation transition states. direct_chloronium_formation_transition_states.docx Cartesian coordinates of direct Cl+ delivery to alkene transition states. iodane_ligand_exchange.docx Cartesian coordinates of iodanes IF2, IFCl and ICl2 and ligand exchange transition states between them with different sites and extents of HF coordination. iodine(III)iranium_vs_iodine(III)-π_complex.docx Cartesian coordinates of iodine(III)iranium and iodine(III)–π complex with model homoallylic amine showing latter is favoured thermodynamically. isolated_fluoride_chloride_hf_clusters.docx Cartesian coordinates of fluoride and chloride with 0–6 HF coordinated to anions. ligand_coupling_transition_states.docx Cartesian coordinates of ligand coupling of fluoride or chloride from C–I(III) intermediates. syn-1,2-halo-λ3-iodanation.docx Cartesian coordinates of alkene syn-difunctionalisation to form C–I(III) and C–X (X = F or Cl). [file 41557_2024_1561_MOESM3_ESM.zip › Calculations archive/Ligand coupling transition state.docx]

### Ligand coupling transition state

#### LC_CCl_-INT-1

H -0.59448700 1.19251600 1.90983000

C 0.41861400 0.92766200 2.22398900

H 0.77754600 1.70945800 2.90528200

H 0.39551800 -0.01768700 2.77119400

C 1.38094100 0.87112200 1.06142800

H 2.37230500 0.50089600 1.33511500

C 1.51543500 2.17588200 0.30324800

H 1.89449800 2.88810800 1.04948700

C 2.39722800 2.18976500 -0.92091200

H 1.97230500 1.57633900 -1.72114800

H 3.38713100 1.79794600 -0.66943200

H 2.49997100 3.21659800 -1.28206800

I 0.78851400 -0.81784400 -0.27429900

Cl 3.94548300 -1.15731600 -0.06603000

H -1.30120900 0.93419300 -1.81916500

C -1.89782000 0.39701900 -1.08844300

C -3.43551200 -0.98274800 0.78907000

C -3.27591500 0.58397800 -1.03088200

C -1.30227400 -0.48524400 -0.18919300

C -2.05482100 -1.18404400 0.74855600

C -4.06313900 -0.09899900 -0.09343900

H -3.74829700 1.27273500 -1.72748400

H -1.58202400 -1.87526500 1.43982400

H -4.02934700 -1.52507000 1.52035300

C -5.55126900 0.12471600 -0.04607700

H -5.77786600 1.16085500 0.22898700

H -6.02796600 -0.53462400 0.68397300

H -6.00475900 -0.05926800 -1.02579200

F 0.23482100 2.62974600 -0.05363200

SCF Done: E(RM062X) = -1285.82880080 A.U. after 22 cycles

Zero-point correction= 0.235365 (Hartree/Particle)

Thermal correction to Energy= 0.246460

Thermal correction to Enthalpy= 0.247179

Thermal correction to Gibbs Free Energy= 0.201564

#### LC_CCl_-INT-2

C 1.16038000 1.09835800 0.64878100

H 2.21437700 1.17944000 0.36828200

C 0.35806700 2.28529400 0.15874000

H -0.71790700 2.10233600 0.25629600

C 0.75706400 3.55975300 0.87900700

H 1.84230300 3.69911600 0.85299400

H 0.42080900 3.53463100 1.91844300

H 0.27450600 4.40591300 0.38199100

I 0.62774000 -0.67732300 -0.59900100

Cl 3.90237400 -0.64419300 -0.69043900

H -1.81248600 0.60482300 -2.04851600

C -2.24983700 0.13558700 -1.17230400

C -3.37103100 -1.06681700 1.08536300

C -3.62069800 0.20931400 -0.94112300

C -1.45351000 -0.54679100 -0.25411800

C -1.99446800 -1.15566000 0.87332600

C -4.20003600 -0.38867700 0.18623800

H -4.25090300 0.73995400 -1.65094900

H -1.36333200 -1.69116400 1.57607800

H -3.80307900 -1.53823700 1.96428000

C -5.68744100 -0.31026100 0.40440400

H -6.03675600 0.72573700 0.34546500

H -5.96798500 -0.71551700 1.38003100

H -6.21943300 -0.87849700 -0.36696000

F 0.61309900 2.45830200 -1.20973500

C 0.94784100 0.76096300 2.10504200

H 1.46688800 -0.15990400 2.37847400

H 1.37039200 1.57421400 2.70688700

H -0.11680000 0.67043300 2.34593200

F 3.34685600 -1.73827500 1.90065300

H 3.57124600 -1.37050900 1.01738400

SCF Done: E(RM062X) = -1386.30599825 A.U. after 19 cycles

Zero-point correction= 0.246700 (Hartree/Particle)

Thermal correction to Energy= 0.259350

Thermal correction to Enthalpy= 0.260069

Thermal correction to Gibbs Free Energy= 0.210380

#### LC_CCl_-INT-3

C -1.36445700 0.87329400 0.91579600

H -0.47103800 1.17720500 1.46673600

C -1.86536100 1.93810900 -0.02461300

H -2.64348300 1.54604000 -0.68460500

C -2.35128400 3.14429900 0.75145900

H -1.56601200 3.52264200 1.41339500

H -3.22518600 2.87165700 1.35026300

H -2.64142500 3.93319900 0.05179800

I -0.73724400 -0.91499300 -0.14983500

Cl -3.87296600 -1.11116800 -0.05119100

H 1.57773000 -1.36100400 1.89498000

C 2.05803800 -0.84358300 1.06983600

C 3.29985200 0.47639600 -1.05125400

C 3.41964500 -0.54212300 1.12587500

C 1.33432700 -0.46781400 -0.05650800

C 1.93959800 0.18786900 -1.12582700

C 4.05727700 0.11988500 0.07238700

H 3.99057000 -0.82933000 2.00510400

H 1.36471300 0.47592900 -2.00060600

H 3.78011100 0.99126100 -1.88007600

C 5.52686500 0.44222900 0.12901400

H 5.95691400 0.16362700 1.09456000

H 5.69685500 1.51258600 -0.02857700

H 6.07150900 -0.09386200 -0.65611500

F -0.79481400 2.33264400 -0.84764800

H -2.15059900 0.50937900 1.57647300

SCF Done: E(RM062X) = -1246.51840458 A.U. after 21 cycles

Zero-point correction= 0.207218 (Hartree/Particle)

Thermal correction to Energy= 0.217506

Thermal correction to Enthalpy= 0.218225

Thermal correction to Gibbs Free Energy= 0.173542

#### LC_CCl_-INT-4

C 1.08370400 0.90857600 -1.16260600

H 0.16397900 1.21944300 -1.66424400

C 1.63431800 1.96377700 -0.23847700

H 2.45434700 1.57346200 0.36963600

C 2.06344300 3.18389300 -1.02626200

H 1.23370100 3.56895600 -1.62715800

H 2.89622100 2.92551500 -1.68678000

H 2.39699400 3.96198100 -0.33396700

I 0.49811000 -0.89242500 -0.08540600

H -1.99378000 -1.36975300 -1.89743500

C -2.39665800 -0.84821900 -1.03467600

C -3.43492200 0.47990700 1.19189400

C -3.75742100 -0.56525000 -0.95283500

C -1.57044100 -0.44866900 0.01306300

C -2.06714300 0.20962700 1.13133200

C -4.29511500 0.09999300 0.15730100

H -4.41290300 -0.87027100 -1.76519500

H -1.41032000 0.51176600 1.94093900

H -3.83436200 0.99582400 2.06110700

C -5.77071500 0.39066100 0.22444300

H -6.08113800 1.01862700 -0.61770600

H -6.03070900 0.90704400 1.15205200

H -6.35151800 -0.53656300 0.17205400

F 0.61439600 2.33787500 0.65305100

H 1.83129200 0.54881500 -1.86914100

F 3.48433500 -0.15699300 2.27966200

H 3.59994200 -0.48172500 1.36191200

Cl 3.72444300 -1.09107700 -0.42841500

SCF Done: E(RM062X) = -1346.99543017 A.U. after 22 cycles

Zero-point correction= 0.218285 (Hartree/Particle)

Thermal correction to Energy= 0.230102

Thermal correction to Enthalpy= 0.230821

Thermal correction to Gibbs Free Energy= 0.182879

#### LC_CF_-INT-1

H 0.62320200 0.97299500 2.37234800

C 1.65712800 0.97279400 2.01059900

H 2.14348900 1.89383900 2.35171700

H 2.19448600 0.13005500 2.45089600

C 1.71758800 0.92117700 0.49958300

H 2.73833600 0.88522100 0.11512800

C 0.89732900 1.98991900 -0.18924500

H -0.16755100 1.89242300 0.04824900

C 1.39838300 3.38338400 0.14068400

H 2.47796300 3.45877600 -0.02379300

H 1.17408500 3.63724000 1.17975600

H 0.88664800 4.10213600 -0.50553400

I 1.06069500 -1.07647300 -0.18042000

F 3.46529400 -1.25418300 -0.10027300

H -1.17227500 -0.10355900 -2.15960900

C -1.69255100 -0.19299700 -1.21002100

C -3.03409800 -0.40001400 1.22356300

C -3.04861900 0.11215700 -1.12617800

C -1.00749000 -0.60157300 -0.06463600

C -1.67520700 -0.71324100 1.15259300

C -3.73842700 0.01615500 0.09003700

H -3.58095600 0.43145400 -2.01953400

H -1.14875600 -1.04370700 2.04387500

H -3.55284200 -0.48657300 2.17518200

C -5.20587100 0.34767800 0.15971600

H -5.78669400 -0.32260600 -0.48349700

H -5.39144200 1.37097100 -0.18425800

H -5.58484400 0.25456900 1.18091400

F 1.01485600 1.80609200 -1.57747600

SCF Done: E(RM062X) = -925.456080754 A.U. after 22 cycles

Zero-point correction= 0.236190 (Hartree/Particle)

Thermal correction to Energy= 0.246878

Thermal correction to Enthalpy= 0.247598

Thermal correction to Gibbs Free Energy= 0.204070

#### LC_CF_-INT-2

C 1.42161000 0.93113800 0.59792800

H 2.49126400 0.85005000 0.39002900

C 0.82759500 2.17746800 -0.02444700

H -0.26762900 2.14759300 0.00006200

C 1.34862200 3.43669200 0.64261500

H 2.44200800 3.42651700 0.69292600

H 0.94198800 3.53395900 1.65220400

H 1.02485600 4.30160500 0.05693500

I 0.73824900 -0.83701800 -0.56257700

H -1.60511600 0.60393000 -2.02252300

C -2.07076500 0.19228500 -1.13172200

C -3.26862200 -0.86623400 1.15629900

C -3.42966300 0.38134400 -0.89587900

C -1.32016800 -0.52969900 -0.20400200

C -1.90510100 -1.06884000 0.93829500

C -4.04705300 -0.14185000 0.24824400

H -4.02083200 0.94374800 -1.61486400

H -1.31503900 -1.63818600 1.65030000

H -3.73111800 -1.28386100 2.04690600

C -5.51844900 0.07619700 0.48097400

H -5.74391900 1.14528500 0.56117200

H -5.85201500 -0.41449000 1.39899300

H -6.10640400 -0.31925400 -0.35425100

F 1.19839700 2.21676700 -1.37706300

C 1.07194200 0.73987700 2.05558200

H 1.42532900 -0.22430000 2.42656500

H 1.57622100 1.52343800 2.63270500

H -0.00618900 0.82506500 2.22777900

F 3.42013300 -0.92241800 -0.69681700

F 3.96200300 -1.49383600 1.45747400

H 3.73493900 -1.23402900 0.43599900

SCF Done: E(RM062X) = -1025.94793776 A.U. after 22 cycles

Zero-point correction= 0.246619 (Hartree/Particle)

Thermal correction to Energy= 0.258853

Thermal correction to Enthalpy= 0.259572

Thermal correction to Gibbs Free Energy= 0.210134

#### LC_CF_-INT-3

C 1.00374100 1.37044500 0.52393200

H 2.09565200 1.36403200 0.47918600

C 0.41724000 2.36734300 -0.45434200

H -0.66530000 2.23157500 -0.55420600

C 0.74921500 3.79374700 -0.05796000

H 1.82283800 3.90712200 0.12252000

H 0.19959200 4.08092400 0.84190000

H 0.45009100 4.46054100 -0.87141900

I 0.62795600 -0.68406300 -0.24552600

H -1.71834800 0.18384900 -2.09234400

C -2.21149200 -0.12666300 -1.17596500

C -3.47721400 -0.93563300 1.17788600

C -3.59770700 -0.06664700 -1.06421700

C -1.47174400 -0.59046700 -0.08885600

C -2.08636100 -1.00431900 1.08873500

C -4.24923700 -0.46685600 0.11031700

H -4.18329100 0.29570900 -1.90586700

H -1.50031000 -1.37208300 1.92542900

H -3.96577000 -1.25482400 2.09485700

C -5.74982600 -0.39400200 0.20567600

H -6.09877400 0.63308100 0.05322600

H -6.10217400 -0.73528700 1.18239400

H -6.21774700 -1.01540400 -0.56552900

F 0.97205200 2.12661600 -1.71966900

C 0.45357100 1.48516500 1.92624600

H 0.81189600 0.67465600 2.56431000

H 0.81012400 2.42880300 2.35489000

H -0.64167500 1.49619800 1.92942500

F 3.41140800 -0.43510400 -0.14395300

F 3.54370500 -0.49714300 2.21361600

H 3.52627400 -0.48556600 1.20678700

F 3.82123500 -2.48527400 -1.25265700

H 3.67853300 -1.61250800 -0.77741900

SCF Done: E(RM062X) = -1126.42872288 A.U. after 20 cycles

Zero-point correction= 0.258988 (Hartree/Particle)

Thermal correction to Energy= 0.272687

Thermal correction to Enthalpy= 0.273406

Thermal correction to Gibbs Free Energy= 0.221400

#### LC_CF_-INT-4

C -1.62601500 0.68921900 0.90870900

H -0.73674000 1.08658000 1.40349600

C -2.26055500 1.68682400 -0.02556300

H -3.03557700 1.21430800 -0.63326700

C -2.79774500 2.87883400 0.73829900

H -2.00760900 3.34207600 1.33767100

H -3.60917000 2.56175500 1.39974100

H -3.19162900 3.61693600 0.03380100

I -0.93324700 -1.06091900 -0.14552000

F -3.30651400 -1.31640000 -0.04878300

H 1.40250100 -1.20452400 1.94861200

C 1.85122700 -0.69978600 1.09715000

C 3.01973500 0.58287700 -1.08156600

C 3.18464300 -0.30098000 1.14840900

C 1.10106400 -0.44469900 -0.05134700

C 1.68062700 0.19252700 -1.14408200

C 3.78810600 0.34608100 0.06175400

H 3.76791100 -0.49869300 2.04514000

H 1.09964900 0.39204500 -2.04024400

H 3.47032200 1.08071300 -1.93666100

C 5.22937800 0.77711100 0.13777400

H 5.87560100 -0.06365300 0.41134600

H 5.36235600 1.55274900 0.90043400

H 5.57370500 1.17886100 -0.81899800

F -1.26968200 2.14193300 -0.91762800

H -2.34622700 0.29170700 1.62196700

SCF Done: E(RM062X) = -886.146574283 A.U. after 20 cycles

Zero-point correction= 0.207806 (Hartree/Particle)

Thermal correction to Energy= 0.217698

Thermal correction to Enthalpy= 0.218417

Thermal correction to Gibbs Free Energy= 0.175897

#### LC_CF_-INT-5

C -1.30063300 0.78552500 1.11531700

H -0.39496600 1.16002300 1.59838200

C -1.91781300 1.78530800 0.17191100

H -2.71830500 1.33204900 -0.41798600

C -2.40564200 3.00228700 0.93007500

H -1.59148900 3.45052800 1.50818200

H -3.21447900 2.71838400 1.60934700

H -2.79040500 3.74040600 0.22074700

I -0.64430400 -1.00339400 0.07589500

H 1.85045600 -1.22367100 1.94246400

C 2.23164600 -0.72975300 1.05378000

C 3.21901800 0.52786800 -1.23386300

C 3.57298900 -0.36547600 0.97266400

C 1.39414500 -0.44935400 -0.02448800

C 1.87037600 0.17447700 -1.17193500

C 4.08583500 0.26532100 -0.16888200

H 4.23330000 -0.57869900 1.81009400

H 1.20902400 0.38899100 -2.00577400

H 3.59794200 1.01763000 -2.12717500

C 5.54294000 0.63795200 -0.23950000

H 5.82781000 1.26210400 0.61403400

H 5.76743100 1.18642300 -1.15797000

H 6.17274200 -0.25829100 -0.21367300

F -0.92722400 2.19442500 -0.73958300

H -2.02156700 0.40629900 1.83791900

F -3.28062300 -1.15127900 0.40860000

F -4.20781700 -0.42654800 -1.56185100

H -3.79554900 -0.77662200 -0.63600800

SCF Done: E(RM062X) = -986.637289748 A.U. after 20 cycles

Zero-point correction= 0.218325 (Hartree/Particle)

Thermal correction to Energy= 0.229700

Thermal correction to Enthalpy= 0.230420

Thermal correction to Gibbs Free Energy= 0.183641

#### LC_CF_-INT-6

C 1.00546400 0.97433600 -1.17210600

H 0.07754300 1.16579700 -1.71628800

C 1.47605300 2.16585000 -0.37935700

H 2.31832200 1.90625100 0.26640500

C 1.81671800 3.31616400 -1.30372000

H 0.96045100 3.57003600 -1.93634900

H 2.66442200 3.04465400 -1.93936700

H 2.09425800 4.19018700 -0.70793700

I 0.51729600 -0.70098100 0.12109600

H -1.85207600 -1.70994500 -1.63131100

C -2.33015100 -1.08318300 -0.88486800

C -3.56070900 0.51825300 1.04353600

C -3.71206700 -0.91441300 -0.88505200

C -1.58014300 -0.43330800 0.09298000

C -2.17387200 0.36448700 1.06393300

C -4.34611200 -0.11487100 0.07548200

H -4.30774800 -1.41608400 -1.64404300

H -1.57759500 0.86095100 1.82285400

H -4.03465400 1.14174600 1.79713900

C -5.84311000 0.04405800 0.05947000

H -6.18231200 0.43659900 -0.90506300

H -6.17810300 0.72537000 0.84573900

H -6.33597200 -0.92271000 0.21011500

F 0.42731700 2.56501000 0.46766200

H 1.78436300 0.57763800 -1.82196400

F 3.26410800 -0.48060900 -0.14049900

F 3.81144100 0.20544900 2.05818400

H 3.60828100 -0.09833500 1.12399800

F 3.76891100 -2.68929300 -0.82122900

H 3.58569800 -1.74941400 -0.52087700

SCF Done: E(RM062X) = -1087.11773253 A.U. after 21 cycles

Zero-point correction= 0.230321 (Hartree/Particle)

Thermal correction to Energy= 0.243489

Thermal correction to Enthalpy= 0.244208

Thermal correction to Gibbs Free Energy= 0.191552

#### LC_CCl_-TS-1

C 2.65520600 1.32669900 0.46003200

H 3.49577900 1.05251400 -0.17668600

C 1.59028800 2.13361300 -0.12447900

H 0.59789200 1.89505800 0.26685800

C 1.98845200 3.57979100 0.25905800

H 3.01426600 3.80834900 -0.04088100

H 1.86160900 3.74169300 1.33089300

H 1.30102500 4.22771500 -0.29179100

I 0.36970500 -1.23934700 -0.12631400

Cl 3.74845100 -1.44725000 -0.29052900

H -1.53166700 0.43952900 -1.94658900

C -2.14617400 0.21362500 -1.07960100

C -3.73866100 -0.36404200 1.12877000

C -3.45686700 0.68192300 -1.01276800

C -1.63354600 -0.54897500 -0.02879400

C -2.42597000 -0.83953200 1.07848500

C -4.27400100 0.40214400 0.08981300

H -3.85191000 1.27384000 -1.83593600

H -2.03402400 -1.43557300 1.89737900

H -4.35405500 -0.59803300 1.99444600

C -5.68899200 0.91771700 0.14194200

H -5.70744100 2.01330600 0.12951900

H -6.20023400 0.57927400 1.04741200

H -6.26373000 0.57264700 -0.72441900

F 1.57854700 2.01981400 -1.50053800

C 2.73999200 1.08061600 1.88329100

H 3.05454400 0.03867900 2.03076100

H 3.60049200 1.67411500 2.24581300

H 1.83413900 1.32575500 2.43720800

SCF Done: E(RM062X) = -1285.79370367 A.U. after 20 cycles

Zero-point correction= 0.231553 (Hartree/Particle)

Thermal correction to Energy= 0.243120

Thermal correction to Enthalpy= 0.243839

Thermal correction to Gibbs Free Energy= 0.196281

#### LC_CCl_-TS-2

C 2.09576700 1.67440200 0.63053700

H 3.09557300 1.66633300 0.19761700

C 1.04586800 2.34775300 -0.12102900

H 0.05654700 1.89731500 -0.00002700

C 1.06804200 3.77643300 0.48155900

H 2.07259200 4.20678500 0.46299600

H 0.67626500 3.76954500 1.49998200

H 0.41109900 4.37060100 -0.15987700

I 0.30518800 -1.17385900 -0.53081600

Cl 3.77983600 -0.63639100 -0.63403100

H -1.78724700 0.71084400 -1.87854900

C -2.32657700 0.29223700 -1.03344600

C -3.72919800 -0.78516600 1.11688900

C -3.64704200 0.66731300 -0.79223100

C -1.71033500 -0.62915000 -0.18527400

C -2.40565000 -1.17073700 0.89194900

C -4.36918500 0.13705700 0.28436200

H -4.12517400 1.38372100 -1.45711800

H -1.92998900 -1.89040100 1.55145200

H -4.26975200 -1.21462500 1.95716200

C -5.79694600 0.55350600 0.52630900

H -5.86521800 1.63139300 0.71083200

H -6.21964600 0.03307100 1.39008400

H -6.42221100 0.33184600 -0.34544700

F 1.35867100 2.41763900 -1.46285100

C 1.91046400 1.22923800 1.99265400

H 2.37557200 0.23707200 2.10579000

H 2.54340400 1.88250300 2.62181200

H 0.87395600 1.23255700 2.32993600

F 3.28471500 -1.78796500 1.92388500

H 3.48031900 -1.43973500 1.02217000

SCF Done: E(RM062X) = -1386.27156801 A.U. after 21 cycles

Zero-point correction= 0.243076 (Hartree/Particle)

Thermal correction to Energy= 0.256017

Thermal correction to Enthalpy= 0.256736

Thermal correction to Gibbs Free Energy= 0.205730

#### LC_CCl_-TS-3

C 2.63468700 1.35613600 -0.22950300

H 2.57578000 1.12608800 -1.29028100

C 1.63943700 2.20103700 0.39109200

H 1.45944500 2.00610000 1.44989600

C 2.32307500 3.59123100 0.21165300

H 2.62627700 3.77188200 -0.82324200

H 3.17585000 3.68934100 0.88379500

H 1.55090100 4.31583800 0.48698700

I 0.65797900 -1.07167900 -0.02446200

Cl 3.95208800 -1.21196300 0.01798700

H -1.48241900 -0.18923200 -2.13918500

C -2.02714200 -0.18175700 -1.19971200

C -3.43886500 -0.16018100 1.20084200

C -3.37456100 0.17113300 -1.17630900

C -1.38891100 -0.52591100 -0.00794800

C -2.08762300 -0.51380500 1.19560800

C -4.10189800 0.18705300 0.02098100

H -3.87050700 0.43766000 -2.10738500

H -1.59333500 -0.78207600 2.12468500

H -3.98247200 -0.15656300 2.14268100

C -5.55977500 0.56737400 0.02388900

H -5.70069400 1.58228900 -0.36393400

H -5.97616300 0.52744800 1.03424000

H -6.14186800 -0.10843700 -0.61230500

F 0.44517100 2.19982200 -0.29507600

H 3.58372200 1.22974400 0.27686700

SCF Done: E(RM062X) = -1246.46588719 A.U. after 20 cycles

Zero-point correction= 0.203981 (Hartree/Particle)

Thermal correction to Energy= 0.214525

Thermal correction to Enthalpy= 0.215244

Thermal correction to Gibbs Free Energy= 0.169370

#### LC_CCl_-TS-4

C 2.18994800 1.19162800 -1.21785300

H 1.40500300 0.95767900 -1.93292700

C 1.93023300 2.08043800 -0.12232100

H 2.49472200 1.88619900 0.79269400

C 2.43250000 3.40657000 -0.79405300

H 2.01155000 3.56055200 -1.79153200

H 3.52166000 3.42762000 -0.82493000

H 2.06021500 4.19154800 -0.12880800

I 0.32003000 -1.16564300 -0.04168900

H -2.20032100 -1.36506100 -1.89109200

C -2.54921400 -0.79261800 -1.03687700

C -3.46251200 0.66722100 1.15222900

C -3.86980600 -0.35148200 -0.98352600

C -1.68784900 -0.49665100 0.01931500

C -2.13577100 0.23287600 1.11656900

C -4.34776500 0.38407400 0.10845600

H -4.54118500 -0.58551400 -1.80708600

H -1.46525400 0.46342500 1.93897900

H -3.81028400 1.23563900 2.01159700

C -5.77949800 0.85209900 0.14545800

H -5.99234100 1.52675500 -0.69125400

H -5.99781000 1.38391100 1.07556600

H -6.47023600 0.00561100 0.06547200

F 0.58968800 2.19810500 0.15927900

H 3.22002900 0.97558800 -1.47638900

F 3.39060600 0.23203900 2.14274100

H 3.51889900 -0.30303200 1.33159600

Cl 3.71409000 -1.20576800 -0.32382000

SCF Done: E(RM062X) = -1346.94340221 A.U. after 22 cycles

Zero-point correction= 0.215112 (Hartree/Particle)

Thermal correction to Energy= 0.227240

Thermal correction to Enthalpy= 0.227959

Thermal correction to Gibbs Free Energy= 0.178537

#### LC_CF_-TS-1

C 3.07374600 0.59181500 0.39529200

H 3.67528100 0.10771300 -0.37135300

C 2.30462600 1.78216400 0.03796400

H 1.34579300 1.85424600 0.55700600

C 3.23520000 2.94714600 0.44581000

H 4.23359800 2.83338500 0.01499000

H 3.30115800 3.02290300 1.53273900

H 2.77317500 3.85342000 0.04502800

I 0.39724600 -1.20190500 -0.25694000

H -1.21307200 1.16735200 -1.52238000

C -1.91879800 0.72308300 -0.82548700

C -3.74907700 -0.40996400 0.93602900

C -3.19854700 1.25977200 -0.69638200

C -1.55279900 -0.38820600 -0.06349300

C -2.46844300 -0.95536200 0.81983500

C -4.13429200 0.70371800 0.18484500

H -3.47575100 2.12475000 -1.29570500

H -2.19490500 -1.82265900 1.41398700

H -4.45892200 -0.86346600 1.62416200

C -5.51212000 1.30031200 0.31534800

H -5.46419000 2.30442400 0.75212700

H -6.15073200 0.68392000 0.95428600

H -5.99478700 1.39360600 -0.66333900

F 2.08664400 1.84166700 -1.32613600

C 3.25257100 0.18658000 1.77560400

H 3.20490800 -0.90884100 1.80573100

H 4.30119700 0.42499800 2.03125600

H 2.56537900 0.65977400 2.47737800

F 3.08062400 -1.79509900 -0.41722900

SCF Done: E(RM062X) = -925.417271267 A.U. after 19 cycles

Zero-point correction= 0.232108 (Hartree/Particle)

Thermal correction to Energy= 0.243338

Thermal correction to Enthalpy= 0.244057

Thermal correction to Gibbs Free Energy= 0.198253

#### LC_CF_-TS-2

C 2.82504800 1.14466200 0.43780500

H 3.61022500 0.87387400 -0.26570800

C 1.87326000 2.16773800 0.02398800

H 0.86297000 2.00884400 0.40846300

C 2.48538900 3.46111100 0.61800600

H 3.53865700 3.57253100 0.34732900

H 2.37049800 3.47393100 1.70305100

H 1.91337600 4.28278900 0.17825100

I 0.25097500 -1.03628500 -0.49851100

H -1.69101100 1.13320000 -1.62647500

C -2.28326200 0.63087800 -0.86673900

C -3.81983600 -0.65381300 1.06555100

C -3.59298200 1.03827800 -0.62090000

C -1.74385800 -0.42782000 -0.13476500

C -2.50777000 -1.07323000 0.83332100

C -4.38215300 0.40488700 0.34737800

H -4.00948500 1.86283700 -1.19586500

H -2.09338500 -1.89921800 1.40361500

H -4.41283600 -1.16419500 1.82100900

C -5.79586800 0.86157400 0.59967500

H -5.81470800 1.89560200 0.96189800

H -6.28748400 0.23150300 1.34601400

H -6.38910100 0.82796100 -0.32055000

F 1.82891100 2.27877100 -1.35144100

C 2.88753900 0.64592800 1.78916700

H 3.00993400 -0.45161700 1.74073900

H 3.85383600 0.98208100 2.20735600

H 2.04993600 0.94191200 2.42021300

F 3.18153800 -1.13806200 -0.79135000

F 3.55871300 -2.36699600 1.09578700

H 3.39140900 -1.81349000 0.15985000

SCF Done: E(RM062X) = -1025.91336878 A.U. after 20 cycles

Zero-point correction= 0.242586 (Hartree/Particle)

Thermal correction to Energy= 0.255172

Thermal correction to Enthalpy= 0.255891

Thermal correction to Gibbs Free Energy= 0.205901

#### LC_CF_-TS-3

C -2.46004000 1.62514400 -0.62934500

H -3.32555100 1.42874900 0.00048200

C -1.37696900 2.40645400 -0.05541100

H -0.38612200 2.13315300 -0.42529300

C -1.74896000 3.84407200 -0.50966200

H -2.78015800 4.09920500 -0.25223600

H -1.58201400 3.96392900 -1.58134300

H -1.07069100 4.49988800 0.04365000

I -0.18539700 -1.01635000 0.12062400

H 1.74728200 0.80709400 1.75971600

C 2.38517600 0.38945500 0.98593100

C 4.03689000 -0.68441100 -0.98113000

C 3.73400800 0.73511600 0.93004500

C 1.86659800 -0.49996200 0.04417600

C 2.68659300 -1.03995100 -0.94227700

C 4.58124500 0.20554400 -0.05144000

H 4.13511000 1.42741900 1.66743400

H 2.28684000 -1.73532100 -1.67418100

H 4.67463300 -1.11220700 -1.75112400

C 6.03758400 0.59028600 -0.09239500

H 6.15398800 1.66826500 -0.25063800

H 6.56117000 0.06952600 -0.89884000

H 6.53446900 0.34407700 0.85234400

F -1.39104500 2.34961600 1.32175200

C -2.52081500 1.29784600 -2.03509600

H -2.81090500 0.23924600 -2.11282400

H -3.38743100 1.83708200 -2.45657100

H -1.60621700 1.51105600 -2.58736200

F -3.20461000 -0.72006300 0.13958800

F -3.38323200 -2.48198600 -1.42608600

H -3.31956000 -1.75322300 -0.73728500

F -3.35791500 -1.12595800 2.47012200

H -3.29543600 -0.97341900 1.48223600

SCF Done: E(RM062X) = -1126.39311206 A.U. after 20 cycles

Zero-point correction= 0.254843 (Hartree/Particle)

Thermal correction to Energy= 0.269233

Thermal correction to Enthalpy= 0.269952

Thermal correction to Gibbs Free Energy= 0.214260

#### LC_CF_-TS-4

C 2.84025100 0.93861500 -0.18499600

H 2.92250100 0.73223000 -1.24691800

C 1.86329800 1.93751000 0.26887000

H 1.52214500 1.79251400 1.29549100

C 2.63474800 3.26499200 0.13405700

H 3.04956600 3.38663800 -0.87040600

H 3.43293700 3.32213400 0.87622800

H 1.91156500 4.06428100 0.31962700

I 0.88210700 -1.19126000 0.02370500

H -1.34287300 -0.65237800 -2.12185800

C -1.82390100 -0.40401500 -1.18017600

C -3.07215100 0.24134100 1.22338500

C -3.14362000 0.04096500 -1.16818100

C -1.12878700 -0.52967900 0.02341700

C -1.74821100 -0.20412400 1.22730300

C -3.78861200 0.37176500 0.03092100

H -3.68230300 0.13442800 -2.10892800

H -1.21224200 -0.29959200 2.16738900

H -3.55170200 0.49048800 2.16715300

C -5.21545400 0.85563300 0.02146000

H -5.30834000 1.78749500 -0.54746200

H -5.57751900 1.03928500 1.03679100

H -5.87598400 0.11928700 -0.44924200

F 0.75959700 1.98227700 -0.56720000

H 3.72615400 0.82444800 0.42311100

F 3.54363500 -1.16922500 0.05901100

SCF Done: E(RM062X) = -886.091407674 A.U. after 18 cycles

Zero-point correction= 0.205174 (Hartree/Particle)

Thermal correction to Energy= 0.215157

Thermal correction to Enthalpy= 0.215876

Thermal correction to Gibbs Free Energy= 0.172848

#### LC_CF_-TS-5

C 2.41599500 0.86655700 -1.23975000

H 1.53629300 0.82188300 -1.87646200

C 2.43976800 1.74539700 -0.09571900

H 2.98800900 1.33948100 0.76075400

C 3.17801500 2.98979900 -0.66713900

H 2.72431400 3.34572600 -1.59586900

H 4.23454000 2.76593500 -0.81940300

H 3.07148600 3.76062000 0.10100400

I 0.38924800 -1.19409900 -0.00342300

H -2.18062300 -1.43625200 -1.77561900

C -2.48132600 -0.77222300 -0.97078000

C -3.27028800 0.92768400 1.08896100

C -3.77768800 -0.26304400 -0.93306300

C -1.58071300 -0.42500900 0.03635200

C -1.96751200 0.42505900 1.06831200

C -4.19349100 0.59326300 0.09448700

H -4.47892600 -0.53769400 -1.71825800

H -1.26748200 0.69707100 1.85264100

H -3.56889300 1.59092400 1.89736300

C -5.60205500 1.12761000 0.11973800

H -5.83336600 1.66808300 -0.80461600

H -5.75163200 1.80987600 0.96102200

H -6.32744300 0.31138100 0.21025400

F 1.16684500 2.11767400 0.29060600

H 3.35518000 0.48091500 -1.61434200

F 3.23785700 -1.22669600 -0.41996900

F 4.06291200 -0.42253700 1.56721900

H 3.69563300 -0.83726300 0.65385100

SCF Done: E(RM062X) = -986.584978553 A.U. after 20 cycles

Zero-point correction= 0.215546 (Hartree/Particle)

Thermal correction to Energy= 0.227004

Thermal correction to Enthalpy= 0.227723

Thermal correction to Gibbs Free Energy= 0.180569

#### LC_CF_-TS-6

C 2.14225900 1.29769400 -1.25127100

H 1.30189100 1.03152200 -1.88832700

C 1.97712500 2.25766000 -0.19857000

H 2.58582200 2.08321300 0.69233000

C 2.48361900 3.53179400 -0.95520900

H 2.00964500 3.65753000 -1.93258200

H 3.56883000 3.50818100 -1.05249700

H 2.18214600 4.36039200 -0.30790500

I 0.30604300 -0.98577200 0.16990900

H -2.17570700 -1.76700600 -1.57321300

C -2.56931500 -1.03715400 -0.87224700

C -3.59530100 0.82962100 0.92236300

C -3.91566300 -0.68082500 -0.91590600

C -1.73961300 -0.45077500 0.08323400

C -2.24392300 0.48287400 0.98358200

C -4.45049400 0.25570700 -0.02224600

H -4.56224300 -1.14139600 -1.65987600

H -1.59797700 0.93930500 1.72758400

H -3.98648300 1.55979700 1.62685500

C -5.91182900 0.61839000 -0.07898700

H -6.19740400 0.94453000 -1.08480900

H -6.14672800 1.42379600 0.62229100

H -6.53736000 -0.24518500 0.17379300

F 0.65841300 2.43679400 0.15006700

H 3.14638800 1.04190600 -1.56611300

F 3.24355800 -0.55441000 -0.17439200

F 3.59606100 0.28820000 2.01114300

H 3.46543300 -0.11499500 1.10340300

F 3.63289200 -2.81419400 -0.82228200

H 3.48395100 -1.87307800 -0.53540200

SCF Done: E(RM062X) = -1087.06407648 A.U. after 20 cycles

Zero-point correction= 0.227420 (Hartree/Particle)

Thermal correction to Energy= 0.240742

Thermal correction to Enthalpy= 0.241461

Thermal correction to Gibbs Free Energy= 0.188321
