# Supplementary material for: Diastereodivergent nucleophile–nucleophile alkene chlorofluorination
Source: Nat Chem. 2024 Jul 1;16(10):1647–55. doi: 10.1038/s41557-024-01561-6 (PMC11446824; doi:10.1038/s41557-024-01561-6)
Supplement: Supplementary file 3 — Eight files of xyz coordinates: 1,2_chloride_shift.docx Cartesian coordinates of model alkene forming anti-chlorofluoride through 1,2-chloride shift via chloronium cation. alkene_activation.docx Cartesian coordinates of I(III)–alkene complexes and complexation transition states. direct_chloronium_formation_transition_states.docx Cartesian coordinates of direct Cl+ delivery to alkene transition states. iodane_ligand_exchange.docx Cartesian coordinates of iodanes IF2, IFCl and ICl2 and ligand exchange transition states between them with different sites and extents of HF coordination. iodine(III)iranium_vs_iodine(III)-π_complex.docx Cartesian coordinates of iodine(III)iranium and iodine(III)–π complex with model homoallylic amine showing latter is favoured thermodynamically. isolated_fluoride_chloride_hf_clusters.docx Cartesian coordinates of fluoride and chloride with 0–6 HF coordinated to anions. ligand_coupling_transition_states.docx Cartesian coordinates of ligand coupling of fluoride or chloride from C–I(III) intermediates. syn-1,2-halo-λ3-iodanation.docx Cartesian coordinates of alkene syn-difunctionalisation to form C–I(III) and C–X (X = F or Cl). [file 41557_2024_1561_MOESM3_ESM.zip › Calculations archive/Iodine(III)iranium vs iodine(III)-╧Ç complex.docx]

### Iodine(III)iranium *vs* iodine(III)-π complex

#### IF2-2HF

C -1.08283500 -0.01574600 -0.44633100

C 0.71470400 -0.63694400 1.57387600

C -0.41701300 -1.23886300 -0.49326900

C -0.88279000 0.90437000 0.57732400

C 0.02334200 0.57930600 1.58550200

C 0.48037900 -1.53791700 0.52606200

H -0.58881000 -1.94109200 -1.30282100

H -1.41218900 1.85158800 0.59461300

H 0.19238700 1.28690100 2.39261600

H 1.00948900 -2.48728300 0.50515800

I -2.44729700 0.44099700 -1.96294500

C 1.70622300 -0.97728700 2.65309600

H 2.72718800 -0.94705800 2.25557900

H 1.53423200 -1.98808900 3.03630700

H 1.64493300 -0.27267900 3.48640600

F -3.84364100 -0.44518900 -0.91978400

F -0.72062400 1.34768200 -2.89816600

F 0.17427200 3.40275300 -1.77899900

F 0.96070600 -0.30165900 -3.74022400

H -0.12511600 2.60512600 -2.22674100

H 0.35250000 0.37109200 -3.41724800

SCF Done: E(RM062X) = -969.171776083 A.U. after 19 cycles

Zero-point correction= 0.145475 (Hartree/Particle)

Thermal correction to Energy= 0.155701

Thermal correction to Enthalpy= 0.156417

Thermal correction to Gibbs Free Energy= 0.112843

#### 40a-onium

H -4.09673500 -0.03166700 -1.91231300

C -3.72326400 -0.64802900 -1.09917800

C -2.75507700 -2.22811900 1.03237800

C -2.38443800 -0.60666600 -0.72915900

C -4.58057000 -1.49443000 -0.39196900

C -4.10139300 -2.27689100 0.66241500

C -1.90659200 -1.38558100 0.32412600

H -5.63255600 -1.53747700 -0.65862700

H -4.78446200 -2.92297100 1.20617500

H -2.38405700 -2.82996200 1.85755000

C -1.25931300 0.18934000 -1.33017100

H -1.48503300 1.23944400 -1.52726400

H -0.85852900 -0.27712800 -2.23622700

C -0.42938200 -1.16147200 0.50353600

H -0.11026000 -1.02499200 1.53798700

H 0.17155400 -1.94867800 0.03386500

N -0.20241200 0.11629200 -0.25936400

C 1.18449500 0.30325000 -0.77887400

H 1.18107300 1.24475000 -1.33580900

H 1.38442800 -0.52065700 -1.47101100

C 2.20720400 0.34342000 0.35300600

H 1.89405100 1.09254500 1.09081200

H 2.24352400 -0.62543400 0.86063600

C 3.55975900 0.71744200 -0.19721900

H 3.72306400 1.78340800 -0.35356600

C 4.53558600 -0.13647200 -0.52745900

H 5.46637800 0.28184400 -0.91416700

C 4.51353300 -1.63419100 -0.39672200

H 4.80049500 -2.07489300 -1.36023100

H 3.50345700 -1.99486700 -0.17420300

C 5.48778200 -2.12005000 0.68261000

H 5.19696800 -1.73717100 1.66697200

H 6.50596000 -1.77251500 0.47452200

H 5.50679100 -3.21382200 0.73147700

H -0.39606000 0.91418800 0.38788700

F -0.64006600 2.40432500 1.08708900

H -1.91149600 2.82959600 0.72154800

F -2.82263200 3.10755900 0.43296200

H 0.38235500 3.12814300 0.49808400

F 1.13225300 3.60088900 0.04078600

SCF Done: E(RM062X) = -901.063990856 A.U. after 10 cycles

Zero-point correction= 0.341639 (Hartree/Particle)

Thermal correction to Energy= 0.354993

Thermal correction to Enthalpy= 0.355713

Thermal correction to Gibbs Free Energy= 0.303544

#### 40a-IF2

C 4.89862135 -2.50197718 -0.69496205

C 5.26598438 -1.29102209 -0.09579801

C 4.40160532 -0.19938001 -0.08662401

C 3.15683123 -0.34114302 -0.69211405

C 2.75671720 -1.52563511 -1.30378210

C 3.63962026 -2.60145519 -1.29967509

H 6.24151944 -1.19780309 0.37406003

H 4.69562934 0.73467905 0.38234403

H 1.77852913 -1.61616612 -1.76671913

H 3.33822024 -3.53556126 -1.76671113

I 1.84620813 1.29629710 -0.75620905

C 5.82255039 -3.68845426 -0.65909705

H 5.56783140 -4.33597331 0.18848701

H 5.73397141 -4.28636831 -1.57065411

H 6.86400551 -3.37701524 -0.54142704

C 1.42118210 0.84980306 1.85822413

C 0.52019304 0.02367000 1.23393909

C 1.10360608 2.15979715 2.51207818

C -0.95279407 0.27115502 1.13901908

H -1.19242808 1.33128109 1.25932709

H -1.39236310 -0.27728902 1.98411214

C -1.53061311 -0.27345402 -0.16103101

N -3.00465722 -0.05957200 -0.21885002

F 2.67826019 1.80010613 -2.48839818

H 0.26286302 2.65183419 2.01430114

C 0.77381606 1.92380614 3.99422629

H 1.97136414 2.82228820 2.42939017

H 1.60564811 1.43276810 4.50923032

H -0.11425701 1.29258909 4.10079129

H 0.57981004 2.87990921 4.48914132

C -3.78424927 -0.68457605 0.91199807

C -3.62476526 -0.54281804 -1.50614611

H -1.35257010 -1.34832810 -0.25357902

H -3.78036827 -0.00586600 1.76655412

H -1.11262108 0.24233802 -1.03065107

H -3.15541323 0.96701207 -0.19047501

H 0.86254206 -0.96660207 0.92996806

H 2.42057617 0.44591503 2.02791514

H -3.28515324 -1.62176912 1.18243208

H -8.28066857 -1.39820810 -1.88671913

C -7.38990355 -1.26101709 -1.28089409

C -5.13582237 -0.89975106 0.28932802

C -6.16778944 -0.98975307 -1.90128314

C -7.47930853 -1.34988910 0.11120201

C -6.34900243 -1.16835109 0.91197306

C -5.04688236 -0.81153806 -1.09917608

H -6.09825243 -0.91628907 -2.98293822

H -8.43875260 -1.55576611 0.57647904

H -6.41883245 -1.23286409 1.99418614

F -0.50883604 2.92727821 -0.44072803

H -1.76731912 2.82642620 -0.49858104

F -2.80476120 2.67141619 -0.53380404

H -3.49398525 0.22756502 -2.26831816

H -3.10254822 -1.45616110 -1.80958313

F 0.53252704 4.95320536 0.29566302

H 0.06986501 4.13298030 -0.01203500

F -1.38875910 -2.80129020 1.80358213

H -0.49076704 -2.98360321 1.33845610

F 2.53268918 -2.59036919 1.99782914

H 1.73524713 -2.86983220 1.44478410

F 0.64051305 -3.16934323 0.71130405

SCF Done: E(RM062X) = -1870.24010292 A.U. after 21 cycles

Zero-point correction= 0.487253 (Hartree/Particle)

Thermal correction to Energy= 0.511536

Thermal correction to Enthalpy= 0.512253

Thermal correction to Gibbs Free Energy= 0.433916

#### 40a-IF^+^

H 3.90378000 0.06372000 2.09393300

C 3.61947500 -0.54438300 1.23986800

C 2.88202900 -2.10679300 -0.99620500

C 2.30451900 -0.57633300 0.79179600

C 4.57000100 -1.30770900 0.55719400

C 4.20537600 -2.08067900 -0.54876800

C 1.94089100 -1.34708900 -0.31197800

H 5.60523900 -1.29249900 0.88443900

H 4.96038500 -2.65992400 -1.07195000

H 2.60034400 -2.70037200 -1.86147100

C 1.09626200 0.12476000 1.34804300

H 1.24180900 1.17671900 1.60077200

H 0.66513000 -0.40486700 2.20423800

C 0.46537200 -1.20952700 -0.57239900

H 0.19711100 -1.06899300 -1.62081900

H -0.11007000 -2.04160700 -0.15114000

N 0.12076600 0.03412000 0.20188600

C -1.30220900 0.14950100 0.62219200

H -1.39290100 1.10723000 1.14346700

H -1.50945300 -0.66234700 1.32494800

C -2.23496800 0.09509100 -0.58991700

H -1.84860800 0.75944400 -1.37133600

H -2.27350600 -0.92029900 -0.99565600

C -3.59320500 0.58342800 -0.17261100

H -3.68234200 1.66238700 -0.04325400

C -4.64666200 -0.19973300 0.21697400

H -5.53314600 0.31877000 0.58682300

C -4.67956700 -1.69129000 0.31680500

H -3.82334100 -2.14566100 -0.18953700

H -5.59084500 -2.06093000 -0.16755300

C -4.70127900 -2.10521900 1.79660100

H -5.56619300 -1.67440100 2.31091900

H -3.79583300 -1.77258600 2.31340500

H -4.76401000 -3.19410700 1.87643900

H 0.31061300 0.86291000 -0.40584000

F 0.27185200 2.43369800 -0.98288100

H 1.26131000 3.13535700 -0.28207900

F 1.96708700 3.59398800 0.24317400

H -0.99632100 2.92246100 -0.84503500

F -1.95305900 3.21061600 -0.73781200

F -5.75935000 0.81624400 -4.22037300

C -7.50211200 1.66866100 -1.24858400

C -6.21807900 2.00796400 -1.65884000

C -5.72115900 3.30711100 -1.58966600

C -6.55655800 4.29688100 -1.08252200

C -7.85950300 4.00167100 -0.65687000

C -8.31744300 2.68238200 -0.74835000

H -7.86386300 0.64703800 -1.31178200

H -4.71327600 3.54501200 -1.91433600

H -6.18799100 5.31747200 -1.01758900

H -9.32545700 2.43907400 -0.42382700

I -4.99039500 0.49709700 -2.44038700

C -8.74012700 5.09302800 -0.11314500

H -9.71531800 4.70332000 0.18863200

H -8.27261200 5.56933300 0.75531400

H -8.89657900 5.87198200 -0.86711600

SCF Done: E(RM062X) = -1569.25124384 A.U. after 21 cycles

Zero-point correction= 0.465441 (Hartree/Particle)

Thermal correction to Energy= 0.485265

Thermal correction to Enthalpy= 0.485985

Thermal correction to Gibbs Free Energy= 0.419670
