# Supplementary material for: Diastereodivergent nucleophile–nucleophile alkene chlorofluorination
Source: Nat Chem. 2024 Jul 1;16(10):1647–55. doi: 10.1038/s41557-024-01561-6 (PMC11446824; doi:10.1038/s41557-024-01561-6)
Supplement: Supplementary file 3 — Eight files of xyz coordinates: 1,2_chloride_shift.docx Cartesian coordinates of model alkene forming anti-chlorofluoride through 1,2-chloride shift via chloronium cation. alkene_activation.docx Cartesian coordinates of I(III)–alkene complexes and complexation transition states. direct_chloronium_formation_transition_states.docx Cartesian coordinates of direct Cl+ delivery to alkene transition states. iodane_ligand_exchange.docx Cartesian coordinates of iodanes IF2, IFCl and ICl2 and ligand exchange transition states between them with different sites and extents of HF coordination. iodine(III)iranium_vs_iodine(III)-π_complex.docx Cartesian coordinates of iodine(III)iranium and iodine(III)–π complex with model homoallylic amine showing latter is favoured thermodynamically. isolated_fluoride_chloride_hf_clusters.docx Cartesian coordinates of fluoride and chloride with 0–6 HF coordinated to anions. ligand_coupling_transition_states.docx Cartesian coordinates of ligand coupling of fluoride or chloride from C–I(III) intermediates. syn-1,2-halo-λ3-iodanation.docx Cartesian coordinates of alkene syn-difunctionalisation to form C–I(III) and C–X (X = F or Cl). [file 41557_2024_1561_MOESM3_ESM.zip › Calculations archive/Ligand exchange.docx]

### Ligand exchange

#### Chloride

Cl 0.00000000 0.00000000 0.00000000

SCF Done: E(RM062X) = -460.355413773 A.U. after 7 cycles

Zero-point correction= 0.000000 (Hartree/Particle)

Thermal correction to Energy= 0.001079

Thermal correction to Enthalpy= 0.001798

Thermal correction to Gibbs Free Energy= -0.010956

#### Chloride-1HF

F 0.00000000 0.00000000 1.82633500

H 0.00000000 0.00000000 0.83700900

Cl 0.00000000 0.00000000 -1.01611900

SCF Done: E(RM062X) = -560.837783047 A.U. after 10 cycles

Zero-point correction= 0.010877 (Hartree/Particle)

Thermal correction to Energy= 0.012979

Thermal correction to Enthalpy= 0.013698

Thermal correction to Gibbs Free Energy= -0.005633

#### Chloride-2HF

Cl 0.00000000 0.93241800 0.00000000

F 2.27944600 -0.85214500 0.00000000

F -2.27944300 -0.85227200 0.00000000

H 1.50980600 -0.25561400 0.00000000

H -1.50983900 -0.25573000 0.00000000

SCF Done: E(RM062X) = -661.313645002 A.U. after 10 cycles

Zero-point correction= 0.021740 (Hartree/Particle)

Thermal correction to Energy= 0.025342

Thermal correction to Enthalpy= 0.026062

Thermal correction to Gibbs Free Energy= 0.000143

#### Fluoride-2HF

F -2.00989400 -0.40726400 0.00000100

H -1.12873900 0.10838000 -0.00001700

F 2.00988500 -0.40727100 -0.00000200

H 1.12873800 0.10840700 0.00003000

F 0.00000900 0.79044800 -0.00000100

SCF Done: E(RM062X) = -300.961878309 A.U. after 9 cycles

Zero-point correction= 0.022118 (Hartree/Particle)

Thermal correction to Energy= 0.025182

Thermal correction to Enthalpy= 0.025902

Thermal correction to Gibbs Free Energy= 0.002145

#### IF2-0HF

C 0.00344700 -0.44374100 0.00107200

C 0.00742100 -3.22525800 -0.00699200

C -1.21390200 -1.12077400 -0.02328600

C 1.22018000 -1.11623000 0.02233000

C 1.20926200 -2.51093900 0.01707700

C -1.19883800 -2.51231400 -0.02533500

H -2.15447400 -0.57942900 -0.03857200

H 2.15986700 -0.57353100 0.04315500

H 2.15381000 -3.04822000 0.03393900

H -2.14172300 -3.05358500 -0.04239500

C -0.00248700 -4.73047700 -0.02485300

H -0.37380800 -5.10110700 -0.98702000

H -0.66181400 -5.12604100 0.75453800

H 1.00070100 -5.13530700 0.13158900

I -0.00001100 1.65194400 0.00716400

F 0.16064900 1.50401600 -1.99770100

F -0.16160300 1.49249000 2.01114600

SCF Done: E(RM062X) = -768.230313875 A.U. after 14 cycles

Zero-point correction= 0.122261 (Hartree/Particle)

Thermal correction to Energy= 0.129202

Thermal correction to Enthalpy= 0.129921

Thermal correction to Gibbs Free Energy= 0.094780

#### IF2-1HF

C 0.51566200 -0.26448000 0.00281200

C 3.28616100 -0.05548400 -0.00291800

C 1.13355800 0.46321300 -1.01129000

C 1.23897300 -0.89624000 1.00795700

C 2.62861500 -0.78650500 0.99207000

C 2.52155400 0.56219200 -1.00152400

H 0.55166400 0.94736500 -1.78911000

H 0.73947900 -1.46059400 1.78899500

H 3.20707100 -1.27626500 1.77093400

H 3.01798400 1.13006200 -1.78448300

I -1.57352300 -0.40803200 0.01963000

C 4.78422000 0.08835200 0.00322000

H 5.06822900 1.10023200 0.31487500

H 5.19836700 -0.07460500 -0.99660900

H 5.24803100 -0.62096900 0.69360000

F -1.37873100 -2.11402800 -0.97004300

F -1.47278300 1.39590200 1.06272500

F -1.04612100 3.44660400 -0.23562200

H -1.20781900 2.65788600 0.30593000

SCF Done: E(RM062X) = -868.702050933 A.U. after 19 cycles

Zero-point correction= 0.133729 (Hartree/Particle)

Thermal correction to Energy= 0.142299

Thermal correction to Enthalpy= 0.143015

Thermal correction to Gibbs Free Energy= 0.103259

#### TS­_IF2-IFCl_-0HF

I 0.98627100 -1.00983000 -0.14276200

Cl 3.16563800 0.47996700 1.31901300

F 0.33386000 -2.78350100 -0.65911400

F -0.29004800 2.69785800 1.14610000

H 0.23456700 2.21502500 0.44686500

F 0.91655100 1.52138700 -0.53340900

F 2.68537700 2.61320300 -1.70637700

H 1.98348000 2.15629400 -1.16853800

C -5.15458500 0.67131700 0.13244400

C -2.97213700 0.47322100 -1.11127100

C -3.69924700 0.29325300 0.07607000

C -3.04533800 -0.22935800 1.19475400

C -1.62501700 0.14324900 -1.18808600

H -3.46900100 0.88493900 -1.98644200

C -1.01107800 -0.38304300 -0.05255700

C -1.69403500 -0.57173100 1.14316500

H -3.59244400 -0.36798600 2.12335100

H -1.19119800 -0.96968000 2.01904900

H -1.06414300 0.30100700 -2.10322400

H -5.72283000 0.14441400 -0.64167000

H -5.27992500 1.74536400 -0.04377800

H -5.58974100 0.43021600 1.10564700

SCF Done: E(RM062X) = -1429.52414446 A.U. after 21 cycles

Zero-point correction= 0.144967 (Hartree/Particle)

Thermal correction to Energy= 0.156193

Thermal correction to Enthalpy= 0.156912

Thermal correction to Gibbs Free Energy= 0.110851

#### TS­_IF2-IFCl_-1HF

I 0.84096100 1.30040300 0.02776300

Cl 2.66944900 -0.61711800 -1.64293100

F 0.08543700 3.07274000 0.24248400

F -0.25127900 -2.77297400 -0.17004300

H 0.30409400 -2.09342800 0.29759200

F 1.05479500 -1.12432300 0.97071800

F 0.62138700 -0.78901200 3.26782100

H 0.80815100 -0.96866400 2.29458200

C -4.99905500 -1.16581100 -0.48411800

C -3.05109900 -0.36102400 0.89516500

C -3.62062000 -0.57509800 -0.36936500

C -2.87865100 -0.25100100 -1.50945700

C -1.76789000 0.15318300 1.02851500

H -3.61662400 -0.61360500 1.78855700

C -1.06463700 0.47140900 -0.13317900

C -1.59225200 0.27482800 -1.40490000

H -3.30514600 -0.41630600 -2.49506900

H -1.01831700 0.51562100 -2.29425800

H -1.32368100 0.29373300 2.00878700

H -5.37246300 -1.10707600 -1.50958900

H -5.70272500 -0.64810900 0.17562300

H -4.98521000 -2.22044800 -0.18591900

F 3.48499500 -2.86925600 -0.00532700

H 3.14754700 -2.10558800 -0.50877800

SCF Done: E(RM062X) = -1530.00148507 A.U. after 24 cycles

Zero-point correction= 0.156180 (Hartree/Particle)

Thermal correction to Energy= 0.169013

Thermal correction to Enthalpy= 0.169733

Thermal correction to Gibbs Free Energy= 0.119627

#### TS­_IF2-IFCl_-2HF

I 0.77694200 1.08893900 0.53808400

Cl 1.88571000 -0.86368900 -1.69982000

F 0.21746900 2.85472400 1.06659100

F -0.66594800 -2.92458700 -0.23550600

H -0.07723800 -2.33848000 0.30388300

F 0.70570200 -1.53980200 1.10074700

F 0.02134700 -1.31463100 3.31856500

H 0.32370900 -1.44721400 2.36812200

C -5.11874900 -0.49456600 -1.21558500

C -3.30068500 -0.30806600 0.51464400

C -3.72531200 -0.10536300 -0.80652900

C -2.82867100 0.44161300 -1.73148900

C -2.00932000 0.01323600 0.91188300

H -3.98673400 -0.73800200 1.23930700

C -1.14816600 0.55967000 -0.03989400

C -1.52961300 0.77817200 -1.36066300

H -3.14401400 0.59880000 -2.75880300

H -0.83462800 1.18956300 -2.08524600

H -1.67511200 -0.16992300 1.92826800

H -5.34772300 -0.14932100 -2.22599200

H -5.85729800 -0.07646200 -0.52515900

H -5.22809100 -1.58403000 -1.19224800

F 3.11613100 -3.02749500 -0.11745000

H 2.64453000 -2.32940900 -0.56209900

F 3.79558100 1.30185500 -2.00106400

H 3.19813900 0.55467400 -1.93268400

SCF Done: E(RM062X) = -1630.47538821 A.U. after 20 cycles

Zero-point correction= 0.166649 (Hartree/Particle)

Thermal correction to Energy= 0.178940

Thermal correction to Enthalpy= 0.179659

Thermal correction to Gibbs Free Energy= 0.131113

#### IFCl-0HF

C 0.55130000 -0.13190900 0.00000000

C 3.31719800 0.10153400 -0.00000100

C 1.21828000 -0.07189800 1.21883800

C 1.21827700 -0.07187600 -1.21883700

C 2.60601100 0.04563500 -1.20470700

C 2.60601400 0.04561300 1.20470700

H 0.67614900 -0.11028900 2.15830500

H 0.67614400 -0.11025000 -2.15830400

H 3.14232600 0.09626700 -2.14875500

H 3.14233200 0.09622800 2.14875400

C 4.81932400 0.19242300 0.00000000

H 5.25936200 -0.81202600 0.00003300

H 5.18406600 0.71460700 0.88888500

H 5.18407100 0.71455300 -0.88891500

I -1.53602000 -0.35439600 0.00000000

F -1.21660100 -2.32192400 -0.00001000

Cl -1.70143500 2.26082800 0.00000700

SCF Done: E(RM062X) = -1128.59039174

Zero-point correction= 0.121189 (Hartree/Particle)

Thermal correction to Energy= 0.128611

Thermal correction to Enthalpy= 0.129330

Thermal correction to Gibbs Free Energy= 0.091941

#### IFCl-1HF

C -0.59147700 -0.16256400 0.08825800

C -3.35841300 -0.28990500 -0.00302600

C -1.24521700 0.33337300 -1.03611900

C -1.26740200 -0.72777700 1.16245100

C -2.65863100 -0.78769400 1.10159800

C -2.63452500 0.26532700 -1.06678600

H -0.69361800 0.76420200 -1.86553000

H -0.73398100 -1.11346700 2.02527600

H -3.20531200 -1.22709400 1.93160400

H -3.16391800 0.65132800 -1.93426700

C -4.86179300 -0.32894200 -0.05009300

H -5.21390800 -0.70587900 -1.01537000

H -5.27250100 0.67927200 0.07779200

H -5.26745600 -0.96351000 0.74196400

I 1.49944500 0.00030800 0.18178400

F 1.14134800 1.89801800 0.92004700

Cl 1.71684200 -2.38022100 -0.72984000

H 0.68279600 2.97289100 -0.06254500

F 0.40484800 3.61073900 -0.73159100

SCF Done: E(RM062X) = -1229.06243692 A.U. after 20 cycles

Zero-point correction= 0.132895 (Hartree/Particle)

Thermal correction to Energy= 0.141783

Thermal correction to Enthalpy= 0.142500

Thermal correction to Gibbs Free Energy= 0.102304

#### IFCl-1HF(Cl)

C 0.58182800 -0.35718800 0.02382700

C 3.34065900 -0.08763900 0.11045200

C 1.23478000 0.19352900 -1.07583100

C 1.25827700 -0.77596200 1.16315100

C 2.64445500 -0.63415000 1.19375200

C 2.61832200 0.32413700 -1.01786400

H 0.68622800 0.51858100 -1.95385500

H 0.72698900 -1.19857600 2.01003600

H 3.18938200 -0.95657800 2.07685900

H 3.14522600 0.75442100 -1.86592900

C 4.83848100 0.05180100 0.13971500

H 5.13676500 1.08345700 -0.07392000

H 5.29771000 -0.58624700 -0.62360300

H 5.24499300 -0.23239900 1.11358400

I -1.49576400 -0.62530500 -0.06897800

F -1.16407800 -2.37925800 -0.89895400

Cl -1.71920500 1.78936900 1.07630900

H -0.74778800 2.85967600 -0.38429100

F -0.31129400 3.28952500 -1.11921400

SCF Done: E(RM062X) = -1229.05622995 A.U. after 19 cycles

Zero-point correction= 0.132259 (Hartree/Particle)

Thermal correction to Energy= 0.141536

Thermal correction to Enthalpy= 0.142255

Thermal correction to Gibbs Free Energy= 0.100469

#### IFCl-2HF(Cl)

C -0.55811000 -0.54091500 0.04089400

C -3.31018000 -0.28831900 0.21937800

C -1.10411100 -0.00945600 1.20700500

C -1.33572700 -0.95449500 -1.03521000

C -2.71881400 -0.82436600 -0.92978200

C -2.48684200 0.11525700 1.28007700

H -0.47361600 0.30524200 2.03267200

H -0.88311000 -1.36323100 -1.93303400

H -3.34457300 -1.14198000 -1.75939300

H -2.93407500 0.53190700 2.17896200

C -4.80206000 -0.12489400 0.31861000

H -5.07199900 0.93566500 0.25740400

H -5.17362900 -0.50313900 1.27616200

H -5.31430400 -0.65350300 -0.48916500

I 1.51704400 -0.77779300 -0.07403600

F 1.33613900 -2.61538800 0.54990600

Cl 1.55539800 1.83682300 -0.92309100

H 1.56904100 2.57410300 1.03942200

F 1.56287400 2.73694100 1.97904600

F -1.43386500 2.41878000 -1.24585000

H -0.51010100 2.21207500 -1.14035300

SCF Done: E(RM062X) = -1329.52127591 A.U. after 22 cycles

Zero-point correction= 0.143189 (Hartree/Particle)

Thermal correction to Energy= 0.154263

Thermal correction to Enthalpy= 0.154982

Thermal correction to Gibbs Free Energy= 0.109857

#### TS­_IFCl-ICl2_-0HF

I 1.23081800 0.56544900 -0.07597000

Cl 3.36676600 -1.19667200 -0.77211300

F -1.62441500 -2.78816000 -0.87396700

H -0.73899600 -2.45949900 -0.53334200

F 0.48522600 -2.03334800 -0.09888800

F 1.03650300 -2.56802200 2.13627500

H 0.81007400 -2.37065000 1.17606700

C -5.13630800 0.06177500 -0.02260400

C -2.89275400 -0.08165800 1.11309700

C -3.63519600 0.16017300 -0.05273700

C -2.95516200 0.48572800 -1.22983800

C -1.50582900 -0.00243700 1.11241500

H -3.40937000 -0.34065500 2.03419300

C -0.86853200 0.33446800 -0.07957800

C -1.56328900 0.57555400 -1.25751200

H -3.51592200 0.67012100 -2.14230800

H -1.04248500 0.82744200 -2.17614400

H -0.93705900 -0.20052200 2.01574700

H -5.55515100 0.75460400 0.71548500

H -5.45020400 -0.94846400 0.26205400

H -5.56944800 0.29430000 -0.99882400

Cl 0.89451500 3.02659500 0.51936300

SCF Done: E(RM062X) = -1789.89282109 A.U. after 21 cycles

Zero-point correction= 0.143708 (Hartree/Particle)

Thermal correction to Energy= 0.155354

Thermal correction to Enthalpy= 0.156073

Thermal correction to Gibbs Free Energy= 0.108808

#### TS­_IFCl-ICl2_-1HF

I -0.87085100 -1.05729600 -0.00885700

Cl -3.12907400 0.58689500 -1.24963600

F 0.41881500 2.91753000 -0.42750100

H -0.16225400 2.29947500 0.09140000

F -0.96501400 1.40713200 0.80415700

F -0.59622700 1.21615600 3.13479900

H -0.75504900 1.33397000 2.15060000

C 5.07109500 1.19737500 -0.46349600

C 3.05415300 0.53861100 0.89292500

C 3.67156800 0.65614600 -0.36214900

C 2.96056400 0.27410500 -1.50366400

C 1.75645700 0.05991100 1.01523600

H 3.59566500 0.83537100 1.78769800

C 1.08561100 -0.31203000 -0.14817400

C 1.65781600 -0.21384000 -1.41086500

H 3.42310200 0.36486500 -2.48264900

H 1.10781400 -0.49988800 -2.30194100

H 1.27867000 -0.01280500 1.98699000

H 5.43394200 1.17103800 -1.49397200

H 5.75790200 0.61758100 0.16238500

H 5.10567000 2.23479600 -0.11264700

F -3.02518500 3.31306600 -0.20035800

H -2.96442000 2.38543100 -0.47822800

Cl -0.02980900 -3.38570400 0.26381500

SCF Done: E(RM062X) = -1890.36773996 A.U. after 23 cycles

Zero-point correction= 0.155321 (Hartree/Particle)

Thermal correction to Energy= 0.168475

Thermal correction to Enthalpy= 0.169194

Thermal correction to Gibbs Free Energy= 0.118214

#### TS­_IFCl-ICl2_-2HF

I 0.83069800 0.90830900 0.29535200

Cl 1.75514500 -1.51607900 -1.52496600

F -0.96496900 -3.02404400 0.36709800

H -0.30043200 -2.39813100 0.76324100

F 0.60025900 -1.52712400 1.38804100

F -0.05533500 -0.79575100 3.52779200

H 0.22914900 -1.14646200 2.62204700

C -5.15861900 -0.69292700 -1.10333600

C -3.32122000 -0.22522500 0.55108400

C -3.74026700 -0.30802800 -0.78509900

C -2.81982600 -0.03536200 -1.80362500

C -2.01421500 0.11464100 0.87491500

H -4.02521400 -0.44280500 1.35022100

C -1.13499800 0.38487200 -0.17168100

C -1.50409300 0.31477400 -1.51058900

H -3.12981600 -0.10347500 -2.84265800

H -0.79209000 0.51749700 -2.30411200

H -1.68704400 0.15732500 1.90917800

H -5.34208200 -0.68372600 -2.18055900

H -5.86268100 -0.00389600 -0.62430200

H -5.37580700 -1.69772000 -0.72457400

F 3.43221100 -2.66751100 0.61876300

H 2.80462300 -2.30970300 -0.01947500

F 3.56075900 0.51179600 -2.56895700

H 2.98420300 -0.19857400 -2.24500800

Cl 0.33152100 3.30293300 0.48600800

SCF Done: E(RM062X) = -1990.84211736 A.U. after 22 cycles

Zero-point correction= 0.166064 (Hartree/Particle)

Thermal correction to Energy= 0.181159

Thermal correction to Enthalpy= 0.181878

Thermal correction to Gibbs Free Energy= 0.125005

#### ICl-1HF

C -0.58223500 -0.26647000 0.12751400

C -3.34680700 -0.34377200 0.00919400

C -1.20627900 0.09510600 -1.06233500

C -1.28342900 -0.66095400 1.25935100

C -2.67479600 -0.69468000 1.18494300

C -2.59557600 0.05041500 -1.10672200

H -0.63402700 0.40104700 -1.93201400

H -0.76985000 -0.93679000 2.17478000

H -3.24234800 -1.00055800 2.05956700

H -3.10379400 0.32576600 -2.02748200

C -4.84871000 -0.38018700 -0.06809300

H -5.17769500 -1.05154000 -0.86852000

H -5.24700200 0.61534700 -0.29225800

H -5.28683200 -0.72307200 0.87254100

I 1.52261700 -0.22296100 0.20900900

Cl 1.48413200 2.22847800 0.90614100

Cl 1.50620900 -2.78669200 -0.51772200

H 0.61600800 -2.46912500 -2.36263400

F 0.23939300 -2.20341900 -3.19898000

SCF Done: E(RM062X) = -1589.42066423 A.U. after 20 cycles

Zero-point correction= 0.131160 (Hartree/Particle)

Thermal correction to Energy= 0.140896

Thermal correction to Enthalpy= 0.141615

Thermal correction to Gibbs Free Energy= 0.098086

#### TS_met_

C -1.66273900 -0.87451700 -0.23471600

C -4.36037700 -1.49692100 -0.48520400

C -2.29320200 -1.56152600 0.79593700

C -2.33901600 -0.48856100 -1.38910100

C -3.68895600 -0.80542000 -1.50282900

C -3.64651500 -1.86860600 0.65852800

H -1.74947700 -1.85245100 1.68942300

H -1.82916600 0.04787000 -2.18325800

H -4.22998300 -0.51161400 -2.39917000

H -4.15134300 -2.40654200 1.45657600

C -5.82346300 -1.82017400 -0.62881000

H -6.42173100 -0.90219100 -0.63920300

H -6.17389700 -2.44673800 0.19536500

H -6.01542100 -2.34609500 -1.56986900

I 0.38491000 -0.45056800 -0.07415600

F 0.70475500 -2.21538600 -0.94613700

C 1.07511100 4.74873600 1.53291200

C -1.24127200 6.26218500 1.55259500

C 0.33019300 4.81213700 2.71116500

C 0.70004100 5.40611100 0.36445100

C -0.46564600 6.16658400 0.39102300

C -0.82589600 5.57972100 2.70730700

H 0.63993800 4.27246300 3.60054900

H 1.29404800 5.32942000 -0.54050100

H -0.77747900 6.68976100 -0.50864700

H -1.42284500 5.64467700 3.61337500

C -2.50424600 7.07651000 1.57702100

H -2.44355600 7.85708200 2.34326900

H -3.36337300 6.44334800 1.82426700

H -2.69058000 7.55205200 0.61109400

I 2.80626300 3.57562400 1.50747400

Cl 4.25265700 5.39270500 2.27068300

Cl -0.31065500 1.81507900 1.04586000

F 2.68667900 1.12357300 0.67822800

F 2.87840700 0.00487400 2.79648600

H 2.81647000 0.44338200 1.91370800

F 4.52872700 0.99020000 -0.84537300

H 3.77296700 1.01717200 -0.20686200

SCF Done: E(RM062X) = -2458.11607774 A.U. after 22 cycles

Zero-point correction= 0.266001 (Hartree/Particle)

Thermal correction to Energy= 0.284821

Thermal correction to Enthalpy= 0.285540

Thermal correction to Gibbs Free Energy= 0.219698
